# Metabolic Alterations in Older Women With Low Bone Mineral Density Supplemented With *Lactobacillus reuteri*

**DOI:** 10.1002/jbm4.10478

**Published:** 2021-03-15

**Authors:** Peishun Li, Daniel Sundh, Boyang Ji, Dimitra Lappa, Lingqun Ye, Jens Nielsen, Mattias Lorentzon

**Affiliations:** ^1^ Department of Biology and Biological Engineering Chalmers University of Technology Gothenburg Sweden; ^2^ Geriatric Medicine, Department of Internal Medicine and Clinical Nutrition, Institute of Medicine, Sahlgrenska Academy University of Gothenburg Gothenburg Sweden; ^3^ Novo Nordisk Foundation Center for Biosustainability Technical University of Denmark Kgs. Lyngby Denmark; ^4^ BioInnovation Institute Copenhagen Denmark; ^5^ Region Västra Götaland, Geriatric Medicine Clinic Sahlgrenska University Hospital Mölndal Sweden; ^6^ Mary MacKillop Institute for Health Research Australian Catholic University Melbourne Victoria Australia

**Keywords:** BONE LOSS, *LACTOBACILLUS REUTERI*, METABOLOMICS, OSTEOPOROSIS, PROBIOTICS

## Abstract

Osteoporosis and its associated fractures are highly prevalent in older women. Recent studies have shown that gut microbiota play important roles in regulating bone metabolism. A previous randomized controlled trial (RCT) found that supplementation with *Lactobacillus reuteri* ATCC PTA 6475 (*L.reuteri*) led to substantially reduced bone loss in older women with low BMD. However, the total metabolic effects of *L. reuteri* supplementation on older women are still not clear. In this study, a post hoc analysis (not predefined) of serum metabolomic profiles of older women from the previous RCT was performed to investigate the metabolic dynamics over 1 year and to evaluate the effects of *L. reuteri* supplementation on human metabolism. Distinct segregation of the *L. reuteri* and placebo groups in response to the treatment was revealed by partial least squares‐discriminant analysis. Although no individual metabolite was differentially and significantly associated with treatment after correction for multiple testing, 97 metabolites responded differentially at any one time point between *L. reuteri* and placebo groups (variable importance in projection score >1 and *p* value <0.05). These metabolites were involved in multiple processes, including amino acid, peptide, and lipid metabolism. Butyrylcarnitine was particularly increased at all investigated time points in the *L. reuteri* group compared with placebo, indicating that the effects of *L. reuteri* on bone loss are mediated through butyrate signaling. Furthermore, the metabolomic profiles in a case (low BMD) and control population (high BMD) of elderly women were analyzed to confirm the associations between BMD and the identified metabolites regulated by *L. reuteri* supplementation. The amino acids, especially branched‐chain amino acids, showed association with *L. reuteri* treatment and with low BMD in older women, and may serve as potential therapeutic targets. © 2021 The Authors. *JBMR Plus* published by Wiley Periodicals LLC on behalf of American Society for Bone and Mineral Research.

## Introduction

Osteoporosis is a disease characterized by reduced BMD, deteriorated bone microarchitecture, and reduced bone strength, which increases the susceptibility to low energy or fragility fractures primarily affecting the older population.^(^ ^1^, ^2^ ^)^ Accumulating evidence has revealed that the gut microbiota is a key regulator of bone metabolism and could affect bone health.^(^ ^3^, ^4^, ^5^, ^6^ ^)^ Previous studies in mice have revealed that gut microbiota influences bone health by the regulation of the immune system and balance.^(^ ^7^ ^)^ The supplementation of probiotic strain *Lactobacillus reuteri* ATCC PTA 6475 (*L. reuteri*) has been shown to reduce bone loss and increase bone density in mice with estrogen deficiency or increased inflammation.^(^ ^8^, ^9^ ^)^ These data suggest that *L. reuteri* may be a potential therapeutic strategy to prevent postmenopausal bone loss.

In our previous study, we observed that supplementation with *L. reuteri* could reduce bone loss by approximately 50% in older women with low BMD.^(^ ^10^ ^)^ In mice, probiotic supplements affect bone metabolism via the production of short chain fatty acids in the intestine.^(^ ^7^ ^)^ However, the mechanisms related to the effect of the different probiotic supplementations on human metabolism remains unknown. An understanding of the dynamic metabolic changes incurred by probiotic supplementation may provide new insights into the regulation of bone metabolism and could be crucial for the development of novel osteoporosis treatments. Untargeted metabolomics have provided an opportunity to investigate the global metabolic changes in human populations.^(^ ^11^, ^12^ ^)^ Although the existing metabolomic studies related to osteoporosis have been performed in rats and humans,^(^ ^13^, ^14^, ^15^, ^16^ ^)^ the effects of probiotic supplementation on human bone metabolism have not been explored. Therefore, we investigated the effect of daily supplementation with *L. reuteri* or placebo on serum metabolomic profiles in older women who had been included in a 1‐year randomized controlled trial (RCT) in a nonpredefined post hoc analysis.^(^ ^10^ ^)^ We further examined differences in metabolic profiles between older women with severe osteoporosis (case group) and high bone density (control group).

In this study, we identified the key serum metabolic makers that were regulated by *L. reuteri* treatment and discriminated subjects with severe osteoporosis from controls. We found that amino acids and peptides not only were regulated by treatment with *L. reuteri*, but also differed in bone loss between the control and case group. Interestingly, butyrylcarnitine (C4) showed a robust increase in subjects who had had *L. reuteri* treatment. Thus, the present study's results suggest that the affected metabolic pathways by *L. reuteri* contribute to the regulation of bone loss in older women.

## Patients and Methods

### Study populations

As previously described,^(^ ^10^ ^)^ we studied the per protocol population in a placebo‐controlled cohort (68 elderly women with osteopenia who completed the study and had not used medication in violation of the study protocol), of whom 32 had been randomized to treatment with *L. reuteri* and 36 to placebo. In addition, we set‐up a separate cross‐sectional study to investigate case and matched controls of 240 elderly women, of whom 120 had high BMD and 120 had very low BMD. Information regarding previous fragility fractures was obtained using questionnaires. The case and control groups were selected from the population‐based SUPERB (Sahlgrenska University Hospital Prospective Evaluation of Risk of Bone Fractures) cohort (from 2013–2016) of 3028 women who were 75 to 80 years old,^(^ ^17^ ^)^ by identifying the 120 women with the lowest tibia total volumetric BMD (total vBMD; case group) and matching them to 120 women with the highest tibia volumetric BMD (control group) by age, height, weight, and BMI, using propensity score matching with R package MatchIt.^(^ ^18^ ^)^

### Measurements of bone traits, body composition, and serum markers

The lower leg (tibia) of the nondominant side (based on the dominant arm) was measured to determine bone microstructure and geometry, using HR‐pQCT (Xtreme CT; Scanco Medical AG). All measurement procedures were performed according to instructions and protocols from the manufacturer.^(^ ^19^ ^)^ A three‐dimensional construction of the bone was assembled using 110 slices obtained from each measurement. The nominal isotropic resolution was 82 μm. After image processing,^(^ ^20^ ^)^ the following variables were obtained: total volumetric BMD (total vBMD, mg/cm^3^), trabecular bone volume fraction (BV/TV, %), cortical thickness (Ct.Th, mm), and cortical volumetric BMD (Ct.vBMD, mg/cm^3^). Image quality was assessed and quality was graded from 1 to 5; a perfect quality was defined as 1 and a suboptimal quality was 5. Only images with a quality level between one and three were used for analysis. The coefficients of variation (CV) for the generated bone variables at the tibia ultradistal site were 0.2% for the tibia total vBMD for 0.5% for the BV/TV, 0.5% for Ct.Th, and 0.3% for the Ct.vBMD.

Areal BMD (aBMD, g/cm^2^) was measured at the lumbar spine (L1–L4), total hip, femoral neck, and body composition of the total body was determined with a Hologic Discovery A DEXA (Hologic). The CVs for aBMD were 0.7% for lumbar spine, 0.8% for total hip, and 1.3% for the femoral neck.^(^ ^10^ ^)^

N‐telopeptide of type I collagen (NTx; Osteomark; Alere Scarborough, Inc; intra‐ and interassay variability of 4.6 and 6.9%, respectively), bone‐specific alkaline phosphatase (BAP; Microvue; Quidel Corp; intra‐ and interassay variability of 5.0 and 5.9%, respectively), ultrasensitive C‐reactive protein (usCRP; BioMerica, Inc; intra‐ and interassay variability of 4.4 and 3.3%, respectively) and TNF‐α (Quantikine; R&D Systems, Inc; intra‐ and interassay variability of 2.0 and 6.7%, respectively) were measured in duplicate by TECO Medical AG.

### Serum sample preparation and metabolomic profiling

Serum samples were collected from the case and control subjects (240 elderly women) at fasting, as well as from women in the *L. reuteri* and placebo arms of the RCT (68 elderly women) at four time points: month 0 (baseline), 3, 6, and 12. Serum samples were frozen immediately after collection and stored in −80°C until analysis. The metabolomics analysis was performed blinded to study group. Metabolomics were performed using liquid chromatography‐tandem mass spectroscopy (LC–MS) at Metabolon, Inc, with their standard and platform method (Supplementary Materials and Methods).^(^ ^21^ ^)^ Peak intensities of all metabolites in original scale (provided by Metabolon; Supplementary Information Data Set) were analyzed. First, we used an imputation method drawing random values from the normal distribution to simulate signals from low abundant metabolites that were assumed to give rise to missing values.^(^ ^22^ ^)^ The width of the normal distribution was set as 0.5 times the SD of all measured values, and the center was downshifted by 2.5 times of the SD. Second, metabolites with <25% missing values across all samples were selected and analyzed further, which enabled the identification of important metabolites with high confidence. Finally, peak intensities of the filtered metabolites within sample *i* (*i* = 1, 2, 3…*n*; *n* is the total number of samples) were normalized to the total intensity. This normalization method considered the differences of sample volume used in LC–MS and assumes that all samples have equal total intensity.^(^ ^23^ ^)^ The normalization process included the following steps: (i) the total intensity of sample *i* was summed up; (ii) the correction factor of sample *i* was calculated by dividing the total intensity with the lowest total intensity of all samples; and (iii) peak areas of all metabolites within sample *i* were divided by its individual correction factor. The normalized metabolite abundance was log‐transformed if necessary. Three subjects (LB36, LB44, LB56) with incomplete serum samples obtained during follow‐up were excluded from metabolomics analysis.

**Table 1:** Baseline Characteristics of the *Lactobacillus reuteri 6475* Placebo‐Controlled Randomized Controlled Trial Cohort

Characteristics	*L. reuteri* (*N* = 32)	Placebo (*N* = 36)
Age, y	76.3 ± 0.9	76.2 ± 1.1
Height, cm	162.3 ± 4.8	164.0 ± 5.7
Weight, kg	67.0 ± 8.3	68.2 ± 10.5
BMI, kg/m^2^	25.5 ± 3.4	25.3 ± 3.5
BMD, *T* score		
Lumbar spine	−0.86 ± 0.98	−0.99 ± 0.91
Total hip	−1.05 ± 0.71	−1.18 ± 0.52
Femoral neck	−1.60 ± 0.62	−1.70 ± 0.64
HR‐pQCT–derived bone variables		
Total tibia volumetric BMD, mg/cm^3^	235 ± 42.2	231 ± 45.6
Trabecular bone volume fraction, %	12.2 ± 2.2	12.6 ± 2.4
Cortical volumetric BMD, mg/cm^3^	767 ± 67.0	740 ± 66.0
Cortical thickness, mm	0.81 ± 0.2	0.76 ± 0.3
Serum markers		
N‐terminal telopeptide, nM	14.3 ± 3.5	15.6 ± 8.0
Bone‐specific alkaline phosphatase, U/L	17.2 ± 4.2	18.0 ± 6.7
C‐reactive protein, mg/L^a^	1.34 (0.80–2.86)	1.36 (0.75 to 3.63)
TNF‐α, pg/mL	1.34 ± 0.4	1.30 ± 0.3
Body composition, kg		
Total fat mass	25.0 ± 5.9	25.8 ± 6.7
Total lean mass	42.4 ± 3.4	42.7 ± 5.2

Note: Mean ± SD. The characteristics of the per protocol population were calculated.^(^ ^10^ ^)^

^a^ Nonnormally distributed variables are presented as median with interquartile range.

**Table 2:** Clinical Characteristics of the Case and Control Groups

Characteristics	Case (Low BMD)	Control (High BMD)	*p* Value^d^
	(*N* = 120)	(*N* = 120)	
Age, y	78.14 ± 1.6	78.21 ± 1.72	0.75
Height, cm	1625.84 ± 64.99	1624.35 ± 57.32	0.85
Weight, kg	64.2 ± 11.07	64.92 ± 9.61	0.59
BMI, kg/m^2^	24.27 ± 3.83	24.6 ± 3.44	0.47
Total tibia volumetric BMD, mg/cm^3^	**157.4 ± 21.92**	**267.76 ± 29.58**	**<0.001**
BMD			
Lumbar spine (L1–L4), g/cm^2^	**0.82 ± 0.13**	**1.00 ± 0.16**	**<0.001**
Lumbar spine *T* score	**−2.04 ± 1.16**	**−0.39 ± 1.50**	**<0.001**
Total hip, g/cm^2^	**0.69 ± 0.1**	**0.87 ± 0.1**	**<0.001**
Total hip *T* score	**−2.07 ± 0.81**	**−0.62 ± 0.86**	**<0.001**
Femoral neck, g/cm^2^	**0.58 ± 0.09**	**0.72 ± 0.09**	**<0.001**
Femoral neck *T* score	**−2.31 ± 0.78**	**−1.19 ± 0.79**	**<0.001**
Fracture risk assessment tool score^a^	**34.15 ± 13.59**	**16.65 ± 8.16**	**<0.001**
Prevalent fracture, no. (%)^b^	111 (92.5)	0 (0)	**<0.001**
Physical health—physical component score^c^	45.52 ± 11.16	47.71 ± 9.5	0.1

Note: Mean ± SD.

^a^ Ten‐year probability of major osteoporotic fracture, calculated with femoral neck BMD.

^b^ Any clinical prevalent fracture after age 50 years.

^c^ Physical component score derived from the 12‐Item Short Form Health Survey.

^d^ *p* Values were derived from Student's *t* test for continuous variables or from Fisher's exact test for dichotomous variables. The characteristics significantly different (*p* < 0.001) between case and control groups are highlighted in bold.

**Table 3:** The Differential Pathways Between the Case and Control Groups

KEGG pathway	Total^a^	Hits^b^	Metabolites	*p* Value^c^	Adjusted *p* Value^d^	Impact^e^
Aminoacyl‐tRNA biosynthesis	75	5	Arginine, valine, lysine, leucine, glutamate	0.005	0.30	0.113
Valine, leucine, and isoleucine biosynthesis	27	3	Leucine, 3‐methyl‐2‐oxobutyrate, valine	0.007	0.30	0.115
Steroid hormone biosynthesis	99	5	Cortisol, DHEA sulfate, 5‐androstenediol, etiocholanolone glucuronide, androsterone glucuronide	0.016	0.36	0.042
Butanoate metabolism	40	3	Glutamate, maleate, fumarate	0.022	0.36	0.038
Valine, leucine, and isoleucine degradation	40	3	Leucine, valine, 3‐methyl‐2‐oxobutyrate	0.022	0.36	0.039
Arginine and proline metabolism	77	4	Arginine, glutamate, creatinine, fumarate	0.028	0.38	0.169
Citrate cycle (TCA cycle)	20	2	Malate, fumarate	0.036	0.42	0.060

^a^ It is the total number of metabolites present in the KEGG pathway.

^b^ It is the number of metabolites matched into the corresponding pathway.

^c^ The *p* values were calculated from the enrichment analysis.

^d^ The adjusted *p* values were obtained using false discovery rate correction.

^e^ The pathway impact values were calculated from a pathway topology analysis.

**Table 4:** The Differential Metabolites Between the Case (Low BMD) and Control Groups (High BMD) and Simultaneously Associated With the *Lactobacillus reuteri* Supplementation

Metabolites	Class	VIP score^a^	FC^b^	*p* Value^c^	Adjusted *p* Value^d^	R_3^e^	R_6^e^	R_12^e^
N,N,N‐trimethyl‐alanylproline betaine (TMAP)	Amino acid	1.31	0.93	0.031	0.41	Up	‐	‐
Valine	Amino acid	1.80	0.93	0.0049	0.24	‐	Up	‐
Cysteine S‐sulfate	Amino acid	1.17	0.85	0.049	0.48	Down	‐	Down
Isovalerylcarnitine (C5)	Amino acid	1.57	0.89	0.0071	0.28	Up	‐	‐
Deoxycarnitine	Lipid	1.48	0.94	0.026	0.41	‐	Up	‐
Sphingomyelin (d17:2/16:0, d18:2/15:0)*	Lipid	1.30	0.92	0.039	0.44	‐	Down	‐
Sphingomyelin (d18:1/22:2, d18:2/22:1, d16:1/24:2)*	Lipid	1.33	0.94	0.048	0.48	‐	Down	‐
Sphingomyelin (d18:2/21:0, d16:2/23:0)*	Lipid	1.63	0.91	0.0064	0.26	‐	Down	‐
Sphingomyelin (d18:2/23:1)*	Lipid	1.38	0.94	0.039	0.44	‐	Down	‐
Fibrinopeptide A (3‐15)	Peptide	1.63	0.84	0.0053	0.24	Down	‐	‐
Gamma‐glutamyl‐alpha‐lysine	Peptide	1.57	0.94	0.02	0.37	‐	Up	‐
Gamma‐glutamyl leucine	Peptide	1.37	0.94	0.043	0.46	Up	Up	‐

^a^ The variable importance in projection (VIP) scores were obtained from the partial least squares‐discriminant analysis model.

^b^ Fold change (FC) was calculated by dividing the mean value of metabolite levels in the case group with the control group.

^c^ The *p* values were derived from linear regression model, adjusted by for BMI and age.

^d^ The adjusted *p* values were obtained using false discovery rate correction.

^e^ R_3, R_6, R_12 represented differential responses to treatment with *L. reuteri* at 3, 6, and 12 months, respectively. Up and Down indicates upregulated and downregulated, respectively (*p* value < 0.05). “‐” indicates insignificantly differential responses.

### Statistical analysis

To evaluate the effects of *L. reuteri* on metabolites during the 1‐year intervention, we calculated the metabolite levels at different time points as a ratio of the baseline value, and then the ratios between *L. reuteri* and placebo groups were compared using the two‐tailed Wilcoxon rank‐sum test. In comparisons between the two groups at each time point, we performed 993 tests for the metabolites with <25% missing values obtained after data normalization. The *p* values were adjusted by the false discovery rate (FDR) or Bonferroni test to control for multiple‐comparison error using R function p.adjust (Supplemental Table 2). To distinguish metabolomic responses (namely relative changes from baseline) in the *L. reuteri* group from the placebo group, partial least squares‐discriminant analysis (PLS‐DA) were performed using the mixOmics package with default parameters.^(^ ^24^ ^)^ The response of a metabolite was considered to be the differential between the placebo and *L. reuteri* groups at any one time point when the metabolite was qualified by using the cut‐off of a variable importance in projection (VIP) score >1 in the PLS‐DA model and *p* value <0.05 in the Wilcoxon rank‐sum test.

To assess the associations between metabolites and clinical variables, we performed the Pearson's correlation analysis and hierarchical clustering with default parameters. The *p* values were adjusted by the FDR to correct for multiple testing. To identify different response patterns during the 1‐year intervention, a clustering analysis of the metabolites that responded differently between the *L. reuteri* and placebo groups was performed. The cluster::diana function was used based on a correlation coefficients matrix as a distance measure, and then by cutting the tree using the divisive coefficient of the clustering. For each cluster of metabolites, only the ones with a minimum cluster size of 10 metabolites were selected. To visualize the metabolic difference between the case group and the control group, a principal components analysis (PCA) and a PLS‐DA were performed. The fold changes of metabolites between the case and control groups were compared using linear regression models adjusted by covariates’ age and BMI. In total, we performed 1007 tests for the metabolites with <25% missing values obtained after data normalization. The *p* values were adjusted by the FDR or a Bonferroni test to control for multiple‐comparison error (Supplemental Table 4). Differential metabolites between the case and control groups were identified by using the VIP score cut‐off of >1 in PLS‐DA and *p* value <0.05 in the fold changes of metabolites.

To discriminate the BMD status of the case subjects from the controls, a random forest model was trained based on metabolite profiles using the randomForest package in R with default parameters and 1000 trees. To determine the contribution of metabolites to the classification models, the metric “mean decrease in accuracy” was calculated, and the performance of the predictive model was evaluated by the receiver operating characteristic (ROC) analysis and the area under the ROC curve (AUC).

Pathway analysis of the metabolites was performed using a web‐based tool MetaboAnalyst 4.0 (https://www.metaboanalyst.ca).^(^ ^25^ ^)^ The significant enrichment of pathways was determined by Fisher's exact test (*p* value <0.05).

## Results

### Global metabolic shifts in *L. reuteri* 6475 placebo‐controlled RCT cohort

**Fig 1:**
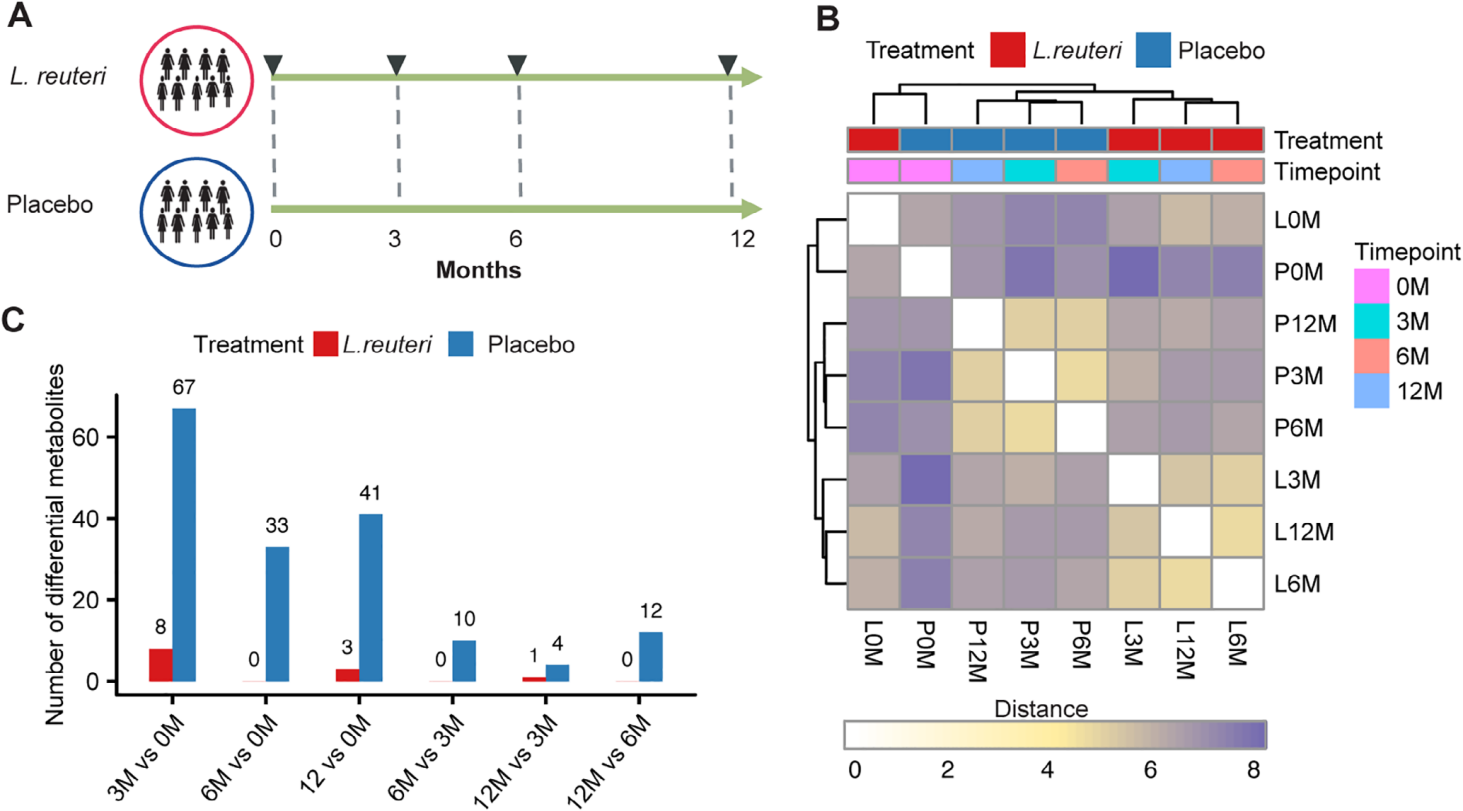
The metabolomic profiling of the cohort supplemented with placebo or *Lactobacillus reuteri*. (*A*) The scheme diagram of experimental design. Serum samples were collected from older women with bone loss at baseline, and 3, 6, and 12 months later. (*B*) The heatmap shows the hierarchical clustering of Euclidean distances between serum samples. (*C*) Numbers of significantly differential metabolites among all time points in the *L. reuteri* group and placebo group (adjusted *p* < 0.1 with false discovery rate by the Wilcoxon signed‐rank test) are shown.

Over a follow‐up period of 1 year, we collected serum samples from 32 subjects with *L. reuteri* supplementation and 36 subjects with placebo. The demographic and clinical characteristics of these 68 subjects at baseline are given in Table 1 and have been previously described.^(^ ^10^ ^)^ There were no significant differences in the clinical characteristics between the *L. reuteri* and placebo groups at baseline. The design of the study is depicted in Fig. 1*A*. Overall, we collected 269 serum samples from these subjects during 1‐year follow‐up at four time points: baseline; 3, 6, and 12 months. The resulting metabolomics data set comprised a total of 1232 metabolites with 958 compounds of known identity (named metabolites) and 274 compounds of unknown structural identity (unnamed metabolites), which were measured by LC–MS. After data normalization, we obtained measurements for 993 metabolites with <25% missing values for downstream analyses. As shown in Fig. 1*B*, we calculated the Euclidean distances between *L. reuteri* and placebo groups at different time points based on metabolic profiles and performed hierarchical clustering analysis. It was clear that *L. reuteri* and placebo groups clustered together at baseline, which showed higher metabolic similarity and agreement with similar clinical parameters. In addition, metabolic profiles exhibited differences between the *L. reuteri* and placebo groups in the follow‐up period.

### The dynamic alterations of metabolomic profiles in *L. reuteri* and placebo groups during follow‐up

To investigate how the human serum metabolome changed during the 1‐year follow‐up, we first identified differences between two sampling time points in the *L. reuteri* or placebo groups, respectively. The significantly differential metabolites over time in each group are shown in Fig. 1 *C* and Supplementary Information Table S1 (adjusted *p* < 0.1 with FDR by the Wilcoxon signed‐rank test). Over the course of the intervention, 67 (7 upregulated and 60 downregulated), 33 (2 upregulated and 31 downregulated), and 41 (10 upregulated and 31 downregulated) metabolites were significantly differential at 3, 6, and 12 months, respectively, compared with baseline in the placebo group, while a few metabolites were detected differentially in the *L. reuteri* group. Less metabolic variations were observed when comparing different sampling time points after baseline in both *L. reuteri* and placebo groups. In addition, there were only three common metabolites that changed significantly over time both in the *L. reuteri* and placebo groups.

### Differential metabolic responses linked to probiotic supplementation

**Fig 2:**
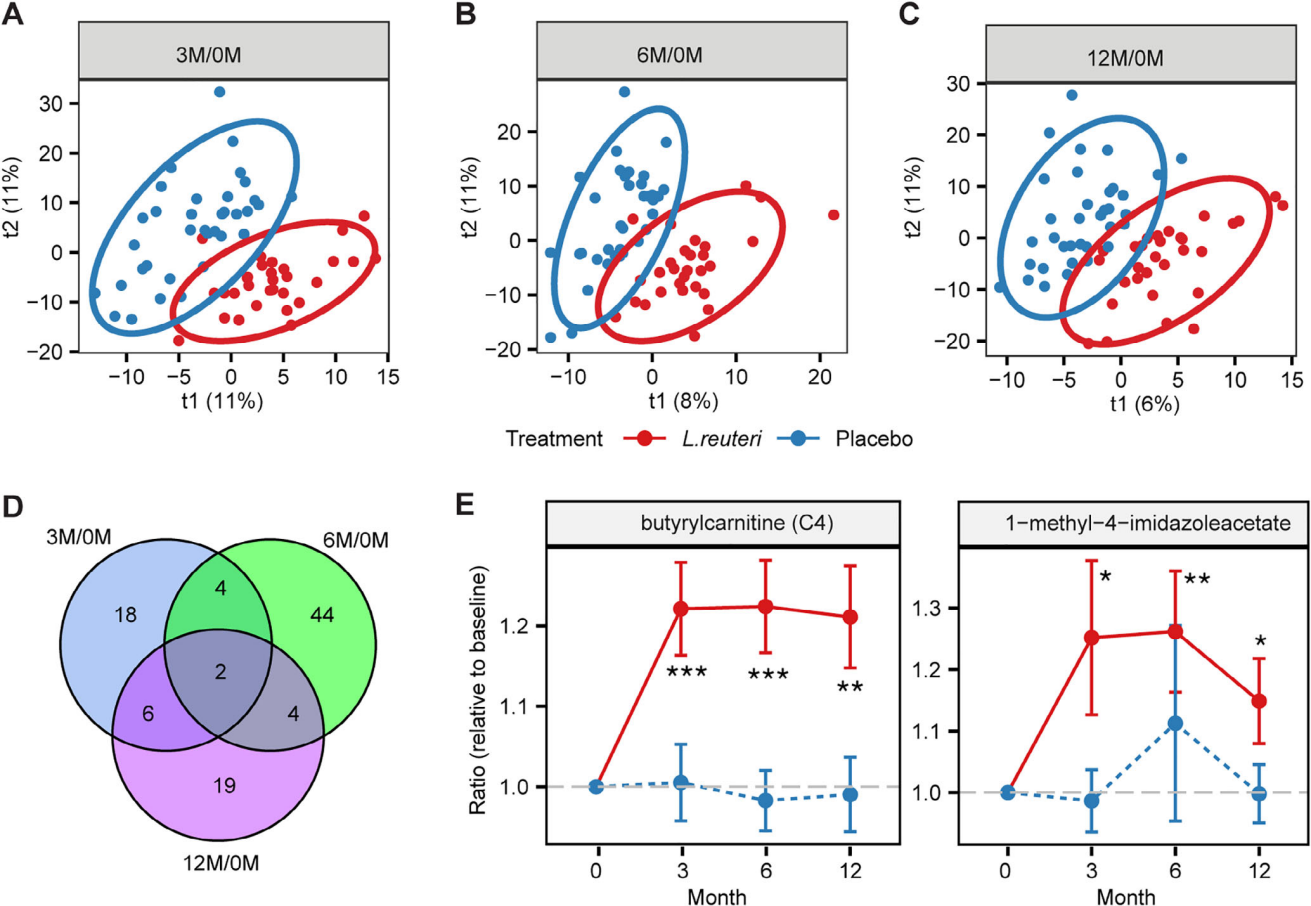
The differential responses to the treatment with *Lactobacillus reuteri*. The score plots of partial least squares‐discriminant analysis models discriminating the *L. reuteri* group from the placebo group based on metabolic responses (i.e., changes from baseline) at 3 (*A*), 6 (*B*), and 12 (*C*) months, respectively. (*D*) The metabolites differed in changing from baseline between *L. reuteri* and placebo groups at 3, 6, and 12 months (variable importance in projection score >1 and *p* value <0.05). (*E*) The relative changes from baseline (mean ± SE) of butyrylcarnitine (C4) and 1‐methyl‐4‐imidazoleacetate that responded differentially between the *L. reuteri* and placebo groups at all time points are shown. * *p* < 0.05; **p < 0.01; ***p < 0.001.

Next, we investigated whether probiotic‐specific responses occurred during 1 year, which might promote our understanding of the mechanistic effects of *L. reuteri* on human metabolism. Therefore, we first calculated the metabolite level at follow‐up time points as a ratio of the baseline value that was referred to as the metabolic response to the treatment. Then, the variances in the metabolic response were investigated by PLS‐DA. The PLS‐DA results revealed a distinct segregation of the *L. reuteri* and placebo groups in response to treatment at 3, 6, and 12 months, respectively (Fig. 2*A*‐*C*). We further identified metabolites that showed differences in relative change from baseline between *L. reuteri* and placebo groups by the Wilcoxon rank‐sum test. There were no significant metabolites identified when we corrected for multiple testing (adjusted *p* < 0.1). In total, 97 metabolites responded differentially at any one time point between *L. reuteri* and placebo groups, when using the cutoff of both VIP score cut‐off of >1 in the PLS‐DA model and *p* value <0.05 in the Wilcoxon rank‐sum test (Supplementary Information Table S2 and Supplementary Information Fig. S1). There were 30, 54, and 31 metabolites that changed from baseline differentially between *L. reuteri* and placebo groups at 3, 6, and 12 months, respectively (Fig. 2*D*). Of these, 16 metabolites were changed differentially between *L. reuteri* and placebo groups at two follow‐up time points. Interestingly, two metabolites (butyrylcarnitine (C4) and 1‐methyl‐4‐imidazoleacetate) responded differentially at three follow‐up time points (Fig. 2*E*). Most of the identified 97 metabolites are known to be involved in biological processes, including lipid, amino acid, peptide, and cofactors metabolism (Supplementary Information Fig. S2).

### Associations between metabolic levels and clinical outcomes

The associations between 16 clinical variables at baseline and 12 months were examined, respectively (Supplementary Information Fig. S3). At baseline, total tibia vBMD, cortical vBMD, and cortical thickness were associated positively with usCRP, which is a marker of inflammation (adjusted *p* < 0.1). After a 12‐month intervention, we did not observe significant associations between usCRP and bone‐related traits. Total body weight, fat mass, and BMI were positively associated with usCRP and TNF‐α at both time points (adjusted *p* < 0.05). In addition, two biochemical markers of bone turnover, BAP and NTX, were associated negatively with bone‐related traits, such as total tibia vBMD and cortical vBMD, which were consistent with previous results.^(^ ^26^, ^27^, ^28^, ^29^ ^)^

**Fig 3:**
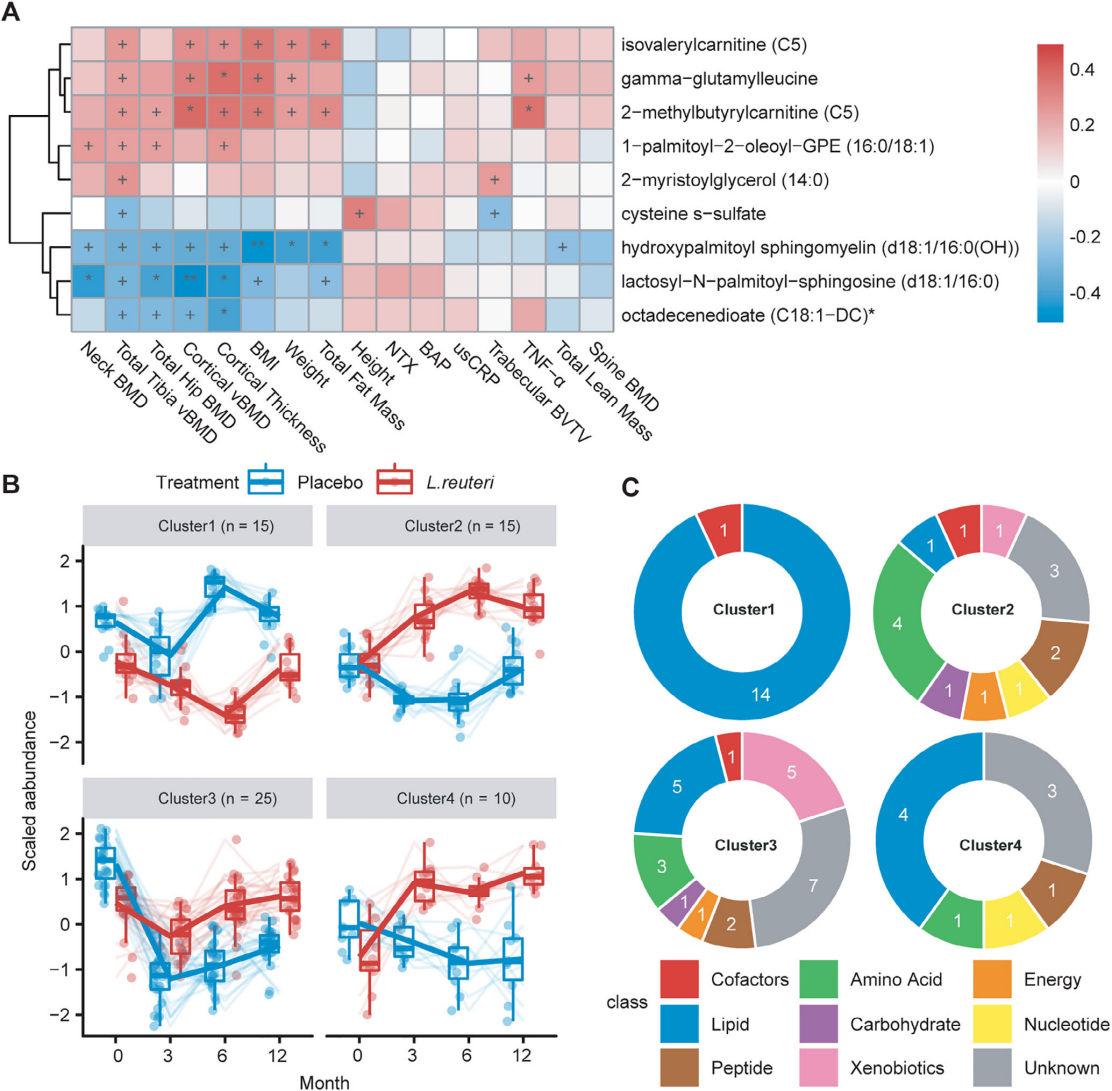
(*A*) The Pearson's correlation coefficients between 16 clinical variables and 9 BMD‐associated metabolites at 12 months are shown. +*p* < 0.05; *adjusted *p* < 0.1; **adjusted *p* < 0.05. The false discovery rate was used to correct for multiple testing. (*B*) Clustering of differential metabolites potentially mediated by *Lactobacillus reuteri* supplementation. Four clusters were identified with different response patterns over time in *L. reuteri* and placebo groups. (*C*) The class information of metabolites in each cluster (detailed information is listed in Supplementary Information Table S3). BAP = bone‐specific alkaline phosphatase; BVTV = bone volume fraction; NTX = N‐terminal telopeptide; usCRP = ultrasensitive C‐reactive protein; vBMD = volumetric bone mineral density.

To investigate associations between clinical outcomes and the metabolites that responded differently between *L. reuteri* and placebo groups, we calculated the Pearson's correlation coefficients between clinical characteristics and differential metabolites at baseline and 12 months, respectively. We found that 13 and 9 metabolites were correlated (*p* < 0.05) with total tibia vBMD at baseline and 12 months, respectively (Supplementary Information Fig. S4 and Fig. 3*A*). Out of them, 1‐methyl‐4‐imidazoleacetate, 1‐ribosyl‐imidazoleacetate*, isovalerylglycine, and proline were positively associated with total tibia vBMD at baseline. Gamma‐glutamyl leucine, 2‐methylbutyrylcarnitine (C5), and cysteine S‐sulfate were associated with total tibia vBMD at 12 months. Interestingly, isovalerylcarnitine (C5) was positively correlated with total tibia vBMD at both baseline and 12 months. In addition, one class of metabolites, including 1‐methyl‐4‐imidazoleacetate, 1‐ribosyl‐imidazoleacetate*, gamma‐glutamyl leucine, and 2‐methylbutyrylcarnitine (C5) were not only positively associated with total tibia vBMD, but also with TNF‐α and BMI at baseline or 12 months.

### Dynamic patterns of differential metabolites associated with *L. reuteri* supplementation

Clustering analyses were performed to identify different alteration trends of metabolites in response to supplementation with *L. reuteri* or placebo. The differential metabolites were clustered into mainly four different response patterns (Fig. 3*B*). The class information of metabolites in each cluster is shown in Fig. 3*C*. Cluster 1 (*n* = 15) showed reduced levels in the *L. reuteri* group in comparison with the placebo group and enrichment in the metabolism of sphingomyelins, which were decreased in the early stage and then increased at 6 months. Cluster 2 (*n* = 15) increased and peaked at 6 months in the *L. reuteri* group, whereas it was steady in the placebo group. In this cluster, similar responses were found for four metabolites involved in amino acid metabolism: 1‐methyl‐4‐imidazoleacetate, 1‐ribosyl‐imidazoleacetate*, N,N,N‐trimethyl‐alanylproline betaine (TMAP), and N6,N6‐dimethyllysine. Besides, two peptides gamma‐glutamyl‐alpha‐lysine and gamma‐glutamyl tryptophan reacted very similarly to the intervention. Metabolites of cluster 3 (*n* = 25) were reduced at 3 months and then showed increased levels in both groups, although at higher levels in the *L. reuteri* group. In addition, metabolites of cluster 4 (*n* = 10) increased over time in the *L. reuteri* group, whereas they decreased in the placebo group. In this cluster, four metabolites associated with lipid metabolism, butyrylcarnitine (C4), glycerol, heptenedioate (C7:1‐DC)*, and androsteroid monosulfate C19H28O6S (1)*, responded differentially between *L. reuteri* and placebo groups. Thus, by using clustering analysis more insights were provided into the affected biological processes having similar regulation during the 1‐year intervention.

### Metabolic pathways affected by treatment with *L. reuteri*

The important metabolites that differed in response to supplementation with *L. reuteri* and placebo at any one time point (VIP score >1 and *p* value <0.05) and the related metabolic pathways are described in detail below.

#### *Amino acid metabolism*

Among the 97 differential metabolites, 15 of them were involved in amino acid metabolism. The plasma levels of three amino acids, including proline, threonine, and valine, showed a different response between the *L. reuteri* and placebo groups (Supplementary Information Fig. S5*A*‐*C*). They all showed increased trends after supplementation with *L. reuteri* compared with placebo. In response to probiotic supplementation, isovalerylcarnitine (C5), 2‐methylbutyrylcarnitine (C5), and isovalerylglycine, involved in branched chain amino acid (BCAA) metabolism, exhibited differences between *L. reuteri* and placebo groups (Supplementary Information Fig. S5*D*‐*F*). Subjects in the *L. reuteri* group had higher levels of BCAA‐derived metabolites in comparison with the placebo group. The level of cysteine S‐sulfate related to cysteine metabolism decreased in response to treatment with *L. reuteri* compared with the placebo group (Supplementary Information Fig. S5*G*). Simultaneously, the level of cysteine S‐sulfate correlated negatively with total tibia vBMD at 12 months (Fig. 3*A*). In addition, 1‐methyl‐4‐imidazoleacetate and 1‐ribosyl‐imidazoleacetate*, involved in histidine metabolism, showed increased levels in the *L. reuteri* group compared with the placebo group (Fig. 2 *E* and Supplementary Information Fig. S5*H*). Simultaneously, they were associated positively (*r* = 0.3 and *r* = 0.34, respectively; *p* < 0.05) with total tibia vBMD at baseline (Supplementary Information Fig. S4). Conversely, the levels of indolepropionate and indole‐3‐carboxylate, involved in tryptophan metabolism, was at a steady level in the *L. reuteri* group, whereas it increased over time in the placebo group (Supplementary Information Fig. S5 *I*,*J*).

#### *Peptide metabolism*

The plasma levels of four peptide metabolites, gamma‐glutamyl‐alpha‐lysine, gamma‐glutamyl leucine, gamma‐glutamyl threonine, and gamma‐glutamyl tyrosine, showed different responses between the *L. reuteri* and placebo groups (Supplementary Information Fig. S6 *A*‐*D*). They all showed increased levels compared with the placebo group. This indicates an increased uptake of amino acids in the *L. reuteri* group in comparison with the placebo group (Supplementary Information Fig. S5 *A*‐*C*).

#### *Lipid metabolism*

Several metabolites in the fatty acid subclass that responded differentially between the *L. reuteri* and placebo groups were identified. Butyrylcarnitine (C4), caprate (10:0), 2‐hydroxydecanoate, heptenedioate (C7:1‐DC)*, and octadecenedioate (C18:1‐DC)* showed increased levels compared with the placebo group (Fig. 2 *E* and Supplementary Information Fig. S7 *A*‐*D*). Moreover, the levels of succinylcarnitine (C4) and deoxycarnitine, which are involved in carnitine metabolism, increased compared with the placebo group (Supplementary Information Fig. S7 *E*,*F*). In addition, the glycerol level was increased in the *L. reuteri* group compared with the placebo group, which indicates that the glycerol and fatty acids increased consistently (Supplementary Information Fig. S7 *G*). Conversely, linolenoylcarnitine (C18:3)*, linoleoylcarnitine (C18:2)*, and sphingomyelin (d18:1/22:2, d18:2/22:1, d16:1/24:2)* levels were reduced in the *L. reuteri* group compared with the placebo group (Supplementary Information Fig. S7 *H*‐*J*).

### Differential metabolites distinguishing the case group from the control group

To confirm the associations between BMD and the identified metabolites mediated by *L. reuteri* supplementation, we further performed metabolomics profiling in a case–control cohort including 120 older women with high BMD (control) and 120 older women with very low BMD (case). In total, 240 serum samples were collected and measured. The clinical characteristics of the subjects in the cohort are provided in Table 2. After data normalization, we obtained 1007 metabolites for downstream analyses. We investigated the variances in the metabolomics data by PCA and PLS‐DA. The PCA results did not reveal distinct metabolomic profiles between the case and control groups (Supplementary Information Fig. S8 *A*). We also examined the correlations of the first two principal components of the PCA with measured clinical parameters (Supplementary Information Fig. S8 *B*). BMI and weight were correlated with the first two principal components. In addition, the PLS‐DA analysis showed better segregation of the case and control groups (Supplementary Information Fig. S9 *A*).

**Fig 4:**
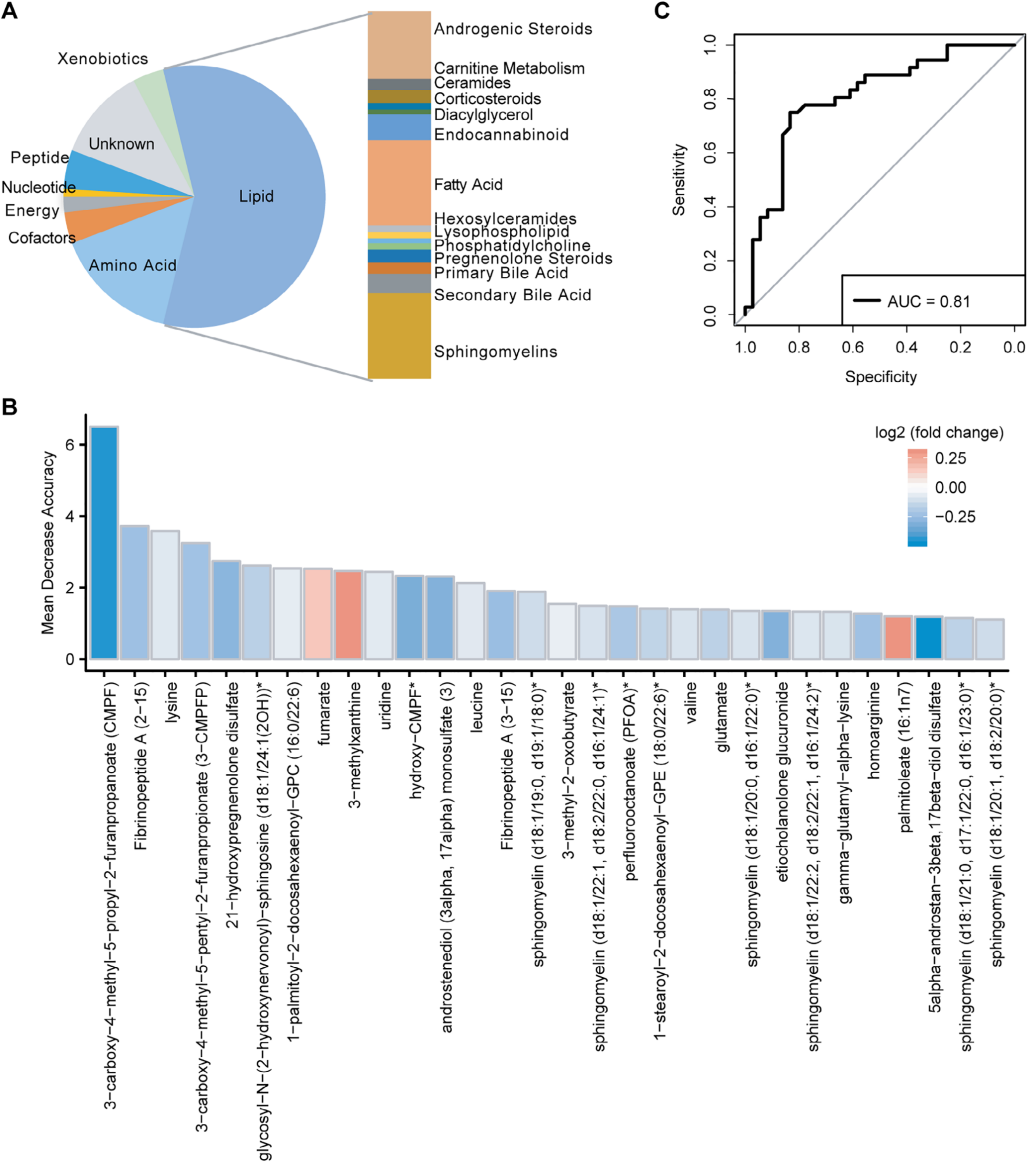
The metabolites identified in the case group versus the control group. (*A*) The class information of 104 differential metabolites is shown. (*B*) The receiver operating characteristic (ROC) curve of the random forest model using 104 metabolites. Area under the ROC curve (AUC) = 0.81 for control versus case groups (*n* = 36, respectively). (*C*) The top 30 important metabolites identified by the random forest model. The color shows log2‐fold change.

Next, the differences of metabolites between the case and control groups were compared using linear regression models adjusted by the confounders age and BMI. No individually significantly differential metabolites were observed (adjusted *p* < 0.1; Supplementary Information Table S4). Furthermore, differential metabolites between the case and control groups were selected by using the cut‐off of the VIP score of >1 in the PLS‐DA model and *p* value <0.05 in the fold changes of metabolites. A total of 104 differential metabolites were identified (Supplementary Information Fig. S9 *B*): 81 metabolites showed lower levels and 23 metabolites showed higher levels in the case group compared with the control group, which mainly classified into lipid, amino acid, peptide, and cofactors (Fig. 4*A*).

To achieve better discrimination between the case group and control group, a random forest model was developed based on a training set using the 104 differential metabolite abundance of 70% of the subjects selected randomly from the case and control groups (*n* = 84, respectively). Performance of the random forest model was evaluated by using a validation set (consisting of 30% of subjects selected randomly from the case and control groups, *n* = 36, respectively) and scored the predictive power by the ROC analysis. The discriminatory power of these metabolites was calculated as the AUC. Low BMD status could be correctly identified with an AUC of 0.81 (Fig. 4*B*). The variable importance by a mean decrease in accuracy was calculated for the random forest model. The top 30 differential metabolites ordered by importance are given in Fig. 4*C*: 3‐carboxy‐4‐methyl‐5‐propyl‐2‐furanpropanoate (CMPF) related to fatty acid metabolism was the most important metabolite for discrimination of the case and control groups. Lysine and fumarate were among the top 10 important metabolites. Moreover, leucine, valine, glutamate, gamma‐glutamyl‐alpha‐lysine, and homoarginine were important metabolites for the identification of low BMD.

### Pathway analysis of differential metabolites between the case and control groups

Differential metabolites between the case and control groups were further analyzed using the web‐based tool MetaboAnalyst that has integrated pathway enrichment analysis with the topology analysis. These differential metabolites were mapped to 38 relevant KEGG pathways (Supplementary Information Fig. S10). The significantly enriched metabolic pathways (*p* < 0.05; Table 3) were mainly involved in amino acid metabolism, steroid hormone biosynthesis, butanoate metabolism, and the citrate cycle. Thus, amino acid metabolism was not only associated with low BMD but was also affected by treatment with *L. reuteri* (Supplementary Information Fig. S5). In addition, steroid hormone biosynthesis was significantly differential between case and control groups—consistent with the fact that steroid hormones influence bone metabolism by regulating the balance of osteoclasts and osteoblasts.^(^ ^30^, ^31^, ^32^ ^)^

### Metabolites differed between the case and control groups and simultaneously were regulated by treatment with *L. reuteri*

Among 104 differential metabolites differing between the case and control groups, 12 of them also showed differential responses to *L. reuteri* supplementation (Table 4). Valine, TMAP, and isovalerylcarnitine (C5) levels were higher in the control (high BMD) group compared with the case (low BMD) group, which was consistent with previous observations that they had increased levels after supplementation with *L. reuteri* (Supplementary Information Fig. S5). Cysteine S‐sulfate levels were higher in the control group, whereas they decreased in response to supplementation with *L. reuteri* (Supplementary Information Fig. S5 *G*). Thus, these metabolites involved in amino acid metabolism were not only differential between the case and control groups, but also affected by supplementation of *L. reuteri*. In addition, gamma‐glutamyl‐alpha‐lysine and gamma‐glutamyl leucine levels were higher in the control group, consistent with the fact that they were increased by supplementation of *L. reuteri* (Supplementary Information Fig. S6 *A*, *B*). Moreover, deoxycarnitine levels were higher in the control group and simultaneously rose in response to supplementation with *L. reuteri* (Supplementary Information Fig. S7 *F*).

## Discussion

Supplementation of *L. reuteri* was recently reported to substantially reduce bone loss in older women.^(^ ^10^ ^)^ However, the effects of *L. reuteri* on human metabolism are still not clear. Here, we first analyzed time‐series metabolomic profiles of elderly women with bone loss to evaluate if metabolic changes were caused by the supplementation of *L. reuteri*. The study population showed no significant differences in the clinical characteristics between *L. reuteri* and placebo groups at baseline, indicating a successful randomization. The positive correlation between cortical vBMD and us‐CRP was only observed at baseline, inconsistent with previous findings that the CRP level was inversely associated with BMD.^(^ ^33^ ^)^ In addition, BMI was positively associated with us‐CRP and TNF‐α at both time points. This supports the previous finding that obesity is associated with increased inflammation.^(^ ^34^, ^35^ ^)^ The bone turnover markers BAP and NTx were associated negatively with bone‐related traits, such as total tibia vBMD and cortical vBMD, which was consistent with previous results.^(^ ^26^, ^27^, ^28^, ^29^ ^)^

Serum samples were collected from these subjects in the placebo‐controlled RCT cohort during 1‐year follow‐up at baseline and 3, 6, and 12 months. Similar metabolic profiles were observed at baseline between the *L. reuteri* and placebo groups. Less variations were observed over time in the *L. reuteri* group by pairwise comparisons of different time points. Overall, the metabolic profiles exhibited differences between the *L. reuteri* and placebo groups at the follow‐up time points. We further investigated whether metabolic changes were triggered by the probiotic treatment, which might help understand the mechanistic effects of *L. reuteri* on human metabolism. Using the cut‐off of a VIP score of >1 in the PLS‐DA model and *p* value <0.05 in the Wilcoxon rank‐sum test, 97 metabolites showed differential responses between the *L. reuteri* and placebo groups, and were involved in multiple metabolic processes including amino acid, peptide, and lipid metabolism. Interestingly, these differential metabolites were clustered into mainly four different response patterns, thus providing more insights into the biological processes in which metabolites were consistently regulated by the supplementation of *L. reuteri*.

**Fig 5:**
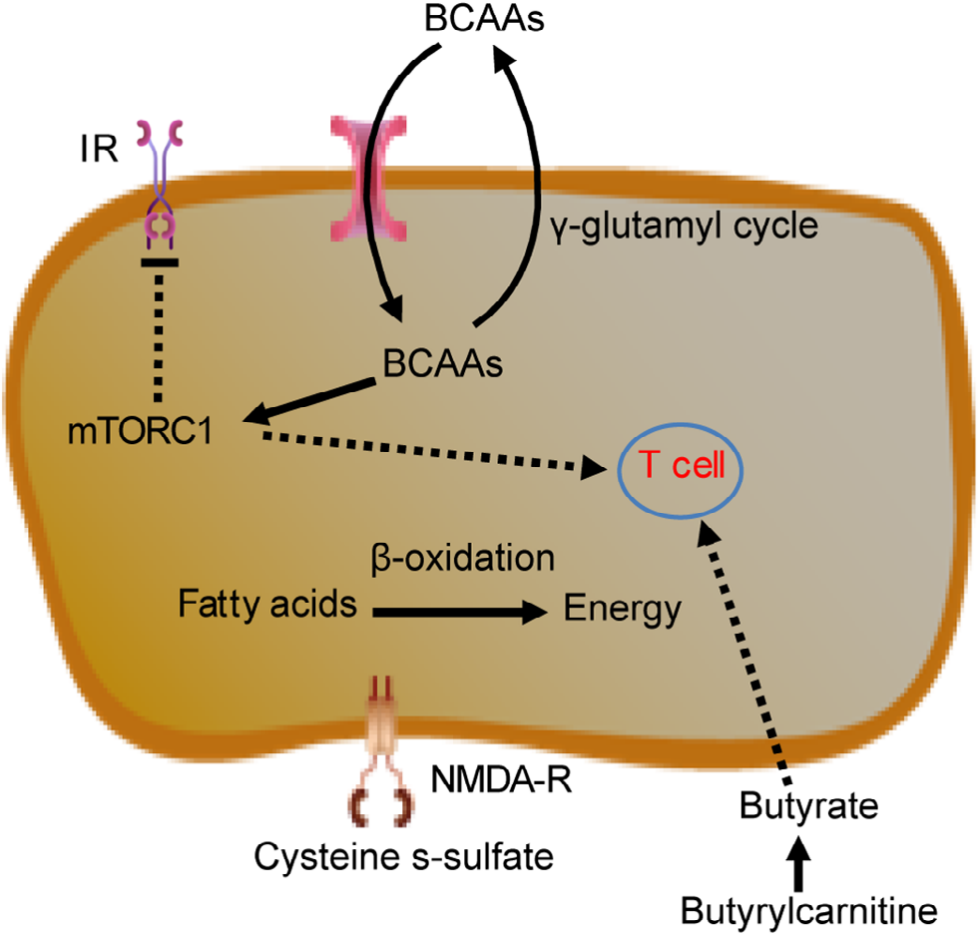
Illustration of the hypothetical mechanism of the effects of probiotic *Lactobacillus reuteri* on bone metabolism. BCAAs could be transported by the γ‐glutamyl cycle and activate complex 1 of mammalian target of rapamycin (mTORC1). mTORC1 stimulates osteoblast differentiation and improves bone health directly. In addition, mTORC1 plays a critical role in T‐cell regulation and insulin signaling, which affect bone homeostasis indirectly. Cysteine S‐sulfate may be an important regulator of bone metabolism by interacting with NMDA‐R that is involved in bone resorption. The energy produced during the β oxidation of the identified fatty acids may promote bone formation. Butyrylcarnitine may act as the pool and transporter of butyrate and have potential for stimulating bone formation. γ‐glutamyl‐AAs = gamma‐glutamyl‐amino acids; BCAAs = branched chain amino acids; IR = insulin receptor; mTORC1 = complex 1 of mammalian target of rapamycin; NMDA‐R = N‐methyl‐D‐aspartate receptor.

Amino acids, including proline, threonine, and valine, showed differential responses between the *L. reuteri* and placebo groups. It had previously been reported that amino acids as signaling molecules modulate bone turnover and are associated with higher BMD.^(^ ^36^, ^37^ ^)^ Therefore, our data support that supplementation of the probiotic *L. reuteri* will increase the serum amino acid levels that may regulate bone metabolism in older women. The branched‐chain amino acids (valine) and their derivatives, isovalerylcarnitine (C5), 2‐methylbutyrylcarnitine (C5), and isovalerylglycine, especially increased with the supplementation of *L. reuteri*. Interestingly, isovalerylcarnitine (C5) showed a positive correlation with total tibia vBMD at both baseline and 12 months, which suggests it may be a potential biomarker of bone loss. The results were consistent with the rising levels of gamma‐glutamyl‐amino acids, which may transport amino acids by the γ‐glutamyl cycle.^(^ ^38^ ^)^ As seen in Fig. 5, BCAAs could be transported into the cytoplasm by the γ‐glutamyl cycle. Then, intracellular BCAAs may stimulate osteoblast differentiation and improve bone metabolism by activating complex 1 of the mammalian target of rapamycin (mTORC1) directly.^(^ ^39^, ^40^ ^)^ It is possible that BCAAs could also improve bone density by indirectly regulating mTORC1, which plays critical roles in T‐cell regulation and insulin signaling that affects bone homeostasis.^(^ ^41^, ^42^, ^43^ ^)^

The level of cysteine S‐sulfate decreased in response to treatment with *L. reuteri* compared with the placebo group. Simultaneously, the metabolite was correlated negatively with total tibia vBMD at 12 months. Interestingly, cysteine S‐sulfate is a structural analog of glutamate that acts as an N‐methyl‐D‐aspartate receptor (NMDA‐R) agonist.^(^ ^44^ ^)^ A previous study has reported expression and function of NMDA‐R in osteoclasts involved in bone resorption.^(^ ^45^ ^)^ Thus, cysteine S‐sulfate may be a potential regulator interacting with NMDA‐R that is required for bone metabolism,^(^ ^46^ ^)^ as illustrated in Fig. 5. In addition, 1‐methyl‐4‐imidazoleacetate and 1‐ribosyl‐imidazoleacetate* that are involved in histidine metabolism were not only regulated by *L. reuteri* treatment, but also associated positively with total tibia vBMD at baseline. Simultaneously, they were associated positively with TNF‐α and BMI at baseline. These results suggest that these two metabolites may be regulated by TNF‐α, which is known to affect bone metabolism.^(^ ^47^ ^)^ The levels of indolepropionate and indole‐3‐carboxylate involved in tryptophan metabolism were kept steady in the *L. reuteri* group, whereas they increased over time in the placebo group. This is consistent with previous reports on tryptophan metabolites having an influence on bone remodeling.^(^ ^48^ ^)^

Previous studies have shown that fatty acid oxidation plays an important role in the energy metabolism of bone.^(^ ^49^, ^50^ ^)^ In this study, butyrylcarnitine (C4) and caprate (10:0), related to fatty acid metabolism, also showed increased levels in the *L. reuteri* group compared with the placebo group. Thus, the energy produced during the β oxidation of the identified fatty acids may promote bone formation. Notably, butyrylcarnitine (C4), a butyrate ester of carnitine, may act as the pool and transporter of butyrate,^(^ ^51^ ^)^ which has previously been shown to inhibit bone resorption and stimulate bone formation in mice through a mechanism involving regulatory T cells and Wnt10b.^(^ ^6^, ^7^ ^)^ Butyrate supplementation was recently reported to increase bone mass in WT mice and to prevent ovariectomy‐induced bone loss.^(^ ^52^ ^)^ Thus, the robust increase in this metabolite during the whole treatment period may explain the effect of *L. reuteri* in reducing bone loss as recently shown by our research group.^(^ ^10^ ^)^ Based on data from an experimental study in mice, Yan and colleagues proposed that short‐chain fatty acids may restore IGF‐1 levels depleted by antibiotic treatment. IGF‐1 is known to promote skeletal growth and has effects on both bone formation and resorption.^(^ ^53^ ^)^ In a recent cohort study, associations between different metabolites and plasma IGF‐1 levels were investigated in women.^(^ ^54^ ^)^ Serum levels of the amino acid threonine, which was altered by *L. reuteri* treatment in the present study, was found to be associated with serum IGF‐1 levels, indicating that the IGF‐1 signaling pathway and pathways affected by *L. reuteri* treatment could overlap, at least some extent.

It has previously been shown that carnitine participates in the transportation of fatty acids into the mitochondrion where fatty acid oxidation takes place and improves BMD by suppressing bone turnover.^(^ ^55^ ^)^ Moreover, sphingomyelin (d18:1/22:2, d18:2/22:1, d16:1/24:2)* level decreased in the *L. reuteri* group compared with the placebo group, which agreed with previous results that sphingomyelin affects bone mineralization.^(^ ^56^, ^57^ ^)^

To validate the associations between low BMD and the identified metabolites regulated by *L. reuteri* supplementation, we analyzed the metabolomic profiles of a case and control population of elderly women with severe osteoporosis or high BMD. A total of 104 differential metabolites were identified, which were mainly involved in lipid, amino acid, peptide, and cofactors. Using a machine‐learning approach, low BMD status could be correctly predicted with an AUC of 0.81 by a random forest model using the validation set, showing a robust association between these metabolites and BMD. Metabolites CMPF, lysine, fumarate, leucine, valine, glutamate, gamma‐glutamyl‐alpha‐lysine, and homoarginine were the top metabolites discriminating the case and control groups. Homoarginine has been reported to be associated with bone density previously.^(^ ^58^ ^)^

In addition, we found significant differences in BCAAs biosynthesis and steroid hormone biosynthesis between the case (low BMD) and control (high BMD) groups. It is well known that steroid hormones, including cortisol, DHEA sulfate, and other sex steroids, influence bone metabolism by regulating the balance between osteoclasts and osteoblasts.^(^ ^30^, ^31^, ^32^ ^)^ In total, 12 metabolites differed between the case and control groups and were simultaneously regulated by treatment with *L. reuteri*; valine, cysteine S‐sulfate, and isovalerylcarnitine (C5) were identified. Therefore, these metabolites may be not only potential biomarkers of low BMD but also possible links between bone metabolism and probiotic treatment.

It must be acknowledged that the metabolomics analysis was not a predefined analysis determined before unblinding in the RCT and that the number of women included in the RCT was small, which could have influenced our results. Moreover, our ability to identify differentially regulated metabolites could have been affected by multiple comparisons. Although no identified single metabolite remained significantly differentially regulated by *L. reuteri* supplementation when applying a Bonferroni test or FDR correction, the PLS‐DA analysis showed a specific set of metabolites able to segregate the *L. reuteri* from the placebo group. The single differentially regulated metabolites identified in this study, are hypothesis generating but these findings require confirmation in future studies. Furthermore, causal relationships cannot be determined by the identified associations. The results were obtained in older women and may not be representative of men and other age groups.

In conclusion, metabolomic profiling was employed to explore the effect of *L. reuteri* on the metabolic dynamics in older women with bone loss. Global shifting of metabolic status was observed during the 1‐year trial. Our previous RCT found that supplementation with probiotic *L. reuteri* reduces bone loss, and our present analysis indicates that the treatment is associated with changes in amino acid, peptide, and lipid metabolism in elderly women. Butyrylcarnitine (C4) especially exhibited a robust increase in subjects with *L. reuteri* treatment, indicating the involvement of butyrate signaling in bone metabolism. Further studies are, however, needed to identify the mechanisms and determine how gut microbiota changes are caused by supplementation with probiotic *L. reuteri* and how such changes are linked to human metabolomic dynamics.

## Author Contributions

**Li Peishun:** Data curation; formal analysis; investigation; methodology; software; validation; visualization; writing‐original draft. **Daniel Sundh:** Data curation; investigation; project administration; resources; validation; writing‐review & editing. **Ji Boyang:** Data curation; formal analysis; investigation; methodology; software; validation; visualization; writing‐review & editing. **Dimitri Lappa:** Data curation; formal analysis; investigation; methodology; software; validation; visualization; writing‐review & editing. **Ye Lingqun:** Data curation; formal analysis; investigation; methodology; software; supervision; validation; writing‐review & editing. **Mattias Lorentzon:** Conceptualization; data curation; funding acquisition; investigation; methodology; project administration; resources; software; supervision; validation; visualization; writing‐original draft; writing‐review & editing.

## Authors' roles

ML and JN conceived and designed the study. DS and ML collected samples. PL, BJ, and DL performed data analyses. LY helped with statistical analysis. PL, BJ, ML, and JN wrote and revised the manuscript. All authors critically reviewed and approved the manuscript.

## Conflict of Interest

The authors declare no competing interests. Prof Lorentzon has received lecture or consulting fees from Amgen, Astellas, Lilly, Renapharma, UCB Pharma, Radius Health, Meda, GE‐Lunar, and Santax Medico/Hologic, all outside the herein presented work. BioGaia AB provided funding for the randomized controlled trial,^(^ ^10^ ^)^ which the current report is partially based on. BioGaia AB did not have access to any metabolomics data and has not participated in the analysis, interpretation, or presentation of results of the present study. Jens Nielsen is a minority shareholder in Metabogen AB.

### Peer Review

The peer review history for this article is available at https://publons.com/publon/10.1002/jbm4.10478.

## Supporting information


**Fig. S1** Quantile‐quantile (QQ) plots of − log_10_[P] of comparisons of changes from baseline (i.e. ratios) between the *L. reuteri* and placebo groups at 3 (A), 6 (B) and 12 (C) months, respectively.
**Fig. S2**. Pie chart displaying pathway distribution of metabolites responding differently to the intervention.
**Fig. S3**. Heatmap of the Pearson's correlation coefficients between clinical characteristics and serum parameters at baseline (A) and 12 months (B). ‘+’ denotes *p* < 0.05; ‘*’ denotes adjusted *p* < 0.1; ‘**’ denotes adjusted *p* < 0.05. The FDR was used to correct for multiple testing. BMI, body mass index; Neck BMD, femoral neck BMD; Spine BMD, lumbar spine BMD; Total Tibia vBMD, total tibia volumetric BMD; Cortical vBMD, cortical volumetric BMD; Trabecular BVTV, trabecular bone volume fraction; BAP, bone‐specific alkaline phosphatase; NTX, N‐terminal telopeptide; usCRP, ultrasensitive C‐reactive protein; TNF‐α, tumor necrosis factor alpha.
**Fig. S4**. Heatmap of the Pearson's correlation coefficients between 16 clinical variables and 13 BMD‐associated metabolites at baseline. ‘+’ denotes *p* < 0.05; ‘*’ denotes adjusted *p* < 0.1; ‘**’ denotes adjusted *p* < 0.05. The FDR was used to correct for multiple testing.
**Fig. S5**. The metabolites involving in amino acid metabolism that responded differentially between *L. reuteri* and placebo groups. The relative changes from baseline (Mean ± SEM) of amino acids (A‐C), branched chain amino acids derivatives (D‐F), cysteine derivatives (G), histidine derivatives (H) and tryptophan derivatives (I, J) were shown. ‘*’, *p* < 0.05; ‘**’, *p* < 0.01; ‘***’, *p* < 0.001.
**Fig. S6**. The metabolites involving in peptide metabolism that responded differentially between *L. reuteri* and placebo groups. The relative change from baseline (Mean ± SE) of peptides (A‐D) were shown. ‘*’, *p* < 0.05; ‘**’, *p* < 0.01.
**Fig. S7**. The metabolites involving in lipid metabolism that responded differentially between *L. reuteri* and placebo groups. The relative change from baseline (Mean ± SE) of fatty acids (A‐D), carnitine (E, F), glycerol (G), acyl carnitine (H, I) and sphingomyelin (J) were shown. ‘*’, *p* < 0.05; ‘**’, *p* < 0.01.
**Fig. S8**. PCA based on the metabolic profiles from case and control groups. (A) The score plot of PCA. (B) The correlations of the first two principal components of PCA with measured clinical variables by Spearman's rank correlation analysis. Adjusted *p* < 0.1 indicates significant correlations. Weight, total body weight; BMI, body mass index; FN BMD, femoral neck BMD; Spine BMD, lumbar spine BMD; THipBMD, total hip BMD; Tibia vBMD, total tibia volumetric BMD; FRAX MOF, the fracture risk assessment tool FRAX score for the 10‐year probability of a major osteoporotic fracture; PCS, physical component score derived from SF‐12 (measure of physical activity).
**Fig. S9**. Metabolites identified to distinguish the case group from the control group. (A) The score plot of PLS‐DA model based on the metabolic profiles. (B) The volcano plot displaying differential metabolites between two groups identified by *p* value in linear regression models and Metabolomics profiling reveals Rehmanniae radix preparata extract protects against glucocorticoid‐induced osteoporosis mainly via intervening steroid hormone biosynthesis (VIP) in PLS‐DA model. *p* < 0.05; The red dots represent the significantly up‐regulated expressed metabolites; the green dots represent the downregulated expressed metabolites.
**Fig. S10**. KEGG pathways to which the differential metabolites were mapped using web‐based tool Metaboanalyst for pathway analysis. Pathway impact value is calculated from pathway topology analysis; *p* value is calculated from the enrichment analysis.
**Fig. S11**. Preparation of client‐specific technical replicates. A small aliquot of each client sample (colored cylinders) is pooled to create a CMTRX technical replicate sample (multi‐colored cylinder), which is then injected periodically throughout the platform run. Variability among consistently detected biochemicals can be used to calculate an estimate of overall process and platform variability.
**Fig. S12**. Visualization of data normalization steps for a multiday platform run.

**Table S1**(a): The differential metabolites over time in the L. reuteri group, compared by the Wilcoxon signed‐rank test.

**Table S1**(b): The differential metabolites over time in the placebo group, compared by the Wilcoxon signed‐rank test.

**Table S2**: Metabolites responding differentially at any one time point between L. reuteri and placebo groups, identified by using the cut‐off of both VIP score of >1 in the PLS‐DA model and p value >0.05 in the Wilcoxon rank‐sum test.

**Table S3**: The four response patterns identified by clustering analysis of the metabolites that responded differently between the L. reuteri and placebo groups.

**Table S4**: The differential metabolites between the case and control groups, comparing by linear regression models adjusted by the confounders age and BMI.
