# Supplementary material for: Metabolic Alterations in Older Women With Low Bone Mineral Density Supplemented With Lactobacillus reuteri
Source: JBMR Plus. 2021 Mar 15;5(4):e10478. doi: 10.1002/jbm4.10478 (PMC8046097; doi:10.1002/jbm4.10478)
Supplement: Supplementary file 1 — Fig. S1 Quantile‐quantile (QQ) plots of − log10[P] of comparisons of changes from baseline (i.e. ratios) between the L. reuteri and placebo groups at 3 (A), 6 (B) and 12 (C) months, respectively. Fig. S2. Pie chart displaying pathway distribution of metabolites responding differently to the intervention. Fig. S3. Heatmap of the Pearson's correlation coefficients between clinical characteristics and serum parameters at baseline (A) and 12 months (B). ‘+’ denotes p < 0.05; ‘*’ denotes adjusted p < 0.1; ‘**’ denotes adjusted p < 0.05. The FDR was used to correct for multiple testing. BMI, body mass index; Neck BMD, femoral neck BMD; Spine BMD, lumbar spine BMD; Total Tibia vBMD, total tibia volumetric BMD; Cortical vBMD, cortical volumetric BMD; Trabecular BVTV, trabecular bone volume fraction; BAP, bone‐specific alkaline phosphatase; NTX, N‐terminal telopeptide; usCRP, ultrasensitive C‐reactive protein; TNF‐α, tumor necrosis factor alpha. Fig. S4. Heatmap of the Pearson's correlation coefficients between 16 clinical variables and 13 BMD‐associated metabolites at baseline. ‘+’ denotes p < 0.05; ‘*’ denotes adjusted p < 0.1; ‘**’ denotes adjusted p < 0.05. The FDR was used to correct for multiple testing. Fig. S5. The metabolites involving in amino acid metabolism that responded differentially between L. reuteri and placebo groups. The relative changes from baseline (Mean ± SEM) of amino acids (A‐C), branched chain amino acids derivatives (D‐F), cysteine derivatives (G), histidine derivatives (H) and tryptophan derivatives (I, J) were shown. ‘*’, p < 0.05; ‘**’, p < 0.01; ‘***’, p < 0.001. Fig. S6. The metabolites involving in peptide metabolism that responded differentially between L. reuteri and placebo groups. The relative change from baseline (Mean ± SE) of peptides (A‐D) were shown. ‘*’, p < 0.05; ‘**’, p < 0.01. Fig. S7. The metabolites involving in lipid metabolism that responded differentially between L. reuteri and placebo groups. The relative change from basel [file JBM4-5-e10478-s005.docx]

Supplemental Materials and Methods

Supplemental Figures


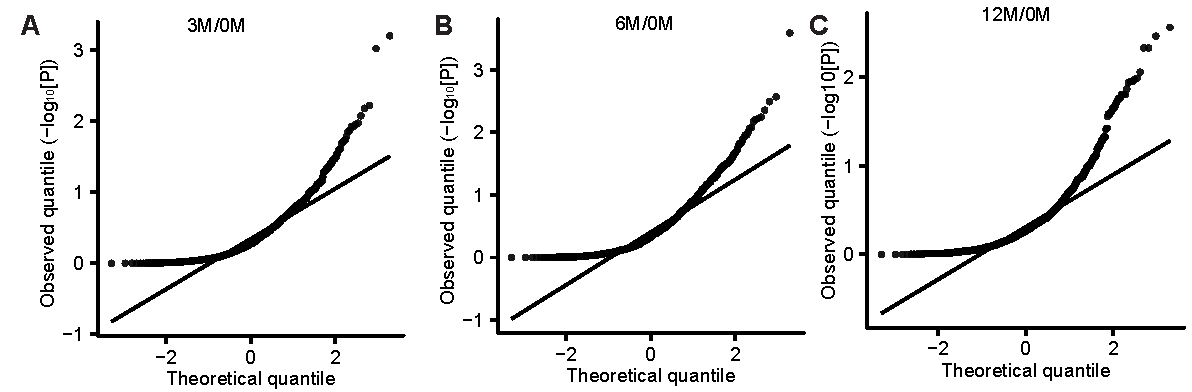


**Figure S1. Quantile-quantile (QQ) plots of −log_10_[P] of comparisons of changes from baseline (i.e. ratios) between the *L. reuteri* and placebo groups at 3 (A), 6 (B) and 12 (C) months, respectively.**


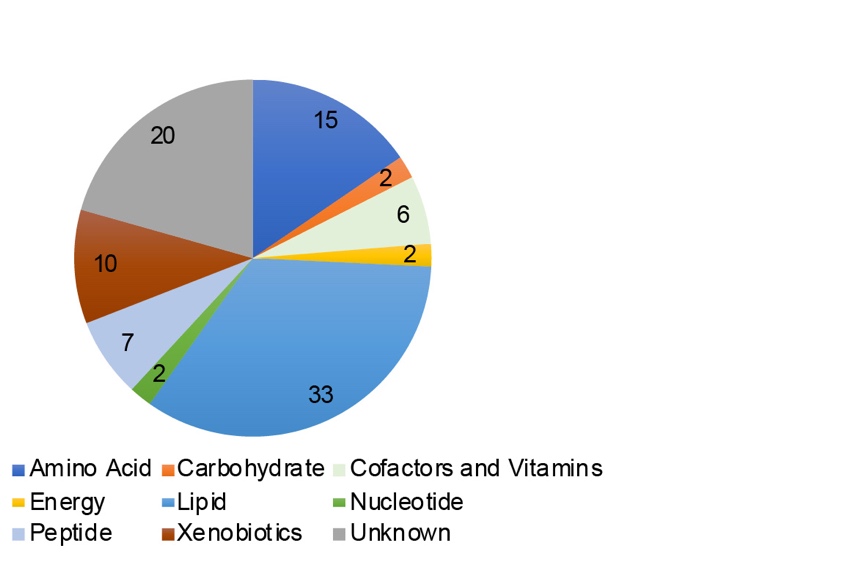


**Figure S2. Pie chart displaying pathway distribution of metabolites responding differently to the intervention.**

**Figure S3. Heatmap of the Pearson’s correlation coefficients between clinical characteristics and serum parameters at baseline (A) and 12 months (B).** ‘+’ denotes *P* < 0.05; ‘*’ denotes adjusted *P* < 0.1; ‘**’ denotes adjusted *P* < 0.05. The FDR was used to correct for multiple testing. BMI, body mass index; Neck BMD, femoral neck BMD; Spine BMD, lumbar spine BMD; Total Tibia vBMD, total tibia volumetric BMD; Cortical vBMD, cortical volumetric BMD; Trabecular BVTV, trabecular bone volume fraction; BAP, bone-specific alkaline phosphatase; NTX, N-terminal telopeptide; usCRP, ultrasensitive C-reactive protein; TNF-α, tumor necrosis factor alpha.

**Figure S4. Heatmap of the Pearson’s correlation coefficients between 16 clinical variables and 13 BMD-associated metabolites at baseline.** ‘+’ denotes *P* < 0.05; ‘*’ denotes adjusted *P* < 0.1; ‘**’ denotes adjusted *P* < 0.05. The FDR was used to correct for multiple testing.


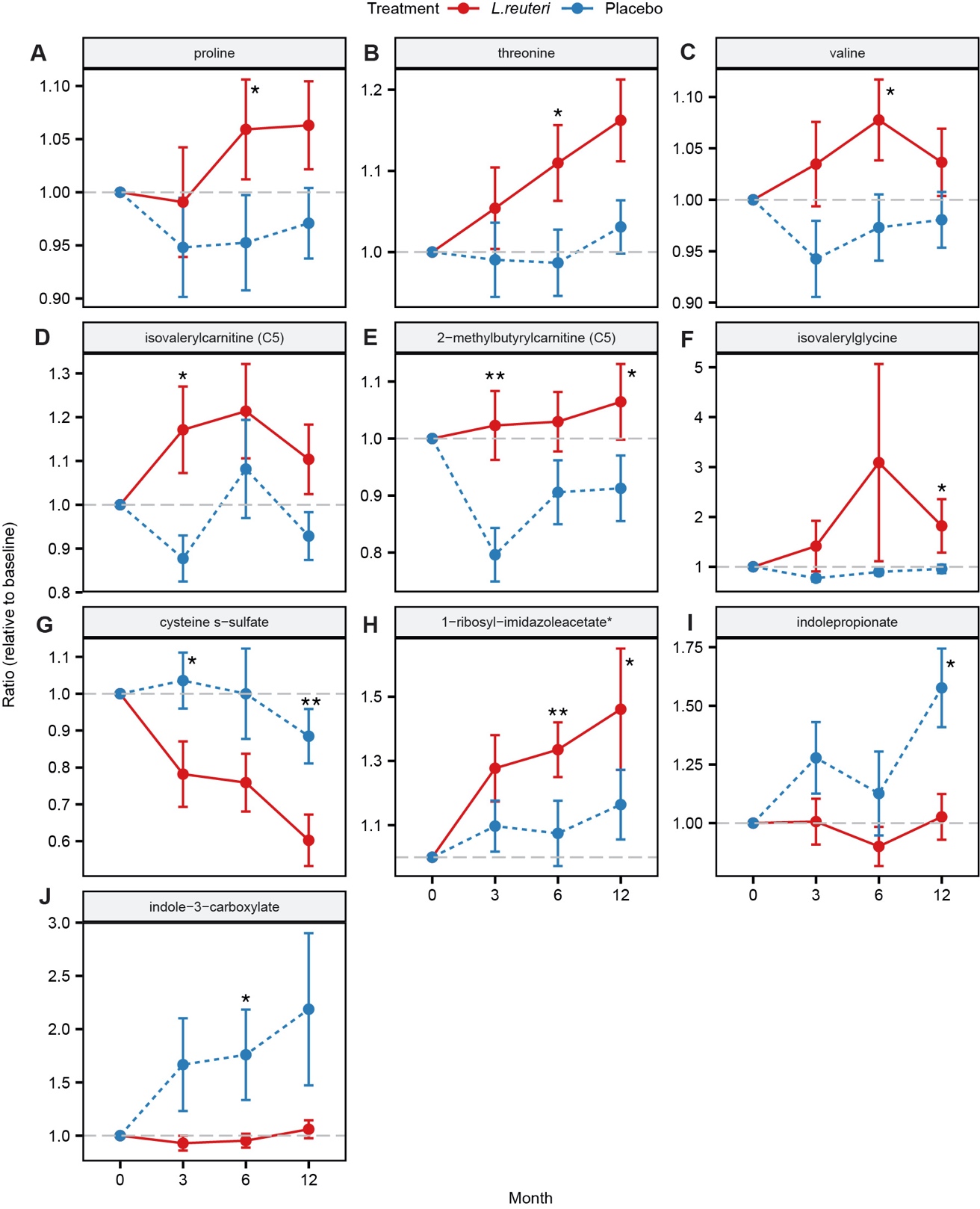


**Figure S5. The metabolites involving in amino acid metabolism that responded differentially between *L. reuteri* and placebo groups.** The relative changes from baseline (Mean ± SEM) of amino acids (A-C), branched chain amino acids derivatives (D-F), cysteine derivatives (G), histidine derivatives (H) and tryptophan derivatives (I, J) were shown. ‘*’, *P* < 0.05; ‘**’, *P* < 0.01; ‘***’, *P* < 0.001.

^
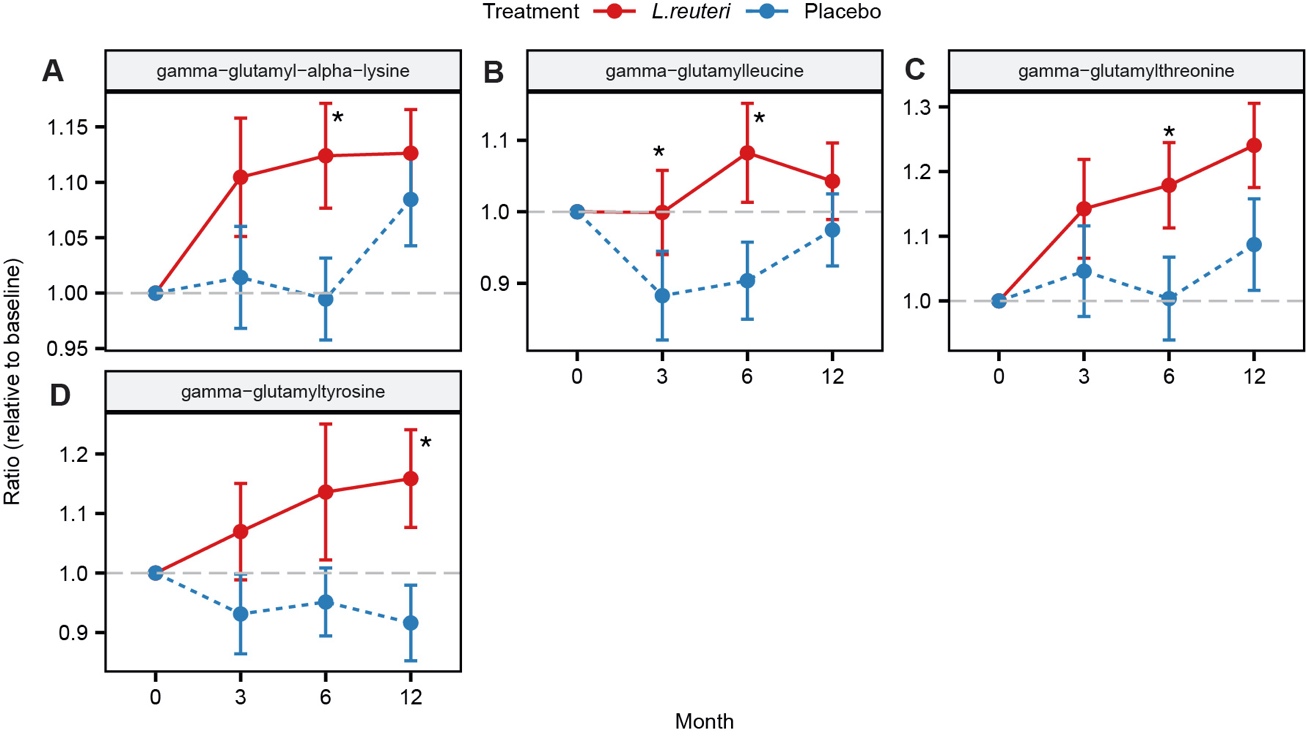
^

**Figure S6. The metabolites involving in peptide metabolism that responded differentially between *L. reuteri* and placebo groups.** The relative change from baseline (Mean ± SE) of peptides (A-D) were shown. ‘*’, *P* < 0.05; ‘**’, *P* < 0.01.


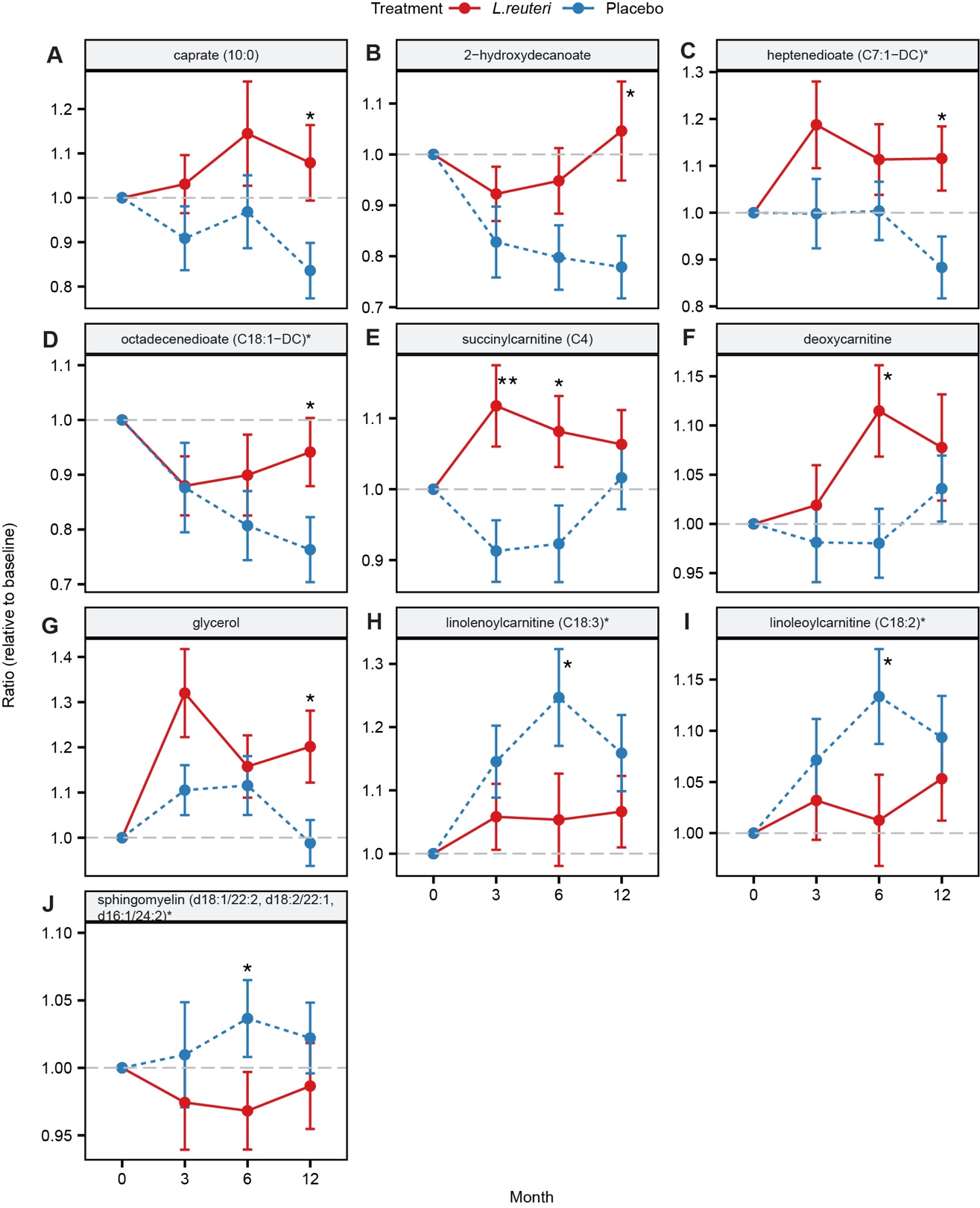


**Figure S7. The metabolites involving in lipid metabolism that responded differentially between *L. reuteri* and placebo groups.** The relative change from baseline (Mean ± SE) of fatty acids (A-D), carnitine (E, F), glycerol (G), acyl carnitine (H, I) and sphingomyelin (J) were shown. ‘*’, *P* < 0.05; ‘**’, *P* < 0.01.

**Figure S8. PCA based on the metabolic profiles from case and control groups.** (A) The score plot of PCA. (B) The correlations of the first two principal components of PCA with measured clinical variables by Spearman's rank correlation analysis. Adjusted *P* < 0.1 indicates significant correlations. Weight, total body weight; BMI, body mass index; FN BMD, femoral neck BMD; Spine BMD, lumbar spine BMD; THipBMD, total hip BMD; Tibia vBMD, total tibia volumetric BMD; FRAX MOF, the fracture risk assessment tool FRAX score for the 10-year probability of a major osteoporotic fracture; PCS, physical component score derived from SF-12 (measure of physical activity).

**Figure S9. Metabolites identified to distinguish the case group from the control group.** (A) The score plot of PLS-DA model based on the metabolic profiles. (B) The volcano plot displaying differential metabolites between two groups identified by *P* value in linear regression models and Variable Importance in Projection (VIP) in PLS-DA model. *P* < 0.05; The red dots represent the significantly up-regulated expressed metabolites; the green dots represent the downregulated expressed metabolites.

**Figure S10. KEGG pathways to which the differential metabolites were mapped using web-based tool Metaboanalyst for pathway analysis.** Pathway impact value is calculated from pathway topology analysis; *P* value is calculated from the enrichment analysis.

Supplemental Methods

**Study Parameters**

**Table S5. Data Quality: Instrument and Process Variability.**

| QC Sample | Measurement | Median RSD |
| --- | --- | --- |
| Internal Standards | Instrument Variability | 7% |
| Endogenous Biochemicals | Total Process Variability | 10% |

Instrument variability was determined by calculating the median relative standard deviation (RSD) for the internal standards that were added to each sample prior to injection into the mass spectrometers. Overall process variability was determined by calculating the median RSD for all endogenous metabolites (i.e., non-instrument standards) present in 100% of the Client Matrix samples, which are technical replicates of pooled client samples. Values for instrument and process variability meet Metabolon’s acceptance criteria as shown in **Supplemental Table 5**.

**Metabolon Platform**

**Sample Accessioning**: Following receipt, samples were inventoried and immediately stored at -80°C. Each sample received was accessioned into the Metabolon LIMS system and was assigned by the LIMS a unique identifier that was associated with the original source identifier only. This identifier was used to track all sample handling, tasks, results, etc. The samples (and all derived aliquots) were tracked by the LIMS system. All portions of any sample were automatically assigned their own unique identifiers by the LIMS when a new task was created; the relationship of these samples was also tracked. All samples were maintained at -80°C until processed.

**Sample Preparation**: Samples were prepared using the automated MicroLab STAR^®^ system from Hamilton Company. Several recovery standards were added prior to the first step in the extraction process for QC purposes. To remove protein, dissociate small molecules bound to protein or trapped in the precipitated protein matrix, and to recover chemically diverse metabolites, proteins were precipitated with methanol under vigorous shaking for 2 min (Glen Mills GenoGrinder 2000) followed by centrifugation. The resulting extract was divided into five fractions: two for analysis by two separate reverse phase (RP)/UPLC-MS/MS methods with positive ion mode electrospray ionization (ESI), one for analysis by RP/UPLC-MS/MS with negative ion mode ESI, one for analysis by HILIC/UPLC-MS/MS with negative ion mode ESI, and one sample was reserved for backup. Samples were placed briefly on a TurboVap^®^ (Zymark) to remove the organic solvent. The sample extracts were stored overnight under nitrogen before preparation for analysis.

**QA/QC**: Several types of controls were analyzed in concert with the experimental samples: a pooled matrix sample generated by taking a small volume of each experimental sample (or alternatively, use of a pool of well-characterized human plasma) served as a technical replicate throughout the data set; extracted water samples served as process blanks; and a cocktail of QC standards that were carefully chosen not to interfere with the measurement of endogenous compounds were spiked into every analyzed sample, allowed instrument performance monitoring and aided chromatographic alignment. **Supplemental Table 6 and 7** describe these QC samples and standards. Instrument variability was determined by calculating the median relative standard deviation (RSD) for the standards that were added to each sample prior to injection into the mass spectrometers. Overall process variability was determined by calculating the median RSD for all endogenous metabolites (i.e., non-instrument standards) present in 100% of the pooled matrix samples. Experimental samples were randomized across the platform run with QC samples spaced evenly among the injections, as outlined in **Supplemental Figure 11**.

**Table S6: Description of Metabolon QC Samples**

| Type | Description | Purpose |
| --- | --- | --- |
| MTRX | Large pool of human plasma maintained by Metabolon that has been characterized extensively. | Assure that all aspects of the Metabolon process are operating within specifications. |
| CMTRX | Pool created by taking a small aliquot from every customer sample. | Assess the effect of a non-plasma matrix on the Metabolon process and distinguish biological variability from process variability. |
| PRCS | Aliquot of ultra-pure water | Process Blank used to assess the contribution to compound signals from the process. |
| SOLV | Aliquot of solvents used in extraction. | Solvent Blank used to segregate contamination sources in the extraction. |

**Table S7: Metabolon QC Standards**

| Type | Description | Purpose |
| --- | --- | --- |
| RS | Recovery Standard | Assess variability and verify performance of extraction and instrumentation. |
| IS | Internal Standard | Assess variability and performance of instrument. |

**Figure S11. Preparation of client-specific technical replicates.** A small aliquot of each client sample (colored cylinders) is pooled to create a CMTRX technical replicate sample (multi-colored cylinder), which is then injected periodically throughout the platform run. Variability among consistently detected biochemicals can be used to calculate an estimate of overall process and platform variability.

**Ultrahigh Performance Liquid Chromatography-Tandem Mass Spectroscopy (UPLC-MS/MS):** All methods utilized a Waters ACQUITY ultra-performance liquid chromatography (UPLC) and a Thermo Scientific Q-Exactive high resolution/accurate mass spectrometer interfaced with a heated electrospray ionization (HESI-II) source and Orbitrap mass analyzer operated at 35,000 mass resolution. The sample extract was dried then reconstituted in solvents compatible to each of the four methods. Each reconstitution solvent contained a series of standards at fixed concentrations to ensure injection and chromatographic consistency. One aliquot was analyzed using acidic positive ion conditions, chromatographically optimized for more hydrophilic compounds. In this method, the extract was gradient eluted from a C18 column (Waters UPLC BEH C18-2.1x100 mm, 1.7 µm) using water and methanol, containing 0.05% perfluoropentanoic acid (PFPA) and 0.1% formic acid (FA). Another aliquot was also analyzed using acidic positive ion conditions, however it was chromatographically optimized for more hydrophobic compounds. In this method, the extract was gradient eluted from the same afore mentioned C18 column using methanol, acetonitrile, water, 0.05% PFPA and 0.01% FA and was operated at an overall higher organic content. Another aliquot was analyzed using basic negative ion optimized conditions using a separate dedicated C18 column. The basic extracts were gradient eluted from the column using methanol and water, however with 6.5mM Ammonium Bicarbonate at pH 8. The fourth aliquot was analyzed via negative ionization following elution from a HILIC column (Waters UPLC BEH Amide 2.1x150 mm, 1.7 µm) using a gradient consisting of water and acetonitrile with 10mM Ammonium Formate, pH 10.8. The MS analysis alternated between MS and data-dependent MS^n^ scans using dynamic exclusion. The scan range varied slighted between methods but covered 70-1000 m/z. Raw data files are archived and extracted as described below.

**Bioinformatics:** The informatics system consisted of four major components, the Laboratory Information Management System (LIMS), the data extraction and peak-identification software, data processing tools for QC and compound identification, and a collection of information interpretation and visualization tools for use by data analysts. The hardware and software foundations for these informatics components were the LAN backbone, and a database server running Oracle 10.2.0.1 Enterprise Edition.

**LIMS:** The purpose of the Metabolon LIMS system was to enable fully auditable laboratory automation through a secure, easy to use, and highly specialized system. The scope of the Metabolon LIMS system encompasses sample accessioning, sample preparation and instrumental analysis and reporting and advanced data analysis. All of the subsequent software systems are grounded in the LIMS data structures. It has been modified to leverage and interface with the in-house information extraction and data visualization systems, as well as third party instrumentation and data analysis software.

**Data Extraction and Compound Identification:** Raw data was extracted, peak-identified and QC processed using Metabolon’s hardware and software. These systems are built on a web-service platform utilizing Microsoft’s .NET technologies, which run on high-performance application servers and fiber-channel storage arrays in clusters to provide active failover and load-balancing. Compounds were identified by comparison to library entries of purified standards or recurrent unknown entities. Metabolon maintains a library based on authenticated standards that contains the retention time/index (RI), mass to charge ratio (m/z), and chromatographic data (including MS/MS spectral data) on all molecules present in the library. Furthermore, biochemical identifications are based on three criteria: retention index within a narrow RI window of the proposed identification, accurate mass match to the library +/- 10 ppm, and the MS/MS forward and reverse scores between the experimental data and authentic standards. The MS/MS scores are based on a comparison of the ions present in the experimental spectrum to the ions present in the library spectrum. While there may be similarities between these molecules based on one of these factors, the use of all three data points can be utilized to distinguish and differentiate biochemicals. More than 3300 commercially available purified standard compounds have been acquired and registered into LIMS for analysis on all platforms for determination of their analytical characteristics. Additional mass spectral entries have been created for structurally unnamed biochemicals, which have been identified by virtue of their recurrent nature (both chromatographic and mass spectral). These compounds have the potential to be identified by future acquisition of a matching purified standard or by classical structural analysis.

**Curation:** A variety of curation procedures were carried out to ensure that a high quality data set was made available for statistical analysis and data interpretation. The QC and curation processes were designed to ensure accurate and consistent identification of true chemical entities, and to remove those representing system artifacts, mis-assignments, and background noise. Metabolon data analysts use proprietary visualization and interpretation software to confirm the consistency of peak identification among the various samples. Library matches for each compound were checked for each sample and corrected if necessary.

**Metabolite Quantification and Data Normalization:** Peaks were quantified using area under the curve. For studies spanning multiple days, a data normalization step was performed to correct variation resulting from instrument inter-day tuning differences. Essentially, each compound was corrected in run-day blocks by registering the medians to equal one (1.00) and normalizing each data point proportionately (termed the “block correction”; **Supplemental Figure 12**). For studies that did not require more than one day of analysis, no normalization is necessary, other than for purposes of data visualization. In certain instances, biochemical data may have been normalized to an additional factor (e.g., cell counts, total protein as determined by Bradford assay, osmolality, etc.) to account for differences in metabolite levels due to differences in the amount of material present in each sample.

**Figure S12.** Visualization of data normalization steps for a multiday platform run.
