# Supplementary material for: Metabolic Alterations in Older Women With Low Bone Mineral Density Supplemented With Lactobacillus reuteri
Source: JBMR Plus. 2021 Mar 15;5(4):e10478. doi: 10.1002/jbm4.10478 (PMC8046097; doi:10.1002/jbm4.10478)
Supplement: Supplementary file 2 — Table S1(a): The differential metabolites over time in the L. reuteri group, compared by the Wilcoxon signed‐rank test. [file JBM4-5-e10478-s003.pdf]

Supplemental Table 1

			Placebo group																							
			3M vs 0M				6M vs 0M				12M vs 0M				6M vs 3M				12M vs 3M				12M vs 6M			
Biochemical Name	Super Pathwa	Sub Pathway	Fold Chang	P	Adj.P FDR	Adj. P Bonf.	Fold Chang	P	Adj.P FDR	Adj. P Bonf.	Fold Chang	P	Adj.P FDR	Adj. P Bonf.	Fold Chang	P	Adj.P FDR	Adj. P Bonf.	Fold Chang	P	Adj.P FDR	Adj. P Bonf.	Fold Chang	P	Adj.P FDR	Adj. P Bonf.
(16 or 17)-methylstearate (a19:0 or i19:0)	Lipid	Fatty Acid, Branched	1.08	0.57	0.82	1.00	1.06	0.37	0.74	1.00	1.03	0.79	0.96	1.00	0.98	0.92	0.98	1.00	0.95	0.31	0.66	1.00	0.97	0.36	0.78	1.00
(N(1) + N(8))-acetylspermidine	Amino Acid	Polyamine Metabolism	1.06	0.19	0.54	1.00	1.01	0.94	1.00	1.00	1.17	0.00	0.06	1.00	0.95	0.24	0.76	1.00	1.11	0.02	0.40	1.00	1.16	0.00	0.05	0.51
1-(1-enyl-palmitoyl)-2-arachidonoyl-GPC (P-16:0/	Lipid	Plasmalogen	0.99	0.98	0.99	1.00	1.01	0.94	1.00	1.00	1.03	0.55	0.93	1.00	1.02	0.41	0.83	1.00	1.04	0.19	0.58	1.00	1.02	0.60	0.86	1.00
1-(1-enyl-palmitoyl)-2-arachidonoyl-GPE (P-16:0/	Lipid	Plasmalogen	0.98	0.83	0.95	1.00	0.97	0.47	0.80	1.00	1.03	0.31	0.81	1.00	0.99	0.87	0.97	1.00	1.05	0.14	0.55	1.00	1.07	0.25	0.71	1.00
1-(1-enyl-palmitoyl)-2-linoleoyl-GPC (P-16:0/18:	Lipid	Plasmalogen	0.99	0.60	0.84	1.00	1.01	0.73	0.90	1.00	1.02	0.75	0.95	1.00	1.02	0.20	0.72	1.00	1.03	0.41	0.72	1.00	1.01	0.68	0.89	1.00
1-(1-enyl-palmitoyl)-2-linoleoyl-GPE (P-16:0/18:	Lipid	Plasmalogen	0.94	0.64	0.86	1.00	0.98	0.98	1.00	1.00	1.02	0.74	0.95	1.00	1.04	0.38	0.82	1.00	1.09	0.07	0.49	1.00	1.05	0.38	0.78	1.00
1-(1-enyl-palmitoyl)-2-oleoyl-GPC (P-16:0/18:1)*	Lipid	Plasmalogen	0.98	0.53	0.81	1.00	0.99	0.89	0.98	1.00	0.98	0.59	0.94	1.00	1.01	0.64	0.92	1.00	1.00	0.85	0.95	1.00	0.99	0.75	0.92	1.00
1-(1-enyl-palmitoyl)-2-oleoyl-GPE (P-16:0/18:1)*	Lipid	Plasmalogen	0.92	0.26	0.60	1.00	0.97	0.70	0.90	1.00	0.97	0.37	0.84	1.00	1.05	0.28	0.79	1.00	1.05	0.28	0.63	1.00	1.01	0.78	0.94	1.00
1-(1-enyl-palmitoyl)-2-palmitoleoyl-GPC (P-16:0/	Lipid	Plasmalogen	1.02	0.45	0.76	1.00	1.00	0.83	0.95	1.00	1.01	0.61	0.94	1.00	0.98	0.21	0.72	1.00	0.99	0.88	0.96	1.00	1.01	0.60	0.86	1.00
1-(1-enyl-palmitoyl)-2-palmitoyl-GPC (P-16:0/16	Lipid	Plasmalogen	1.01	0.75	0.92	1.00	1.00	0.92	0.98	1.00	1.01	0.57	0.94	1.00	0.98	0.49	0.88	1.00	1.00	0.98	0.99	1.00	1.01	0.63	0.87	1.00
1-(1-enyl-palmitoyl)-GPC (P-16:0)*	Lipid	Lysoplasmalogen	0.97	0.48	0.77	1.00	1.00	0.99	1.00	1.00	1.03	0.62	0.94	1.00	1.03	0.10	0.62	1.00	1.06	0.08	0.49	1.00	1.03	0.54	0.84	1.00
1-(1-enyl-palmitoyl)-GPE (P-16:0)*	Lipid	Lysoplasmalogen	1.01	1.00	1.00	1.00	0.97	0.92	0.98	1.00	1.04	0.34	0.83	1.00	0.96	0.83	0.97	1.00	1.03	0.11	0.53	1.00	1.07	0.16	0.66	1.00
1-(1-enyl-stearoyl)-2-arachidonoyl-GPE (P-18:0/2	Lipid	Plasmalogen	0.95	0.42	0.73	1.00	0.99	0.80	0.94	1.00	1.00	1.00	1.00	1.00	1.04	0.27	0.79	1.00	1.05	0.27	0.63	1.00	1.01	0.95	0.99	1.00
1-(1-enyl-stearoyl)-2-linoleoyl-GPE (P-18:0/18:2)	Lipid	Plasmalogen	0.91	0.20	0.55	1.00	0.99	0.87	0.97	1.00	0.99	0.67	0.94	1.00	1.08	0.08	0.62	1.00	1.08	0.11	0.53	1.00	1.00	0.99	1.00	1.00
1-(1-enyl-stearoyl)-2-oleoyl-GPE (P-18:0/18:1)	Lipid	Plasmalogen	0.94	0.35	0.67	1.00	0.97	0.46	0.80	1.00	1.02	0.95	1.00	1.00	1.03	0.61	0.91	1.00	1.08	0.10	0.52	1.00	1.05	0.39	0.78	1.00
1-(1-enyl-stearoyl)-GPE (P-18:0)*	Lipid	Lysoplasmalogen	0.99	0.53	0.81	1.00	0.96	0.79	0.94	1.00	1.02	0.70	0.94	1.00	0.97	0.70	0.93	1.00	1.03	0.08	0.49	1.00	1.07	0.40	0.78	1.00
1,2-dilinoleoyl-GPC (18:2/18:2)	Lipid	Phosphatidylcholine (PC)	0.95	0.32	0.65	1.00	1.02	0.69	0.89	1.00	1.03	0.97	1.00	1.00	1.07	0.08	0.62	1.00	1.08	0.11	0.53	1.00	1.01	0.76	0.93	1.00
1,2-dipalmitoyl-GPC (16:0/16:0)	Lipid	Phosphatidylcholine (PC)	0.96	0.44	0.75	1.00	0.96	0.20	0.60	1.00	0.96	0.10	0.51	1.00	1.00	0.78	0.95	1.00	1.00	0.97	0.99	1.00	1.00	0.89	0.97	1.00
1,3-dimethylurate	Xenobiotics	Xanthine Metabolism	0.91	0.27	0.61	1.00	0.95	0.50	0.81	1.00	1.07	0.85	0.98	1.00	1.05	0.89	0.97	1.00	1.18	0.23	0.63	1.00	1.12	0.19	0.68	1.00
1,5-anhydroglucitol (1,5-AG)	Carbohydrate	Glycolysis, Gluconeogenesis, and Py	0.98	0.57	0.82	1.00	0.99	0.76	0.92	1.00	1.01	0.61	0.94	1.00	1.01	0.97	0.99	1.00	1.03	0.44	0.74	1.00	1.02	0.37	0.78	1.00
1,7-dimethylurate	Xenobiotics	Xanthine Metabolism	0.88	0.18	0.53	1.00	0.89	0.10	0.46	1.00	1.03	0.70	0.94	1.00	1.00	0.95	0.99	1.00	1.17	0.14	0.55	1.00	1.16	0.09	0.64	1.00
10-heptadecenoate (17:1n7)	Lipid	Long Chain Fatty Acid	1.29	0.16	0.52	1.00	1.19	0.23	0.61	1.00	1.06	0.55	0.93	1.00	0.93	0.51	0.88	1.00	0.82	0.05	0.45	1.00	0.88	0.17	0.66	1.00
10-nonadecenoate (19:1n9)	Lipid	Long Chain Fatty Acid	1.31	0.17	0.52	1.00	1.20	0.11	0.46	1.00	1.06	0.48	0.92	1.00	0.91	0.41	0.83	1.00	0.81	0.10	0.53	1.00	0.89	0.20	0.68	1.00
10-undecenoate (11:1n1)	Lipid	Medium Chain Fatty Acid	1.09	0.33	0.66	1.00	1.04	0.69	0.89	1.00	0.97	0.33	0.82	1.00	0.96	0.41	0.83	1.00	0.89	0.05	0.45	1.00	0.93	0.21	0.68	1.00
13-HODE + 9-HODE	Lipid	Fatty Acid, Monohydroxy	0.75	0.03	0.21	1.00	0.84	0.47	0.80	1.00	0.69	0.00	0.05	1.00	1.12	0.08	0.62	1.00	0.92	0.16	0.56	1.00	0.83	0.03	0.48	1.00
16a-hydroxy DHEA 3-sulfate	Lipid	Androgenic Steroids	0.91	0.18	0.53	1.00	0.85	0.14	0.52	1.00	0.96	0.44	0.90	1.00	0.94	0.13	0.66	1.00	1.05	0.40	0.72	1.00	1.12	0.11	0.64	1.00
16-hydroxypalmitate	Lipid	Fatty Acid, Monohydroxy	1.05	0.39	0.71	1.00	1.12	0.16	0.56	1.00	1.01	0.80	0.96	1.00	1.06	0.28	0.79	1.00	0.96	0.72	0.88	1.00	0.90	0.11	0.64	1.00
1-arachidonoyl-GPC* (20:4)*	Lipid	Lysophospholipid	0.98	0.62	0.85	1.00	1.01	0.95	1.00	1.00	1.02	0.87	0.98	1.00	1.03	0.21	0.72	1.00	1.04	0.11	0.53	1.00	1.01	1.00	1.00	1.00
1-arachidonoyl-GPE (20:4n6)*	Lipid	Lysophospholipid	0.88	0.01	0.09	1.00	0.91	0.01	0.20	1.00	0.99	0.94	1.00	1.00	1.04	0.11	0.63	1.00	1.13	0.00	0.11	0.68	1.08	0.01	0.44	1.00
1-arachidonoyl-GPI* (20:4)*	Lipid	Lysophospholipid	0.90	0.12	0.47	1.00	0.99	0.74	0.91	1.00	0.97	0.64	0.94	1.00	1.09	0.13	0.66	1.00	1.07	0.40	0.72	1.00	0.98	0.66	0.88	1.00
1-arachidonylglycerol (20:4)	Lipid	Monoacylglycerol	0.93	0.34	0.67	1.00	1.00	0.94	1.00	1.00	0.88	0.15	0.62	1.00	1.08	0.19	0.72	1.00	0.94	0.66	0.85	1.00	0.88	0.24	0.70	1.00
1-carboxyethylphenylalanine	Amino Acid	Phenylalanine Metabolism	0.61	0.01	0.14	1.00	0.68	0.03	0.27	1.00	0.61	0.01	0.13	1.00	1.11	0.20	0.72	1.00	1.00	0.35	0.69	1.00	0.90	0.72	0.91	1.00
1-dihomo-linolenylglycerol (20:3)	Lipid	Monoacylglycerol	0.90	0.20	0.55	1.00	1.03	0.85	0.96	1.00	0.94	0.53	0.93	1.00	1.14	0.11	0.63	1.00	1.04	0.12	0.54	1.00	0.92	0.36	0.78	1.00
1-docosahexaenoylglycerol (22:6)	Lipid	Monoacylglycerol	1.03	0.81	0.94	1.00	1.09	0.42	0.78	1.00	0.94	0.22	0.72	1.00	1.06	0.36	0.81	1.00	0.91	0.29	0.65	1.00	0.86	0.02	0.44	1.00
1-linolenoyl-GPC (18:3)*	Lipid	Lysophospholipid	0.92	0.38	0.70	1.00	1.08	0.39	0.76	1.00	1.06	0.44	0.90	1.00	1.17	0.01	0.22	1.00	1.15	0.02	0.39	1.00	0.98	0.93	0.98	1.00
1-linoleoyl-2-linolenoyl-GPC (18:2/18:3)*	Lipid	Phosphatidylcholine (PC)	0.91	0.35	0.67	1.00	1.13	0.09	0.45	1.00	1.09	0.60	0.94	1.00	1.24	0.00	0.13	1.00	1.19	0.13	0.55	1.00	0.96	0.54	0.84	1.00
1-linoleoylglycerol (18:2)	Lipid	Monoacylglycerol	0.86	0.16	0.52	1.00	0.85	0.21	0.61	1.00	0.82	0.10	0.50	1.00	0.99	0.67	0.92	1.00	0.96	0.88	0.96	1.00	0.96	0.73	0.91	1.00
1-linoleoyl-GPC (18:2)	Lipid	Lysophospholipid	0.94	0.20	0.55	1.00	0.97	0.39	0.76	1.00	1.00	0.93	1.00	1.00	1.04	0.30	0.81	1.00	1.06	0.05	0.45	1.00	1.02	0.50	0.82	1.00
1-linoleoyl-GPE (18:2)*	Lipid	Lysophospholipid	0.72	0.00	0.02	0.28	0.83	0.01	0.17	1.00	0.88	0.06	0.40	1.00	1.16	0.03	0.44	1.00	1.23	0.00	0.10	0.38	1.06	0.28	0.72	1.00
1-linoleoyl-GPI* (18:2)*	Lipid	Lysophospholipid	0.89	0.15	0.50	1.00	0.99	0.87	0.97	1.00	1.00	0.64	0.94	1.00	1.11	0.07	0.59	1.00	1.12	0.13	0.55	1.00	1.01	0.92	0.98	1.00
1-methyl-4-imidazoleacetate	Amino Acid	Histidine Metabolism	0.95	0.49	0.77	1.00	1.19	0.10	0.46	1.00	0.98	0.34	0.83	1.00	1.25	0.73	0.94	1.00	1.02	0.24	0.63	1.00	0.82	0.27	0.72	1.00
1-methylhistidine	Amino Acid	Histidine Metabolism	0.93	0.15	0.50	1.00	0.98	0.83	0.95	1.00	1.03	0.64	0.94	1.00	1.06	0.25	0.76	1.00	1.12	0.03	0.43	1.00	1.05	0.60	0.86	1.00
1-methylnicotinamide	Cofactors and	Nicotinate and Nicotinamide Metab	0.77	0.21	0.55	1.00	0.82	0.58	0.86	1.00	0.89	0.70	0.94	1.00	1.06	0.32	0.81	1.00	1.16	0.08	0.49	1.00	1.09	0.40	0.78	1.00
1-myristoyl-2-arachidonoyl-GPC (14:0/20:4)*	Lipid	Phosphatidylcholine (PC)	0.86	0.12	0.47	1.00	1.03	0.73	0.90	1.00	0.98	0.99	1.00	1.00	1.20	0.00	0.04	0.16	1.14	0.06	0.46	1.00	0.95	0.83	0.95	1.00
1-myristoyl-2-palmitoyl-GPC (14:0/16:0)	Lipid	Phosphatidylcholine (PC)	0.86	0.03	0.24	1.00	0.98	0.63	0.87	1.00	1.00	1.00	1.00	1.00	1.14	0.03	0.44	1.00	1.17	0.03	0.43	1.00	1.02	0.53	0.83	1.00
1-myristoylglycerol (14:0)	Lipid	Monoacylglycerol	0.72	0.08	0.41	1.00	0.80	0.42	0.78	1.00	0.79	0.30	0.81	1.00	1.10	0.16	0.66	1.00	1.09	0.11	0.53	1.00	0.99	0.63	0.87	1.00
1-oleoyl-2-docosahexaenoyl-GPC (18:1/22:6)*	Lipid	Phosphatidylcholine (PC)	0.98	0.66	0.87	1.00	0.99	1.00	1.00	1.00	1.01	0.99	1.00	1.00	1.01	0.75	0.95	1.00	1.03	0.42	0.73	1.00	1.02	0.62	0.87	1.00
1-oleoyl-2-linoleoyl-GPE (18:1/18:2)*	Lipid	Phosphatidylethanolamine (PE)	0.60	0.00	0.01	0.10	0.73	0.01	0.16	1.00	0.75	0.00	0.07	1.00	1.23	0.02	0.37	1.00	1.25	0.00	0.14	1.00	1.02	0.43	0.78	1.00

1-oleoylglycerol (18:1)	Lipid	Monoacylglycerol	0.83	0.42	0.73	1.00	0.82	0.37	0.74	1.00	0.84	0.64	0.94	1.00	0.99	0.79	0.96	1.00	1.01	0.39	0.71	1.00	1.02	0.25	0.71	1.00
1-oleoyl-GPC (18:1)	Lipid	Lysophospholipid	0.96	0.50	0.78	1.00	1.00	0.92	0.98	1.00	1.03	0.40	0.88	1.00	1.04	0.24	0.76	1.00	1.07	0.06	0.46	1.00	1.03	0.14	0.65	1.00
1-oleoyl-GPE (18:1)	Lipid	Lysophospholipid	0.70	0.00	0.02	0.55	0.81	0.04	0.32	1.00	0.88	0.13	0.58	1.00	1.16	0.05	0.53	1.00	1.25	0.00	0.07	0.19	1.08	0.14	0.64	1.00
1-oleoyl-GPI (18:1)*	Lipid	Lysophospholipid	0.82	0.12	0.47	1.00	1.03	0.98	1.00	1.00	0.99	0.99	1.00	1.00	1.26	0.04	0.52	1.00	1.21	0.03	0.44	1.00	0.96	0.94	0.99	1.00
1-palmitoleoylglycerol (16:1)*	Lipid	Monoacylglycerol	0.76	0.39	0.71	1.00	0.79	0.43	0.78	1.00	0.76	0.21	0.71	1.00	1.04	0.72	0.94	1.00	0.99	0.55	0.80	1.00	0.95	0.81	0.94	1.00
1-palmitoleoyl-GPC* (16:1)*	Lipid	Lysophospholipid	0.97	0.76	0.92	1.00	1.04	0.49	0.81	1.00	1.05	0.15	0.61	1.00	1.07	0.03	0.46	1.00	1.08	0.21	0.60	1.00	1.01	0.83	0.95	1.00
1-palmitoyl-2-arachidonoyl-GPC (16:0/20:4n6)	Lipid	Phosphatidylcholine (PC)	0.94	0.18	0.53	1.00	0.96	0.58	0.86	1.00	0.98	0.69	0.94	1.00	1.03	0.27	0.78	1.00	1.04	0.11	0.53	1.00	1.02	0.68	0.89	1.00
1-palmitoyl-2-arachidonoyl-GPE (16:0/20:4)*	Lipid	Phosphatidylethanolamine (PE)	0.85	0.00	0.05	1.00	0.90	0.11	0.46	1.00	0.96	0.54	0.93	1.00	1.06	0.07	0.61	1.00	1.13	0.01	0.26	1.00	1.06	0.13	0.64	1.00
1-palmitoyl-2-arachidonoyl-GPI (16:0/20:4)*	Lipid	Phosphatidylinositol (PI)	0.91	0.44	0.75	1.00	0.99	0.83	0.95	1.00	0.92	0.85	0.98	1.00	1.09	0.14	0.66	1.00	1.01	0.43	0.73	1.00	0.93	0.48	0.82	1.00
1-palmitoyl-2-dihomo-linolenoyl-GPC (16:0/20:	Lipid	Phosphatidylcholine (PC)	0.97	0.68	0.89	1.00	1.01	0.38	0.75	1.00	1.02	0.40	0.88	1.00	1.03	0.19	0.72	1.00	1.05	0.14	0.55	1.00	1.01	0.59	0.86	1.00
1-palmitoyl-2-docosahexaenoyl-GPC (16:0/22:6)	Lipid	Phosphatidylcholine (PC)	1.01	0.57	0.82	1.00	0.99	0.90	0.98	1.00	0.99	0.83	0.98	1.00	0.98	0.35	0.81	1.00	0.99	0.49	0.77	1.00	1.01	0.80	0.94	1.00
1-palmitoyl-2-docosahexaenoyl-GPE (16:0/22:6)	Lipid	Phosphatidylethanolamine (PE)	0.95	0.29	0.63	1.00	0.97	0.68	0.89	1.00	1.01	0.97	1.00	1.00	1.02	0.70	0.93	1.00	1.06	0.24	0.63	1.00	1.04	0.27	0.72	1.00
1-palmitoyl-2-linoleoyl-GPC (16:0/18:2)	Lipid	Phosphatidylcholine (PC)	0.97	0.53	0.81	1.00	1.00	0.46	0.80	1.00	1.01	0.52	0.93	1.00	1.03	0.19	0.72	1.00	1.04	0.11	0.53	1.00	1.01	0.73	0.91	1.00
1-palmitoyl-2-linoleoyl-GPE (16:0/18:2)	Lipid	Phosphatidylethanolamine (PE)	0.72	0.00	0.02	0.38	0.82	0.02	0.27	1.00	0.84	0.02	0.27	1.00	1.13	0.02	0.37	1.00	1.16	0.01	0.30	1.00	1.03	0.52	0.83	1.00
1-palmitoyl-2-linoleoyl-GPI (16:0/18:2)	Lipid	Phosphatidylinositol (PI)	0.88	0.20	0.55	1.00	1.03	0.70	0.90	1.00	0.95	0.98	1.00	1.00	1.17	0.01	0.23	1.00	1.07	0.02	0.38	1.00	0.92	0.41	0.78	1.00
1-palmitoyl-2-oleoyl-GPC (16:0/18:1)	Lipid	Phosphatidylcholine (PC)	0.95	0.40	0.72	1.00	0.98	0.93	0.99	1.00	0.98	0.55	0.93	1.00	1.03	0.15	0.66	1.00	1.03	0.53	0.79	1.00	1.00	0.83	0.95	1.00
1-palmitoyl-2-oleoyl-GPI (16:0/18:1)*	Lipid	Phosphatidylinositol (PI)	0.86	0.03	0.23	1.00	0.99	0.54	0.83	1.00	0.95	0.51	0.93	1.00	1.15	0.02	0.37	1.00	1.11	0.01	0.26	1.00	0.96	0.99	1.00	1.00
1-palmitoyl-2-palmitoleoyl-GPC (16:0/16:1)*	Lipid	Phosphatidylcholine (PC)	0.90	0.27	0.61	1.00	0.97	0.98	1.00	1.00	0.98	0.93	1.00	1.00	1.07	0.14	0.66	1.00	1.08	0.16	0.56	1.00	1.01	0.68	0.89	1.00
1-palmitoyl-2-stearoyl-GPC (16:0/18:0)	Lipid	Phosphatidylcholine (PC)	0.97	0.39	0.71	1.00	0.98	0.60	0.86	1.00	0.99	0.75	0.95	1.00	1.01	0.88	0.97	1.00	1.02	0.35	0.69	1.00	1.01	0.68	0.89	1.00
1-palmitoyl-GPC (16:0)	Lipid	Lysophospholipid	0.94	0.26	0.60	1.00	0.98	0.70	0.90	1.00	0.99	0.80	0.96	1.00	1.04	0.16	0.66	1.00	1.05	0.08	0.51	1.00	1.01	0.52	0.83	1.00
1-palmitoyl-GPE (16:0)	Lipid	Lysophospholipid	0.89	0.03	0.22	1.00	0.92	0.10	0.46	1.00	0.97	0.52	0.93	1.00	1.03	0.23	0.75	1.00	1.10	0.11	0.53	1.00	1.06	0.21	0.68	1.00
1-palmitoyl-GPI* (16:0)	Lipid	Lysophospholipid	0.81	0.08	0.39	1.00	1.01	0.92	0.98	1.00	0.95	0.90	0.99	1.00	1.25	0.05	0.53	1.00	1.18	0.18	0.58	1.00	0.94	0.79	0.94	1.00
1-ribosyl-imidazoleacetate*	Amino Acid	Histidine Metabolism	1.01	0.93	0.97	1.00	1.00	0.37	0.74	1.00	1.05	0.88	0.99	1.00	0.99	0.14	0.66	1.00	1.04	0.41	0.72	1.00	1.05	0.11	0.64	1.00
1-stearoyl-2-arachidonoyl-GPC (18:0/20:4)	Lipid	Phosphatidylcholine (PC)	0.96	0.24	0.59	1.00	0.98	0.61	0.86	1.00	0.99	0.67	0.94	1.00	1.01	0.51	0.88	1.00	1.03	0.21	0.60	1.00	1.01	0.66	0.88	1.00
1-stearoyl-2-arachidonoyl-GPE (18:0/20:4)	Lipid	Phosphatidylethanolamine (PE)	0.87	0.00	0.05	1.00	0.94	0.19	0.59	1.00	0.96	0.38	0.85	1.00	1.08	0.02	0.37	1.00	1.11	0.00	0.14	1.00	1.03	0.52	0.83	1.00
1-stearoyl-2-arachidonoyl-GPI (18:0/20:4)	Lipid	Phosphatidylinositol (PI)	0.96	0.31	0.64	1.00	1.00	0.73	0.90	1.00	0.95	0.39	0.86	1.00	1.05	0.23	0.75	1.00	1.00	0.95	0.99	1.00	0.95	0.14	0.64	1.00
1-stearoyl-2-docosahexaenoyl-GPC (18:0/22:6)	Lipid	Phosphatidylcholine (PC)	1.03	0.29	0.63	1.00	1.00	0.64	0.87	1.00	1.00	0.99	1.00	1.00	0.97	0.52	0.88	1.00	0.97	0.48	0.76	1.00	1.00	1.00	1.00	1.00
1-stearoyl-2-docosahexaenoyl-GPE (18:0/22:6)*	Lipid	Phosphatidylethanolamine (PE)	0.92	0.06	0.34	1.00	0.95	0.58	0.86	1.00	0.93	0.10	0.51	1.00	1.04	0.31	0.81	1.00	1.02	0.40	0.72	1.00	0.98	0.60	0.86	1.00
1-stearoyl-2-linoleoyl-GPC (18:0/18:2)*	Lipid	Phosphatidylcholine (PC)	0.95	0.24	0.59	1.00	0.98	0.70	0.90	1.00	1.00	0.53	0.93	1.00	1.03	0.29	0.80	1.00	1.05	0.04	0.45	1.00	1.02	0.51	0.83	1.00
1-stearoyl-2-linoleoyl-GPE (18:0/18:2)*	Lipid	Phosphatidylethanolamine (PE)	0.69	0.00	0.01	0.11	0.82	0.03	0.32	1.00	0.82	0.01	0.20	1.00	1.18	0.01	0.23	1.00	1.18	0.00	0.17	1.00	1.00	0.81	0.94	1.00
1-stearoyl-2-linoleoyl-GPI (18:0/18:2)	Lipid	Phosphatidylinositol (PI)	0.92	0.19	0.54	1.00	1.04	0.63	0.87	1.00	1.00	0.68	0.94	1.00	1.13	0.01	0.26	1.00	1.08	0.03	0.42	1.00	0.96	0.45	0.80	1.00
1-stearoyl-2-oleoyl-GPC (18:0/18:1)	Lipid	Phosphatidylcholine (PC)	0.93	0.11	0.47	1.00	0.98	0.57	0.85	1.00	0.99	0.68	0.94	1.00	1.05	0.12	0.66	1.00	1.07	0.13	0.54	1.00	1.01	0.42	0.78	1.00
1-stearoyl-2-oleoyl-GPI (18:0/18:1)*	Lipid	Phosphatidylinositol (PI)	0.94	0.27	0.61	1.00	1.05	0.83	0.95	1.00	1.03	0.74	0.95	1.00	1.12	0.08	0.62	1.00	1.10	0.05	0.45	1.00	0.98	0.88	0.97	1.00
1-stearoyl-GPC (18:0)	Lipid	Lysophospholipid	0.95	0.30	0.63	1.00	0.99	0.89	0.98	1.00	1.01	0.81	0.97	1.00	1.04	0.12	0.65	1.00	1.06	0.09	0.52	1.00	1.02	0.50	0.82	1.00
1-stearoyl-GPE (18:0)	Lipid	Lysophospholipid	0.89	0.03	0.21	1.00	0.95	0.31	0.71	1.00	1.00	0.74	0.95	1.00	1.07	0.06	0.57	1.00	1.12	0.02	0.37	1.00	1.05	0.27	0.72	1.00
1-stearoyl-GPI (18:0)	Lipid	Lysophospholipid	1.05	0.69	0.89	1.00	1.11	0.16	0.55	1.00	1.07	0.41	0.89	1.00	1.05	0.19	0.72	1.00	1.02	0.46	0.75	1.00	0.97	0.74	0.91	1.00
2,3-dihydroxy-5-methylthio-4-pentenoate (DMTP	Amino Acid	Methionine, Cysteine, SAM and Tau	0.95	0.30	0.63	1.00	0.96	0.07	0.43	1.00	0.99	0.58	0.94	1.00	1.00	0.97	0.99	1.00	1.04	0.28	0.63	1.00	1.04	0.21	0.68	1.00
2-aminobutyrate	Amino Acid	Glutathione Metabolism	1.00	0.88	0.96	1.00	0.97	0.46	0.80	1.00	1.03	0.36	0.84	1.00	0.97	0.35	0.81	1.00	1.03	0.45	0.74	1.00	1.06	0.10	0.64	1.00
2-aminooctanoate	Lipid	Fatty Acid, Amino	0.90	0.16	0.52	1.00	1.00	0.67	0.88	1.00	1.12	0.45	0.90	1.00	1.10	0.24	0.76	1.00	1.24	0.09	0.51	1.00	1.12	0.17	0.66	1.00
2-aminophenol sulfate	Xenobiotics	Chemical	1.24	0.61	0.85	1.00	0.99	0.51	0.82	1.00	1.02	0.84	0.98	1.00	0.80	0.18	0.71	1.00	0.82	0.31	0.66	1.00	1.03	0.68	0.89	1.00
2-hydroxy-3-methylvalerate	Amino Acid	Leucine, Isoleucine and Valine Meta	0.83	0.11	0.47	1.00	0.89	0.40	0.76	1.00	0.90	0.43	0.89	1.00	1.06	0.60	0.91	1.00	1.08	0.18	0.58	1.00	1.01	0.61	0.87	1.00
2-hydroxybutyrate/2-hydroxyisobutyrate	Amino Acid	Glutathione Metabolism	0.93	0.23	0.58	1.00	0.88	0.03	0.28	1.00	0.91	0.11	0.54	1.00	0.95	0.06	0.59	1.00	0.99	0.41	0.72	1.00	1.04	0.90	0.97	1.00
2-hydroxydecanoate	Lipid	Fatty Acid, Monohydroxy	0.69	0.01	0.12	1.00	0.69	0.00	0.06	1.00	0.66	0.00	0.03	0.47	1.00	0.38	0.82	1.00	0.95	0.47	0.75	1.00	0.95	0.33	0.75	1.00
2-hydroxyglutarate	Lipid	Fatty Acid, Dicarboxylate	1.01	0.60	0.84	1.00	0.97	0.64	0.87	1.00	1.07	0.40	0.88	1.00	0.95	0.48	0.88	1.00	1.05	0.37	0.70	1.00	1.10	0.26	0.71	1.00
2-hydroxynervonate*	Lipid	Fatty Acid, Monohydroxy	0.99	0.70	0.90	1.00	0.90	0.09	0.45	1.00	0.94	0.29	0.81	1.00	0.92	0.32	0.81	1.00	0.95	0.49	0.77	1.00	1.04	0.59	0.86	1.00
2-hydroxyoctanoate	Lipid	Fatty Acid, Monohydroxy	0.84	0.02	0.16	1.00	0.94	0.27	0.66	1.00	1.32	0.34	0.83	1.00	1.12	0.43	0.84	1.00	1.57	0.18	0.58	1.00	1.40	0.70	0.90	1.00
2-hydroxypalmitate	Lipid	Fatty Acid, Monohydroxy	1.00	0.87	0.96	1.00	0.96	0.37	0.74	1.00	1.00	0.94	1.00	1.00	0.95	0.62	0.91	1.00	0.99	0.84	0.95	1.00	1.04	0.92	0.98	1.00
2-hydroxystearate	Lipid	Fatty Acid, Monohydroxy	0.94	0.33	0.66	1.00	0.95	0.54	0.83	1.00	1.01	0.79	0.96	1.00	1.01	0.58	0.90	1.00	1.08	0.17	0.57	1.00	1.07	0.50	0.82	1.00
2-methylcitrate/homocitrate	Energy	TCA Cycle	0.95	0.31	0.64	1.00	0.96	0.20	0.60	1.00	0.99	0.70	0.94	1.00	1.00	0.83	0.97	1.00	1.04	0.25	0.63	1.00	1.04	0.59	0.86	1.00
2-oleoylglycerol (18:1)	Lipid	Monoacylglycerol	0.79	0.19	0.54	1.00	0.86	0.50	0.81	1.00	0.87	0.75	0.95	1.00	1.08	0.21	0.72	1.00	1.09	0.20	0.59	1.00	1.01	0.83	0.95	1.00
2-palmitoyl-GPC* (16:0)*	Lipid	Lysophospholipid	0.90	0.41	0.73	1.00	0.84	0.18	0.58	1.00	0.98	0.90	0.99	1.00	0.93	0.80	0.97	1.00	1.09	0.66	0.85	1.00	1.17	0.32	0.75	1.00
2-piperidinone	Xenobiotics	Food Component/Plant	0.86	0.06	0.34	1.00	0.95	0.48	0.81	1.00	0.98	0.72	0.95	1.00	1.11	0.14	0.66	1.00	1.15	0.02	0.37	1.00	1.04	0.80	0.94	1.00
2-pyrrolidinone	Amino Acid	Glutamate Metabolism	0.99	0.75	0.92	1.00	0.96	0.44	0.79	1.00	1.20	0.00	0.03	0.38	0.97	0.51	0.88	1.00	1.22	0.00	0.10	0.51	1.25	0.00	0.04	0.21
2-stearoyl-GPE (18:0)*	Lipid	Lysophospholipid	0.88	0.02	0.19	1.00	0.84	0.02	0.26	1.00	0.97	0.50	0.93	1.00	0.96	0.66	0.92	1.00	1.11	0.15	0.55	1.00	1.15	0.03	0.48	1.00

3-(4-hydroxyphenyl)lactate (HPLA)	Amino Acid	Tyrosine Metabolism	0.87	0.04	0.26	1.00	0.90	0.05	0.37	1.00	0.93	0.14	0.58	1.00	1.04	0.36	0.81	1.00	1.08	0.03	0.44	1.00	1.03	0.38	0.78	1.00
3,4-dihydroxybutyrate	Amino Acid	Glutamate Metabolism	0.87	0.08	0.42	1.00	0.82	0.01	0.16	1.00	0.86	0.07	0.43	1.00	0.94	0.28	0.79	1.00	0.99	0.87	0.96	1.00	1.05	0.26	0.71	1.00
3-aminoisobutyrate	Nucleotide	Pyrimidine Metabolism, Thymine co	1.07	0.11	0.47	1.00	1.00	0.68	0.89	1.00	1.04	0.42	0.89	1.00	0.94	0.08	0.62	1.00	0.97	0.60	0.82	1.00	1.04	0.33	0.75	1.00
3beta,7alpha-dihydroxy-5-cholestenoate	Lipid	Sterol	0.96	0.23	0.58	1.00	0.94	0.18	0.58	1.00	1.02	1.00	1.00	1.00	0.98	0.25	0.76	1.00	1.06	0.52	0.79	1.00	1.08	0.21	0.68	1.00
3beta-hydroxy-5-cholestenoate	Lipid	Sterol	0.97	0.55	0.82	1.00	0.93	0.06	0.41	1.00	0.98	0.81	0.97	1.00	0.96	0.40	0.83	1.00	1.01	0.70	0.88	1.00	1.06	0.20	0.68	1.00
3-carboxy-4-methyl-5-pentyl-2-furanpropionate (3	Lipid	Fatty Acid, Dicarboxylate	1.04	0.67	0.88	1.00	1.00	0.87	0.97	1.00	1.00	0.89	0.99	1.00	0.96	0.61	0.91	1.00	0.95	0.83	0.95	1.00	1.00	0.98	1.00	1.00
3-carboxy-4-methyl-5-propyl-2-furanpropanoate (	Lipid	Fatty Acid, Dicarboxylate	0.92	0.17	0.52	1.00	0.89	0.05	0.38	1.00	0.88	0.11	0.54	1.00	0.97	0.60	0.91	1.00	0.96	0.46	0.75	1.00	0.99	0.99	1.00	1.00
3-formylindole	Xenobiotics	Food Component/Plant	0.91	0.14	0.48	1.00	0.96	0.58	0.86	1.00	0.95	0.30	0.81	1.00	1.05	0.35	0.81	1.00	1.04	0.32	0.67	1.00	0.99	0.89	0.97	1.00
3-hydroxy-2-ethylpropionate	Amino Acid	Leucine, Isoleucine and Valine Meta	0.95	0.22	0.56	1.00	0.94	0.15	0.54	1.00	0.92	0.02	0.28	1.00	0.99	0.51	0.88	1.00	0.97	0.46	0.75	1.00	0.98	0.66	0.88	1.00
3-hydroxy-3-methylglutarate	Lipid	Mevalonate Metabolism	0.86	0.14	0.50	1.00	0.88	0.17	0.57	1.00	0.90	0.39	0.87	1.00	1.02	0.62	0.91	1.00	1.04	0.45	0.74	1.00	1.02	0.83	0.95	1.00
3-hydroxybutyrate (BHBA)	Lipid	Ketone Bodies	1.26	0.26	0.60	1.00	1.00	0.79	0.94	1.00	0.75	0.13	0.57	1.00	0.79	0.07	0.59	1.00	0.60	0.04	0.45	1.00	0.75	0.20	0.68	1.00
3-hydroxydecanoate	Lipid	Fatty Acid, Monohydroxy	1.14	0.15	0.50	1.00	1.07	0.40	0.76	1.00	1.03	0.84	0.98	1.00	0.94	0.19	0.72	1.00	0.91	0.24	0.63	1.00	0.96	0.63	0.87	1.00
3-hydroxyhexanoate	Lipid	Fatty Acid, Monohydroxy	0.98	0.80	0.94	1.00	0.91	0.19	0.59	1.00	0.87	0.03	0.35	1.00	0.93	0.16	0.66	1.00	0.89	0.09	0.52	1.00	0.95	0.32	0.75	1.00
3-hydroxyhippurate	Xenobiotics	Benzoate Metabolism	1.14	0.28	0.62	1.00	1.29	0.34	0.74	1.00	1.35	0.43	0.89	1.00	1.14	0.97	0.99	1.00	1.18	0.97	0.99	1.00	1.04	0.53	0.83	1.00
3-hydroxylaurate	Lipid	Fatty Acid, Monohydroxy	1.40	0.00	0.07	1.00	1.32	0.02	0.22	1.00	1.20	0.04	0.35	1.00	0.94	0.34	0.81	1.00	0.86	0.14	0.55	1.00	0.91	0.35	0.78	1.00
3-hydroxyoctanoate	Lipid	Fatty Acid, Monohydroxy	1.02	0.72	0.91	1.00	0.97	0.54	0.83	1.00	0.93	0.14	0.60	1.00	0.95	0.24	0.76	1.00	0.91	0.17	0.57	1.00	0.96	0.33	0.75	1.00
3-hydroxyoleate*	Lipid	Fatty Acid, Monohydroxy	1.09	0.73	0.91	1.00	1.05	0.67	0.88	1.00	1.04	0.60	0.94	1.00	0.96	0.94	0.98	1.00	0.96	0.79	0.92	1.00	1.00	0.95	0.99	1.00
3-hydroxypyridine sulfate	Xenobiotics	Chemical	0.22	0.00	0.02	0.22	0.29	0.00	0.03	0.38	0.31	0.00	0.07	1.00	1.34	0.32	0.81	1.00	1.42	0.14	0.55	1.00	1.05	0.30	0.74	1.00
3-indoxyl sulfate	Amino Acid	Tryptophan Metabolism	0.91	0.44	0.75	1.00	0.97	0.43	0.78	1.00	0.98	0.98	1.00	1.00	1.07	0.19	0.72	1.00	1.08	0.52	0.79	1.00	1.01	0.60	0.86	1.00
3-methyl-2-oxobutyrate	Amino Acid	Leucine, Isoleucine and Valine Meta	0.95	0.19	0.54	1.00	0.99	0.61	0.86	1.00	1.00	0.95	1.00	1.00	1.04	0.22	0.75	1.00	1.06	0.09	0.52	1.00	1.02	0.92	0.98	1.00
3-methyl-2-oxovalerate	Amino Acid	Leucine, Isoleucine and Valine Meta	0.88	0.05	0.32	1.00	0.87	0.01	0.19	1.00	0.95	0.29	0.80	1.00	0.99	0.74	0.95	1.00	1.08	0.04	0.45	1.00	1.09	0.04	0.48	1.00
3-methylglutaconate	Amino Acid	Leucine, Isoleucine and Valine Meta	0.98	0.39	0.71	1.00	0.98	0.47	0.80	1.00	1.01	0.66	0.94	1.00	1.00	0.57	0.90	1.00	1.03	0.63	0.84	1.00	1.03	0.95	0.99	1.00
3-methylhistidine	Amino Acid	Histidine Metabolism	0.96	0.83	0.95	1.00	1.49	0.23	0.62	1.00	1.11	0.31	0.81	1.00	1.55	0.10	0.62	1.00	1.15	0.57	0.81	1.00	0.74	0.40	0.78	1.00
3-ureidopropionate	Nucleotide	Pyrimidine Metabolism, Uracil cont	0.99	0.95	0.98	1.00	0.94	0.52	0.82	1.00	1.06	0.39	0.87	1.00	0.95	0.62	0.91	1.00	1.07	0.33	0.68	1.00	1.13	0.00	0.28	1.00
4-acetamidobutanoate	Amino Acid	Polyamine Metabolism	0.97	0.24	0.59	1.00	0.97	0.41	0.77	1.00	0.97	0.36	0.84	1.00	1.00	0.76	0.95	1.00	1.00	0.89	0.96	1.00	1.00	0.64	0.88	1.00
4-allylphenol sulfate	Xenobiotics	Food Component/Plant	0.60	0.02	0.17	1.00	1.06	0.99	1.00	1.00	0.84	0.15	0.61	1.00	1.76	0.00	0.10	1.00	1.41	0.16	0.56	1.00	0.80	0.39	0.78	1.00
4-ethylphenyl sulfate	Xenobiotics	Benzoate Metabolism	0.78	0.02	0.18	1.00	0.93	0.10	0.46	1.00	1.14	0.04	0.35	1.00	1.19	0.48	0.88	1.00	1.46	0.92	0.98	1.00	1.22	0.88	0.97	1.00
4-hydroxychlorothalonil	Xenobiotics	Chemical	0.91	0.07	0.38	1.00	0.93	0.22	0.61	1.00	0.93	0.11	0.52	1.00	1.02	0.55	0.90	1.00	1.02	0.75	0.91	1.00	1.00	0.94	0.99	1.00
4-hydroxyphenylpyruvate	Amino Acid	Tyrosine Metabolism	0.76	0.00	0.06	1.00	0.83	0.03	0.28	1.00	0.81	0.04	0.35	1.00	1.09	0.08	0.62	1.00	1.07	0.10	0.52	1.00	0.98	0.93	0.98	1.00
4-methyl-2-oxopentanoate	Amino Acid	Leucine, Isoleucine and Valine Meta	0.91	0.10	0.46	1.00	0.94	0.26	0.66	1.00	0.98	0.81	0.97	1.00	1.02	0.66	0.92	1.00	1.07	0.05	0.45	1.00	1.05	0.42	0.78	1.00
4-methylcatechol sulfate	Xenobiotics	Benzoate Metabolism	1.10	0.34	0.67	1.00	1.20	0.72	0.90	1.00	1.30	0.50	0.93	1.00	1.09	0.90	0.98	1.00	1.18	0.81	0.94	1.00	1.08	0.50	0.82	1.00
4-vinylphenol sulfate	Xenobiotics	Benzoate Metabolism	0.86	0.05	0.31	1.00	1.07	0.12	0.48	1.00	1.26	0.55	0.93	1.00	1.25	0.29	0.80	1.00	1.46	0.10	0.52	1.00	1.17	0.59	0.86	1.00
5,6-dihydrouridine	Nucleotide	Pyrimidine Metabolism, Uracil cont	1.00	0.79	0.93	1.00	1.01	0.79	0.94	1.00	1.04	0.29	0.80	1.00	1.01	0.76	0.95	1.00	1.04	0.14	0.55	1.00	1.03	0.41	0.78	1.00
5-acetylamino-6-amino-3-methyluracil	Xenobiotics	Xanthine Metabolism	0.98	0.63	0.86	1.00	0.97	1.00	1.00	1.00	1.08	0.33	0.82	1.00	0.99	0.42	0.84	1.00	1.10	0.33	0.68	1.00	1.12	0.21	0.68	1.00
5-dodecenoate (12:1n7)	Lipid	Medium Chain Fatty Acid	1.30	0.00	0.04	1.00	1.31	0.05	0.38	1.00	1.15	0.10	0.51	1.00	1.01	0.98	0.99	1.00	0.89	0.06	0.47	1.00	0.88	0.10	0.64	1.00
5-dodecenoylcarnitine (C12:1)	Lipid	Fatty Acid Metabolism(Acyl Carnitin	1.26	0.02	0.15	1.00	1.26	0.02	0.25	1.00	1.12	0.21	0.71	1.00	1.00	0.94	0.98	1.00	0.89	0.16	0.56	1.00	0.89	0.27	0.72	1.00
5-hydroxylysine	Amino Acid	Lysine Metabolism	1.03	0.69	0.89	1.00	0.97	0.74	0.91	1.00	1.02	0.48	0.92	1.00	0.95	0.33	0.81	1.00	1.00	0.58	0.81	1.00	1.05	0.20	0.68	1.00
5-methylthioadenosine (MTA)	Amino Acid	Polyamine Metabolism	0.98	0.88	0.96	1.00	0.95	0.50	0.81	1.00	0.98	0.47	0.92	1.00	0.97	0.50	0.88	1.00	0.99	0.97	0.99	1.00	1.03	0.51	0.83	1.00
5-methylthioribose	Amino Acid	Methionine, Cysteine, SAM and Tau	1.01	0.89	0.96	1.00	0.99	0.92	0.98	1.00	1.02	0.62	0.94	1.00	0.98	0.38	0.82	1.00	1.01	0.50	0.77	1.00	1.03	0.31	0.75	1.00
5-methyluridine (ribothymidine)	Nucleotide	Pyrimidine Metabolism, Uracil cont	0.99	0.95	0.98	1.00	0.99	0.99	1.00	1.00	1.02	0.69	0.94	1.00	1.00	0.68	0.93	1.00	1.03	0.54	0.80	1.00	1.03	0.41	0.78	1.00
5-oxoproline	Amino Acid	Glutathione Metabolism	0.99	0.97	0.98	1.00	0.96	0.23	0.61	1.00	1.02	0.74	0.95	1.00	0.97	0.28	0.79	1.00	1.04	0.47	0.75	1.00	1.06	0.03	0.47	1.00
6-bromotryptophan	Amino Acid	Tryptophan Metabolism	0.93	0.27	0.61	1.00	0.94	0.29	0.68	1.00	0.99	0.90	0.99	1.00	1.01	0.85	0.97	1.00	1.07	0.09	0.52	1.00	1.06	0.21	0.68	1.00
6-hydroxyindole sulfate	Xenobiotics	Chemical	0.95	0.97	0.98	1.00	1.00	0.29	0.69	1.00	0.99	0.83	0.98	1.00	1.05	0.36	0.81	1.00	1.04	0.64	0.85	1.00	0.99	0.39	0.78	1.00
6-oxopiperidine-2-carboxylate	Amino Acid	Lysine Metabolism	0.78	0.00	0.04	1.00	0.81	0.01	0.17	1.00	0.79	0.00	0.09	1.00	1.03	0.54	0.89	1.00	1.01	0.50	0.77	1.00	0.98	0.93	0.98	1.00
7-HOCA	Lipid	Sterol	0.94	0.13	0.48	1.00	0.95	0.21	0.61	1.00	1.00	0.70	0.94	1.00	1.01	0.84	0.97	1.00	1.07	0.25	0.63	1.00	1.05	0.27	0.72	1.00
7-methylguanine	Nucleotide	Purine Metabolism, Guanine contai	0.97	0.57	0.82	1.00	0.99	0.83	0.95	1.00	1.04	0.52	0.93	1.00	1.01	0.57	0.90	1.00	1.06	0.12	0.54	1.00	1.05	0.22	0.68	1.00
9-hydroxystearate	Lipid	Fatty Acid, Monohydroxy	0.94	0.41	0.73	1.00	1.05	0.95	1.00	1.00	0.91	0.32	0.82	1.00	1.12	0.12	0.65	1.00	0.97	0.61	0.83	1.00	0.87	0.17	0.66	1.00
acetylcarnitine (C2)	Lipid	Fatty Acid Metabolism(Acyl Carnitin	0.99	0.80	0.94	1.00	0.97	0.24	0.64	1.00	0.92	0.17	0.64	1.00	0.99	0.67	0.92	1.00	0.93	0.18	0.58	1.00	0.94	0.39	0.78	1.00
acisoga	Amino Acid	Polyamine Metabolism	1.12	0.02	0.18	1.00	1.04	0.11	0.46	1.00	1.12	0.04	0.35	1.00	0.93	0.06	0.59	1.00	1.00	0.85	0.95	1.00	1.07	0.17	0.66	1.00
aconitate [cis or trans]	Energy	TCA Cycle	0.93	0.21	0.55	1.00	0.94	0.16	0.55	1.00	0.94	0.09	0.48	1.00	1.02	0.63	0.92	1.00	1.01	0.89	0.96	1.00	0.99	0.80	0.94	1.00
adrenate (22:4n6)	Lipid	Polyunsaturated Fatty Acid (n3 and	1.24	0.22	0.56	1.00	1.20	0.14	0.53	1.00	1.07	0.25	0.76	1.00	0.97	0.95	0.99	1.00	0.86	0.61	0.83	1.00	0.89	0.45	0.80	1.00
alanine	Amino Acid	Alanine and Aspartate Metabolism	0.94	0.46	0.76	1.00	0.95	0.42	0.78	1.00	0.99	0.75	0.95	1.00	1.01	0.81	0.97	1.00	1.06	0.21	0.60	1.00	1.05	0.21	0.68	1.00
allantoin	Nucleotide	Purine Metabolism, (Hypo)Xanthine	0.95	0.48	0.77	1.00	0.85	0.06	0.41	1.00	0.94	0.52	0.93	1.00	0.90	0.10	0.62	1.00	0.99	0.79	0.92	1.00	1.10	0.05	0.52	1.00
alpha-hydroxyisocaproate	Amino Acid	Leucine, Isoleucine and Valine Meta	0.90	0.23	0.57	1.00	0.90	0.10	0.46	1.00	0.97	0.99	1.00	1.00	1.00	0.94	0.98	1.00	1.08	0.06	0.47	1.00	1.08	0.07	0.59	1.00

alpha-hydroxyisovalerate	Amino Acid	Leucine, Isoleucine and Valine Meta	1.02	0.66	0.87	1.00	0.99	0.88	0.97	1.00	1.03	0.93	1.00	1.00	0.96	0.50	0.88	1.00	1.00	0.99	0.99	1.00	1.04	0.93	0.98	1.00
alpha-ketoglutaramate*	Amino Acid	Glutamate Metabolism	0.94	0.20	0.55	1.00	0.90	0.05	0.37	1.00	0.94	0.16	0.63	1.00	0.95	0.14	0.66	1.00	0.99	0.95	0.99	1.00	1.05	0.19	0.68	1.00
alpha-ketoglutarate	Energy	TCA Cycle	0.96	0.93	0.97	1.00	0.95	0.95	1.00	1.00	0.94	0.74	0.95	1.00	0.99	0.60	0.91	1.00	0.97	0.90	0.97	1.00	0.98	0.99	1.00	1.00
alpha-tocopherol	Cofactors anc	Tocopherol Metabolism	0.98	0.88	0.96	1.00	0.93	0.48	0.81	1.00	0.96	0.80	0.96	1.00	0.95	0.50	0.88	1.00	0.98	0.99	0.99	1.00	1.04	0.49	0.82	1.00
androstenediol (3beta,17beta) disulfate (1)	Lipid	Androgenic Steroids	0.97	0.75	0.92	1.00	0.88	0.54	0.83	1.00	1.03	0.54	0.93	1.00	0.91	0.60	0.91	1.00	1.06	0.14	0.55	1.00	1.17	0.02	0.47	1.00
androstenediol (3beta,17beta) disulfate (2)	Lipid	Androgenic Steroids	1.01	0.76	0.92	1.00	0.94	0.45	0.80	1.00	1.02	0.80	0.96	1.00	0.93	0.13	0.66	1.00	1.01	0.59	0.82	1.00	1.08	0.13	0.64	1.00
androstenediol (3beta,17beta) monosulfate (1)	Lipid	Androgenic Steroids	0.90	0.48	0.77	1.00	0.91	0.85	0.96	1.00	0.95	0.19	0.67	1.00	1.01	0.33	0.81	1.00	1.05	0.63	0.84	1.00	1.04	0.69	0.90	1.00
androsterone glucuronide	Lipid	Androgenic Steroids	0.92	0.88	0.96	1.00	0.95	1.00	1.00	1.00	1.03	0.34	0.83	1.00	1.03	0.81	0.97	1.00	1.12	0.49	0.77	1.00	1.09	0.21	0.68	1.00
androsterone sulfate	Lipid	Androgenic Steroids	1.01	0.89	0.96	1.00	0.97	0.64	0.87	1.00	0.97	0.69	0.94	1.00	0.96	0.85	0.97	1.00	0.96	0.94	0.98	1.00	1.00	0.42	0.78	1.00
arabitol/xylitol	Carbohydrate	Pentose Metabolism	0.93	0.14	0.48	1.00	0.90	0.03	0.32	1.00	0.98	0.59	0.94	1.00	0.98	0.83	0.97	1.00	1.06	0.33	0.68	1.00	1.09	0.25	0.71	1.00
arabonate/xylonate	Carbohydrate	Pentose Metabolism	0.89	0.09	0.42	1.00	0.89	0.09	0.45	1.00	0.94	0.51	0.93	1.00	1.01	0.87	0.97	1.00	1.06	0.39	0.71	1.00	1.05	0.31	0.75	1.00
arachidate (20:0)	Lipid	Long Chain Fatty Acid	1.00	0.95	0.98	1.00	0.87	0.08	0.44	1.00	0.89	0.08	0.46	1.00	0.87	0.05	0.53	1.00	0.89	0.13	0.54	1.00	1.02	0.66	0.88	1.00
arachidonate (20:4n6)	Lipid	Polyunsaturated Fatty Acid (n3 and	0.94	0.81	0.94	1.00	0.97	0.62	0.87	1.00	0.99	0.84	0.98	1.00	1.03	0.48	0.88	1.00	1.05	0.20	0.59	1.00	1.02	1.00	1.00	1.00
arachidonoylcarnitine (C20:4)	Lipid	Fatty Acid Metabolism(Acyl Carnitin	1.03	0.93	0.97	1.00	1.09	0.03	0.29	1.00	1.11	0.01	0.19	1.00	1.06	0.10	0.62	1.00	1.08	0.06	0.46	1.00	1.02	0.62	0.87	1.00
arachidonoylcholine	Lipid	Fatty Acid Metabolism (Acyl Cholin	0.92	0.20	0.55	1.00	1.15	0.18	0.58	1.00	1.03	0.87	0.98	1.00	1.25	0.00	0.04	0.18	1.12	0.03	0.42	1.00	0.89	0.17	0.66	1.00
argininate*	Amino Acid	Urea cycle; Arginine and Proline Met	0.84	0.08	0.41	1.00	0.88	0.57	0.85	1.00	0.97	0.85	0.98	1.00	1.05	0.59	0.91	1.00	1.16	0.02	0.37	1.00	1.11	0.12	0.64	1.00
arginine	Amino Acid	Urea cycle; Arginine and Proline Met	1.00	0.76	0.92	1.00	0.98	0.80	0.94	1.00	1.02	0.42	0.89	1.00	0.98	0.50	0.88	1.00	1.02	0.61	0.83	1.00	1.04	0.20	0.68	1.00
asparagine	Amino Acid	Alanine and Aspartate Metabolism	0.98	0.95	0.98	1.00	0.98	1.00	1.00	1.00	1.03	0.32	0.82	1.00	0.99	0.85	0.97	1.00	1.05	0.23	0.61	1.00	1.05	0.06	0.56	1.00
aspartate	Amino Acid	Alanine and Aspartate Metabolism	1.00	0.72	0.91	1.00	0.95	0.58	0.86	1.00	0.97	0.60	0.94	1.00	0.96	0.31	0.81	1.00	0.98	0.89	0.96	1.00	1.02	0.33	0.75	1.00
azelate (nonanedioate; C9)	Lipid	Fatty Acid, Dicarboxylate	0.75	0.02	0.15	1.00	0.74	0.01	0.16	1.00	0.73	0.02	0.27	1.00	0.98	0.69	0.93	1.00	0.98	0.47	0.75	1.00	0.99	0.94	0.99	1.00
behenoyl dihydrosphingomyelin (d18:0/22:0)*	Lipid	Dihydrosphingomyelins	0.93	0.33	0.66	1.00	0.96	0.79	0.94	1.00	0.98	0.69	0.94	1.00	1.03	0.90	0.98	1.00	1.05	0.32	0.67	1.00	1.02	0.84	0.95	1.00
behenoyl sphingomyelin (d18:1/22:0)*	Lipid	Sphingomyelins	0.96	0.31	0.64	1.00	0.96	0.60	0.86	1.00	0.97	0.43	0.89	1.00	1.00	0.98	0.99	1.00	1.01	0.51	0.78	1.00	1.01	0.70	0.90	1.00
beta-alanine	Nucleotide	Pyrimidine Metabolism, Uracil cont	1.01	0.89	0.96	1.00	0.87	0.01	0.16	1.00	1.07	0.09	0.48	1.00	0.86	0.00	0.14	1.00	1.06	0.21	0.61	1.00	1.23	0.00	0.00	0.00
beta-citrylglutamate	Amino Acid	Glutamate Metabolism	1.06	0.17	0.52	1.00	0.98	0.64	0.87	1.00	1.00	0.75	0.95	1.00	0.93	0.06	0.57	1.00	0.94	0.03	0.42	1.00	1.01	0.38	0.78	1.00
beta-cryptoxanthin	Cofactors anc	Vitamin A Metabolism	0.87	0.55	0.82	1.00	1.07	0.53	0.83	1.00	0.92	0.55	0.93	1.00	1.24	0.16	0.66	1.00	1.06	0.80	0.93	1.00	0.86	0.35	0.78	1.00
beta-hydroxyisovalerate	Amino Acid	Leucine, Isoleucine and Valine Meta	0.96	0.68	0.89	1.00	0.97	0.66	0.88	1.00	1.02	0.98	1.00	1.00	1.02	0.84	0.97	1.00	1.06	0.66	0.85	1.00	1.04	0.83	0.95	1.00
betaine	Amino Acid	Glycine, Serine and Threonine Metab	1.00	0.94	0.98	1.00	0.97	0.33	0.73	1.00	1.00	0.90	0.99	1.00	0.96	0.21	0.73	1.00	1.00	0.99	0.99	1.00	1.04	0.17	0.66	1.00
bilirubin (E,E)*	Cofactors anc	Hemoglobin and Porphyrin Metabo	1.23	0.00	0.04	1.00	1.21	0.01	0.16	1.00	1.13	0.03	0.31	1.00	0.99	0.89	0.97	1.00	0.92	0.20	0.59	1.00	0.93	0.34	0.77	1.00
bilirubin (E,Z or Z,E)*	Cofactors anc	Hemoglobin and Porphyrin Metabo	1.03	0.63	0.86	1.00	1.13	0.04	0.34	1.00	1.12	0.03	0.35	1.00	1.10	0.10	0.62	1.00	1.09	0.08	0.49	1.00	0.99	0.70	0.90	1.00
bilirubin	Cofactors anc	Hemoglobin and Porphyrin Metabo	1.01	0.78	0.93	1.00	1.05	0.20	0.60	1.00	1.05	0.29	0.81	1.00	1.04	0.21	0.72	1.00	1.04	0.33	0.68	1.00	1.00	0.92	0.98	1.00
biliverdin	Cofactors anc	Hemoglobin and Porphyrin Metabo	1.15	0.03	0.22	1.00	1.13	0.08	0.45	1.00	1.08	0.24	0.74	1.00	0.98	0.67	0.92	1.00	0.93	0.26	0.63	1.00	0.96	0.69	0.90	1.00
butyrylcarnitine (C4)	Lipid	Fatty Acid Metabolism (also BCAA M	0.95	0.41	0.73	1.00	0.95	0.10	0.46	1.00	0.99	0.33	0.82	1.00	1.00	0.94	0.98	1.00	1.04	0.70	0.88	1.00	1.05	0.99	1.00	1.00
caffeine	Xenobiotics	Xanthine Metabolism	0.47	0.00	0.03	0.89	0.53	0.00	0.05	1.00	0.66	0.06	0.40	1.00	1.12	0.50	0.88	1.00	1.39	0.10	0.53	1.00	1.24	0.14	0.65	1.00
caprate (10:0)	Lipid	Medium Chain Fatty Acid	0.72	0.04	0.29	1.00	0.81	0.20	0.60	1.00	0.68	0.00	0.07	1.00	1.13	0.35	0.81	1.00	0.95	0.28	0.63	1.00	0.84	0.05	0.52	1.00
carnitine	Lipid	Carnitine Metabolism	0.96	0.32	0.65	1.00	0.97	0.60	0.86	1.00	0.96	0.34	0.83	1.00	1.01	0.73	0.94	1.00	1.00	0.75	0.91	1.00	1.00	0.97	0.99	1.00
carotene diol (1)	Cofactors anc	Vitamin A Metabolism	0.80	0.00	0.04	1.00	0.87	0.78	0.93	1.00	0.83	0.07	0.41	1.00	1.09	0.18	0.72	1.00	1.03	0.25	0.63	1.00	0.95	0.49	0.82	1.00
carotene diol (2)	Cofactors anc	Vitamin A Metabolism	0.85	0.03	0.21	1.00	0.88	0.85	0.96	1.00	0.90	0.60	0.94	1.00	1.04	0.09	0.62	1.00	1.06	0.08	0.49	1.00	1.02	0.83	0.95	1.00
catechol sulfate	Xenobiotics	Benzoate Metabolism	0.74	0.02	0.18	1.00	0.77	0.07	0.42	1.00	1.21	0.89	0.99	1.00	1.04	0.81	0.97	1.00	1.64	0.03	0.42	1.00	1.58	0.15	0.66	1.00
ceramide (d16:1/24:1, d18:1/22:1)*	Lipid	Ceramides	0.96	0.37	0.69	1.00	0.98	0.76	0.92	1.00	1.01	0.98	1.00	1.00	1.02	0.69	0.93	1.00	1.05	0.29	0.64	1.00	1.03	0.80	0.94	1.00
ceramide (d18:1/14:0, d16:1/16:0)*	Lipid	Ceramides	0.93	0.29	0.63	1.00	0.99	0.88	0.97	1.00	0.96	0.45	0.90	1.00	1.07	0.06	0.59	1.00	1.03	0.43	0.73	1.00	0.97	0.47	0.82	1.00
ceramide (d18:1/20:0, d16:1/22:0, d20:1/18:0	Lipid	Ceramides	0.97	0.66	0.87	1.00	0.91	0.06	0.41	1.00	0.96	0.73	0.95	1.00	0.95	0.29	0.79	1.00	1.00	0.99	0.99	1.00	1.05	0.26	0.71	1.00
ceramide (d18:2/24:1, d18:1/24:2)*	Lipid	Ceramides	0.99	0.62	0.85	1.00	0.99	0.98	1.00	1.00	1.02	0.76	0.95	1.00	1.00	0.90	0.98	1.00	1.03	0.69	0.87	1.00	1.03	0.81	0.94	1.00
cerotoylcarnitine (C26)*	Lipid	Fatty Acid Metabolism(Acyl Carnitin	0.93	0.07	0.38	1.00	0.97	0.22	0.61	1.00	0.95	0.38	0.85	1.00	1.04	0.21	0.72	1.00	1.02	0.19	0.58	1.00	0.98	0.87	0.97	1.00
C-glycosyltryptophan	Amino Acid	Tryptophan Metabolism	1.02	0.89	0.96	1.00	0.97	0.42	0.78	1.00	1.04	0.51	0.93	1.00	0.95	0.29	0.80	1.00	1.02	0.40	0.72	1.00	1.07	0.07	0.57	1.00
cholate	Lipid	Primary Bile Acid Metabolism	0.99	0.95	0.98	1.00	0.88	0.74	0.91	1.00	0.65	0.92	1.00	1.00	0.89	0.37	0.81	1.00	0.65	0.20	0.59	1.00	0.74	0.48	0.82	1.00
cholesterol	Lipid	Sterol	0.96	0.48	0.77	1.00	0.97	0.49	0.81	1.00	0.93	0.04	0.37	1.00	1.02	0.60	0.91	1.00	0.97	0.30	0.65	1.00	0.95	0.21	0.68	1.00
choline	Lipid	Phospholipid Metabolism	1.00	0.64	0.86	1.00	1.00	0.61	0.86	1.00	1.04	0.29	0.80	1.00	1.00	0.95	0.99	1.00	1.04	0.32	0.67	1.00	1.04	0.29	0.73	1.00
cinnamoylglycine	Xenobiotics	Food Component/Plant	1.10	0.13	0.47	1.00	0.83	0.54	0.83	1.00	0.99	0.35	0.83	1.00	0.75	0.01	0.27	1.00	0.89	0.40	0.72	1.00	1.18	0.02	0.47	1.00
cis-4-decenoate (10:1n6)*	Lipid	Medium Chain Fatty Acid	1.20	0.02	0.15	1.00	1.19	0.03	0.31	1.00	1.13	0.22	0.72	1.00	0.99	0.84	0.97	1.00	0.94	0.39	0.71	1.00	0.96	0.14	0.65	1.00
cis-4-decenoylcarnitine (C10:1)	Lipid	Fatty Acid Metabolism(Acyl Carnitin	1.07	0.33	0.66	1.00	1.08	0.29	0.68	1.00	1.03	0.58	0.94	1.00	1.01	0.64	0.92	1.00	0.97	0.72	0.88	1.00	0.96	0.53	0.83	1.00
citraconate/glutaconate	Energy	TCA Cycle	0.29	0.00	0.01	0.03	0.34	0.00	0.01	0.01	0.40	0.00	0.05	1.00	1.14	0.88	0.97	1.00	1.36	0.13	0.54	1.00	1.20	0.36	0.78	1.00
citrate	Energy	TCA Cycle	1.02	0.39	0.71	1.00	0.97	0.27	0.66	1.00	0.99	0.34	0.83	1.00	0.95	0.22	0.75	1.00	0.97	0.33	0.68	1.00	1.02	0.39	0.78	1.00
citrulline	Amino Acid	Urea cycle; Arginine and Proline Met	0.99	0.92	0.97	1.00	0.99	0.72	0.90	1.00	1.04	0.20	0.68	1.00	1.00	0.85	0.97	1.00	1.05	0.29	0.64	1.00	1.05	0.17	0.66	1.00
cortisol	Lipid	Corticosteroids	1.03	0.74	0.92	1.00	1.00	0.98	1.00	1.00	1.25	0.00	0.09	1.00	0.97	0.68	0.93	1.00	1.22	0.00	0.07	0.21	1.26	0.00	0.00	0.00

creatine	Amino Acid	Creatine Metabolism	0.91	0.14	0.48	1.00	0.96	0.88	0.97	1.00	0.96	0.64	0.94	1.00	1.05	0.14	0.66	1.00	1.06	0.14	0.55	1.00	1.00	0.72	0.91	1.00
creatinine	Amino Acid	Creatine Metabolism	0.97	0.47	0.77	1.00	0.98	0.43	0.78	1.00	1.01	0.70	0.94	1.00	1.00	0.97	0.99	1.00	1.04	0.23	0.61	1.00	1.03	0.21	0.68	1.00
cys-gly, oxidized	Amino Acid	Glutathione Metabolism	1.04	0.26	0.60	1.00	0.98	0.81	0.95	1.00	1.10	0.05	0.37	1.00	0.95	0.15	0.66	1.00	1.06	0.24	0.63	1.00	1.12	0.01	0.37	1.00
cysteine	Amino Acid	Methionine, Cysteine, SAM and Tau	0.91	0.48	0.77	1.00	0.95	0.85	0.96	1.00	0.97	0.90	0.99	1.00	1.05	0.10	0.62	1.00	1.07	0.14	0.55	1.00	1.02	0.78	0.94	1.00
cysteine-glutathione disulfide	Amino Acid	Glutathione Metabolism	1.02	0.67	0.88	1.00	1.05	0.60	0.86	1.00	1.19	0.02	0.27	1.00	1.03	0.84	0.97	1.00	1.17	0.05	0.45	1.00	1.14	0.04	0.48	1.00
cysteinylglycine disulfide*	Amino Acid	Glutathione Metabolism	1.00	1.00	1.00	1.00	0.96	0.39	0.76	1.00	1.05	0.29	0.81	1.00	0.97	0.16	0.66	1.00	1.05	0.12	0.54	1.00	1.09	0.01	0.44	1.00
cystine	Amino Acid	Methionine, Cysteine, SAM and Tau	1.07	0.62	0.85	1.00	1.07	0.62	0.87	1.00	1.08	0.28	0.79	1.00	1.01	0.72	0.94	1.00	1.01	0.73	0.89	1.00	1.01	0.88	0.97	1.00
decanoylcarnitine (C10)	Lipid	Fatty Acid Metabolism(Acyl Carnitin	1.21	0.09	0.42	1.00	1.09	0.54	0.83	1.00	1.09	0.24	0.74	1.00	0.90	0.33	0.81	1.00	0.90	0.16	0.56	1.00	1.00	0.89	0.97	1.00
dehydroepiandrosterone sulfate (DHEA-S)	Lipid	Androgenic Steroids	0.96	0.93	0.97	1.00	0.95	0.81	0.95	1.00	0.96	0.57	0.94	1.00	0.99	0.95	0.99	1.00	1.00	0.79	0.92	1.00	1.01	0.90	0.97	1.00
deoxycarnitine	Lipid	Carnitine Metabolism	0.96	0.49	0.77	1.00	0.97	0.46	0.80	1.00	1.01	0.67	0.94	1.00	1.00	0.87	0.97	1.00	1.05	0.03	0.43	1.00	1.05	0.13	0.64	1.00
diacylglycerol (12:0/18:1, 14:0/16:1, 16:0/14:1	Lipid	Diacylglycerol	0.61	0.00	0.08	1.00	0.67	0.09	0.45	1.00	0.74	0.17	0.64	1.00	1.10	0.33	0.81	1.00	1.21	0.03	0.43	1.00	1.10	0.40	0.78	1.00
diacylglycerol (14:0/18:1, 16:0/16:1) [1]*	Lipid	Diacylglycerol	0.76	0.00	0.08	1.00	0.78	0.04	0.34	1.00	0.81	0.14	0.58	1.00	1.04	0.50	0.88	1.00	1.07	0.11	0.53	1.00	1.04	0.38	0.78	1.00
diacylglycerol (14:0/18:1, 16:0/16:1) [2]*	Lipid	Diacylglycerol	0.73	0.00	0.08	1.00	0.79	0.06	0.41	1.00	0.82	0.23	0.73	1.00	1.07	0.39	0.83	1.00	1.11	0.14	0.55	1.00	1.04	0.40	0.78	1.00
diacylglycerol (16:1/18:2 [2], 16:0/18:3 [1])*	Lipid	Diacylglycerol	0.85	0.16	0.52	1.00	0.87	0.43	0.78	1.00	0.92	0.76	0.95	1.00	1.02	0.59	0.91	1.00	1.08	0.28	0.63	1.00	1.05	0.36	0.78	1.00
dihomolinoleate (20:2n6)	Lipid	Polyunsaturated Fatty Acid (n3 and	1.23	0.25	0.60	1.00	1.14	0.22	0.61	1.00	1.04	0.76	0.95	1.00	0.92	0.41	0.83	1.00	0.84	0.23	0.61	1.00	0.91	0.20	0.68	1.00
dihomolinolenate (20:3n3 or 3n6)	Lipid	Polyunsaturated Fatty Acid (n3 and	0.91	0.55	0.82	1.00	1.01	0.54	0.83	1.00	1.00	0.69	0.94	1.00	1.11	0.09	0.62	1.00	1.09	0.15	0.56	1.00	0.98	0.48	0.82	1.00
dihomo-linolenoyl-choline	Lipid	Fatty Acid Metabolism (Acyl Cholin	0.90	0.15	0.50	1.00	1.17	0.16	0.55	1.00	1.04	0.72	0.95	1.00	1.29	0.00	0.10	1.00	1.15	0.02	0.37	1.00	0.89	0.41	0.78	1.00
dihydroorotate	Nucleotide	Pyrimidine Metabolism, Orotate co	0.93	0.42	0.73	1.00	0.92	0.43	0.78	1.00	0.96	0.97	1.00	1.00	0.98	0.73	0.94	1.00	1.02	0.76	0.92	1.00	1.04	0.12	0.64	1.00
dimethylarginine (ADMA + SDMA)	Amino Acid	Urea cycle; Arginine and Proline Me	1.03	0.32	0.65	1.00	1.01	0.53	0.83	1.00	1.07	0.04	0.37	1.00	0.98	0.35	0.81	1.00	1.04	0.24	0.63	1.00	1.05	0.11	0.64	1.00
dimethylglycine	Amino Acid	Glycine, Serine and Threonine Meta	0.97	0.79	0.93	1.00	0.92	0.17	0.57	1.00	0.97	0.57	0.94	1.00	0.95	0.09	0.62	1.00	1.00	1.00	1.00	1.00	1.05	0.07	0.60	1.00
docosadienoate (22:2n6)	Lipid	Polyunsaturated Fatty Acid (n3 and	1.16	0.22	0.56	1.00	1.14	0.04	0.35	1.00	0.98	0.95	1.00	1.00	0.98	0.92	0.98	1.00	0.84	0.06	0.45	1.00	0.85	0.01	0.37	1.00
docosahexaenoate (DHA; 22:6n3)	Lipid	Polyunsaturated Fatty Acid (n3 and	1.05	0.54	0.82	1.00	1.03	0.98	1.00	1.00	1.02	0.63	0.94	1.00	0.98	0.98	0.99	1.00	0.97	0.55	0.80	1.00	0.99	0.89	0.97	1.00
docosahexaenoylcarnitine (C22:6)*	Lipid	Fatty Acid Metabolism(Acyl Carnitin	1.06	0.44	0.75	1.00	1.08	0.28	0.67	1.00	1.06	0.33	0.82	1.00	1.02	0.60	0.91	1.00	1.01	0.81	0.94	1.00	0.99	0.16	0.66	1.00
docosahexaenoylcholine	Lipid	Fatty Acid Metabolism (Acyl Cholin	0.94	0.31	0.64	1.00	1.15	0.14	0.54	1.00	1.05	0.52	0.93	1.00	1.22	0.00	0.10	1.00	1.12	0.04	0.45	1.00	0.92	0.33	0.75	1.00
docosapentaenoate (DPA; 22:5n3)	Lipid	Polyunsaturated Fatty Acid (n3 and	1.15	0.25	0.60	1.00	1.09	0.32	0.72	1.00	1.02	0.64	0.94	1.00	0.95	0.64	0.92	1.00	0.89	0.26	0.63	1.00	0.94	0.40	0.78	1.00
dodecadienoate (12:2)*	Lipid	Fatty Acid, Dicarboxylate	1.11	0.08	0.41	1.00	1.06	0.35	0.74	1.00	1.08	0.15	0.61	1.00	0.95	0.16	0.66	1.00	0.97	0.37	0.70	1.00	1.01	0.98	1.00	1.00
dodecanedioate (C12)	Lipid	Fatty Acid, Dicarboxylate	0.82	0.11	0.47	1.00	0.87	0.10	0.46	1.00	0.78	0.08	0.46	1.00	1.06	0.66	0.92	1.00	0.95	0.58	0.81	1.00	0.90	0.29	0.73	1.00
dodecenedioate (C12:1-DC)*	Lipid	Fatty Acid, Dicarboxylate	0.99	0.72	0.91	1.00	0.95	0.37	0.74	1.00	0.87	0.06	0.40	1.00	0.96	0.26	0.78	1.00	0.88	0.33	0.68	1.00	0.92	0.49	0.82	1.00
dopamine 3-O-sulfate	Amino Acid	Tyrosine Metabolism	0.46	0.01	0.12	1.00	0.43	0.01	0.13	1.00	0.51	0.01	0.23	1.00	0.94	0.62	0.91	1.00	1.10	0.11	0.53	1.00	1.17	0.73	0.91	1.00
eicosanedioate (C20-DC)	Lipid	Fatty Acid, Dicarboxylate	0.87	0.08	0.39	1.00	0.86	0.05	0.37	1.00	1.05	0.61	0.94	1.00	1.00	0.64	0.92	1.00	1.21	0.00	0.17	1.00	1.21	0.01	0.44	1.00
eicosapentaenoate (EPA; 20:5n3)	Lipid	Polyunsaturated Fatty Acid (n3 and	0.95	0.69	0.89	1.00	0.96	0.44	0.79	1.00	0.95	0.36	0.84	1.00	1.01	0.85	0.97	1.00	1.00	0.98	0.99	1.00	0.99	0.97	0.99	1.00
eicosenoate (20:1n9 or 1n11)	Lipid	Long Chain Fatty Acid	1.25	0.17	0.52	1.00	1.22	0.06	0.39	1.00	1.03	0.44	0.90	1.00	0.97	0.81	0.97	1.00	0.83	0.05	0.45	1.00	0.85	0.03	0.48	1.00
ergothioneine	Xenobiotics	Food Component/Plant	0.99	0.64	0.86	1.00	0.88	0.11	0.47	1.00	0.89	0.23	0.73	1.00	0.89	0.53	0.89	1.00	0.91	0.62	0.83	1.00	1.02	0.49	0.82	1.00
erucate (22:1n9)	Lipid	Long Chain Fatty Acid	1.07	0.45	0.76	1.00	0.96	0.66	0.88	1.00	0.94	0.53	0.93	1.00	0.90	0.34	0.81	1.00	0.88	0.25	0.63	1.00	0.97	0.98	1.00	1.00
erythritol	Xenobiotics	Food Component/Plant	0.49	0.49	0.77	1.00	0.48	0.22	0.61	1.00	0.55	0.57	0.94	1.00	0.99	0.93	0.98	1.00	1.12	0.18	0.58	1.00	1.13	0.02	0.44	1.00
erythronate*	Carbohydrate	Aminosugar Metabolism	0.93	0.05	0.30	1.00	0.93	0.09	0.45	1.00	0.98	0.67	0.94	1.00	1.00	0.94	0.98	1.00	1.06	0.18	0.58	1.00	1.05	0.05	0.52	1.00
ethylmalonate	Amino Acid	Leucine, Isoleucine and Valine Meta	0.96	0.58	0.83	1.00	0.92	0.34	0.74	1.00	0.95	0.59	0.94	1.00	0.96	0.28	0.79	1.00	0.99	0.66	0.85	1.00	1.03	0.49	0.82	1.00
etiocholanolone glucuronide	Lipid	Androgenic Steroids	0.99	0.76	0.92	1.00	0.97	0.93	0.99	1.00	1.04	0.40	0.88	1.00	0.98	0.18	0.72	1.00	1.06	0.16	0.56	1.00	1.08	0.04	0.48	1.00
Fibrinopeptide A (2-15)	Peptide	Fibrinogen Cleavage Peptide	0.99	0.85	0.95	1.00	0.94	0.04	0.33	1.00	1.00	0.49	0.92	1.00	0.94	0.09	0.62	1.00	1.01	0.83	0.95	1.00	1.07	0.11	0.64	1.00
Fibrinopeptide A (3-15)	Peptide	Fibrinogen Cleavage Peptide	1.03	0.43	0.74	1.00	0.99	0.64	0.87	1.00	1.03	0.90	0.99	1.00	0.96	0.20	0.72	1.00	1.00	0.73	0.89	1.00	1.04	0.34	0.77	1.00
Fibrinopeptide A (3-16)	Peptide	Fibrinogen Cleavage Peptide	1.00	0.89	0.96	1.00	0.95	0.76	0.92	1.00	1.04	0.85	0.98	1.00	0.95	0.48	0.88	1.00	1.04	0.47	0.75	1.00	1.09	0.29	0.74	1.00
Fibrinopeptide A (4-15)	Peptide	Fibrinogen Cleavage Peptide	0.98	0.72	0.91	1.00	1.03	1.00	1.00	1.00	1.05	0.64	0.94	1.00	1.05	0.31	0.81	1.00	1.07	0.33	0.68	1.00	1.02	0.31	0.75	1.00
Fibrinopeptide A (5-16)*	Peptide	Fibrinogen Cleavage Peptide	1.03	0.94	0.98	1.00	0.96	0.51	0.82	1.00	1.03	0.76	0.95	1.00	0.94	0.51	0.88	1.00	1.01	0.72	0.88	1.00	1.07	0.25	0.71	1.00
Fibrinopeptide A (7-16)*	Peptide	Fibrinogen Cleavage Peptide	1.10	0.26	0.60	1.00	0.98	0.61	0.86	1.00	1.05	0.74	0.95	1.00	0.89	0.10	0.62	1.00	0.95	0.70	0.88	1.00	1.07	0.21	0.68	1.00
Fibrinopeptide A (8-16)	Peptide	Fibrinogen Cleavage Peptide	1.12	0.16	0.52	1.00	1.03	0.97	1.00	1.00	1.04	0.64	0.94	1.00	0.92	0.14	0.66	1.00	0.93	0.43	0.73	1.00	1.01	0.62	0.87	1.00
DSGEGDFXAEGGGVR*	Peptide	Fibrinogen Cleavage Peptide	1.03	0.85	0.95	1.00	0.98	0.81	0.95	1.00	1.05	0.47	0.92	1.00	0.95	0.83	0.97	1.00	1.02	0.55	0.80	1.00	1.07	0.33	0.75	1.00
Fibrinopeptide B (1-12)	Peptide	Fibrinogen Cleavage Peptide	1.01	0.58	0.83	1.00	0.96	0.07	0.42	1.00	0.98	0.63	0.94	1.00	0.95	0.07	0.61	1.00	0.97	0.79	0.92	1.00	1.02	0.37	0.78	1.00
Fibrinopeptide B (1-13)	Peptide	Fibrinogen Cleavage Peptide	1.02	0.43	0.74	1.00	0.96	0.50	0.81	1.00	1.02	0.95	1.00	1.00	0.95	0.26	0.78	1.00	1.00	0.79	0.92	1.00	1.06	0.15	0.66	1.00
fructose	Carbohydrate	Fructose, Mannose and Galactose M	0.90	0.18	0.53	1.00	0.96	0.40	0.76	1.00	0.95	0.55	0.93	1.00	1.07	0.12	0.66	1.00	1.06	0.32	0.67	1.00	0.99	0.94	0.99	1.00
gamma-CEHC	Cofactors an	Tocopherol Metabolism	0.86	0.36	0.68	1.00	0.99	0.70	0.90	1.00	1.02	0.70	0.94	1.00	1.15	0.14	0.66	1.00	1.18	0.31	0.66	1.00	1.03	0.95	0.99	1.00
gamma-glutamylalanine	Peptide	Gamma-glutamyl Amino Acid	0.98	0.83	0.95	1.00	0.99	0.64	0.87	1.00	0.99	0.89	0.99	1.00	1.01	0.81	0.97	1.00	1.01	0.42	0.73	1.00	1.00	0.88	0.97	1.00
gamma-glutamyl-alpha-lysine	Peptide	Gamma-glutamyl Amino Acid	0.99	0.89	0.96	1.00	0.97	0.55	0.84	1.00	1.04	0.25	0.76	1.00	0.98	0.61	0.91	1.00	1.06	0.12	0.54	1.00	1.08	0.02	0.44	1.00
gamma-glutamylcitrulline*	Peptide	Gamma-glutamyl Amino Acid	0.99	0.73	0.91	1.00	0.98	0.64	0.87	1.00	1.07	0.35	0.83	1.00	1.00	0.80	0.97	1.00	1.09	0.13	0.55	1.00	1.09	0.09	0.62	1.00
gamma-glutamyl-epsilon-lysine	Peptide	Gamma-glutamyl Amino Acid	0.95	0.70	0.90	1.00	0.98	0.73	0.90	1.00	1.00	1.00	1.00	1.00	1.03	0.42	0.84	1.00	1.05	0.26	0.63	1.00	1.02	0.69	0.90	1.00

gamma-glutamylglutamine	Peptide	Gamma-glutamyl Amino Acid	1.01	0.92	0.97	1.00	0.97	0.50	0.81	1.00	1.05	0.32	0.82	1.00	0.96	0.35	0.81	1.00	1.04	0.38	0.70	1.00	1.08	0.03	0.48	1.00
gamma-glutamylglycine	Peptide	Gamma-glutamyl Amino Acid	1.03	0.79	0.93	1.00	1.00	0.74	0.91	1.00	1.03	0.49	0.92	1.00	0.97	0.90	0.98	1.00	1.00	0.68	0.86	1.00	1.03	0.39	0.78	1.00
gamma-glutamylhistidine	Peptide	Gamma-glutamyl Amino Acid	0.97	0.94	0.98	1.00	1.03	0.61	0.86	1.00	1.04	0.57	0.94	1.00	1.06	0.12	0.65	1.00	1.08	0.06	0.47	1.00	1.02	0.79	0.94	1.00
gamma-glutamylisoleucine*	Peptide	Gamma-glutamyl Amino Acid	0.91	0.24	0.59	1.00	0.92	0.16	0.56	1.00	0.99	0.79	0.96	1.00	1.01	0.54	0.89	1.00	1.09	0.06	0.45	1.00	1.08	0.14	0.65	1.00
gamma-glutamylleucine	Peptide	Gamma-glutamyl Amino Acid	0.81	0.00	0.05	1.00	0.86	0.01	0.16	1.00	0.92	0.07	0.42	1.00	1.06	0.21	0.73	1.00	1.13	0.03	0.43	1.00	1.06	0.29	0.74	1.00
gamma-glutamylmethionine	Peptide	Gamma-glutamyl Amino Acid	0.94	0.85	0.95	1.00	0.93	0.48	0.81	1.00	0.97	0.66	0.94	1.00	0.98	0.48	0.88	1.00	1.02	0.60	0.82	1.00	1.04	0.13	0.64	1.00
gamma-glutamylthreonine	Peptide	Gamma-glutamyl Amino Acid	0.97	0.72	0.91	1.00	0.94	0.45	0.80	1.00	1.01	0.99	1.00	1.00	0.97	0.59	0.91	1.00	1.04	0.38	0.70	1.00	1.08	0.17	0.66	1.00
gamma-glutamylvaline	Peptide	Gamma-glutamyl Amino Acid	0.94	0.25	0.60	1.00	0.97	0.69	0.89	1.00	1.03	0.85	0.98	1.00	1.04	0.29	0.80	1.00	1.10	0.01	0.32	1.00	1.06	0.09	0.62	1.00
gamma-tocopherol/beta-tocopherol	Cofactors and	Tocopherol Metabolism	1.06	0.40	0.72	1.00	1.02	0.64	0.87	1.00	1.06	0.32	0.82	1.00	0.96	0.84	0.97	1.00	1.00	0.85	0.95	1.00	1.04	0.98	1.00	1.00
gluconate	Xenobiotics	Food Component/Plant	0.94	0.49	0.77	1.00	0.92	0.18	0.58	1.00	0.99	0.64	0.94	1.00	0.98	0.47	0.88	1.00	1.05	0.46	0.75	1.00	1.07	0.24	0.70	1.00
glucose	Carbohydrate	Glycolysis, Gluconeogenesis, and Py	1.01	0.94	0.98	1.00	1.00	0.72	0.90	1.00	1.05	0.10	0.50	1.00	0.99	0.40	0.83	1.00	1.04	0.11	0.53	1.00	1.05	0.04	0.48	1.00
glucuronate	Carbohydrate	Aminosugar Metabolism	0.96	0.08	0.40	1.00	1.03	0.26	0.66	1.00	0.91	0.03	0.35	1.00	1.08	0.21	0.72	1.00	0.95	0.69	0.87	1.00	0.88	0.66	0.88	1.00
glu-gly-asn-val	Peptide	Polypeptide	0.84	0.55	0.82	1.00	0.84	0.23	0.61	1.00	0.84	0.51	0.93	1.00	1.00	0.39	0.83	1.00	0.92	0.98	1.00	1.00	1.00	0.54	0.84	1.00
glutamate	Amino Acid	Glutamate Metabolism	1.09	0.09	0.42	1.00	1.08	0.13	0.51	1.00	1.03	0.36	0.84	1.00	0.99	0.94	0.98	1.00	0.95	0.53	0.79	1.00	0.95	0.40	0.78	1.00
glutamine	Amino Acid	Glutamate Metabolism	1.00	0.70	0.90	1.00	0.98	0.38	0.75	1.00	1.03	0.15	0.61	1.00	0.98	0.36	0.81	1.00	1.03	0.63	0.84	1.00	1.05	0.12	0.64	1.00
glycerate	Carbohydrate	Glycolysis, Gluconeogenesis, and Py	0.96	0.49	0.77	1.00	0.91	0.08	0.44	1.00	0.96	0.15	0.62	1.00	0.95	0.16	0.66	1.00	1.00	0.72	0.88	1.00	1.05	0.18	0.68	1.00
glycerol	Lipid	Glycerolipid Metabolism	1.07	0.23	0.57	1.00	1.08	0.39	0.76	1.00	0.96	0.43	0.89	1.00	1.01	0.74	0.95	1.00	0.89	0.02	0.38	1.00	0.88	0.08	0.61	1.00
glycerophosphorylcholine (GPC)	Lipid	Phospholipid Metabolism	1.05	0.87	0.96	1.00	1.05	0.54	0.83	1.00	1.18	0.13	0.58	1.00	1.00	0.36	0.81	1.00	1.13	0.03	0.43	1.00	1.13	0.02	0.44	1.00
glycine	Amino Acid	Glycine, Serine and Threonine Metab	1.04	0.30	0.63	1.00	1.00	0.97	1.00	1.00	1.03	0.35	0.83	1.00	0.97	0.39	0.83	1.00	0.99	0.67	0.85	1.00	1.02	0.44	0.80	1.00
glycochenodeoxycholate	Lipid	Primary Bile Acid Metabolism	0.57	0.01	0.12	1.00	0.81	0.26	0.66	1.00	0.72	0.21	0.71	1.00	1.43	0.06	0.57	1.00	1.28	0.06	0.45	1.00	0.89	0.97	0.99	1.00
glycocholate	Lipid	Primary Bile Acid Metabolism	0.68	0.06	0.35	1.00	0.84	0.41	0.77	1.00	0.69	0.18	0.67	1.00	1.23	0.16	0.66	1.00	1.02	0.26	0.63	1.00	0.82	0.27	0.72	1.00
glycocholenate sulfate*	Lipid	Secondary Bile Acid Metabolism	1.04	0.70	0.90	1.00	0.95	0.41	0.77	1.00	1.06	0.63	0.94	1.00	0.92	0.18	0.72	1.00	1.02	0.99	0.99	1.00	1.12	0.05	0.52	1.00
glycolithocholate sulfate*	Lipid	Secondary Bile Acid Metabolism	0.98	0.49	0.77	1.00	0.84	0.02	0.24	1.00	0.87	0.07	0.42	1.00	0.86	0.16	0.66	1.00	0.88	0.29	0.65	1.00	1.03	0.81	0.94	1.00
glycosyl ceramide (d18:2/24:1, d18:1/24:2)*	Lipid	Hexosylceramides (HCER)	0.98	0.97	0.98	1.00	0.99	0.84	0.96	1.00	0.97	0.30	0.81	1.00	1.00	0.81	0.97	1.00	0.98	0.16	0.56	1.00	0.98	0.16	0.66	1.00
glycosyl-N-palmitoyl-sphingosine (d18:1/16:0)	Lipid	Hexosylceramides (HCER)	0.99	0.60	0.84	1.00	0.94	0.33	0.73	1.00	0.93	0.24	0.75	1.00	0.95	0.64	0.92	1.00	0.94	0.48	0.76	1.00	0.99	0.60	0.86	1.00
glycosyl-N-stearoyl-sphingosine (d18:1/18:0)	Lipid	Hexosylceramides (HCER)	0.96	0.51	0.79	1.00	0.94	0.33	0.73	1.00	0.96	0.42	0.89	1.00	0.98	0.60	0.91	1.00	1.00	0.62	0.83	1.00	1.02	0.94	0.99	1.00
guaiacol sulfate	Xenobiotics	Benzoate Metabolism	0.77	0.15	0.50	1.00	0.79	0.12	0.48	1.00	1.12	0.78	0.96	1.00	1.03	0.93	0.98	1.00	1.44	0.11	0.53	1.00	1.41	0.10	0.64	1.00
guanidinoacetate	Amino Acid	Creatine Metabolism	1.07	0.28	0.62	1.00	0.95	0.40	0.76	1.00	1.03	0.49	0.92	1.00	0.89	0.01	0.22	1.00	0.97	0.35	0.69	1.00	1.09	0.08	0.60	1.00
guanosine	Nucleotide	Purine Metabolism, Guanine contai	1.10	0.39	0.71	1.00	1.16	0.06	0.41	1.00	1.07	0.64	0.94	1.00	1.05	0.76	0.95	1.00	0.97	0.98	0.99	1.00	0.92	0.57	0.86	1.00
heptenedioate (C7:1-DC)*	Lipid	Fatty Acid, Dicarboxylate	0.94	0.43	0.74	1.00	0.96	0.50	0.81	1.00	0.83	0.04	0.35	1.00	1.02	0.90	0.98	1.00	0.88	0.03	0.42	1.00	0.87	0.05	0.52	1.00
hexadecadienoate (16:2n6)	Lipid	Polyunsaturated Fatty Acid (n3 and	1.16	0.10	0.45	1.00	1.02	0.97	1.00	1.00	1.04	0.63	0.94	1.00	0.88	0.03	0.47	1.00	0.90	0.06	0.45	1.00	1.02	0.95	0.99	1.00
hexadecanedioate (C16)	Lipid	Fatty Acid, Dicarboxylate	0.98	0.58	0.83	1.00	1.01	0.70	0.90	1.00	0.86	0.01	0.23	1.00	1.03	0.64	0.92	1.00	0.88	0.27	0.63	1.00	0.86	0.13	0.64	1.00
hexadecenedioate (C16:1-DC)*	Lipid	Fatty Acid, Dicarboxylate	0.86	0.04	0.29	1.00	0.85	0.03	0.27	1.00	0.81	0.00	0.10	1.00	0.99	0.21	0.72	1.00	0.95	0.87	0.96	1.00	0.96	0.90	0.97	1.00
hexanoylcarnitine (C6)	Lipid	Fatty Acid Metabolism(Acyl Carnitin	1.06	0.03	0.22	1.00	1.03	0.28	0.67	1.00	1.00	0.64	0.94	1.00	0.98	0.54	0.89	1.00	0.95	0.23	0.63	1.00	0.97	0.59	0.86	1.00
hexanoylglutamine	Lipid	Fatty Acid Metabolism (Acyl Glutam	1.10	0.10	0.44	1.00	0.94	0.66	0.88	1.00	0.89	0.33	0.82	1.00	0.85	0.06	0.59	1.00	0.81	0.12	0.54	1.00	0.95	0.51	0.83	1.00
hippurate	Xenobiotics	Benzoate Metabolism	0.90	0.61	0.85	1.00	0.85	0.21	0.61	1.00	1.02	1.00	1.00	1.00	0.94	0.53	0.89	1.00	1.14	0.28	0.63	1.00	1.21	0.27	0.72	1.00
histidine	Amino Acid	Histidine Metabolism	0.95	0.34	0.67	1.00	0.98	0.61	0.86	1.00	1.01	0.61	0.94	1.00	1.03	0.44	0.85	1.00	1.06	0.11	0.53	1.00	1.03	0.24	0.71	1.00
homoarginine	Amino Acid	Urea cycle; Arginine and Proline Met	0.99	0.62	0.85	1.00	1.03	0.78	0.93	1.00	1.07	0.17	0.64	1.00	1.04	0.67	0.92	1.00	1.08	0.14	0.55	1.00	1.04	0.11	0.64	1.00
homostachydrine*	Xenobiotics	Food Component/Plant	0.91	0.66	0.87	1.00	0.80	0.54	0.83	1.00	0.86	0.88	0.99	1.00	0.89	0.12	0.66	1.00	0.95	0.79	0.92	1.00	1.07	0.54	0.84	1.00
hydroxyasparagine	Amino Acid	Alanine and Aspartate Metabolism	1.01	0.70	0.90	1.00	0.99	0.92	0.98	1.00	1.02	0.36	0.84	1.00	0.98	0.21	0.72	1.00	1.01	0.68	0.86	1.00	1.03	0.25	0.71	1.00
hydroxy-CMPF*	Lipid	Fatty Acid, Dicarboxylate	0.95	0.10	0.45	1.00	0.94	0.17	0.57	1.00	0.94	0.16	0.63	1.00	0.99	0.66	0.92	1.00	0.99	0.83	0.95	1.00	1.00	0.57	0.86	1.00
hydroxy-N6,N6,N6-trimethyllysine*	Amino Acid	Lysine Metabolism	1.02	0.83	0.95	1.00	1.00	0.97	1.00	1.00	1.04	0.51	0.93	1.00	0.98	0.48	0.88	1.00	1.02	0.58	0.81	1.00	1.04	0.39	0.78	1.00
hydroxypalmitoyl sphingomyelin (d18:1/16:0(O	Lipid	Sphingomyelins	1.00	0.95	0.98	1.00	1.01	0.45	0.80	1.00	1.01	0.73	0.95	1.00	1.01	0.84	0.97	1.00	1.01	0.74	0.90	1.00	1.00	0.79	0.94	1.00
hypotaurine	Amino Acid	Methionine, Cysteine, SAM and Tau	0.98	0.76	0.92	1.00	0.95	0.33	0.73	1.00	1.02	0.79	0.96	1.00	0.98	0.61	0.91	1.00	1.04	0.54	0.80	1.00	1.07	0.11	0.64	1.00
hypoxanthine	Nucleotide	Purine Metabolism, (Hypo)Xanthine	1.06	0.24	0.59	1.00	1.01	0.72	0.90	1.00	1.08	0.06	0.40	1.00	0.94	0.33	0.81	1.00	1.01	0.54	0.80	1.00	1.07	0.21	0.68	1.00
imidazole lactate	Amino Acid	Histidine Metabolism	1.02	0.66	0.87	1.00	0.99	0.70	0.90	1.00	1.09	0.15	0.62	1.00	0.97	0.28	0.79	1.00	1.07	0.21	0.60	1.00	1.10	0.05	0.52	1.00
indoleacetate	Amino Acid	Tryptophan Metabolism	0.83	0.07	0.38	1.00	0.94	0.87	0.97	1.00	0.98	0.27	0.78	1.00	1.12	0.08	0.62	1.00	1.17	0.18	0.58	1.00	1.04	0.70	0.90	1.00
indolelactate	Amino Acid	Tryptophan Metabolism	0.92	0.09	0.44	1.00	0.94	0.17	0.57	1.00	1.01	0.97	1.00	1.00	1.02	0.61	0.91	1.00	1.10	0.03	0.43	1.00	1.08	0.20	0.68	1.00
indolepropionate	Amino Acid	Tryptophan Metabolism	1.05	0.47	0.77	1.00	0.86	0.05	0.37	1.00	1.29	0.03	0.35	1.00	0.82	0.22	0.75	1.00	1.23	0.03	0.43	1.00	1.50	0.00	0.03	0.09
inosine	Nucleotide	Purine Metabolism, (Hypo)Xanthine	1.19	0.09	0.44	1.00	1.26	0.05	0.38	1.00	1.11	0.44	0.90	1.00	1.06	0.78	0.95	1.00	0.93	0.69	0.87	1.00	0.88	0.47	0.82	1.00
isobutyrylcarnitine (C4)	Amino Acid	Leucine, Isoleucine and Valine Meta	0.74	0.00	0.02	0.55	0.82	0.08	0.44	1.00	0.87	0.10	0.51	1.00	1.11	0.05	0.53	1.00	1.16	0.03	0.43	1.00	1.05	0.51	0.83	1.00
isoleucine	Amino Acid	Leucine, Isoleucine and Valine Meta	0.93	0.35	0.67	1.00	0.92	0.10	0.46	1.00	0.97	0.62	0.94	1.00	0.99	0.89	0.97	1.00	1.04	0.34	0.69	1.00	1.05	0.20	0.68	1.00
isovalerylcarnitine (C5)	Amino Acid	Leucine, Isoleucine and Valine Meta	0.82	0.01	0.08	1.00	0.94	0.29	0.68	1.00	0.89	0.16	0.63	1.00	1.14	0.15	0.66	1.00	1.08	0.38	0.70	1.00	0.95	0.73	0.91	1.00
kynurenate	Amino Acid	Tryptophan Metabolism	0.91	0.11	0.47	1.00	0.96	0.57	0.85	1.00	0.97	0.99	1.00	1.00	1.06	0.15	0.66	1.00	1.06	0.14	0.55	1.00	1.01	0.80	0.94	1.00

kynurenine	Amino Acid	Tryptophan Metabolism	0.89	0.08	0.39	1.00	0.93	0.27	0.66	1.00	0.99	0.84	0.98	1.00	1.04	0.26	0.78	1.00	1.11	0.02	0.37	1.00	1.07	0.08	0.62	1.00
lactate	Carbohydrate	Glycolysis, Gluconeogenesis, and Py	1.02	0.61	0.85	1.00	0.98	0.80	0.94	1.00	1.02	0.47	0.92	1.00	0.97	0.37	0.81	1.00	1.01	0.79	0.92	1.00	1.04	0.08	0.60	1.00
lactosyl-N-nervonoyl-sphingosine (d18:1/24:1)*	Lipid	Lactosylceramides (LCER)	1.01	0.95	0.98	1.00	0.97	0.52	0.82	1.00	0.98	0.79	0.96	1.00	0.97	0.50	0.88	1.00	0.98	0.78	0.92	1.00	1.01	0.81	0.94	1.00
lactosyl-N-palmitoyl-sphingosine (d18:1/16:0)	Lipid	Lactosylceramides (LCER)	1.01	0.88	0.96	1.00	1.02	0.30	0.70	1.00	1.01	0.88	0.99	1.00	1.01	0.83	0.97	1.00	1.01	0.93	0.98	1.00	0.99	0.94	0.99	1.00
laurate (12:0)	Lipid	Medium Chain Fatty Acid	0.98	0.79	0.93	1.00	1.04	0.60	0.86	1.00	0.97	0.85	0.98	1.00	1.07	0.54	0.89	1.00	0.99	0.57	0.81	1.00	0.93	0.27	0.72	1.00
laurylcarnitine (C12)	Lipid	Fatty Acid Metabolism(Acyl Carnitin	1.17	0.11	0.47	1.00	1.20	0.12	0.49	1.00	1.03	0.60	0.94	1.00	1.03	0.50	0.88	1.00	0.88	0.14	0.55	1.00	0.86	0.04	0.48	1.00
leucine	Amino Acid	Leucine, Isoleucine and Valine Meta	0.95	0.72	0.91	1.00	0.96	0.69	0.89	1.00	0.98	0.97	1.00	1.00	1.02	0.69	0.93	1.00	1.04	0.24	0.63	1.00	1.02	0.35	0.78	1.00
lignoceroyl sphingomyelin (d18:1/24:0)	Lipid	Sphingomyelins	0.96	0.20	0.55	1.00	0.94	0.49	0.81	1.00	0.96	0.31	0.81	1.00	0.98	0.99	1.00	1.00	1.00	0.64	0.85	1.00	1.02	0.88	0.97	1.00
lignoceroylcarnitine (C24)*	Lipid	Fatty Acid Metabolism(Acyl Carnitin	0.92	0.05	0.29	1.00	0.99	0.23	0.61	1.00	0.98	0.16	0.63	1.00	1.07	0.04	0.50	1.00	1.06	0.14	0.55	1.00	0.99	0.57	0.86	1.00
linoleate (18:2n6)	Lipid	Polyunsaturated Fatty Acid (n3 and	1.08	0.54	0.82	1.00	1.06	0.45	0.80	1.00	1.00	0.95	1.00	1.00	0.99	0.88	0.97	1.00	0.93	0.30	0.65	1.00	0.94	0.30	0.74	1.00
linolenate (18:3n3 or 3n6)	Lipid	Polyunsaturated Fatty Acid (n3 and	1.10	0.37	0.69	1.00	1.07	0.55	0.84	1.00	0.96	0.75	0.95	1.00	0.97	0.80	0.97	1.00	0.87	0.06	0.46	1.00	0.90	0.06	0.57	1.00
linolenoylcarnitine (C18:3)*	Lipid	Fatty Acid Metabolism(Acyl Carnitin	1.11	0.04	0.28	1.00	1.18	0.00	0.08	1.00	1.11	0.06	0.40	1.00	1.07	0.24	0.76	1.00	1.00	0.79	0.92	1.00	0.94	0.24	0.70	1.00
linoleoyl-arachidonoyl-glycerol (18:2/20:4) [2]*	Lipid	Diacylglycerol	0.84	0.02	0.20	1.00	0.90	0.13	0.51	1.00	0.89	0.19	0.67	1.00	1.07	0.54	0.89	1.00	1.07	0.84	0.95	1.00	1.00	0.61	0.87	1.00
linoleoylcarnitine (C18:2)*	Lipid	Fatty Acid Metabolism(Acyl Carnitin	1.05	0.14	0.48	1.00	1.12	0.01	0.16	1.00	1.07	0.06	0.39	1.00	1.06	0.24	0.76	1.00	1.02	0.58	0.81	1.00	0.96	0.48	0.82	1.00
linoleoylcholine*	Lipid	Fatty Acid Metabolism (Acyl Cholin	0.85	0.10	0.45	1.00	1.13	0.43	0.78	1.00	1.02	0.98	1.00	1.00	1.32	0.00	0.04	0.06	1.19	0.01	0.26	1.00	0.90	0.39	0.78	1.00
linoleoyl-linoleoyl-glycerol (18:2/18:2) [1]*	Lipid	Diacylglycerol	0.94	0.14	0.49	1.00	0.96	0.42	0.78	1.00	0.96	0.62	0.94	1.00	1.02	0.90	0.98	1.00	1.02	0.99	0.99	1.00	1.00	0.74	0.91	1.00
lysine	Amino Acid	Lysine Metabolism	1.00	0.72	0.91	1.00	1.01	0.66	0.88	1.00	1.05	0.07	0.41	1.00	1.01	0.87	0.97	1.00	1.05	0.18	0.58	1.00	1.04	0.17	0.66	1.00
malate	Energy	TCA Cycle	0.98	0.74	0.92	1.00	0.96	0.73	0.90	1.00	1.04	0.80	0.96	1.00	0.98	0.39	0.82	1.00	1.06	0.30	0.65	1.00	1.09	0.04	0.48	1.00
mannitol/sorbitol	Carbohydrate	Fructose, Mannose and Galactose M	0.64	0.12	0.47	1.00	0.81	0.06	0.41	1.00	0.95	0.35	0.83	1.00	1.26	0.43	0.84	1.00	1.49	0.50	0.77	1.00	1.18	0.32	0.75	1.00
mannonate*	Xenobiotics	Food Component/Plant	0.98	0.76	0.92	1.00	0.97	0.54	0.83	1.00	1.00	0.99	1.00	1.00	0.98	0.57	0.90	1.00	1.02	0.48	0.76	1.00	1.04	0.28	0.72	1.00
mannose	Carbohydrate	Fructose, Mannose and Galactose M	0.98	0.64	0.86	1.00	0.98	0.44	0.79	1.00	1.06	0.13	0.57	1.00	0.99	0.41	0.83	1.00	1.07	0.05	0.45	1.00	1.08	0.00	0.28	1.00
margarate (17:0)	Lipid	Long Chain Fatty Acid	1.14	0.48	0.77	1.00	1.05	0.85	0.96	1.00	0.98	0.80	0.96	1.00	0.92	0.34	0.81	1.00	0.86	0.15	0.55	1.00	0.93	0.23	0.69	1.00
margaroylcarnitine (C17)*	Lipid	Fatty Acid Metabolism(Acyl Carnitin	0.99	0.60	0.84	1.00	1.00	0.75	0.92	1.00	0.97	0.49	0.92	1.00	1.01	0.87	0.97	1.00	0.98	0.42	0.73	1.00	0.97	0.67	0.89	1.00
methionine	Amino Acid	Methionine, Cysteine, SAM and Tau	0.96	0.87	0.96	1.00	0.97	0.67	0.88	1.00	1.01	0.55	0.93	1.00	1.01	0.81	0.97	1.00	1.06	0.14	0.55	1.00	1.05	0.08	0.61	1.00
methionine sulfone	Amino Acid	Methionine, Cysteine, SAM and Tau	0.97	0.74	0.92	1.00	1.01	0.78	0.93	1.00	1.03	0.70	0.94	1.00	1.04	0.16	0.66	1.00	1.06	0.15	0.55	1.00	1.02	0.51	0.83	1.00
methionine sulfoxide	Amino Acid	Methionine, Cysteine, SAM and Tau	0.85	0.06	0.34	1.00	0.86	0.03	0.32	1.00	0.87	0.11	0.52	1.00	1.01	0.61	0.91	1.00	1.03	0.26	0.63	1.00	1.01	0.50	0.82	1.00
methyl glucopyranoside (alpha + beta)	Xenobiotics	Food Component/Plant	0.74	0.02	0.18	1.00	0.74	0.19	0.59	1.00	0.93	0.87	0.98	1.00	1.00	0.85	0.97	1.00	1.25	0.24	0.63	1.00	1.26	0.40	0.78	1.00
methylsuccinate	Amino Acid	Leucine, Isoleucine and Valine Meta	0.86	0.01	0.13	1.00	0.86	0.03	0.27	1.00	0.91	0.11	0.53	1.00	1.00	0.75	0.95	1.00	1.05	0.52	0.79	1.00	1.06	0.52	0.83	1.00
myo-inositol	Lipid	Inositol Metabolism	0.89	0.13	0.47	1.00	0.89	0.08	0.44	1.00	1.01	0.74	0.95	1.00	0.99	0.93	0.98	1.00	1.13	0.08	0.49	1.00	1.14	0.25	0.71	1.00
myristate (14:0)	Lipid	Long Chain Fatty Acid	1.10	0.53	0.81	1.00	1.07	0.63	0.87	1.00	0.95	0.67	0.94	1.00	0.97	0.88	0.97	1.00	0.86	0.08	0.49	1.00	0.89	0.24	0.71	1.00
myristoleate (14:1n5)	Lipid	Long Chain Fatty Acid	1.23	0.01	0.12	1.00	1.25	0.05	0.37	1.00	1.11	0.34	0.83	1.00	1.02	0.85	0.97	1.00	0.90	0.05	0.45	1.00	0.89	0.08	0.60	1.00
myristoleoylcarnitine (C14:1)*	Lipid	Fatty Acid Metabolism(Acyl Carnitin	1.30	0.01	0.12	1.00	1.32	0.01	0.17	1.00	1.10	0.27	0.79	1.00	1.02	0.67	0.92	1.00	0.85	0.09	0.52	1.00	0.83	0.02	0.46	1.00
myristoyl dihydrosphingomyelin (d18:0/14:0)*	Lipid	Dihydrosphingomyelins	0.95	0.41	0.73	1.00	1.04	0.22	0.61	1.00	1.03	0.52	0.93	1.00	1.09	0.01	0.26	1.00	1.08	0.06	0.46	1.00	0.99	0.89	0.97	1.00
myristoylcarnitine (C14)	Lipid	Fatty Acid Metabolism(Acyl Carnitin	1.07	0.34	0.67	1.00	1.11	0.05	0.37	1.00	1.01	0.97	1.00	1.00	1.04	0.53	0.89	1.00	0.94	0.54	0.80	1.00	0.91	0.05	0.52	1.00
N,N,N-trimethyl-5-aminovalerate	Amino Acid	Lysine Metabolism	0.98	0.68	0.89	1.00	1.00	0.97	1.00	1.00	1.00	0.76	0.95	1.00	1.01	0.95	0.99	1.00	1.02	0.25	0.63	1.00	1.00	0.53	0.83	1.00
N,N,N-trimethyl-alanylproline betaine (TMAP)	Amino Acid	Urea cycle; Arginine and Proline Met	1.00	0.84	0.95	1.00	0.98	0.61	0.86	1.00	1.00	0.67	0.94	1.00	0.98	0.83	0.97	1.00	1.01	0.88	0.96	1.00	1.02	0.58	0.86	1.00
N1-Methyl-2-pyridone-5-carboxamide	Cofactors and	Nicotinate and Nicotinamide Metab	0.83	0.48	0.77	1.00	0.81	0.55	0.84	1.00	0.88	0.62	0.94	1.00	0.98	0.81	0.97	1.00	1.06	0.11	0.53	1.00	1.08	0.39	0.78	1.00
1-methyladenosine	Nucleotide	Purine Metabolism, Adenine contai	0.99	0.78	0.93	1.00	0.97	0.78	0.93	1.00	1.07	0.16	0.63	1.00	0.98	0.35	0.81	1.00	1.08	0.08	0.49	1.00	1.10	0.02	0.44	1.00
N1-methylinosine	Nucleotide	Purine Metabolism, (Hypo)Xanthine	1.07	0.48	0.77	1.00	1.03	0.48	0.81	1.00	1.15	0.08	0.45	1.00	0.97	0.37	0.81	1.00	1.08	0.53	0.79	1.00	1.11	0.14	0.64	1.00
N2,N2-dimethylguanosine	Nucleotide	Purine Metabolism, Guanine contai	1.06	0.38	0.70	1.00	1.02	0.85	0.96	1.00	1.14	0.05	0.39	1.00	0.96	0.05	0.55	1.00	1.07	0.38	0.70	1.00	1.12	0.02	0.44	1.00
N6,N6,N6-trimethyllysine	Amino Acid	Lysine Metabolism	1.01	0.54	0.82	1.00	0.97	0.75	0.92	1.00	1.04	0.27	0.78	1.00	0.96	0.80	0.97	1.00	1.03	0.10	0.52	1.00	1.07	0.14	0.64	1.00
N6,N6-dimethyllysine	Amino Acid	Lysine Metabolism	1.00	0.78	0.93	1.00	0.96	0.24	0.64	1.00	1.01	0.75	0.95	1.00	0.96	0.10	0.62	1.00	1.01	0.64	0.85	1.00	1.06	0.06	0.57	1.00
N6-acetyllysine	Amino Acid	Lysine Metabolism	0.94	0.21	0.55	1.00	0.94	0.10	0.46	1.00	0.98	0.49	0.92	1.00	1.01	0.83	0.97	1.00	1.05	0.16	0.56	1.00	1.04	0.10	0.64	1.00
N6-carbamoylthreonyladenosine	Nucleotide	Purine Metabolism, Adenine contai	1.00	0.97	0.98	1.00	1.04	0.12	0.49	1.00	1.04	0.18	0.66	1.00	1.04	0.11	0.62	1.00	1.04	0.27	0.63	1.00	1.00	0.73	0.91	1.00
N6-methyllysine	Amino Acid	Lysine Metabolism	0.96	0.30	0.63	1.00	0.95	0.68	0.89	1.00	0.98	0.66	0.94	1.00	0.99	0.59	0.91	1.00	1.03	0.21	0.60	1.00	1.04	0.05	0.52	1.00
N-acetyl-2-aminooctanoate*	Lipid	Fatty Acid, Amino	0.97	0.69	0.89	1.00	0.98	0.72	0.90	1.00	1.18	0.18	0.67	1.00	1.01	0.85	0.97	1.00	1.21	0.15	0.55	1.00	1.20	0.17	0.66	1.00
N-acetylalanine	Amino Acid	Alanine and Aspartate Metabolism	1.00	0.90	0.96	1.00	0.98	0.49	0.81	1.00	1.06	0.05	0.39	1.00	0.98	0.33	0.81	1.00	1.06	0.13	0.55	1.00	1.08	0.00	0.09	1.00
N-acetylarginine	Amino Acid	Urea cycle; Arginine and Proline Met	1.00	0.88	0.96	1.00	1.00	0.83	0.95	1.00	1.00	0.98	1.00	1.00	0.99	0.87	0.97	1.00	1.00	0.79	0.92	1.00	1.00	0.85	0.97	1.00
N-acetylasparagine	Amino Acid	Alanine and Aspartate Metabolism	0.98	0.85	0.95	1.00	0.99	0.46	0.80	1.00	1.02	0.99	1.00	1.00	1.01	0.76	0.95	1.00	1.04	0.75	0.91	1.00	1.03	0.58	0.86	1.00
N-acetyl-beta-alanine	Nucleotide	Pyrimidine Metabolism, Uracil cont	1.02	0.80	0.94	1.00	1.09	0.14	0.53	1.00	1.20	0.00	0.01	0.02	1.07	0.14	0.66	1.00	1.17	0.00	0.07	0.15	1.10	0.00	0.07	0.78
N-acetylglutamate	Amino Acid	Glutamate Metabolism	0.78	0.02	0.17	1.00	0.78	0.03	0.32	1.00	0.83	0.09	0.48	1.00	1.00	0.88	0.97	1.00	1.07	0.27	0.63	1.00	1.07	0.24	0.71	1.00
N-acetylglutamine	Amino Acid	Glutamate Metabolism	1.00	0.89	0.96	1.00	0.95	0.34	0.74	1.00	0.97	0.69	0.94	1.00	0.95	0.36	0.81	1.00	0.97	0.70	0.88	1.00	1.02	0.63	0.87	1.00
N-acetylglycine	Amino Acid	Glycine, Serine and Threonine Metab	1.03	0.33	0.66	1.00	0.99	0.97	1.00	1.00	0.90	0.27	0.79	1.00	0.97	0.61	0.91	1.00	0.88	0.05	0.45	1.00	0.91	0.14	0.65	1.00
N-acetyl-isoputreanine*	Amino Acid	Polyamine Metabolism	1.08	0.05	0.30	1.00	1.03	0.20	0.60	1.00	1.14	0.00	0.07	1.00	0.96	0.24	0.76	1.00	1.06	0.52	0.79	1.00	1.11	0.04	0.48	1.00

N-acetylleucine	Amino Acid	Leucine, Isoleucine and Valine Meta	0.83	0.01	0.15	1.00	0.86	0.16	0.55	1.00	0.87	0.01	0.21	1.00	1.04	0.72	0.94	1.00	1.05	0.41	0.72	1.00	1.01	0.88	0.97	1.00
N-acetylmethionine	Amino Acid	Methionine, Cysteine, SAM and Tau	0.97	0.49	0.77	1.00	0.96	0.25	0.65	1.00	1.01	0.84	0.98	1.00	0.99	0.68	0.93	1.00	1.04	0.18	0.58	1.00	1.05	0.05	0.52	1.00
N-acetylputrescine	Amino Acid	Polyamine Metabolism	1.00	0.85	0.95	1.00	0.99	0.99	1.00	1.00	1.00	0.64	0.94	1.00	0.99	0.66	0.92	1.00	1.00	0.67	0.85	1.00	1.01	0.68	0.89	1.00
N-acetylserine	Amino Acid	Glycine, Serine and Threonine Metak	0.99	0.93	0.97	1.00	1.00	0.97	1.00	1.00	1.04	0.35	0.83	1.00	1.01	0.76	0.95	1.00	1.05	0.45	0.74	1.00	1.03	0.33	0.75	1.00
N-acetyltaurine	Amino Acid	Methionine, Cysteine, SAM and Tau	0.99	0.98	0.99	1.00	0.96	0.73	0.90	1.00	1.01	0.79	0.96	1.00	0.97	0.52	0.88	1.00	1.02	0.80	0.93	1.00	1.04	0.16	0.66	1.00
N-acetylthreonine	Amino Acid	Glycine, Serine and Threonine Metak	1.00	0.88	0.96	1.00	0.99	0.57	0.85	1.00	1.04	0.37	0.84	1.00	0.99	0.81	0.97	1.00	1.05	0.21	0.61	1.00	1.06	0.17	0.66	1.00
N-acetyltryptophan	Amino Acid	Tryptophan Metabolism	0.89	0.18	0.53	1.00	0.92	0.43	0.78	1.00	1.00	0.84	0.98	1.00	1.03	0.39	0.83	1.00	1.13	0.03	0.42	1.00	1.09	0.13	0.64	1.00
N-acetylvaline	Amino Acid	Leucine, Isoleucine and Valine Meta	0.97	0.46	0.76	1.00	0.99	0.98	1.00	1.00	1.01	0.72	0.95	1.00	1.02	0.54	0.89	1.00	1.05	0.17	0.57	1.00	1.02	0.43	0.78	1.00
N-behenoyl-sphingadienine (d18:2/22:0)*	Lipid	Ceramides	0.97	0.64	0.86	1.00	0.98	0.51	0.82	1.00	1.00	0.90	0.99	1.00	1.01	0.83	0.97	1.00	1.03	0.44	0.74	1.00	1.02	0.57	0.86	1.00
N-delta-acetylornithine	Amino Acid	Urea cycle; Arginine and Proline Met	0.80	0.15	0.51	1.00	0.82	0.18	0.58	1.00	0.98	0.79	0.96	1.00	1.03	0.85	0.97	1.00	1.23	0.05	0.45	1.00	1.19	0.04	0.50	1.00
N-formylmethionine	Amino Acid	Methionine, Cysteine, SAM and Tau	0.95	0.27	0.61	1.00	0.96	0.27	0.66	1.00	1.03	0.66	0.94	1.00	1.01	0.72	0.94	1.00	1.07	0.04	0.45	1.00	1.06	0.04	0.48	1.00
N-methylpipecolate	Xenobiotics	Bacterial/Fungal	0.93	0.11	0.47	1.00	0.91	0.10	0.46	1.00	1.01	0.80	0.96	1.00	0.98	0.45	0.85	1.00	1.09	0.07	0.48	1.00	1.11	0.10	0.64	1.00
N-methylproline	Amino Acid	Urea cycle; Arginine and Proline Met	0.70	0.81	0.94	1.00	0.81	0.73	0.90	1.00	0.96	0.81	0.97	1.00	1.15	0.07	0.59	1.00	1.37	0.24	0.63	1.00	1.19	0.64	0.88	1.00
N-oleoylserine	Lipid	Endocannabinoid	0.99	0.78	0.93	1.00	0.96	0.48	0.81	1.00	0.96	0.53	0.93	1.00	0.97	0.66	0.92	1.00	0.98	0.52	0.79	1.00	1.00	0.74	0.91	1.00
N-oleoyltaurine	Lipid	Endocannabinoid	1.10	0.24	0.59	1.00	1.07	0.36	0.74	1.00	0.99	0.55	0.93	1.00	0.98	0.84	0.97	1.00	0.90	0.11	0.53	1.00	0.92	0.18	0.68	1.00
nonadecanoate (19:0)	Lipid	Long Chain Fatty Acid	1.07	0.47	0.77	1.00	1.03	0.46	0.80	1.00	0.97	0.99	1.00	1.00	0.96	0.69	0.93	1.00	0.91	0.29	0.64	1.00	0.94	0.19	0.68	1.00
nonanoylcarnitine (C9)	Lipid	Fatty Acid Metabolism(Acyl Carnitin	1.05	0.87	0.96	1.00	1.13	0.21	0.61	1.00	1.03	0.37	0.84	1.00	1.08	0.27	0.78	1.00	0.98	0.95	0.99	1.00	0.91	0.28	0.72	1.00
N-palmitoylglycine	Lipid	Fatty Acid Metabolism(Acyl Glycine)	1.10	0.33	0.66	1.00	1.03	0.60	0.86	1.00	1.04	0.44	0.90	1.00	0.94	0.46	0.87	1.00	0.94	0.40	0.72	1.00	1.01	1.00	1.00	1.00
N-palmitoyl-heptadecasphingosine (d17:1/16:0)	Lipid	Ceramides	0.96	0.41	0.73	1.00	0.97	0.76	0.92	1.00	0.97	0.24	0.74	1.00	1.01	0.87	0.97	1.00	1.01	0.87	0.96	1.00	1.00	0.80	0.94	1.00
N-palmitoyl-sphingadienine (d18:2/16:0)*	Lipid	Ceramides	0.99	0.59	0.84	1.00	1.02	0.59	0.86	1.00	0.98	0.38	0.85	1.00	1.02	0.38	0.82	1.00	0.99	0.47	0.75	1.00	0.97	0.21	0.68	1.00
N-palmitoyl-sphinganine (d18:0/16:0)	Lipid	Dihydroceramides	0.78	0.00	0.02	0.21	0.78	0.00	0.01	0.13	0.82	0.00	0.03	0.30	1.00	0.99	1.00	1.00	1.04	0.52	0.79	1.00	1.04	0.39	0.78	1.00
N-palmitoyl-sphingosine (d18:1/16:0)	Lipid	Ceramides	0.95	0.18	0.53	1.00	0.90	0.01	0.19	1.00	0.93	0.07	0.43	1.00	0.95	0.14	0.66	1.00	0.98	0.47	0.75	1.00	1.03	0.83	0.95	1.00
N-stearoyl-sphingadienine (d18:2/18:0)*	Lipid	Ceramides	0.98	0.32	0.65	1.00	0.91	0.04	0.34	1.00	0.94	0.21	0.71	1.00	0.93	0.09	0.62	1.00	0.96	0.49	0.77	1.00	1.04	0.42	0.78	1.00
N-stearoyl-sphingosine (d18:1/18:0)*	Lipid	Ceramides	1.00	0.99	1.00	1.00	0.92	0.07	0.42	1.00	0.94	0.23	0.73	1.00	0.92	0.04	0.52	1.00	0.95	0.34	0.69	1.00	1.03	0.49	0.82	1.00
octadecadienedioate (C18:2-DC)*	Lipid	Fatty Acid, Dicarboxylate	0.72	0.33	0.66	1.00	0.76	0.09	0.45	1.00	0.80	0.25	0.76	1.00	1.06	0.76	0.95	1.00	1.12	0.72	0.88	1.00	1.06	0.88	0.97	1.00
octadecanedioate (C18)	Lipid	Fatty Acid, Dicarboxylate	1.00	0.84	0.95	1.00	0.96	0.52	0.82	1.00	0.91	0.23	0.73	1.00	0.96	0.30	0.81	1.00	0.91	0.29	0.64	1.00	0.95	0.90	0.97	1.00
octadecanedioylcarnitine (C18-DC)*	Lipid	Fatty Acid Metabolism(Acyl Carnitin	1.02	0.34	0.67	1.00	1.02	0.87	0.97	1.00	1.00	0.90	0.99	1.00	1.00	0.99	1.00	1.00	0.98	0.60	0.82	1.00	0.98	0.80	0.94	1.00
octadecenedioate (C18:1-DC)*	Lipid	Fatty Acid, Dicarboxylate	0.73	0.00	0.05	1.00	0.71	0.00	0.01	0.14	0.67	0.00	0.00	0.00	0.97	0.55	0.90	1.00	0.92	0.18	0.58	1.00	0.94	0.55	0.85	1.00
octadecenedioylcarnitine (C18:1-DC)*	Lipid	Fatty Acid Metabolism(Acyl Carnitin	0.98	0.83	0.95	1.00	0.93	0.45	0.80	1.00	0.90	0.19	0.67	1.00	0.94	0.55	0.90	1.00	0.92	0.35	0.69	1.00	0.97	0.83	0.95	1.00
octanoylcarnitine (C8)	Lipid	Fatty Acid Metabolism(Acyl Carnitin	1.13	0.18	0.53	1.00	1.19	0.06	0.41	1.00	1.07	0.16	0.63	1.00	1.05	0.50	0.88	1.00	0.95	0.35	0.69	1.00	0.90	0.10	0.64	1.00
oleate/vaccenate (18:1)	Lipid	Long Chain Fatty Acid	1.15	0.17	0.52	1.00	1.11	0.24	0.64	1.00	1.06	0.45	0.90	1.00	0.96	0.37	0.81	1.00	0.91	0.09	0.51	1.00	0.95	0.26	0.71	1.00
oleoyl ethanolamide	Lipid	Endocannabinoid	1.21	0.08	0.41	1.00	1.02	0.76	0.92	1.00	1.05	0.57	0.94	1.00	0.85	0.02	0.33	1.00	0.86	0.17	0.57	1.00	1.02	0.80	0.94	1.00
oleoylcarnitine (C18)	Lipid	Fatty Acid Metabolism(Acyl Carnitin	1.13	0.01	0.08	1.00	1.12	0.01	0.17	1.00	1.09	0.05	0.39	1.00	0.99	0.52	0.88	1.00	0.96	0.17	0.58	1.00	0.97	0.66	0.88	1.00
oleoylcholine	Lipid	Fatty Acid Metabolism (Acyl Cholin	0.89	0.13	0.47	1.00	1.11	0.34	0.74	1.00	1.02	0.73	0.95	1.00	1.26	0.00	0.09	0.89	1.15	0.02	0.37	1.00	0.92	0.58	0.86	1.00
oleoyl-linoleoyl-glycerol (18:1/18:2) [1]	Lipid	Diacylglycerol	0.93	0.14	0.48	1.00	0.92	0.27	0.66	1.00	0.96	0.37	0.84	1.00	0.99	0.43	0.84	1.00	1.03	0.94	0.98	1.00	1.04	0.70	0.90	1.00
oleoyl-linoleoyl-glycerol (18:1/18:2) [2]	Lipid	Diacylglycerol	0.95	0.27	0.61	1.00	0.94	0.38	0.75	1.00	0.99	0.43	0.89	1.00	0.99	0.39	0.82	1.00	1.04	0.81	0.94	1.00	1.05	0.87	0.97	1.00
oleoyl-oleoyl-glycerol (18:1/18:1) [1]*	Lipid	Diacylglycerol	0.95	0.33	0.66	1.00	0.91	0.20	0.60	1.00	1.00	0.57	0.94	1.00	0.96	0.23	0.75	1.00	1.05	0.83	0.95	1.00	1.09	0.29	0.73	1.00
oleoyl-oleoyl-glycerol (18:1/18:1) [2]*	Lipid	Diacylglycerol	0.92	0.14	0.49	1.00	0.87	0.05	0.38	1.00	1.00	0.63	0.94	1.00	0.94	0.42	0.84	1.00	1.08	0.61	0.83	1.00	1.14	0.10	0.64	1.00
ornithine	Amino Acid	Urea cycle; Arginine and Proline Met	0.94	0.16	0.52	1.00	0.93	0.11	0.48	1.00	0.99	0.70	0.94	1.00	0.99	0.89	0.97	1.00	1.05	0.16	0.56	1.00	1.06	0.07	0.57	1.00
orotate	Nucleotide	Pyrimidine Metabolism, Orotate cor	0.94	0.36	0.68	1.00	0.94	0.34	0.74	1.00	0.95	0.54	0.93	1.00	0.99	0.76	0.95	1.00	1.00	0.84	0.95	1.00	1.01	0.67	0.89	1.00
orotidine	Nucleotide	Pyrimidine Metabolism, Orotate cor	0.89	0.09	0.42	1.00	0.82	0.02	0.27	1.00	0.92	0.43	0.89	1.00	0.92	0.15	0.66	1.00	1.04	0.27	0.63	1.00	1.12	0.00	0.05	0.55
O-sulfo-L-tyrosine	Xenobiotics	Chemical	1.06	0.21	0.55	1.00	1.08	0.08	0.44	1.00	1.09	0.05	0.39	1.00	1.01	0.66	0.92	1.00	1.02	0.26	0.63	1.00	1.01	0.50	0.82	1.00
oxalate (ethanedioate)	Cofactors anc	Ascorbate and Aldarate Metabolism	0.97	0.53	0.81	1.00	0.93	0.09	0.45	1.00	0.99	0.49	0.92	1.00	0.96	0.14	0.66	1.00	1.02	0.61	0.83	1.00	1.07	0.14	0.64	1.00
palmitate (16:0)	Lipid	Long Chain Fatty Acid	1.07	0.50	0.78	1.00	1.05	0.57	0.85	1.00	1.01	0.89	0.99	1.00	0.98	0.69	0.93	1.00	0.94	0.19	0.58	1.00	0.96	0.38	0.78	1.00
palmitoleate (16:1n7)	Lipid	Long Chain Fatty Acid	1.30	0.08	0.42	1.00	1.21	0.18	0.58	1.00	1.09	0.28	0.79	1.00	0.93	0.45	0.85	1.00	0.84	0.03	0.44	1.00	0.90	0.27	0.72	1.00
palmitoleoylcarnitine (C16:1)*	Lipid	Fatty Acid Metabolism(Acyl Carnitin	1.19	0.01	0.12	1.00	1.19	0.03	0.27	1.00	1.10	0.10	0.51	1.00	0.99	0.81	0.97	1.00	0.92	0.22	0.61	1.00	0.93	0.14	0.65	1.00
palmitoleoyl-linoleoyl-glycerol (16:1/18:2) [1]*	Lipid	Diacylglycerol	0.87	0.17	0.52	1.00	0.86	0.35	0.74	1.00	0.92	0.87	0.98	1.00	0.98	0.62	0.91	1.00	1.06	0.33	0.68	1.00	1.08	0.38	0.78	1.00
palmitoloelycholine	Lipid	Fatty Acid Metabolism (Acyl Cholin	0.84	0.09	0.42	1.00	1.13	0.31	0.71	1.00	1.02	0.94	1.00	1.00	1.35	0.00	0.04	0.15	1.22	0.01	0.27	1.00	0.91	0.67	0.89	1.00
palmitoyl dihydrosphingomyelin (d18:0/16:0)*	Lipid	Dihydrosphingomyelins	0.99	0.94	0.98	1.00	1.00	0.70	0.90	1.00	1.02	0.59	0.94	1.00	1.01	0.94	0.98	1.00	1.02	0.33	0.68	1.00	1.01	0.54	0.84	1.00
palmitoyl ethanolamide	Lipid	Endocannabinoid	1.09	0.21	0.56	1.00	0.94	0.27	0.66	1.00	0.95	0.42	0.89	1.00	0.86	0.00	0.05	0.33	0.87	0.01	0.26	1.00	1.01	0.50	0.82	1.00
palmitoyl sphingomyelin (d18:1/16:0)	Lipid	Sphingomyelins	0.99	0.90	0.96	1.00	1.01	0.39	0.76	1.00	1.00	0.67	0.94	1.00	1.02	0.31	0.81	1.00	1.02	0.45	0.74	1.00	0.99	0.63	0.87	1.00
palmitoylcarnitine (C16)	Lipid	Fatty Acid Metabolism(Acyl Carnitin	1.02	0.81	0.94	1.00	1.05	0.07	0.42	1.00	1.01	0.62	0.94	1.00	1.02	0.63	0.92	1.00	0.99	0.79	0.92	1.00	0.97	0.29	0.73	1.00
palmitoylcholine	Lipid	Fatty Acid Metabolism (Acyl Cholin	0.90	0.12	0.47	1.00	1.14	0.33	0.73	1.00	1.04	0.59	0.94	1.00	1.26	0.00	0.10	1.00	1.16	0.01	0.31	1.00	0.92	0.67	0.89	1.00
palmitoyl-docosahexaenoyl-glycerol (16:0/22:6)	Lipid	Diacylglycerol	1.02	0.43	0.74	1.00	1.09	0.40	0.76	1.00	0.99	0.73	0.95	1.00	1.07	0.62	0.91	1.00	0.97	0.67	0.85	1.00	0.91	0.60	0.86	1.00

palmitoyl-myristoyl-glycerol (16:0/14:0) [1]*	Lipid	Diacylglycerol	0.58	0.00	0.02	0.33	0.64	0.01	0.17	1.00	0.65	0.01	0.20	1.00	1.11	0.25	0.76	1.00	1.12	0.08	0.49	1.00	1.02	0.69	0.90	1.00
palmitoyl-oleoyl-glycerol (16:0/18:1) [1]*	Lipid	Diacylglycerol	0.84	0.01	0.11	1.00	0.84	0.04	0.34	1.00	0.88	0.04	0.35	1.00	0.99	0.83	0.97	1.00	1.04	0.45	0.74	1.00	1.05	0.42	0.78	1.00
palmitoyl-oleoyl-glycerol (16:0/18:1) [2]*	Lipid	Diacylglycerol	0.85	0.01	0.14	1.00	0.84	0.02	0.25	1.00	0.88	0.05	0.39	1.00	0.99	0.97	0.99	1.00	1.04	0.44	0.74	1.00	1.06	0.39	0.78	1.00
palmitoyl-palmitoyl-glycerol (16:0/16:0) [2]*	Lipid	Diacylglycerol	0.77	0.02	0.16	1.00	0.81	0.08	0.44	1.00	0.80	0.05	0.37	1.00	1.05	0.33	0.81	1.00	1.04	0.54	0.80	1.00	0.99	0.68	0.89	1.00
pantothenate (Vitamin B5)	Cofactors and	Pantothenate and CoA Metabolism	0.89	0.76	0.92	1.00	0.89	0.95	1.00	1.00	0.94	0.99	1.00	1.00	0.99	0.75	0.95	1.00	1.05	0.33	0.68	1.00	1.06	0.75	0.92	1.00
p-cresol sulfate	Xenobiotics	Benzoate Metabolism	1.04	0.60	0.84	1.00	1.10	0.25	0.65	1.00	0.99	1.00	1.00	1.00	1.06	0.34	0.81	1.00	0.95	0.81	0.94	1.00	0.90	0.18	0.68	1.00
pelargonate (9:0)	Lipid	Medium Chain Fatty Acid	1.04	0.39	0.71	1.00	0.96	0.07	0.42	1.00	0.92	0.09	0.49	1.00	0.93	0.62	0.91	1.00	0.89	0.90	0.97	1.00	0.96	0.72	0.91	1.00
pentadecanoate (15:0)	Lipid	Long Chain Fatty Acid	1.09	0.49	0.77	1.00	1.06	0.53	0.83	1.00	0.98	0.98	1.00	1.00	0.98	1.00	1.00	1.00	0.90	0.22	0.61	1.00	0.92	0.54	0.84	1.00
perfluorooctanesulfonate (PFOS)	Xenobiotics	Chemical	0.95	0.17	0.52	1.00	0.93	0.03	0.28	1.00	0.93	0.03	0.35	1.00	0.97	0.60	0.91	1.00	0.98	0.88	0.96	1.00	1.01	0.87	0.97	1.00
perfluorooctanoate (PFOA)*	Xenobiotics	Chemical	0.96	0.11	0.47	1.00	0.94	0.01	0.16	1.00	0.92	0.00	0.03	0.41	0.98	0.33	0.81	1.00	0.96	0.07	0.48	1.00	0.98	0.57	0.86	1.00
phenol sulfate	Amino Acid	Tyrosine Metabolism	0.90	0.40	0.72	1.00	0.79	0.20	0.60	1.00	1.01	0.64	0.94	1.00	0.87	0.33	0.81	1.00	1.11	0.98	0.99	1.00	1.27	0.15	0.66	1.00
phenylacetylglutamine	Peptide	Acetylated Peptides	1.01	0.84	0.95	1.00	1.02	0.64	0.87	1.00	1.01	0.79	0.96	1.00	1.01	0.87	0.97	1.00	1.00	0.90	0.97	1.00	0.99	0.48	0.82	1.00
phenylalanine	Amino Acid	Phenylalanine Metabolism	0.93	0.33	0.66	1.00	0.95	0.51	0.82	1.00	0.99	0.85	0.98	1.00	1.03	0.39	0.83	1.00	1.06	0.08	0.49	1.00	1.03	0.22	0.68	1.00
phenyllactate (PLA)	Amino Acid	Phenylalanine Metabolism	0.78	0.01	0.08	1.00	0.93	0.11	0.47	1.00	0.88	0.10	0.51	1.00	1.19	0.02	0.37	1.00	1.13	0.01	0.26	1.00	0.95	0.13	0.64	1.00
phenylpyruvate	Amino Acid	Phenylalanine Metabolism	0.87	0.11	0.47	1.00	0.91	0.22	0.61	1.00	0.97	0.99	1.00	1.00	1.05	0.61	0.91	1.00	1.12	0.21	0.60	1.00	1.07	0.10	0.64	1.00
phosphoethanolamine (PE)	Lipid	Phospholipid Metabolism	0.93	0.10	0.44	1.00	0.93	0.17	0.57	1.00	0.97	0.74	0.95	1.00	1.01	0.94	0.98	1.00	1.05	0.22	0.61	1.00	1.04	0.35	0.78	1.00
phytanate	Xenobiotics	Food Component/Plant	0.94	0.78	0.93	1.00	1.01	0.81	0.95	1.00	0.92	0.40	0.88	1.00	1.07	0.54	0.89	1.00	0.97	0.53	0.79	1.00	0.91	0.33	0.75	1.00
pipecolate	Amino Acid	Lysine Metabolism	0.99	0.33	0.66	1.00	0.87	0.83	0.95	1.00	1.28	0.20	0.69	1.00	0.88	0.39	0.82	1.00	1.28	0.06	0.47	1.00	1.46	0.33	0.75	1.00
pregnen-diol disulfate*	Lipid	Pregnenolone Steroids	1.02	0.63	0.86	1.00	0.90	0.10	0.46	1.00	1.03	0.59	0.94	1.00	0.88	0.05	0.54	1.00	1.01	0.99	0.99	1.00	1.14	0.02	0.44	1.00
pregnenediol sulfate (C21H34O5S)*	Lipid	Pregnenolone Steroids	1.00	0.95	0.98	1.00	1.01	0.60	0.86	1.00	1.07	0.12	0.56	1.00	1.00	0.94	0.98	1.00	1.07	0.09	0.52	1.00	1.07	0.20	0.68	1.00
pregnenetriol disulfate*	Lipid	Pregnenolone Steroids	0.95	0.84	0.95	1.00	0.89	0.21	0.61	1.00	0.96	0.72	0.95	1.00	0.93	0.27	0.78	1.00	1.00	0.85	0.95	1.00	1.08	0.19	0.68	1.00
pregnenetriol sulfate*	Lipid	Pregnenolone Steroids	0.96	0.45	0.76	1.00	0.94	0.52	0.82	1.00	0.99	0.59	0.94	1.00	0.98	0.67	0.92	1.00	1.03	0.67	0.85	1.00	1.05	0.24	0.71	1.00
prolylhydroxyproline	Amino Acid	Urea cycle; Arginine and Proline Met	1.13	0.03	0.24	1.00	1.07	0.23	0.61	1.00	1.07	0.33	0.82	1.00	0.95	0.09	0.62	1.00	0.94	0.10	0.53	1.00	1.00	0.98	1.00	1.00
proline	Amino Acid	Urea cycle; Arginine and Proline Met	0.90	0.11	0.47	1.00	0.91	0.04	0.32	1.00	0.94	0.27	0.78	1.00	1.01	0.81	0.97	1.00	1.04	0.27	0.63	1.00	1.03	0.20	0.68	1.00
propionylcamitine (C3)	Lipid	Fatty Acid Metabolism (also BCAA M	0.86	0.04	0.28	1.00	0.91	0.18	0.58	1.00	0.91	0.08	0.45	1.00	1.05	0.08	0.62	1.00	1.06	0.18	0.58	1.00	1.00	0.88	0.97	1.00
propionylglycine (C3)	Lipid	Fatty Acid Metabolism (also BCAA M	0.88	0.09	0.43	1.00	0.95	0.89	0.98	1.00	0.90	0.12	0.56	1.00	1.08	0.18	0.71	1.00	1.03	0.51	0.78	1.00	0.95	0.35	0.78	1.00
propyl 4-hydroxybenzoate sulfate	Xenobiotics	Benzoate Metabolism	0.96	0.51	0.79	1.00	0.90	0.12	0.48	1.00	0.91	0.16	0.63	1.00	0.94	0.54	0.89	1.00	0.95	0.42	0.73	1.00	1.01	0.83	0.95	1.00
pseudouridine	Nucleotide	Pyrimidine Metabolism, Uracil cont	1.03	0.47	0.77	1.00	1.02	0.46	0.80	1.00	1.05	0.16	0.63	1.00	0.99	0.35	0.81	1.00	1.02	0.66	0.85	1.00	1.03	0.37	0.78	1.00
pyridoxate	Cofactors and	Vitamin B6 Metabolism	0.40	0.04	0.28	1.00	0.41	0.12	0.48	1.00	0.72	0.33	0.83	1.00	1.04	0.33	0.81	1.00	1.83	0.01	0.29	1.00	1.76	0.01	0.44	1.00
pyroglutamine*	Amino Acid	Glutamate Metabolism	1.11	0.24	0.59	1.00	1.06	0.74	0.91	1.00	1.17	0.19	0.67	1.00	0.95	0.27	0.79	1.00	1.06	0.62	0.83	1.00	1.11	0.04	0.48	1.00
pyruvate	Carbohydrate	Glycolysis, Gluconeogenesis, and Py	1.05	0.17	0.52	1.00	1.07	0.33	0.73	1.00	0.99	0.70	0.94	1.00	1.01	1.00	1.00	1.00	0.94	0.84	0.95	1.00	0.93	0.54	0.84	1.00
quinate	Xenobiotics	Food Component/Plant	0.77	0.00	0.07	1.00	0.75	0.00	0.09	1.00	0.88	0.17	0.65	1.00	0.98	0.35	0.81	1.00	1.14	0.10	0.53	1.00	1.16	0.13	0.64	1.00
quinolinate	Cofactors and	Nicotinate and Nicotinamide Metab	0.93	0.23	0.58	1.00	0.87	0.15	0.54	1.00	0.96	0.28	0.79	1.00	0.94	0.55	0.90	1.00	1.03	0.29	0.65	1.00	1.10	0.48	0.82	1.00
retinol (Vitamin A)	Cofactors and	Vitamin A Metabolism	0.91	0.04	0.29	1.00	0.95	0.37	0.74	1.00	0.98	0.53	0.93	1.00	1.04	0.09	0.62	1.00	1.08	0.02	0.38	1.00	1.03	0.23	0.70	1.00
ribitol	Carbohydrate	Pentose Metabolism	0.99	0.75	0.92	1.00	0.97	0.42	0.78	1.00	1.03	0.39	0.86	1.00	0.98	0.60	0.91	1.00	1.04	0.29	0.64	1.00	1.06	0.17	0.66	1.00
ribonate (ribonolactone)	Carbohydrate	Pentose Metabolism	0.89	0.06	0.36	1.00	0.90	0.14	0.52	1.00	0.89	0.05	0.39	1.00	1.02	0.74	0.95	1.00	1.00	0.90	0.97	1.00	0.98	0.87	0.97	1.00
salicylate	Xenobiotics	Drug - Topical Agents	1.24	0.85	0.95	1.00	1.60	0.11	0.46	1.00	1.10	0.72	0.95	1.00	1.29	0.20	0.72	1.00	0.89	0.62	0.83	1.00	0.69	0.06	0.53	1.00
sebacate (C10-DC)	Lipid	Fatty Acid, Dicarboxylate	0.99	0.99	1.00	1.00	0.94	0.36	0.74	1.00	1.05	0.48	0.92	1.00	0.95	0.78	0.95	1.00	1.07	0.25	0.63	1.00	1.12	0.13	0.64	1.00
serine	Amino Acid	Glycine, Serine and Threonine Metab	0.99	0.88	0.96	1.00	0.97	0.33	0.73	1.00	0.98	0.52	0.93	1.00	0.98	0.55	0.90	1.00	1.00	0.80	0.93	1.00	1.01	0.61	0.87	1.00
serotonin	Amino Acid	Tryptophan Metabolism	1.00	0.79	0.93	1.00	0.97	0.52	0.82	1.00	0.99	0.60	0.94	1.00	0.97	0.40	0.83	1.00	0.98	0.94	0.98	1.00	1.02	0.60	0.86	1.00
S-methylcysteine	Amino Acid	Methionine, Cysteine, SAM and Tau	1.09	0.24	0.59	1.00	1.17	0.37	0.74	1.00	1.25	0.19	0.67	1.00	1.07	0.83	0.97	1.00	1.15	0.42	0.73	1.00	1.07	0.66	0.88	1.00
S-methylcysteine sulfoxide	Amino Acid	Methionine, Cysteine, SAM and Tau	1.13	0.46	0.76	1.00	1.32	0.09	0.45	1.00	1.27	0.12	0.56	1.00	1.17	0.80	0.97	1.00	1.12	0.60	0.82	1.00	0.96	0.98	1.00	1.00
sphinganine-1-phosphate	Lipid	Sphingolipid Synthesis	1.01	0.40	0.72	1.00	1.05	0.14	0.53	1.00	1.05	0.22	0.72	1.00	1.04	0.38	0.82	1.00	1.04	0.50	0.77	1.00	1.00	0.84	0.95	1.00
sphingomyelin (d17:1/14:0, d16:1/15:0)*	Lipid	Sphingomyelins	0.90	0.14	0.48	1.00	1.04	0.24	0.64	1.00	0.99	0.85	0.98	1.00	1.16	0.00	0.04	0.09	1.10	0.12	0.54	1.00	0.95	0.08	0.60	1.00
sphingomyelin (d17:1/16:0, d18:1/15:0, d16:1	Lipid	Sphingomyelins	0.97	0.41	0.73	1.00	1.01	0.52	0.82	1.00	0.99	0.80	0.96	1.00	1.05	0.12	0.66	1.00	1.03	0.46	0.75	1.00	0.98	0.59	0.86	1.00
sphingomyelin (d17:2/16:0, d18:2/15:0)*	Lipid	Sphingomyelins	0.92	0.21	0.55	1.00	1.03	0.43	0.78	1.00	0.97	0.63	0.94	1.00	1.12	0.00	0.10	1.00	1.05	0.34	0.69	1.00	0.94	0.05	0.52	1.00
sphingomyelin (d18:0/18:0, d19:0/17:0)*	Lipid	Dihydrosphingomyelins	0.96	0.73	0.91	1.00	0.95	0.60	0.86	1.00	0.93	0.08	0.44	1.00	0.99	0.93	0.98	1.00	0.97	0.53	0.79	1.00	0.98	0.47	0.82	1.00
sphingomyelin (d18:0/20:0, d16:0/22:0)*	Lipid	Dihydrosphingomyelins	0.93	0.51	0.79	1.00	0.99	0.97	1.00	1.00	0.97	0.61	0.94	1.00	1.07	0.49	0.88	1.00	1.05	0.49	0.77	1.00	0.98	0.59	0.86	1.00
sphingomyelin (d18:1/14:0, d16:1/16:0)*	Lipid	Sphingomyelins	0.95	0.25	0.60	1.00	1.02	0.37	0.74	1.00	0.98	0.54	0.93	1.00	1.07	0.01	0.23	1.00	1.03	0.30	0.65	1.00	0.96	0.30	0.74	1.00
sphingomyelin (d18:1/17:0, d17:1/18:0, d19:1	Lipid	Sphingomyelins	0.98	0.94	0.98	1.00	1.00	0.83	0.95	1.00	0.96	0.24	0.74	1.00	1.02	0.42	0.84	1.00	0.99	0.66	0.85	1.00	0.97	0.30	0.74	1.00
sphingomyelin (d18:1/18:1, d18:2/18:0)	Lipid	Sphingomyelins	0.99	0.70	0.90	1.00	1.00	0.64	0.87	1.00	0.98	0.69	0.94	1.00	1.01	0.70	0.93	1.00	0.99	0.68	0.86	1.00	0.98	0.28	0.72	1.00
sphingomyelin (d18:1/19:0, d19:1/18:0)*	Lipid	Sphingomyelins	0.94	0.21	0.55	1.00	1.00	0.63	0.87	1.00	0.95	0.14	0.60	1.00	1.06	0.06	0.57	1.00	1.01	0.97	0.99	1.00	0.95	0.13	0.64	1.00
sphingomyelin (d18:1/20:0, d16:1/22:0)*	Lipid	Sphingomyelins	0.98	0.85	0.95	1.00	0.99	0.84	0.96	1.00	0.98	0.45	0.90	1.00	1.01	0.89	0.97	1.00	1.00	0.63	0.84	1.00	1.00	0.97	0.99	1.00
sphingomyelin (d18:1/20:1, d18:2/20:0)*	Lipid	Sphingomyelins	1.00	0.73	0.91	1.00	1.00	0.36	0.74	1.00	0.99	0.84	0.98	1.00	1.01	0.66	0.92	1.00	1.00	0.88	0.96	1.00	0.99	0.50	0.82	1.00

sphingomyelin (d18:1/20:2, d18:2/20:1, d16:1	Lipid	Sphingomyelins	0.98	0.58	0.83	1.00	1.03	0.61	0.86	1.00	1.02	0.46	0.92	1.00	1.05	0.16	0.66	1.00	1.04	0.28	0.63	1.00	1.00	0.63	0.87	1.00
sphingomyelin (d18:1/21:0, d17:1/22:0, d16:1	Lipid	Sphingomyelins	0.94	0.29	0.63	1.00	1.03	0.29	0.69	1.00	0.98	0.75	0.95	1.00	1.09	0.04	0.52	1.00	1.04	0.34	0.69	1.00	0.95	0.12	0.64	1.00
sphingomyelin (d18:1/22:1, d18:2/22:0, d16:1	Lipid	Sphingomyelins	0.97	0.57	0.82	1.00	1.00	0.46	0.80	1.00	0.99	0.78	0.96	1.00	1.04	0.13	0.66	1.00	1.02	0.23	0.63	1.00	0.98	0.40	0.78	1.00
sphingomyelin (d18:1/22:2, d18:2/22:1, d16:1	Lipid	Sphingomyelins	0.98	0.92	0.97	1.00	1.01	0.23	0.61	1.00	1.00	0.45	0.90	1.00	1.04	0.11	0.63	1.00	1.02	0.29	0.65	1.00	0.98	0.54	0.84	1.00
sphingomyelin (d18:1/24:1, d18:2/24:0)*	Lipid	Sphingomyelins	0.97	0.51	0.79	1.00	0.97	0.39	0.76	1.00	0.97	0.35	0.83	1.00	1.00	0.98	0.99	1.00	1.01	0.62	0.83	1.00	1.01	0.97	0.99	1.00
sphingomyelin (d18:2/14:0, d18:1/14:1)*	Lipid	Sphingomyelins	0.92	0.39	0.71	1.00	1.06	0.20	0.60	1.00	1.00	0.89	0.99	1.00	1.15	0.00	0.06	0.51	1.08	0.21	0.61	1.00	0.94	0.04	0.48	1.00
sphingomyelin (d18:2/16:0, d18:1/16:1)*	Lipid	Sphingomyelins	0.98	0.84	0.95	1.00	1.01	0.64	0.87	1.00	0.99	0.49	0.92	1.00	1.03	0.28	0.79	1.00	1.00	0.95	0.99	1.00	0.98	0.41	0.78	1.00
sphingomyelin (d18:2/18:1)*	Lipid	Sphingomyelins	0.98	0.87	0.96	1.00	1.01	0.59	0.86	1.00	0.97	0.74	0.95	1.00	1.03	0.41	0.83	1.00	0.99	0.63	0.84	1.00	0.96	0.37	0.78	1.00
sphingomyelin (d18:2/21:0, d16:2/23:0)*	Lipid	Sphingomyelins	0.94	0.23	0.57	1.00	1.03	0.17	0.57	1.00	0.97	0.46	0.92	1.00	1.09	0.00	0.11	1.00	1.03	0.46	0.75	1.00	0.95	0.06	0.57	1.00
sphingomyelin (d18:2/23:0, d18:1/23:1, d17:1	Lipid	Sphingomyelins	0.96	0.35	0.67	1.00	1.06	0.04	0.34	1.00	0.97	0.57	0.94	1.00	1.11	0.01	0.23	1.00	1.01	0.62	0.83	1.00	0.92	0.04	0.48	1.00
sphingomyelin (d18:2/23:1)*	Lipid	Sphingomyelins	0.95	0.23	0.58	1.00	1.02	0.11	0.47	1.00	0.98	0.67	0.94	1.00	1.08	0.00	0.14	1.00	1.03	0.39	0.72	1.00	0.96	0.16	0.66	1.00
sphingomyelin (d18:2/24:1, d18:1/24:2)*	Lipid	Sphingomyelins	0.98	0.73	0.91	1.00	1.00	0.39	0.76	1.00	0.98	0.85	0.98	1.00	1.02	0.20	0.72	1.00	1.01	0.69	0.87	1.00	0.98	0.29	0.74	1.00
sphingomyelin (d18:2/24:2)*	Lipid	Sphingomyelins	0.97	0.61	0.85	1.00	1.01	0.39	0.76	1.00	0.99	0.59	0.94	1.00	1.04	0.14	0.66	1.00	1.03	0.38	0.70	1.00	0.99	0.60	0.86	1.00
sphingosine 1-phosphate	Lipid	Sphingosines	1.00	0.72	0.91	1.00	1.03	0.14	0.52	1.00	1.05	0.10	0.51	1.00	1.04	0.20	0.72	1.00	1.06	0.11	0.53	1.00	1.02	0.81	0.94	1.00
stearate (18:0)	Lipid	Long Chain Fatty Acid	1.06	0.36	0.68	1.00	1.05	0.31	0.71	1.00	1.04	0.48	0.92	1.00	0.99	0.93	0.98	1.00	0.98	0.89	0.96	1.00	0.99	0.84	0.95	1.00
stearidonate (18:4n3)	Lipid	Polyunsaturated Fatty Acid (n3 and	1.02	0.57	0.82	1.00	1.07	0.21	0.61	1.00	1.00	0.67	0.94	1.00	1.05	0.28	0.79	1.00	0.98	0.51	0.78	1.00	0.94	0.14	0.64	1.00
stearoyl sphingomyelin (d18:1/18:0)	Lipid	Sphingomyelins	0.98	0.93	0.97	1.00	0.96	0.40	0.76	1.00	0.95	0.14	0.60	1.00	0.98	0.57	0.90	1.00	0.97	0.41	0.72	1.00	0.99	0.63	0.87	1.00
stearoyl-arachidonoyl-glycerol (18:0/20:4) [1]*	Lipid	Diacylglycerol	1.06	0.76	0.92	1.00	1.11	0.15	0.55	1.00	1.04	0.37	0.84	1.00	1.05	0.46	0.87	1.00	0.98	0.94	0.98	1.00	0.94	0.75	0.92	1.00
stearoylcarnitine (C18)	Lipid	Fatty Acid Metabolism(Acyl Carnitin	0.98	0.87	0.96	1.00	1.01	0.70	0.90	1.00	0.99	0.93	1.00	1.00	1.03	0.72	0.94	1.00	1.01	0.58	0.81	1.00	0.98	1.00	1.00	1.00
succinate	Energy	TCA Cycle	1.02	0.67	0.88	1.00	1.00	0.92	0.98	1.00	1.08	0.27	0.79	1.00	0.98	0.31	0.81	1.00	1.05	0.52	0.79	1.00	1.08	0.08	0.61	1.00
succinimide	Xenobiotics	Chemical	1.04	0.29	0.63	1.00	1.00	0.73	0.90	1.00	0.98	0.92	1.00	1.00	0.97	0.06	0.57	1.00	0.95	0.20	0.59	1.00	0.98	0.22	0.68	1.00
sulfate*	Xenobiotics	Chemical	0.94	0.12	0.47	1.00	0.93	0.01	0.19	1.00	0.95	0.06	0.40	1.00	0.99	0.73	0.94	1.00	1.02	0.45	0.74	1.00	1.03	0.36	0.78	1.00
tartronate (hydroxymalonate)	Xenobiotics	Food Component/Plant	1.04	0.32	0.65	1.00	0.94	0.32	0.72	1.00	1.04	0.48	0.92	1.00	0.90	0.00	0.18	1.00	0.99	0.81	0.94	1.00	1.10	0.06	0.56	1.00
taurine	Amino Acid	Methionine, Cysteine, SAM and Tau	0.95	0.30	0.63	1.00	0.95	0.12	0.49	1.00	0.99	0.62	0.94	1.00	0.99	0.93	0.98	1.00	1.04	0.43	0.73	1.00	1.04	0.25	0.71	1.00
taurochenodeoxycholate	Lipid	Primary Bile Acid Metabolism	0.68	0.14	0.48	1.00	1.06	0.72	0.90	1.00	0.74	0.31	0.81	1.00	1.56	0.03	0.44	1.00	1.09	0.12	0.54	1.00	0.70	0.06	0.56	1.00
taurocholate	Lipid	Primary Bile Acid Metabolism	0.88	0.23	0.57	1.00	1.08	0.87	0.97	1.00	0.79	0.10	0.50	1.00	1.22	0.17	0.69	1.00	0.90	0.49	0.77	1.00	0.74	0.03	0.48	1.00
taurocholenate sulfate*	Lipid	Secondary Bile Acid Metabolism	0.81	0.05	0.30	1.00	0.82	0.08	0.45	1.00	0.75	0.02	0.30	1.00	1.01	0.73	0.94	1.00	0.92	0.11	0.53	1.00	0.92	0.36	0.78	1.00
tetradecadienoate (14:2)*	Lipid	Polyunsaturated Fatty Acid (n3 and	1.34	0.00	0.04	1.00	1.29	0.01	0.17	1.00	1.19	0.05	0.37	1.00	0.97	0.48	0.88	1.00	0.89	0.15	0.55	1.00	0.92	0.21	0.68	1.00
tetradecanedioate (C14)	Lipid	Fatty Acid, Dicarboxylate	0.96	0.27	0.61	1.00	0.94	0.21	0.61	1.00	0.83	0.03	0.32	1.00	0.98	0.21	0.73	1.00	0.86	0.15	0.55	1.00	0.88	0.43	0.78	1.00
theobromine	Xenobiotics	Xanthine Metabolism	0.74	0.06	0.36	1.00	0.83	0.21	0.61	1.00	1.03	0.92	1.00	1.00	1.12	0.30	0.81	1.00	1.40	0.05	0.45	1.00	1.25	0.15	0.66	1.00
theophylline	Xenobiotics	Xanthine Metabolism	0.85	0.03	0.24	1.00	0.92	0.16	0.56	1.00	1.00	0.85	0.98	1.00	1.07	0.41	0.83	1.00	1.17	0.13	0.54	1.00	1.09	0.26	0.71	1.00
thioproline	Xenobiotics	Chemical	1.04	0.57	0.82	1.00	1.04	0.83	0.95	1.00	1.11	0.14	0.58	1.00	1.00	0.99	1.00	1.00	1.06	0.16	0.56	1.00	1.06	0.04	0.48	1.00
threonate	Cofactors and	Ascorbate and Aldarate Metabolism	0.97	0.42	0.73	1.00	0.92	0.04	0.35	1.00	0.97	0.41	0.89	1.00	0.95	0.20	0.72	1.00	1.00	0.93	0.98	1.00	1.06	0.17	0.66	1.00
threonine	Amino Acid	Glycine, Serine and Threonine Metab	0.95	0.55	0.82	1.00	0.96	0.27	0.66	1.00	1.01	0.54	0.93	1.00	1.01	0.68	0.93	1.00	1.06	0.35	0.69	1.00	1.05	0.23	0.69	1.00
thyroxine	Amino Acid	Tyrosine Metabolism	0.96	0.24	0.59	1.00	0.96	0.09	0.45	1.00	0.99	0.57	0.94	1.00	1.00	0.87	0.97	1.00	1.02	0.36	0.70	1.00	1.03	0.33	0.75	1.00
hydroxyproline	Amino Acid	Urea cycle; Arginine and Proline Met	1.01	0.57	0.82	1.00	0.98	0.92	0.98	1.00	1.06	0.59	0.94	1.00	0.97	0.60	0.91	1.00	1.06	0.43	0.73	1.00	1.09	0.42	0.78	1.00
tricosanoyl sphingomyelin (d18:1/23:0)*	Lipid	Sphingomyelins	0.93	0.10	0.46	1.00	0.94	0.48	0.81	1.00	0.95	0.54	0.93	1.00	1.01	0.75	0.95	1.00	1.02	0.54	0.80	1.00	1.01	0.76	0.93	1.00
tridecenedioate (C13:1-DC)*	Lipid	Fatty Acid, Dicarboxylate	1.07	0.61	0.85	1.00	1.04	0.75	0.92	1.00	0.87	0.04	0.37	1.00	0.98	0.33	0.81	1.00	0.82	0.02	0.42	1.00	0.84	0.13	0.64	1.00
triethanolamine	Xenobiotics	Chemical	0.92	0.12	0.47	1.00	0.87	0.00	0.03	0.51	1.00	0.04	0.37	1.00	0.95	0.07	0.59	1.00	1.09	0.46	0.75	1.00	1.15	0.04	0.48	1.00
trigonelline (N'-methylnicotinate)	Cofactors and	Nicotinate and Nicotinamide Metab	0.69	0.00	0.06	1.00	0.68	0.00	0.09	1.00	0.77	0.03	0.35	1.00	0.99	0.99	1.00	1.00	1.11	0.68	0.86	1.00	1.12	0.53	0.83	1.00
trimethylamine N-oxide	Lipid	Phospholipid Metabolism	0.90	0.39	0.71	1.00	0.95	0.89	0.98	1.00	1.00	0.75	0.95	1.00	1.06	0.23	0.76	1.00	1.12	0.27	0.63	1.00	1.06	0.58	0.86	1.00
tryptophan	Amino Acid	Tryptophan Metabolism	0.91	0.15	0.50	1.00	0.92	0.12	0.49	1.00	0.98	0.68	0.94	1.00	1.01	0.67	0.92	1.00	1.08	0.05	0.45	1.00	1.07	0.05	0.52	1.00
tyrosine	Amino Acid	Tyrosine Metabolism	0.88	0.08	0.39	1.00	0.92	0.07	0.42	1.00	0.93	0.20	0.68	1.00	1.04	0.25	0.76	1.00	1.06	0.10	0.53	1.00	1.02	0.38	0.78	1.00
urate	Nucleotide	Purine Metabolism, (Hypo)Xanthine	1.01	0.90	0.96	1.00	1.01	0.30	0.70	1.00	1.04	0.27	0.79	1.00	1.00	0.66	0.92	1.00	1.04	0.14	0.55	1.00	1.03	0.22	0.68	1.00
urea	Amino Acid	Urea cycle; Arginine and Proline Met	0.92	0.05	0.31	1.00	0.96	0.39	0.76	1.00	0.98	0.68	0.94	1.00	1.05	0.29	0.80	1.00	1.07	0.05	0.45	1.00	1.02	0.41	0.78	1.00
uridine	Nucleotide	Pyrimidine Metabolism, Uracil cont	1.06	0.14	0.48	1.00	1.04	0.42	0.78	1.00	1.10	0.01	0.19	1.00	0.99	0.73	0.94	1.00	1.04	0.38	0.70	1.00	1.06	0.14	0.65	1.00
valine	Amino Acid	Leucine, Isoleucine and Valine Meta	0.92	0.11	0.47	1.00	0.95	0.34	0.74	1.00	0.97	0.26	0.77	1.00	1.04	0.28	0.79	1.00	1.05	0.20	0.59	1.00	1.01	0.64	0.88	1.00
vanillylmandelate (VMA)	Amino Acid	Tyrosine Metabolism	1.03	0.63	0.86	1.00	1.01	0.60	0.86	1.00	1.02	0.72	0.95	1.00	0.99	0.43	0.84	1.00	1.00	0.38	0.70	1.00	1.01	1.00	1.00	1.00
xanthine	Nucleotide	Purine Metabolism, (Hypo)Xanthine	1.02	0.63	0.86	1.00	0.98	0.95	1.00	1.00	1.07	0.15	0.62	1.00	0.96	0.43	0.84	1.00	1.04	0.34	0.69	1.00	1.08	0.11	0.64	1.00
ximenoylcarnitine (C26:1)*	Lipid	Fatty Acid Metabolism(Acyl Carnitin	0.95	0.27	0.61	1.00	0.97	0.37	0.74	1.00	0.99	0.73	0.95	1.00	1.02	0.51	0.88	1.00	1.04	0.47	0.75	1.00	1.02	0.74	0.91	1.00
X - 09789	Unknown	Unknown	1.00	0.81	0.94	1.00	0.84	0.10	0.46	1.00	0.90	0.40	0.88	1.00	0.83	0.16	0.66	1.00	0.90	0.37	0.70	1.00	1.08	0.37	0.78	1.00
X - 11261	Unknown	Unknown	0.97	0.54	0.82	1.00	0.96	0.81	0.95	1.00	1.02	0.81	0.97	1.00	0.99	0.81	0.97	1.00	1.04	0.93	0.98	1.00	1.06	0.64	0.88	1.00
X - 11308	Unknown	Unknown	0.96	0.38	0.70	1.00	0.94	0.16	0.56	1.00	0.99	0.74	0.95	1.00	0.98	0.64	0.92	1.00	1.04	0.34	0.69	1.00	1.05	0.11	0.64	1.00
X - 11315	Unknown	Unknown	0.99	0.68	0.89	1.00	0.99	0.93	0.99	1.00	1.00	0.70	0.94	1.00	1.00	0.97	0.99	1.00	1.01	0.72	0.88	1.00	1.01	0.92	0.98	1.00

X - 11372	Unknown	Unknown	0.93	0.23	0.57	1.00	0.94	0.27	0.67	1.00	1.03	0.19	0.67	1.00	1.01	0.93	0.98	1.00	1.11	0.01	0.30	1.00	1.10	0.03	0.47	1.00
X - 11444	Unknown	Unknown	1.03	0.63	0.86	1.00	1.02	0.55	0.84	1.00	1.04	0.23	0.73	1.00	0.99	0.87	0.97	1.00	1.01	0.87	0.96	1.00	1.02	0.52	0.83	1.00
X - 11470	Unknown	Unknown	1.04	0.29	0.63	1.00	1.06	0.27	0.66	1.00	1.14	0.03	0.35	1.00	1.02	0.57	0.90	1.00	1.09	0.05	0.45	1.00	1.07	0.05	0.52	1.00
X - 11530	Unknown	Unknown	1.02	0.30	0.63	1.00	0.92	0.74	0.91	1.00	1.01	0.78	0.96	1.00	0.90	0.23	0.75	1.00	0.99	0.62	0.83	1.00	1.10	0.20	0.68	1.00
X - 11787	Unknown	Unknown	0.98	0.68	0.89	1.00	0.97	0.60	0.86	1.00	1.00	0.83	0.98	1.00	0.99	0.34	0.81	1.00	1.02	0.53	0.79	1.00	1.03	0.33	0.75	1.00
X - 11795	Unknown	Unknown	1.10	0.74	0.92	1.00	0.97	0.92	0.98	1.00	0.87	0.74	0.95	1.00	0.88	0.92	0.98	1.00	0.79	0.84	0.95	1.00	0.90	0.93	0.98	1.00
X - 12026	Unknown	Unknown	1.05	0.58	0.83	1.00	1.01	0.52	0.82	1.00	1.09	0.06	0.39	1.00	0.96	0.36	0.81	1.00	1.04	0.39	0.71	1.00	1.08	0.06	0.54	1.00
X - 12063	Unknown	Unknown	1.01	0.98	0.99	1.00	0.98	0.88	0.97	1.00	0.99	0.80	0.96	1.00	0.96	0.70	0.93	1.00	0.98	0.51	0.78	1.00	1.01	0.26	0.71	1.00
X - 12100	Unknown	Unknown	0.97	0.55	0.82	1.00	0.94	0.38	0.75	1.00	1.00	0.93	1.00	1.00	0.97	0.24	0.76	1.00	1.04	0.18	0.58	1.00	1.07	0.04	0.50	1.00
X - 12104	Unknown	Unknown	1.12	0.02	0.18	1.00	1.07	0.17	0.57	1.00	1.15	0.00	0.06	1.00	0.95	0.19	0.72	1.00	1.02	0.40	0.72	1.00	1.08	0.03	0.47	1.00
X - 12206	Unknown	Unknown	1.02	0.84	0.95	1.00	1.01	0.84	0.96	1.00	1.05	0.14	0.60	1.00	0.98	0.44	0.85	1.00	1.03	0.39	0.72	1.00	1.04	0.14	0.64	1.00
X - 12216	Unknown	Unknown	1.11	0.46	0.76	1.00	1.09	0.51	0.82	1.00	1.10	0.41	0.89	1.00	0.99	0.79	0.96	1.00	0.99	0.84	0.95	1.00	1.01	0.99	1.00	1.00
X - 12230	Unknown	Unknown	0.40	0.00	0.01	0.12	0.52	0.00	0.10	1.00	0.47	0.00	0.02	0.12	1.31	0.27	0.78	1.00	1.17	0.26	0.63	1.00	0.90	0.64	0.88	1.00
X - 12411	Unknown	Unknown	0.88	0.04	0.28	1.00	0.90	0.10	0.46	1.00	1.12	0.94	1.00	1.00	1.02	0.66	0.92	1.00	1.27	0.04	0.45	1.00	1.25	0.11	0.64	1.00
X - 12462	Unknown	Unknown	0.94	0.33	0.66	1.00	0.90	0.11	0.46	1.00	0.91	0.12	0.56	1.00	0.96	0.46	0.87	1.00	0.97	0.47	0.75	1.00	1.01	0.55	0.85	1.00
X - 12472	Unknown	Unknown	1.15	0.75	0.92	1.00	0.87	0.23	0.62	1.00	0.87	0.14	0.60	1.00	0.76	0.06	0.57	1.00	0.76	0.26	0.63	1.00	1.00	0.90	0.97	1.00
X - 12524	Unknown	Unknown	0.95	0.44	0.75	1.00	0.96	0.60	0.86	1.00	0.96	0.68	0.94	1.00	1.01	0.61	0.91	1.00	1.02	0.58	0.81	1.00	1.00	0.87	0.97	1.00
X - 12544	Unknown	Unknown	1.15	0.04	0.28	1.00	1.03	0.51	0.82	1.00	0.70	0.05	0.39	1.00	0.90	0.23	0.76	1.00	0.61	0.16	0.56	1.00	0.68	0.73	0.91	1.00
X - 12844	Unknown	Unknown	1.02	0.76	0.92	1.00	0.99	0.80	0.94	1.00	1.01	0.80	0.96	1.00	0.97	0.44	0.85	1.00	0.99	0.27	0.63	1.00	1.02	0.67	0.89	1.00
X - 12846	Unknown	Unknown	1.01	0.67	0.88	1.00	1.04	0.37	0.74	1.00	1.11	0.14	0.58	1.00	1.03	0.70	0.93	1.00	1.10	0.05	0.45	1.00	1.06	0.24	0.70	1.00
X - 13431	Unknown	Unknown	0.89	0.39	0.71	1.00	1.01	0.25	0.65	1.00	0.94	0.78	0.96	1.00	1.13	0.02	0.37	1.00	1.05	0.35	0.69	1.00	0.93	0.10	0.64	1.00
X - 13729	Unknown	Unknown	1.12	0.18	0.53	1.00	1.16	0.16	0.56	1.00	1.16	0.04	0.37	1.00	1.04	0.43	0.84	1.00	1.04	0.85	0.95	1.00	1.00	0.95	0.99	1.00
X - 13866	Unknown	Unknown	1.03	0.80	0.94	1.00	0.99	0.90	0.98	1.00	1.00	0.39	0.86	1.00	0.96	0.83	0.97	1.00	0.97	0.50	0.77	1.00	1.01	0.89	0.97	1.00
X - 14056	Unknown	Unknown	0.97	0.98	0.99	1.00	1.05	0.60	0.86	1.00	0.99	0.80	0.96	1.00	1.07	0.29	0.79	1.00	1.02	0.95	0.99	1.00	0.95	0.49	0.82	1.00
X - 14939	Unknown	Unknown	1.08	0.66	0.87	1.00	0.96	0.50	0.81	1.00	1.17	0.15	0.61	1.00	0.89	0.08	0.62	1.00	1.08	0.58	0.81	1.00	1.21	0.09	0.62	1.00
X - 15245	Unknown	Unknown	1.08	0.60	0.84	1.00	1.05	0.53	0.83	1.00	0.93	0.35	0.83	1.00	0.98	0.89	0.97	1.00	0.87	0.54	0.80	1.00	0.89	0.39	0.78	1.00
X - 15469	Unknown	Unknown	1.06	0.34	0.67	1.00	1.11	0.23	0.61	1.00	1.02	0.59	0.94	1.00	1.04	0.51	0.88	1.00	0.96	0.53	0.79	1.00	0.92	0.15	0.66	1.00
X - 15486	Unknown	Unknown	1.15	0.17	0.52	1.00	1.03	0.80	0.94	1.00	1.11	0.42	0.89	1.00	0.89	0.25	0.76	1.00	0.97	0.61	0.83	1.00	1.09	0.42	0.78	1.00
X - 15492	Unknown	Unknown	1.12	0.08	0.41	1.00	1.12	0.07	0.42	1.00	1.24	0.00	0.05	1.00	0.99	0.97	0.99	1.00	1.10	0.01	0.26	1.00	1.11	0.02	0.44	1.00
X - 15503	Unknown	Unknown	0.92	0.12	0.47	1.00	0.96	0.38	0.75	1.00	1.00	0.73	0.95	1.00	1.04	0.31	0.81	1.00	1.08	0.17	0.58	1.00	1.04	0.79	0.94	1.00
X - 16087	Unknown	Unknown	1.09	0.62	0.85	1.00	0.99	0.99	1.00	1.00	1.02	0.93	1.00	1.00	0.92	0.35	0.81	1.00	0.94	0.52	0.79	1.00	1.02	0.87	0.97	1.00
X - 16580	Unknown	Unknown	1.02	0.85	0.95	1.00	0.88	0.27	0.67	1.00	0.93	0.35	0.83	1.00	0.86	0.11	0.62	1.00	0.91	0.27	0.63	1.00	1.06	0.53	0.83	1.00
X - 16938	Unknown	Unknown	1.06	0.50	0.78	1.00	0.88	0.06	0.41	1.00	1.01	0.84	0.98	1.00	0.83	0.00	0.11	1.00	0.95	0.35	0.69	1.00	1.15	0.01	0.44	1.00
X - 16944	Unknown	Unknown	1.01	0.97	0.98	1.00	0.86	0.11	0.47	1.00	1.06	0.37	0.84	1.00	0.85	0.03	0.45	1.00	1.05	0.84	0.95	1.00	1.24	0.08	0.61	1.00
X - 17335	Unknown	Unknown	1.11	0.20	0.55	1.00	1.07	0.95	1.00	1.00	1.01	0.89	0.99	1.00	0.96	0.41	0.83	1.00	0.90	0.16	0.56	1.00	0.95	0.28	0.72	1.00
X - 17337	Unknown	Unknown	1.06	0.90	0.96	1.00	1.05	0.33	0.73	1.00	0.97	0.80	0.96	1.00	0.98	0.36	0.81	1.00	0.92	0.55	0.80	1.00	0.93	0.10	0.64	1.00
X - 17340	Unknown	Unknown	1.11	0.14	0.48	1.00	1.12	0.05	0.36	1.00	1.19	0.01	0.16	1.00	1.01	0.78	0.95	1.00	1.07	0.11	0.53	1.00	1.06	0.33	0.75	1.00
X - 17357	Unknown	Unknown	0.97	0.46	0.76	1.00	0.95	0.59	0.86	1.00	0.91	0.05	0.37	1.00	0.98	0.50	0.88	1.00	0.94	0.11	0.53	1.00	0.96	0.14	0.65	1.00
X - 17653	Unknown	Unknown	0.97	0.94	0.98	1.00	1.00	0.90	0.98	1.00	1.10	0.17	0.64	1.00	1.03	0.79	0.96	1.00	1.13	0.06	0.47	1.00	1.10	0.23	0.69	1.00
X - 17654	Unknown	Unknown	0.95	0.15	0.51	1.00	0.95	0.32	0.72	1.00	1.01	0.35	0.83	1.00	1.01	0.85	0.97	1.00	1.07	0.13	0.54	1.00	1.06	0.11	0.64	1.00
X - 17676	Unknown	Unknown	0.69	0.00	0.03	0.78	0.70	0.00	0.01	0.07	0.75	0.00	0.08	1.00	1.01	0.99	1.00	1.00	1.08	0.40	0.72	1.00	1.08	0.21	0.68	1.00
X - 18249	Unknown	Unknown	0.90	0.01	0.10	1.00	0.86	0.00	0.01	0.01	0.83	0.00	0.00	0.00	0.96	0.24	0.76	1.00	0.92	0.01	0.26	1.00	0.96	0.10	0.64	1.00
X - 18779	Unknown	Unknown	0.94	0.29	0.63	1.00	0.98	0.72	0.90	1.00	0.95	0.34	0.83	1.00	1.04	0.78	0.95	1.00	1.01	0.98	0.99	1.00	0.97	0.43	0.78	1.00
X - 18913	Unknown	Unknown	0.46	0.00	0.03	0.83	0.48	0.00	0.02	0.26	0.46	0.00	0.02	0.14	1.03	0.16	0.66	1.00	1.00	0.37	0.70	1.00	0.97	0.78	0.94	1.00
X - 18914	Unknown	Unknown	0.95	0.13	0.47	1.00	0.89	0.00	0.03	0.44	0.89	0.00	0.02	0.13	0.94	0.03	0.44	1.00	0.94	0.02	0.37	1.00	1.00	0.78	0.94	1.00
X - 18921	Unknown	Unknown	1.12	0.29	0.63	1.00	0.96	0.95	1.00	1.00	0.95	0.61	0.94	1.00	0.86	0.06	0.57	1.00	0.85	0.04	0.45	1.00	0.99	0.66	0.88	1.00
X - 18922	Unknown	Unknown	0.97	0.62	0.85	1.00	0.94	0.06	0.41	1.00	1.04	0.97	1.00	1.00	0.97	0.37	0.81	1.00	1.07	0.59	0.82	1.00	1.10	0.26	0.71	1.00
X - 19141	Unknown	Unknown	0.97	0.75	0.92	1.00	0.93	0.16	0.56	1.00	1.00	0.85	0.98	1.00	0.96	0.40	0.83	1.00	1.04	0.59	0.82	1.00	1.08	0.11	0.64	1.00
X - 21258	Unknown	Unknown	0.85	0.29	0.63	1.00	1.00	0.40	0.76	1.00	1.02	0.34	0.83	1.00	1.18	0.33	0.81	1.00	1.20	0.78	0.92	1.00	1.02	0.64	0.88	1.00
X - 21286	Unknown	Unknown	1.04	0.43	0.74	1.00	1.03	0.50	0.81	1.00	0.99	0.75	0.95	1.00	0.99	0.73	0.94	1.00	0.95	0.58	0.81	1.00	0.97	0.60	0.86	1.00
X - 21310	Unknown	Unknown	0.93	0.21	0.55	1.00	1.00	0.88	0.97	1.00	0.98	0.90	0.99	1.00	1.08	0.10	0.62	1.00	1.05	0.48	0.76	1.00	0.98	0.59	0.86	1.00
X - 21319	Unknown	Unknown	1.05	0.46	0.76	1.00	0.88	0.29	0.68	1.00	1.11	0.30	0.81	1.00	0.84	0.02	0.37	1.00	1.05	0.54	0.80	1.00	1.26	0.04	0.49	1.00
X - 21339	Unknown	Unknown	0.89	0.09	0.43	1.00	0.92	0.24	0.64	1.00	0.98	0.52	0.93	1.00	1.03	0.43	0.84	1.00	1.09	0.33	0.68	1.00	1.06	0.89	0.97	1.00
X - 21353	Unknown	Unknown	1.22	0.03	0.21	1.00	1.11	0.15	0.55	1.00	1.06	0.30	0.81	1.00	0.91	0.29	0.79	1.00	0.87	0.24	0.63	1.00	0.95	0.39	0.78	1.00

X - 21383	Unknown	Unknown	1.03	0.80	0.94	1.00	1.06	0.76	0.92	1.00	1.08	0.29	0.80	1.00	1.02	0.68	0.93	1.00	1.05	0.83	0.95	1.00	1.03	0.09	0.62	1.00
X - 21411	Unknown	Unknown	0.84	0.02	0.16	1.00	0.90	0.15	0.55	1.00	1.05	0.60	0.94	1.00	1.07	0.10	0.62	1.00	1.26	0.04	0.45	1.00	1.17	0.62	0.87	1.00
X - 21628	Unknown	Unknown	1.01	0.92	0.97	1.00	0.91	0.00	0.06	1.00	0.98	0.34	0.83	1.00	0.90	0.03	0.44	1.00	0.97	0.70	0.88	1.00	1.08	0.04	0.48	1.00
X - 21736	Unknown	Unknown	1.07	0.94	0.98	1.00	0.95	0.28	0.67	1.00	1.01	0.54	0.93	1.00	0.89	0.11	0.62	1.00	0.94	0.58	0.81	1.00	1.06	0.57	0.86	1.00
X - 21785	Unknown	Unknown	0.98	0.80	0.94	1.00	0.98	0.79	0.94	1.00	1.02	0.39	0.87	1.00	1.00	0.92	0.98	1.00	1.05	0.09	0.52	1.00	1.05	0.12	0.64	1.00
X - 21796	Unknown	Unknown	0.97	0.45	0.76	1.00	0.93	0.22	0.61	1.00	1.05	0.27	0.79	1.00	0.95	0.43	0.84	1.00	1.08	0.30	0.65	1.00	1.13	0.06	0.57	1.00
X - 21829	Unknown	Unknown	1.03	0.58	0.83	1.00	0.98	0.79	0.94	1.00	0.98	0.68	0.94	1.00	0.95	0.55	0.90	1.00	0.95	0.41	0.72	1.00	1.00	0.63	0.87	1.00
X - 22162	Unknown	Unknown	1.02	0.60	0.84	1.00	0.98	0.85	0.96	1.00	1.11	0.18	0.66	1.00	0.96	0.53	0.89	1.00	1.09	0.03	0.43	1.00	1.13	0.01	0.44	1.00
X - 22519	Unknown	Unknown	1.02	0.79	0.93	1.00	0.98	0.72	0.90	1.00	0.99	0.45	0.90	1.00	0.97	0.51	0.88	1.00	0.97	0.52	0.79	1.00	1.00	0.62	0.87	1.00
X - 22771	Unknown	Unknown	0.91	0.14	0.50	1.00	0.88	0.06	0.41	1.00	0.96	0.58	0.94	1.00	0.97	0.33	0.81	1.00	1.05	0.15	0.55	1.00	1.08	0.03	0.48	1.00
X - 22775	Unknown	Unknown	0.98	0.69	0.89	1.00	0.96	0.16	0.55	1.00	1.00	0.81	0.97	1.00	0.98	0.39	0.82	1.00	1.02	0.74	0.90	1.00	1.04	0.16	0.66	1.00
X - 23314	Unknown	Unknown	0.58	0.20	0.55	1.00	0.57	0.15	0.55	1.00	0.71	0.68	0.94	1.00	0.99	0.49	0.88	1.00	1.22	0.20	0.59	1.00	1.24	0.90	0.97	1.00
X - 23369	Unknown	Unknown	0.88	0.17	0.52	1.00	0.81	0.00	0.06	1.00	0.86	0.03	0.35	1.00	0.92	0.30	0.81	1.00	0.98	0.89	0.96	1.00	1.06	0.33	0.75	1.00
X - 23585	Unknown	Unknown	1.00	0.76	0.92	1.00	1.02	0.83	0.95	1.00	1.01	0.92	1.00	1.00	1.02	0.83	0.97	1.00	1.01	0.43	0.73	1.00	0.98	0.74	0.91	1.00
X - 23593	Unknown	Unknown	0.96	0.59	0.84	1.00	0.90	0.14	0.54	1.00	1.01	0.98	1.00	1.00	0.94	0.10	0.62	1.00	1.05	0.21	0.61	1.00	1.12	0.01	0.44	1.00
X - 23639	Unknown	Unknown	1.07	0.27	0.61	1.00	1.00	0.85	0.96	1.00	1.10	0.24	0.75	1.00	0.94	0.44	0.85	1.00	1.03	0.64	0.85	1.00	1.10	0.14	0.65	1.00
X - 23680	Unknown	Unknown	1.00	0.74	0.92	1.00	1.02	0.80	0.94	1.00	0.97	0.68	0.94	1.00	1.02	0.89	0.97	1.00	0.98	0.87	0.96	1.00	0.96	0.55	0.85	1.00
X - 23974	Unknown	Unknown	1.11	0.05	0.30	1.00	1.04	0.23	0.62	1.00	1.05	0.34	0.83	1.00	0.94	0.33	0.81	1.00	0.95	0.44	0.74	1.00	1.01	0.52	0.83	1.00
X - 23997	Unknown	Unknown	1.03	0.94	0.98	1.00	1.07	0.48	0.81	1.00	1.01	0.85	0.98	1.00	1.05	0.36	0.81	1.00	0.99	0.93	0.98	1.00	0.94	0.44	0.80	1.00
X - 24106 - retired for palmitoyl-sphingosine-pho	Unknown	Unknown	0.98	0.74	0.92	1.00	0.99	0.66	0.88	1.00	0.98	0.51	0.93	1.00	1.01	0.63	0.92	1.00	1.00	0.97	0.99	1.00	0.99	0.63	0.87	1.00
X - 24435	Unknown	Unknown	0.83	0.02	0.15	1.00	0.86	0.08	0.44	1.00	0.81	0.00	0.05	0.95	1.04	0.36	0.81	1.00	0.97	0.90	0.97	1.00	0.93	0.60	0.86	1.00
X - 24549	Unknown	Unknown	0.81	0.01	0.12	1.00	0.96	0.39	0.76	1.00	0.97	0.47	0.92	1.00	1.18	0.00	0.10	1.00	1.19	0.00	0.13	1.00	1.01	0.92	0.98	1.00
X - 24588	Unknown	Unknown	0.93	0.30	0.63	1.00	0.91	0.09	0.45	1.00	0.96	0.26	0.77	1.00	0.97	0.37	0.81	1.00	1.03	0.43	0.73	1.00	1.06	0.13	0.64	1.00
X - 24699	Unknown	Unknown	1.02	0.55	0.82	1.00	1.00	0.58	0.86	1.00	1.05	0.09	0.48	1.00	0.98	0.55	0.90	1.00	1.04	0.24	0.63	1.00	1.05	0.17	0.66	1.00
X - 24765	Unknown	Unknown	1.00	0.80	0.94	1.00	0.96	0.50	0.81	1.00	1.02	0.88	0.99	1.00	0.96	0.21	0.72	1.00	1.01	0.66	0.85	1.00	1.06	0.18	0.67	1.00
X - 24813	Unknown	Unknown	0.95	0.30	0.63	1.00	0.93	0.15	0.54	1.00	0.94	0.24	0.75	1.00	0.98	0.72	0.94	1.00	1.00	0.85	0.95	1.00	1.01	0.85	0.97	1.00
X - 24951	Unknown	Unknown	0.85	0.08	0.40	1.00	1.10	0.45	0.80	1.00	0.98	0.73	0.95	1.00	1.30	0.01	0.32	1.00	1.16	0.15	0.56	1.00	0.89	0.32	0.75	1.00
X - 24952	Unknown	Unknown	0.85	0.25	0.60	1.00	1.15	0.01	0.19	1.00	1.05	0.49	0.92	1.00	1.35	0.00	0.10	1.00	1.24	0.01	0.28	1.00	0.92	0.27	0.72	1.00
X - 25422	Unknown	Unknown	0.97	0.90	0.96	1.00	0.97	0.70	0.90	1.00	0.98	0.81	0.97	1.00	1.00	0.89	0.97	1.00	1.02	0.16	0.56	1.00	1.01	0.84	0.95	1.00
(14 or 15)-methylpalmitate (a17:0 or i17:0)	Lipid	Fatty Acid, Branched	1.14	0.55	0.82	1.00	1.08	0.66	0.88	1.00	0.99	0.85	0.98	1.00	0.94	0.58	0.90	1.00	0.86	0.24	0.63	1.00	0.92	0.33	0.75	1.00
1-(1-enyl-oleoyl)-GPE (P-18:1)*	Lipid	Lysoplasmalogen	1.03	0.83	0.95	1.00	0.92	0.80	0.94	1.00	0.96	0.89	0.99	1.00	0.90	0.70	0.93	1.00	0.94	0.99	0.99	1.00	1.04	0.92	0.98	1.00
1,2,3-benzenetriol sulfate (2)	Xenobiotics	Chemical	0.46	0.01	0.15	1.00	0.58	0.10	0.46	1.00	0.90	0.42	0.89	1.00	1.26	0.22	0.75	1.00	1.97	0.27	0.63	1.00	1.56	0.55	0.85	1.00
1,3,7-trimethylurate	Xenobiotics	Xanthine Metabolism	0.54	0.00	0.03	0.73	0.61	0.00	0.05	1.00	0.69	0.01	0.21	1.00	1.12	0.41	0.83	1.00	1.28	0.18	0.58	1.00	1.14	0.19	0.68	1.00
12,13-DiHOME	Lipid	Fatty Acid, Dihydroxy	0.63	0.01	0.12	1.00	0.62	0.01	0.19	1.00	0.58	0.01	0.12	1.00	1.00	0.44	0.85	1.00	0.93	0.94	0.98	1.00	0.94	0.61	0.87	1.00
12-HETE	Lipid	Eicosanoid	1.24	0.02	0.18	1.00	1.11	0.49	0.81	1.00	1.18	0.11	0.53	1.00	0.89	0.15	0.66	1.00	0.95	0.79	0.92	1.00	1.07	0.29	0.74	1.00
12-HHTrE	Lipid	Eicosanoid	1.12	0.17	0.52	1.00	0.91	0.72	0.90	1.00	1.19	0.03	0.35	1.00	0.81	0.07	0.59	1.00	1.05	0.99	0.99	1.00	1.31	0.03	0.47	1.00
1-arachidonoyl-GPA (20:4)	Lipid	Lysophospholipid	1.00	0.88	0.96	1.00	1.01	0.93	0.99	1.00	0.97	0.69	0.94	1.00	1.01	0.69	0.93	1.00	0.97	0.60	0.82	1.00	0.96	0.97	0.99	1.00
1-carboxyethylisoleucine	Amino Acid	Leucine, Isoleucine and Valine Meta	0.44	0.00	0.02	0.26	0.54	0.00	0.04	0.73	0.51	0.00	0.03	0.38	1.22	0.41	0.83	1.00	1.16	0.08	0.49	1.00	0.95	0.48	0.82	1.00
1-carboxyethylleucine	Amino Acid	Leucine, Isoleucine and Valine Meta	0.55	0.00	0.04	1.00	0.65	0.02	0.25	1.00	0.60	0.00	0.10	1.00	1.19	0.11	0.62	1.00	1.10	0.06	0.47	1.00	0.93	0.93	0.98	1.00
1-carboxyethylvaline	Amino Acid	Leucine, Isoleucine and Valine Meta	0.57	0.00	0.03	0.89	0.67	0.04	0.34	1.00	0.64	0.02	0.27	1.00	1.19	0.05	0.55	1.00	1.13	0.04	0.45	1.00	0.95	0.88	0.97	1.00
1-heptadecenoylglycerol (17:1)*	Lipid	Monoacylglycerol	0.86	0.58	0.83	1.00	0.79	0.13	0.50	1.00	0.79	0.44	0.90	1.00	0.91	0.47	0.88	1.00	0.92	0.80	0.93	1.00	1.00	0.58	0.86	1.00
1-lignoceroyl-GPC (24:0)	Lipid	Lysophospholipid	0.92	0.17	0.52	1.00	1.07	0.43	0.78	1.00	1.02	0.75	0.95	1.00	1.16	0.01	0.26	1.00	1.11	0.04	0.45	1.00	0.95	0.41	0.78	1.00
1-linolenoylglycerol (18:3)	Lipid	Monoacylglycerol	0.80	0.21	0.56	1.00	0.97	0.81	0.95	1.00	0.86	0.31	0.81	1.00	1.21	0.04	0.52	1.00	1.08	0.24	0.63	1.00	0.89	0.16	0.66	1.00
1-linoleoyl-2-arachidonoyl-GPC (18:2/20:4n6)*	Lipid	Phosphatidylcholine (PC)	0.94	0.39	0.71	1.00	1.00	1.00	1.00	1.00	0.96	0.21	0.71	1.00	1.06	0.21	0.73	1.00	1.02	0.51	0.78	1.00	0.96	0.41	0.78	1.00
1-linoleoyl-GPG (18:2)*	Lipid	Lysophospholipid	0.77	0.01	0.12	1.00	0.74	0.02	0.23	1.00	0.70	0.00	0.07	1.00	0.96	0.93	0.98	1.00	0.91	0.67	0.85	1.00	0.95	0.42	0.78	1.00
1-methyl-5-imidazoleacetate	Amino Acid	Histidine Metabolism	1.07	0.62	0.85	1.00	1.23	0.43	0.78	1.00	1.19	0.29	0.80	1.00	1.15	0.23	0.75	1.00	1.11	0.88	0.96	1.00	0.97	0.43	0.78	1.00
1-methylurate	Xenobiotics	Xanthine Metabolism	0.99	0.78	0.93	1.00	1.05	0.81	0.95	1.00	1.18	0.18	0.67	1.00	1.06	0.70	0.93	1.00	1.20	0.28	0.63	1.00	1.13	0.39	0.78	1.00
1-methylxanthine	Xenobiotics	Xanthine Metabolism	0.82	0.08	0.42	1.00	0.88	0.09	0.45	1.00	0.98	0.98	1.00	1.00	1.06	0.74	0.95	1.00	1.19	0.16	0.56	1.00	1.12	0.10	0.64	1.00
1-oleoyl-2-arachidonoyl-GPE (18:1/20:4)*	Lipid	Phosphatidylethanolamine (PE)	0.84	0.06	0.33	1.00	0.85	0.11	0.48	1.00	0.97	0.88	0.99	1.00	1.02	0.64	0.92	1.00	1.16	0.03	0.42	1.00	1.14	0.06	0.56	1.00
1-palmitoleoyl-2-linolenoyl-GPC (16:1/18:3)*	Lipid	Phosphatidylcholine (PC)	1.02	0.75	0.92	1.00	1.18	0.05	0.36	1.00	1.10	0.48	0.92	1.00	1.16	0.01	0.26	1.00	1.08	0.20	0.59	1.00	0.94	0.61	0.87	1.00
1-palmitoyl-2-oleoyl-GPE (16:0/18:1)	Lipid	Phosphatidylethanolamine (PE)	0.73	0.00	0.02	0.26	0.80	0.04	0.34	1.00	0.87	0.04	0.35	1.00	1.11	0.14	0.66	1.00	1.20	0.00	0.14	1.00	1.08	0.39	0.78	1.00
1-palmitoyl-GPG (16:0)*	Lipid	Lysophospholipid	0.75	0.01	0.12	1.00	0.70	0.00	0.10	1.00	0.76	0.01	0.20	1.00	0.94	0.97	0.99	1.00	1.02	0.38	0.70	1.00	1.09	0.16	0.66	1.00
1-stearoyl-2-oleoyl-GPE (18:0/18:1)	Lipid	Phosphatidylethanolamine (PE)	0.64	0.00	0.02	0.51	0.73	0.02	0.25	1.00	0.81	0.06	0.39	1.00	1.14	0.17	0.68	1.00	1.26	0.00	0.26	1.00	1.11	0.38	0.78	1.00
1-stearoyl-2-oleoyl-GPS (18:0/18:1)	Lipid	Phosphatidylserine (PS)	1.04	0.85	0.95	1.00	1.00	0.99	1.00	1.00	1.04	0.83	0.98	1.00	0.96	0.95	0.99	1.00	1.00	0.66	0.85	1.00	1.05	0.90	0.97	1.00

1-stearoyl-GPG (18:0)	Lipid	Lysophospholipid	0.87	0.17	0.52	1.00	0.85	0.17	0.57	1.00	0.92	0.35	0.83	1.00	0.97	0.98	0.99	1.00	1.05	0.53	0.79	1.00	1.08	0.39	0.78	1.00
2,2'-Methylenebis(6-tert-butyl-p-cresol)	Xenobiotics	Chemical	1.15	0.28	0.62	1.00	1.16	0.06	0.41	1.00	0.87	0.70	0.94	1.00	1.01	0.76	0.95	1.00	0.76	0.14	0.55	1.00	0.75	0.01	0.44	1.00
2,3-dihydroxy-2-methylbutyrate	Amino Acid	Leucine, Isoleucine and Valine Meta	0.78	0.07	0.39	1.00	0.80	0.04	0.35	1.00	0.85	0.06	0.40	1.00	1.02	1.00	1.00	1.00	1.08	0.94	0.98	1.00	1.06	0.74	0.91	1.00
2,3-dihydroxyisovalerate	Xenobiotics	Food Component/Plant	0.60	0.20	0.55	1.00	0.73	0.27	0.66	1.00	0.90	0.39	0.87	1.00	1.22	0.34	0.81	1.00	1.50	0.27	0.63	1.00	1.23	0.93	0.98	1.00
21-hydroxypregnenolone disulfate	Lipid	Pregnenolone Steroids	1.02	0.64	0.86	1.00	0.95	0.69	0.89	1.00	1.07	0.41	0.89	1.00	0.93	0.10	0.62	1.00	1.05	0.49	0.77	1.00	1.13	0.04	0.49	1.00
2-aminoadipate	Amino Acid	Lysine Metabolism	0.94	0.49	0.77	1.00	1.05	0.99	1.00	1.00	0.95	0.60	0.94	1.00	1.12	0.17	0.69	1.00	1.01	0.75	0.91	1.00	0.90	0.10	0.64	1.00
2-aminoheptanoate	Lipid	Fatty Acid, Amino	1.50	0.01	0.08	1.00	1.13	0.44	0.79	1.00	1.34	0.13	0.57	1.00	0.75	0.01	0.23	1.00	0.89	0.08	0.49	1.00	1.18	0.29	0.74	1.00
2-arachidonoylglycerol (20:4)	Lipid	Monoacylglycerol	0.91	0.22	0.56	1.00	1.06	0.88	0.97	1.00	0.79	0.08	0.45	1.00	1.17	0.02	0.37	1.00	0.88	0.63	0.84	1.00	0.75	0.02	0.45	1.00
2'-deoxyuridine	Nucleotide	Pyrimidine Metabolism, Uracil cont	1.18	0.14	0.49	1.00	1.02	0.94	1.00	1.00	1.10	0.26	0.77	1.00	0.87	0.12	0.66	1.00	0.93	0.29	0.65	1.00	1.08	0.33	0.75	1.00
2-docosahexaenoylglycerol (22:6)*	Lipid	Monoacylglycerol	0.88	0.24	0.59	1.00	1.04	0.67	0.88	1.00	0.98	0.81	0.97	1.00	1.18	0.06	0.57	1.00	1.11	0.18	0.58	1.00	0.94	0.47	0.82	1.00
2-hydroxybehenate	Lipid	Fatty Acid, Monohydroxy	0.85	0.01	0.15	1.00	0.92	0.08	0.45	1.00	0.92	0.07	0.43	1.00	1.08	0.23	0.75	1.00	1.08	0.33	0.68	1.00	1.00	1.00	1.00	1.00
2-hydroxyhippurate (salicylurate)	Xenobiotics	Benzoate Metabolism	0.78	0.08	0.39	1.00	2.72	0.03	0.30	1.00	1.35	0.59	0.94	1.00	3.49	0.79	0.96	1.00	1.74	0.15	0.56	1.00	0.50	0.10	0.64	1.00
2-hydroxyphenylacetate	Amino Acid	Phenylalanine Metabolism	0.92	0.30	0.63	1.00	0.92	0.29	0.69	1.00	1.01	0.41	0.89	1.00	1.00	0.89	0.97	1.00	1.09	0.40	0.72	1.00	1.09	0.99	1.00	1.00
2-keto-3-deoxy-gluconate	Xenobiotics	Food Component/Plant	0.82	0.03	0.23	1.00	0.88	0.05	0.37	1.00	0.90	0.16	0.63	1.00	1.07	0.29	0.80	1.00	1.11	0.21	0.60	1.00	1.03	0.41	0.78	1.00
2-linoleoylglycerol (18:2)	Lipid	Monoacylglycerol	0.84	0.11	0.47	1.00	0.93	0.37	0.74	1.00	0.85	0.05	0.39	1.00	1.10	0.16	0.66	1.00	1.01	0.62	0.83	1.00	0.92	0.23	0.70	1.00
2-methylbutyrylcarnitine (C5)	Amino Acid	Leucine, Isoleucine and Valine Meta	0.77	0.00	0.01	0.04	0.83	0.01	0.17	1.00	0.85	0.00	0.04	0.55	1.09	0.10	0.62	1.00	1.11	0.15	0.56	1.00	1.02	0.73	0.91	1.00
2-myristoylglycerol (14:0)	Lipid	Monoacylglycerol	0.81	0.88	0.96	1.00	0.89	0.88	0.97	1.00	1.13	0.04	0.36	1.00	1.10	0.23	0.75	1.00	1.39	0.00	0.14	1.00	1.27	0.02	0.47	1.00
2-naphthol sulfate	Xenobiotics	Chemical	0.99	0.66	0.87	1.00	0.77	0.10	0.46	1.00	1.15	0.73	0.95	1.00	0.78	0.13	0.66	1.00	1.17	0.22	0.61	1.00	1.50	0.02	0.44	1.00
2'-O-methyluridine	Nucleotide	Pyrimidine Metabolism, Uracil cont	0.93	0.48	0.77	1.00	0.93	0.18	0.58	1.00	1.05	0.48	0.92	1.00	0.99	0.66	0.92	1.00	1.13	0.05	0.45	1.00	1.14	0.02	0.44	1.00
2-oxoarginine*	Amino Acid	Urea cycle; Arginine and Proline Met	0.66	0.00	0.04	1.00	0.72	0.03	0.32	1.00	0.78	0.03	0.35	1.00	1.10	0.10	0.62	1.00	1.18	0.04	0.45	1.00	1.07	0.49	0.82	1.00
2-palmitoleoylglycerol (16:1)*	Lipid	Monoacylglycerol	0.81	0.38	0.70	1.00	0.88	0.72	0.90	1.00	0.85	0.33	0.82	1.00	1.08	0.33	0.81	1.00	1.04	0.39	0.72	1.00	0.96	0.73	0.91	1.00
2-palmitoleoyl-GPC* (16:1)*	Lipid	Lysophospholipid	0.72	0.01	0.13	1.00	0.86	0.16	0.55	1.00	0.91	0.45	0.90	1.00	1.21	0.14	0.66	1.00	1.27	0.23	0.63	1.00	1.05	0.72	0.91	1.00
3-(3-amino-3-carboxypropyl)uridine*	Nucleotide	Pyrimidine Metabolism, Uracil cont	0.95	0.21	0.55	1.00	0.96	0.27	0.67	1.00	1.02	0.51	0.93	1.00	1.01	0.67	0.92	1.00	1.07	0.12	0.54	1.00	1.07	0.17	0.66	1.00
3-(3-hydroxyphenyl)propionate sulfate	Xenobiotics	Benzoate Metabolism	0.91	0.52	0.80	1.00	1.07	0.83	0.95	1.00	1.12	0.68	0.94	1.00	1.17	0.81	0.97	1.00	1.23	0.76	0.92	1.00	1.05	0.40	0.78	1.00
3,4-methyleneheptanoate	Xenobiotics	Food Component/Plant	1.19	0.42	0.73	1.00	0.95	0.66	0.88	1.00	1.14	0.54	0.93	1.00	0.80	0.24	0.76	1.00	0.96	0.93	0.98	1.00	1.20	0.07	0.57	1.00
3,7-dimethylurate	Xenobiotics	Xanthine Metabolism	0.66	0.10	0.44	1.00	0.77	0.39	0.76	1.00	0.99	0.90	0.99	1.00	1.17	0.29	0.79	1.00	1.51	0.07	0.48	1.00	1.29	0.20	0.68	1.00
3b-hydroxy-5-cholenoic acid	Lipid	Secondary Bile Acid Metabolism	1.09	0.50	0.78	1.00	0.85	0.12	0.49	1.00	0.93	0.40	0.88	1.00	0.78	0.06	0.57	1.00	0.85	0.20	0.59	1.00	1.10	0.52	0.83	1.00
3-ethylcatechol sulfate (1)	Xenobiotics	Food Component/Plant	0.61	0.01	0.12	1.00	0.52	0.00	0.01	0.11	0.58	0.00	0.03	0.38	0.85	0.29	0.79	1.00	0.94	0.29	0.65	1.00	1.11	0.60	0.86	1.00
3-hydroxyadipate*	Lipid	Fatty Acid, Dicarboxylate	0.85	0.25	0.60	1.00	0.75	0.11	0.47	1.00	0.70	0.02	0.25	1.00	0.89	0.63	0.92	1.00	0.82	0.18	0.58	1.00	0.93	0.46	0.82	1.00
3-hydroxybutyroylglycine	Lipid	Fatty Acid Metabolism(Acyl Glycine)	1.08	0.55	0.82	1.00	0.88	0.36	0.74	1.00	0.85	0.19	0.67	1.00	0.82	0.05	0.53	1.00	0.79	0.09	0.52	1.00	0.97	0.75	0.92	1.00
3-hydroxybutyrylcarnitine (1)	Lipid	Fatty Acid Metabolism(Acyl Carnitin	0.98	0.74	0.92	1.00	0.77	0.35	0.74	1.00	0.68	0.05	0.39	1.00	0.78	0.14	0.66	1.00	0.69	0.16	0.56	1.00	0.88	0.32	0.75	1.00
3-hydroxybutyrylcarnitine (2)	Lipid	Fatty Acid Metabolism(Acyl Carnitin	0.92	0.46	0.76	1.00	0.87	0.24	0.64	1.00	0.84	0.03	0.32	1.00	0.95	0.37	0.81	1.00	0.92	0.19	0.58	1.00	0.97	0.27	0.72	1.00
3-hydroxyhippurate sulfate	Xenobiotics	Benzoate Metabolism	1.07	0.27	0.61	1.00	1.37	0.67	0.88	1.00	1.31	0.49	0.92	1.00	1.28	0.32	0.81	1.00	1.22	0.80	0.93	1.00	0.96	0.43	0.78	1.00
3-hydroxyisobutyrate	Amino Acid	Leucine, Isoleucine and Valine Meta	0.95	0.16	0.52	1.00	0.99	0.33	0.73	1.00	0.92	0.21	0.71	1.00	1.04	0.62	0.91	1.00	0.97	0.84	0.95	1.00	0.94	0.51	0.83	1.00
3-hydroxymyristate	Lipid	Fatty Acid, Monohydroxy	1.28	0.10	0.45	1.00	1.29	0.03	0.27	1.00	1.17	0.06	0.40	1.00	1.01	0.97	0.99	1.00	0.92	0.38	0.70	1.00	0.91	0.19	0.68	1.00
3-hydroxysebacate	Lipid	Fatty Acid, Monohydroxy	1.11	0.89	0.96	1.00	0.88	0.28	0.67	1.00	0.74	0.05	0.39	1.00	0.79	0.02	0.37	1.00	0.66	0.20	0.59	1.00	0.84	0.62	0.87	1.00
3-hydroxystachydrine*	Xenobiotics	Food Component/Plant	0.49	0.24	0.59	1.00	0.58	0.53	0.83	1.00	0.63	0.13	0.57	1.00	1.18	0.10	0.62	1.00	1.29	0.79	0.92	1.00	1.09	0.78	0.94	1.00
3-methoxytyrosine	Amino Acid	Tyrosine Metabolism	1.05	0.67	0.88	1.00	0.94	0.36	0.74	1.00	1.08	0.42	0.89	1.00	0.89	0.08	0.62	1.00	1.02	0.78	0.92	1.00	1.14	0.03	0.48	1.00
3-methyl catechol sulfate (1)	Xenobiotics	Benzoate Metabolism	0.42	0.00	0.01	0.07	0.43	0.00	0.01	0.06	0.47	0.00	0.04	0.63	1.03	0.57	0.90	1.00	1.11	0.58	0.81	1.00	1.08	0.49	0.82	1.00
3-methyladipate	Lipid	Fatty Acid, Dicarboxylate	0.97	0.53	0.81	1.00	0.93	0.61	0.86	1.00	0.90	0.20	0.68	1.00	0.96	0.81	0.97	1.00	0.93	0.39	0.71	1.00	0.97	0.80	0.94	1.00
3-methylglutarylcarnitine (2)	Amino Acid	Leucine, Isoleucine and Valine Meta	1.08	0.92	0.97	1.00	1.07	0.45	0.80	1.00	1.13	0.28	0.79	1.00	0.99	0.59	0.91	1.00	1.05	0.23	0.61	1.00	1.06	0.16	0.66	1.00
3-methylxanthine	Xenobiotics	Xanthine Metabolism	0.70	0.18	0.53	1.00	0.77	0.47	0.80	1.00	1.00	0.68	0.94	1.00	1.11	0.51	0.88	1.00	1.44	0.06	0.46	1.00	1.30	0.04	0.48	1.00
3-phenylpropionate (hydrocinnamate)	Xenobiotics	Benzoate Metabolism	1.02	0.95	0.98	1.00	0.88	0.51	0.82	1.00	1.01	0.42	0.89	1.00	0.86	0.21	0.72	1.00	0.99	0.94	0.98	1.00	1.15	0.10	0.64	1.00
3-sulfo-L-alanine	Amino Acid	Methionine, Cysteine, SAM and Tau	0.93	0.36	0.68	1.00	0.95	0.39	0.76	1.00	0.99	0.51	0.93	1.00	1.01	0.92	0.98	1.00	1.06	0.83	0.95	1.00	1.05	0.39	0.78	1.00
4-cholesten-3-one	Lipid	Sterol	1.00	0.95	0.98	1.00	0.95	0.80	0.94	1.00	0.95	0.37	0.84	1.00	0.95	0.45	0.85	1.00	0.95	0.26	0.63	1.00	0.99	0.97	0.99	1.00
4-guanidinobutanoate	Amino Acid	Guanidino and Acetamido Metaboli	0.78	0.23	0.57	1.00	0.73	0.20	0.60	1.00	0.83	0.52	0.93	1.00	0.94	0.62	0.91	1.00	1.07	0.54	0.80	1.00	1.14	0.14	0.65	1.00
4-hydroxycoumarin	Xenobiotics	Drug - Cardiovascular	0.99	0.84	0.95	1.00	1.04	0.69	0.89	1.00	0.97	0.84	0.98	1.00	1.05	0.69	0.93	1.00	0.98	0.73	0.89	1.00	0.93	0.70	0.90	1.00
4-hydroxyhippurate	Xenobiotics	Benzoate Metabolism	0.79	0.10	0.45	1.00	1.24	0.75	0.92	1.00	1.34	1.00	1.00	1.00	1.56	0.06	0.57	1.00	1.68	0.08	0.49	1.00	1.08	0.68	0.89	1.00
4-methoxyphenol sulfate	Amino Acid	Tyrosine Metabolism	1.45	0.06	0.36	1.00	1.18	0.39	0.76	1.00	1.19	0.35	0.83	1.00	0.81	0.39	0.82	1.00	0.82	0.31	0.66	1.00	1.00	0.73	0.91	1.00
4-methylguaiacol sulfate	Xenobiotics	Benzoate Metabolism	1.26	0.38	0.70	1.00	1.34	0.88	0.97	1.00	1.60	0.67	0.94	1.00	1.07	0.93	0.98	1.00	1.27	0.97	0.99	1.00	1.19	0.25	0.71	1.00
5-(galactosylhydroxy)-L-lysine	Amino Acid	Lysine Metabolism	0.95	0.89	0.96	1.00	1.00	0.98	1.00	1.00	1.05	0.45	0.90	1.00	1.05	0.85	0.97	1.00	1.10	0.20	0.59	1.00	1.05	0.38	0.78	1.00
5,6-dihydrothymine	Nucleotide	Pyrimidine Metabolism, Thymine co	1.02	0.44	0.75	1.00	1.03	0.18	0.58	1.00	1.04	0.51	0.93	1.00	1.01	0.83	0.97	1.00	1.02	0.92	0.98	1.00	1.01	0.67	0.89	1.00
5,6-dihydrouracil	Nucleotide	Pyrimidine Metabolism, Uracil cont	1.14	0.14	0.49	1.00	1.12	0.18	0.58	1.00	1.18	0.04	0.35	1.00	0.98	0.55	0.90	1.00	1.03	0.45	0.74	1.00	1.05	0.27	0.72	1.00
5-acetylamino-6-formylamino-3-methyluracil	Xenobiotics	Xanthine Metabolism	0.92	0.84	0.95	1.00	0.97	0.93	0.99	1.00	1.11	0.48	0.92	1.00	1.06	0.79	0.96	1.00	1.21	0.37	0.70	1.00	1.14	0.12	0.64	1.00

5alpha-androstan-3alpha,17beta-diol monosulfa	Lipid	Androgenic Steroids	1.00	0.72	0.91	1.00	0.95	0.61	0.86	1.00	0.98	0.97	1.00	1.00	0.95	0.75	0.95	1.00	0.97	0.27	0.63	1.00	1.03	0.27	0.72	1.00
5alpha-androstan-3beta,17alpha-diol disulfate	Lipid	Androgenic Steroids	1.07	0.92	0.97	1.00	1.07	0.74	0.91	1.00	1.05	0.63	0.94	1.00	1.00	0.97	0.99	1.00	0.98	0.64	0.85	1.00	0.98	0.73	0.91	1.00
5alpha-androstan-3beta,17beta-diol disulfate	Lipid	Androgenic Steroids	1.06	0.87	0.96	1.00	0.94	0.87	0.97	1.00	1.12	0.27	0.79	1.00	0.89	0.49	0.88	1.00	1.05	0.52	0.79	1.00	1.19	0.31	0.75	1.00
5alpha-pregnan-3beta,20alpha-diol disulfate	Lipid	Progestin Steroids	1.04	0.98	0.99	1.00	0.95	0.49	0.81	1.00	1.02	0.81	0.97	1.00	0.92	0.73	0.94	1.00	0.98	0.66	0.85	1.00	1.07	0.55	0.85	1.00
5alpha-pregnan-3beta,20alpha-diol monosulfate	Lipid	Progestin Steroids	0.92	0.11	0.47	1.00	0.91	0.23	0.61	1.00	0.88	0.09	0.49	1.00	0.99	0.52	0.88	1.00	0.96	0.95	0.99	1.00	0.98	0.64	0.88	1.00
5alpha-pregnan-3beta,20beta-diol monosulfate (1	Lipid	Progestin Steroids	1.03	0.62	0.85	1.00	1.00	0.39	0.76	1.00	0.99	0.88	0.99	1.00	0.97	0.55	0.90	1.00	0.96	0.22	0.61	1.00	0.98	0.50	0.82	1.00
5-hydroxyhexanoate	Lipid	Fatty Acid, Monohydroxy	1.02	0.76	0.92	1.00	0.95	0.52	0.82	1.00	1.02	0.99	1.00	1.00	0.93	0.08	0.62	1.00	1.00	0.98	0.99	1.00	1.08	0.43	0.78	1.00
7-methylxanthine	Xenobiotics	Xanthine Metabolism	0.71	0.20	0.55	1.00	0.84	0.60	0.86	1.00	1.03	0.42	0.89	1.00	1.18	0.07	0.59	1.00	1.44	0.01	0.27	1.00	1.22	0.09	0.63	1.00
9,10-DiHOME	Lipid	Fatty Acid, Dihydroxy	0.47	0.01	0.14	1.00	0.44	0.01	0.16	1.00	0.40	0.01	0.13	1.00	0.94	0.45	0.85	1.00	0.85	0.27	0.63	1.00	0.90	0.29	0.74	1.00
adenine	Nucleotide	Purine Metabolism, Adenine contai	1.17	0.12	0.47	1.00	1.03	0.92	0.98	1.00	1.23	0.21	0.71	1.00	0.88	0.04	0.52	1.00	1.05	0.60	0.82	1.00	1.19	0.09	0.62	1.00
adipoylcarnitine (C6-DC)	Lipid	Fatty Acid Metabolism(Acyl Carnitin	0.95	0.69	0.89	1.00	0.91	0.94	1.00	1.00	0.96	0.99	1.00	1.00	0.95	0.49	0.88	1.00	1.01	0.92	0.98	1.00	1.06	0.42	0.78	1.00
adrenoylcarnitine (C22:4)*	Lipid	Fatty Acid Metabolism(Acyl Carnitin	0.94	0.66	0.87	1.00	1.05	0.72	0.90	1.00	1.08	0.68	0.94	1.00	1.12	0.30	0.81	1.00	1.14	0.27	0.63	1.00	1.02	0.53	0.83	1.00
alpha-ketobutyrate	Amino Acid	Methionine, Cysteine, SAM and Tau	1.03	0.97	0.98	1.00	0.96	0.29	0.68	1.00	0.79	0.02	0.27	1.00	0.92	0.53	0.89	1.00	0.76	0.01	0.36	1.00	0.82	0.11	0.64	1.00
andro steroid monosulfate C19H28O6S (1)*	Lipid	Androgenic Steroids	0.96	0.42	0.73	1.00	0.89	0.11	0.46	1.00	1.02	0.94	1.00	1.00	0.93	0.14	0.66	1.00	1.06	0.92	0.98	1.00	1.15	0.08	0.61	1.00
androstenediol (3alpha, 17alpha) monosulfate (3	Lipid	Androgenic Steroids	1.00	0.88	0.96	1.00	0.98	0.64	0.87	1.00	0.97	0.98	1.00	1.00	0.98	0.99	1.00	1.00	0.97	0.99	0.99	1.00	0.99	0.88	0.97	1.00
androstenediol (3beta,17beta) monosulfate (2)	Lipid	Androgenic Steroids	0.97	0.76	0.92	1.00	0.91	0.67	0.88	1.00	1.07	0.47	0.92	1.00	0.94	0.15	0.66	1.00	1.10	0.13	0.54	1.00	1.17	0.02	0.44	1.00
arabinose	Carbohydrate	Pentose Metabolism	0.63	0.00	0.04	1.00	0.65	0.00	0.03	0.41	0.80	0.03	0.35	1.00	1.03	0.81	0.97	1.00	1.27	0.10	0.52	1.00	1.23	0.08	0.61	1.00
arachidoylcarnitine (C20)*	Lipid	Fatty Acid Metabolism(Acyl Carnitin	0.95	0.18	0.53	1.00	0.94	0.36	0.74	1.00	0.98	0.29	0.81	1.00	1.00	0.78	0.95	1.00	1.03	0.34	0.69	1.00	1.03	0.94	0.99	1.00
behenoylcarnitine (C22)*	Lipid	Fatty Acid Metabolism(Acyl Carnitin	0.92	0.11	0.47	1.00	1.01	0.67	0.88	1.00	0.93	0.04	0.35	1.00	1.10	0.14	0.66	1.00	1.01	0.88	0.96	1.00	0.92	0.05	0.52	1.00
benzoate	Xenobiotics	Benzoate Metabolism	0.85	0.07	0.39	1.00	0.89	0.02	0.27	1.00	0.90	0.01	0.16	1.00	1.05	0.75	0.95	1.00	1.06	0.60	0.82	1.00	1.02	0.97	0.99	1.00
benzoylcarnitine*	Xenobiotics	Chemical	0.91	0.46	0.76	1.00	0.93	0.67	0.88	1.00	1.08	0.92	1.00	1.00	1.02	0.89	0.97	1.00	1.19	0.11	0.53	1.00	1.16	0.16	0.66	1.00
caproate (6:0)	Lipid	Medium Chain Fatty Acid	0.67	0.33	0.66	1.00	0.77	0.21	0.61	1.00	0.74	0.90	0.99	1.00	1.14	0.90	0.98	1.00	1.09	0.44	0.74	1.00	0.96	0.39	0.78	1.00
caprylate (8:0)	Lipid	Medium Chain Fatty Acid	0.71	0.00	0.08	1.00	0.77	0.08	0.45	1.00	0.69	0.00	0.06	1.00	1.09	0.33	0.81	1.00	0.97	0.57	0.81	1.00	0.89	0.37	0.78	1.00
carboxyethyl-GABA	Amino Acid	Glutamate Metabolism	0.98	0.89	0.96	1.00	0.93	0.40	0.76	1.00	1.00	0.99	1.00	1.00	0.95	0.50	0.88	1.00	1.03	0.73	0.89	1.00	1.08	0.52	0.83	1.00
carotene diol (3)	Cofactors and	Vitamin A Metabolism	0.90	0.15	0.50	1.00	0.94	0.41	0.77	1.00	0.95	0.80	0.96	1.00	1.05	0.27	0.79	1.00	1.06	0.10	0.52	1.00	1.01	0.89	0.97	1.00
ceramide (d18:1/17:0, d17:1/18:0)*	Lipid	Ceramides	0.96	0.46	0.76	1.00	0.88	0.03	0.27	1.00	0.92	0.14	0.58	1.00	0.92	0.16	0.66	1.00	0.95	0.28	0.63	1.00	1.04	0.61	0.87	1.00
phosphocholine	Lipid	Phospholipid Metabolism	0.95	0.21	0.56	1.00	0.93	0.28	0.67	1.00	0.96	0.33	0.82	1.00	0.99	1.00	1.00	1.00	1.02	0.88	0.96	1.00	1.03	0.87	0.97	1.00
corticosterone	Lipid	Corticosteroids	0.85	0.78	0.93	1.00	0.76	0.63	0.87	1.00	1.10	0.06	0.40	1.00	0.89	0.97	0.99	1.00	1.29	0.00	0.13	1.00	1.45	0.00	0.04	0.30
cortisone	Lipid	Corticosteroids	1.09	0.12	0.47	1.00	1.09	0.10	0.46	1.00	1.16	0.00	0.10	1.00	1.00	0.93	0.98	1.00	1.07	0.04	0.45	1.00	1.07	0.04	0.48	1.00
cysteine s-sulfate	Amino Acid	Methionine, Cysteine, SAM and Tau	0.93	0.55	0.82	1.00	0.83	0.24	0.64	1.00	0.80	0.03	0.35	1.00	0.89	0.36	0.81	1.00	0.85	0.05	0.45	1.00	0.96	0.89	0.97	1.00
cysteine sulfinic acid	Amino Acid	Methionine, Cysteine, SAM and Tau	1.11	0.39	0.71	1.00	0.90	0.33	0.73	1.00	1.06	0.97	1.00	1.00	0.81	0.01	0.23	1.00	0.96	0.58	0.81	1.00	1.19	0.12	0.64	1.00
cysteinylglycine	Amino Acid	Glutathione Metabolism	0.85	0.62	0.85	1.00	0.95	0.98	1.00	1.00	0.97	0.99	1.00	1.00	1.11	0.57	0.90	1.00	1.14	0.27	0.63	1.00	1.03	0.53	0.83	1.00
cytidine	Nucleotide	Pyrimidine Metabolism, Cytidine co	0.88	0.73	0.91	1.00	0.97	0.64	0.87	1.00	1.07	0.81	0.97	1.00	1.10	0.74	0.95	1.00	1.21	0.99	0.99	1.00	1.10	0.94	0.99	1.00
deoxycholate	Lipid	Secondary Bile Acid Metabolism	0.88	0.54	0.82	1.00	0.81	0.29	0.68	1.00	0.73	0.06	0.40	1.00	0.92	0.47	0.88	1.00	0.82	0.90	0.97	1.00	0.90	0.52	0.83	1.00
diacylglycerol (12:0/18:1, 14:0/16:1, 16:0/14:1	Lipid	Diacylglycerol	0.62	0.00	0.08	1.00	0.70	0.14	0.52	1.00	0.73	0.14	0.60	1.00	1.12	0.25	0.76	1.00	1.17	0.08	0.51	1.00	1.04	0.45	0.80	1.00
dihomo-linolenoylcarnitine (C20:3n3 or 6)*	Lipid	Fatty Acid Metabolism(Acyl Carnitin	1.17	0.26	0.60	1.00	1.23	0.11	0.46	1.00	1.03	0.48	0.92	1.00	1.04	0.75	0.95	1.00	0.87	0.18	0.58	1.00	0.84	0.30	0.74	1.00
dihomo-linoleoylcarnitine (C20:2)*	Lipid	Fatty Acid Metabolism(Acyl Carnitin	1.08	0.08	0.39	1.00	1.12	0.02	0.25	1.00	1.09	0.06	0.40	1.00	1.04	0.58	0.90	1.00	1.00	0.92	0.98	1.00	0.97	0.46	0.82	1.00
dihydrocaffeate sulfate (2)	Xenobiotics	Food Component/Plant	0.44	0.01	0.12	1.00	0.46	0.00	0.10	1.00	0.60	0.04	0.37	1.00	1.03	0.39	0.82	1.00	1.35	0.14	0.55	1.00	1.31	0.08	0.61	1.00
docosapentaenoate (n6 DPA; 22:5n6)	Lipid	Polyunsaturated Fatty Acid (n3 and	0.82	0.17	0.52	1.00	0.98	0.94	1.00	1.00	0.88	0.59	0.94	1.00	1.20	0.33	0.81	1.00	1.08	0.63	0.84	1.00	0.90	0.62	0.87	1.00
docosapentaenoylcarnitine (C22:5n3)*	Lipid	Fatty Acid Metabolism(Acyl Carnitin	1.13	0.11	0.47	1.00	1.14	0.05	0.37	1.00	1.12	0.08	0.45	1.00	1.00	0.75	0.95	1.00	0.99	0.98	0.99	1.00	0.99	0.92	0.98	1.00
ectoine	Xenobiotics	Chemical	0.95	0.40	0.72	1.00	1.11	0.51	0.82	1.00	1.36	0.00	0.10	1.00	1.17	0.35	0.81	1.00	1.44	0.10	0.53	1.00	1.23	0.27	0.72	1.00
eicosapentaenoylcholine	Lipid	Fatty Acid Metabolism (Acyl Cholin	0.89	0.22	0.56	1.00	1.14	0.36	0.74	1.00	0.98	0.48	0.92	1.00	1.28	0.01	0.22	1.00	1.11	0.10	0.52	1.00	0.87	0.29	0.73	1.00
eicosenedioate (C20:1-DC)*	Lipid	Fatty Acid, Dicarboxylate	0.84	0.03	0.22	1.00	0.87	0.06	0.41	1.00	0.96	0.89	0.99	1.00	1.03	0.89	0.97	1.00	1.13	0.05	0.45	1.00	1.10	0.39	0.78	1.00
eicosenoylcarnitine (C20:1)*	Lipid	Fatty Acid Metabolism(Acyl Carnitin	1.03	0.64	0.86	1.00	1.05	0.38	0.75	1.00	1.03	0.94	1.00	1.00	1.02	0.99	1.00	1.00	1.00	0.60	0.82	1.00	0.98	0.27	0.72	1.00
epiandrosterone sulfate	Lipid	Androgenic Steroids	1.01	0.81	0.94	1.00	0.97	0.76	0.92	1.00	0.96	0.59	0.94	1.00	0.96	0.93	0.98	1.00	0.95	0.57	0.81	1.00	0.99	0.88	0.97	1.00
ADSGEGDFXAEGGGVR*	Peptide	Fibrinogen Cleavage Peptide	0.90	0.39	0.71	1.00	0.82	0.10	0.46	1.00	0.81	0.19	0.67	1.00	0.92	0.49	0.88	1.00	0.91	0.40	0.72	1.00	0.99	0.89	0.97	1.00
ADpSGEGDFXAEGGGVR*	Peptide	Fibrinogen Cleavage Peptide	1.13	0.13	0.48	1.00	0.95	0.34	0.74	1.00	0.95	0.43	0.89	1.00	0.84	0.02	0.33	1.00	0.85	0.06	0.46	1.00	1.00	0.80	0.94	1.00
Fibrinopeptide B (1-11)	Peptide	Fibrinogen Cleavage Peptide	1.01	0.61	0.85	1.00	0.93	0.26	0.66	1.00	0.99	0.97	1.00	1.00	0.92	0.21	0.72	1.00	0.98	0.78	0.92	1.00	1.07	0.15	0.66	1.00
Fibrinopeptide B (1-9)	Peptide	Fibrinogen Cleavage Peptide	0.88	0.37	0.69	1.00	0.80	0.05	0.37	1.00	0.96	0.68	0.94	1.00	0.90	0.63	0.92	1.00	1.09	0.27	0.63	1.00	1.20	0.06	0.53	1.00
fumarate	Energy	TCA Cycle	1.01	0.84	0.95	1.00	0.93	0.27	0.67	1.00	1.05	0.38	0.85	1.00	0.92	0.07	0.59	1.00	1.05	0.39	0.72	1.00	1.14	0.01	0.37	1.00
galactonate	Carbohydrate	Fructose, Mannose and Galactose M	0.42	0.00	0.02	0.30	0.38	0.00	0.01	0.02	0.44	0.01	0.13	1.00	0.92	0.40	0.83	1.00	1.06	0.79	0.92	1.00	1.16	0.41	0.78	1.00
gamma-glutamyl-2-aminobutyrate	Peptide	Gamma-glutamyl Amino Acid	1.21	0.20	0.55	1.00	1.11	0.63	0.87	1.00	1.20	0.17	0.64	1.00	0.92	0.27	0.78	1.00	0.99	0.98	0.99	1.00	1.08	0.52	0.83	1.00
gamma-glutamylserine	Peptide	Gamma-glutamyl Amino Acid	0.96	0.73	0.91	1.00	0.96	0.47	0.80	1.00	0.99	0.98	1.00	1.00	0.99	0.89	0.97	1.00	1.03	0.93	0.98	1.00	1.04	0.36	0.78	1.00
gamma-glutamyltryptophan	Peptide	Gamma-glutamyl Amino Acid	0.90	0.06	0.36	1.00	0.94	0.28	0.67	1.00	0.96	0.23	0.73	1.00	1.05	0.24	0.76	1.00	1.06	0.24	0.63	1.00	1.01	0.72	0.91	1.00

gamma-glutamyltyrosine	Peptide	Gamma-glutamyl Amino Acid	0.85	0.04	0.29	1.00	0.87	0.05	0.37	1.00	0.85	0.02	0.24	1.00	1.03	0.52	0.88	1.00	1.00	0.39	0.72	1.00	0.97	0.66	0.88	1.00
gentisate	Amino Acid	Tyrosine Metabolism	0.72	0.00	0.08	1.00	0.79	0.00	0.10	1.00	1.01	0.29	0.80	1.00	1.10	0.31	0.81	1.00	1.41	0.07	0.48	1.00	1.27	0.55	0.85	1.00
glucuronide of piperine metabolite $C_{17}H_{21}NO_3$	Xenobiotics	Food Component/Plant	1.24	0.05	0.30	1.00	1.20	0.09	0.45	1.00	1.38	0.09	0.48	1.00	0.97	0.43	0.84	1.00	1.12	0.43	0.73	1.00	1.16	0.38	0.78	1.00
glucuronide of piperine metabolite $C_{17}H_{21}NO_3$	Xenobiotics	Food Component/Plant	1.14	0.20	0.55	1.00	1.17	0.06	0.39	1.00	1.35	0.06	0.40	1.00	1.03	0.60	0.91	1.00	1.19	0.24	0.63	1.00	1.16	0.37	0.78	1.00
glucuronide of piperine metabolite $C_{17}H_{21}NO_3$	Xenobiotics	Food Component/Plant	1.24	0.09	0.44	1.00	1.08	0.42	0.78	1.00	1.39	0.21	0.71	1.00	0.87	0.11	0.62	1.00	1.12	0.47	0.75	1.00	1.29	0.16	0.66	1.00
glutarate (C5-DC)	Lipid	Fatty Acid, Dicarboxylate	0.85	0.27	0.61	1.00	0.95	0.47	0.80	1.00	0.97	0.97	1.00	1.00	1.11	0.33	0.81	1.00	1.14	0.69	0.87	1.00	1.02	0.80	0.94	1.00
glutaroylcarnitine (C5)	Amino Acid	Lysine Metabolism	1.01	0.85	0.95	1.00	0.99	0.54	0.83	1.00	1.04	0.33	0.82	1.00	0.98	0.92	0.98	1.00	1.04	0.38	0.70	1.00	1.06	0.12	0.64	1.00
glycerol 3-phosphate	Lipid	Glycerolipid Metabolism	1.03	0.81	0.94	1.00	0.99	0.98	1.00	1.00	1.10	0.33	0.82	1.00	0.96	0.70	0.93	1.00	1.07	0.27	0.63	1.00	1.12	0.19	0.68	1.00
glycerophosphoethanolamine	Lipid	Phospholipid Metabolism	0.94	0.28	0.62	1.00	0.95	0.49	0.81	1.00	1.04	0.85	0.98	1.00	1.02	0.98	0.99	1.00	1.11	0.08	0.49	1.00	1.09	0.13	0.64	1.00
glycerophosphoinositol*	Lipid	Phospholipid Metabolism	1.00	0.43	0.74	1.00	1.23	0.23	0.61	1.00	1.61	0.53	0.93	1.00	1.23	0.01	0.23	1.00	1.61	0.21	0.61	1.00	1.30	0.79	0.94	1.00
glycine conjugate of $C_{10}H_{14}O_2$ (1)*	Partially Char	Partially Characterized Molecules	1.04	0.78	0.93	1.00	0.96	0.46	0.80	1.00	1.02	0.93	1.00	1.00	0.92	0.37	0.81	1.00	0.98	0.33	0.68	1.00	1.06	0.63	0.87	1.00
glyco-beta-muricholate	Lipid	Primary Bile Acid Metabolism	0.61	0.58	0.83	1.00	0.59	0.29	0.69	1.00	0.51	0.03	0.35	1.00	0.97	0.84	0.97	1.00	0.84	0.29	0.65	1.00	0.86	0.67	0.89	1.00
glycochenodeoxycholate 3-sulfate	Lipid	Primary Bile Acid Metabolism	0.65	0.03	0.21	1.00	0.71	0.08	0.44	1.00	0.92	0.51	0.93	1.00	1.09	0.54	0.89	1.00	1.43	0.05	0.45	1.00	1.31	0.41	0.78	1.00
glycochenodeoxycholate glucuronide (1)	Lipid	Primary Bile Acid Metabolism	0.82	0.05	0.30	1.00	0.76	0.00	0.09	1.00	0.73	0.00	0.07	1.00	0.92	0.27	0.78	1.00	0.89	0.41	0.72	1.00	0.96	0.57	0.86	1.00
glycodeoxycholate	Lipid	Secondary Bile Acid Metabolism	0.74	0.29	0.63	1.00	0.86	0.41	0.77	1.00	0.69	0.09	0.48	1.00	1.17	0.97	0.99	1.00	0.93	0.23	0.61	1.00	0.80	0.17	0.66	1.00
glycodeoxycholate 3-sulfate	Lipid	Secondary Bile Acid Metabolism	0.77	0.11	0.47	1.00	0.71	0.01	0.20	1.00	0.72	0.03	0.33	1.00	0.93	0.57	0.90	1.00	0.93	0.84	0.95	1.00	1.01	0.70	0.90	1.00
glycosyl ceramide (d18:1/23:1, d17:1/24:1)*	Lipid	Hexosylceramides (HCER)	1.06	0.53	0.81	1.00	1.07	0.59	0.86	1.00	1.06	0.58	0.94	1.00	1.01	0.59	0.91	1.00	0.99	0.74	0.90	1.00	0.99	0.66	0.88	1.00
glycosyl-N-(2-hydroxynervonoyl)-sphingosine (d1	Lipid	Hexosylceramides (HCER)	0.98	0.94	0.98	1.00	0.81	0.00	0.08	1.00	0.93	0.68	0.94	1.00	0.82	0.02	0.37	1.00	0.94	0.37	0.70	1.00	1.14	0.20	0.68	1.00
glycosyl-N-behenoyl-sphingadienine (d18:2/22:0	Lipid	Hexosylceramides (HCER)	0.94	0.12	0.47	1.00	0.96	0.51	0.82	1.00	0.96	0.48	0.92	1.00	1.03	0.68	0.93	1.00	1.02	0.93	0.98	1.00	1.00	0.43	0.78	1.00
glycosyl-N-tricosanoyl-sphingadienine (d18:2/23	Lipid	Hexosylceramides (HCER)	1.05	0.47	0.77	1.00	1.04	0.37	0.74	1.00	1.03	0.98	1.00	1.00	0.99	0.73	0.94	1.00	0.98	0.67	0.85	1.00	0.99	0.21	0.68	1.00
glycoursodeoxycholate	Lipid	Secondary Bile Acid Metabolism	0.45	0.01	0.14	1.00	0.67	0.42	0.78	1.00	0.53	0.10	0.50	1.00	1.47	0.33	0.81	1.00	1.16	0.39	0.72	1.00	0.79	0.58	0.86	1.00
guanidinosuccinate	Amino Acid	Guanidino and Acetamido Metaboli	0.97	0.46	0.76	1.00	0.94	0.23	0.62	1.00	0.95	0.39	0.87	1.00	0.96	0.42	0.84	1.00	0.98	0.66	0.85	1.00	1.02	0.58	0.86	1.00
gulonate*	Cofactors an	Ascorbate and Aldarate Metabolism	1.01	0.58	0.83	1.00	0.95	0.33	0.73	1.00	1.04	0.32	0.82	1.00	0.94	0.45	0.85	1.00	1.03	0.39	0.72	1.00	1.09	0.08	0.62	1.00
heneicosapentaenoate (21:5n3)	Lipid	Polyunsaturated Fatty Acid (n3 and	1.04	0.75	0.92	1.00	0.98	0.99	1.00	1.00	0.88	0.68	0.94	1.00	0.95	0.47	0.88	1.00	0.85	0.08	0.49	1.00	0.90	0.20	0.68	1.00
hexanoylglycine (C6)	Lipid	Fatty Acid Metabolism(Acyl Glycine)	0.96	0.44	0.75	1.00	0.97	0.94	1.00	1.00	0.81	0.85	0.98	1.00	1.01	0.54	0.89	1.00	0.84	0.39	0.71	1.00	0.83	0.60	0.86	1.00
homocitrulline	Amino Acid	Urea cycle; Arginine and Proline Met	0.85	0.49	0.77	1.00	0.91	0.17	0.57	1.00	0.92	0.59	0.94	1.00	1.07	0.98	0.99	1.00	1.08	0.73	0.89	1.00	1.01	0.33	0.75	1.00
homovanillate (HVA)	Amino Acid	Tyrosine Metabolism	0.98	0.68	0.89	1.00	0.90	0.33	0.73	1.00	1.04	0.94	1.00	1.00	0.92	0.42	0.84	1.00	1.06	0.29	0.65	1.00	1.15	0.14	0.64	1.00
hydantoin-5-propionate	Amino Acid	Histidine Metabolism	0.84	0.49	0.77	1.00	0.84	0.37	0.74	1.00	0.79	0.16	0.63	1.00	1.00	0.94	0.98	1.00	0.94	0.88	0.96	1.00	0.95	0.95	0.99	1.00
hydroquinone sulfate	Xenobiotics	Drug - Topical Agents	0.78	0.27	0.61	1.00	0.65	0.06	0.41	1.00	0.66	0.06	0.40	1.00	0.84	0.58	0.90	1.00	0.86	0.40	0.72	1.00	1.03	0.62	0.87	1.00
hyocholate	Lipid	Secondary Bile Acid Metabolism	1.02	0.93	0.97	1.00	0.84	0.51	0.82	1.00	0.70	0.54	0.93	1.00	0.83	0.24	0.76	1.00	0.69	0.37	0.70	1.00	0.83	0.21	0.68	1.00
imidazole propionate	Amino Acid	Histidine Metabolism	1.46	0.29	0.63	1.00	1.05	0.66	0.88	1.00	1.27	0.45	0.90	1.00	0.72	0.15	0.66	1.00	0.87	0.73	0.89	1.00	1.21	0.88	0.97	1.00
indole-3-carboxylate	Amino Acid	Tryptophan Metabolism	1.07	0.45	0.76	1.00	1.15	0.08	0.44	1.00	1.17	0.08	0.45	1.00	1.07	0.51	0.88	1.00	1.10	0.14	0.55	1.00	1.02	0.52	0.83	1.00
indoleacetylcarnitine*	Xenobiotics	Chemical	0.78	0.11	0.47	1.00	1.13	0.31	0.71	1.00	0.94	0.06	0.40	1.00	1.44	0.01	0.23	1.00	1.20	0.76	0.92	1.00	0.83	0.03	0.48	1.00
indoleacetylglutamine	Amino Acid	Tryptophan Metabolism	0.89	0.34	0.67	1.00	1.09	0.25	0.65	1.00	1.11	0.67	0.94	1.00	1.23	0.04	0.51	1.00	1.25	0.18	0.58	1.00	1.02	0.29	0.74	1.00
isobutyrylglycine (C4)	Amino Acid	Leucine, Isoleucine and Valine Meta	0.76	0.00	0.04	1.00	0.87	0.22	0.61	1.00	0.89	0.62	0.94	1.00	1.14	0.25	0.76	1.00	1.17	0.04	0.45	1.00	1.02	0.76	0.93	1.00
isocitrate	Energy	TCA Cycle	0.95	0.48	0.77	1.00	0.96	0.55	0.84	1.00	1.00	0.78	0.96	1.00	1.01	0.92	0.98	1.00	1.05	0.51	0.78	1.00	1.04	0.33	0.75	1.00
isoleucylglycine	Peptide	Dipeptide	0.86	0.24	0.59	1.00	0.96	0.95	1.00	1.00	0.92	0.79	0.96	1.00	1.12	0.18	0.72	1.00	1.07	0.29	0.65	1.00	0.96	0.79	0.94	1.00
isoursodeoxycholate	Lipid	Secondary Bile Acid Metabolism	0.67	0.76	0.92	1.00	0.59	0.02	0.24	1.00	0.72	0.06	0.40	1.00	0.88	0.05	0.54	1.00	1.08	0.57	0.81	1.00	1.22	0.28	0.72	1.00
isovalerate (C5)	Amino Acid	Leucine, Isoleucine and Valine Meta	1.10	0.51	0.79	1.00	1.08	0.89	0.98	1.00	1.01	0.87	0.98	1.00	0.98	0.61	0.91	1.00	0.92	0.66	0.85	1.00	0.94	0.88	0.97	1.00
isovalerylglycine	Amino Acid	Leucine, Isoleucine and Valine Meta	0.67	0.00	0.04	1.00	0.77	0.03	0.32	1.00	0.80	0.16	0.63	1.00	1.15	0.15	0.66	1.00	1.18	0.01	0.27	1.00	1.03	0.79	0.94	1.00
lactosyl-N-behenoyl-sphingosine (d18:1/22:0)*	Lipid	Lactosylceramides (LCER)	1.28	0.09	0.43	1.00	0.99	1.00	1.00	1.00	1.20	0.14	0.58	1.00	0.78	0.27	0.79	1.00	0.94	0.74	0.90	1.00	1.21	0.14	0.65	1.00
leucylalanine	Peptide	Dipeptide	0.96	0.73	0.91	1.00	0.93	0.21	0.61	1.00	0.87	0.07	0.43	1.00	0.97	0.53	0.89	1.00	0.90	0.14	0.55	1.00	0.94	0.46	0.82	1.00
linoleoyl ethanolamide	Lipid	Endocannabinoid	1.07	0.44	0.75	1.00	1.04	0.74	0.91	1.00	0.97	0.64	0.94	1.00	0.98	0.57	0.90	1.00	0.91	0.20	0.59	1.00	0.93	0.26	0.71	1.00
linoleoyl-arachidonoyl-glycerol (18:2/20:4) [1]*	Lipid	Diacylglycerol	0.96	0.83	0.95	1.00	0.96	0.76	0.92	1.00	1.04	0.94	1.00	1.00	1.00	0.75	0.95	1.00	1.09	0.26	0.63	1.00	1.09	0.43	0.78	1.00
linoleoyl-docosahexaenoyl-glycerol (18:2/22:6) [	Lipid	Diacylglycerol	1.14	0.47	0.77	1.00	1.18	0.11	0.48	1.00	1.11	0.53	0.93	1.00	1.04	0.89	0.97	1.00	0.97	0.62	0.83	1.00	0.93	0.32	0.75	1.00
lithocholate sulfate (1)	Lipid	Secondary Bile Acid Metabolism	1.07	0.13	0.47	1.00	0.98	0.62	0.87	1.00	0.93	0.97	1.00	1.00	0.92	0.23	0.76	1.00	0.87	0.26	0.63	1.00	0.94	0.79	0.94	1.00
maleate	Lipid	Fatty Acid, Dicarboxylate	0.45	0.00	0.02	0.22	0.58	0.00	0.01	0.11	0.36	0.00	0.05	0.83	1.29	0.73	0.94	1.00	0.80	0.74	0.90	1.00	0.62	0.43	0.78	1.00
malonate	Lipid	Fatty Acid Synthesis	1.14	0.28	0.62	1.00	1.10	0.64	0.87	1.00	1.17	0.30	0.81	1.00	0.96	0.39	0.83	1.00	1.03	0.87	0.96	1.00	1.06	0.23	0.70	1.00
methyl-4-hydroxybenzoate sulfate	Xenobiotics	Benzoate Metabolism	0.91	0.75	0.92	1.00	0.89	0.88	0.97	1.00	1.11	0.72	0.95	1.00	0.98	0.93	0.98	1.00	1.21	0.74	0.90	1.00	1.24	0.84	0.95	1.00
methylmalonate (MMA)	Lipid	Fatty Acid Metabolism (also BCAA M	0.92	0.39	0.71	1.00	0.93	0.19	0.59	1.00	0.96	0.64	0.94	1.00	1.01	0.61	0.91	1.00	1.04	0.70	0.88	1.00	1.03	0.70	0.90	1.00
N-(2-furoyl)glycine	Xenobiotics	Food Component/Plant	0.08	0.00	0.01	0.15	0.20	0.00	0.01	0.08	0.24	0.00	0.06	1.00	2.36	0.75	0.95	1.00	2.81	0.06	0.46	1.00	1.19	0.11	0.64	1.00
N2,N5-diacetylornithine	Amino Acid	Urea cycle; Arginine and Proline Met	0.84	0.17	0.52	1.00	0.91	0.76	0.92	1.00	1.05	0.24	0.75	1.00	1.08	0.24	0.76	1.00	1.25	0.04	0.45	1.00	1.15	0.16	0.66	1.00
N2-acetyl,N6-methyllysine	Amino Acid	Lysine Metabolism	1.08	0.23	0.57	1.00	1.05	0.46	0.80	1.00	1.08	0.23	0.73	1.00	0.97	0.38	0.82	1.00	1.00	0.88	0.96	1.00	1.03	0.33	0.75	1.00
N4-acetylcytidine	Nucleotide	Pyrimidine Metabolism, Cytidine co	1.07	0.50	0.78	1.00	1.07	0.66	0.88	1.00	1.13	0.29	0.80	1.00	1.00	0.51	0.88	1.00	1.05	0.54	0.80	1.00	1.05	0.68	0.89	1.00

N6-succinyladenosine	Nucleotide	Purine Metabolism, Adenine contai	1.01	0.89	0.96	1.00	0.99	0.68	0.89	1.00	1.02	0.76	0.95	1.00	0.97	0.51	0.88	1.00	1.01	0.85	0.95	1.00	1.04	0.47	0.82	1.00
N-acetyl-1-methylhistidine*	Amino Acid	Histidine Metabolism	1.13	0.32	0.65	1.00	1.07	0.64	0.87	1.00	1.13	0.27	0.78	1.00	0.95	0.49	0.88	1.00	1.00	0.67	0.85	1.00	1.05	0.45	0.80	1.00
N-acetylaspartate (NAA)	Amino Acid	Alanine and Aspartate Metabolism	0.97	0.87	0.96	1.00	0.93	0.81	0.95	1.00	0.98	1.00	1.00	1.00	0.96	0.52	0.88	1.00	1.01	0.87	0.96	1.00	1.05	0.34	0.77	1.00
N-acetylcarnosine	Amino Acid	Histidine Metabolism	1.01	0.97	0.98	1.00	1.03	0.75	0.92	1.00	1.07	0.47	0.92	1.00	1.03	0.89	0.97	1.00	1.07	0.42	0.73	1.00	1.04	0.37	0.78	1.00
N-acetylcitrulline	Amino Acid	Urea cycle; Arginine and Proline Met	0.77	0.02	0.20	1.00	0.85	0.14	0.54	1.00	0.83	0.28	0.79	1.00	1.11	0.13	0.66	1.00	1.08	0.28	0.63	1.00	0.97	0.95	0.99	1.00
N-acetylglucosamine/N-acetylgalactosamine	Carbohydrate	Aminosugar Metabolism	1.04	0.72	0.91	1.00	1.04	0.97	1.00	1.00	1.07	0.54	0.93	1.00	1.00	0.63	0.92	1.00	1.03	0.60	0.82	1.00	1.03	0.49	0.82	1.00
N-acetylhistidine	Amino Acid	Histidine Metabolism	1.18	0.18	0.53	1.00	1.17	0.45	0.80	1.00	1.11	0.66	0.94	1.00	0.99	0.94	0.98	1.00	0.94	0.58	0.81	1.00	0.95	0.42	0.78	1.00
N-acetylisoleucine	Amino Acid	Leucine, Isoleucine and Valine Meta	0.89	0.11	0.47	1.00	0.88	0.08	0.44	1.00	0.90	0.13	0.58	1.00	0.99	0.61	0.91	1.00	1.01	0.94	0.98	1.00	1.02	0.79	0.94	1.00
N-acetylneuraminate	Carbohydrate	Aminosugar Metabolism	0.95	0.61	0.85	1.00	0.93	0.21	0.61	1.00	0.95	0.98	1.00	1.00	0.97	0.51	0.88	1.00	1.00	0.97	0.99	1.00	1.03	0.39	0.78	1.00
N-acetylphenylalanine	Amino Acid	Phenylalanine Metabolism	0.84	0.05	0.29	1.00	0.89	0.18	0.58	1.00	0.93	0.30	0.81	1.00	1.06	0.40	0.83	1.00	1.11	0.02	0.38	1.00	1.05	0.19	0.68	1.00
N-acetyltyrosine	Amino Acid	Tyrosine Metabolism	0.90	0.29	0.63	1.00	0.92	0.40	0.76	1.00	0.94	0.61	0.94	1.00	1.02	0.69	0.93	1.00	1.04	0.40	0.72	1.00	1.02	0.50	0.82	1.00
N-carbamoylvaline	Amino Acid	Leucine, Isoleucine and Valine Meta	0.96	0.33	0.66	1.00	0.99	0.61	0.86	1.00	0.90	0.10	0.51	1.00	1.03	0.90	0.98	1.00	0.94	0.45	0.74	1.00	0.91	0.40	0.78	1.00
nervonoylcarnitine (C24:1)*	Lipid	Fatty Acid Metabolism(Acyl Carnitin	0.93	0.32	0.65	1.00	0.96	0.98	1.00	1.00	0.95	0.33	0.83	1.00	1.03	0.37	0.81	1.00	1.02	0.93	0.98	1.00	0.99	0.43	0.78	1.00
N-formylanthranilic acid	Amino Acid	Tryptophan Metabolism	0.88	0.26	0.60	1.00	0.72	0.00	0.10	1.00	0.82	0.02	0.24	1.00	0.82	0.02	0.33	1.00	0.93	0.50	0.77	1.00	1.14	0.21	0.68	1.00
nicotinamide	Cofactors and	Nicotinate and Nicotinamide Metab	1.23	0.02	0.18	1.00	1.08	0.09	0.45	1.00	1.24	0.03	0.35	1.00	0.88	0.09	0.62	1.00	1.01	0.70	0.88	1.00	1.14	0.34	0.77	1.00
nisinate (24:6n3)	Lipid	Polyunsaturated Fatty Acid (n3 and	0.86	0.21	0.56	1.00	1.13	0.55	0.84	1.00	0.90	0.39	0.86	1.00	1.32	0.03	0.46	1.00	1.04	0.24	0.63	1.00	0.79	0.44	0.80	1.00
N-palmitoylserine	Lipid	Endocannabinoid	0.87	0.21	0.55	1.00	0.80	0.21	0.61	1.00	0.89	0.18	0.66	1.00	0.92	0.64	0.92	1.00	1.02	0.46	0.75	1.00	1.11	0.68	0.89	1.00
N-stearoylserine*	Lipid	Endocannabinoid	0.91	0.50	0.78	1.00	0.95	0.68	0.89	1.00	0.99	0.76	0.95	1.00	1.04	0.67	0.92	1.00	1.08	0.27	0.63	1.00	1.04	0.57	0.86	1.00
N-stearoyltaurine	Lipid	Endocannabinoid	0.87	0.36	0.68	1.00	0.84	0.17	0.57	1.00	0.93	0.54	0.93	1.00	0.96	0.57	0.90	1.00	1.06	0.89	0.96	1.00	1.10	0.27	0.72	1.00
o-cresol sulfate	Xenobiotics	Benzoate Metabolism	0.66	0.02	0.15	1.00	0.47	0.00	0.01	0.16	0.61	0.01	0.13	1.00	0.72	0.11	0.62	1.00	0.92	0.66	0.85	1.00	1.29	0.16	0.66	1.00
oleoyl-arachidonoyl-glycerol (18:1/20:4) [1]*	Lipid	Diacylglycerol	0.96	0.62	0.85	1.00	1.00	0.84	0.96	1.00	0.99	0.84	0.98	1.00	1.04	0.94	0.98	1.00	1.04	0.61	0.83	1.00	1.00	0.83	0.95	1.00
oleoyl-arachidonoyl-glycerol (18:1/20:4) [2]*	Lipid	Diacylglycerol	0.93	0.51	0.79	1.00	0.93	0.50	0.81	1.00	0.95	0.46	0.92	1.00	0.99	0.93	0.98	1.00	1.02	0.78	0.92	1.00	1.02	0.92	0.98	1.00
oleoyl-linolenoyl-glycerol (18:1/18:3) [2]*	Lipid	Diacylglycerol	0.96	0.60	0.84	1.00	0.93	0.88	0.97	1.00	1.07	0.36	0.84	1.00	0.96	0.79	0.96	1.00	1.11	0.36	0.70	1.00	1.16	0.32	0.75	1.00
palmitoleoyl-oleoyl-glycerol (16:1/18:1) [2]*	Lipid	Diacylglycerol	1.53	0.16	0.52	1.00	1.01	0.95	1.00	1.00	1.64	0.11	0.52	1.00	0.66	0.13	0.66	1.00	1.08	0.84	0.95	1.00	1.63	0.16	0.66	1.00
palmitoyl-arachidonoyl-glycerol (16:0/20:4) [1]*	Lipid	Diacylglycerol	0.89	0.68	0.89	1.00	1.10	0.22	0.61	1.00	1.05	0.67	0.94	1.00	1.24	0.11	0.64	1.00	1.18	0.16	0.56	1.00	0.95	0.49	0.82	1.00
palmitoyl-arachidonoyl-glycerol (16:0/20:4) [2]*	Lipid	Diacylglycerol	0.83	0.15	0.50	1.00	0.91	0.73	0.90	1.00	0.83	0.03	0.35	1.00	1.09	0.15	0.66	1.00	0.99	0.97	0.99	1.00	0.91	0.24	0.71	1.00
palmitoyl-linolenoyl-glycerol (16:0/18:3) [2]*	Lipid	Diacylglycerol	0.76	0.01	0.09	1.00	0.76	0.04	0.35	1.00	0.77	0.05	0.39	1.00	1.00	0.99	1.00	1.00	1.02	0.84	0.95	1.00	1.02	0.78	0.94	1.00
palmitoyl-linoleoyl-glycerol (16:0/18:2) [1]*	Lipid	Diacylglycerol	0.74	0.13	0.48	1.00	0.94	0.95	1.00	1.00	0.90	0.69	0.94	1.00	1.28	0.20	0.72	1.00	1.23	0.34	0.69	1.00	0.96	0.95	0.99	1.00
palmitoyl-linoleoyl-glycerol (16:0/18:2) [2]*	Lipid	Diacylglycerol	0.77	0.18	0.53	1.00	0.91	0.88	0.97	1.00	0.71	0.05	0.39	1.00	1.18	0.06	0.59	1.00	0.93	0.37	0.70	1.00	0.79	0.03	0.47	1.00
palmitoyl-myristoyl-glycerol (16:0/14:0) [2]	Lipid	Diacylglycerol	0.56	0.00	0.02	0.21	0.62	0.01	0.17	1.00	0.64	0.02	0.27	1.00	1.11	0.29	0.80	1.00	1.15	0.16	0.56	1.00	1.03	0.70	0.90	1.00
paraxanthine	Xenobiotics	Xanthine Metabolism	0.76	0.01	0.14	1.00	0.86	0.01	0.20	1.00	0.89	0.20	0.69	1.00	1.14	0.27	0.79	1.00	1.17	0.07	0.48	1.00	1.03	0.49	0.82	1.00
p-cresol glucuronide*	Amino Acid	Tyrosine Metabolism	1.16	0.33	0.66	1.00	1.03	0.68	0.89	1.00	0.87	0.78	0.96	1.00	0.89	0.76	0.95	1.00	0.75	0.90	0.97	1.00	0.85	0.30	0.74	1.00
phenylacetate	Amino Acid	Phenylalanine Metabolism	0.88	0.29	0.63	1.00	0.86	0.23	0.62	1.00	0.80	0.16	0.63	1.00	0.98	0.88	0.97	1.00	0.91	0.55	0.80	1.00	0.93	0.58	0.86	1.00
phenylacetylcarnitine	Peptide	Acetylated Peptides	0.83	0.15	0.50	1.00	0.89	0.58	0.86	1.00	0.80	0.20	0.69	1.00	1.08	0.49	0.88	1.00	0.97	0.85	0.95	1.00	0.90	0.51	0.83	1.00
phenylacetylglutamate	Peptide	Acetylated Peptides	1.00	0.84	0.95	1.00	1.08	0.09	0.45	1.00	1.04	0.88	0.99	1.00	1.09	0.62	0.91	1.00	1.04	0.87	0.96	1.00	0.96	0.76	0.93	1.00
phenylalanylglycine	Peptide	Dipeptide	1.06	0.80	0.94	1.00	0.93	0.49	0.81	1.00	0.98	0.55	0.93	1.00	0.88	0.09	0.62	1.00	0.92	0.21	0.60	1.00	1.06	0.33	0.75	1.00
phosphate	Energy	Oxidative Phosphorylation	1.07	0.42	0.73	1.00	1.32	0.00	0.05	1.00	1.26	0.00	0.10	1.00	1.23	0.00	0.08	0.68	1.18	0.01	0.27	1.00	0.96	0.41	0.78	1.00
picolinate	Amino Acid	Tryptophan Metabolism	0.75	0.01	0.12	1.00	0.79	0.01	0.20	1.00	0.84	0.02	0.30	1.00	1.05	0.94	0.98	1.00	1.11	0.64	0.85	1.00	1.06	0.67	0.89	1.00
piperine	Xenobiotics	Food Component/Plant	1.22	0.11	0.47	1.00	1.05	0.51	0.82	1.00	1.60	0.04	0.35	1.00	0.86	0.29	0.80	1.00	1.31	0.38	0.70	1.00	1.52	0.11	0.64	1.00
pregnanediol-3-glucuronide	Lipid	Progestin Steroids	1.07	0.17	0.52	1.00	0.97	0.93	0.99	1.00	1.07	0.39	0.86	1.00	0.90	0.09	0.62	1.00	0.99	0.74	0.90	1.00	1.10	0.05	0.52	1.00
pregnenolone sulfate	Lipid	Pregnenolone Steroids	1.01	0.98	0.99	1.00	0.93	0.68	0.89	1.00	1.03	0.58	0.94	1.00	0.93	0.42	0.84	1.00	1.02	0.33	0.68	1.00	1.10	0.11	0.64	1.00
pyrraline	Xenobiotics	Food Component/Plant	0.77	0.09	0.42	1.00	0.74	0.03	0.27	1.00	0.80	0.20	0.68	1.00	0.96	0.64	0.92	1.00	1.03	0.57	0.81	1.00	1.08	0.24	0.70	1.00
ribulonate/xylulonate*	Carbohydrate	Pentose Metabolism	0.98	0.78	0.93	1.00	0.85	0.35	0.74	1.00	1.01	0.33	0.83	1.00	0.87	0.03	0.45	1.00	1.03	0.41	0.72	1.00	1.19	0.00	0.05	0.55
S-1-pyrroline-5-carboxylate	Amino Acid	Glutamate Metabolism	0.89	0.39	0.71	1.00	0.88	0.29	0.68	1.00	0.98	0.78	0.96	1.00	0.99	0.98	0.99	1.00	1.10	0.20	0.59	1.00	1.11	0.18	0.67	1.00
S-adenosylhomocysteine (SAH)	Amino Acid	Methionine, Cysteine, SAM and Tau	0.95	0.90	0.96	1.00	0.90	0.27	0.66	1.00	1.03	0.53	0.93	1.00	0.94	0.36	0.81	1.00	1.09	0.43	0.73	1.00	1.15	0.16	0.66	1.00
sarcosine	Amino Acid	Glycine, Serine and Threonine Metab	0.89	0.11	0.47	1.00	0.84	0.05	0.37	1.00	0.98	0.60	0.94	1.00	0.94	0.32	0.81	1.00	1.10	0.13	0.54	1.00	1.16	0.06	0.53	1.00
sphinganine	Lipid	Sphingolipid Synthesis	0.90	0.26	0.60	1.00	0.96	0.93	0.99	1.00	0.92	0.31	0.81	1.00	1.06	0.14	0.66	1.00	1.02	0.68	0.86	1.00	0.96	0.50	0.82	1.00
sphingomyelin (d18:1/25:0, d19:0/24:1, d20:1	Lipid	Sphingomyelins	0.94	0.33	0.66	1.00	0.97	0.97	1.00	1.00	0.93	0.23	0.73	1.00	1.03	0.25	0.76	1.00	0.99	0.94	0.98	1.00	0.96	0.28	0.72	1.00
stachydrine	Xenobiotics	Food Component/Plant	0.75	0.41	0.73	1.00	0.95	0.89	0.98	1.00	0.92	0.60	0.94	1.00	1.27	0.01	0.22	1.00	1.22	0.27	0.63	1.00	0.97	0.70	0.90	1.00
stearoyl-arachidonoyl-glycerol (18:0/20:4) [2]*	Lipid	Diacylglycerol	1.07	0.72	0.91	1.00	1.07	0.63	0.87	1.00	0.99	0.68	0.94	1.00	1.00	0.68	0.93	1.00	0.92	0.35	0.69	1.00	0.93	0.39	0.78	1.00
stearoylcholine*	Lipid	Fatty Acid Metabolism (Acyl Cholin	0.89	0.15	0.50	1.00	1.15	0.35	0.74	1.00	1.02	0.97	1.00	1.00	1.29	0.00	0.05	0.35	1.15	0.02	0.38	1.00	0.89	0.38	0.78	1.00
suberate (C8-DC)	Lipid	Fatty Acid, Dicarboxylate	0.91	0.38	0.70	1.00	0.75	0.09	0.45	1.00	0.81	0.17	0.65	1.00	0.83	0.14	0.66	1.00	0.89	0.41	0.72	1.00	1.08	0.83	0.95	1.00
suberoylcarnitine (C8-DC)	Lipid	Fatty Acid Metabolism(Acyl Carnitin	0.87	0.54	0.82	1.00	0.79	0.63	0.87	1.00	0.81	0.31	0.81	1.00	0.92	0.64	0.92	1.00	0.93	0.60	0.82	1.00	1.02	0.88	0.97	1.00
succinylcarnitine (C4)	Energy	TCA Cycle	0.90	0.04	0.26	1.00	0.91	0.08	0.44	1.00	0.99	0.94	1.00	1.00	1.01	0.75	0.95	1.00	1.10	0.05	0.45	1.00	1.09	0.09	0.64	1.00

sucrose	Carbohydrate	Disaccharides and Oligosaccharides	0.58	0.01	0.12	1.00	0.83	0.11	0.46	1.00	0.81	0.27	0.78	1.00	1.43	0.59	0.91	1.00	1.39	0.05	0.45	1.00	0.97	0.26	0.71	1.00
sulfate of piperine metabolite C16H19NO3 (2)*	Xenobiotics	Food Component/Plant	1.18	0.12	0.47	1.00	1.18	0.11	0.48	1.00	1.40	0.04	0.35	1.00	1.00	0.84	0.97	1.00	1.18	0.33	0.68	1.00	1.19	0.32	0.75	1.00
sulfate of piperine metabolite C16H19NO3 (3)*	Xenobiotics	Food Component/Plant	1.13	0.18	0.53	1.00	1.09	0.33	0.73	1.00	1.42	0.11	0.52	1.00	0.97	0.52	0.88	1.00	1.26	0.23	0.61	1.00	1.30	0.17	0.66	1.00
tartarate	Xenobiotics	Food Component/Plant	0.62	0.31	0.64	1.00	0.59	0.31	0.71	1.00	0.92	0.67	0.94	1.00	0.95	0.69	0.93	1.00	1.47	0.14	0.55	1.00	1.55	0.15	0.66	1.00
taurodeoxycholate	Lipid	Secondary Bile Acid Metabolism	0.73	0.21	0.56	1.00	1.02	0.78	0.93	1.00	0.64	0.04	0.37	1.00	1.39	0.39	0.83	1.00	0.87	0.24	0.63	1.00	0.63	0.10	0.64	1.00
taurolithocholate 3-sulfate	Lipid	Secondary Bile Acid Metabolism	0.71	0.03	0.22	1.00	0.71	0.00	0.13	1.00	0.62	0.00	0.01	0.05	1.00	0.95	0.99	1.00	0.87	0.19	0.58	1.00	0.87	0.30	0.74	1.00
tetradecadienedioate (C14:2-DC)*	Lipid	Fatty Acid, Dicarboxylate	1.17	0.02	0.15	1.00	1.05	0.97	1.00	1.00	1.04	0.92	1.00	1.00	0.90	0.02	0.37	1.00	0.89	0.28	0.63	1.00	0.99	0.90	0.97	1.00
threonylphenylalanine	Peptide	Dipeptide	0.96	0.63	0.86	1.00	0.85	0.06	0.40	1.00	1.00	0.73	0.95	1.00	0.88	0.33	0.81	1.00	1.04	0.64	0.85	1.00	1.17	0.02	0.44	1.00
thymol sulfate	Xenobiotics	Food Component/Plant	0.66	0.01	0.08	1.00	1.03	0.31	0.71	1.00	0.69	0.29	0.80	1.00	1.56	0.02	0.37	1.00	1.05	0.17	0.58	1.00	0.68	0.14	0.65	1.00
tiglyl carnitine (C5)	Amino Acid	Leucine, Isoleucine and Valine Meta	0.84	0.00	0.05	1.00	0.91	0.13	0.51	1.00	0.93	0.06	0.39	1.00	1.09	0.11	0.64	1.00	1.11	0.10	0.53	1.00	1.02	0.60	0.86	1.00
trans-urocanate	Amino Acid	Histidine Metabolism	1.18	0.12	0.47	1.00	1.05	0.48	0.81	1.00	0.98	0.69	0.94	1.00	0.89	0.39	0.82	1.00	0.83	0.07	0.48	1.00	0.93	0.61	0.87	1.00
tryptophan betaine	Amino Acid	Tryptophan Metabolism	0.95	0.61	0.85	1.00	0.89	0.73	0.90	1.00	0.78	0.62	0.94	1.00	0.93	0.15	0.66	1.00	0.83	0.31	0.66	1.00	0.88	0.94	0.99	1.00
vanillactate	Amino Acid	Tyrosine Metabolism	0.90	0.23	0.57	1.00	0.81	0.01	0.13	1.00	0.98	0.59	0.94	1.00	0.90	0.04	0.52	1.00	1.09	0.22	0.61	1.00	1.21	0.00	0.03	0.11
xanthosine	Nucleotide	Purine Metabolism, (Hypo)Xanthine	0.88	0.23	0.57	1.00	0.99	0.47	0.80	1.00	0.91	0.49	0.92	1.00	1.12	0.16	0.66	1.00	1.04	0.62	0.83	1.00	0.93	0.66	0.88	1.00
xylose	Carbohydrate	Pentose Metabolism	0.59	0.17	0.52	1.00	0.59	0.13	0.50	1.00	0.79	0.68	0.94	1.00	1.01	0.45	0.85	1.00	1.35	0.17	0.57	1.00	1.33	0.04	0.48	1.00
X - 11299	Unknown	Unknown	0.69	0.20	0.55	1.00	0.60	0.14	0.52	1.00	1.10	0.61	0.94	1.00	0.87	0.76	0.95	1.00	1.60	0.22	0.61	1.00	1.83	0.39	0.78	1.00
X - 11378	Unknown	Unknown	0.88	0.35	0.67	1.00	0.94	0.68	0.89	1.00	0.79	0.04	0.35	1.00	1.07	0.49	0.88	1.00	0.91	0.66	0.85	1.00	0.85	0.46	0.82	1.00
X - 11381	Unknown	Unknown	0.94	0.13	0.47	1.00	0.89	0.02	0.23	1.00	0.89	0.00	0.07	1.00	0.95	0.13	0.66	1.00	0.95	0.24	0.63	1.00	1.00	0.70	0.90	1.00
X - 11407	Unknown	Unknown	0.99	0.93	0.97	1.00	1.11	0.49	0.81	1.00	1.38	0.06	0.39	1.00	1.11	0.69	0.93	1.00	1.39	0.07	0.49	1.00	1.25	0.13	0.64	1.00
X - 11441	Unknown	Unknown	1.11	0.80	0.94	1.00	0.79	0.17	0.57	1.00	0.99	0.95	1.00	1.00	0.71	0.02	0.37	1.00	0.89	0.48	0.76	1.00	1.24	0.01	0.44	1.00
X - 11442	Unknown	Unknown	1.09	0.63	0.86	1.00	0.81	0.22	0.61	1.00	1.00	0.93	1.00	1.00	0.74	0.04	0.52	1.00	0.91	0.53	0.79	1.00	1.23	0.01	0.44	1.00
X - 11478	Unknown	Unknown	1.14	0.60	0.84	1.00	0.90	0.29	0.69	1.00	1.14	0.26	0.77	1.00	0.79	0.01	0.32	1.00	1.00	0.66	0.85	1.00	1.27	0.04	0.48	1.00
X - 11483	Unknown	Unknown	0.76	0.22	0.56	1.00	0.68	0.20	0.60	1.00	1.25	0.62	0.94	1.00	0.89	0.85	0.97	1.00	1.64	0.27	0.63	1.00	1.84	0.37	0.78	1.00
X - 11491	Unknown	Unknown	1.19	0.52	0.80	1.00	1.20	0.67	0.88	1.00	1.28	0.24	0.75	1.00	1.01	0.49	0.88	1.00	1.08	0.41	0.72	1.00	1.06	0.23	0.69	1.00
X - 11522	Unknown	Unknown	1.00	0.35	0.67	1.00	0.89	0.79	0.94	1.00	0.96	0.94	1.00	1.00	0.89	0.31	0.81	1.00	0.96	0.42	0.73	1.00	1.07	0.45	0.80	1.00
X - 11843	Unknown	Unknown	1.15	0.99	1.00	1.00	1.22	0.10	0.46	1.00	1.06	0.46	0.92	1.00	1.06	0.39	0.82	1.00	0.92	0.51	0.78	1.00	0.87	0.49	0.82	1.00
X - 11850	Unknown	Unknown	1.18	0.36	0.68	1.00	1.07	0.39	0.76	1.00	1.04	0.62	0.94	1.00	0.91	0.95	0.99	1.00	0.88	0.89	0.96	1.00	0.97	0.88	0.97	1.00
X - 11852	Unknown	Unknown	0.81	0.61	0.85	1.00	0.74	0.98	1.00	1.00	0.92	0.87	0.98	1.00	0.92	0.41	0.83	1.00	1.14	0.81	0.94	1.00	1.24	0.80	0.94	1.00
X - 11880	Unknown	Unknown	0.82	0.01	0.12	1.00	0.89	0.09	0.45	1.00	0.95	0.64	0.94	1.00	1.09	0.60	0.91	1.00	1.16	0.14	0.55	1.00	1.07	0.52	0.83	1.00
X - 12007	Unknown	Unknown	0.37	0.00	0.04	1.00	0.52	0.01	0.19	1.00	0.43	0.01	0.20	1.00	1.43	0.63	0.92	1.00	1.16	0.98	0.99	1.00	0.81	0.69	0.90	1.00
X - 12013	Unknown	Unknown	1.16	0.35	0.67	1.00	1.15	0.15	0.54	1.00	1.04	0.41	0.89	1.00	0.99	0.72	0.94	1.00	0.90	0.75	0.91	1.00	0.91	0.47	0.82	1.00
X - 12101	Unknown	Unknown	0.92	0.41	0.73	1.00	0.86	0.14	0.53	1.00	0.86	0.06	0.40	1.00	0.93	0.24	0.76	1.00	0.93	0.36	0.70	1.00	1.00	0.75	0.92	1.00
X - 12111	Unknown	Unknown	0.65	0.42	0.73	1.00	0.85	0.90	0.98	1.00	1.02	0.94	1.00	1.00	1.31	0.05	0.53	1.00	1.57	0.24	0.63	1.00	1.20	0.83	0.95	1.00
X - 12117	Unknown	Unknown	1.00	0.85	0.95	1.00	1.05	0.67	0.88	1.00	1.13	0.11	0.52	1.00	1.05	0.37	0.81	1.00	1.13	0.07	0.48	1.00	1.07	0.25	0.71	1.00
X - 12126	Unknown	Unknown	1.09	0.55	0.82	1.00	1.16	0.37	0.74	1.00	1.12	0.23	0.73	1.00	1.07	0.50	0.88	1.00	1.03	0.46	0.75	1.00	0.97	0.99	1.00	1.00
X - 12170	Unknown	Unknown	0.67	0.00	0.01	0.09	0.79	0.01	0.17	1.00	0.94	0.08	0.45	1.00	1.18	0.01	0.32	1.00	1.40	0.01	0.27	1.00	1.18	0.97	0.99	1.00
X - 12212	Unknown	Unknown	1.13	0.29	0.63	1.00	1.09	0.60	0.86	1.00	1.47	0.21	0.71	1.00	0.96	0.57	0.90	1.00	1.30	0.04	0.45	1.00	1.35	0.18	0.68	1.00
X - 12283	Unknown	Unknown	1.25	0.14	0.48	1.00	0.93	0.97	1.00	1.00	1.56	0.02	0.27	1.00	0.74	0.10	0.62	1.00	1.24	0.06	0.45	1.00	1.68	0.00	0.15	1.00
X - 12680	Unknown	Unknown	0.78	0.00	0.07	1.00	0.85	0.20	0.60	1.00	0.81	0.07	0.41	1.00	1.09	0.44	0.85	1.00	1.04	0.35	0.69	1.00	0.95	0.60	0.86	1.00
X - 12707	Unknown	Unknown	0.95	0.61	0.85	1.00	0.90	0.09	0.45	1.00	1.01	0.89	0.99	1.00	0.94	0.39	0.82	1.00	1.06	0.40	0.72	1.00	1.12	0.07	0.60	1.00
X - 12718	Unknown	Unknown	1.01	0.99	1.00	1.00	1.04	0.60	0.86	1.00	1.09	0.61	0.94	1.00	1.03	0.62	0.91	1.00	1.08	0.59	0.82	1.00	1.04	0.87	0.97	1.00
X - 12739	Unknown	Unknown	0.99	0.99	1.00	1.00	0.85	0.29	0.68	1.00	0.97	0.64	0.94	1.00	0.86	0.11	0.63	1.00	0.98	0.94	0.98	1.00	1.14	0.22	0.68	1.00
X - 12798	Unknown	Unknown	0.95	0.47	0.77	1.00	0.96	0.55	0.84	1.00	1.01	0.80	0.96	1.00	1.00	0.70	0.93	1.00	1.06	0.12	0.54	1.00	1.06	0.28	0.72	1.00
X - 12812	Unknown	Unknown	0.41	0.00	0.04	1.00	0.55	0.02	0.27	1.00	0.45	0.01	0.14	1.00	1.35	0.46	0.87	1.00	1.09	0.36	0.70	1.00	0.81	0.59	0.86	1.00
X - 12816	Unknown	Unknown	1.07	0.88	0.96	1.00	1.04	0.74	0.91	1.00	1.03	0.63	0.94	1.00	0.97	0.58	0.90	1.00	0.97	0.84	0.95	1.00	0.99	0.68	0.89	1.00
X - 12822	Unknown	Unknown	0.84	0.07	0.39	1.00	0.86	0.15	0.54	1.00	0.83	0.01	0.19	1.00	1.02	0.88	0.97	1.00	0.99	0.55	0.80	1.00	0.97	0.75	0.92	1.00
X - 12847	Unknown	Unknown	0.62	0.26	0.60	1.00	0.58	0.12	0.49	1.00	1.10	0.95	1.00	1.00	0.93	0.95	0.99	1.00	1.78	0.14	0.55	1.00	1.91	0.17	0.66	1.00
X - 12849	Unknown	Unknown	0.39	0.00	0.04	1.00	0.38	0.01	0.16	1.00	0.38	0.05	0.39	1.00	0.98	0.78	0.95	1.00	0.98	0.81	0.94	1.00	1.00	0.37	0.78	1.00
X - 12851	Unknown	Unknown	1.29	0.95	0.98	1.00	0.90	0.20	0.60	1.00	0.98	0.28	0.79	1.00	0.70	0.09	0.62	1.00	0.77	0.08	0.49	1.00	1.10	0.95	0.99	1.00
X - 13684	Unknown	Unknown	1.01	0.83	0.95	1.00	0.87	0.02	0.25	1.00	0.91	0.13	0.58	1.00	0.86	0.01	0.21	1.00	0.90	0.01	0.29	1.00	1.05	0.61	0.87	1.00
X - 13728	Unknown	Unknown	0.80	0.34	0.67	1.00	0.89	0.95	1.00	1.00	1.09	0.48	0.92	1.00	1.12	0.28	0.79	1.00	1.37	0.08	0.49	1.00	1.22	0.23	0.70	1.00
X - 13737	Unknown	Unknown	0.99	0.68	0.89	1.00	0.97	0.51	0.82	1.00	1.09	0.72	0.95	1.00	0.99	0.89	0.97	1.00	1.10	0.14	0.55	1.00	1.12	0.03	0.48	1.00
X - 13835	Unknown	Unknown	1.02	0.93	0.97	1.00	1.20	0.32	0.72	1.00	1.16	0.38	0.85	1.00	1.18	0.32	0.81	1.00	1.14	0.47	0.75	1.00	0.96	0.76	0.93	1.00
X - 13844	Unknown	Unknown	0.75	0.06	0.36	1.00	0.70	0.01	0.17	1.00	0.88	0.17	0.65	1.00	0.93	0.60	0.91	1.00	1.18	0.76	0.92	1.00	1.27	0.23	0.69	1.00

X - 15461	Unknown	Unknown	0.92	0.20	0.55	1.00	0.89	0.18	0.58	1.00	0.85	0.06	0.40	1.00	0.97	0.58	0.90	1.00	0.93	0.89	0.96	1.00	0.96	0.68	0.89	1.00
X - 15666	Unknown	Unknown	0.85	0.48	0.77	1.00	0.88	0.63	0.87	1.00	1.09	0.97	1.00	1.00	1.04	0.33	0.81	1.00	1.29	0.27	0.63	1.00	1.24	0.09	0.63	1.00
X - 15674	Unknown	Unknown	1.00	0.40	0.72	1.00	1.03	0.43	0.78	1.00	0.86	0.08	0.45	1.00	1.03	0.41	0.83	1.00	0.87	0.84	0.95	1.00	0.84	0.23	0.70	1.00
X - 15728	Unknown	Unknown	0.74	0.05	0.30	1.00	0.86	0.37	0.74	1.00	0.73	0.01	0.21	1.00	1.17	0.25	0.76	1.00	0.98	0.92	0.98	1.00	0.84	0.22	0.68	1.00
X - 16397	Unknown	Unknown	1.08	0.25	0.60	1.00	0.91	0.31	0.71	1.00	0.97	0.61	0.94	1.00	0.84	0.02	0.37	1.00	0.90	0.15	0.55	1.00	1.06	0.33	0.75	1.00
X - 16570	Unknown	Unknown	1.18	0.36	0.68	1.00	0.96	0.98	1.00	1.00	1.10	0.47	0.92	1.00	0.82	0.09	0.62	1.00	0.93	0.67	0.85	1.00	1.14	0.19	0.68	1.00
X - 16654	Unknown	Unknown	0.82	0.90	0.96	1.00	0.64	0.26	0.66	1.00	0.58	0.06	0.40	1.00	0.78	0.14	0.66	1.00	0.71	0.06	0.45	1.00	0.91	0.90	0.97	1.00
X - 16946	Unknown	Unknown	1.18	0.07	0.38	1.00	0.97	0.88	0.97	1.00	0.98	0.70	0.94	1.00	0.81	0.09	0.62	1.00	0.83	0.09	0.52	1.00	1.02	0.64	0.88	1.00
X - 16964	Unknown	Unknown	0.91	0.29	0.63	1.00	0.87	0.08	0.44	1.00	0.96	0.74	0.95	1.00	0.95	0.20	0.72	1.00	1.05	0.05	0.45	1.00	1.11	0.02	0.47	1.00
X - 17010	Unknown	Unknown	0.88	0.21	0.55	1.00	0.97	0.40	0.76	1.00	0.86	0.12	0.56	1.00	1.10	0.52	0.88	1.00	0.98	0.98	0.99	1.00	0.89	0.48	0.82	1.00
X - 17145	Unknown	Unknown	0.82	0.18	0.53	1.00	0.95	0.83	0.95	1.00	1.04	0.66	0.94	1.00	1.16	0.11	0.62	1.00	1.27	0.10	0.53	1.00	1.10	0.58	0.86	1.00
X - 17146	Unknown	Unknown	1.13	0.57	0.82	1.00	1.56	0.04	0.33	1.00	0.94	0.74	0.95	1.00	1.39	0.23	0.75	1.00	0.83	0.31	0.66	1.00	0.60	0.01	0.44	1.00
X - 17185	Unknown	Unknown	0.24	0.00	0.01	0.06	0.34	0.00	0.03	0.55	0.43	0.02	0.27	1.00	1.40	0.13	0.66	1.00	1.77	0.01	0.32	1.00	1.26	0.81	0.94	1.00
X - 17325	Unknown	Unknown	0.63	0.08	0.42	1.00	0.70	0.08	0.44	1.00	0.88	0.23	0.73	1.00	1.12	0.69	0.93	1.00	1.40	0.26	0.63	1.00	1.25	0.69	0.90	1.00
X - 17327	Unknown	Unknown	1.12	0.81	0.94	1.00	0.85	0.43	0.78	1.00	0.92	0.46	0.92	1.00	0.76	0.02	0.37	1.00	0.83	0.27	0.63	1.00	1.09	0.97	0.99	1.00
X - 17351	Unknown	Unknown	1.14	0.16	0.52	1.00	0.89	0.74	0.91	1.00	1.36	0.01	0.20	1.00	0.78	0.04	0.52	1.00	1.19	0.07	0.48	1.00	1.52	0.00	0.04	0.30
X - 17354	Unknown	Unknown	1.17	0.16	0.52	1.00	0.87	0.92	0.98	1.00	1.06	0.75	0.95	1.00	0.74	0.58	0.90	1.00	0.90	0.70	0.88	1.00	1.22	0.72	0.91	1.00
X - 17359	Unknown	Unknown	1.15	0.27	0.61	1.00	1.24	0.03	0.32	1.00	1.19	0.06	0.39	1.00	1.08	0.21	0.72	1.00	1.03	0.83	0.95	1.00	0.96	0.29	0.74	1.00
X - 17367	Unknown	Unknown	0.73	0.45	0.76	1.00	0.84	0.36	0.74	1.00	1.05	0.97	1.00	1.00	1.15	0.67	0.92	1.00	1.44	0.26	0.63	1.00	1.25	0.73	0.91	1.00
X - 17438	Unknown	Unknown	1.00	0.88	0.96	1.00	0.89	0.31	0.71	1.00	0.91	0.49	0.92	1.00	0.89	0.88	0.97	1.00	0.91	0.11	0.53	1.00	1.02	0.73	0.91	1.00
X - 17612	Unknown	Unknown	1.21	0.17	0.52	1.00	1.09	0.59	0.86	1.00	0.88	0.20	0.68	1.00	0.90	0.11	0.62	1.00	0.73	0.01	0.36	1.00	0.81	0.25	0.71	1.00
X - 18345	Unknown	Unknown	0.37	0.00	0.04	1.00	0.58	0.20	0.60	1.00	0.54	0.53	0.93	1.00	1.60	0.09	0.62	1.00	1.48	0.03	0.44	1.00	0.93	0.81	0.94	1.00
X - 18886	Unknown	Unknown	0.97	0.98	0.99	1.00	0.92	0.24	0.64	1.00	0.93	0.55	0.93	1.00	0.95	0.45	0.85	1.00	0.96	0.58	0.81	1.00	1.01	0.94	0.99	1.00
X - 18899	Unknown	Unknown	0.86	0.02	0.18	1.00	0.89	0.18	0.58	1.00	0.92	0.37	0.84	1.00	1.04	0.14	0.66	1.00	1.08	0.28	0.63	1.00	1.03	0.49	0.82	1.00
X - 18901	Unknown	Unknown	0.54	0.21	0.56	1.00	0.66	0.92	0.98	1.00	0.42	0.04	0.35	1.00	1.22	0.54	0.89	1.00	0.77	0.94	0.98	1.00	0.63	0.59	0.86	1.00
X - 21364	Unknown	Unknown	0.99	0.93	0.97	1.00	0.93	0.19	0.59	1.00	1.04	0.89	0.99	1.00	0.94	0.20	0.72	1.00	1.05	0.30	0.65	1.00	1.11	0.05	0.52	1.00
X - 21441	Unknown	Unknown	0.96	0.57	0.82	1.00	0.87	0.16	0.55	1.00	1.06	0.52	0.93	1.00	0.91	0.06	0.57	1.00	1.11	0.61	0.83	1.00	1.22	0.11	0.64	1.00
X - 21442	Unknown	Unknown	0.85	0.11	0.47	1.00	0.86	0.23	0.62	1.00	0.76	0.08	0.44	1.00	1.01	0.29	0.79	1.00	0.90	0.30	0.65	1.00	0.89	0.48	0.82	1.00
X - 21448	Unknown	Unknown	1.12	0.22	0.56	1.00	0.91	0.47	0.80	1.00	1.04	0.67	0.94	1.00	0.81	0.13	0.66	1.00	0.93	0.45	0.74	1.00	1.14	0.11	0.64	1.00
X - 21467	Unknown	Unknown	1.09	0.21	0.55	1.00	1.01	0.69	0.89	1.00	1.09	0.30	0.81	1.00	0.92	0.14	0.66	1.00	0.99	0.60	0.82	1.00	1.08	0.27	0.72	1.00
X - 21470	Unknown	Unknown	0.93	0.84	0.95	1.00	0.90	0.90	0.98	1.00	0.99	0.33	0.83	1.00	0.96	0.50	0.88	1.00	1.06	0.99	0.99	1.00	1.11	0.23	0.70	1.00
X - 21471	Unknown	Unknown	0.93	0.27	0.61	1.00	0.87	0.05	0.37	1.00	0.97	0.39	0.86	1.00	0.94	0.31	0.81	1.00	1.04	0.98	0.99	1.00	1.11	0.21	0.68	1.00
X - 21607	Unknown	Unknown	1.08	0.17	0.52	1.00	1.08	0.64	0.87	1.00	1.05	0.23	0.73	1.00	1.00	0.76	0.95	1.00	0.97	0.17	0.57	1.00	0.97	0.73	0.91	1.00
X - 21729	Unknown	Unknown	1.14	0.85	0.95	1.00	1.07	0.46	0.80	1.00	1.07	0.42	0.89	1.00	0.94	0.39	0.82	1.00	0.94	0.49	0.77	1.00	1.00	0.79	0.94	1.00
X - 21752	Unknown	Unknown	0.94	0.79	0.93	1.00	0.99	0.89	0.98	1.00	0.82	0.57	0.94	1.00	1.06	0.67	0.92	1.00	0.87	0.35	0.69	1.00	0.82	0.31	0.75	1.00
X - 21792	Unknown	Unknown	1.16	0.16	0.52	1.00	1.00	0.61	0.86	1.00	1.09	0.32	0.82	1.00	0.86	0.08	0.62	1.00	0.94	0.95	0.99	1.00	1.09	0.17	0.66	1.00
X - 21821	Unknown	Unknown	1.12	0.27	0.61	1.00	1.00	0.92	0.98	1.00	1.50	0.00	0.09	1.00	0.89	0.46	0.87	1.00	1.34	0.01	0.26	1.00	1.50	0.00	0.28	1.00
X - 22508	Unknown	Unknown	1.02	0.85	0.95	1.00	1.06	0.55	0.84	1.00	1.06	0.97	1.00	1.00	1.03	0.68	0.93	1.00	1.04	0.99	0.99	1.00	1.01	0.63	0.87	1.00
X - 22509	Unknown	Unknown	1.14	0.13	0.47	1.00	1.12	0.32	0.72	1.00	0.94	0.72	0.95	1.00	0.98	0.62	0.91	1.00	0.83	0.31	0.66	1.00	0.84	0.89	0.97	1.00
X - 23196	Unknown	Unknown	0.51	0.00	0.01	0.15	0.52	0.00	0.01	0.05	0.50	0.00	0.02	0.19	1.01	0.83	0.97	1.00	0.97	0.88	0.96	1.00	0.96	0.48	0.82	1.00
X - 23276	Unknown	Unknown	0.90	0.17	0.52	1.00	0.97	0.79	0.94	1.00	0.95	0.25	0.76	1.00	1.08	0.29	0.79	1.00	1.06	0.41	0.72	1.00	0.99	0.75	0.92	1.00
X - 23296	Unknown	Unknown	1.38	0.01	0.10	1.00	1.22	0.28	0.67	1.00	1.21	0.13	0.58	1.00	0.88	0.38	0.82	1.00	0.88	0.23	0.61	1.00	0.99	0.84	0.95	1.00
X - 23297	Unknown	Unknown	1.29	0.01	0.10	1.00	1.18	0.07	0.42	1.00	1.14	0.10	0.51	1.00	0.92	0.17	0.68	1.00	0.88	0.10	0.53	1.00	0.96	0.50	0.82	1.00
X - 23587	Unknown	Unknown	0.71	0.05	0.31	1.00	0.88	0.27	0.67	1.00	0.94	0.55	0.93	1.00	1.24	0.88	0.97	1.00	1.32	0.18	0.58	1.00	1.07	0.45	0.80	1.00
X - 23641	Unknown	Unknown	0.93	0.26	0.60	1.00	1.04	0.47	0.80	1.00	1.06	0.52	0.93	1.00	1.13	0.06	0.59	1.00	1.15	0.11	0.53	1.00	1.02	1.00	1.00	1.00
X - 23644	Unknown	Unknown	0.62	0.03	0.21	1.00	0.84	0.66	0.88	1.00	1.14	0.72	0.95	1.00	1.36	0.32	0.81	1.00	1.83	0.29	0.65	1.00	1.35	0.61	0.87	1.00
X - 23659	Unknown	Unknown	1.03	0.68	0.89	1.00	1.03	0.72	0.90	1.00	1.05	0.66	0.94	1.00	0.99	0.83	0.97	1.00	1.02	0.50	0.77	1.00	1.02	0.70	0.90	1.00
X - 23665	Unknown	Unknown	0.74	0.05	0.29	1.00	0.79	0.41	0.77	1.00	0.71	0.02	0.25	1.00	1.07	0.97	0.99	1.00	0.95	0.57	0.81	1.00	0.89	0.38	0.78	1.00
X - 23666	Unknown	Unknown	0.95	0.39	0.71	1.00	0.94	0.37	0.74	1.00	0.98	0.80	0.96	1.00	0.99	0.73	0.94	1.00	1.03	0.44	0.74	1.00	1.04	0.32	0.75	1.00
X - 23739	Unknown	Unknown	0.82	0.03	0.21	1.00	0.85	0.08	0.44	1.00	0.97	0.74	0.95	1.00	1.03	0.41	0.83	1.00	1.18	0.00	0.14	1.00	1.14	0.02	0.44	1.00
X - 23782	Unknown	Unknown	1.18	0.14	0.50	1.00	1.08	0.28	0.67	1.00	1.01	0.70	0.94	1.00	0.92	0.42	0.84	1.00	0.86	0.14	0.55	1.00	0.93	0.50	0.82	1.00
X - 23787	Unknown	Unknown	0.96	0.88	0.96	1.00	0.98	0.93	0.99	1.00	1.04	0.50	0.93	1.00	1.03	0.80	0.97	1.00	1.08	0.72	0.88	1.00	1.05	0.95	0.99	1.00
X - 24243	Unknown	Unknown	1.02	0.42	0.73	1.00	1.29	0.38	0.75	1.00	1.02	0.90	0.99	1.00	1.27	0.78	0.95	1.00	0.99	0.45	0.74	1.00	0.79	0.67	0.89	1.00
X - 24293	Unknown	Unknown	0.93	0.59	0.84	1.00	1.06	0.87	0.97	1.00	1.02	0.97	1.00	1.00	1.14	0.39	0.83	1.00	1.09	0.79	0.92	1.00	0.96	0.70	0.90	1.00

X - 24295	Unknown	Unknown	1.10	0.11	0.47	1.00	0.95	0.60	0.86	1.00	0.96	0.98	1.00	1.00	0.86	0.54	0.89	1.00	0.87	0.49	0.77	1.00	1.01	1.00	1.00	1.00
X - 24328	Unknown	Unknown	0.85	0.01	0.13	1.00	0.84	0.04	0.35	1.00	0.92	0.29	0.81	1.00	0.98	0.48	0.88	1.00	1.08	0.39	0.72	1.00	1.10	0.33	0.75	1.00
X - 24337	Unknown	Unknown	0.78	0.03	0.22	1.00	0.85	0.14	0.54	1.00	1.00	0.98	1.00	1.00	1.09	0.10	0.62	1.00	1.29	0.01	0.26	1.00	1.18	0.02	0.44	1.00
X - 24455	Unknown	Unknown	0.69	0.01	0.14	1.00	0.70	0.01	0.16	1.00	0.97	0.24	0.74	1.00	1.01	0.73	0.94	1.00	1.41	0.05	0.45	1.00	1.40	0.02	0.44	1.00
X - 24473	Unknown	Unknown	0.46	0.01	0.13	1.00	0.63	0.08	0.44	1.00	0.76	0.23	0.73	1.00	1.35	0.14	0.66	1.00	1.65	0.03	0.43	1.00	1.22	0.22	0.68	1.00
X - 24475	Unknown	Unknown	1.01	0.89	0.96	1.00	1.16	0.93	0.99	1.00	1.08	0.76	0.95	1.00	1.15	0.64	0.92	1.00	1.07	0.61	0.83	1.00	0.93	0.97	0.99	1.00
X - 24494	Unknown	Unknown	1.07	0.12	0.47	1.00	1.06	0.52	0.82	1.00	1.15	0.02	0.27	1.00	0.98	0.70	0.93	1.00	1.08	0.23	0.61	1.00	1.09	0.09	0.63	1.00
X - 24527	Unknown	Unknown	1.05	0.95	0.98	1.00	0.86	0.40	0.76	1.00	0.99	0.62	0.94	1.00	0.82	0.04	0.50	1.00	0.95	0.68	0.86	1.00	1.16	0.19	0.68	1.00
X - 24542	Unknown	Unknown	0.41	0.00	0.07	1.00	0.44	0.00	0.02	0.28	0.47	0.00	0.10	1.00	1.07	0.69	0.93	1.00	1.14	0.18	0.58	1.00	1.07	0.44	0.80	1.00
X - 24544	Unknown	Unknown	1.00	0.89	0.96	1.00	0.97	0.36	0.74	1.00	1.04	0.62	0.94	1.00	0.97	0.75	0.95	1.00	1.04	0.55	0.80	1.00	1.07	0.22	0.68	1.00
X - 24556	Unknown	Unknown	1.01	0.98	0.99	1.00	0.97	0.50	0.81	1.00	1.05	0.83	0.98	1.00	0.96	0.70	0.93	1.00	1.03	0.66	0.85	1.00	1.08	0.79	0.94	1.00
X - 24571	Unknown	Unknown	1.77	0.83	0.95	1.00	0.98	0.21	0.61	1.00	1.44	0.98	1.00	1.00	0.56	0.70	0.93	1.00	0.82	0.75	0.91	1.00	1.47	0.78	0.94	1.00
X - 24686	Unknown	Unknown	1.04	0.92	0.97	1.00	1.02	0.99	1.00	1.00	0.94	0.26	0.77	1.00	0.98	0.58	0.90	1.00	0.90	0.39	0.72	1.00	0.92	0.88	0.97	1.00
X - 24748	Unknown	Unknown	0.92	0.58	0.83	1.00	0.89	0.64	0.87	1.00	0.94	0.92	1.00	1.00	0.96	0.66	0.92	1.00	1.02	0.60	0.82	1.00	1.06	0.73	0.91	1.00
X - 24757	Unknown	Unknown	0.78	0.49	0.77	1.00	0.83	0.30	0.70	1.00	1.02	0.75	0.95	1.00	1.07	0.89	0.97	1.00	1.31	0.35	0.69	1.00	1.22	0.76	0.93	1.00
X - 24809	Unknown	Unknown	1.12	0.30	0.63	1.00	1.12	0.09	0.45	1.00	1.15	0.11	0.53	1.00	1.00	0.74	0.95	1.00	1.03	0.88	0.96	1.00	1.03	0.39	0.78	1.00
X - 24811	Unknown	Unknown	0.56	0.03	0.22	1.00	0.58	0.01	0.17	1.00	0.73	0.21	0.71	1.00	1.04	0.89	0.97	1.00	1.30	0.09	0.51	1.00	1.25	0.08	0.61	1.00
X - 24849	Unknown	Unknown	1.00	0.42	0.73	1.00	0.91	0.97	1.00	1.00	1.01	0.54	0.93	1.00	0.91	0.32	0.81	1.00	1.01	0.98	0.99	1.00	1.12	0.11	0.64	1.00
X - 24947	Unknown	Unknown	1.03	0.45	0.76	1.00	0.97	0.68	0.89	1.00	1.01	0.88	0.99	1.00	0.95	0.57	0.90	1.00	0.98	0.35	0.69	1.00	1.04	0.52	0.83	1.00
X - 24949	Unknown	Unknown	1.00	0.79	0.93	1.00	0.89	0.28	0.67	1.00	1.07	0.63	0.94	1.00	0.89	0.29	0.79	1.00	1.08	0.98	0.99	1.00	1.20	0.22	0.68	1.00
X - 24972	Unknown	Unknown	0.95	0.58	0.83	1.00	0.94	0.70	0.90	1.00	0.96	0.57	0.94	1.00	0.99	0.76	0.95	1.00	1.01	0.88	0.96	1.00	1.02	0.48	0.82	1.00
X - 25271	Unknown	Unknown	1.33	0.58	0.83	1.00	1.72	0.80	0.94	1.00	1.32	0.61	0.94	1.00	1.30	0.50	0.88	1.00	1.00	0.88	0.96	1.00	0.77	0.63	0.87	1.00
X - 25343	Unknown	Unknown	1.17	0.05	0.30	1.00	1.21	0.15	0.54	1.00	1.04	0.62	0.94	1.00	1.03	0.49	0.88	1.00	0.89	0.34	0.69	1.00	0.86	0.42	0.78	1.00
X - 25419	Unknown	Unknown	0.70	0.04	0.29	1.00	0.75	0.08	0.44	1.00	0.85	0.22	0.72	1.00	1.06	0.24	0.76	1.00	1.21	0.74	0.90	1.00	1.14	0.43	0.78	1.00
X - 25420	Unknown	Unknown	1.27	0.04	0.28	1.00	1.05	0.53	0.83	1.00	1.09	0.52	0.93	1.00	0.83	0.08	0.62	1.00	0.86	0.18	0.58	1.00	1.04	0.48	0.82	1.00
X - 25519	Unknown	Unknown	1.19	0.28	0.62	1.00	1.23	0.27	0.66	1.00	1.14	0.48	0.92	1.00	1.03	0.90	0.98	1.00	0.96	0.74	0.90	1.00	0.93	0.80	0.94	1.00
X - 25520	Unknown	Unknown	0.97	0.85	0.95	1.00	1.04	0.64	0.87	1.00	1.32	0.50	0.93	1.00	1.08	0.41	0.83	1.00	1.37	0.37	0.70	1.00	1.26	0.97	0.99	1.00
