# Supplementary material for: Metabolic Alterations in Older Women With Low Bone Mineral Density Supplemented With Lactobacillus reuteri
Source: JBMR Plus. 2021 Mar 15;5(4):e10478. doi: 10.1002/jbm4.10478 (PMC8046097; doi:10.1002/jbm4.10478)
Supplement: Supplementary file 3 — Table S1(b): The differential metabolites over time in the placebo group, compared by the Wilcoxon signed‐rank test. [file JBM4-5-e10478-s006.pdf]

Supplemental Table 1

			L.reuteri group																							
			3M vs 0M				6M vs 0M				12M vs 0M				6M vs 3M				12M vs 3M				12M vs 6M			
Biochemical Name	Super Pathwa	Sub Pathway	Fold Change	P	Adj. P FDR	Adj. P Bonf.	Fold Change	P	Adj. P FDR	Adj. P Bonf.	Fold Change	P	Adj. P FDR	Adj. P Bonf.	Fold Change	P	Adj. P FDR	Adj. P Bonf.	Fold Change	P	Adj. P FDR	Adj. P Bonf.	Fold Change	P	Adj. P FDR	Adj. P Bonf.
(16 or 17)-methylstearate (a19:0 or i19:0)	Lipid	Fatty Acid, Branched	0.97	0.76	0.95	1.00	1.03	0.43	0.83	1.00	0.96	0.43	0.87	1.00	1.06	0.54	0.85	1.00	0.99	0.87	0.95	1.00	0.93	0.50	0.86	1.00
(N(1) + N(8))-acetylspermidine	Amino Acid	Polyamine Metabolism	1.06	0.09	0.50	1.00	1.05	0.13	0.69	1.00	1.10	0.04	0.54	1.00	0.99	0.46	0.82	1.00	1.04	0.22	0.62	1.00	1.05	0.20	0.73	1.00
1-(1-enyl-palmitoyl)-2-arachidonoyl-GPC (P-16:0	Lipid	Plasmalogen	0.98	0.40	0.79	1.00	0.99	0.64	0.92	1.00	1.05	0.21	0.76	1.00	1.01	0.58	0.87	1.00	1.07	0.02	0.31	1.00	1.06	0.02	0.52	1.00
1-(1-enyl-palmitoyl)-2-arachidonoyl-GPE (P-16:0,	Lipid	Plasmalogen	0.94	0.19	0.64	1.00	1.03	0.98	0.99	1.00	1.05	0.60	0.92	1.00	1.10	0.25	0.70	1.00	1.12	0.02	0.31	1.00	1.02	0.72	0.93	1.00
1-(1-enyl-palmitoyl)-2-linoleoyl-GPC (P-16:0/18:	Lipid	Plasmalogen	0.98	0.50	0.84	1.00	1.01	0.66	0.92	1.00	1.04	0.58	0.91	1.00	1.02	0.94	0.99	1.00	1.06	0.04	0.37	1.00	1.03	0.18	0.73	1.00
1-(1-enyl-palmitoyl)-2-linoleoyl-GPE (P-16:0/18:	Lipid	Plasmalogen	1.00	0.95	0.99	1.00	1.12	0.10	0.65	1.00	1.11	0.10	0.64	1.00	1.12	0.13	0.62	1.00	1.11	0.04	0.37	1.00	0.99	0.57	0.88	1.00
1-(1-enyl-palmitoyl)-2-oleoyl-GPC (P-16:0/18:1)'	Lipid	Plasmalogen	1.02	0.95	0.99	1.00	0.97	0.22	0.77	1.00	1.04	0.46	0.89	1.00	0.95	0.20	0.67	1.00	1.02	0.53	0.82	1.00	1.07	0.02	0.50	1.00
1-(1-enyl-palmitoyl)-2-oleoyl-GPE (P-16:0/18:1)*	Lipid	Plasmalogen	0.95	0.10	0.51	1.00	1.02	0.75	0.94	1.00	1.07	0.25	0.80	1.00	1.07	0.15	0.63	1.00	1.12	0.00	0.24	0.79	1.05	0.63	0.90	1.00
1-(1-enyl-palmitoyl)-2-palmitoleoyl-GPC (P-16:0,	Lipid	Plasmalogen	1.03	0.18	0.62	1.00	1.00	0.90	0.98	1.00	1.07	0.06	0.57	1.00	0.97	0.40	0.79	1.00	1.04	0.15	0.52	1.00	1.07	0.06	0.64	1.00
1-(1-enyl-palmitoyl)-2-palmitoyl-GPC (P-16:0/16	Lipid	Plasmalogen	1.01	0.97	0.99	1.00	0.95	0.10	0.65	1.00	1.03	0.38	0.86	1.00	0.94	0.13	0.61	1.00	1.02	0.26	0.64	1.00	1.09	0.01	0.50	1.00
1-(1-enyl-palmitoyl)-GPC (P-16:0)*	Lipid	Lysoplasmalogen	1.02	0.84	0.95	1.00	0.97	0.84	0.95	1.00	1.06	0.12	0.65	1.00	0.95	0.43	0.79	1.00	1.04	0.13	0.50	1.00	1.09	0.06	0.64	1.00
1-(1-enyl-palmitoyl)-GPE (P-16:0)*	Lipid	Lysoplasmalogen	0.99	0.72	0.95	1.00	1.03	0.54	0.89	1.00	1.06	0.30	0.82	1.00	1.05	0.60	0.87	1.00	1.08	0.06	0.39	1.00	1.03	0.25	0.75	1.00
1-(1-enyl-stearoyl)-2-arachidonoyl-GPE (P-18:0/2	Lipid	Plasmalogen	0.94	0.09	0.50	1.00	0.98	0.33	0.79	1.00	1.04	0.57	0.90	1.00	1.04	0.27	0.70	1.00	1.11	0.02	0.31	1.00	1.06	0.37	0.81	1.00
1-(1-enyl-stearoyl)-2-linoleoyl-GPE (P-18:0/18:2)	Lipid	Plasmalogen	0.97	0.22	0.66	1.00	1.02	0.69	0.92	1.00	1.07	0.26	0.81	1.00	1.05	0.32	0.73	1.00	1.10	0.03	0.35	1.00	1.05	0.67	0.92	1.00
1-(1-enyl-stearoyl)-2-oleoyl-GPE (P-18:0/18:1)	Lipid	Plasmalogen	0.96	0.23	0.66	1.00	1.02	0.70	0.93	1.00	1.07	0.36	0.85	1.00	1.06	0.10	0.61	1.00	1.12	0.01	0.31	1.00	1.05	0.48	0.85	1.00
1-(1-enyl-stearoyl)-GPE (P-18:0)*	Lipid	Lysoplasmalogen	0.99	0.86	0.96	1.00	1.03	0.46	0.85	1.00	1.07	0.20	0.76	1.00	1.04	0.39	0.79	1.00	1.07	0.06	0.39	1.00	1.03	0.45	0.83	1.00
1,2-dilinoleoyl-GPC (18:2/18:2)	Lipid	Phosphatidylcholine (PC)	0.93	0.09	0.50	1.00	0.98	0.84	0.95	1.00	1.02	0.73	0.94	1.00	1.04	0.54	0.85	1.00	1.09	0.05	0.39	1.00	1.05	0.37	0.81	1.00
1,2-dipalmitoyl-GPC (16:0/16:0)	Lipid	Phosphatidylcholine (PC)	0.95	0.05	0.43	1.00	0.93	0.01	0.39	1.00	0.98	0.40	0.86	1.00	0.98	0.84	0.96	1.00	1.03	0.06	0.39	1.00	1.06	0.01	0.50	1.00
1,3-dimethylurate	Xenobiotics	Xanthine Metabolism	0.83	0.13	0.56	1.00	1.04	0.37	0.82	1.00	1.12	0.18	0.76	1.00	1.25	0.01	0.44	1.00	1.35	0.01	0.31	1.00	1.08	0.25	0.75	1.00
1,5-anhydroglucitol (1,5-AG)	Carbohydrate	Glycolysis, Gluconeogenesi	1.00	0.49	0.83	1.00	1.01	0.89	0.97	1.00	1.01	0.76	0.94	1.00	1.01	0.57	0.87	1.00	1.02	0.35	0.71	1.00	1.01	0.82	0.96	1.00
1,7-dimethylurate	Xenobiotics	Xanthine Metabolism	0.83	0.10	0.51	1.00	0.98	0.90	0.98	1.00	1.04	0.67	0.93	1.00	1.18	0.12	0.61	1.00	1.25	0.04	0.37	1.00	1.07	0.44	0.83	1.00
10-heptadecenoate (17:1n7)	Lipid	Long Chain Fatty Acid	1.18	0.05	0.45	1.00	1.09	0.45	0.85	1.00	1.06	0.82	0.96	1.00	0.92	0.10	0.60	1.00	0.90	0.07	0.41	1.00	0.98	0.58	0.88	1.00
10-nonadecenoate (19:1n9)	Lipid	Long Chain Fatty Acid	1.09	0.36	0.76	1.00	1.05	0.23	0.77	1.00	1.01	0.60	0.92	1.00	0.97	0.53	0.84	1.00	0.93	0.29	0.67	1.00	0.96	0.48	0.85	1.00
10-undecenoate (11:1n1)	Lipid	Medium Chain Fatty Acid	1.12	0.04	0.40	1.00	1.06	0.17	0.72	1.00	1.00	0.82	0.96	1.00	0.95	0.36	0.77	1.00	0.90	0.03	0.31	1.00	0.94	0.15	0.73	1.00
13-HODE + 9-HODE	Lipid	Fatty Acid, Monohydroxy	0.92	0.76	0.95	1.00	0.89	0.26	0.77	1.00	0.93	0.44	0.87	1.00	0.97	0.48	0.82	1.00	1.02	0.78	0.93	1.00	1.05	0.53	0.87	1.00
16a-hydroxy DHEA 3-sulfate	Lipid	Androgenic Steroids	0.98	0.84	0.95	1.00	0.91	0.82	0.95	1.00	1.05	0.21	0.76	1.00	0.93	0.70	0.92	1.00	1.07	0.07	0.41	1.00	1.15	0.23	0.75	1.00
16-hydroxypalmitate	Lipid	Fatty Acid, Monohydroxy	1.12	0.01	0.23	1.00	1.09	0.10	0.65	1.00	1.12	0.05	0.57	1.00	0.97	0.46	0.82	1.00	0.99	0.58	0.85	1.00	1.02	0.81	0.96	1.00
1-arachidonoyl-GPC* (20:4)*	Lipid	Lysophospholipid	0.99	0.90	0.98	1.00	0.99	0.76	0.94	1.00	1.05	0.30	0.82	1.00	1.00	0.75	0.93	1.00	1.05	0.09	0.43	1.00	1.06	0.08	0.65	1.00
1-arachidonoyl-GPE (20:4n6)*	Lipid	Lysophospholipid	0.95	0.84	0.95	1.00	0.94	0.95	0.99	1.00	1.00	0.53	0.89	1.00	0.99	0.95	0.99	1.00	1.05	0.07	0.41	1.00	1.06	0.18	0.73	1.00
1-arachidonoyl-GPI* (20:4)*	Lipid	Lysophospholipid	0.87	0.10	0.51	1.00	0.99	0.98	0.99	1.00	0.94	0.37	0.85	1.00	1.13	0.06	0.51	1.00	1.07	0.25	0.64	1.00	0.95	0.13	0.73	1.00
1-arachidonylglycerol (20:4)	Lipid	Monoacylglycerol	0.96	0.53	0.86	1.00	1.04	0.97	0.99	1.00	1.14	0.33	0.83	1.00	1.08	0.43	0.79	1.00	1.19	0.03	0.31	1.00	1.10	0.19	0.73	1.00
1-carboxyethylphenylalanine	Amino Acid	Phenylalanine Metabolism	0.72	0.00	0.20	1.00	0.80	0.08	0.62	1.00	0.75	0.08	0.60	1.00	1.11	0.04	0.51	1.00	1.04	0.09	0.43	1.00	0.94	0.89	0.98	1.00
1-dihomo-linolenylglycerol (20:3)	Lipid	Monoacylglycerol	0.96	0.54	0.87	1.00	1.15	0.49	0.87	1.00	1.06	0.81	0.95	1.00	1.19	0.13	0.61	1.00	1.10	0.50	0.80	1.00	0.92	0.76	0.95	1.00
1-docosahexaenoylglycerol (22:6)	Lipid	Monoacylglycerol	0.98	0.73	0.95	1.00	0.97	0.48	0.86	1.00	1.05	0.57	0.90	1.00	0.99	0.58	0.87	1.00	1.07	0.42	0.74	1.00	1.08	0.24	0.75	1.00
1-linolenoyl-GPC (18:3)*	Lipid	Lysophospholipid	1.00	0.94	0.99	1.00	1.02	0.56	0.89	1.00	1.08	0.64	0.92	1.00	1.03	0.87	0.97	1.00	1.08	0.24	0.63	1.00	1.05	0.58	0.88	1.00
1-linoleoyl-2-linolenoyl-GPC (18:2/18:3)*	Lipid	Phosphatidylcholine (PC)	0.86	0.03	0.36	1.00	0.87	0.18	0.73	1.00	1.00	0.70	0.94	1.00	1.01	0.58	0.87	1.00	1.16	0.09	0.43	1.00	1.15	0.23	0.75	1.00
1-linoleoylglycerol (18:2)	Lipid	Monoacylglycerol	0.91	0.43	0.80	1.00	0.95	0.53	0.89	1.00	1.03	0.61	0.92	1.00	1.05	0.95	0.99	1.00	1.13	0.10	0.46	1.00	1.08	0.07	0.65	1.00
1-linoleoyl-GPC (18:2)	Lipid	Lysophospholipid	0.98	0.52	0.85	1.00	0.99	0.97	0.99	1.00	1.04	0.44	0.87	1.00	1.02	0.40	0.79	1.00	1.07	0.09	0.43	1.00	1.05	0.26	0.75	1.00
1-linoleoyl-GPE (18:2)*	Lipid	Lysophospholipid	0.84	0.24	0.68	1.00	0.84	0.54	0.89	1.00	0.90	0.70	0.94	1.00	1.00	0.63	0.88	1.00	1.08	0.16	0.55	1.00	1.07	0.43	0.83	1.00
1-linoleoyl-GPI* (18:2)*	Lipid	Lysophospholipid	0.81	0.04	0.40	1.00	1.03	0.49	0.87	1.00	0.89	0.26	0.81	1.00	1.27	0.02	0.44	1.00	1.10	0.42	0.74	1.00	0.87	0.02	0.50	1.00
1-methyl-4-imidazoleacetate	Amino Acid	Histidine Metabolism	1.13	0.02	0.30	1.00	1.14	0.02	0.49	1.00	1.05	0.05	0.57	1.00	1.01	0.24	0.68	1.00	0.93	0.45	0.77	1.00	0.92	0.25	0.75	1.00
1-methylhistidine	Amino Acid	Histidine Metabolism	1.05	0.98	1.00	1.00	1.03	0.54	0.89	1.00	1.17	0.18	0.76	1.00	0.97	0.75	0.93	1.00	1.11	0.20	0.60	1.00	1.14	0.16	0.73	1.00
1-methylnicotinamide	Cofactors and	Nicotinate and Nicotinamic	0.44	0.29	0.73	1.00	0.49	0.66	0.92	1.00	0.47	0.92	0.98	1.00	1.10	0.26	0.70	1.00	1.07	0.27	0.64	1.00	0.98	0.70	0.93	1.00
1-myristoyl-2-arachidonoyl-GPC (14:0/20:4)*	Lipid	Phosphatidylcholine (PC)	0.96	0.64	0.93	1.00	0.98	0.98	0.99	1.00	1.03	0.63	0.92	1.00	1.02	0.69	0.91	1.00	1.08	0.31	0.68	1.00	1.06	0.40	0.82	1.00
1-myristoyl-2-palmitoyl-GPC (14:0/16:0)	Lipid	Phosphatidylcholine (PC)	0.98	0.42	0.79	1.00	0.94	0.34	0.80	1.00	1.02	0.81	0.95	1.00	0.96	0.66	0.90	1.00	1.04	0.31	0.68	1.00	1.09	0.11	0.71	1.00
1-myristoylglycerol (14:0)	Lipid	Monoacylglycerol	0.77	0.10	0.51	1.00	0.88	0.40	0.82	1.00	0.89	0.52	0.89	1.00	1.15	0.42	0.79	1.00	1.16	0.08	0.43	1.00	1.01	0.44	0.83	1.00
1-oleoyl-2-docosahexaenoyl-GPC (18:1/22:6)*	Lipid	Phosphatidylcholine (PC)	0.99	0.58	0.90	1.00	0.94	0.11	0.65	1.00	0.95	0.21	0.76	1.00	0.95	0.10	0.61	1.00	0.96	0.21	0.61	1.00	1.01	0.70	0.93	1.00
1-oleoyl-2-linoleoyl-GPE (18:1/18:2)*	Lipid	Phosphatidylethanolamine	0.73	0.23	0.66	1.00	0.75	0.28	0.77	1.00	0.81	0.40	0.86	1.00	1.02	0.64	0.89	1.00	1.10	0.37	0.72	1.00	1.08	0.42	0.82	1.00
1-oleoylglycerol (18:1)	Lipid	Monoacylglycerol	0.91	0.70	0.94	1.00	0.91	0.46	0.85	1.00	0.92	0.52	0.89	1.00	0.99	0.46	0.82	1.00	1.00	0.94	0.98	1.00	1.01	0.24	0.75	1.00

1-oleoyl-GPC (18:1)	Lipid	Lysophospholipid	1.02	0.67	0.93	1.00	1.02	0.63	0.91	1.00	1.06	0.16	0.75	1.00	1.00	0.67	0.91	1.00	1.04	0.21	0.60	1.00	1.04	0.24	0.75	1.00
1-oleoyl-GPE (18:1)	Lipid	Lysophospholipid	0.77	0.15	0.58	1.00	0.81	0.15	0.70	1.00	0.91	0.97	1.00	1.00	1.06	0.32	0.73	1.00	1.19	0.02	0.31	1.00	1.12	0.16	0.73	1.00
1-oleoyl-GPI (18:1)*	Lipid	Lysophospholipid	0.78	0.07	0.49	1.00	1.00	0.60	0.91	1.00	0.90	0.58	0.91	1.00	1.28	0.07	0.55	1.00	1.16	0.15	0.52	1.00	0.90	0.35	0.80	1.00
1-palmitoleoylglycerol (16:1)*	Lipid	Monoacylglycerol	0.90	0.75	0.95	1.00	0.95	0.94	0.98	1.00	0.95	0.87	0.97	1.00	1.05	0.72	0.92	1.00	1.06	0.27	0.64	1.00	1.01	0.22	0.74	1.00
1-palmitoleoyl-GPC* (16:1)*	Lipid	Lysophospholipid	1.02	0.75	0.95	1.00	0.99	0.78	0.94	1.00	1.09	0.28	0.82	1.00	0.97	0.79	0.94	1.00	1.06	0.15	0.52	1.00	1.09	0.10	0.70	1.00
1-palmitoyl-2-arachidonoyl-GPC (16:0/20:4n6)	Lipid	Phosphatidylcholine (PC)	0.96	0.18	0.61	1.00	0.96	0.21	0.75	1.00	1.00	0.76	0.94	1.00	1.00	0.98	0.99	1.00	1.04	0.04	0.37	1.00	1.04	0.02	0.50	1.00
1-palmitoyl-2-arachidonoyl-GPE (16:0/20:4)*	Lipid	Phosphatidylethanolamine	0.99	0.52	0.85	1.00	0.96	0.76	0.94	1.00	0.99	0.64	0.92	1.00	0.97	0.18	0.67	1.00	0.99	0.82	0.94	1.00	1.03	0.61	0.90	1.00
1-palmitoyl-2-arachidonoyl-GPI (16:0/20:4)*	Lipid	Phosphatidylinositol (PI)	0.95	0.84	0.95	1.00	0.88	0.24	0.77	1.00	0.98	0.57	0.90	1.00	0.93	0.13	0.62	1.00	1.03	0.42	0.74	1.00	1.11	0.07	0.64	1.00
1-palmitoyl-2-dihomo-linolenoyl-GPC (16:0/20:	Lipid	Phosphatidylcholine (PC)	0.99	0.82	0.95	1.00	1.01	0.63	0.91	1.00	1.01	0.61	0.92	1.00	1.02	0.22	0.68	1.00	1.02	0.21	0.60	1.00	1.00	0.92	0.98	1.00
1-palmitoyl-2-docosahexaenoyl-GPC (16:0/22:6)	Lipid	Phosphatidylcholine (PC)	0.95	0.12	0.54	1.00	0.91	0.01	0.42	1.00	0.95	0.09	0.60	1.00	0.95	0.13	0.61	1.00	1.00	0.94	0.98	1.00	1.05	0.13	0.73	1.00
1-palmitoyl-2-docosahexaenoyl-GPE (16:0/22:6)	Lipid	Phosphatidylethanolamine	0.99	0.90	0.98	1.00	0.91	0.37	0.82	1.00	0.97	0.49	0.89	1.00	0.92	0.04	0.50	1.00	0.98	0.43	0.75	1.00	1.07	0.16	0.73	1.00
1-palmitoyl-2-linoleoyl-GPC (16:0/18:2)	Lipid	Phosphatidylcholine (PC)	0.97	0.33	0.74	1.00	0.97	0.46	0.85	1.00	1.00	0.90	0.98	1.00	1.00	0.64	0.89	1.00	1.03	0.16	0.54	1.00	1.03	0.18	0.73	1.00
1-palmitoyl-2-linoleoyl-GPE (16:0/18:2)	Lipid	Phosphatidylethanolamine	0.91	1.00	1.00	1.00	0.90	0.94	0.98	1.00	0.94	0.48	0.89	1.00	0.99	0.60	0.87	1.00	1.03	0.86	0.95	1.00	1.04	0.70	0.93	1.00
1-palmitoyl-2-linoleoyl-GPI (16:0/18:2)	Lipid	Phosphatidylinositol (PI)	0.92	0.46	0.82	1.00	0.88	0.15	0.70	1.00	0.96	0.70	0.94	1.00	0.96	0.10	0.61	1.00	1.05	0.79	0.93	1.00	1.10	0.31	0.78	1.00
1-palmitoyl-2-oleoyl-GPC (16:0/18:1)	Lipid	Phosphatidylcholine (PC)	1.01	0.64	0.93	1.00	0.99	0.97	0.99	1.00	1.01	0.40	0.86	1.00	0.98	0.81	0.95	1.00	1.01	0.60	0.86	1.00	1.03	0.24	0.75	1.00
1-palmitoyl-2-oleoyl-GPI (16:0/18:1)*	Lipid	Phosphatidylinositol (PI)	0.92	0.45	0.81	1.00	0.87	0.11	0.65	1.00	0.95	0.57	0.90	1.00	0.95	0.34	0.75	1.00	1.03	0.48	0.78	1.00	1.08	0.11	0.71	1.00
1-palmitoyl-2-palmitoleoyl-GPC (16:0/16:1)*	Lipid	Phosphatidylcholine (PC)	1.06	0.34	0.74	1.00	1.02	0.56	0.89	1.00	1.09	0.21	0.76	1.00	0.96	0.38	0.79	1.00	1.03	0.53	0.82	1.00	1.07	0.21	0.73	1.00
1-palmitoyl-2-stearoyl-GPC (16:0/18:0)	Lipid	Phosphatidylcholine (PC)	0.95	0.10	0.51	1.00	0.91	0.00	0.29	1.00	1.00	0.97	1.00	1.00	0.96	0.49	0.83	1.00	1.06	0.04	0.37	1.00	1.10	0.01	0.50	1.00
1-palmitoyl-GPC (16:0)	Lipid	Lysophospholipid	0.98	0.48	0.82	1.00	0.97	0.27	0.77	1.00	1.04	0.22	0.76	1.00	1.00	0.66	0.90	1.00	1.07	0.01	0.31	1.00	1.07	0.04	0.60	1.00
1-palmitoyl-GPE (16:0)	Lipid	Lysophospholipid	0.99	0.79	0.95	1.00	0.92	0.24	0.77	1.00	1.02	0.50	0.89	1.00	0.93	0.24	0.69	1.00	1.04	0.39	0.73	1.00	1.12	0.06	0.64	1.00
1-palmitoyl-GPI* (16:0)	Lipid	Lysophospholipid	0.78	0.12	0.54	1.00	0.98	0.78	0.94	1.00	0.84	0.28	0.82	1.00	1.25	0.07	0.53	1.00	1.07	0.44	0.76	1.00	0.85	0.09	0.68	1.00
1-ribosyl-imidazoleacetate*	Amino Acid	Histidine Metabolism	1.27	0.02	0.33	1.00	1.33	0.00	0.24	0.95	1.44	0.00	0.24	1.00	1.05	0.22	0.68	1.00	1.14	0.58	0.85	1.00	1.09	0.86	0.97	1.00
1-stearoyl-2-arachidonoyl-GPC (18:0/20:4)	Lipid	Phosphatidylcholine (PC)	0.95	0.18	0.62	1.00	0.95	0.10	0.65	1.00	0.98	0.97	1.00	1.00	0.99	0.79	0.94	1.00	1.03	0.11	0.48	1.00	1.03	0.04	0.57	1.00
1-stearoyl-2-arachidonoyl-GPE (18:0/20:4)	Lipid	Phosphatidylethanolamine	0.94	0.60	0.91	1.00	0.91	0.36	0.82	1.00	0.94	0.30	0.82	1.00	0.97	0.35	0.76	1.00	1.01	0.97	0.99	1.00	1.03	0.73	0.93	1.00
1-stearoyl-2-arachidonoyl-GPI (18:0/20:4)	Lipid	Phosphatidylinositol (PI)	0.95	0.45	0.81	1.00	0.91	0.25	0.77	1.00	0.96	0.84	0.96	1.00	0.96	0.32	0.73	1.00	1.01	0.57	0.85	1.00	1.05	0.24	0.75	1.00
1-stearoyl-2-docosahexaenoyl-GPC (18:0/22:6)	Lipid	Phosphatidylcholine (PC)	0.93	0.24	0.68	1.00	0.88	0.01	0.39	1.00	0.95	0.13	0.69	1.00	0.95	0.11	0.61	1.00	1.02	0.82	0.94	1.00	1.08	0.16	0.73	1.00
1-stearoyl-2-docosahexaenoyl-GPE (18:0/22:6)*	Lipid	Phosphatidylethanolamine	0.92	0.13	0.55	1.00	0.88	0.15	0.70	1.00	0.88	0.06	0.57	1.00	0.96	0.33	0.74	1.00	0.96	0.24	0.63	1.00	1.00	0.97	1.00	1.00
1-stearoyl-2-linoleoyl-GPC (18:0/18:2)*	Lipid	Phosphatidylcholine (PC)	0.95	0.07	0.49	1.00	0.97	0.24	0.77	1.00	0.99	0.78	0.94	1.00	1.01	0.35	0.76	1.00	1.04	0.18	0.57	1.00	1.02	0.36	0.81	1.00
1-stearoyl-2-linoleoyl-GPE (18:0/18:2)*	Lipid	Phosphatidylethanolamine	0.82	0.29	0.73	1.00	0.82	0.37	0.82	1.00	0.85	0.34	0.84	1.00	1.01	0.81	0.95	1.00	1.05	0.58	0.85	1.00	1.04	0.73	0.93	1.00
1-stearoyl-2-linoleoyl-GPI (18:0/18:2)	Lipid	Phosphatidylinositol (PI)	0.90	0.16	0.59	1.00	0.86	0.04	0.51	1.00	0.92	0.58	0.91	1.00	0.95	0.30	0.73	1.00	1.02	0.35	0.71	1.00	1.08	0.17	0.73	1.00
1-stearoyl-2-oleoyl-GPC (18:0/18:1)	Lipid	Phosphatidylcholine (PC)	1.00	0.86	0.96	1.00	0.98	0.58	0.90	1.00	1.02	0.61	0.92	1.00	0.98	0.78	0.94	1.00	1.02	0.38	0.73	1.00	1.04	0.26	0.75	1.00
1-stearoyl-2-oleoyl-GPI (18:0/18:1)*	Lipid	Phosphatidylinositol (PI)	0.87	0.10	0.51	1.00	0.85	0.08	0.62	1.00	0.96	0.79	0.95	1.00	0.98	0.87	0.97	1.00	1.09	0.09	0.43	1.00	1.12	0.19	0.73	1.00
1-stearoyl-GPC (18:0)	Lipid	Lysophospholipid	0.99	0.73	0.95	1.00	0.97	0.26	0.77	1.00	1.02	0.56	0.89	1.00	0.98	0.57	0.87	1.00	1.03	0.39	0.73	1.00	1.05	0.16	0.73	1.00
1-stearoyl-GPE (18:0)	Lipid	Lysophospholipid	0.97	0.67	0.93	1.00	0.92	0.19	0.75	1.00	1.01	0.90	0.98	1.00	0.95	0.33	0.74	1.00	1.04	0.37	0.72	1.00	1.10	0.16	0.73	1.00
1-stearoyl-GPI (18:0)	Lipid	Lysophospholipid	1.03	0.75	0.95	1.00	0.95	0.66	0.92	1.00	1.03	0.53	0.89	1.00	0.93	0.32	0.73	1.00	1.00	0.69	0.88	1.00	1.08	0.28	0.75	1.00
2,3-dihydroxy-5-methylthio-4-pentenoate (DMTP	Amino Acid	Methionine, Cysteine, SAM	1.01	0.64	0.93	1.00	1.03	0.37	0.82	1.00	1.02	0.24	0.79	1.00	1.02	0.34	0.75	1.00	1.02	0.36	0.71	1.00	1.00	0.87	0.97	1.00
2-aminobutyrate	Amino Acid	Glutathione Metabolism	1.01	0.54	0.87	1.00	1.02	0.67	0.92	1.00	1.02	0.66	0.93	1.00	1.00	0.87	0.97	1.00	1.00	0.61	0.87	1.00	1.00	0.73	0.93	1.00
2-aminooctanoate	Lipid	Fatty Acid, Amino	0.84	0.95	0.99	1.00	0.83	0.78	0.94	1.00	0.82	0.43	0.87	1.00	0.99	0.97	0.99	1.00	0.97	0.94	0.98	1.00	0.98	0.81	0.96	1.00
2-aminophenol sulfate	Xenobiotics	Chemical	0.81	0.12	0.55	1.00	0.72	0.07	0.61	1.00	0.86	0.36	0.85	1.00	0.88	0.69	0.91	1.00	1.05	0.63	0.87	1.00	1.19	0.12	0.73	1.00
2-hydroxy-3-methylvalerate	Amino Acid	Leucine, Isoleucine and Val	0.88	0.17	0.60	1.00	0.92	0.17	0.72	1.00	0.94	0.48	0.89	1.00	1.04	0.92	0.99	1.00	1.06	0.32	0.69	1.00	1.03	0.23	0.75	1.00
2-hydroxybutyrate/2-hydroxyisobutyrate	Amino Acid	Glutathione Metabolism	0.90	0.20	0.64	1.00	0.85	0.05	0.54	1.00	0.85	0.02	0.47	1.00	0.95	0.44	0.80	1.00	0.95	0.39	0.73	1.00	1.00	0.69	0.93	1.00
2-hydroxydecanoate	Lipid	Fatty Acid, Monohydroxy	0.82	0.05	0.45	1.00	0.82	0.12	0.67	1.00	0.88	0.26	0.81	1.00	1.00	1.00	1.00	1.00	1.08	0.15	0.52	1.00	1.08	0.27	0.75	1.00
2-hydroxyglutarate	Lipid	Fatty Acid, Dicarboxylate	1.02	0.60	0.91	1.00	1.03	0.27	0.77	1.00	1.05	0.44	0.87	1.00	1.01	0.84	0.96	1.00	1.03	0.45	0.77	1.00	1.03	0.82	0.96	1.00
2-hydroxynervonate*	Lipid	Fatty Acid, Monohydroxy	0.79	0.00	0.07	0.25	0.82	0.00	0.29	1.00	0.83	0.01	0.36	1.00	1.04	0.69	0.91	1.00	1.05	0.84	0.94	1.00	1.01	0.42	0.82	1.00
2-hydroxyoctanoate	Lipid	Fatty Acid, Monohydroxy	0.84	0.06	0.47	1.00	0.67	0.00	0.29	1.00	0.69	0.15	0.73	1.00	0.80	0.36	0.77	1.00	0.83	0.87	0.95	1.00	1.03	0.43	0.83	1.00
2-hydroxypalmitate	Lipid	Fatty Acid, Monohydroxy	0.87	0.04	0.40	1.00	0.93	0.25	0.77	1.00	0.91	0.22	0.76	1.00	1.07	0.38	0.79	1.00	1.04	0.76	0.92	1.00	0.98	0.78	0.95	1.00
2-hydroxystearate	Lipid	Fatty Acid, Monohydroxy	0.81	0.00	0.20	1.00	0.89	0.05	0.54	1.00	0.85	0.03	0.47	1.00	1.09	0.11	0.61	1.00	1.05	0.63	0.87	1.00	0.96	0.57	0.88	1.00
2-methylcitrate/homocitrate	Energy	TCA Cycle	0.98	0.81	0.95	1.00	0.99	0.92	0.98	1.00	1.01	0.84	0.96	1.00	1.01	0.22	0.68	1.00	1.03	0.14	0.52	1.00	1.02	0.84	0.97	1.00
2-oleoylglycerol (18:1)	Lipid	Monoacylglycerol	0.91	0.53	0.86	1.00	0.99	1.00	1.00	1.00	0.93	0.52	0.89	1.00	1.09	0.63	0.88	1.00	1.03	0.94	0.98	1.00	0.95	0.94	0.99	1.00
2-palmitoyl-GPC* (16:0)*	Lipid	Lysophospholipid	0.85	0.10	0.51	1.00	0.93	0.78	0.94	1.00	0.84	0.09	0.60	1.00	1.09	0.19	0.67	1.00	0.98	0.78	0.93	1.00	0.90	0.38	0.82	1.00
2-piperidinone	Xenobiotics	Food Component/Plant	1.04	0.09	0.50	1.00	1.01	0.61	0.91	1.00	1.06	0.29	0.82	1.00	0.98	0.69	0.91	1.00	1.02	0.86	0.95	1.00	1.05	0.45	0.83	1.00
2-pyrrolidinone	Amino Acid	Glutamate Metabolism	0.98	0.72	0.95	1.00	0.97	0.75	0.94	1.00	1.08	0.08	0.58	1.00	0.98	0.73	0.93	1.00	1.10	0.08	0.43	1.00	1.12	0.07	0.65	1.00
2-stearoyl-GPE (18:0)*	Lipid	Lysophospholipid	0.81	0.06	0.47	1.00	0.85	0.20	0.75	1.00	0.86	0.13	0.68	1.00	1.05	0.50	0.84	1.00	1.06	0.08	0.43	1.00	1.01	0.66	0.92	1.00
3-(4-hydroxyphenyl)lactate (HPLA)	Amino Acid	Tyrosine Metabolism	0.94	0.18	0.61	1.00	0.98	0.66	0.92	1.00	1.03	0.53	0.89	1.00	1.04	0.16	0.64	1.00	1.09	0.01	0.31	1.00	1.05	0.23	0.75	1.00

3,4-dihydroxybutyrate	Amino Acid	Glutamate Metabolism	0.88	0.05	0.45	1.00	0.93	0.76	0.94	1.00	0.87	0.05	0.57	1.00	1.06	0.16	0.64	1.00	0.99	0.82	0.94	1.00	0.94	0.17	0.73	1.00
3-aminoisobutyrate	Nucleotide	Pyrimidine Metabolism, Th	1.07	0.07	0.49	1.00	1.04	0.36	0.82	1.00	1.06	0.32	0.83	1.00	0.97	0.52	0.84	1.00	0.99	0.69	0.88	1.00	1.02	0.69	0.93	1.00
3beta,7alpha-dihydroxy-5-cholestenoate	Lipid	Sterol	1.15	0.06	0.47	1.00	1.12	0.11	0.65	1.00	1.12	0.12	0.65	1.00	0.97	0.45	0.81	1.00	0.97	0.40	0.74	1.00	1.00	0.43	0.83	1.00
3beta-hydroxy-5-cholestenoate	Lipid	Sterol	0.88	0.05	0.43	1.00	0.95	0.58	0.90	1.00	0.88	0.06	0.57	1.00	1.07	0.26	0.70	1.00	1.00	0.50	0.80	1.00	0.93	0.07	0.64	1.00
3-carboxy-4-methyl-5-pentyl-2-furanpropionate (	Lipid	Fatty Acid, Dicarboxylate	0.99	0.76	0.95	1.00	1.01	0.98	0.99	1.00	1.05	0.17	0.75	1.00	1.02	0.86	0.97	1.00	1.06	0.09	0.43	1.00	1.04	0.23	0.75	1.00
3-carboxy-4-methyl-5-propyl-2-furanpropanoate (	Lipid	Fatty Acid, Dicarboxylate	0.96	0.76	0.95	1.00	0.86	0.06	0.58	1.00	0.86	0.03	0.47	1.00	0.89	0.29	0.72	1.00	0.90	0.06	0.39	1.00	1.01	0.53	0.87	1.00
3-formylindole	Xenobiotics	Food Component/Plant	1.04	0.37	0.76	1.00	1.03	0.63	0.91	1.00	1.04	0.30	0.82	1.00	0.99	0.53	0.84	1.00	1.00	0.97	0.99	1.00	1.01	0.72	0.93	1.00
3-hydroxy-2-ethylpropionate	Amino Acid	Leucine, Isoleucine and Val	0.99	0.64	0.93	1.00	0.94	0.37	0.82	1.00	0.98	0.76	0.94	1.00	0.95	0.48	0.82	1.00	1.00	0.79	0.93	1.00	1.05	0.13	0.73	1.00
3-hydroxy-3-methylglutarate	Lipid	Mevalonate Metabolism	0.89	0.21	0.66	1.00	0.93	0.58	0.90	1.00	0.88	0.66	0.93	1.00	1.05	0.13	0.61	1.00	0.99	0.31	0.68	1.00	0.95	0.69	0.93	1.00
3-hydroxybutyrate (BHBA)	Lipid	Ketone Bodies	1.22	0.04	0.40	1.00	0.93	0.86	0.95	1.00	0.84	0.64	0.92	1.00	0.76	0.13	0.61	1.00	0.69	0.08	0.43	1.00	0.91	0.36	0.81	1.00
3-hydroxydecanoate	Lipid	Fatty Acid, Monohydroxy	1.05	0.32	0.73	1.00	0.96	0.79	0.94	1.00	0.87	0.78	0.94	1.00	0.92	0.82	0.95	1.00	0.83	0.37	0.72	1.00	0.91	0.29	0.75	1.00
3-hydroxyhexanoate	Lipid	Fatty Acid, Monohydroxy	0.99	0.67	0.93	1.00	0.91	0.18	0.73	1.00	0.87	0.11	0.65	1.00	0.92	0.42	0.79	1.00	0.87	0.40	0.74	1.00	0.95	0.39	0.82	1.00
3-hydroxyhippurate	Xenobiotics	Benzoate Metabolism	0.91	0.84	0.95	1.00	0.79	0.21	0.75	1.00	0.92	0.53	0.89	1.00	0.87	0.22	0.68	1.00	1.01	0.98	1.00	1.00	1.16	0.66	0.92	1.00
3-hydroxylaurate	Lipid	Fatty Acid, Monohydroxy	1.15	0.08	0.50	1.00	1.15	0.18	0.73	1.00	1.11	0.28	0.82	1.00	1.00	0.56	0.86	1.00	0.97	0.81	0.93	1.00	0.97	0.52	0.86	1.00
3-hydroxyoctanoate	Lipid	Fatty Acid, Monohydroxy	0.96	0.27	0.71	1.00	0.80	0.11	0.66	1.00	0.70	0.26	0.81	1.00	0.83	0.64	0.89	1.00	0.73	0.87	0.95	1.00	0.88	0.87	0.97	1.00
3-hydroxyoleate*	Lipid	Fatty Acid, Monohydroxy	1.00	0.82	0.95	1.00	1.01	0.69	0.92	1.00	0.96	0.34	0.84	1.00	1.01	0.57	0.87	1.00	0.97	0.32	0.69	1.00	0.96	0.20	0.73	1.00
3-hydroxypyridine sulfate	Xenobiotics	Chemical	0.46	0.00	0.20	1.00	0.96	0.44	0.84	1.00	0.59	0.34	0.84	1.00	2.07	0.02	0.44	1.00	1.28	0.03	0.31	1.00	0.62	0.63	0.90	1.00
3-indoxyl sulfate	Amino Acid	Tryptophan Metabolism	0.98	0.39	0.78	1.00	1.01	0.98	0.99	1.00	1.12	0.17	0.75	1.00	1.03	0.43	0.79	1.00	1.14	0.10	0.45	1.00	1.11	0.16	0.73	1.00
3-methyl-2-oxobutyrate	Amino Acid	Leucine, Isoleucine and Val	1.00	0.95	0.99	1.00	1.00	0.97	0.99	1.00	1.01	0.70	0.94	1.00	1.00	0.70	0.92	1.00	1.01	0.72	0.89	1.00	1.01	1.00	1.00	1.00
3-methyl-2-oxovalerate	Amino Acid	Leucine, Isoleucine and Val	0.90	0.02	0.30	1.00	0.91	0.04	0.51	1.00	0.94	0.09	0.60	1.00	1.01	0.54	0.85	1.00	1.05	0.23	0.62	1.00	1.04	0.39	0.82	1.00
3-methylglutaconate	Amino Acid	Leucine, Isoleucine and Val	1.01	0.69	0.94	1.00	1.04	0.27	0.77	1.00	1.05	0.15	0.74	1.00	1.03	0.33	0.74	1.00	1.05	0.43	0.75	1.00	1.01	0.60	0.89	1.00
3-methylhistidine	Amino Acid	Histidine Metabolism	0.74	0.21	0.66	1.00	0.64	0.12	0.67	1.00	0.77	0.54	0.89	1.00	0.87	0.98	0.99	1.00	1.04	0.49	0.79	1.00	1.20	0.46	0.84	1.00
3-ureidopropionate	Nucleotide	Pyrimidine Metabolism, Ur	0.97	0.84	0.95	1.00	0.97	0.67	0.92	1.00	0.93	0.18	0.76	1.00	1.00	0.67	0.91	1.00	0.95	0.21	0.60	1.00	0.96	0.18	0.73	1.00
4-acetamidobutanoate	Amino Acid	Polyamine Metabolism	1.12	0.13	0.55	1.00	1.12	0.06	0.56	1.00	1.13	0.01	0.44	1.00	1.00	0.57	0.87	1.00	1.02	0.15	0.52	1.00	1.02	0.35	0.80	1.00
4-allylphenol sulfate	Xenobiotics	Food Component/Plant	0.96	0.42	0.79	1.00	1.11	0.53	0.89	1.00	0.78	0.76	0.94	1.00	1.16	0.27	0.70	1.00	0.81	0.53	0.82	1.00	0.70	0.24	0.75	1.00
4-ethylphenyl sulfate	Xenobiotics	Benzoate Metabolism	0.55	0.32	0.73	1.00	0.77	0.20	0.75	1.00	0.73	0.78	0.94	1.00	1.40	0.02	0.44	1.00	1.33	0.22	0.62	1.00	0.95	0.20	0.73	1.00
4-hydroxychlorothalonil	Xenobiotics	Chemical	0.93	0.04	0.40	1.00	1.09	0.12	0.67	1.00	1.04	0.64	0.92	1.00	1.17	0.00	0.21	0.42	1.12	0.02	0.31	1.00	0.96	0.21	0.73	1.00
4-hydroxyphenylpyruvate	Amino Acid	Tyrosine Metabolism	0.88	0.03	0.40	1.00	0.95	0.17	0.72	1.00	0.94	0.33	0.83	1.00	1.07	0.32	0.73	1.00	1.06	0.27	0.64	1.00	0.99	0.98	1.00	1.00
4-methyl-2-oxopentanoate	Amino Acid	Leucine, Isoleucine and Val	0.95	0.20	0.64	1.00	0.92	0.10	0.65	1.00	0.95	0.15	0.74	1.00	0.97	0.79	0.94	1.00	1.00	0.97	0.99	1.00	1.03	0.43	0.83	1.00
4-methylcatechol sulfate	Xenobiotics	Benzoate Metabolism	0.63	0.35	0.74	1.00	0.74	0.50	0.88	1.00	0.55	0.05	0.57	1.00	1.17	1.00	1.00	1.00	0.87	0.45	0.77	1.00	0.74	0.43	0.83	1.00
4-vinylphenol sulfate	Xenobiotics	Benzoate Metabolism	1.62	0.27	0.71	1.00	0.80	0.28	0.77	1.00	1.03	0.31	0.82	1.00	0.49	0.64	0.89	1.00	0.63	1.00	1.00	1.00	1.28	0.35	0.80	1.00
5,6-dihydrouridine	Nucleotide	Pyrimidine Metabolism, Ur	1.04	0.37	0.76	1.00	1.04	0.30	0.78	1.00	1.03	0.34	0.84	1.00	1.00	0.82	0.95	1.00	0.99	0.95	0.99	1.00	0.99	0.95	0.99	1.00
5-acetylamino-6-amino-3-methyluracil	Xenobiotics	Xanthine Metabolism	0.83	0.02	0.33	1.00	1.04	0.57	0.89	1.00	1.11	0.22	0.76	1.00	1.26	0.02	0.44	1.00	1.34	0.02	0.31	1.00	1.06	0.58	0.88	1.00
5-dodecenoate (12:1n7)	Lipid	Medium Chain Fatty Acid	1.23	0.01	0.20	1.00	1.10	0.18	0.73	1.00	1.21	0.12	0.65	1.00	0.90	0.05	0.51	1.00	0.98	0.21	0.60	1.00	1.10	0.86	0.97	1.00
5-dodecenoylcarnitine (C12:1)	Lipid	Fatty Acid Metabolism(Acyl	1.22	0.01	0.26	1.00	1.11	0.27	0.77	1.00	1.09	0.21	0.76	1.00	0.90	0.15	0.63	1.00	0.89	0.05	0.39	1.00	0.98	0.73	0.93	1.00
5-hydroxylysine	Amino Acid	Lysine Metabolism	1.00	0.95	0.99	1.00	0.97	0.29	0.78	1.00	1.12	0.44	0.87	1.00	0.97	0.63	0.88	1.00	1.12	0.18	0.57	1.00	1.16	0.04	0.57	1.00
5-methylthioadenosine (MTA)	Amino Acid	Polyamine Metabolism	1.05	0.56	0.88	1.00	1.08	0.13	0.69	1.00	1.07	0.29	0.82	1.00	1.03	0.32	0.73	1.00	1.03	0.75	0.92	1.00	1.00	0.89	0.98	1.00
5-methylthioribose	Amino Acid	Methionine, Cysteine, SAM	1.05	0.23	0.66	1.00	1.01	0.72	0.94	1.00	1.02	0.56	0.89	1.00	0.96	0.15	0.63	1.00	0.97	0.23	0.62	1.00	1.01	0.72	0.93	1.00
5-methyluridine (ribothymidine)	Nucleotide	Pyrimidine Metabolism, Ur	0.96	0.35	0.74	1.00	0.99	0.89	0.97	1.00	1.02	0.50	0.89	1.00	1.03	0.48	0.82	1.00	1.06	0.01	0.31	1.00	1.03	0.27	0.75	1.00
5-oxoproline	Amino Acid	Glutathione Metabolism	1.00	0.98	1.00	1.00	1.05	0.33	0.79	1.00	1.05	0.19	0.76	1.00	1.05	0.08	0.58	1.00	1.05	0.15	0.52	1.00	1.00	0.84	0.97	1.00
6-bromotryptophan	Amino Acid	Tryptophan Metabolism	0.95	0.63	0.93	1.00	0.97	0.54	0.89	1.00	1.04	0.52	0.89	1.00	1.02	0.95	0.99	1.00	1.10	0.09	0.43	1.00	1.08	0.12	0.73	1.00
6-hydroxyindole sulfate	Xenobiotics	Chemical	0.96	0.32	0.73	1.00	0.98	0.69	0.92	1.00	1.11	0.30	0.82	1.00	1.03	0.54	0.85	1.00	1.16	0.06	0.39	1.00	1.13	0.13	0.73	1.00
6-oxopiperidine-2-carboxylate	Amino Acid	Lysine Metabolism	0.96	0.61	0.92	1.00	1.03	0.94	0.98	1.00	0.95	0.79	0.95	1.00	1.07	0.31	0.73	1.00	0.99	0.94	0.98	1.00	0.92	0.27	0.75	1.00
7-HOCA	Lipid	Sterol	1.03	0.32	0.73	1.00	1.00	0.92	0.98	1.00	1.01	0.70	0.94	1.00	0.97	0.40	0.79	1.00	0.98	0.97	0.99	1.00	1.00	1.00	1.00	1.00
7-methylguanine	Nucleotide	Purine Metabolism, Guanir	1.02	0.86	0.96	1.00	1.05	0.20	0.75	1.00	1.06	0.16	0.75	1.00	1.04	0.06	0.51	1.00	1.04	0.06	0.39	1.00	1.01	0.79	0.96	1.00
9-hydroxystearate	Lipid	Fatty Acid, Monohydroxy	1.01	0.79	0.95	1.00	1.06	0.26	0.77	1.00	1.10	0.26	0.81	1.00	1.06	0.40	0.79	1.00	1.10	0.22	0.62	1.00	1.04	0.79	0.96	1.00
acetylcarnitine (C2)	Lipid	Fatty Acid Metabolism(Acyl	1.11	0.04	0.42	1.00	1.08	0.24	0.77	1.00	0.99	0.86	0.96	1.00	0.97	0.39	0.79	1.00	0.89	0.00	0.31	1.00	0.92	0.17	0.73	1.00
acisoga	Amino Acid	Polyamine Metabolism	1.15	0.01	0.23	1.00	1.04	0.34	0.80	1.00	1.04	0.31	0.82	1.00	0.91	0.05	0.51	1.00	0.90	0.15	0.52	1.00	0.99	0.98	1.00	1.00
aconitate [cis or trans]	Energy	TCA Cycle	0.93	0.08	0.50	1.00	0.94	0.06	0.56	1.00	0.96	0.52	0.89	1.00	1.00	0.81	0.95	1.00	1.03	0.10	0.46	1.00	1.03	0.21	0.73	1.00
adrenate (22:4n6)	Lipid	Polyunsaturated Fatty Acid	0.99	0.50	0.84	1.00	1.03	0.11	0.66	1.00	0.93	0.95	0.99	1.00	1.04	0.16	0.64	1.00	0.94	1.00	1.00	1.00	0.91	0.07	0.64	1.00
alanine	Amino Acid	Alanine and Aspartate Meta	0.99	0.84	0.95	1.00	1.06	0.24	0.77	1.00	1.04	0.33	0.83	1.00	1.07	0.19	0.67	1.00	1.05	0.60	0.86	1.00	0.98	0.78	0.95	1.00
allantoin	Nucleotide	Purine Metabolism, (Hypo)	0.82	0.02	0.35	1.00	1.00	0.78	0.94	1.00	0.90	0.28	0.82	1.00	1.22	0.00	0.31	1.00	1.10	0.02	0.31	1.00	0.90	0.09	0.68	1.00
alpha-hydroxyisocaproate	Amino Acid	Leucine, Isoleucine and Val	0.90	0.04	0.40	1.00	0.88	0.05	0.53	1.00	0.95	0.43	0.87	1.00	0.99	0.92	0.99	1.00	1.06	0.28	0.65	1.00	1.07	0.21	0.73	1.00
alpha-hydroxyisovalerate	Amino Acid	Leucine, Isoleucine and Val	1.02	0.75	0.95	1.00	1.02	0.76	0.94	1.00	0.95	0.54	0.89	1.00	1.00	0.72	0.92	1.00	0.93	0.24	0.63	1.00	0.93	0.08	0.65	1.00

alpha-ketoglutaramate*	Amino Acid	Glutamate Metabolism	0.92	0.10	0.51	1.00	1.03	0.97	0.99	1.00	0.94	0.08	0.60	1.00	1.12	0.05	0.51	1.00	1.02	0.81	0.93	1.00	0.91	0.03	0.57	1.00
alpha-ketoglutarate	Energy	TCA Cycle	1.00	1.00	1.00	1.00	1.00	0.79	0.94	1.00	1.02	0.98	1.00	1.00	1.00	0.81	0.95	1.00	1.02	0.66	0.88	1.00	1.02	0.69	0.93	1.00
alpha-tocopherol	Cofactors and	Tocopherol Metabolism	0.99	0.58	0.90	1.00	0.99	0.94	0.98	1.00	0.96	0.56	0.89	1.00	1.00	0.75	0.93	1.00	0.97	0.38	0.73	1.00	0.97	0.92	0.98	1.00
androstenediol (3beta,17beta) disulfate (1)	Lipid	Androgenic Steroids	1.00	0.97	0.99	1.00	1.04	0.79	0.94	1.00	1.04	0.84	0.96	1.00	1.04	0.43	0.79	1.00	1.04	0.53	0.82	1.00	1.00	0.73	0.93	1.00
androstenediol (3beta,17beta) disulfate (2)	Lipid	Androgenic Steroids	0.99	0.97	0.99	1.00	0.95	0.72	0.94	1.00	1.03	0.61	0.92	1.00	0.96	0.30	0.73	1.00	1.04	0.54	0.83	1.00	1.09	0.16	0.73	1.00
androstenediol (3beta,17beta) monosulfate (1)	Lipid	Androgenic Steroids	1.06	0.38	0.77	1.00	1.08	0.40	0.82	1.00	1.02	0.90	0.98	1.00	1.02	0.95	0.99	1.00	0.97	0.64	0.87	1.00	0.95	0.13	0.73	1.00
androsterone glucuronide	Lipid	Androgenic Steroids	0.98	0.94	0.99	1.00	0.99	0.81	0.95	1.00	1.05	0.22	0.76	1.00	1.02	0.95	0.99	1.00	1.07	0.12	0.49	1.00	1.05	0.75	0.94	1.00
androsterone sulfate	Lipid	Androgenic Steroids	1.06	0.39	0.78	1.00	1.04	0.44	0.84	1.00	1.04	0.95	0.99	1.00	0.98	0.26	0.70	1.00	0.98	0.56	0.83	1.00	1.00	0.54	0.87	1.00
arabitol/xylitol	Carbohydrate	Pentose Metabolism	1.01	0.89	0.98	1.00	1.03	0.69	0.92	1.00	0.99	0.95	0.99	1.00	1.02	0.97	0.99	1.00	0.98	0.89	0.96	1.00	0.96	0.95	0.99	1.00
arabonate/xylonate	Carbohydrate	Pentose Metabolism	0.83	0.04	0.40	1.00	0.91	0.22	0.77	1.00	0.82	0.03	0.47	1.00	1.09	0.21	0.67	1.00	0.99	0.81	0.93	1.00	0.91	0.29	0.75	1.00
arachidate (20:0)	Lipid	Long Chain Fatty Acid	0.81	0.02	0.30	1.00	0.88	0.15	0.70	1.00	0.81	0.00	0.29	1.00	1.08	0.25	0.70	1.00	0.99	0.84	0.94	1.00	0.92	0.45	0.83	1.00
arachidonate (20:4n6)	Lipid	Polyunsaturated Fatty Acid	0.91	0.16	0.59	1.00	1.00	0.92	0.98	1.00	1.02	0.72	0.94	1.00	1.10	0.12	0.61	1.00	1.12	0.01	0.31	1.00	1.02	0.97	1.00	1.00
arachidonoylcarnitine (C20:4)	Lipid	Fatty Acid Metabolism(Acyl	1.01	0.70	0.94	1.00	1.08	0.09	0.64	1.00	1.08	0.07	0.58	1.00	1.06	0.10	0.60	1.00	1.07	0.05	0.39	1.00	1.00	0.86	0.97	1.00
arachidonoylcholine	Lipid	Fatty Acid Metabolism (Acy	0.96	0.98	1.00	1.00	0.98	0.84	0.95	1.00	1.11	0.34	0.84	1.00	1.02	0.84	0.96	1.00	1.16	0.01	0.31	1.00	1.13	0.08	0.65	1.00
argininate*	Amino Acid	Urea cycle; Arginine and Prc	0.92	0.78	0.95	1.00	1.06	0.46	0.85	1.00	1.08	0.36	0.85	1.00	1.15	0.03	0.46	1.00	1.18	0.02	0.31	1.00	1.02	0.89	0.98	1.00
arginine	Amino Acid	Urea cycle; Arginine and Prc	1.00	0.92	0.99	1.00	1.02	0.57	0.89	1.00	1.03	0.27	0.82	1.00	1.01	0.66	0.90	1.00	1.03	0.18	0.57	1.00	1.02	0.49	0.85	1.00
asparagine	Amino Acid	Alanine and Aspartate Meta	1.00	0.82	0.95	1.00	1.03	0.38	0.82	1.00	1.05	0.06	0.57	1.00	1.04	0.22	0.68	1.00	1.06	0.02	0.31	1.00	1.02	0.34	0.80	1.00
aspartate	Amino Acid	Alanine and Aspartate Meta	0.94	0.17	0.60	1.00	1.05	0.48	0.86	1.00	0.98	0.63	0.92	1.00	1.12	0.07	0.55	1.00	1.04	0.42	0.74	1.00	0.93	0.49	0.85	1.00
azelate (nonanedioate; C9)	Lipid	Fatty Acid, Dicarboxylate	0.74	0.05	0.43	1.00	0.78	0.32	0.79	1.00	1.06	0.31	0.82	1.00	1.05	0.78	0.94	1.00	1.43	0.57	0.85	1.00	1.36	0.50	0.86	1.00
behenoyl dihydrosphingomyelin (d18:0/22:0)*	Lipid	Dihydrosphingomyelins	0.96	0.94	0.99	1.00	0.88	0.16	0.72	1.00	0.98	0.78	0.94	1.00	0.91	0.10	0.60	1.00	1.01	0.98	1.00	1.00	1.12	0.04	0.57	1.00
behenoyl sphingomyelin (d18:1/22:0)*	Lipid	Sphingomyelins	0.99	0.81	0.95	1.00	0.93	0.15	0.70	1.00	1.00	0.86	0.96	1.00	0.94	0.18	0.66	1.00	1.01	0.42	0.74	1.00	1.08	0.02	0.50	1.00
beta-alanine	Nucleotide	Pyrimidine Metabolism, Ur	0.87	0.21	0.66	1.00	0.92	0.92	0.98	1.00	0.96	0.13	0.69	1.00	1.05	0.43	0.79	1.00	1.10	0.02	0.31	1.00	1.05	0.16	0.73	1.00
beta-citrylglutamate	Amino Acid	Glutamate Metabolism	1.01	0.82	0.95	1.00	1.08	0.14	0.70	1.00	1.04	0.56	0.89	1.00	1.07	0.19	0.67	1.00	1.03	0.35	0.71	1.00	0.97	0.98	1.00	1.00
beta-cryptoxanthin	Cofactors and	Vitamin A Metabolism	0.95	0.95	0.99	1.00	0.92	0.57	0.89	1.00	0.92	0.61	0.92	1.00	0.97	0.73	0.93	1.00	0.97	0.86	0.95	1.00	1.01	0.79	0.96	1.00
beta-hydroxyisovalerate	Amino Acid	Leucine, Isoleucine and Val	0.96	0.49	0.83	1.00	0.97	0.75	0.94	1.00	0.97	1.00	1.00	1.00	1.01	0.58	0.87	1.00	1.01	0.53	0.82	1.00	1.01	0.84	0.97	1.00
betaine	Amino Acid	Glycine, Serine and Threoni	0.97	0.40	0.79	1.00	0.97	0.30	0.78	1.00	0.97	0.29	0.82	1.00	1.00	0.58	0.87	1.00	1.00	0.95	0.99	1.00	1.00	1.00	1.00	1.00
bilirubin (E,E)*	Cofactors and	Hemoglobin and Porphyrin	1.12	0.11	0.53	1.00	0.96	0.57	0.89	1.00	1.03	0.61	0.92	1.00	0.85	0.04	0.50	1.00	0.92	0.07	0.41	1.00	1.07	0.25	0.75	1.00
bilirubin (E,Z or Z,E)*	Cofactors and	Hemoglobin and Porphyrin	1.02	0.44	0.80	1.00	0.95	0.39	0.82	1.00	1.02	0.69	0.94	1.00	0.94	0.24	0.68	1.00	1.00	0.81	0.93	1.00	1.07	0.20	0.73	1.00
bilirubin	Cofactors and	Hemoglobin and Porphyrin	1.04	0.42	0.79	1.00	0.97	0.52	0.88	1.00	1.04	0.52	0.89	1.00	0.94	0.17	0.65	1.00	1.01	0.92	0.98	1.00	1.07	0.10	0.68	1.00
biliverdin	Cofactors and	Hemoglobin and Porphyrin	1.27	0.00	0.20	1.00	1.04	0.63	0.91	1.00	1.09	0.20	0.76	1.00	0.82	0.01	0.44	1.00	0.86	0.06	0.39	1.00	1.05	0.45	0.83	1.00
butyrylcarnitine (C4)	Lipid	Fatty Acid Metabolism (alsc	1.19	0.00	0.06	0.12	1.19	0.00	0.24	0.38	1.19	0.01	0.38	1.00	1.00	0.73	0.93	1.00	1.00	0.40	0.74	1.00	1.01	0.40	0.82	1.00
caffeine	Xenobiotics	Xanthine Metabolism	0.64	0.04	0.42	1.00	0.78	0.12	0.67	1.00	0.66	0.03	0.47	1.00	1.23	0.05	0.51	1.00	1.04	0.43	0.75	1.00	0.85	0.60	0.89	1.00
caprate (10:0)	Lipid	Medium Chain Fatty Acid	0.86	0.92	0.99	1.00	0.91	0.92	0.98	1.00	0.87	0.97	1.00	1.00	1.06	0.97	0.99	1.00	1.01	0.84	0.94	1.00	0.95	0.78	0.95	1.00
carnitine	Lipid	Carnitine Metabolism	1.00	0.94	0.99	1.00	1.03	0.34	0.80	1.00	1.01	0.64	0.92	1.00	1.03	0.48	0.82	1.00	1.00	0.58	0.85	1.00	0.98	0.70	0.93	1.00
carotene diol (1)	Cofactors and	Vitamin A Metabolism	0.94	0.29	0.73	1.00	0.94	0.14	0.70	1.00	1.04	0.49	0.89	1.00	1.00	0.78	0.94	1.00	1.10	0.08	0.43	1.00	1.10	0.06	0.64	1.00
carotene diol (2)	Cofactors and	Vitamin A Metabolism	0.98	0.87	0.97	1.00	1.00	0.79	0.94	1.00	1.04	0.26	0.81	1.00	1.03	0.35	0.76	1.00	1.06	0.10	0.45	1.00	1.04	0.08	0.65	1.00
catechol sulfate	Xenobiotics	Benzoate Metabolism	0.97	0.78	0.95	1.00	0.79	0.12	0.67	1.00	0.84	0.13	0.68	1.00	0.81	0.14	0.62	1.00	0.87	0.50	0.80	1.00	1.07	0.24	0.75	1.00
ceramide (d16:1/24:1, d18:1/22:1)*	Lipid	Ceramides	1.01	0.92	0.99	1.00	0.99	0.94	0.98	1.00	0.99	0.86	0.96	1.00	0.99	0.94	0.99	1.00	0.99	0.46	0.77	1.00	1.00	0.73	0.93	1.00
ceramide (d18:1/14:0, d16:1/16:0)*	Lipid	Ceramides	1.03	0.92	0.99	1.00	1.06	0.81	0.95	1.00	1.02	0.75	0.94	1.00	1.02	0.89	0.98	1.00	0.99	0.76	0.92	1.00	0.97	0.58	0.88	1.00
ceramide (d18:1/20:0, d16:1/22:0, d20:1/18:0	Lipid	Ceramides	1.07	0.31	0.73	1.00	1.01	0.90	0.98	1.00	1.02	0.92	0.98	1.00	0.94	0.60	0.87	1.00	0.95	0.18	0.57	1.00	1.01	0.90	0.98	1.00
ceramide (d18:2/24:1, d18:1/24:2)*	Lipid	Ceramides	1.06	0.20	0.64	1.00	1.03	0.56	0.89	1.00	1.00	1.00	1.00	1.00	0.97	0.48	0.82	1.00	0.94	0.01	0.31	1.00	0.97	0.29	0.75	1.00
cerotoylcarnitine (C26)*	Lipid	Fatty Acid Metabolism(Acyl	0.98	0.35	0.74	1.00	0.90	0.00	0.29	1.00	0.98	0.37	0.85	1.00	0.92	0.01	0.44	1.00	1.00	1.00	1.00	1.00	1.09	0.03	0.57	1.00
C-glycosyltryptophan	Amino Acid	Tryptophan Metabolism	0.99	0.92	0.99	1.00	1.03	0.52	0.88	1.00	1.04	0.20	0.76	1.00	1.04	0.52	0.84	1.00	1.05	0.11	0.48	1.00	1.01	0.70	0.93	1.00
cholate	Lipid	Primary Bile Acid Metabolis	0.83	0.98	1.00	1.00	1.13	0.45	0.85	1.00	1.09	0.48	0.89	1.00	1.35	0.39	0.79	1.00	1.30	0.36	0.71	1.00	0.96	0.84	0.97	1.00
cholesterol	Lipid	Sterol	1.01	0.57	0.89	1.00	0.98	0.84	0.95	1.00	1.00	0.70	0.94	1.00	0.98	0.52	0.84	1.00	0.99	0.82	0.94	1.00	1.01	0.54	0.87	1.00
choline	Lipid	Phospholipid Metabolism	0.99	0.69	0.94	1.00	0.99	0.98	0.99	1.00	1.01	0.81	0.95	1.00	1.00	0.75	0.93	1.00	1.01	0.73	0.90	1.00	1.01	0.75	0.94	1.00
cinnamoylglycine	Xenobiotics	Food Component/Plant	1.01	0.63	0.93	1.00	0.82	0.22	0.77	1.00	1.04	0.56	0.89	1.00	0.81	0.43	0.79	1.00	1.03	0.48	0.78	1.00	1.27	0.10	0.70	1.00
cis-4-decenoate (10:1n6)*	Lipid	Medium Chain Fatty Acid	1.20	0.01	0.22	1.00	1.09	0.16	0.72	1.00	1.13	0.11	0.65	1.00	0.91	0.16	0.64	1.00	0.94	0.21	0.61	1.00	1.03	1.00	1.00	1.00
cis-4-decenoylcarnitine (C10:1)	Lipid	Fatty Acid Metabolism(Acyl	1.11	0.14	0.57	1.00	1.01	0.69	0.92	1.00	1.07	0.21	0.76	1.00	0.90	0.18	0.66	1.00	0.96	0.54	0.83	1.00	1.06	0.40	0.82	1.00
citraconate/glutaconate	Energy	TCA Cycle	0.52	0.01	0.20	1.00	0.85	0.32	0.79	1.00	0.63	0.19	0.76	1.00	1.64	0.01	0.44	1.00	1.23	0.01	0.31	1.00	0.75	0.97	1.00	1.00
citrate	Energy	TCA Cycle	0.93	0.18	0.62	1.00	0.90	0.01	0.30	1.00	0.95	0.30	0.82	1.00	0.96	0.21	0.67	1.00	1.02	0.50	0.80	1.00	1.06	0.08	0.65	1.00
citrulline	Amino Acid	Urea cycle; Arginine and Prc	1.05	0.37	0.76	1.00	1.06	0.21	0.76	1.00	1.12	0.07	0.58	1.00	1.01	0.75	0.93	1.00	1.06	0.29	0.67	1.00	1.05	0.21	0.73	1.00
cortisol	Lipid	Corticosteroids	0.96	0.66	0.93	1.00	0.93	0.82	0.95	1.00	1.16	0.05	0.57	1.00	0.97	1.00	1.00	1.00	1.20	0.00	0.31	1.00	1.24	0.00	0.15	0.46
creatine	Amino Acid	Creatine Metabolism	0.93	0.40	0.79	1.00	0.97	0.79	0.94	1.00	0.99	1.00	1.00	1.00	1.04	0.12	0.61	1.00	1.06	0.27	0.64	1.00	1.02	0.46	0.84	1.00

creatinine	Amino Acid	Creatine Metabolism	1.01	0.84	0.95	1.00	1.01	0.38	0.82	1.00	1.00	0.75	0.94	1.00	1.01	0.63	0.88	1.00	1.00	0.76	0.92	1.00	0.99	0.61	0.90	1.00
cys-gly, oxidized	Amino Acid	Glutathione Metabolism	1.02	0.48	0.82	1.00	1.05	0.10	0.65	1.00	1.12	0.01	0.42	1.00	1.03	0.44	0.80	1.00	1.10	0.02	0.31	1.00	1.06	0.17	0.73	1.00
cysteine	Amino Acid	Methionine, Cysteine, SAM	1.09	0.81	0.95	1.00	1.12	0.21	0.75	1.00	1.18	0.03	0.47	1.00	1.02	0.53	0.84	1.00	1.08	0.15	0.52	1.00	1.05	0.07	0.65	1.00
cysteine-glutathione disulfide	Amino Acid	Glutathione Metabolism	1.02	0.70	0.94	1.00	1.02	0.63	0.91	1.00	1.12	0.07	0.58	1.00	1.00	0.67	0.91	1.00	1.10	0.08	0.43	1.00	1.10	0.14	0.73	1.00
cysteinylglycine disulfide*	Amino Acid	Glutathione Metabolism	1.00	0.97	0.99	1.00	1.01	0.36	0.82	1.00	1.05	0.05	0.57	1.00	1.01	0.78	0.94	1.00	1.05	0.12	0.48	1.00	1.05	0.10	0.68	1.00
cystine	Amino Acid	Methionine, Cysteine, SAM	1.09	0.32	0.73	1.00	0.99	0.86	0.95	1.00	1.00	0.72	0.94	1.00	0.90	0.26	0.70	1.00	0.92	0.54	0.83	1.00	1.01	0.78	0.95	1.00
decanoylcarnitine (C10)	Lipid	Fatty Acid Metabolism(Acyl	1.03	0.97	0.99	1.00	0.95	0.82	0.95	1.00	0.98	0.76	0.94	1.00	0.92	0.39	0.79	1.00	0.95	0.30	0.67	1.00	1.03	0.78	0.95	1.00
dehydroepiandrosterone sulfate (DHEA-S)	Lipid	Androgenic Steroids	0.98	0.92	0.99	1.00	1.01	0.87	0.96	1.00	1.01	0.94	0.99	1.00	1.03	0.84	0.96	1.00	1.03	0.64	0.87	1.00	1.00	0.82	0.96	1.00
deoxycarnitine	Lipid	Carnitine Metabolism	0.99	0.81	0.95	1.00	1.08	0.13	0.69	1.00	1.03	0.48	0.89	1.00	1.10	0.03	0.46	1.00	1.05	0.25	0.64	1.00	0.96	0.26	0.75	1.00
diacylglycerol (12:0/18:1, 14:0/16:1, 16:0/14:1	Lipid	Diacylglycerol	0.76	0.28	0.72	1.00	0.87	0.58	0.90	1.00	0.78	0.48	0.89	1.00	1.14	0.75	0.93	1.00	1.03	0.52	0.82	1.00	0.90	0.81	0.96	1.00
diacylglycerol (14:0/18:1, 16:0/16:1) [1]*	Lipid	Diacylglycerol	0.91	0.67	0.93	1.00	0.90	0.82	0.95	1.00	0.85	0.48	0.89	1.00	0.98	0.56	0.86	1.00	0.93	0.66	0.88	1.00	0.95	0.66	0.92	1.00
diacylglycerol (14:0/18:1, 16:0/16:1) [2]*	Lipid	Diacylglycerol	0.90	0.63	0.93	1.00	0.89	0.72	0.94	1.00	0.88	0.52	0.89	1.00	0.99	0.64	0.89	1.00	0.98	0.95	0.99	1.00	0.99	0.57	0.88	1.00
diacylglycerol (16:1/18:2 [2], 16:0/18:3 [1])*	Lipid	Diacylglycerol	0.89	0.61	0.92	1.00	0.82	0.20	0.75	1.00	0.85	0.20	0.76	1.00	0.92	0.25	0.70	1.00	0.95	0.27	0.64	1.00	1.04	0.87	0.97	1.00
dihomolinoleate (20:2n6)	Lipid	Polyunsaturated Fatty Acid	1.03	0.40	0.79	1.00	1.03	0.29	0.78	1.00	0.97	0.64	0.92	1.00	0.99	0.76	0.93	1.00	0.94	0.34	0.70	1.00	0.94	0.43	0.83	1.00
dihomolinolenate (20:3n3 or 3n6)	Lipid	Polyunsaturated Fatty Acid	0.95	0.66	0.93	1.00	1.05	0.29	0.78	1.00	1.01	0.76	0.94	1.00	1.10	0.31	0.73	1.00	1.06	0.44	0.76	1.00	0.96	0.46	0.84	1.00
dihomo-linolenoyl-choline	Lipid	Fatty Acid Metabolism (Acy	1.01	0.76	0.95	1.00	1.04	0.70	0.93	1.00	1.15	0.22	0.76	1.00	1.03	0.52	0.84	1.00	1.14	0.15	0.52	1.00	1.11	0.58	0.88	1.00
dihydroorotate	Nucleotide	Pyrimidine Metabolism, Or	0.90	0.49	0.83	1.00	0.93	0.53	0.89	1.00	0.92	0.73	0.94	1.00	1.03	0.89	0.98	1.00	1.02	0.46	0.77	1.00	0.99	0.52	0.86	1.00
dimethylarginine (ADMA + SDMA)	Amino Acid	Urea cycle; Arginine and Pro	1.05	0.08	0.50	1.00	1.06	0.04	0.51	1.00	1.07	0.02	0.44	1.00	1.01	0.92	0.99	1.00	1.02	0.34	0.70	1.00	1.01	0.54	0.87	1.00
dimethylglycine	Amino Acid	Glycine, Serine and Threoni	0.98	0.75	0.95	1.00	0.99	0.57	0.89	1.00	1.01	0.79	0.95	1.00	1.01	0.75	0.93	1.00	1.03	0.44	0.76	1.00	1.02	0.38	0.82	1.00
docosadienoate (22:2n6)	Lipid	Polyunsaturated Fatty Acid	0.97	0.72	0.95	1.00	0.99	0.69	0.92	1.00	0.93	0.33	0.83	1.00	1.01	0.87	0.97	1.00	0.96	0.49	0.79	1.00	0.94	0.30	0.77	1.00
docosahexaenoate (DHA; 22:6n3)	Lipid	Polyunsaturated Fatty Acid	0.89	0.27	0.71	1.00	0.92	0.26	0.77	1.00	0.93	0.52	0.89	1.00	1.03	0.50	0.84	1.00	1.04	0.76	0.92	1.00	1.01	1.00	1.00	1.00
docosahexaenoylcarnitine (C22:6)*	Lipid	Fatty Acid Metabolism(Acyl	1.05	0.35	0.74	1.00	0.97	0.98	0.99	1.00	1.03	0.81	0.95	1.00	0.92	0.21	0.67	1.00	0.98	0.84	0.94	1.00	1.06	0.46	0.84	1.00
docosahexaenoylcholine	Lipid	Fatty Acid Metabolism (Acy	0.95	0.95	0.99	1.00	0.91	0.44	0.84	1.00	1.07	0.53	0.89	1.00	0.97	0.73	0.93	1.00	1.13	0.10	0.45	1.00	1.17	0.10	0.68	1.00
docosapentaenoate (DPA; 22:5n3)	Lipid	Polyunsaturated Fatty Acid	1.03	0.63	0.93	1.00	0.96	0.76	0.94	1.00	0.96	0.61	0.92	1.00	0.94	0.42	0.79	1.00	0.94	0.24	0.63	1.00	1.00	0.87	0.97	1.00
dodecadienoate (12:2)*	Lipid	Fatty Acid, Dicarboxylate	1.11	0.21	0.65	1.00	1.05	0.72	0.94	1.00	1.08	0.16	0.75	1.00	0.94	0.20	0.67	1.00	0.97	0.27	0.64	1.00	1.03	0.73	0.93	1.00
dodecanedioate (C12)	Lipid	Fatty Acid, Dicarboxylate	0.80	0.05	0.43	1.00	0.70	0.02	0.42	1.00	0.74	0.08	0.60	1.00	0.88	0.29	0.72	1.00	0.93	0.58	0.85	1.00	1.06	0.38	0.82	1.00
dodecenedioate (C12:1-DC)*	Lipid	Fatty Acid, Dicarboxylate	0.91	0.45	0.81	1.00	0.78	0.04	0.51	1.00	0.81	0.20	0.76	1.00	0.85	0.08	0.56	1.00	0.88	0.50	0.80	1.00	1.03	0.64	0.91	1.00
dopamine 3-O-sulfate	Amino Acid	Tyrosine Metabolism	0.75	0.13	0.56	1.00	0.91	0.33	0.79	1.00	0.80	0.25	0.80	1.00	1.21	0.63	0.88	1.00	1.06	0.82	0.94	1.00	0.87	0.86	0.97	1.00
eicosanedioate (C20-DC)	Lipid	Fatty Acid, Dicarboxylate	0.90	0.07	0.48	1.00	1.10	0.76	0.94	1.00	1.04	0.92	0.98	1.00	1.22	0.01	0.44	1.00	1.16	0.10	0.45	1.00	0.95	0.86	0.97	1.00
eicosapentaenoate (EPA; 20:5n3)	Lipid	Polyunsaturated Fatty Acid	0.92	0.34	0.74	1.00	0.96	0.95	0.99	1.00	1.02	0.67	0.93	1.00	1.04	0.34	0.75	1.00	1.11	0.13	0.50	1.00	1.06	0.28	0.75	1.00
eicosenoate (20:1n9 or 1n11)	Lipid	Long Chain Fatty Acid	1.04	0.42	0.79	1.00	0.98	0.84	0.95	1.00	0.93	0.54	0.89	1.00	0.94	0.24	0.68	1.00	0.90	0.10	0.46	1.00	0.95	0.56	0.88	1.00
ergothioneine	Xenobiotics	Food Component/Plant	1.01	0.79	0.95	1.00	0.99	0.94	0.98	1.00	1.02	0.81	0.95	1.00	0.98	0.79	0.94	1.00	1.00	0.64	0.87	1.00	1.03	0.97	1.00	1.00
erucate (22:1n9)	Lipid	Long Chain Fatty Acid	0.78	0.15	0.58	1.00	0.78	0.26	0.77	1.00	0.67	0.04	0.54	1.00	1.01	0.79	0.94	1.00	0.87	0.22	0.62	1.00	0.86	0.04	0.57	1.00
erythritol	Xenobiotics	Food Component/Plant	1.02	0.79	0.95	1.00	1.01	1.00	1.00	1.00	0.90	0.18	0.76	1.00	0.98	0.82	0.95	1.00	0.88	0.24	0.63	1.00	0.90	0.27	0.75	1.00
erythronate*	Carbohydrate	Aminosugar Metabolism	0.95	0.10	0.51	1.00	1.00	0.75	0.94	1.00	0.95	0.18	0.76	1.00	1.05	0.13	0.62	1.00	1.00	0.87	0.95	1.00	0.95	0.07	0.65	1.00
ethylmalonate	Amino Acid	Leucine, Isoleucine and Val	1.12	0.10	0.51	1.00	1.06	0.14	0.70	1.00	1.02	0.69	0.94	1.00	0.94	0.25	0.70	1.00	0.91	0.08	0.43	1.00	0.97	0.10	0.68	1.00
etiocholanolone glucuronide	Lipid	Androgenic Steroids	0.98	0.64	0.93	1.00	1.02	0.66	0.92	1.00	1.06	0.25	0.80	1.00	1.04	0.42	0.79	1.00	1.08	0.13	0.50	1.00	1.04	0.54	0.87	1.00
Fibrinopeptide A (2-15)	Peptide	Fibrinogen Cleavage Peptide	0.92	0.04	0.42	1.00	0.98	0.49	0.87	1.00	0.97	0.50	0.89	1.00	1.07	0.12	0.61	1.00	1.06	0.15	0.52	1.00	0.99	0.72	0.93	1.00
Fibrinopeptide A (3-15)	Peptide	Fibrinogen Cleavage Peptide	0.87	0.00	0.11	1.00	0.98	0.49	0.87	1.00	0.94	0.76	0.94	1.00	1.12	0.11	0.61	1.00	1.08	0.01	0.31	1.00	0.97	0.79	0.96	1.00
Fibrinopeptide A (3-16)	Peptide	Fibrinogen Cleavage Peptide	1.08	0.25	0.70	1.00	1.05	0.53	0.89	1.00	1.10	0.33	0.83	1.00	0.97	0.34	0.75	1.00	1.02	0.72	0.89	1.00	1.04	0.40	0.82	1.00
Fibrinopeptide A (4-15)	Peptide	Fibrinogen Cleavage Peptide	0.88	0.01	0.26	1.00	1.01	0.79	0.94	1.00	0.93	0.69	0.94	1.00	1.14	0.02	0.44	1.00	1.05	0.18	0.57	1.00	0.92	0.48	0.85	1.00
Fibrinopeptide A (5-16)*	Peptide	Fibrinogen Cleavage Peptide	1.02	0.79	0.95	1.00	1.05	0.66	0.92	1.00	1.06	0.28	0.82	1.00	1.03	0.90	0.98	1.00	1.05	0.46	0.77	1.00	1.02	0.57	0.88	1.00
Fibrinopeptide A (7-16)*	Peptide	Fibrinogen Cleavage Peptide	0.93	0.13	0.56	1.00	0.97	0.40	0.82	1.00	0.98	0.48	0.89	1.00	1.04	0.53	0.84	1.00	1.04	0.23	0.62	1.00	1.01	0.92	0.98	1.00
Fibrinopeptide A (8-16)	Peptide	Fibrinogen Cleavage Peptide	0.93	0.20	0.64	1.00	1.03	0.52	0.88	1.00	1.04	0.67	0.93	1.00	1.10	0.33	0.74	1.00	1.11	0.11	0.47	1.00	1.01	0.89	0.98	1.00
DSGEGDFXAEGGGVR*	Peptide	Fibrinogen Cleavage Peptide	1.07	0.21	0.65	1.00	1.04	0.67	0.92	1.00	1.10	0.22	0.76	1.00	0.96	0.28	0.72	1.00	1.02	0.73	0.90	1.00	1.06	0.21	0.73	1.00
Fibrinopeptide B (1-12)	Peptide	Fibrinogen Cleavage Peptide	0.96	0.42	0.79	1.00	0.98	0.56	0.89	1.00	1.02	0.75	0.94	1.00	1.02	0.98	0.99	1.00	1.06	0.26	0.64	1.00	1.04	0.01	0.50	1.00
Fibrinopeptide B (1-13)	Peptide	Fibrinogen Cleavage Peptide	1.03	0.61	0.92	1.00	1.02	0.66	0.92	1.00	1.05	0.33	0.83	1.00	0.99	0.60	0.87	1.00	1.02	0.76	0.92	1.00	1.02	0.44	0.83	1.00
fructose	Carbohydrate	Fructose, Mannose and Gal	0.91	0.32	0.73	1.00	1.05	0.69	0.92	1.00	0.92	0.43	0.87	1.00	1.15	0.04	0.51	1.00	1.01	1.00	1.00	1.00	0.88	0.02	0.54	1.00
gamma-CEHC	Cofactors and	Tocopherol Metabolism	0.82	0.05	0.43	1.00	0.89	0.15	0.70	1.00	1.07	0.35	0.85	1.00	1.08	0.87	0.97	1.00	1.30	0.01	0.31	1.00	1.21	0.03	0.57	1.00
gamma-glutamylalanine	Peptide	Gamma-glutamyl Amino Ac	0.82	0.09	0.50	1.00	0.80	0.03	0.51	1.00	0.85	0.40	0.86	1.00	0.98	0.90	0.98	1.00	1.04	0.52	0.82	1.00	1.05	0.28	0.75	1.00
gamma-glutamyl-alpha-lysine	Peptide	Gamma-glutamyl Amino Ac	1.07	0.14	0.57	1.00	1.09	0.04	0.51	1.00	1.09	0.02	0.47	1.00	1.02	0.34	0.75	1.00	1.02	0.34	0.70	1.00	1.00	0.84	0.97	1.00
gamma-glutamylcitrulline*	Peptide	Gamma-glutamyl Amino Ac	1.03	0.60	0.91	1.00	1.08	0.13	0.69	1.00	1.14	0.08	0.58	1.00	1.04	0.40	0.79	1.00	1.10	0.02	0.31	1.00	1.06	0.22	0.74	1.00
gamma-glutamyl-epsilon-lysine	Peptide	Gamma-glutamyl Amino Ac	0.96	0.32	0.73	1.00	0.96	0.53	0.89	1.00	1.04	0.42	0.87	1.00	1.00	0.92	0.99	1.00	1.08	0.13	0.50	1.00	1.08	0.06	0.64	1.00
gamma-glutamylglutamine	Peptide	Gamma-glutamyl Amino Ac	1.01	0.79	0.95	1.00	1.04	0.19	0.75	1.00	1.05	0.04	0.54	1.00	1.03	0.27	0.70	1.00	1.05	0.08	0.43	1.00	1.01	0.87	0.97	1.00

gamma-glutamylglycine	Peptide	Gamma-glutamyl Amino Ac	1.04	0.43	0.80	1.00	1.09	0.08	0.62	1.00	1.11	0.02	0.47	1.00	1.05	0.36	0.77	1.00	1.06	0.04	0.36	1.00	1.02	0.45	0.83	1.00
gamma-glutamylhistidine	Peptide	Gamma-glutamyl Amino Ac	0.98	0.64	0.93	1.00	0.92	0.40	0.82	1.00	1.08	0.07	0.58	1.00	0.94	0.30	0.73	1.00	1.10	0.03	0.31	1.00	1.17	0.01	0.50	1.00
gamma-glutamylisoleucine*	Peptide	Gamma-glutamyl Amino Ac	1.00	0.97	0.99	1.00	1.07	0.37	0.82	1.00	1.08	0.37	0.85	1.00	1.07	0.12	0.61	1.00	1.08	0.33	0.69	1.00	1.01	0.73	0.93	1.00
gamma-glutamylleucine	Peptide	Gamma-glutamyl Amino Ac	0.95	0.27	0.71	1.00	1.02	0.82	0.95	1.00	0.99	0.75	0.94	1.00	1.07	0.16	0.64	1.00	1.04	0.38	0.73	1.00	0.97	0.82	0.96	1.00
gamma-glutamylmethionine	Peptide	Gamma-glutamyl Amino Ac	0.95	0.58	0.90	1.00	1.04	0.27	0.77	1.00	1.06	0.16	0.75	1.00	1.09	0.02	0.44	1.00	1.12	0.01	0.31	1.00	1.02	0.35	0.80	1.00
gamma-glutamylthreonine	Peptide	Gamma-glutamyl Amino Ac	1.08	0.32	0.73	1.00	1.13	0.03	0.51	1.00	1.18	0.00	0.29	1.00	1.04	0.17	0.65	1.00	1.09	0.06	0.40	1.00	1.04	0.32	0.78	1.00
gamma-glutamylvaline	Peptide	Gamma-glutamyl Amino Ac	1.06	0.29	0.73	1.00	1.11	0.02	0.48	1.00	1.05	0.33	0.83	1.00	1.04	0.15	0.63	1.00	0.99	0.76	0.92	1.00	0.95	0.27	0.75	1.00
gamma-tocopherol/beta-tocopherol	Cofactors and	Tocopherol Metabolism	1.04	0.45	0.81	1.00	1.08	0.16	0.72	1.00	1.00	0.50	0.89	1.00	1.04	0.58	0.87	1.00	0.96	0.89	0.96	1.00	0.93	0.43	0.83	1.00
gluconate	Xenobiotics	Food Component/Plant	1.01	0.84	0.95	1.00	1.07	0.29	0.78	1.00	0.99	0.75	0.94	1.00	1.06	0.24	0.68	1.00	0.97	0.79	0.93	1.00	0.92	0.07	0.64	1.00
glucose	Carbohydrate	Glycolysis, Gluconeogenesi	1.00	0.98	1.00	1.00	1.02	0.26	0.77	1.00	1.01	0.69	0.94	1.00	1.02	0.26	0.70	1.00	1.01	0.38	0.73	1.00	0.99	0.35	0.80	1.00
glucuronate	Carbohydrate	Aminosugar Metabolism	0.94	0.10	0.51	1.00	0.98	0.50	0.88	1.00	0.95	0.15	0.74	1.00	1.04	0.04	0.50	1.00	1.01	0.40	0.74	1.00	0.97	0.40	0.82	1.00
glu-gly-asn-val	Peptide	Polypeptide	0.82	0.08	0.50	1.00	1.16	0.82	0.95	1.00	0.80	0.21	0.76	1.00	1.41	0.11	0.61	1.00	0.97	0.66	0.88	1.00	0.69	0.49	0.85	1.00
glutamate	Amino Acid	Glutamate Metabolism	1.04	0.70	0.94	1.00	1.19	0.03	0.51	1.00	1.05	0.56	0.89	1.00	1.14	0.03	0.46	1.00	1.01	0.97	0.99	1.00	0.88	0.04	0.57	1.00
glutamine	Amino Acid	Glutamate Metabolism	1.01	0.56	0.88	1.00	1.05	0.08	0.62	1.00	1.05	0.02	0.47	1.00	1.03	0.26	0.70	1.00	1.04	0.11	0.48	1.00	1.00	0.84	0.97	1.00
glycerate	Carbohydrate	Glycolysis, Gluconeogenesi	0.95	0.27	0.71	1.00	0.99	0.64	0.92	1.00	0.94	0.15	0.73	1.00	1.04	0.21	0.67	1.00	0.99	0.56	0.83	1.00	0.95	0.18	0.73	1.00
glycerol	Lipid	Glycerolipid Metabolism	1.23	0.00	0.09	0.72	1.10	0.13	0.69	1.00	1.13	0.07	0.58	1.00	0.90	0.03	0.48	1.00	0.92	0.15	0.52	1.00	1.03	0.39	0.82	1.00
glycerophosphorylcholine (GPC)	Lipid	Phospholipid Metabolism	1.19	0.87	0.97	1.00	0.99	0.27	0.77	1.00	1.07	0.12	0.67	1.00	0.83	0.84	0.96	1.00	0.90	0.09	0.43	1.00	1.08	0.23	0.75	1.00
glycine	Amino Acid	Glycine, Serine and Threoni	1.01	0.63	0.93	1.00	1.07	0.04	0.51	1.00	1.09	0.01	0.38	1.00	1.05	0.13	0.61	1.00	1.07	0.02	0.31	1.00	1.02	0.56	0.88	1.00
glycochenodeoxycholate	Lipid	Primary Bile Acid Metabolis	0.64	0.01	0.20	1.00	0.98	0.12	0.67	1.00	0.77	0.09	0.60	1.00	1.55	0.53	0.84	1.00	1.22	0.09	0.43	1.00	0.79	0.49	0.85	1.00
glycocholate	Lipid	Primary Bile Acid Metabolis	0.72	0.16	0.59	1.00	0.88	0.50	0.88	1.00	0.85	0.39	0.86	1.00	1.23	0.67	0.91	1.00	1.19	0.29	0.67	1.00	0.97	0.27	0.75	1.00
glycocholenate sulfate*	Lipid	Secondary Bile Acid Metabo	1.04	0.72	0.95	1.00	1.03	0.87	0.96	1.00	1.07	0.49	0.89	1.00	1.00	0.92	0.99	1.00	1.03	0.64	0.87	1.00	1.04	0.40	0.82	1.00
glycolithocholate sulfate*	Lipid	Secondary Bile Acid Metabo	0.87	0.07	0.49	1.00	0.77	0.00	0.29	1.00	0.89	0.33	0.83	1.00	0.89	0.52	0.84	1.00	1.03	0.39	0.73	1.00	1.16	0.16	0.73	1.00
glycosyl ceramide (d18:2/24:1, d18:1/24:2)*	Lipid	Hexosylceramides (HCER)	1.03	0.48	0.82	1.00	0.95	0.21	0.76	1.00	1.03	0.35	0.85	1.00	0.92	0.22	0.68	1.00	1.00	0.30	0.67	1.00	1.08	0.08	0.65	1.00
glycosyl-N-palmitoyl-sphingosine (d18:1/16:0)	Lipid	Hexosylceramides (HCER)	1.00	0.70	0.94	1.00	0.97	0.21	0.75	1.00	1.00	0.46	0.89	1.00	0.97	0.32	0.73	1.00	0.99	0.98	1.00	1.00	1.03	0.39	0.82	1.00
glycosyl-N-stearoyl-sphingosine (d18:1/18:0)	Lipid	Hexosylceramides (HCER)	1.11	0.09	0.50	1.00	1.01	0.76	0.94	1.00	1.11	0.05	0.57	1.00	0.91	0.06	0.51	1.00	1.00	0.98	1.00	1.00	1.10	0.13	0.73	1.00
guaiacol sulfate	Xenobiotics	Benzoate Metabolism	1.03	0.84	0.95	1.00	0.88	0.30	0.78	1.00	0.96	0.37	0.85	1.00	0.85	0.22	0.68	1.00	0.94	0.90	0.97	1.00	1.10	0.22	0.74	1.00
guanidinoacetate	Amino Acid	Creatine Metabolism	1.08	0.26	0.71	1.00	1.08	0.16	0.71	1.00	1.11	0.08	0.58	1.00	1.00	0.90	0.98	1.00	1.03	0.56	0.83	1.00	1.03	0.58	0.88	1.00
guanosine	Nucleotide	Purine Metabolism, Guanir	1.20	0.07	0.48	1.00	1.08	0.57	0.89	1.00	1.11	0.12	0.67	1.00	0.90	0.20	0.67	1.00	0.92	0.43	0.75	1.00	1.03	0.48	0.85	1.00
heptenedioate (C7:1-DC)*	Lipid	Fatty Acid, Dicarboxylate	1.13	0.27	0.71	1.00	1.04	0.61	0.91	1.00	1.05	0.37	0.85	1.00	0.92	0.31	0.73	1.00	0.92	0.81	0.93	1.00	1.01	1.00	1.00	1.00
hexadecadienoate (16:2n6)	Lipid	Polyunsaturated Fatty Acid	1.12	0.20	0.64	1.00	1.01	0.97	0.99	1.00	1.00	0.73	0.94	1.00	0.91	0.06	0.51	1.00	0.89	0.12	0.49	1.00	0.99	0.86	0.97	1.00
hexadecanedioate (C16)	Lipid	Fatty Acid, Dicarboxylate	1.03	0.48	0.82	1.00	0.91	0.33	0.79	1.00	0.99	0.78	0.94	1.00	0.88	0.06	0.51	1.00	0.96	0.72	0.89	1.00	1.09	0.06	0.64	1.00
hexadecenedioate (C16:1-DC)*	Lipid	Fatty Acid, Dicarboxylate	0.92	0.27	0.71	1.00	0.90	0.14	0.70	1.00	1.00	0.94	0.99	1.00	0.98	0.78	0.94	1.00	1.09	0.07	0.43	1.00	1.11	0.25	0.75	1.00
hexanoylcarnitine (C6)	Lipid	Fatty Acid Metabolism(Acyl	1.18	0.01	0.20	1.00	1.17	0.01	0.40	1.00	1.17	0.03	0.53	1.00	0.99	0.94	0.99	1.00	0.99	0.75	0.92	1.00	1.00	0.76	0.95	1.00
hexanoylglutamine	Lipid	Fatty Acid Metabolism (Acy	1.13	0.05	0.43	1.00	0.92	0.82	0.95	1.00	0.94	0.70	0.94	1.00	0.82	0.16	0.64	1.00	0.83	0.22	0.62	1.00	1.02	0.82	0.96	1.00
hippurate	Xenobiotics	Benzoate Metabolism	0.85	0.22	0.66	1.00	0.76	0.09	0.65	1.00	0.98	0.25	0.80	1.00	0.89	0.56	0.86	1.00	1.15	0.64	0.87	1.00	1.28	0.38	0.82	1.00
histidine	Amino Acid	Histidine Metabolism	0.97	0.35	0.74	1.00	0.99	0.67	0.92	1.00	1.02	0.32	0.83	1.00	1.02	0.33	0.74	1.00	1.06	0.03	0.31	1.00	1.03	0.05	0.64	1.00
homoarginine	Amino Acid	Urea cycle; Arginine and Pro	1.03	0.81	0.95	1.00	1.02	0.43	0.83	1.00	1.06	0.33	0.83	1.00	1.00	0.90	0.98	1.00	1.03	0.32	0.69	1.00	1.03	0.48	0.85	1.00
homostachydrine*	Xenobiotics	Food Component/Plant	0.99	0.27	0.71	1.00	0.70	0.18	0.73	1.00	0.97	0.84	0.96	1.00	0.71	0.58	0.87	1.00	0.98	0.07	0.41	1.00	1.38	0.26	0.75	1.00
hydroxyasparagine	Amino Acid	Alanine and Aspartate Meta	1.10	0.01	0.20	1.00	1.10	0.01	0.42	1.00	1.11	0.00	0.29	1.00	0.99	0.57	0.87	1.00	1.01	0.69	0.88	1.00	1.02	0.48	0.85	1.00
hydroxy-CMPF*	Lipid	Fatty Acid, Dicarboxylate	0.94	0.37	0.76	1.00	0.87	0.05	0.54	1.00	0.89	0.03	0.47	1.00	0.93	0.01	0.44	1.00	0.95	0.11	0.48	1.00	1.03	0.63	0.90	1.00
hydroxy-N6,N6,N6-trimethyllysine*	Amino Acid	Lysine Metabolism	1.05	0.38	0.77	1.00	1.16	0.07	0.60	1.00	1.16	0.11	0.65	1.00	1.10	0.23	0.68	1.00	1.10	0.67	0.88	1.00	1.00	0.84	0.97	1.00
hydroxypalmitoyl sphingomyelin (d18:1/16:0(O	Lipid	Sphingomyelins	0.98	0.97	0.99	1.00	0.93	0.01	0.39	1.00	0.99	0.78	0.94	1.00	0.95	0.11	0.61	1.00	1.01	0.29	0.67	1.00	1.07	0.02	0.54	1.00
hypotaurine	Amino Acid	Methionine, Cysteine, SAM	0.92	0.30	0.73	1.00	1.06	0.26	0.77	1.00	1.10	0.01	0.39	1.00	1.15	0.01	0.44	1.00	1.19	0.00	0.27	1.00	1.03	0.42	0.82	1.00
hypoxanthine	Nucleotide	Purine Metabolism, (Hypo)	0.99	0.90	0.98	1.00	1.02	0.70	0.93	1.00	1.02	1.00	1.00	1.00	1.03	0.69	0.91	1.00	1.03	0.63	0.87	1.00	1.00	0.67	0.92	1.00
imidazole lactate	Amino Acid	Histidine Metabolism	1.04	0.49	0.83	1.00	1.04	0.25	0.77	1.00	1.06	0.24	0.79	1.00	1.00	0.73	0.93	1.00	1.02	0.35	0.71	1.00	1.01	0.57	0.88	1.00
indoleacetate	Amino Acid	Tryptophan Metabolism	0.88	0.24	0.67	1.00	0.97	0.49	0.87	1.00	0.96	0.92	0.98	1.00	1.11	0.27	0.70	1.00	1.10	0.12	0.49	1.00	0.99	0.94	0.99	1.00
indolelactate	Amino Acid	Tryptophan Metabolism	0.97	0.43	0.80	1.00	1.00	0.97	0.99	1.00	1.02	0.45	0.88	1.00	1.04	0.31	0.73	1.00	1.05	0.11	0.47	1.00	1.01	0.82	0.96	1.00
indolepropionate	Amino Acid	Tryptophan Metabolism	0.98	0.69	0.94	1.00	0.83	0.02	0.42	1.00	0.94	0.54	0.89	1.00	0.84	0.30	0.73	1.00	0.96	0.64	0.87	1.00	1.13	0.35	0.80	1.00
inosine	Nucleotide	Purine Metabolism, (Hypo)	1.18	0.10	0.51	1.00	1.03	1.00	1.00	1.00	1.04	0.84	0.96	1.00	0.87	0.10	0.60	1.00	0.88	0.33	0.69	1.00	1.01	0.57	0.88	1.00
isobutyrylcarnitine (C4)	Amino Acid	Leucine, Isoleucine and Val	0.95	0.39	0.78	1.00	1.04	0.76	0.94	1.00	1.05	0.81	0.95	1.00	1.10	0.39	0.79	1.00	1.11	0.39	0.73	1.00	1.00	0.50	0.86	1.00
isoleucine	Amino Acid	Leucine, Isoleucine and Val	0.96	0.43	0.80	1.00	0.98	0.79	0.94	1.00	1.01	0.53	0.89	1.00	1.02	0.44	0.80	1.00	1.06	0.16	0.55	1.00	1.03	0.15	0.73	1.00
isovalerylcarnitine (C5)	Amino Acid	Leucine, Isoleucine and Val	1.04	0.57	0.89	1.00	1.09	0.32	0.79	1.00	1.00	0.75	0.94	1.00	1.05	0.50	0.84	1.00	0.97	0.73	0.90	1.00	0.92	0.70	0.93	1.00
kynurenate	Amino Acid	Tryptophan Metabolism	1.00	0.34	0.74	1.00	1.01	0.89	0.97	1.00	1.00	0.97	1.00	1.00	1.00	0.90	0.98	1.00	1.00	0.54	0.83	1.00	0.99	0.81	0.96	1.00
kynurenine	Amino Acid	Tryptophan Metabolism	1.01	0.75	0.95	1.00	1.02	0.64	0.92	1.00	1.04	0.32	0.83	1.00	1.01	0.95	0.99	1.00	1.03	0.39	0.73	1.00	1.02	0.67	0.92	1.00

lactate	Carbohydrate	Glycolysis, Gluconeogenesi	0.93	0.07	0.49	1.00	1.02	0.72	0.94	1.00	1.01	0.97	1.00	1.00	1.10	0.06	0.51	1.00	1.09	0.04	0.37	1.00	0.99	0.73	0.93	1.00
lactosyl-N-nervonoyl-sphingosine (d18:1/24:1)*	Lipid	Lactosylceramides (LCER)	1.04	0.60	0.91	1.00	0.92	0.17	0.72	1.00	1.01	0.94	0.99	1.00	0.89	0.06	0.51	1.00	0.98	0.92	0.98	1.00	1.10	0.04	0.60	1.00
lactosyl-N-palmitoyl-sphingosine (d18:1/16:0)	Lipid	Lactosylceramides (LCER)	0.99	0.53	0.86	1.00	0.92	0.04	0.51	1.00	0.99	0.53	0.89	1.00	0.93	0.05	0.51	1.00	1.00	0.87	0.95	1.00	1.07	0.00	0.15	0.46
laurate (12:0)	Lipid	Medium Chain Fatty Acid	1.07	0.26	0.71	1.00	1.00	0.84	0.95	1.00	1.05	0.76	0.94	1.00	0.93	0.15	0.63	1.00	0.98	0.36	0.71	1.00	1.05	0.97	1.00	1.00
laurylcarnitine (C12)	Lipid	Fatty Acid Metabolism(Acyl	1.13	0.08	0.50	1.00	1.09	0.11	0.66	1.00	1.07	0.37	0.85	1.00	0.97	0.70	0.92	1.00	0.95	0.16	0.54	1.00	0.98	0.53	0.87	1.00
leucine	Amino Acid	Leucine, Isoleucine and Val	0.97	0.66	0.93	1.00	0.99	0.79	0.94	1.00	0.99	0.69	0.94	1.00	1.02	0.50	0.84	1.00	1.02	0.33	0.69	1.00	1.00	0.57	0.88	1.00
lignoceroyl sphingomyelin (d18:1/24:0)	Lipid	Sphingomyelins	1.02	0.66	0.93	1.00	0.90	0.10	0.65	1.00	1.04	0.36	0.85	1.00	0.88	0.06	0.51	1.00	1.02	0.44	0.76	1.00	1.15	0.01	0.50	1.00
lignoceroylcarnitine (C24)*	Lipid	Fatty Acid Metabolism(Acyl	1.05	0.97	0.99	1.00	0.94	0.06	0.60	1.00	1.04	0.32	0.83	1.00	0.90	0.03	0.46	1.00	0.99	0.52	0.82	1.00	1.10	0.01	0.50	1.00
linoleate (18:2n6)	Lipid	Polyunsaturated Fatty Acid	1.04	0.30	0.73	1.00	1.02	0.67	0.92	1.00	0.98	0.40	0.86	1.00	0.97	0.23	0.68	1.00	0.93	0.09	0.43	1.00	0.96	0.24	0.75	1.00
linolenate (18:3n3 or 3n6)	Lipid	Polyunsaturated Fatty Acid	1.07	0.67	0.93	1.00	0.98	0.69	0.92	1.00	0.94	0.26	0.81	1.00	0.92	0.07	0.53	1.00	0.88	0.09	0.43	1.00	0.95	0.29	0.75	1.00
linolenoylcarnitine (C18:3)*	Lipid	Fatty Acid Metabolism(Acyl	1.00	0.78	0.95	1.00	0.98	0.48	0.86	1.00	1.01	0.58	0.91	1.00	0.98	0.75	0.93	1.00	1.00	0.87	0.95	1.00	1.03	0.94	0.99	1.00
linoleoyl-arachidonoyl-glycerol (18:2/20:4) [2]*	Lipid	Diacylglycerol	0.86	0.03	0.39	1.00	0.76	0.01	0.39	1.00	0.83	0.03	0.52	1.00	0.88	0.18	0.66	1.00	0.96	0.78	0.93	1.00	1.09	0.44	0.83	1.00
linoleoylcarnitine (C18:2)*	Lipid	Fatty Acid Metabolism(Acyl	1.01	0.89	0.98	1.00	0.99	0.78	0.94	1.00	1.03	0.60	0.92	1.00	0.98	0.81	0.95	1.00	1.02	0.76	0.92	1.00	1.04	0.54	0.87	1.00
linoleoylcholine*	Lipid	Fatty Acid Metabolism (Acy	0.97	0.73	0.95	1.00	0.99	0.87	0.96	1.00	1.12	0.43	0.87	1.00	1.01	0.89	0.98	1.00	1.15	0.05	0.39	1.00	1.14	0.20	0.73	1.00
linoleoyl-linoleoyl-glycerol (18:2/18:2) [1]*	Lipid	Diacylglycerol	0.96	0.78	0.95	1.00	0.88	0.42	0.83	1.00	0.92	0.35	0.85	1.00	0.91	0.21	0.67	1.00	0.95	0.46	0.77	1.00	1.05	0.90	0.98	1.00
lysine	Amino Acid	Lysine Metabolism	1.03	0.34	0.74	1.00	1.05	0.07	0.60	1.00	1.06	0.02	0.47	1.00	1.02	0.12	0.61	1.00	1.04	0.09	0.43	1.00	1.01	0.50	0.86	1.00
malate	Energy	TCA Cycle	0.98	0.73	0.95	1.00	0.97	0.38	0.82	1.00	1.01	0.50	0.89	1.00	0.98	0.61	0.88	1.00	1.02	0.08	0.43	1.00	1.04	0.17	0.73	1.00
mannitol/sorbitol	Carbohydrate	Fructose, Mannose and Gal	1.77	0.63	0.93	1.00	3.56	0.06	0.60	1.00	1.89	0.10	0.63	1.00	2.02	0.02	0.44	1.00	1.07	0.26	0.64	1.00	0.53	0.11	0.71	1.00
mannonate*	Xenobiotics	Food Component/Plant	0.98	0.52	0.85	1.00	1.04	0.44	0.84	1.00	0.97	0.84	0.96	1.00	1.06	0.16	0.64	1.00	0.99	0.97	0.99	1.00	0.93	0.04	0.57	1.00
mannose	Carbohydrate	Fructose, Mannose and Gal	1.05	0.10	0.51	1.00	1.07	0.05	0.56	1.00	1.05	0.11	0.65	1.00	1.02	0.35	0.76	1.00	1.00	0.95	0.99	1.00	0.98	0.17	0.73	1.00
margarate (17:0)	Lipid	Long Chain Fatty Acid	0.99	0.89	0.98	1.00	0.99	0.79	0.94	1.00	0.95	0.44	0.87	1.00	1.01	0.98	0.99	1.00	0.96	0.42	0.74	1.00	0.95	0.36	0.81	1.00
margaroylcarnitine (C17)*	Lipid	Fatty Acid Metabolism(Acyl	1.03	0.46	0.82	1.00	0.97	0.46	0.85	1.00	1.04	0.24	0.78	1.00	0.93	0.16	0.64	1.00	1.00	1.00	1.00	1.00	1.07	0.06	0.64	1.00
methionine	Amino Acid	Methionine, Cysteine, SAM	1.00	0.90	0.98	1.00	1.03	0.45	0.85	1.00	1.06	0.13	0.68	1.00	1.03	0.21	0.67	1.00	1.06	0.09	0.43	1.00	1.03	0.16	0.73	1.00
methionine sulfone	Amino Acid	Methionine, Cysteine, SAM	0.96	0.40	0.79	1.00	0.99	0.86	0.95	1.00	0.97	0.64	0.92	1.00	1.03	0.44	0.80	1.00	1.02	0.46	0.77	1.00	0.98	0.89	0.98	1.00
methionine sulfoxide	Amino Acid	Methionine, Cysteine, SAM	0.82	0.00	0.17	1.00	0.92	0.21	0.75	1.00	0.88	0.06	0.57	1.00	1.12	0.04	0.50	1.00	1.08	0.05	0.39	1.00	0.96	0.73	0.93	1.00
methyl glucopyranoside (alpha + beta)	Xenobiotics	Food Component/Plant	1.15	0.79	0.95	1.00	0.65	0.03	0.51	1.00	0.78	0.35	0.85	1.00	0.56	0.01	0.44	1.00	0.67	0.07	0.41	1.00	1.20	0.08	0.65	1.00
methylsuccinate	Amino Acid	Leucine, Isoleucine and Val	0.85	0.08	0.50	1.00	0.95	0.53	0.89	1.00	0.95	0.76	0.94	1.00	1.12	0.06	0.51	1.00	1.13	0.05	0.39	1.00	1.01	0.82	0.96	1.00
myo-inositol	Lipid	Inositol Metabolism	0.86	0.35	0.74	1.00	0.86	0.24	0.77	1.00	0.93	0.37	0.85	1.00	0.99	0.64	0.89	1.00	1.08	0.11	0.48	1.00	1.09	0.04	0.57	1.00
myristate (14:0)	Lipid	Long Chain Fatty Acid	1.11	0.12	0.54	1.00	1.05	0.43	0.83	1.00	1.02	1.00	1.00	1.00	0.95	0.15	0.63	1.00	0.92	0.10	0.45	1.00	0.96	0.48	0.85	1.00
myristoleate (14:1n5)	Lipid	Long Chain Fatty Acid	1.29	0.00	0.07	0.50	1.13	0.15	0.70	1.00	1.17	0.09	0.60	1.00	0.87	0.02	0.44	1.00	0.90	0.10	0.46	1.00	1.04	0.95	0.99	1.00
myristoleoylcarnitine (C14:1)*	Lipid	Fatty Acid Metabolism(Acyl	1.19	0.04	0.42	1.00	1.04	0.40	0.82	1.00	1.10	0.29	0.82	1.00	0.88	0.04	0.50	1.00	0.92	0.12	0.48	1.00	1.05	0.63	0.90	1.00
myristoyl dihydrosphingomyelin (d18:0/14:0)*	Lipid	Dihydrosphingomyelins	0.96	0.23	0.66	1.00	0.96	0.38	0.82	1.00	0.99	0.98	1.00	1.00	1.00	0.72	0.92	1.00	1.03	0.50	0.80	1.00	1.03	0.25	0.75	1.00
myristoylcarnitine (C14)	Lipid	Fatty Acid Metabolism(Acyl	1.08	0.12	0.55	1.00	1.04	0.40	0.82	1.00	1.04	0.39	0.86	1.00	0.96	0.50	0.84	1.00	0.96	0.21	0.60	1.00	1.00	0.76	0.95	1.00
N,N,N-trimethyl-5-aminovalerate	Amino Acid	Lysine Metabolism	0.98	0.81	0.95	1.00	1.08	0.03	0.51	1.00	1.05	0.38	0.86	1.00	1.10	0.09	0.60	1.00	1.06	0.21	0.60	1.00	0.97	0.66	0.92	1.00
N,N,N-trimethyl-alanylproline betaine (TMAP)	Amino Acid	Urea cycle; Arginine and Pro	1.10	0.04	0.40	1.00	1.06	0.23	0.77	1.00	1.07	0.13	0.69	1.00	0.96	0.23	0.68	1.00	0.97	0.26	0.64	1.00	1.01	0.95	0.99	1.00
N1-Methyl-2-pyridone-5-carboxamide	Cofactors and	Nicotinate and Nicotinami	0.57	0.02	0.33	1.00	0.64	0.30	0.78	1.00	0.56	0.00	0.29	1.00	1.11	0.23	0.68	1.00	0.98	1.00	1.00	1.00	0.88	0.24	0.75	1.00
1-methyladenosine	Nucleotide	Purine Metabolism, Adenir	1.06	0.11	0.52	1.00	1.07	0.02	0.49	1.00	1.04	0.22	0.76	1.00	1.01	0.89	0.98	1.00	0.99	0.95	0.99	1.00	0.98	0.42	0.82	1.00
N1-methylinosine	Nucleotide	Purine Metabolism, (Hypo)	1.09	0.45	0.81	1.00	1.10	0.26	0.77	1.00	1.07	0.32	0.83	1.00	1.02	0.37	0.78	1.00	0.98	1.00	1.00	1.00	0.97	0.52	0.86	1.00
N2,N2-dimethylguanosine	Nucleotide	Purine Metabolism, Guanir	1.05	0.28	0.72	1.00	1.10	0.04	0.51	1.00	1.07	0.21	0.76	1.00	1.05	0.29	0.72	1.00	1.02	0.45	0.77	1.00	0.97	0.43	0.83	1.00
N6,N6,N6-trimethyllysine	Amino Acid	Lysine Metabolism	1.06	0.31	0.73	1.00	1.31	0.07	0.60	1.00	1.40	0.03	0.47	1.00	1.24	0.08	0.58	1.00	1.32	0.53	0.82	1.00	1.06	0.86	0.97	1.00
N6,N6-dimethyllysine	Amino Acid	Lysine Metabolism	1.06	0.33	0.74	1.00	1.12	0.11	0.65	1.00	1.13	0.07	0.58	1.00	1.06	0.14	0.62	1.00	1.07	0.31	0.68	1.00	1.01	0.67	0.92	1.00
N6-acetyllysine	Amino Acid	Lysine Metabolism	0.99	0.84	0.95	1.00	1.01	0.84	0.95	1.00	1.03	0.57	0.90	1.00	1.02	0.49	0.83	1.00	1.04	0.18	0.57	1.00	1.01	0.32	0.78	1.00
N6-carbamoylthreonyladenosine	Nucleotide	Purine Metabolism, Adenir	1.02	0.37	0.76	1.00	1.07	0.04	0.52	1.00	1.03	0.34	0.84	1.00	1.05	0.21	0.67	1.00	1.01	0.61	0.87	1.00	0.96	0.19	0.73	1.00
N6-methyllysine	Amino Acid	Lysine Metabolism	1.09	0.26	0.71	1.00	1.11	0.05	0.53	1.00	1.04	0.52	0.89	1.00	1.02	0.28	0.72	1.00	0.96	0.35	0.71	1.00	0.94	0.01	0.50	1.00
N-acetyl-2-aminooctanoate*	Lipid	Fatty Acid, Amino	0.85	0.86	0.96	1.00	0.86	0.98	0.99	1.00	0.91	0.90	0.98	1.00	1.00	0.86	0.97	1.00	1.07	0.73	0.90	1.00	1.06	0.35	0.80	1.00
N-acetylalanine	Amino Acid	Alanine and Aspartate Meta	0.99	0.60	0.91	1.00	1.04	0.21	0.75	1.00	1.03	0.18	0.76	1.00	1.05	0.02	0.44	1.00	1.04	0.07	0.41	1.00	0.99	0.58	0.88	1.00
N-acetylarginine	Amino Acid	Urea cycle; Arginine and Pro	1.00	0.67	0.93	1.00	1.04	0.76	0.94	1.00	1.08	0.22	0.76	1.00	1.05	0.48	0.82	1.00	1.08	0.07	0.41	1.00	1.03	0.19	0.73	1.00
N-acetylasparagine	Amino Acid	Alanine and Aspartate Meta	1.00	0.54	0.87	1.00	0.97	0.82	0.95	1.00	1.00	0.98	1.00	1.00	0.97	0.94	0.99	1.00	1.00	0.79	0.93	1.00	1.03	0.52	0.86	1.00
N-acetyl-beta-alanine	Nucleotide	Pyrimidine Metabolism, Ur	1.09	0.20	0.64	1.00	1.21	0.00	0.24	1.00	1.28	0.00	0.00	0.00	1.11	0.01	0.44	1.00	1.17	0.00	0.05	0.05	1.05	0.02	0.54	1.00
N-acetylglutamate	Amino Acid	Glutamate Metabolism	0.87	0.02	0.30	1.00	0.93	0.24	0.77	1.00	0.91	0.16	0.75	1.00	1.07	0.24	0.69	1.00	1.05	0.13	0.50	1.00	0.98	0.82	0.96	1.00
N-acetylglutamine	Amino Acid	Glutamate Metabolism	0.90	0.08	0.50	1.00	1.02	0.92	0.98	1.00	0.95	0.21	0.76	1.00	1.13	0.08	0.58	1.00	1.05	0.92	0.98	1.00	0.93	0.08	0.65	1.00
N-acetylglycine	Amino Acid	Glycine, Serine and Threoni	1.11	0.10	0.51	1.00	1.01	0.98	0.99	1.00	1.01	0.89	0.98	1.00	0.91	0.44	0.80	1.00	0.91	0.18	0.57	1.00	1.00	0.72	0.93	1.00
N-acetyl-isoputreanine*	Amino Acid	Polyamine Metabolism	1.09	0.09	0.50	1.00	1.06	0.33	0.79	1.00	1.05	0.10	0.62	1.00	0.97	0.66	0.90	1.00	0.96	0.33	0.69	1.00	0.99	0.90	0.98	1.00
N-acetylleucine	Amino Acid	Leucine, Isoleucine and Val	0.95	0.46	0.82	1.00	0.99	0.45	0.85	1.00	0.99	0.84	0.96	1.00	1.04	0.66	0.90	1.00	1.04	0.60	0.86	1.00	1.00	0.42	0.82	1.00

N-acetylmethionine	Amino Acid	Methionine, Cysteine, SAM	0.99	0.54	0.87	1.00	1.06	0.08	0.62	1.00	1.07	0.01	0.39	1.00	1.08	0.01	0.44	1.00	1.09	0.00	0.24	1.00	1.01	0.69	0.93	1.00
N-acetylputrescine	Amino Acid	Polyamine Metabolism	1.06	0.24	0.67	1.00	1.05	0.15	0.70	1.00	1.08	0.09	0.60	1.00	0.99	0.87	0.97	1.00	1.02	0.23	0.62	1.00	1.02	0.18	0.73	1.00
N-acetylserine	Amino Acid	Glycine, Serine and Threoni	0.99	0.64	0.93	1.00	1.04	0.43	0.83	1.00	1.02	0.61	0.92	1.00	1.05	0.20	0.67	1.00	1.02	0.81	0.93	1.00	0.98	0.40	0.82	1.00
N-acetyltaurine	Amino Acid	Methionine, Cysteine, SAM	0.95	0.33	0.74	1.00	1.00	0.98	0.99	1.00	0.97	0.39	0.86	1.00	1.05	0.20	0.67	1.00	1.02	0.42	0.74	1.00	0.97	0.92	0.98	1.00
N-acetylthreonine	Amino Acid	Glycine, Serine and Threoni	1.03	0.67	0.93	1.00	1.01	0.73	0.94	1.00	1.06	0.28	0.82	1.00	0.98	0.76	0.93	1.00	1.04	0.49	0.79	1.00	1.06	0.18	0.73	1.00
N-acetyltryptophan	Amino Acid	Tryptophan Metabolism	0.97	0.76	0.95	1.00	1.02	0.92	0.98	1.00	1.11	0.04	0.55	1.00	1.05	0.22	0.68	1.00	1.14	0.00	0.31	1.00	1.09	0.08	0.65	1.00
N-acetylvaline	Amino Acid	Leucine, Isoleucine and Val	1.00	0.94	0.99	1.00	1.03	0.42	0.83	1.00	1.05	0.21	0.76	1.00	1.03	0.22	0.68	1.00	1.05	0.04	0.37	1.00	1.02	0.37	0.81	1.00
N-behenoyl-sphingadienine (d18:2/22:0)*	Lipid	Ceramides	1.12	0.15	0.58	1.00	1.05	0.57	0.89	1.00	1.04	0.64	0.92	1.00	0.94	0.22	0.68	1.00	0.93	0.04	0.37	1.00	0.99	0.67	0.92	1.00
N-delta-acetylornithine	Amino Acid	Urea cycle; Arginine and Pro	0.89	0.76	0.95	1.00	0.88	0.56	0.89	1.00	0.90	0.73	0.94	1.00	0.98	0.95	0.99	1.00	1.01	0.82	0.94	1.00	1.03	0.84	0.97	1.00
N-formylmethionine	Amino Acid	Methionine, Cysteine, SAM	0.98	0.53	0.86	1.00	1.00	0.97	0.99	1.00	1.03	0.22	0.76	1.00	1.03	0.19	0.67	1.00	1.06	0.06	0.39	1.00	1.03	0.25	0.75	1.00
N-methylpipecolate	Xenobiotics	Bacterial/Fungal	1.01	0.53	0.86	1.00	1.09	0.15	0.70	1.00	1.06	0.17	0.75	1.00	1.08	0.01	0.44	1.00	1.06	0.01	0.31	1.00	0.97	0.42	0.82	1.00
N-methylproline	Amino Acid	Urea cycle; Arginine and Pro	0.94	0.75	0.95	1.00	0.62	0.40	0.82	1.00	0.91	0.42	0.87	1.00	0.65	0.46	0.82	1.00	0.96	0.86	0.95	1.00	1.47	0.69	0.93	1.00
N-oleoylserine	Lipid	Endocannabinoid	0.89	0.10	0.51	1.00	1.00	0.79	0.94	1.00	0.90	0.19	0.76	1.00	1.13	0.05	0.51	1.00	1.01	0.89	0.96	1.00	0.90	0.07	0.64	1.00
N-oleoyltaurine	Lipid	Endocannabinoid	0.86	0.15	0.58	1.00	0.84	0.27	0.77	1.00	0.79	0.02	0.47	1.00	0.98	0.94	0.99	1.00	0.92	0.27	0.64	1.00	0.94	0.44	0.83	1.00
nonadecanoate (19:0)	Lipid	Long Chain Fatty Acid	0.90	0.12	0.54	1.00	0.94	0.50	0.88	1.00	0.90	0.16	0.75	1.00	1.04	0.35	0.76	1.00	1.01	0.76	0.92	1.00	0.97	0.58	0.88	1.00
nonanoylcarnitine (C9)	Lipid	Fatty Acid Metabolism(Acyl	1.10	0.16	0.59	1.00	1.02	0.57	0.89	1.00	1.09	0.16	0.75	1.00	0.92	0.43	0.79	1.00	0.98	0.79	0.93	1.00	1.07	0.21	0.73	1.00
N-palmitoylglycine	Lipid	Fatty Acid Metabolism(Acyl	0.93	0.24	0.68	1.00	0.96	0.73	0.94	1.00	0.91	0.18	0.76	1.00	1.03	0.39	0.79	1.00	0.98	0.46	0.77	1.00	0.94	0.25	0.75	1.00
N-palmitoyl-heptadecasphingosine (d17:1/16:0)	Lipid	Ceramides	1.00	0.89	0.98	1.00	0.95	0.18	0.73	1.00	0.97	0.37	0.85	1.00	0.96	0.29	0.72	1.00	0.97	0.12	0.48	1.00	1.01	0.84	0.97	1.00
N-palmitoyl-sphingadienine (d18:2/16:0)*	Lipid	Ceramides	1.10	0.06	0.47	1.00	1.08	0.24	0.77	1.00	1.03	0.63	0.92	1.00	0.98	0.76	0.93	1.00	0.93	0.04	0.36	1.00	0.95	0.44	0.83	1.00
N-palmitoyl-sphinganine (d18:0/16:0)	Lipid	Dihydroceramides	0.90	0.12	0.54	1.00	0.85	0.01	0.42	1.00	0.90	0.27	0.82	1.00	0.94	0.24	0.68	1.00	1.00	0.69	0.88	1.00	1.06	0.13	0.73	1.00
N-palmitoyl-sphingosine (d18:1/16:0)	Lipid	Ceramides	1.00	0.82	0.95	1.00	0.93	0.09	0.65	1.00	0.95	0.15	0.73	1.00	0.93	0.30	0.73	1.00	0.95	0.22	0.62	1.00	1.02	0.61	0.90	1.00
N-stearoyl-sphingadienine (d18:2/18:0)*	Lipid	Ceramides	1.02	1.00	1.00	1.00	0.97	0.20	0.75	1.00	0.93	0.13	0.68	1.00	0.95	0.30	0.73	1.00	0.91	0.03	0.35	1.00	0.96	0.39	0.82	1.00
N-stearoyl-sphingosine (d18:1/18:0)*	Lipid	Ceramides	1.04	0.76	0.95	1.00	0.99	0.36	0.82	1.00	0.96	0.27	0.82	1.00	0.95	0.40	0.79	1.00	0.92	0.05	0.39	1.00	0.96	0.27	0.75	1.00
octadecadienedioate (C18:2-DC)*	Lipid	Fatty Acid, Dicarboxylate	0.89	0.09	0.50	1.00	1.12	0.33	0.79	1.00	0.85	0.44	0.87	1.00	1.25	0.72	0.92	1.00	0.95	0.32	0.69	1.00	0.76	0.38	0.82	1.00
octadecanedioate (C18)	Lipid	Fatty Acid, Dicarboxylate	0.98	0.76	0.95	1.00	0.89	0.12	0.67	1.00	1.01	0.82	0.96	1.00	0.90	0.09	0.60	1.00	1.03	0.69	0.88	1.00	1.14	0.06	0.64	1.00
octadecanedioylcarnitine (C18-DC)*	Lipid	Fatty Acid Metabolism(Acyl	0.98	0.84	0.95	1.00	0.95	0.36	0.82	1.00	1.07	0.20	0.76	1.00	0.97	0.21	0.68	1.00	1.09	0.09	0.43	1.00	1.12	0.01	0.50	1.00
octadecenedioate (C18:1-DC)*	Lipid	Fatty Acid, Dicarboxylate	0.80	0.05	0.43	1.00	0.79	0.05	0.54	1.00	0.85	0.23	0.77	1.00	0.99	0.69	0.91	1.00	1.06	0.09	0.43	1.00	1.08	0.21	0.73	1.00
octadecenedioylcarnitine (C18:1-DC)*	Lipid	Fatty Acid Metabolism(Acyl	0.93	0.44	0.80	1.00	0.89	0.10	0.65	1.00	0.95	0.39	0.86	1.00	0.95	0.30	0.73	1.00	1.01	0.70	0.89	1.00	1.07	0.21	0.73	1.00
octanoylcarnitine (C8)	Lipid	Fatty Acid Metabolism(Acyl	1.12	0.10	0.51	1.00	1.06	0.26	0.77	1.00	1.08	0.18	0.76	1.00	0.95	0.50	0.84	1.00	0.97	0.40	0.74	1.00	1.02	0.79	0.96	1.00
oleate/vaccenate (18:1)	Lipid	Long Chain Fatty Acid	1.08	0.05	0.43	1.00	1.04	0.52	0.88	1.00	1.00	0.82	0.96	1.00	0.96	0.13	0.62	1.00	0.92	0.02	0.31	1.00	0.95	0.21	0.73	1.00
oleoyl ethanolamide	Lipid	Endocannabinoid	0.95	0.21	0.66	1.00	1.02	0.69	0.92	1.00	0.92	0.20	0.76	1.00	1.08	0.16	0.64	1.00	0.97	0.69	0.88	1.00	0.91	0.16	0.73	1.00
oleoylcarnitine (C18)	Lipid	Fatty Acid Metabolism(Acyl	1.05	0.10	0.51	1.00	1.00	1.00	1.00	1.00	1.02	0.61	0.92	1.00	0.95	0.27	0.70	1.00	0.97	0.32	0.69	1.00	1.02	0.70	0.93	1.00
oleoylcholine	Lipid	Fatty Acid Metabolism (Acy	1.01	0.79	0.95	1.00	1.00	0.94	0.98	1.00	1.12	0.40	0.86	1.00	0.99	0.78	0.94	1.00	1.10	0.13	0.50	1.00	1.11	0.34	0.80	1.00
oleoyl-linoleoyl-glycerol (18:1/18:2) [1]	Lipid	Diacylglycerol	0.97	0.82	0.95	1.00	0.88	0.46	0.85	1.00	0.90	0.44	0.87	1.00	0.90	0.19	0.67	1.00	0.92	0.29	0.67	1.00	1.02	0.92	0.98	1.00
oleoyl-linoleoyl-glycerol (18:1/18:2) [2]	Lipid	Diacylglycerol	0.96	0.98	1.00	1.00	0.83	0.24	0.77	1.00	0.90	0.40	0.86	1.00	0.87	0.05	0.51	1.00	0.93	0.53	0.82	1.00	1.08	0.45	0.83	1.00
oleoyl-oleoyl-glycerol (18:1/18:1) [1]*	Lipid	Diacylglycerol	1.01	0.42	0.79	1.00	0.88	0.36	0.82	1.00	0.95	0.86	0.96	1.00	0.87	0.02	0.44	1.00	0.94	0.54	0.83	1.00	1.08	0.17	0.73	1.00
oleoyl-oleoyl-glycerol (18:1/18:1) [2]*	Lipid	Diacylglycerol	1.02	0.45	0.81	1.00	0.90	0.66	0.92	1.00	0.95	0.92	0.98	1.00	0.88	0.05	0.51	1.00	0.93	0.42	0.74	1.00	1.06	0.33	0.79	1.00
ornithine	Amino Acid	Urea cycle; Arginine and Pro	0.99	0.53	0.86	1.00	1.02	0.52	0.88	1.00	1.02	0.43	0.87	1.00	1.03	0.10	0.60	1.00	1.04	0.10	0.45	1.00	1.00	0.97	1.00	1.00
orotate	Nucleotide	Pyrimidine Metabolism, Or	0.90	0.02	0.30	1.00	1.02	0.72	0.94	1.00	0.97	0.36	0.85	1.00	1.14	0.04	0.50	1.00	1.08	0.06	0.39	1.00	0.95	0.31	0.78	1.00
orotidine	Nucleotide	Pyrimidine Metabolism, Or	0.98	0.94	0.99	1.00	1.09	0.07	0.60	1.00	1.01	0.75	0.94	1.00	1.12	0.00	0.44	1.00	1.03	0.26	0.64	1.00	0.92	0.06	0.64	1.00
O-sulfo-L-tyrosine	Xenobiotics	Chemical	1.06	0.18	0.61	1.00	1.08	0.10	0.65	1.00	1.06	0.07	0.58	1.00	1.02	0.35	0.76	1.00	1.00	0.39	0.73	1.00	0.98	0.53	0.87	1.00
oxalate (ethanedioate)	Cofactors and	Ascorbate and Aldarate Met	1.02	0.32	0.73	1.00	0.95	0.11	0.65	1.00	0.99	0.50	0.89	1.00	0.93	0.04	0.50	1.00	0.97	0.37	0.72	1.00	1.04	0.14	0.73	1.00
palmitate (16:0)	Lipid	Long Chain Fatty Acid	1.04	0.38	0.77	1.00	1.03	0.45	0.85	1.00	0.99	0.66	0.93	1.00	0.99	0.42	0.79	1.00	0.96	0.24	0.63	1.00	0.97	0.26	0.75	1.00
palmitoleate (16:1n7)	Lipid	Long Chain Fatty Acid	1.26	0.01	0.20	1.00	1.10	0.24	0.77	1.00	1.09	0.27	0.82	1.00	0.88	0.02	0.44	1.00	0.87	0.03	0.32	1.00	0.99	0.50	0.86	1.00
palmitoleoylcarnitine (C16:1)*	Lipid	Fatty Acid Metabolism(Acyl	1.17	0.00	0.17	1.00	1.05	0.29	0.78	1.00	1.10	0.11	0.65	1.00	0.90	0.08	0.58	1.00	0.94	0.10	0.46	1.00	1.04	0.61	0.90	1.00
palmitoleoyl-linoleoyl-glycerol (16:1/18:2) [1]*	Lipid	Diacylglycerol	1.01	0.70	0.94	1.00	0.91	0.79	0.94	1.00	0.94	0.63	0.92	1.00	0.91	0.18	0.67	1.00	0.93	0.13	0.50	1.00	1.03	0.76	0.95	1.00
palmitoloelycholine	Lipid	Fatty Acid Metabolism (Acy	0.99	0.61	0.92	1.00	1.00	0.54	0.89	1.00	1.17	0.29	0.82	1.00	1.00	0.94	0.99	1.00	1.18	0.31	0.68	1.00	1.17	0.33	0.79	1.00
palmitoyl dihydrosphingomyelin (d18:0/16:0)*	Lipid	Dihydrosphingomyelins	0.96	0.21	0.66	1.00	0.95	0.08	0.62	1.00	1.00	0.95	0.99	1.00	0.98	1.00	1.00	1.00	1.04	0.26	0.64	1.00	1.05	0.09	0.68	1.00
palmitoyl ethanolamide	Lipid	Endocannabinoid	0.94	0.24	0.67	1.00	0.95	0.32	0.79	1.00	0.94	0.28	0.82	1.00	1.01	0.92	0.99	1.00	1.00	0.79	0.93	1.00	0.99	0.54	0.87	1.00
palmitoyl sphingomyelin (d18:1/16:0)	Lipid	Sphingomyelins	0.97	0.15	0.58	1.00	0.95	0.03	0.51	1.00	0.99	0.94	0.99	1.00	0.98	0.69	0.91	1.00	1.03	0.11	0.48	1.00	1.05	0.04	0.60	1.00
palmitoylcarnitine (C16)	Lipid	Fatty Acid Metabolism(Acyl	1.06	0.08	0.50	1.00	0.98	0.53	0.89	1.00	1.05	0.18	0.76	1.00	0.93	0.06	0.51	1.00	1.00	0.98	1.00	1.00	1.08	0.07	0.65	1.00
palmitoylcholine	Lipid	Fatty Acid Metabolism (Acy	0.96	0.82	0.95	1.00	0.96	0.89	0.97	1.00	1.08	0.64	0.92	1.00	1.00	0.97	0.99	1.00	1.12	0.10	0.45	1.00	1.13	0.24	0.75	1.00
palmitoyl-docosahexaenoyl-glycerol (16:0/22:6)	Lipid	Diacylglycerol	0.91	0.64	0.93	1.00	0.81	0.02	0.42	1.00	0.89	0.27	0.82	1.00	0.89	0.04	0.50	1.00	0.97	0.35	0.71	1.00	1.09	0.09	0.68	1.00
palmitoyl-myristoyl-glycerol (16:0/14:0) [1]*	Lipid	Diacylglycerol	0.81	0.17	0.60	1.00	0.89	0.70	0.93	1.00	0.77	0.20	0.76	1.00	1.11	0.54	0.85	1.00	0.95	0.84	0.94	1.00	0.86	0.87	0.97	1.00

palmitoyl-oleoyl-glycerol (16:0/18:1) [1]*	Lipid	Diacylglycerol	0.93	0.92	0.99	1.00	0.86	0.26	0.77	1.00	0.88	0.21	0.76	1.00	0.92	0.07	0.55	1.00	0.95	0.64	0.87	1.00	1.03	0.22	0.74	1.00
palmitoyl-oleoyl-glycerol (16:0/18:1) [2]*	Lipid	Diacylglycerol	0.93	0.97	0.99	1.00	0.88	0.40	0.82	1.00	0.89	0.30	0.82	1.00	0.95	0.10	0.60	1.00	0.96	0.64	0.87	1.00	1.01	0.32	0.78	1.00
palmitoyl-palmitoyl-glycerol (16:0/16:0) [2]*	Lipid	Diacylglycerol	0.85	0.23	0.66	1.00	0.92	0.16	0.72	1.00	0.86	0.05	0.57	1.00	1.09	0.89	0.98	1.00	1.01	0.84	0.94	1.00	0.93	0.67	0.92	1.00
pantothenate (Vitamin B5)	Cofactors and	Pantothenate and CoA Meta	0.96	0.16	0.59	1.00	1.01	0.16	0.72	1.00	0.98	0.76	0.94	1.00	1.05	0.42	0.79	1.00	1.03	0.22	0.62	1.00	0.98	1.00	1.00	1.00
p-cresol sulfate	Xenobiotics	Benzoate Metabolism	0.83	0.17	0.60	1.00	0.95	0.76	0.94	1.00	1.02	0.38	0.86	1.00	1.15	0.07	0.55	1.00	1.24	0.04	0.36	1.00	1.08	0.29	0.75	1.00
pelargonate (9:0)	Lipid	Medium Chain Fatty Acid	0.92	0.50	0.84	1.00	0.94	0.75	0.94	1.00	0.95	0.94	0.99	1.00	1.02	0.67	0.91	1.00	1.04	0.38	0.73	1.00	1.01	0.69	0.93	1.00
pentadecanoate (15:0)	Lipid	Long Chain Fatty Acid	1.04	0.79	0.95	1.00	1.02	0.49	0.87	1.00	1.00	0.81	0.95	1.00	0.98	0.69	0.91	1.00	0.96	0.45	0.77	1.00	0.98	0.73	0.93	1.00
perfluorooctanesulfonate (PFOS)	Xenobiotics	Chemical	0.87	0.00	0.07	0.50	1.04	0.02	0.42	1.00	1.06	0.00	0.29	1.00	1.20	0.15	0.63	1.00	1.22	0.84	0.94	1.00	1.02	0.53	0.87	1.00
perfluorooctanoate (PFOA)*	Xenobiotics	Chemical	0.95	0.03	0.40	1.00	0.93	0.01	0.42	1.00	0.91	0.00	0.24	1.00	0.98	0.31	0.73	1.00	0.95	0.09	0.43	1.00	0.98	0.69	0.93	1.00
phenol sulfate	Amino Acid	Tyrosine Metabolism	1.08	0.48	0.82	1.00	0.94	0.43	0.83	1.00	1.15	0.34	0.84	1.00	0.87	0.30	0.73	1.00	1.07	0.36	0.71	1.00	1.23	0.34	0.80	1.00
phenylacetylglutamine	Peptide	Acetylated Peptides	0.93	0.61	0.92	1.00	1.01	0.84	0.95	1.00	1.10	0.09	0.60	1.00	1.08	0.24	0.68	1.00	1.18	0.02	0.31	1.00	1.09	0.20	0.73	1.00
phenylalanine	Amino Acid	Phenylalanine Metabolism	0.98	0.72	0.95	1.00	1.00	0.75	0.94	1.00	1.01	0.45	0.88	1.00	1.02	0.38	0.79	1.00	1.03	0.18	0.57	1.00	1.01	0.56	0.88	1.00
phenyllactate (PLA)	Amino Acid	Phenylalanine Metabolism	0.90	0.01	0.20	1.00	0.96	0.08	0.62	1.00	0.99	0.76	0.94	1.00	1.07	0.10	0.60	1.00	1.10	0.01	0.31	1.00	1.04	0.05	0.64	1.00
phenylpyruvate	Amino Acid	Phenylalanine Metabolism	0.94	0.42	0.79	1.00	0.95	0.30	0.78	1.00	0.99	0.78	0.94	1.00	1.01	0.34	0.75	1.00	1.05	0.27	0.64	1.00	1.04	0.58	0.88	1.00
phosphoethanolamine (PE)	Lipid	Phospholipid Metabolism	0.95	0.40	0.79	1.00	1.02	0.73	0.94	1.00	1.08	0.09	0.60	1.00	1.07	0.39	0.79	1.00	1.14	0.02	0.31	1.00	1.06	0.21	0.73	1.00
phytanate	Xenobiotics	Food Component/Plant	0.82	0.21	0.65	1.00	0.98	0.66	0.92	1.00	0.99	0.92	0.98	1.00	1.19	0.18	0.66	1.00	1.21	0.07	0.41	1.00	1.02	0.90	0.98	1.00
pipecolate	Amino Acid	Lysine Metabolism	0.77	0.02	0.31	1.00	1.10	0.97	0.99	1.00	0.87	0.69	0.94	1.00	1.43	0.05	0.51	1.00	1.14	0.02	0.31	1.00	0.79	1.00	1.00	1.00
pregnen-diol disulfate*	Lipid	Pregnenolone Steroids	1.03	0.95	0.99	1.00	0.99	0.79	0.94	1.00	1.06	0.57	0.90	1.00	0.97	0.50	0.84	1.00	1.03	0.63	0.87	1.00	1.07	0.42	0.82	1.00
pregnenediol sulfate (C21H34O5S)*	Lipid	Pregnenolone Steroids	0.97	0.78	0.95	1.00	0.96	0.32	0.79	1.00	1.02	0.70	0.94	1.00	0.98	0.86	0.97	1.00	1.05	0.19	0.59	1.00	1.07	0.10	0.68	1.00
pregnenetriol disulfate*	Lipid	Pregnenolone Steroids	1.02	0.70	0.94	1.00	1.01	0.64	0.92	1.00	1.06	0.72	0.94	1.00	0.99	0.94	0.99	1.00	1.04	0.78	0.93	1.00	1.04	1.00	1.00	1.00
pregnenetriol sulfate*	Lipid	Pregnenolone Steroids	1.02	0.64	0.93	1.00	1.00	0.98	0.99	1.00	1.07	0.16	0.75	1.00	0.97	0.97	0.99	1.00	1.04	0.26	0.64	1.00	1.07	0.23	0.75	1.00
prolylhydroxyproline	Amino Acid	Urea cycle; Arginine and Pro	1.01	0.90	0.98	1.00	1.02	0.78	0.94	1.00	1.09	0.48	0.89	1.00	1.00	0.76	0.93	1.00	1.08	0.53	0.82	1.00	1.07	0.48	0.85	1.00
proline	Amino Acid	Urea cycle; Arginine and Pro	0.95	0.23	0.66	1.00	1.00	0.35	0.82	1.00	1.02	0.20	0.76	1.00	1.06	0.01	0.44	1.00	1.08	0.02	0.31	1.00	1.02	0.78	0.95	1.00
propionylcarnitine (C3)	Lipid	Fatty Acid Metabolism (also	0.97	0.46	0.82	1.00	1.07	0.28	0.77	1.00	1.03	0.66	0.93	1.00	1.10	0.09	0.60	1.00	1.06	0.36	0.71	1.00	0.96	1.00	1.00	1.00
propionylglycine (C3)	Lipid	Fatty Acid Metabolism (also	0.83	0.10	0.51	1.00	0.96	0.70	0.93	1.00	1.00	0.60	0.92	1.00	1.16	0.05	0.51	1.00	1.20	0.01	0.31	1.00	1.04	0.22	0.74	1.00
propyl 4-hydroxybenzoate sulfate	Xenobiotics	Benzoate Metabolism	0.82	0.01	0.26	1.00	1.01	0.92	0.98	1.00	0.86	0.05	0.57	1.00	1.23	0.03	0.46	1.00	1.05	0.76	0.92	1.00	0.85	0.17	0.73	1.00
pseudouridine	Nucleotide	Pyrimidine Metabolism, Ur	1.03	0.31	0.73	1.00	1.03	0.50	0.88	1.00	1.02	0.36	0.85	1.00	1.00	0.82	0.95	1.00	0.99	0.87	0.95	1.00	1.00	0.79	0.96	1.00
pyridoxate	Cofactors and	Vitamin B6 Metabolism	0.58	0.00	0.20	1.00	0.68	0.21	0.75	1.00	0.64	0.16	0.75	1.00	1.16	0.23	0.68	1.00	1.10	0.04	0.36	1.00	0.95	0.86	0.97	1.00
pyroglutamine*	Amino Acid	Glutamate Metabolism	1.08	0.29	0.73	1.00	1.12	0.03	0.51	1.00	1.11	0.02	0.47	1.00	1.04	0.61	0.88	1.00	1.03	0.82	0.94	1.00	0.99	0.86	0.97	1.00
pyruvate	Carbohydrate	Glycolysis, Gluconeogenesi	1.05	0.54	0.87	1.00	0.98	0.63	0.91	1.00	1.01	0.95	0.99	1.00	0.93	0.18	0.66	1.00	0.97	0.64	0.87	1.00	1.04	0.92	0.98	1.00
quinate	Xenobiotics	Food Component/Plant	0.71	0.01	0.20	1.00	0.89	0.44	0.84	1.00	0.91	0.63	0.92	1.00	1.25	0.02	0.44	1.00	1.28	0.03	0.35	1.00	1.03	0.90	0.98	1.00
quinolinate	Cofactors and	Nicotinate and Nicotinamid	1.03	0.81	0.95	1.00	1.20	0.16	0.72	1.00	1.05	0.67	0.93	1.00	1.17	0.02	0.44	1.00	1.02	0.70	0.89	1.00	0.87	0.11	0.70	1.00
retinol (Vitamin A)	Cofactors and	Vitamin A Metabolism	0.98	0.54	0.87	1.00	0.98	0.92	0.98	1.00	0.99	0.89	0.98	1.00	1.00	0.94	0.99	1.00	1.02	1.00	1.00	1.00	1.01	0.72	0.93	1.00
ribitol	Carbohydrate	Pentose Metabolism	0.96	0.42	0.79	1.00	1.00	0.90	0.98	1.00	0.95	0.52	0.89	1.00	1.04	0.19	0.67	1.00	1.00	0.95	0.99	1.00	0.96	0.31	0.78	1.00
ribonate (ribonolactone)	Carbohydrate	Pentose Metabolism	1.00	0.69	0.94	1.00	1.05	0.56	0.89	1.00	0.98	0.66	0.93	1.00	1.05	0.43	0.79	1.00	0.98	0.84	0.94	1.00	0.93	0.40	0.82	1.00
salicylate	Xenobiotics	Drug - Topical Agents	0.37	0.98	1.00	1.00	1.63	0.58	0.90	1.00	0.26	0.39	0.86	1.00	4.47	0.89	0.98	1.00	0.70	0.92	0.98	1.00	0.16	0.45	0.83	1.00
sebacate (C10-DC)	Lipid	Fatty Acid, Dicarboxylate	1.09	0.22	0.66	1.00	0.99	0.82	0.95	1.00	1.12	0.12	0.65	1.00	0.91	0.37	0.78	1.00	1.03	0.63	0.87	1.00	1.13	0.18	0.73	1.00
serine	Amino Acid	Glycine, Serine and Threoni	0.99	0.67	0.93	1.00	1.03	0.31	0.79	1.00	1.05	0.05	0.57	1.00	1.04	0.12	0.61	1.00	1.06	0.01	0.31	1.00	1.02	0.22	0.74	1.00
serotonin	Amino Acid	Tryptophan Metabolism	1.02	0.75	0.95	1.00	1.05	0.56	0.89	1.00	1.06	0.21	0.76	1.00	1.02	0.72	0.92	1.00	1.04	0.34	0.70	1.00	1.01	0.48	0.85	1.00
S-methylcysteine	Amino Acid	Methionine, Cysteine, SAM	0.95	0.21	0.66	1.00	1.12	0.02	0.46	1.00	0.96	0.24	0.79	1.00	1.19	0.26	0.70	1.00	1.02	0.78	0.93	1.00	0.86	0.52	0.86	1.00
S-methylcysteine sulfoxide	Amino Acid	Methionine, Cysteine, SAM	0.93	0.24	0.67	1.00	1.18	0.02	0.49	1.00	1.00	0.06	0.57	1.00	1.27	0.37	0.78	1.00	1.08	0.63	0.87	1.00	0.85	0.84	0.97	1.00
sphinganine-1-phosphate	Lipid	Sphingolipid Synthesis	0.96	0.23	0.66	1.00	1.03	0.60	0.91	1.00	1.02	0.89	0.98	1.00	1.07	0.11	0.61	1.00	1.06	0.44	0.76	1.00	0.99	0.70	0.93	1.00
sphingomyelin (d17:1/14:0, d16:1/15:0)*	Lipid	Sphingomyelins	0.96	0.22	0.66	1.00	0.92	0.11	0.66	1.00	1.00	0.61	0.92	1.00	0.95	0.64	0.89	1.00	1.04	0.54	0.83	1.00	1.09	0.10	0.68	1.00
sphingomyelin (d17:1/16:0, d18:1/15:0, d16:1	Lipid	Sphingomyelins	0.97	0.38	0.77	1.00	0.98	0.53	0.89	1.00	1.00	0.95	0.99	1.00	1.01	0.63	0.88	1.00	1.03	0.28	0.65	1.00	1.02	0.17	0.73	1.00
sphingomyelin (d17:2/16:0, d18:2/15:0)*	Lipid	Sphingomyelins	0.98	0.52	0.85	1.00	0.92	0.04	0.51	1.00	0.99	0.95	0.99	1.00	0.94	0.33	0.74	1.00	1.00	0.95	0.99	1.00	1.07	0.11	0.70	1.00
sphingomyelin (d18:0/18:0, d19:0/17:0)*	Lipid	Dihydrosphingomyelins	0.91	0.17	0.60	1.00	0.85	0.02	0.42	1.00	0.91	0.42	0.87	1.00	0.94	0.18	0.66	1.00	1.00	0.66	0.88	1.00	1.07	0.10	0.68	1.00
sphingomyelin (d18:0/20:0, d16:0/22:0)*	Lipid	Dihydrosphingomyelins	0.91	0.45	0.81	1.00	0.83	0.05	0.54	1.00	0.94	0.82	0.96	1.00	0.91	0.16	0.64	1.00	1.04	0.84	0.94	1.00	1.14	0.02	0.50	1.00
sphingomyelin (d18:1/14:0, d16:1/16:0)*	Lipid	Sphingomyelins	0.99	0.57	0.89	1.00	0.97	0.46	0.85	1.00	1.00	0.70	0.94	1.00	0.98	0.61	0.88	1.00	1.02	0.53	0.82	1.00	1.03	0.04	0.57	1.00
sphingomyelin (d18:1/17:0, d17:1/18:0, d19:1	Lipid	Sphingomyelins	0.98	0.35	0.74	1.00	0.96	0.08	0.62	1.00	0.98	0.56	0.89	1.00	0.98	0.42	0.79	1.00	1.01	0.84	0.94	1.00	1.03	0.21	0.73	1.00
sphingomyelin (d18:1/18:1, d18:2/18:0)	Lipid	Sphingomyelins	0.97	0.46	0.82	1.00	0.96	0.39	0.82	1.00	0.98	0.53	0.89	1.00	0.99	0.72	0.92	1.00	1.01	0.53	0.82	1.00	1.02	0.43	0.83	1.00
sphingomyelin (d18:1/19:0, d19:1/18:0)*	Lipid	Sphingomyelins	0.99	0.37	0.76	1.00	0.95	0.11	0.65	1.00	1.00	0.89	0.98	1.00	0.96	0.43	0.79	1.00	1.01	0.56	0.83	1.00	1.05	0.04	0.57	1.00
sphingomyelin (d18:1/20:0, d16:1/22:0)*	Lipid	Sphingomyelins	1.00	0.95	0.99	1.00	0.97	0.27	0.77	1.00	1.01	0.54	0.89	1.00	0.97	0.38	0.79	1.00	1.02	0.66	0.88	1.00	1.05	0.01	0.50	1.00
sphingomyelin (d18:1/20:1, d18:2/20:0)*	Lipid	Sphingomyelins	0.96	0.36	0.76	1.00	0.95	0.12	0.67	1.00	0.97	0.44	0.87	1.00	0.99	1.00	1.00	1.00	1.01	0.92	0.98	1.00	1.02	0.67	0.92	1.00
sphingomyelin (d18:1/20:2, d18:2/20:1, d16:1	Lipid	Sphingomyelins	1.04	0.37	0.76	1.00	1.01	0.82	0.95	1.00	1.01	0.64	0.92	1.00	0.97	0.52	0.84	1.00	0.97	0.38	0.73	1.00	1.00	0.69	0.93	1.00

sphingomyelin (d18:1/21:0, d17:1/22:0, d16:1	Lipid	Sphingomyelins	1.02	0.84	0.95	1.00	0.95	0.35	0.82	1.00	1.06	0.15	0.73	1.00	0.94	0.12	0.61	1.00	1.04	0.33	0.69	1.00	1.11	0.00	0.22	1.00
sphingomyelin (d18:1/22:1, d18:2/22:0, d16:1	Lipid	Sphingomyelins	0.98	0.30	0.73	1.00	0.96	0.11	0.65	1.00	1.01	0.69	0.94	1.00	0.98	0.53	0.84	1.00	1.03	0.27	0.64	1.00	1.05	0.01	0.50	1.00
sphingomyelin (d18:1/22:2, d18:2/22:1, d16:1	Lipid	Sphingomyelins	0.96	0.08	0.50	1.00	0.95	0.15	0.70	1.00	0.97	0.43	0.87	1.00	1.00	0.98	0.99	1.00	1.02	0.66	0.88	1.00	1.02	0.60	0.89	1.00
sphingomyelin (d18:1/24:1, d18:2/24:0)*	Lipid	Sphingomyelins	0.98	0.26	0.71	1.00	0.93	0.01	0.31	1.00	0.99	0.87	0.97	1.00	0.95	0.09	0.60	1.00	1.01	0.56	0.83	1.00	1.07	0.01	0.45	1.00
sphingomyelin (d18:2/14:0, d18:1/14:1)*	Lipid	Sphingomyelins	0.98	0.53	0.86	1.00	0.95	0.24	0.77	1.00	1.00	0.86	0.96	1.00	0.96	0.67	0.91	1.00	1.02	0.66	0.88	1.00	1.06	0.11	0.70	1.00
sphingomyelin (d18:2/16:0, d18:1/16:1)*	Lipid	Sphingomyelins	0.99	0.97	0.99	1.00	0.97	0.56	0.89	1.00	0.99	0.78	0.94	1.00	0.98	0.43	0.79	1.00	1.00	0.64	0.87	1.00	1.02	0.24	0.75	1.00
sphingomyelin (d18:2/18:1)*	Lipid	Sphingomyelins	1.02	0.95	0.99	1.00	1.00	0.87	0.96	1.00	1.00	0.73	0.94	1.00	0.97	0.50	0.84	1.00	0.97	0.48	0.78	1.00	1.00	1.00	1.00	1.00
sphingomyelin (d18:2/21:0, d16:2/23:0)*	Lipid	Sphingomyelins	0.99	0.64	0.93	1.00	0.94	0.07	0.60	1.00	1.00	0.66	0.93	1.00	0.94	0.16	0.64	1.00	1.01	0.81	0.93	1.00	1.07	0.03	0.57	1.00
sphingomyelin (d18:2/23:0, d18:1/23:1, d17:1	Lipid	Sphingomyelins	1.02	0.94	0.99	1.00	0.96	0.28	0.77	1.00	1.06	0.17	0.75	1.00	0.94	0.12	0.61	1.00	1.04	0.34	0.70	1.00	1.11	0.00	0.15	0.66
sphingomyelin (d18:2/23:1)*	Lipid	Sphingomyelins	0.99	0.50	0.84	1.00	0.96	0.24	0.77	1.00	1.00	0.75	0.94	1.00	0.97	0.38	0.79	1.00	1.02	0.67	0.88	1.00	1.05	0.08	0.65	1.00
sphingomyelin (d18:2/24:1, d18:1/24:2)*	Lipid	Sphingomyelins	0.98	0.56	0.88	1.00	0.96	0.14	0.70	1.00	1.01	0.79	0.95	1.00	0.98	0.46	0.82	1.00	1.03	0.18	0.57	1.00	1.05	0.02	0.54	1.00
sphingomyelin (d18:2/24:2)*	Lipid	Sphingomyelins	0.97	0.44	0.80	1.00	0.97	0.26	0.77	1.00	0.98	0.89	0.98	1.00	1.00	0.75	0.93	1.00	1.02	0.66	0.88	1.00	1.02	0.52	0.86	1.00
sphingosine 1-phosphate	Lipid	Sphingosines	0.92	0.07	0.49	1.00	0.96	0.34	0.80	1.00	1.00	1.00	1.00	1.00	1.04	0.32	0.73	1.00	1.09	0.02	0.31	1.00	1.05	0.15	0.73	1.00
stearate (18:0)	Lipid	Long Chain Fatty Acid	0.96	0.64	0.93	1.00	1.00	0.73	0.94	1.00	0.96	0.32	0.83	1.00	1.04	0.32	0.73	1.00	1.00	0.81	0.93	1.00	0.96	0.25	0.75	1.00
stearidonate (18:4n3)	Lipid	Polyunsaturated Fatty Acid	1.12	0.30	0.73	1.00	1.02	0.69	0.92	1.00	0.95	0.79	0.95	1.00	0.91	0.19	0.67	1.00	0.85	0.05	0.39	1.00	0.93	0.17	0.73	1.00
stearoyl sphingomyelin (d18:1/18:0)	Lipid	Sphingomyelins	0.97	0.38	0.77	1.00	0.96	0.24	0.77	1.00	0.97	0.44	0.87	1.00	0.99	0.78	0.94	1.00	1.00	0.70	0.89	1.00	1.01	0.57	0.88	1.00
stearoyl-arachidonoyl-glycerol (18:0/20:4) [1]*	Lipid	Diacylglycerol	1.02	0.73	0.95	1.00	0.97	0.81	0.95	1.00	1.03	1.00	1.00	1.00	0.96	0.49	0.83	1.00	1.01	0.92	0.98	1.00	1.06	0.63	0.90	1.00
stearoylcarnitine (C18)	Lipid	Fatty Acid Metabolism(Acyl	1.02	0.36	0.76	1.00	0.99	0.86	0.95	1.00	1.03	0.53	0.89	1.00	0.97	0.24	0.69	1.00	1.01	0.78	0.93	1.00	1.04	0.08	0.65	1.00
succinate	Energy	TCA Cycle	1.03	0.13	0.56	1.00	1.01	0.87	0.96	1.00	1.06	0.06	0.57	1.00	0.98	0.53	0.84	1.00	1.02	0.58	0.85	1.00	1.05	0.15	0.73	1.00
succinimide	Xenobiotics	Chemical	1.12	0.07	0.48	1.00	1.10	0.02	0.49	1.00	1.04	0.22	0.76	1.00	0.98	0.97	0.99	1.00	0.93	0.17	0.57	1.00	0.95	0.11	0.70	1.00
sulfate*	Xenobiotics	Chemical	0.98	0.30	0.73	1.00	0.99	0.84	0.95	1.00	1.01	0.94	0.99	1.00	1.02	0.36	0.77	1.00	1.03	0.28	0.65	1.00	1.01	0.46	0.84	1.00
tartronate (hydroxymalonate)	Xenobiotics	Food Component/Plant	1.02	0.82	0.95	1.00	0.98	0.60	0.91	1.00	1.03	0.75	0.94	1.00	0.96	0.21	0.68	1.00	1.00	0.90	0.97	1.00	1.05	0.13	0.73	1.00
taurine	Amino Acid	Methionine, Cysteine, SAM	0.92	0.08	0.50	1.00	1.02	0.63	0.91	1.00	1.01	0.73	0.94	1.00	1.10	0.02	0.44	1.00	1.10	0.04	0.36	1.00	1.00	0.98	1.00	1.00
taurochenodeoxycholate	Lipid	Primary Bile Acid Metabolis	0.90	0.58	0.90	1.00	0.90	0.27	0.77	1.00	0.80	0.73	0.94	1.00	1.00	0.15	0.63	1.00	0.89	0.79	0.93	1.00	0.89	0.16	0.73	1.00
taurocholate	Lipid	Primary Bile Acid Metabolis	0.67	0.22	0.66	1.00	0.64	0.17	0.72	1.00	0.66	0.19	0.76	1.00	0.97	0.63	0.88	1.00	1.00	0.95	0.99	1.00	1.03	0.60	0.89	1.00
taurocholenate sulfate*	Lipid	Secondary Bile Acid Metabo	0.85	0.07	0.49	1.00	0.79	0.08	0.62	1.00	0.87	0.21	0.76	1.00	0.94	0.79	0.94	1.00	1.03	0.70	0.89	1.00	1.10	0.73	0.93	1.00
tetradecadienoate (14:2)*	Lipid	Polyunsaturated Fatty Acid	1.17	0.05	0.45	1.00	1.11	0.33	0.79	1.00	1.16	0.18	0.76	1.00	0.95	0.27	0.70	1.00	0.99	0.38	0.73	1.00	1.04	0.82	0.96	1.00
tetradecanedioate (C14)	Lipid	Fatty Acid, Dicarboxylate	0.97	0.90	0.98	1.00	0.83	0.06	0.60	1.00	0.90	0.52	0.89	1.00	0.85	0.02	0.44	1.00	0.93	0.60	0.86	1.00	1.09	0.28	0.75	1.00
theobromine	Xenobiotics	Xanthine Metabolism	0.95	0.29	0.73	1.00	0.96	0.94	0.98	1.00	0.95	0.63	0.92	1.00	1.00	0.56	0.86	1.00	1.00	0.44	0.76	1.00	1.00	0.89	0.98	1.00
theophylline	Xenobiotics	Xanthine Metabolism	0.86	0.13	0.56	1.00	0.96	0.79	0.94	1.00	0.98	0.94	0.99	1.00	1.11	0.21	0.68	1.00	1.14	0.27	0.64	1.00	1.02	0.86	0.97	1.00
thioproline	Xenobiotics	Chemical	1.16	0.03	0.36	1.00	1.12	0.10	0.65	1.00	1.17	0.01	0.36	1.00	0.97	0.82	0.95	1.00	1.01	0.53	0.82	1.00	1.04	0.34	0.80	1.00
threonate	Cofactors and	Ascorbate and Aldarate Met	1.01	0.84	0.95	1.00	0.95	0.20	0.75	1.00	0.95	0.29	0.82	1.00	0.94	0.07	0.53	1.00	0.94	0.11	0.48	1.00	1.01	0.86	0.97	1.00
threonine	Amino Acid	Glycine, Serine and Threoni	1.03	0.70	0.94	1.00	1.08	0.08	0.62	1.00	1.14	0.00	0.29	1.00	1.05	0.24	0.68	1.00	1.10	0.01	0.31	1.00	1.05	0.13	0.73	1.00
thyroxine	Amino Acid	Tyrosine Metabolism	0.98	0.78	0.95	1.00	0.98	0.38	0.82	1.00	1.00	0.92	0.98	1.00	1.00	0.89	0.98	1.00	1.03	0.44	0.76	1.00	1.02	0.48	0.85	1.00
hydroxyproline	Amino Acid	Urea cycle; Arginine and Pro	0.97	1.00	1.00	1.00	1.03	0.34	0.80	1.00	1.13	0.21	0.76	1.00	1.05	0.26	0.70	1.00	1.16	0.14	0.52	1.00	1.10	0.32	0.78	1.00
tricosanoyl sphingomyelin (d18:1/23:0)*	Lipid	Sphingomyelins	1.02	0.66	0.93	1.00	0.93	0.21	0.76	1.00	1.04	0.36	0.85	1.00	0.91	0.18	0.66	1.00	1.03	0.52	0.82	1.00	1.12	0.02	0.54	1.00
tridecenedioate (C13:1-DC)*	Lipid	Fatty Acid, Dicarboxylate	1.08	0.11	0.53	1.00	0.98	0.90	0.98	1.00	0.96	0.73	0.94	1.00	0.91	0.13	0.62	1.00	0.88	0.13	0.50	1.00	0.98	0.84	0.97	1.00
triethanolamine	Xenobiotics	Chemical	0.93	0.13	0.55	1.00	0.92	0.03	0.51	1.00	0.99	0.45	0.88	1.00	0.99	0.97	0.99	1.00	1.07	0.21	0.60	1.00	1.07	0.16	0.73	1.00
trigonelline (N'-methylnicotinate)	Cofactors and	Nicotinate and Nicotinamic	0.79	0.10	0.51	1.00	0.90	0.36	0.82	1.00	0.93	0.84	0.96	1.00	1.14	0.06	0.51	1.00	1.17	0.08	0.43	1.00	1.03	0.52	0.86	1.00
trimethylamine N-oxide	Lipid	Phospholipid Metabolism	0.99	0.42	0.79	1.00	1.11	0.94	0.98	1.00	1.44	0.56	0.89	1.00	1.12	0.75	0.93	1.00	1.45	0.98	1.00	1.00	1.29	0.50	0.86	1.00
tryptophan	Amino Acid	Tryptophan Metabolism	0.99	0.73	0.95	1.00	1.00	0.87	0.96	1.00	1.03	0.29	0.82	1.00	1.01	0.56	0.86	1.00	1.05	0.15	0.52	1.00	1.03	0.12	0.72	1.00
tyrosine	Amino Acid	Tyrosine Metabolism	1.01	0.94	0.99	1.00	1.04	0.38	0.82	1.00	1.05	0.11	0.65	1.00	1.03	0.28	0.72	1.00	1.04	0.24	0.63	1.00	1.00	0.67	0.92	1.00
urate	Nucleotide	Purine Metabolism, (Hypo)	1.00	0.60	0.91	1.00	1.01	0.48	0.86	1.00	1.03	0.43	0.87	1.00	1.01	0.42	0.79	1.00	1.03	0.15	0.52	1.00	1.01	0.73	0.93	1.00
urea	Amino Acid	Urea cycle; Arginine and Pro	1.00	0.98	1.00	1.00	1.05	0.23	0.77	1.00	1.03	0.42	0.87	1.00	1.05	0.15	0.63	1.00	1.03	0.25	0.64	1.00	0.98	0.72	0.93	1.00
uridine	Nucleotide	Pyrimidine Metabolism, Ur	1.03	0.24	0.68	1.00	1.02	0.56	0.89	1.00	1.03	0.56	0.89	1.00	0.99	0.61	0.88	1.00	1.00	0.50	0.80	1.00	1.01	0.63	0.90	1.00
valine	Amino Acid	Leucine, Isoleucine and Val	1.01	0.61	0.92	1.00	1.05	0.15	0.70	1.00	1.01	0.49	0.89	1.00	1.04	0.19	0.67	1.00	1.00	0.61	0.87	1.00	0.96	0.38	0.82	1.00
vanillylmandelate (VMA)	Amino Acid	Tyrosine Metabolism	1.02	0.42	0.79	1.00	1.01	0.56	0.89	1.00	0.98	0.98	1.00	1.00	0.99	0.90	0.98	1.00	0.96	0.34	0.70	1.00	0.97	0.57	0.88	1.00
xanthine	Nucleotide	Purine Metabolism, (Hypo)	0.97	0.43	0.80	1.00	1.00	0.63	0.91	1.00	1.00	0.95	0.99	1.00	1.02	0.86	0.97	1.00	1.03	0.49	0.79	1.00	1.00	0.89	0.98	1.00
ximenoylcarnitine (C26:1)*	Lipid	Fatty Acid Metabolism(Acyl	0.96	0.15	0.58	1.00	0.91	0.04	0.51	1.00	0.98	0.54	0.89	1.00	0.95	0.43	0.79	1.00	1.03	0.26	0.64	1.00	1.08	0.16	0.73	1.00
X - 09789	Unknown	Unknown	1.04	0.95	0.99	1.00	0.81	0.08	0.62	1.00	0.80	0.01	0.41	1.00	0.77	0.02	0.44	1.00	0.77	0.15	0.52	1.00	1.00	0.90	0.98	1.00
X - 11261	Unknown	Unknown	0.91	0.23	0.66	1.00	0.89	0.18	0.73	1.00	0.96	0.82	0.96	1.00	0.98	0.60	0.87	1.00	1.06	0.26	0.64	1.00	1.08	0.13	0.73	1.00
X - 11308	Unknown	Unknown	0.99	1.00	1.00	1.00	1.01	0.56	0.89	1.00	1.00	0.63	0.92	1.00	1.01	0.26	0.70	1.00	1.00	0.79	0.93	1.00	0.99	0.66	0.92	1.00
X - 11315	Unknown	Unknown	1.01	0.70	0.94	1.00	1.09	0.31	0.79	1.00	1.03	0.66	0.93	1.00	1.08	0.52	0.84	1.00	1.02	0.69	0.88	1.00	0.94	0.29	0.75	1.00
X - 11372	Unknown	Unknown	1.05	0.34	0.74	1.00	1.10	0.10	0.65	1.00	1.06	0.31	0.82	1.00	1.04	0.12	0.61	1.00	1.01	0.97	0.99	1.00	0.97	0.13	0.73	1.00

X - 11444	Unknown	Unknown	1.08	0.13	0.56	1.00	1.07	0.06	0.56	1.00	1.00	0.48	0.89	1.00	0.99	0.42	0.79	1.00	0.93	0.24	0.63	1.00	0.94	0.12	0.72	1.00
X - 11470	Unknown	Unknown	1.07	0.48	0.82	1.00	1.04	0.61	0.91	1.00	1.13	0.15	0.74	1.00	0.97	0.92	0.99	1.00	1.05	0.27	0.64	1.00	1.09	0.26	0.75	1.00
X - 11530	Unknown	Unknown	1.07	0.86	0.96	1.00	0.91	0.67	0.92	1.00	1.10	0.54	0.89	1.00	0.85	0.15	0.63	1.00	1.02	0.84	0.94	1.00	1.21	0.12	0.73	1.00
X - 11787	Unknown	Unknown	1.01	0.84	0.95	1.00	1.04	0.24	0.77	1.00	1.06	0.07	0.58	1.00	1.03	0.24	0.68	1.00	1.05	0.06	0.39	1.00	1.02	0.45	0.83	1.00
X - 11795	Unknown	Unknown	0.99	0.76	0.95	1.00	1.08	0.58	0.90	1.00	1.04	0.38	0.86	1.00	1.09	0.44	0.80	1.00	1.05	0.23	0.62	1.00	0.97	0.90	0.98	1.00
X - 12026	Unknown	Unknown	0.99	0.81	0.95	1.00	1.02	0.52	0.88	1.00	1.08	0.02	0.46	1.00	1.03	0.35	0.76	1.00	1.09	0.01	0.31	1.00	1.06	0.02	0.54	1.00
X - 12063	Unknown	Unknown	0.99	0.69	0.94	1.00	1.02	0.49	0.87	1.00	1.04	0.98	1.00	1.00	1.03	0.16	0.64	1.00	1.05	0.18	0.57	1.00	1.02	0.42	0.82	1.00
X - 12100	Unknown	Unknown	1.04	0.32	0.73	1.00	1.08	0.07	0.60	1.00	1.06	0.30	0.82	1.00	1.04	0.08	0.58	1.00	1.02	0.18	0.57	1.00	0.98	0.54	0.87	1.00
X - 12104	Unknown	Unknown	1.06	0.04	0.42	1.00	1.07	0.10	0.65	1.00	1.06	0.08	0.59	1.00	1.00	0.64	0.89	1.00	0.99	0.70	0.89	1.00	0.99	0.81	0.96	1.00
X - 12206	Unknown	Unknown	1.01	0.94	0.99	1.00	0.99	0.84	0.95	1.00	1.03	0.46	0.89	1.00	0.99	0.94	0.99	1.00	1.03	0.35	0.71	1.00	1.04	0.05	0.62	1.00
X - 12216	Unknown	Unknown	1.06	0.58	0.90	1.00	1.16	0.54	0.89	1.00	1.21	0.02	0.44	1.00	1.09	0.66	0.90	1.00	1.15	0.02	0.31	1.00	1.05	0.25	0.75	1.00
X - 12230	Unknown	Unknown	0.52	0.01	0.22	1.00	0.88	0.31	0.79	1.00	0.70	0.09	0.60	1.00	1.68	0.11	0.61	1.00	1.33	0.01	0.31	1.00	0.79	0.45	0.83	1.00
X - 12411	Unknown	Unknown	0.86	0.57	0.89	1.00	0.95	0.94	0.98	1.00	1.09	0.84	0.96	1.00	1.10	0.27	0.70	1.00	1.28	0.63	0.87	1.00	1.16	0.63	0.90	1.00
X - 12462	Unknown	Unknown	0.95	0.56	0.88	1.00	1.01	0.86	0.95	1.00	1.05	0.43	0.87	1.00	1.07	0.21	0.67	1.00	1.11	0.01	0.31	1.00	1.04	0.64	0.91	1.00
X - 12472	Unknown	Unknown	1.14	0.18	0.62	1.00	0.82	0.63	0.91	1.00	0.93	0.98	1.00	1.00	0.72	0.05	0.51	1.00	0.82	0.43	0.75	1.00	1.13	0.69	0.93	1.00
X - 12524	Unknown	Unknown	0.85	0.04	0.40	1.00	0.94	0.61	0.91	1.00	0.92	0.16	0.75	1.00	1.10	0.05	0.51	1.00	1.08	0.31	0.68	1.00	0.98	0.63	0.90	1.00
X - 12544	Unknown	Unknown	2.12	0.60	0.91	1.00	2.68	0.52	0.88	1.00	2.17	0.90	0.98	1.00	1.26	0.81	0.95	1.00	1.02	0.76	0.92	1.00	0.81	0.79	0.96	1.00
X - 12844	Unknown	Unknown	1.08	0.13	0.55	1.00	1.07	0.07	0.61	1.00	1.02	0.49	0.89	1.00	0.99	0.81	0.95	1.00	0.95	0.10	0.45	1.00	0.95	0.43	0.83	1.00
X - 12846	Unknown	Unknown	1.03	0.69	0.94	1.00	1.04	0.61	0.91	1.00	1.12	0.01	0.39	1.00	1.01	0.97	0.99	1.00	1.09	0.12	0.48	1.00	1.08	0.06	0.64	1.00
X - 13431	Unknown	Unknown	1.12	0.20	0.64	1.00	1.23	0.07	0.60	1.00	1.15	0.04	0.54	1.00	1.10	0.11	0.61	1.00	1.03	0.29	0.67	1.00	0.94	0.73	0.93	1.00
X - 13729	Unknown	Unknown	0.98	0.72	0.95	1.00	1.05	0.73	0.94	1.00	1.14	0.07	0.58	1.00	1.07	0.82	0.95	1.00	1.16	0.05	0.38	1.00	1.09	0.05	0.60	1.00
X - 13866	Unknown	Unknown	1.01	0.87	0.97	1.00	0.98	0.76	0.94	1.00	0.95	0.69	0.94	1.00	0.97	0.90	0.98	1.00	0.95	0.60	0.86	1.00	0.97	0.76	0.95	1.00
X - 14056	Unknown	Unknown	1.07	0.92	0.99	1.00	1.10	0.45	0.85	1.00	1.19	0.10	0.64	1.00	1.03	0.42	0.79	1.00	1.11	0.12	0.48	1.00	1.08	0.33	0.79	1.00
X - 14939	Unknown	Unknown	0.99	0.50	0.84	1.00	0.96	0.31	0.79	1.00	0.99	0.79	0.95	1.00	0.97	0.90	0.98	1.00	1.00	0.86	0.95	1.00	1.03	0.49	0.85	1.00
X - 15245	Unknown	Unknown	1.07	0.46	0.82	1.00	0.97	0.92	0.98	1.00	1.06	0.76	0.94	1.00	0.91	0.21	0.67	1.00	0.99	0.92	0.98	1.00	1.09	0.73	0.93	1.00
X - 15469	Unknown	Unknown	1.07	0.40	0.79	1.00	0.99	0.82	0.95	1.00	0.99	0.67	0.93	1.00	0.92	0.66	0.90	1.00	0.93	0.12	0.49	1.00	1.00	0.92	0.98	1.00
X - 15486	Unknown	Unknown	0.99	0.82	0.95	1.00	1.02	0.95	0.99	1.00	1.10	0.29	0.82	1.00	1.02	0.73	0.93	1.00	1.10	0.10	0.46	1.00	1.08	0.04	0.57	1.00
X - 15492	Unknown	Unknown	1.20	0.01	0.26	1.00	1.11	0.05	0.54	1.00	1.20	0.01	0.36	1.00	0.93	0.32	0.73	1.00	1.00	0.87	0.95	1.00	1.08	0.29	0.75	1.00
X - 15503	Unknown	Unknown	1.10	0.04	0.40	1.00	1.11	0.11	0.65	1.00	1.15	0.00	0.24	1.00	1.01	0.98	0.99	1.00	1.04	0.50	0.80	1.00	1.03	0.26	0.75	1.00
X - 16087	Unknown	Unknown	1.01	0.79	0.95	1.00	1.05	0.63	0.91	1.00	1.00	0.53	0.89	1.00	1.03	0.98	0.99	1.00	0.99	0.89	0.96	1.00	0.96	0.90	0.98	1.00
X - 16580	Unknown	Unknown	1.02	0.57	0.89	1.00	1.00	0.73	0.94	1.00	1.10	0.49	0.89	1.00	0.98	0.44	0.80	1.00	1.08	0.28	0.65	1.00	1.10	0.18	0.73	1.00
X - 16938	Unknown	Unknown	1.12	0.21	0.65	1.00	1.07	0.40	0.82	1.00	1.16	0.23	0.77	1.00	0.95	0.10	0.61	1.00	1.04	0.52	0.82	1.00	1.09	0.09	0.67	1.00
X - 16944	Unknown	Unknown	0.92	0.40	0.79	1.00	0.89	0.40	0.82	1.00	0.97	0.97	1.00	1.00	0.97	0.50	0.84	1.00	1.05	0.20	0.60	1.00	1.08	0.08	0.65	1.00
X - 17335	Unknown	Unknown	1.12	0.11	0.53	1.00	1.01	0.98	0.99	1.00	0.98	0.84	0.96	1.00	0.90	0.02	0.44	1.00	0.88	0.00	0.24	1.00	0.98	0.38	0.82	1.00
X - 17337	Unknown	Unknown	1.09	0.12	0.55	1.00	1.09	0.26	0.77	1.00	1.03	0.60	0.92	1.00	0.99	0.73	0.93	1.00	0.94	0.24	0.63	1.00	0.94	0.98	1.00	1.00
X - 17340	Unknown	Unknown	1.10	0.22	0.66	1.00	1.07	0.19	0.75	1.00	1.15	0.02	0.44	1.00	0.97	0.48	0.82	1.00	1.05	0.45	0.77	1.00	1.07	0.04	0.57	1.00
X - 17357	Unknown	Unknown	1.07	0.45	0.81	1.00	1.05	0.29	0.78	1.00	0.94	0.31	0.82	1.00	0.98	0.92	0.99	1.00	0.87	0.01	0.31	1.00	0.89	0.04	0.57	1.00
X - 17653	Unknown	Unknown	1.04	0.48	0.82	1.00	1.09	0.27	0.77	1.00	1.11	0.16	0.75	1.00	1.05	0.23	0.68	1.00	1.07	0.38	0.73	1.00	1.02	0.95	0.99	1.00
X - 17654	Unknown	Unknown	1.03	0.72	0.95	1.00	1.04	0.40	0.82	1.00	1.05	0.38	0.86	1.00	1.02	0.36	0.77	1.00	1.02	0.70	0.89	1.00	1.01	0.98	1.00	1.00
X - 17676	Unknown	Unknown	0.92	0.01	0.26	1.00	1.00	0.72	0.94	1.00	0.92	0.48	0.89	1.00	1.10	0.39	0.79	1.00	1.01	0.34	0.70	1.00	0.92	0.95	0.99	1.00
X - 18249	Unknown	Unknown	0.95	0.04	0.40	1.00	0.92	0.01	0.30	1.00	0.85	0.00	0.06	0.17	0.96	0.19	0.67	1.00	0.89	0.00	0.21	0.42	0.92	0.00	0.15	0.95
X - 18779	Unknown	Unknown	0.98	0.73	0.95	1.00	1.10	0.11	0.65	1.00	1.02	0.73	0.94	1.00	1.13	0.02	0.44	1.00	1.04	0.82	0.94	1.00	0.93	0.14	0.73	1.00
X - 18913	Unknown	Unknown	0.73	0.07	0.49	1.00	0.67	0.01	0.39	1.00	0.66	0.02	0.44	1.00	0.91	0.81	0.95	1.00	0.90	0.69	0.88	1.00	0.99	0.87	0.97	1.00
X - 18914	Unknown	Unknown	0.94	0.05	0.45	1.00	0.99	0.44	0.84	1.00	0.95	0.03	0.53	1.00	1.05	0.38	0.79	1.00	1.01	0.54	0.83	1.00	0.96	0.01	0.50	1.00
X - 18921	Unknown	Unknown	0.95	0.79	0.95	1.00	0.90	0.18	0.73	1.00	0.91	0.63	0.92	1.00	0.95	0.37	0.78	1.00	0.96	0.54	0.83	1.00	1.02	0.39	0.82	1.00
X - 18922	Unknown	Unknown	1.05	0.90	0.98	1.00	1.04	0.86	0.95	1.00	1.04	0.56	0.89	1.00	1.00	0.86	0.97	1.00	1.00	0.36	0.71	1.00	1.00	0.70	0.93	1.00
X - 19141	Unknown	Unknown	0.89	0.13	0.55	1.00	0.99	0.82	0.95	1.00	1.21	0.20	0.76	1.00	1.11	0.07	0.55	1.00	1.35	0.00	0.31	1.00	1.23	0.16	0.73	1.00
X - 21258	Unknown	Unknown	0.73	0.84	0.95	1.00	1.09	0.26	0.77	1.00	0.67	0.92	0.98	1.00	1.49	0.07	0.55	1.00	0.92	0.97	0.99	1.00	0.61	0.08	0.65	1.00
X - 21286	Unknown	Unknown	1.04	0.79	0.95	1.00	1.02	0.67	0.92	1.00	1.12	0.18	0.76	1.00	0.99	0.70	0.92	1.00	1.08	0.18	0.57	1.00	1.09	0.16	0.73	1.00
X - 21310	Unknown	Unknown	1.05	0.66	0.93	1.00	1.07	0.49	0.87	1.00	1.13	0.06	0.57	1.00	1.02	0.56	0.86	1.00	1.07	0.23	0.62	1.00	1.06	0.26	0.75	1.00
X - 21319	Unknown	Unknown	1.02	0.87	0.97	1.00	0.95	0.28	0.77	1.00	1.02	0.87	0.97	1.00	0.93	0.31	0.73	1.00	1.00	0.76	0.92	1.00	1.07	0.21	0.73	1.00
X - 21339	Unknown	Unknown	1.05	0.44	0.80	1.00	1.09	0.44	0.84	1.00	1.04	0.58	0.91	1.00	1.03	0.44	0.80	1.00	0.99	0.37	0.72	1.00	0.95	0.19	0.73	1.00
X - 21353	Unknown	Unknown	1.10	0.17	0.60	1.00	1.05	0.39	0.82	1.00	1.02	0.39	0.86	1.00	0.96	0.97	0.99	1.00	0.92	0.30	0.67	1.00	0.96	0.22	0.74	1.00
X - 21383	Unknown	Unknown	1.04	0.84	0.95	1.00	1.02	0.94	0.98	1.00	1.09	0.44	0.87	1.00	0.98	0.98	0.99	1.00	1.05	0.31	0.68	1.00	1.07	0.60	0.89	1.00

X - 21411	Unknown	Unknown	0.72	0.15	0.58	1.00	0.65	0.00	0.29	1.00	0.66	0.07	0.58	1.00	0.91	0.36	0.77	1.00	0.92	0.61	0.87	1.00	1.01	0.69	0.93	1.00
X - 21628	Unknown	Unknown	0.98	0.60	0.91	1.00	0.96	0.28	0.77	1.00	0.99	0.95	0.99	1.00	0.98	0.43	0.79	1.00	1.01	0.94	0.98	1.00	1.03	0.39	0.82	1.00
X - 21736	Unknown	Unknown	1.01	1.00	1.00	1.00	1.04	0.39	0.82	1.00	1.08	0.69	0.94	1.00	1.03	0.98	0.99	1.00	1.07	0.63	0.87	1.00	1.04	0.70	0.93	1.00
X - 21785	Unknown	Unknown	1.03	0.35	0.74	1.00	0.99	0.84	0.95	1.00	1.01	0.87	0.97	1.00	0.97	0.50	0.84	1.00	0.99	0.95	0.99	1.00	1.02	0.28	0.75	1.00
X - 21796	Unknown	Unknown	1.02	0.28	0.72	1.00	0.99	0.57	0.89	1.00	1.02	0.92	0.98	1.00	0.97	0.72	0.92	1.00	1.00	0.18	0.57	1.00	1.03	0.18	0.73	1.00
X - 21829	Unknown	Unknown	1.04	0.79	0.95	1.00	0.92	0.26	0.77	1.00	0.97	0.76	0.94	1.00	0.89	0.05	0.51	1.00	0.94	0.35	0.71	1.00	1.05	0.21	0.73	1.00
X - 22162	Unknown	Unknown	1.07	0.20	0.64	1.00	1.08	0.12	0.67	1.00	1.05	0.34	0.84	1.00	1.00	0.75	0.93	1.00	0.98	0.69	0.88	1.00	0.98	0.54	0.87	1.00
X - 22519	Unknown	Unknown	1.09	0.54	0.87	1.00	1.04	0.60	0.91	1.00	0.98	0.56	0.89	1.00	0.95	0.52	0.84	1.00	0.90	0.01	0.31	1.00	0.94	0.18	0.73	1.00
X - 22771	Unknown	Unknown	1.08	0.29	0.73	1.00	1.06	0.36	0.82	1.00	1.13	0.16	0.75	1.00	0.98	0.94	0.99	1.00	1.04	0.31	0.68	1.00	1.07	0.24	0.75	1.00
X - 22775	Unknown	Unknown	0.98	0.30	0.73	1.00	0.98	0.32	0.79	1.00	1.00	0.76	0.94	1.00	1.00	0.73	0.93	1.00	1.02	0.18	0.57	1.00	1.02	0.31	0.78	1.00
X - 23314	Unknown	Unknown	1.10	0.98	1.00	1.00	0.88	0.37	0.82	1.00	0.65	0.13	0.69	1.00	0.80	0.14	0.62	1.00	0.59	0.15	0.52	1.00	0.73	0.44	0.83	1.00
X - 23369	Unknown	Unknown	0.97	0.38	0.77	1.00	0.96	0.46	0.85	1.00	1.02	0.63	0.92	1.00	0.99	0.90	0.98	1.00	1.05	0.21	0.60	1.00	1.06	0.20	0.73	1.00
X - 23585	Unknown	Unknown	0.82	0.50	0.84	1.00	0.84	0.67	0.92	1.00	0.80	0.56	0.89	1.00	1.03	0.45	0.81	1.00	0.97	0.89	0.96	1.00	0.94	0.44	0.83	1.00
X - 23593	Unknown	Unknown	1.01	0.61	0.92	1.00	1.05	0.49	0.87	1.00	1.04	0.31	0.82	1.00	1.03	0.28	0.72	1.00	1.03	0.21	0.60	1.00	0.99	0.76	0.95	1.00
X - 23639	Unknown	Unknown	1.04	0.44	0.80	1.00	0.99	0.86	0.95	1.00	1.03	0.60	0.92	1.00	0.96	0.89	0.98	1.00	0.98	0.72	0.89	1.00	1.03	0.61	0.90	1.00
X - 23680	Unknown	Unknown	0.93	0.28	0.72	1.00	0.98	0.67	0.92	1.00	0.97	0.90	0.98	1.00	1.05	0.79	0.94	1.00	1.05	0.48	0.78	1.00	1.00	0.64	0.91	1.00
X - 23974	Unknown	Unknown	1.03	0.42	0.79	1.00	0.94	0.50	0.88	1.00	0.99	0.87	0.97	1.00	0.91	0.19	0.67	1.00	0.96	0.63	0.87	1.00	1.05	0.32	0.78	1.00
X - 23997	Unknown	Unknown	0.85	0.31	0.73	1.00	0.95	0.86	0.95	1.00	1.01	0.40	0.86	1.00	1.12	0.24	0.68	1.00	1.19	0.04	0.37	1.00	1.07	0.29	0.75	1.00
X - 24106 - retired for palmitoyl-sphingosine-ph	Unknown	Unknown	0.98	0.31	0.73	1.00	0.97	0.36	0.82	1.00	1.00	1.00	1.00	1.00	0.99	0.98	0.99	1.00	1.02	0.84	0.94	1.00	1.03	0.33	0.79	1.00
X - 24435	Unknown	Unknown	0.81	0.01	0.24	1.00	0.83	0.05	0.54	1.00	0.80	0.04	0.54	1.00	1.02	0.97	0.99	1.00	1.00	0.82	0.94	1.00	0.97	0.54	0.87	1.00
X - 24549	Unknown	Unknown	0.93	0.52	0.85	1.00	0.99	0.67	0.92	1.00	0.98	0.75	0.94	1.00	1.06	0.07	0.53	1.00	1.06	0.32	0.69	1.00	0.99	0.73	0.93	1.00
X - 24588	Unknown	Unknown	0.93	0.11	0.52	1.00	0.92	0.08	0.62	1.00	0.93	0.06	0.57	1.00	0.99	0.87	0.97	1.00	1.00	0.90	0.97	1.00	1.01	0.81	0.96	1.00
X - 24699	Unknown	Unknown	1.07	0.06	0.47	1.00	1.03	0.40	0.82	1.00	1.03	0.38	0.86	1.00	0.96	0.63	0.88	1.00	0.96	0.28	0.65	1.00	1.00	0.95	0.99	1.00
X - 24765	Unknown	Unknown	0.98	0.81	0.95	1.00	1.05	0.09	0.65	1.00	1.04	0.24	0.79	1.00	1.07	0.02	0.44	1.00	1.06	0.06	0.39	1.00	0.99	0.97	1.00	1.00
X - 24813	Unknown	Unknown	1.02	0.52	0.85	1.00	1.01	0.63	0.91	1.00	1.03	0.36	0.85	1.00	0.99	0.76	0.93	1.00	1.01	0.81	0.93	1.00	1.02	0.50	0.86	1.00
X - 24951	Unknown	Unknown	1.09	0.56	0.88	1.00	1.03	0.64	0.92	1.00	1.04	0.76	0.94	1.00	0.94	0.45	0.81	1.00	0.95	0.73	0.90	1.00	1.01	0.70	0.93	1.00
X - 24952	Unknown	Unknown	0.96	0.73	0.95	1.00	1.11	0.03	0.51	1.00	1.14	0.12	0.65	1.00	1.16	0.05	0.51	1.00	1.19	0.02	0.31	1.00	1.03	0.84	0.97	1.00
X - 25422	Unknown	Unknown	1.10	0.15	0.58	1.00	1.17	0.01	0.30	1.00	1.09	0.05	0.57	1.00	1.07	0.31	0.73	1.00	1.00	0.73	0.90	1.00	0.93	0.19	0.73	1.00
(14 or 15)-methylpalmitate (a17:0 or i17:0)	Lipid	Fatty Acid, Branched	0.99	0.92	0.99	1.00	0.98	0.73	0.94	1.00	0.94	0.45	0.88	1.00	0.99	0.87	0.97	1.00	0.95	0.28	0.65	1.00	0.96	0.27	0.75	1.00
1-(1-enyl-oleoyl)-GPE (P-18:1)*	Lipid	Lysoplasmalogen	1.00	0.94	0.99	1.00	1.02	0.72	0.94	1.00	1.03	0.64	0.92	1.00	1.02	1.00	1.00	1.00	1.03	0.14	0.52	1.00	1.02	0.43	0.83	1.00
1,2,3-benzenetriol sulfate (2)	Xenobiotics	Chemical	0.35	0.01	0.20	1.00	0.68	0.21	0.75	1.00	0.40	0.01	0.38	1.00	1.93	0.09	0.60	1.00	1.14	0.39	0.73	1.00	0.59	0.95	0.99	1.00
1,3,7-trimethylurate	Xenobiotics	Xanthine Metabolism	0.64	0.01	0.20	1.00	0.83	0.40	0.82	1.00	0.81	0.26	0.81	1.00	1.29	0.02	0.44	1.00	1.26	0.05	0.39	1.00	0.97	0.90	0.98	1.00
12,13-DiHOME	Lipid	Fatty Acid, Dihydroxy	0.62	0.22	0.66	1.00	0.60	0.12	0.67	1.00	0.56	0.02	0.46	1.00	0.97	0.82	0.95	1.00	0.89	0.43	0.75	1.00	0.92	0.45	0.83	1.00
12-HETE	Lipid	Eicosanoid	1.25	0.38	0.77	1.00	1.48	0.08	0.62	1.00	1.32	0.08	0.58	1.00	1.19	0.63	0.88	1.00	1.06	0.36	0.71	1.00	0.89	0.61	0.90	1.00
12-HHTrE	Lipid	Eicosanoid	0.98	0.29	0.73	1.00	1.31	0.00	0.29	1.00	1.28	0.10	0.63	1.00	1.34	0.03	0.46	1.00	1.31	0.04	0.36	1.00	0.98	0.89	0.98	1.00
1-arachidonoyl-GPA (20:4)	Lipid	Lysophospholipid	0.70	0.01	0.20	1.00	1.06	0.67	0.92	1.00	0.93	0.37	0.85	1.00	1.51	0.00	0.21	0.42	1.33	0.01	0.31	1.00	0.88	0.39	0.82	1.00
1-carboxyethylisoleucine	Amino Acid	Leucine, Isoleucine and Val	0.61	0.00	0.20	1.00	0.85	0.26	0.77	1.00	0.74	0.12	0.65	1.00	1.40	0.00	0.31	1.00	1.22	0.06	0.40	1.00	0.87	0.35	0.80	1.00
1-carboxyethylleucine	Amino Acid	Leucine, Isoleucine and Val	0.70	0.00	0.20	1.00	0.85	0.12	0.67	1.00	0.87	0.29	0.82	1.00	1.21	0.15	0.63	1.00	1.23	0.02	0.31	1.00	1.02	0.26	0.75	1.00
1-carboxyethylvaline	Amino Acid	Leucine, Isoleucine and Val	0.70	0.00	0.17	1.00	0.80	0.05	0.54	1.00	0.75	0.09	0.60	1.00	1.15	0.18	0.67	1.00	1.07	0.25	0.64	1.00	0.93	0.97	1.00	1.00
1-heptadecenoylglycerol (17:1)*	Lipid	Monoacylglycerol	0.85	0.61	0.92	1.00	0.91	0.73	0.94	1.00	0.97	0.90	0.98	1.00	1.06	0.67	0.91	1.00	1.14	0.21	0.60	1.00	1.07	0.34	0.80	1.00
1-lignoceroyl-GPC (24:0)	Lipid	Lysophospholipid	1.01	0.60	0.91	1.00	0.86	0.03	0.51	1.00	0.98	0.84	0.96	1.00	0.85	0.06	0.51	1.00	0.98	0.86	0.95	1.00	1.15	0.04	0.60	1.00
1-linolenoylglycerol (18:3)	Lipid	Monoacylglycerol	0.86	0.34	0.74	1.00	0.84	0.33	0.79	1.00	0.92	0.75	0.94	1.00	0.98	0.53	0.84	1.00	1.06	0.52	0.82	1.00	1.09	0.14	0.73	1.00
1-linoleoyl-2-arachidonoyl-GPC (18:2/20:4n6)*	Lipid	Phosphatidylcholine (PC)	1.02	0.87	0.97	1.00	1.00	0.66	0.92	1.00	1.05	0.61	0.92	1.00	0.98	0.73	0.93	1.00	1.03	0.48	0.78	1.00	1.06	0.29	0.75	1.00
1-linoleoyl-GPG (18:2)*	Lipid	Lysophospholipid	0.72	0.02	0.30	1.00	0.90	0.33	0.79	1.00	0.67	0.00	0.22	0.87	1.25	0.05	0.51	1.00	0.92	0.63	0.87	1.00	0.74	0.00	0.15	1.00
1-methyl-5-imidazoleacetate	Amino Acid	Histidine Metabolism	0.86	0.69	0.94	1.00	0.85	0.63	0.91	1.00	0.96	0.78	0.94	1.00	0.98	0.78	0.94	1.00	1.12	0.81	0.93	1.00	1.14	0.69	0.93	1.00
1-methylurate	Xenobiotics	Xanthine Metabolism	0.72	0.10	0.51	1.00	0.87	0.48	0.86	1.00	0.98	0.84	0.96	1.00	1.21	0.12	0.61	1.00	1.36	0.06	0.39	1.00	1.12	0.23	0.75	1.00
1-methylxanthine	Xenobiotics	Xanthine Metabolism	0.77	0.13	0.56	1.00	0.89	0.23	0.77	1.00	0.98	0.86	0.96	1.00	1.16	0.08	0.56	1.00	1.26	0.04	0.36	1.00	1.09	0.37	0.81	1.00
1-oleoyl-2-arachidonoyl-GPE (18:1/20:4)*	Lipid	Phosphatidylethanolamine	1.01	0.72	0.95	1.00	0.91	0.37	0.82	1.00	0.91	0.31	0.82	1.00	0.90	0.03	0.46	1.00	0.90	0.22	0.62	1.00	1.00	0.79	0.96	1.00
1-palmitoleoyl-2-linolenoyl-GPC (16:1/18:3)*	Lipid	Phosphatidylcholine (PC)	1.02	0.73	0.95	1.00	0.94	0.56	0.89	1.00	1.04	0.67	0.93	1.00	0.92	0.58	0.87	1.00	1.02	0.94	0.98	1.00	1.11	0.39	0.82	1.00
1-palmitoyl-2-oleoyl-GPE (16:0/18:1)	Lipid	Phosphatidylethanolamine	0.90	0.50	0.84	1.00	0.86	0.32	0.79	1.00	0.93	0.36	0.85	1.00	0.96	0.43	0.79	1.00	1.04	0.58	0.85	1.00	1.08	0.29	0.75	1.00
1-palmitoyl-GPG (16:0)*	Lipid	Lysophospholipid	0.71	0.01	0.20	1.00	0.90	0.24	0.77	1.00	0.77	0.01	0.39	1.00	1.27	0.04	0.50	1.00	1.08	0.73	0.90	1.00	0.85	0.04	0.57	1.00
1-stearoyl-2-oleoyl-GPE (18:0/18:1)	Lipid	Phosphatidylethanolamine	0.71	0.04	0.40	1.00	0.71	0.04	0.52	1.00	0.81	0.17	0.75	1.00	1.01	0.92	0.99	1.00	1.15	0.06	0.40	1.00	1.14	0.06	0.64	1.00
1-stearoyl-2-oleoyl-GPS (18:0/18:1)	Lipid	Phosphatidylserine (PS)	0.85	0.34	0.74	1.00	0.70	0.07	0.61	1.00	1.12	1.00	1.00	1.00	0.82	0.13	0.61	1.00	1.32	0.13	0.50	1.00	1.62	0.02	0.50	1.00
1-stearoyl-GPG (18:0)	Lipid	Lysophospholipid	0.81	0.17	0.60	1.00	0.94	0.49	0.87	1.00	0.78	0.05	0.57	1.00	1.15	0.12	0.61	1.00	0.96	0.87	0.95	1.00	0.84	0.02	0.50	1.00

2,2'-Methylenebis(6-tert-butyl-p-cresol)	Xenobiotics	Chemical	1.12	0.72	0.95	1.00	1.42	0.11	0.66	1.00	1.04	0.84	0.96	1.00	1.26	0.21	0.68	1.00	0.93	0.98	1.00	1.00	0.73	0.08	0.65	1.00
2,3-dihydroxy-2-methylbutyrate	Amino Acid	Leucine, Isoleucine and Val	0.81	0.03	0.36	1.00	0.93	0.39	0.82	1.00	0.86	0.10	0.64	1.00	1.15	0.40	0.79	1.00	1.07	0.46	0.77	1.00	0.93	0.67	0.92	1.00
2,3-dihydroxyisovalerate	Xenobiotics	Food Component/Plant	1.12	0.24	0.67	1.00	1.05	0.18	0.73	1.00	0.53	0.10	0.64	1.00	0.94	0.43	0.79	1.00	0.47	0.69	0.88	1.00	0.50	0.57	0.88	1.00
21-hydroxypregnenolone disulfate	Lipid	Pregnenolone Steroids	1.03	0.84	0.95	1.00	1.00	0.76	0.94	1.00	1.07	0.30	0.82	1.00	0.97	0.82	0.95	1.00	1.04	0.08	0.43	1.00	1.07	0.56	0.88	1.00
2-aminoadipate	Amino Acid	Lysine Metabolism	0.86	0.50	0.84	1.00	0.94	0.92	0.98	1.00	0.85	0.52	0.89	1.00	1.10	0.52	0.84	1.00	0.99	0.89	0.96	1.00	0.90	0.79	0.96	1.00
2-aminoheptanoate	Lipid	Fatty Acid, Amino	0.90	0.25	0.70	1.00	0.87	0.31	0.79	1.00	0.97	0.98	1.00	1.00	0.96	0.97	0.99	1.00	1.08	0.78	0.93	1.00	1.12	0.63	0.90	1.00
2-arachidonoylglycerol (20:4)	Lipid	Monoacylglycerol	1.00	0.94	0.99	1.00	1.00	0.84	0.95	1.00	1.12	0.48	0.89	1.00	1.00	0.95	0.99	1.00	1.12	0.15	0.52	1.00	1.12	0.33	0.79	1.00
2'-deoxyuridine	Nucleotide	Pyrimidine Metabolism, Ur	1.15	0.36	0.76	1.00	1.15	0.39	0.82	1.00	1.31	0.08	0.60	1.00	0.99	0.76	0.93	1.00	1.13	0.24	0.63	1.00	1.14	0.15	0.73	1.00
2-docosahexaenoylglycerol (22:6)*	Lipid	Monoacylglycerol	1.08	0.61	0.92	1.00	1.13	0.24	0.77	1.00	1.30	0.02	0.47	1.00	1.05	0.72	0.92	1.00	1.20	0.25	0.64	1.00	1.15	0.14	0.73	1.00
2-hydroxybehenate	Lipid	Fatty Acid, Monohydroxy	0.75	0.00	0.07	0.34	0.84	0.01	0.31	1.00	0.80	0.00	0.29	1.00	1.11	0.04	0.50	1.00	1.07	0.26	0.64	1.00	0.96	0.60	0.89	1.00
2-hydroxyhippurate (salicylurate)	Xenobiotics	Benzoate Metabolism	0.46	0.53	0.86	1.00	1.55	0.82	0.95	1.00	0.27	0.26	0.81	1.00	3.37	0.73	0.93	1.00	0.58	0.24	0.63	1.00	0.17	0.60	0.89	1.00
2-hydroxyphenylacetate	Amino Acid	Phenylalanine Metabolism	1.06	0.90	0.98	1.00	1.05	0.58	0.90	1.00	1.06	0.78	0.94	1.00	1.00	0.75	0.93	1.00	1.00	0.69	0.88	1.00	1.00	0.98	1.00	1.00
2-keto-3-deoxy-gluconate	Xenobiotics	Food Component/Plant	0.95	0.38	0.77	1.00	1.06	0.23	0.77	1.00	0.96	0.46	0.89	1.00	1.12	0.08	0.58	1.00	1.01	0.87	0.95	1.00	0.90	0.05	0.60	1.00
2-linoleoylglycerol (18:2)	Lipid	Monoacylglycerol	0.87	0.16	0.59	1.00	0.93	0.31	0.79	1.00	1.00	0.64	0.92	1.00	1.06	0.94	0.99	1.00	1.15	0.04	0.37	1.00	1.08	0.37	0.81	1.00
2-methylbutyrylcarnitine (C5)	Amino Acid	Leucine, Isoleucine and Val	0.97	0.50	0.84	1.00	0.99	0.87	0.96	1.00	1.02	0.92	0.98	1.00	1.02	0.58	0.87	1.00	1.05	0.86	0.95	1.00	1.03	0.75	0.94	1.00
2-myristoylglycerol (14:0)	Lipid	Monoacylglycerol	0.73	0.09	0.50	1.00	0.85	0.32	0.79	1.00	0.83	0.35	0.85	1.00	1.16	0.45	0.81	1.00	1.13	0.35	0.71	1.00	0.98	0.86	0.97	1.00
2-naphthol sulfate	Xenobiotics	Chemical	1.06	0.73	0.95	1.00	0.95	1.00	1.00	1.00	1.16	1.00	1.00	1.00	0.90	0.95	0.99	1.00	1.10	0.89	0.96	1.00	1.22	1.00	1.00	1.00
2'-O-methyluridine	Nucleotide	Pyrimidine Metabolism, Ur	1.15	0.01	0.23	1.00	1.19	0.02	0.42	1.00	1.23	0.00	0.29	1.00	1.04	0.29	0.72	1.00	1.07	0.02	0.31	1.00	1.03	0.24	0.75	1.00
2-oxoarginine*	Amino Acid	Urea cycle; Arginine and Pro	0.85	0.16	0.59	1.00	1.00	0.79	0.94	1.00	1.05	1.00	1.00	1.00	1.17	0.06	0.51	1.00	1.23	0.05	0.39	1.00	1.06	0.58	0.88	1.00
2-palmitoleoylglycerol (16:1)*	Lipid	Monoacylglycerol	0.90	0.73	0.95	1.00	1.00	0.95	0.99	1.00	0.98	0.98	1.00	1.00	1.10	0.60	0.87	1.00	1.09	0.25	0.64	1.00	0.99	0.35	0.80	1.00
2-palmitoleoyl-GPC* (16:1)*	Lipid	Lysophospholipid	1.00	0.84	0.95	1.00	1.36	0.16	0.71	1.00	1.39	0.07	0.58	1.00	1.35	0.11	0.61	1.00	1.39	0.08	0.43	1.00	1.02	0.90	0.98	1.00
3-(3-amino-3-carboxypropyl)uridine*	Nucleotide	Pyrimidine Metabolism, Ur	0.99	0.81	0.95	1.00	1.02	0.58	0.90	1.00	1.06	0.13	0.68	1.00	1.03	0.79	0.94	1.00	1.08	0.09	0.43	1.00	1.04	0.10	0.70	1.00
3-(3-hydroxyphenyl)propionate sulfate	Xenobiotics	Benzoate Metabolism	0.94	0.42	0.79	1.00	0.87	0.15	0.70	1.00	0.88	0.61	0.92	1.00	0.92	0.43	0.79	1.00	0.93	0.81	0.93	1.00	1.01	0.79	0.96	1.00
3,4-methyleneheptanoate	Xenobiotics	Food Component/Plant	0.75	0.03	0.36	1.00	0.92	0.45	0.85	1.00	0.85	0.09	0.60	1.00	1.22	0.46	0.82	1.00	1.14	0.15	0.52	1.00	0.93	0.64	0.91	1.00
3,7-dimethylurate	Xenobiotics	Xanthine Metabolism	0.71	0.23	0.66	1.00	0.95	0.79	0.94	1.00	0.92	0.57	0.90	1.00	1.34	0.30	0.73	1.00	1.28	0.33	0.69	1.00	0.96	0.67	0.92	1.00
3b-hydroxy-5-cholenoic acid	Lipid	Secondary Bile Acid Metabo	0.81	0.69	0.94	1.00	0.80	0.92	0.98	1.00	0.83	0.53	0.89	1.00	0.98	0.70	0.92	1.00	1.02	0.98	1.00	1.00	1.03	0.67	0.92	1.00
3-ethylcatechol sulfate (1)	Xenobiotics	Food Component/Plant	0.82	0.15	0.58	1.00	1.01	0.82	0.95	1.00	1.05	0.81	0.95	1.00	1.24	0.10	0.61	1.00	1.28	0.02	0.31	1.00	1.03	0.57	0.88	1.00
3-hydroxyadipate*	Lipid	Fatty Acid, Dicarboxylate	1.12	0.89	0.98	1.00	0.95	0.69	0.92	1.00	0.94	0.73	0.94	1.00	0.85	0.82	0.95	1.00	0.84	0.66	0.88	1.00	0.99	0.94	0.99	1.00
3-hydroxybutyroylglycine	Lipid	Fatty Acid Metabolism(Acyl	1.02	0.98	1.00	1.00	0.93	0.26	0.77	1.00	1.05	0.95	0.99	1.00	0.91	0.20	0.67	1.00	1.03	0.78	0.93	1.00	1.13	0.06	0.64	1.00
3-hydroxybutyrylcarnitine (1)	Lipid	Fatty Acid Metabolism(Acyl	1.14	0.11	0.52	1.00	0.97	0.86	0.95	1.00	0.87	0.31	0.82	1.00	0.85	0.12	0.61	1.00	0.76	0.06	0.40	1.00	0.90	0.95	0.99	1.00
3-hydroxybutyrylcarnitine (2)	Lipid	Fatty Acid Metabolism(Acyl	1.15	0.35	0.74	1.00	1.10	0.44	0.84	1.00	1.03	0.67	0.93	1.00	0.96	0.46	0.82	1.00	0.89	0.38	0.73	1.00	0.93	0.29	0.75	1.00
3-hydroxyhippurate sulfate	Xenobiotics	Benzoate Metabolism	1.00	0.82	0.95	1.00	0.84	0.29	0.78	1.00	0.90	0.72	0.94	1.00	0.84	0.48	0.82	1.00	0.90	0.84	0.94	1.00	1.07	0.66	0.92	1.00
3-hydroxyisobutyrate	Amino Acid	Leucine, Isoleucine and Val	0.89	0.32	0.73	1.00	0.92	0.42	0.83	1.00	0.92	0.25	0.80	1.00	1.03	0.54	0.85	1.00	1.03	0.92	0.98	1.00	1.00	0.53	0.87	1.00
3-hydroxymyristate	Lipid	Fatty Acid, Monohydroxy	1.08	0.44	0.80	1.00	1.08	0.57	0.89	1.00	1.05	0.63	0.92	1.00	1.01	0.81	0.95	1.00	0.98	0.78	0.93	1.00	0.97	0.58	0.88	1.00
3-hydroxysebacate	Lipid	Fatty Acid, Monohydroxy	0.98	0.69	0.94	1.00	0.64	0.03	0.51	1.00	0.47	0.04	0.54	1.00	0.66	0.13	0.61	1.00	0.48	0.24	0.63	1.00	0.74	0.78	0.95	1.00
3-hydroxystachydrine*	Xenobiotics	Food Component/Plant	0.84	0.95	0.99	1.00	0.58	0.76	0.94	1.00	0.92	1.00	1.00	1.00	0.69	0.69	0.91	1.00	1.10	1.00	1.00	1.00	1.58	0.54	0.87	1.00
3-methoxytyrosine	Amino Acid	Tyrosine Metabolism	0.93	0.24	0.67	1.00	1.02	0.81	0.95	1.00	1.06	0.56	0.89	1.00	1.09	0.27	0.70	1.00	1.14	0.12	0.48	1.00	1.04	0.97	1.00	1.00
3-methyl catechol sulfate (1)	Xenobiotics	Benzoate Metabolism	0.58	0.03	0.40	1.00	0.88	0.52	0.88	1.00	0.81	0.75	0.94	1.00	1.52	0.03	0.45	1.00	1.40	0.02	0.31	1.00	0.92	0.60	0.89	1.00
3-methyladipate	Lipid	Fatty Acid, Dicarboxylate	1.01	0.79	0.95	1.00	1.05	0.87	0.96	1.00	1.17	0.40	0.86	1.00	1.04	0.61	0.88	1.00	1.16	0.31	0.68	1.00	1.12	0.36	0.81	1.00
3-methylglutarylcarnitine (2)	Amino Acid	Leucine, Isoleucine and Val	1.03	0.69	0.94	1.00	1.07	0.05	0.53	1.00	1.23	0.05	0.57	1.00	1.04	0.57	0.87	1.00	1.19	0.04	0.37	1.00	1.15	0.26	0.75	1.00
3-methylxanthine	Xenobiotics	Xanthine Metabolism	0.84	0.42	0.79	1.00	0.97	0.95	0.99	1.00	0.98	0.95	0.99	1.00	1.16	0.38	0.79	1.00	1.17	0.37	0.72	1.00	1.00	0.98	1.00	1.00
3-phenylpropionate (hydrocinnamate)	Xenobiotics	Benzoate Metabolism	0.78	0.19	0.64	1.00	0.75	0.03	0.51	1.00	0.81	0.24	0.79	1.00	0.95	0.95	0.99	1.00	1.03	0.67	0.88	1.00	1.09	0.50	0.86	1.00
3-sulfo-L-alanine	Amino Acid	Methionine, Cysteine, SAM	0.96	0.73	0.95	1.00	1.18	0.31	0.79	1.00	1.17	0.21	0.76	1.00	1.23	0.09	0.60	1.00	1.22	0.03	0.31	1.00	0.99	0.76	0.95	1.00
4-cholesten-3-one	Lipid	Sterol	0.94	0.29	0.73	1.00	0.92	0.10	0.65	1.00	0.94	0.20	0.76	1.00	0.97	0.58	0.87	1.00	1.00	0.64	0.87	1.00	1.03	0.43	0.83	1.00
4-guanidinobutanoate	Amino Acid	Guanidino and Acetamido	0.73	0.16	0.59	1.00	0.89	1.00	1.00	1.00	0.83	0.56	0.89	1.00	1.22	0.02	0.44	1.00	1.14	0.67	0.88	1.00	0.94	0.58	0.88	1.00
4-hydroxycoumarin	Xenobiotics	Drug - Cardiovascular	0.88	0.73	0.95	1.00	1.02	0.82	0.95	1.00	0.87	0.90	0.98	1.00	1.16	0.76	0.93	1.00	0.98	0.50	0.80	1.00	0.85	0.64	0.91	1.00
4-hydroxyhippurate	Xenobiotics	Benzoate Metabolism	0.73	0.20	0.64	1.00	0.66	0.12	0.67	1.00	0.76	0.48	0.89	1.00	0.90	0.73	0.93	1.00	1.04	0.56	0.83	1.00	1.16	0.28	0.75	1.00
4-methoxyphenol sulfate	Amino Acid	Tyrosine Metabolism	0.82	0.08	0.50	1.00	0.91	0.28	0.77	1.00	1.02	0.98	1.00	1.00	1.10	0.34	0.75	1.00	1.24	0.13	0.50	1.00	1.12	0.19	0.73	1.00
4-methylguaiacol sulfate	Xenobiotics	Benzoate Metabolism	0.62	0.34	0.74	1.00	0.84	1.00	1.00	1.00	0.56	0.32	0.83	1.00	1.35	0.89	0.98	1.00	0.90	0.63	0.87	1.00	0.67	0.63	0.90	1.00
5-(galactosylhydroxy)-L-lysine	Amino Acid	Lysine Metabolism	0.98	0.43	0.80	1.00	0.95	0.56	0.89	1.00	1.08	0.17	0.75	1.00	0.97	0.73	0.93	1.00	1.10	0.07	0.41	1.00	1.14	0.01	0.50	1.00
5,6-dihydrothymine	Nucleotide	Pyrimidine Metabolism, Th	1.08	0.08	0.50	1.00	1.05	0.15	0.70	1.00	1.14	0.00	0.29	1.00	0.97	0.63	0.88	1.00	1.05	0.13	0.50	1.00	1.08	0.03	0.57	1.00
5,6-dihydrouracil	Nucleotide	Pyrimidine Metabolism, Ur	1.07	0.13	0.55	1.00	1.02	0.56	0.89	1.00	1.01	0.73	0.94	1.00	0.96	0.13	0.61	1.00	0.94	0.08	0.43	1.00	0.99	0.57	0.88	1.00
5-acetylamino-6-formylamino-3-methyluracil	Xenobiotics	Xanthine Metabolism	0.80	0.16	0.59	1.00	0.93	0.78	0.94	1.00	1.06	0.28	0.82	1.00	1.16	0.14	0.62	1.00	1.33	0.04	0.37	1.00	1.15	0.38	0.82	1.00
5alpha-androstan-3alpha,17beta-diol monosulfa	Lipid	Androgenic Steroids	1.12	0.09	0.50	1.00	1.08	0.42	0.83	1.00	1.04	0.81	0.95	1.00	0.96	0.46	0.82	1.00	0.93	0.13	0.50	1.00	0.96	0.15	0.73	1.00

5alpha-androstan-3beta,17alpha-diol disulfate	Lipid	Androgenic Steroids	1.02	0.81	0.95	1.00	1.09	0.82	0.95	1.00	0.97	0.64	0.92	1.00	1.06	0.52	0.84	1.00	0.95	0.86	0.95	1.00	0.89	0.38	0.82	1.00
5alpha-androstan-3beta,17beta-diol disulfate	Lipid	Androgenic Steroids	1.04	0.70	0.94	1.00	1.07	0.50	0.88	1.00	1.05	0.97	1.00	1.00	1.03	0.75	0.93	1.00	1.01	0.63	0.87	1.00	0.98	0.53	0.87	1.00
5alpha-pregnan-3beta,20alpha-diol disulfate	Lipid	Progestin Steroids	0.93	0.46	0.82	1.00	0.85	0.13	0.69	1.00	1.08	0.84	0.96	1.00	0.91	0.10	0.60	1.00	1.16	0.02	0.31	1.00	1.28	0.00	0.17	1.00
5alpha-pregnan-3beta,20alpha-diol monosulfate	Lipid	Progestin Steroids	0.91	0.66	0.93	1.00	0.80	0.01	0.39	1.00	0.96	0.86	0.96	1.00	0.88	0.04	0.50	1.00	1.06	0.60	0.86	1.00	1.20	0.00	0.43	1.00
5alpha-pregnan-3beta,20beta-diol monosulfate (	Lipid	Progestin Steroids	1.02	0.82	0.95	1.00	0.92	0.23	0.77	1.00	1.01	0.78	0.94	1.00	0.90	0.03	0.46	1.00	0.99	0.54	0.83	1.00	1.10	0.10	0.68	1.00
5-hydroxyhexanoate	Lipid	Fatty Acid, Monohydroxy	0.88	0.10	0.51	1.00	0.98	0.72	0.94	1.00	0.93	0.52	0.89	1.00	1.11	0.08	0.58	1.00	1.05	0.42	0.74	1.00	0.95	0.87	0.97	1.00
7-methylxanthine	Xenobiotics	Xanthine Metabolism	0.81	0.37	0.76	1.00	1.00	0.98	0.99	1.00	1.05	0.46	0.89	1.00	1.23	0.39	0.79	1.00	1.30	0.24	0.63	1.00	1.06	0.66	0.92	1.00
9,10-DiHOME	Lipid	Fatty Acid, Dihydroxy	0.74	0.14	0.57	1.00	0.75	0.07	0.61	1.00	0.65	0.07	0.58	1.00	1.01	0.60	0.87	1.00	0.89	0.33	0.69	1.00	0.87	0.38	0.82	1.00
adenine	Nucleotide	Purine Metabolism, Adenir	1.01	0.40	0.79	1.00	0.96	0.64	0.92	1.00	0.96	0.67	0.93	1.00	0.96	0.48	0.82	1.00	0.95	0.54	0.83	1.00	0.99	0.50	0.86	1.00
adipoylcarnitine (C6-DC)	Lipid	Fatty Acid Metabolism(Acyl	1.02	0.67	0.93	1.00	0.95	0.87	0.96	1.00	1.04	0.61	0.92	1.00	0.94	0.81	0.95	1.00	1.03	0.61	0.87	1.00	1.10	0.28	0.75	1.00
adrenoylcarnitine (C22:4)*	Lipid	Fatty Acid Metabolism(Acyl	1.25	0.20	0.64	1.00	1.09	0.48	0.86	1.00	1.10	0.67	0.93	1.00	0.87	0.15	0.63	1.00	0.88	0.05	0.39	1.00	1.01	0.98	1.00	1.00
alpha-ketobutyrate	Amino Acid	Methionine, Cysteine, SAM	1.02	0.87	0.97	1.00	0.79	0.02	0.49	1.00	0.86	0.19	0.76	1.00	0.77	0.01	0.44	1.00	0.84	0.06	0.39	1.00	1.09	0.34	0.80	1.00
andro steroid monosulfate C19H28O6S (1)*	Lipid	Androgenic Steroids	1.03	0.25	0.70	1.00	0.95	0.36	0.82	1.00	1.08	0.21	0.76	1.00	0.92	0.63	0.88	1.00	1.05	0.38	0.73	1.00	1.14	0.73	0.93	1.00
androstenediol (3alpha, 17alpha) monosulfate (:	Lipid	Androgenic Steroids	1.00	0.90	0.98	1.00	1.02	0.69	0.92	1.00	1.04	0.38	0.86	1.00	1.02	0.81	0.95	1.00	1.04	0.97	0.99	1.00	1.02	1.00	1.00	1.00
androstenediol (3beta,17beta) monosulfate (2)	Lipid	Androgenic Steroids	1.04	0.40	0.79	1.00	1.08	0.27	0.77	1.00	1.06	0.34	0.84	1.00	1.04	0.24	0.68	1.00	1.02	0.61	0.87	1.00	0.98	0.82	0.96	1.00
arabinose	Carbohydrate	Pentose Metabolism	1.24	0.08	0.50	1.00	1.22	0.34	0.80	1.00	1.36	0.05	0.57	1.00	0.98	0.84	0.96	1.00	1.10	0.57	0.85	1.00	1.11	0.56	0.88	1.00
arachidoylcarnitine (C20)*	Lipid	Fatty Acid Metabolism(Acyl	0.96	0.45	0.81	1.00	0.97	0.66	0.92	1.00	1.02	0.61	0.92	1.00	1.01	0.69	0.91	1.00	1.07	0.09	0.43	1.00	1.05	0.33	0.79	1.00
behenoylcarnitine (C22)*	Lipid	Fatty Acid Metabolism(Acyl	1.03	0.81	0.95	1.00	0.92	0.07	0.61	1.00	1.04	0.66	0.93	1.00	0.89	0.08	0.56	1.00	1.01	0.36	0.71	1.00	1.13	0.01	0.50	1.00
benzoate	Xenobiotics	Benzoate Metabolism	0.99	0.37	0.76	1.00	1.04	0.52	0.88	1.00	0.88	0.08	0.59	1.00	1.05	0.33	0.74	1.00	0.88	0.30	0.67	1.00	0.84	0.02	0.52	1.00
benzoylcarnitine*	Xenobiotics	Chemical	1.10	0.56	0.88	1.00	0.98	0.90	0.98	1.00	1.10	0.45	0.88	1.00	0.89	0.84	0.96	1.00	1.01	0.43	0.75	1.00	1.13	0.25	0.75	1.00
caproate (6:0)	Lipid	Medium Chain Fatty Acid	0.79	0.54	0.87	1.00	0.81	0.58	0.90	1.00	0.82	0.53	0.89	1.00	1.02	0.92	0.99	1.00	1.04	0.64	0.87	1.00	1.02	0.53	0.87	1.00
caprylate (8:0)	Lipid	Medium Chain Fatty Acid	0.78	0.33	0.74	1.00	0.88	0.81	0.95	1.00	0.85	0.94	0.99	1.00	1.12	0.52	0.84	1.00	1.09	0.33	0.69	1.00	0.97	0.45	0.83	1.00
carboxyethyl-GABA	Amino Acid	Glutamate Metabolism	1.00	0.98	1.00	1.00	0.91	0.22	0.77	1.00	0.91	0.11	0.65	1.00	0.91	0.43	0.79	1.00	0.91	0.20	0.60	1.00	0.99	0.98	1.00	1.00
carotene diol (3)	Cofactors and	Vitamin A Metabolism	1.04	0.73	0.95	1.00	1.10	0.66	0.92	1.00	1.13	0.37	0.85	1.00	1.06	0.90	0.98	1.00	1.08	0.48	0.78	1.00	1.02	0.45	0.83	1.00
ceramide (d18:1/17:0, d17:1/18:0)*	Lipid	Ceramides	1.08	0.37	0.76	1.00	1.02	0.81	0.95	1.00	0.99	0.72	0.94	1.00	0.94	0.23	0.68	1.00	0.92	0.04	0.36	1.00	0.97	0.66	0.92	1.00
phosphocholine	Lipid	Phospholipid Metabolism	1.00	0.94	0.99	1.00	1.00	0.67	0.92	1.00	1.02	0.69	0.94	1.00	0.99	0.97	0.99	1.00	1.02	0.69	0.88	1.00	1.03	0.58	0.88	1.00
corticosterone	Lipid	Corticosteroids	0.79	0.42	0.79	1.00	0.84	0.79	0.94	1.00	1.26	0.25	0.80	1.00	1.07	0.73	0.93	1.00	1.60	0.00	0.24	1.00	1.50	0.00	0.15	0.95
cortisone	Lipid	Corticosteroids	1.11	0.04	0.40	1.00	1.10	0.04	0.51	1.00	1.13	0.01	0.36	1.00	0.99	0.49	0.83	1.00	1.01	0.66	0.88	1.00	1.02	0.49	0.85	1.00
cysteine s-sulfate	Amino Acid	Methionine, Cysteine, SAM	0.69	0.00	0.17	1.00	0.60	0.00	0.29	1.00	0.49	0.00	0.00	0.00	0.87	0.21	0.67	1.00	0.71	0.01	0.31	1.00	0.82	0.10	0.68	1.00
cysteine sulfinic acid	Amino Acid	Methionine, Cysteine, SAM	1.01	1.00	1.00	1.00	1.04	0.78	0.94	1.00	1.06	0.42	0.87	1.00	1.03	0.56	0.86	1.00	1.05	0.63	0.87	1.00	1.02	0.66	0.92	1.00
cysteinylglycine	Amino Acid	Glutathione Metabolism	1.22	0.33	0.74	1.00	1.22	0.25	0.77	1.00	1.21	0.19	0.76	1.00	1.00	0.84	0.96	1.00	0.99	0.90	0.97	1.00	0.99	0.40	0.82	1.00
cytidine	Nucleotide	Pyrimidine Metabolism, Cy	1.16	0.32	0.73	1.00	1.12	0.43	0.83	1.00	1.16	0.23	0.77	1.00	0.96	1.00	1.00	1.00	1.00	0.46	0.77	1.00	1.03	0.61	0.90	1.00
deoxycholate	Lipid	Secondary Bile Acid Metabc	0.83	0.44	0.80	1.00	0.87	0.61	0.91	1.00	1.00	0.53	0.89	1.00	1.05	0.89	0.98	1.00	1.21	0.89	0.96	1.00	1.15	0.48	0.85	1.00
diacylglycerol (12:0/18:1, 14:0/16:1, 16:0/14:1	Lipid	Diacylglycerol	0.76	0.23	0.66	1.00	0.80	0.64	0.92	1.00	0.72	0.36	0.85	1.00	1.06	0.86	0.97	1.00	0.95	0.73	0.90	1.00	0.90	0.69	0.93	1.00
dihomo-linolenoylcarnitine (C20:3n3 or 6)*	Lipid	Fatty Acid Metabolism(Acyl	0.87	0.06	0.47	1.00	0.96	0.64	0.92	1.00	1.18	0.24	0.78	1.00	1.10	0.60	0.87	1.00	1.35	0.01	0.31	1.00	1.23	0.16	0.73	1.00
dihomo-linoleoylcarnitine (C20:2)*	Lipid	Fatty Acid Metabolism(Acyl	1.06	0.18	0.61	1.00	1.04	0.37	0.82	1.00	1.03	0.56	0.89	1.00	0.98	0.75	0.93	1.00	0.98	0.69	0.88	1.00	0.99	0.76	0.95	1.00
dihydrocaffeate sulfate (2)	Xenobiotics	Food Component/Plant	0.41	0.02	0.27	1.00	0.36	0.00	0.29	1.00	0.38	0.04	0.54	1.00	0.88	0.25	0.70	1.00	0.93	0.72	0.89	1.00	1.05	0.56	0.88	1.00
docosapentaenoate (n6 DPA; 22:5n6)	Lipid	Polyunsaturated Fatty Acid	0.93	0.46	0.82	1.00	1.23	0.20	0.75	1.00	1.04	0.94	0.99	1.00	1.32	0.02	0.44	1.00	1.11	0.24	0.63	1.00	0.85	0.26	0.75	1.00
docosapentaenoylcarnitine (C22:5n3)*	Lipid	Fatty Acid Metabolism(Acyl	1.17	0.12	0.54	1.00	1.17	0.18	0.73	1.00	1.13	0.38	0.86	1.00	1.00	1.00	1.00	1.00	0.96	0.67	0.88	1.00	0.96	0.87	0.97	1.00
ectoine	Xenobiotics	Chemical	1.20	0.90	0.98	1.00	2.11	0.04	0.51	1.00	0.63	0.76	0.94	1.00	1.76	0.26	0.70	1.00	0.53	0.60	0.86	1.00	0.30	0.04	0.57	1.00
eicosapentaenoylcholine	Lipid	Fatty Acid Metabolism (Acy	0.91	0.87	0.97	1.00	0.97	0.76	0.94	1.00	1.18	0.28	0.82	1.00	1.07	0.78	0.94	1.00	1.29	0.01	0.31	1.00	1.21	0.07	0.64	1.00
eicosenedioate (C20:1-DC)*	Lipid	Fatty Acid, Dicarboxylate	0.98	0.64	0.93	1.00	1.03	0.81	0.95	1.00	1.06	0.53	0.89	1.00	1.05	0.42	0.79	1.00	1.08	0.39	0.73	1.00	1.02	1.00	1.00	1.00
eicosenoylcarnitine (C20:1)*	Lipid	Fatty Acid Metabolism(Acyl	1.05	0.13	0.56	1.00	0.99	0.89	0.97	1.00	1.05	0.49	0.89	1.00	0.95	0.34	0.75	1.00	1.00	0.94	0.98	1.00	1.05	0.30	0.77	1.00
epiandrosterone sulfate	Lipid	Androgenic Steroids	1.02	0.34	0.74	1.00	1.03	0.69	0.92	1.00	1.02	0.87	0.97	1.00	1.01	0.95	0.99	1.00	1.00	0.73	0.90	1.00	1.00	0.76	0.95	1.00
ADSGEGDFXAEGGGVR*	Peptide	Fibrinogen Cleavage Peptide	1.07	0.84	0.95	1.00	1.24	0.39	0.82	1.00	1.37	0.06	0.57	1.00	1.15	0.58	0.87	1.00	1.27	0.02	0.31	1.00	1.10	0.11	0.70	1.00
ADpSGEGDFXAEGGGVR*	Peptide	Fibrinogen Cleavage Peptide	0.96	0.64	0.93	1.00	0.86	0.10	0.65	1.00	0.89	0.25	0.80	1.00	0.90	0.21	0.67	1.00	0.93	0.25	0.64	1.00	1.03	0.72	0.93	1.00
Fibrinopeptide B (1-11)	Peptide	Fibrinogen Cleavage Peptide	0.93	0.33	0.74	1.00	1.03	0.60	0.91	1.00	0.98	0.81	0.95	1.00	1.10	0.04	0.50	1.00	1.05	0.26	0.64	1.00	0.96	0.52	0.86	1.00
Fibrinopeptide B (1-9)	Peptide	Fibrinogen Cleavage Peptide	0.84	0.32	0.73	1.00	0.97	0.56	0.89	1.00	0.86	0.44	0.87	1.00	1.15	0.60	0.87	1.00	1.02	0.56	0.83	1.00	0.89	0.73	0.93	1.00
fumarate	Energy	TCA Cycle	0.94	0.67	0.93	1.00	0.97	0.86	0.95	1.00	1.02	0.61	0.92	1.00	1.03	0.16	0.64	1.00	1.09	0.20	0.60	1.00	1.05	0.64	0.91	1.00
galactonate	Carbohydrate	Fructose, Mannose and Gal	0.70	0.07	0.49	1.00	0.81	0.16	0.72	1.00	0.74	0.10	0.64	1.00	1.16	0.60	0.87	1.00	1.06	0.79	0.93	1.00	0.91	0.94	0.99	1.00
gamma-glutamyl-2-aminobutyrate	Peptide	Gamma-glutamyl Amino Ac	0.85	0.57	0.89	1.00	0.86	0.37	0.82	1.00	0.98	0.67	0.93	1.00	1.01	0.40	0.79	1.00	1.16	0.04	0.37	1.00	1.15	0.16	0.73	1.00
gamma-glutamylserine	Peptide	Gamma-glutamyl Amino Ac	1.06	0.16	0.59	1.00	1.04	0.45	0.85	1.00	1.08	0.04	0.57	1.00	0.98	0.66	0.90	1.00	1.02	0.35	0.71	1.00	1.04	0.15	0.73	1.00
gamma-glutamyltryptophan	Peptide	Gamma-glutamyl Amino Ac	1.06	0.10	0.51	1.00	1.09	0.11	0.66	1.00	1.13	0.01	0.36	1.00	1.03	0.38	0.79	1.00	1.07	0.03	0.35	1.00	1.04	0.15	0.73	1.00
gamma-glutamyltyrosine	Peptide	Gamma-glutamyl Amino Ac	0.96	0.66	0.93	1.00	1.01	0.79	0.94	1.00	1.04	0.22	0.76	1.00	1.06	0.31	0.73	1.00	1.09	0.04	0.37	1.00	1.03	0.90	0.98	1.00

gentisate	Amino Acid	Tyrosine Metabolism	0.97	0.82	0.95	1.00	1.27	0.79	0.94	1.00	0.77	0.11	0.65	1.00	1.31	0.84	0.96	1.00	0.79	0.24	0.63	1.00	0.60	0.18	0.73	1.00
glucuronide of piperine metabolite C17H21NO3	Xenobiotics	Food Component/Plant	1.15	0.23	0.66	1.00	1.06	0.37	0.82	1.00	0.99	0.87	0.97	1.00	0.92	0.98	0.99	1.00	0.86	0.72	0.89	1.00	0.94	0.56	0.88	1.00
glucuronide of piperine metabolite C17H21NO3	Xenobiotics	Food Component/Plant	1.08	0.86	0.96	1.00	1.04	0.39	0.82	1.00	0.99	0.89	0.98	1.00	0.96	0.45	0.81	1.00	0.91	0.97	0.99	1.00	0.95	0.43	0.83	1.00
glucuronide of piperine metabolite C17H21NO3	Xenobiotics	Food Component/Plant	1.27	0.06	0.47	1.00	1.09	0.20	0.75	1.00	1.01	0.97	1.00	1.00	0.86	0.70	0.92	1.00	0.80	0.54	0.83	1.00	0.93	0.52	0.86	1.00
glutarate (C5-DC)	Lipid	Fatty Acid, Dicarboxylate	0.95	0.98	1.00	1.00	0.95	0.86	0.95	1.00	0.97	0.86	0.96	1.00	1.00	0.92	0.99	1.00	1.02	0.72	0.89	1.00	1.03	0.61	0.90	1.00
glutaroylcarnitine (C5)	Amino Acid	Lysine Metabolism	1.01	0.72	0.95	1.00	1.02	0.52	0.88	1.00	1.03	0.29	0.82	1.00	1.01	0.89	0.98	1.00	1.02	0.36	0.71	1.00	1.02	0.45	0.83	1.00
glycerol 3-phosphate	Lipid	Glycerolipid Metabolism	1.03	0.69	0.94	1.00	1.02	0.46	0.85	1.00	0.94	0.58	0.91	1.00	0.99	0.94	0.99	1.00	0.91	0.08	0.43	1.00	0.92	0.21	0.73	1.00
glycerophosphoethanolamine	Lipid	Phospholipid Metabolism	1.02	0.61	0.92	1.00	0.95	0.86	0.95	1.00	1.01	0.79	0.95	1.00	0.93	0.82	0.95	1.00	0.99	0.25	0.64	1.00	1.06	0.40	0.82	1.00
glycerophosphoinositol*	Lipid	Phospholipid Metabolism	0.99	0.78	0.95	1.00	0.84	0.61	0.91	1.00	1.15	0.56	0.89	1.00	0.85	0.70	0.92	1.00	1.16	0.40	0.74	1.00	1.37	0.12	0.72	1.00
glycine conjugate of C10H14O2 (1)*	Partially Chara	Partially Characterized Mole	0.91	0.06	0.47	1.00	0.93	0.64	0.92	1.00	1.01	0.72	0.94	1.00	1.03	0.56	0.86	1.00	1.12	0.08	0.43	1.00	1.09	0.18	0.73	1.00
glyco-beta-muricholate	Lipid	Primary Bile Acid Metaboli	1.01	0.81	0.95	1.00	1.23	0.89	0.97	1.00	1.20	0.75	0.94	1.00	1.22	0.24	0.68	1.00	1.18	0.01	0.31	1.00	0.97	0.90	0.98	1.00
glycochenodeoxycholate 3-sulfate	Lipid	Primary Bile Acid Metaboli	0.76	0.31	0.73	1.00	0.85	0.22	0.77	1.00	0.89	0.58	0.91	1.00	1.13	0.54	0.85	1.00	1.17	0.36	0.71	1.00	1.04	0.45	0.83	1.00
glycochenodeoxycholate glucuronide (1)	Lipid	Primary Bile Acid Metaboli	0.75	0.09	0.50	1.00	0.79	0.03	0.51	1.00	1.00	0.50	0.89	1.00	1.06	0.72	0.92	1.00	1.34	0.19	0.59	1.00	1.26	0.11	0.70	1.00
glycodeoxycholate	Lipid	Secondary Bile Acid Metabo	0.64	0.08	0.50	1.00	1.05	0.07	0.60	1.00	0.88	0.92	0.98	1.00	1.64	0.61	0.88	1.00	1.37	0.13	0.50	1.00	0.84	0.20	0.73	1.00
glycodeoxycholate 3-sulfate	Lipid	Secondary Bile Acid Metabo	0.73	0.03	0.36	1.00	0.81	0.01	0.42	1.00	0.88	0.64	0.92	1.00	1.11	0.69	0.91	1.00	1.20	0.18	0.57	1.00	1.08	0.11	0.71	1.00
glycosyl ceramide (d18:1/23:1, d17:1/24:1)*	Lipid	Hexosylceramides (HCER)	1.05	0.48	0.82	1.00	0.93	0.25	0.77	1.00	0.99	0.89	0.98	1.00	0.89	0.10	0.61	1.00	0.95	0.61	0.87	1.00	1.07	0.69	0.93	1.00
glycosyl-N-(2-hydroxynervonoyl)-sphingosine (d1	Lipid	Hexosylceramides (HCER)	1.01	0.48	0.82	1.00	0.86	0.08	0.62	1.00	1.01	0.32	0.83	1.00	0.85	0.06	0.51	1.00	0.99	0.53	0.82	1.00	1.18	0.08	0.65	1.00
glycosyl-N-behenoyl-sphingadienine (d18:2/22:(	Lipid	Hexosylceramides (HCER)	1.02	1.00	1.00	1.00	0.95	0.19	0.75	1.00	1.04	0.98	1.00	1.00	0.93	0.24	0.69	1.00	1.02	0.75	0.92	1.00	1.10	0.03	0.57	1.00
glycosyl-N-tricosanoyl-sphingadienine (d18:2/23	Lipid	Hexosylceramides (HCER)	1.15	0.10	0.51	1.00	1.04	0.53	0.89	1.00	1.19	0.02	0.46	1.00	0.90	0.20	0.67	1.00	1.03	0.26	0.64	1.00	1.14	0.01	0.50	1.00
glycoursodeoxycholate	Lipid	Secondary Bile Acid Metabo	0.76	0.08	0.50	1.00	0.95	0.02	0.42	1.00	0.82	0.33	0.83	1.00	1.24	1.00	1.00	1.00	1.07	0.21	0.61	1.00	0.87	0.45	0.83	1.00
guanidinosuccinate	Amino Acid	Guanidino and Acetamido	1.06	0.33	0.74	1.00	1.02	0.58	0.90	1.00	1.01	0.98	1.00	1.00	0.96	0.53	0.84	1.00	0.95	0.42	0.74	1.00	0.99	0.87	0.97	1.00
gulonate*	Cofactors and	Ascorbate and Aldarate Met	0.92	0.34	0.74	1.00	1.00	0.42	0.83	1.00	0.95	0.58	0.91	1.00	1.08	0.13	0.62	1.00	1.03	0.46	0.77	1.00	0.95	0.18	0.73	1.00
heneicosapentaenoate (21:5n3)	Lipid	Polyunsaturated Fatty Acid	0.96	0.79	0.95	1.00	0.84	0.28	0.77	1.00	0.85	0.52	0.89	1.00	0.88	0.10	0.60	1.00	0.89	0.05	0.39	1.00	1.01	0.94	0.99	1.00
hexanoylglycine (C6)	Lipid	Fatty Acid Metabolism(Acyl	1.11	0.24	0.67	1.00	0.97	0.67	0.92	1.00	0.96	0.89	0.98	1.00	0.87	0.32	0.73	1.00	0.86	0.21	0.60	1.00	0.99	0.81	0.96	1.00
homocitrulline	Amino Acid	Urea cycle; Arginine and Pro	1.11	0.30	0.73	1.00	1.12	0.29	0.78	1.00	1.20	0.07	0.58	1.00	1.01	1.00	1.00	1.00	1.08	0.57	0.85	1.00	1.07	0.48	0.85	1.00
homovanillate (HVA)	Amino Acid	Tyrosine Metabolism	1.05	0.48	0.82	1.00	1.04	0.61	0.91	1.00	0.96	0.36	0.85	1.00	0.99	0.86	0.97	1.00	0.91	0.18	0.57	1.00	0.92	0.33	0.79	1.00
hydantoin-5-propionate	Amino Acid	Histidine Metabolism	0.91	0.33	0.74	1.00	0.93	0.38	0.82	1.00	0.98	0.89	0.98	1.00	1.02	0.90	0.98	1.00	1.08	0.61	0.87	1.00	1.06	0.82	0.96	1.00
hydroquinone sulfate	Xenobiotics	Drug - Topical Agents	0.65	0.32	0.73	1.00	0.70	0.39	0.82	1.00	0.75	0.44	0.87	1.00	1.09	0.73	0.93	1.00	1.15	0.37	0.72	1.00	1.06	0.54	0.87	1.00
hyocholate	Lipid	Secondary Bile Acid Metabo	0.91	0.81	0.95	1.00	1.08	0.35	0.82	1.00	1.18	0.42	0.87	1.00	1.19	0.97	0.99	1.00	1.30	0.86	0.95	1.00	1.09	0.54	0.87	1.00
imidazole propionate	Amino Acid	Histidine Metabolism	0.66	0.69	0.94	1.00	1.63	0.78	0.94	1.00	1.14	0.31	0.82	1.00	2.46	0.37	0.78	1.00	1.72	0.18	0.57	1.00	0.70	0.53	0.87	1.00
indole-3-carboxylate	Amino Acid	Tryptophan Metabolism	0.89	0.08	0.50	1.00	0.96	0.24	0.77	1.00	1.04	0.76	0.94	1.00	1.09	0.17	0.65	1.00	1.17	0.11	0.48	1.00	1.08	0.07	0.65	1.00
indoleacetylcarnitine*	Xenobiotics	Chemical	0.75	0.44	0.80	1.00	0.88	0.98	0.99	1.00	0.86	0.92	0.98	1.00	1.18	0.25	0.70	1.00	1.16	0.14	0.52	1.00	0.98	0.86	0.97	1.00
indoleacetylglutamine	Amino Acid	Tryptophan Metabolism	0.93	0.46	0.82	1.00	1.18	0.30	0.78	1.00	1.16	0.30	0.82	1.00	1.27	0.11	0.61	1.00	1.24	0.01	0.31	1.00	0.98	0.90	0.98	1.00
isobutyrylglycine (C4)	Amino Acid	Leucine, Isoleucine and Val	0.95	0.43	0.80	1.00	1.07	0.63	0.91	1.00	1.11	0.30	0.82	1.00	1.12	0.12	0.61	1.00	1.16	0.04	0.36	1.00	1.04	0.84	0.97	1.00
isocitrate	Energy	TCA Cycle	1.03	0.76	0.95	1.00	0.96	0.27	0.77	1.00	1.01	0.97	1.00	1.00	0.94	0.54	0.85	1.00	0.98	0.66	0.88	1.00	1.05	0.21	0.73	1.00
isoleucylglycine	Peptide	Dipeptide	0.77	0.12	0.55	1.00	1.15	0.57	0.89	1.00	1.08	0.38	0.86	1.00	1.49	0.02	0.44	1.00	1.40	0.04	0.37	1.00	0.94	0.92	0.98	1.00
isoursodeoxycholate	Lipid	Secondary Bile Acid Metabo	1.09	0.81	0.95	1.00	1.03	0.57	0.89	1.00	1.21	0.89	0.98	1.00	0.94	0.20	0.67	1.00	1.11	0.72	0.89	1.00	1.18	0.87	0.97	1.00
isovalerate (C5)	Amino Acid	Leucine, Isoleucine and Val	0.86	0.89	0.98	1.00	1.04	0.82	0.95	1.00	0.76	0.12	0.65	1.00	1.22	0.95	0.99	1.00	0.88	0.22	0.62	1.00	0.72	0.05	0.62	1.00
isovalerylglycine	Amino Acid	Leucine, Isoleucine and Val	0.86	0.04	0.40	1.00	1.06	0.76	0.94	1.00	1.15	0.25	0.80	1.00	1.24	0.01	0.44	1.00	1.35	0.01	0.31	1.00	1.08	0.16	0.73	1.00
lactosyl-N-behenoyl-sphingosine (d18:1/22:0)*	Lipid	Lactosylceramides (LCER)	0.95	0.78	0.95	1.00	0.79	0.38	0.82	1.00	0.98	0.98	1.00	1.00	0.83	0.11	0.61	1.00	1.04	1.00	1.00	1.00	1.25	0.40	0.82	1.00
leucylalanine	Peptide	Dipeptide	0.85	0.01	0.23	1.00	0.97	0.73	0.94	1.00	0.84	0.05	0.57	1.00	1.14	0.17	0.65	1.00	0.99	0.95	0.99	1.00	0.87	0.20	0.73	1.00
linoleoyl ethanolamide	Lipid	Endocannabinoid	0.96	0.29	0.73	1.00	1.04	0.54	0.89	1.00	0.97	0.92	0.98	1.00	1.08	0.08	0.58	1.00	1.01	0.39	0.73	1.00	0.93	0.23	0.75	1.00
linoleoyl-arachidonoyl-glycerol (18:2/20:4) [1]*	Lipid	Diacylglycerol	1.01	0.70	0.94	1.00	0.96	0.89	0.97	1.00	0.86	0.30	0.82	1.00	0.96	0.30	0.73	1.00	0.86	0.04	0.36	1.00	0.90	0.10	0.68	1.00
linoleoyl-docosahexaenoyl-glycerol (18:2/22:6) |	Lipid	Diacylglycerol	1.03	0.76	0.95	1.00	0.97	0.81	0.95	1.00	0.97	0.44	0.87	1.00	0.95	0.29	0.72	1.00	0.95	0.33	0.69	1.00	1.00	0.54	0.87	1.00
lithocholate sulfate (1)	Lipid	Secondary Bile Acid Metabo	1.05	0.79	0.95	1.00	0.95	0.35	0.82	1.00	1.12	0.60	0.92	1.00	0.90	0.61	0.88	1.00	1.07	0.81	0.93	1.00	1.19	0.19	0.73	1.00
maleate	Lipid	Fatty Acid, Dicarboxylate	1.17	0.03	0.40	1.00	1.13	0.24	0.77	1.00	0.42	0.06	0.57	1.00	0.97	0.14	0.62	1.00	0.36	0.01	0.31	1.00	0.37	0.79	0.96	1.00
malonate	Lipid	Fatty Acid Synthesis	0.84	0.45	0.81	1.00	0.88	0.24	0.77	1.00	0.94	0.64	0.92	1.00	1.05	0.95	0.99	1.00	1.12	0.09	0.43	1.00	1.06	0.36	0.81	1.00
methyl-4-hydroxybenzoate sulfate	Xenobiotics	Benzoate Metabolism	0.64	0.34	0.74	1.00	0.94	0.43	0.83	1.00	0.87	0.52	0.89	1.00	1.46	0.75	0.93	1.00	1.35	0.56	0.83	1.00	0.93	0.92	0.98	1.00
methylmalonate (MMA)	Lipid	Fatty Acid Metabolism (alsc	1.11	0.78	0.95	1.00	1.18	0.52	0.88	1.00	1.15	0.44	0.87	1.00	1.07	0.39	0.79	1.00	1.04	0.72	0.89	1.00	0.98	0.72	0.93	1.00
N-(2-furoyl)glycine	Xenobiotics	Food Component/Plant	0.66	0.02	0.33	1.00	1.50	0.15	0.70	1.00	0.76	0.86	0.96	1.00	2.27	0.11	0.61	1.00	1.15	0.02	0.31	1.00	0.50	0.15	0.73	1.00
N2,N5-diacetylornithine	Amino Acid	Urea cycle; Arginine and Pro	0.90	0.32	0.73	1.00	0.91	0.63	0.91	1.00	0.92	0.78	0.94	1.00	1.00	0.87	0.97	1.00	1.02	0.33	0.69	1.00	1.01	0.81	0.96	1.00
N2-acetyl,N6-methyllysine	Amino Acid	Lysine Metabolism	1.06	0.32	0.73	1.00	1.09	0.10	0.65	1.00	1.04	0.52	0.89	1.00	1.03	0.10	0.60	1.00	0.99	0.72	0.89	1.00	0.96	0.06	0.64	1.00
N4-acetylcytidine	Nucleotide	Pyrimidine Metabolism, Cy	1.02	0.72	0.95	1.00	1.05	0.33	0.79	1.00	0.94	0.84	0.96	1.00	1.03	0.78	0.94	1.00	0.92	0.05	0.39	1.00	0.90	0.12	0.72	1.00
N6-succinyladenosine	Nucleotide	Purine Metabolism, Adenir	1.07	0.16	0.59	1.00	1.01	0.70	0.93	1.00	0.94	0.22	0.76	1.00	0.95	0.23	0.68	1.00	0.88	0.00	0.31	1.00	0.93	0.08	0.65	1.00

N-acetyl-1-methylhistidine*	Amino Acid	Histidine Metabolism	1.05	0.98	1.00	1.00	1.15	0.23	0.77	1.00	1.03	0.76	0.94	1.00	1.09	0.30	0.73	1.00	0.98	0.58	0.85	1.00	0.89	0.21	0.73	1.00
N-acetylaspartate (NAA)	Amino Acid	Alanine and Aspartate Meta	0.90	0.13	0.56	1.00	1.09	0.94	0.98	1.00	1.05	0.94	0.99	1.00	1.21	0.00	0.31	1.00	1.17	0.03	0.31	1.00	0.97	0.24	0.75	1.00
N-acetylcarnosine	Amino Acid	Histidine Metabolism	1.03	0.43	0.80	1.00	1.03	0.56	0.89	1.00	0.95	0.81	0.95	1.00	1.00	0.86	0.97	1.00	0.92	0.26	0.64	1.00	0.93	0.21	0.73	1.00
N-acetylcitrulline	Amino Acid	Urea cycle; Arginine and Pro	0.92	0.30	0.73	1.00	1.05	0.48	0.86	1.00	1.07	0.75	0.94	1.00	1.15	0.14	0.62	1.00	1.17	0.72	0.89	1.00	1.02	0.92	0.98	1.00
N-acetylglucosamine/N-acetylgalactosamine	Carbohydrate	Aminosugar Metabolism	0.85	0.15	0.58	1.00	0.90	0.66	0.92	1.00	0.90	1.00	1.00	1.00	1.06	0.31	0.73	1.00	1.05	0.32	0.69	1.00	1.00	0.72	0.93	1.00
N-acetylhistidine	Amino Acid	Histidine Metabolism	0.81	0.12	0.54	1.00	1.03	0.75	0.94	1.00	0.95	0.81	0.95	1.00	1.27	0.03	0.46	1.00	1.17	0.23	0.62	1.00	0.92	0.35	0.80	1.00
N-acetylisoleucine	Amino Acid	Leucine, Isoleucine and Val	0.94	0.48	0.82	1.00	1.02	0.78	0.94	1.00	0.99	0.87	0.97	1.00	1.08	0.24	0.69	1.00	1.05	0.46	0.77	1.00	0.97	0.90	0.98	1.00
N-acetylneuraminate	Carbohydrate	Aminosugar Metabolism	1.16	0.08	0.50	1.00	1.17	0.00	0.29	1.00	0.99	1.00	1.00	1.00	1.01	0.84	0.96	1.00	0.85	0.05	0.39	1.00	0.85	0.03	0.57	1.00
N-acetylphenylalanine	Amino Acid	Phenylalanine Metabolism	0.89	0.15	0.58	1.00	0.98	0.37	0.82	1.00	0.97	0.58	0.91	1.00	1.11	0.52	0.84	1.00	1.09	0.04	0.36	1.00	0.99	0.27	0.75	1.00
N-acetyltyrosine	Amino Acid	Tyrosine Metabolism	0.94	0.28	0.72	1.00	1.04	0.66	0.92	1.00	1.15	0.05	0.57	1.00	1.10	0.02	0.44	1.00	1.21	0.00	0.24	1.00	1.11	0.05	0.60	1.00
N-carbamoylvaline	Amino Acid	Leucine, Isoleucine and Val	1.00	0.86	0.96	1.00	0.96	0.36	0.82	1.00	0.95	0.46	0.89	1.00	0.96	0.39	0.79	1.00	0.95	0.42	0.74	1.00	0.99	0.54	0.87	1.00
nervonoylcarnitine (C24:1)*	Lipid	Fatty Acid Metabolism(Acyl	0.96	0.37	0.76	1.00	0.87	0.04	0.51	1.00	0.98	0.81	0.95	1.00	0.90	0.10	0.60	1.00	1.02	0.42	0.74	1.00	1.13	0.17	0.73	1.00
N-formylanthranilic acid	Amino Acid	Tryptophan Metabolism	0.94	0.61	0.92	1.00	0.89	0.28	0.77	1.00	0.94	0.54	0.89	1.00	0.96	0.57	0.87	1.00	1.00	0.86	0.95	1.00	1.05	0.66	0.92	1.00
nicotinamide	Cofactors and	Nicotinate and Nicotinamid	0.28	0.81	0.95	1.00	0.25	0.39	0.82	1.00	0.29	0.75	0.94	1.00	0.90	0.27	0.70	1.00	1.04	0.97	0.99	1.00	1.15	0.12	0.72	1.00
nisinate (24:6n3)	Lipid	Polyunsaturated Fatty Acid	0.79	0.16	0.59	1.00	0.92	0.56	0.89	1.00	0.92	0.67	0.93	1.00	1.16	0.12	0.61	1.00	1.17	0.18	0.57	1.00	1.00	0.82	0.96	1.00
N-palmitoylserine	Lipid	Endocannabinoid	0.61	0.00	0.07	0.38	0.95	0.84	0.95	1.00	0.93	0.54	0.89	1.00	1.56	0.00	0.44	1.00	1.53	0.01	0.31	1.00	0.98	0.82	0.96	1.00
N-stearoylserine*	Lipid	Endocannabinoid	0.75	0.01	0.20	1.00	0.93	0.58	0.90	1.00	0.85	0.08	0.59	1.00	1.24	0.02	0.44	1.00	1.13	0.18	0.57	1.00	0.91	0.25	0.75	1.00
N-stearoyltaurine	Lipid	Endocannabinoid	0.83	0.16	0.59	1.00	0.91	0.39	0.82	1.00	0.86	0.24	0.78	1.00	1.09	0.20	0.67	1.00	1.03	1.00	1.00	1.00	0.94	0.28	0.75	1.00
o-cresol sulfate	Xenobiotics	Benzoate Metabolism	0.69	0.02	0.30	1.00	0.91	0.06	0.56	1.00	0.82	0.39	0.86	1.00	1.32	0.16	0.64	1.00	1.19	0.20	0.60	1.00	0.90	0.60	0.89	1.00
oleoyl-arachidonoyl-glycerol (18:1/20:4) [1]*	Lipid	Diacylglycerol	1.00	0.92	0.99	1.00	0.95	0.92	0.98	1.00	0.92	0.76	0.94	1.00	0.95	0.29	0.72	1.00	0.93	0.32	0.69	1.00	0.97	0.54	0.87	1.00
oleoyl-arachidonoyl-glycerol (18:1/20:4) [2]*	Lipid	Diacylglycerol	0.92	0.48	0.82	1.00	0.85	0.14	0.70	1.00	0.83	0.08	0.58	1.00	0.92	0.02	0.44	1.00	0.91	0.15	0.52	1.00	0.99	0.97	1.00	1.00
oleoyl-linolenoyl-glycerol (18:1/18:3) [2]*	Lipid	Diacylglycerol	0.74	0.16	0.59	1.00	0.66	0.02	0.42	1.00	0.72	0.19	0.76	1.00	0.89	0.18	0.66	1.00	0.96	0.66	0.88	1.00	1.08	0.78	0.95	1.00
palmitoleoyl-oleoyl-glycerol (16:1/18:1) [2]*	Lipid	Diacylglycerol	0.93	0.94	0.99	1.00	0.83	0.92	0.98	1.00	0.75	0.79	0.95	1.00	0.89	0.49	0.83	1.00	0.81	0.49	0.79	1.00	0.90	0.94	0.99	1.00
palmitoyl-arachidonoyl-glycerol (16:0/20:4) [1]*	Lipid	Diacylglycerol	0.91	0.78	0.95	1.00	0.97	0.40	0.82	1.00	0.95	0.39	0.86	1.00	1.06	0.67	0.91	1.00	1.04	0.89	0.96	1.00	0.98	0.86	0.97	1.00
palmitoyl-arachidonoyl-glycerol (16:0/20:4) [2]*	Lipid	Diacylglycerol	0.77	0.07	0.49	1.00	0.78	0.03	0.51	1.00	0.83	0.07	0.58	1.00	1.01	0.39	0.79	1.00	1.07	0.81	0.93	1.00	1.05	0.27	0.75	1.00
palmitoyl-linolenoyl-glycerol (16:0/18:3) [2]*	Lipid	Diacylglycerol	0.74	0.13	0.55	1.00	0.68	0.04	0.51	1.00	0.73	0.20	0.76	1.00	0.92	0.39	0.79	1.00	0.98	0.94	0.98	1.00	1.07	0.52	0.86	1.00
palmitoyl-linoleoyl-glycerol (16:0/18:2) [1]*	Lipid	Diacylglycerol	0.54	0.01	0.20	1.00	0.73	0.16	0.72	1.00	0.64	0.06	0.57	1.00	1.34	0.20	0.67	1.00	1.18	0.32	0.69	1.00	0.88	0.46	0.84	1.00
palmitoyl-linoleoyl-glycerol (16:0/18:2) [2]*	Lipid	Diacylglycerol	0.81	0.32	0.73	1.00	0.80	0.46	0.85	1.00	0.78	0.40	0.86	1.00	0.99	0.67	0.91	1.00	0.96	0.79	0.93	1.00	0.98	0.81	0.96	1.00
palmitoyl-myristoyl-glycerol (16:0/14:0) [2]	Lipid	Diacylglycerol	0.80	0.15	0.58	1.00	0.89	0.61	0.91	1.00	0.80	0.24	0.78	1.00	1.12	0.38	0.79	1.00	1.01	0.69	0.88	1.00	0.90	0.76	0.95	1.00
paraxanthine	Xenobiotics	Xanthine Metabolism	0.75	0.04	0.40	1.00	0.89	0.34	0.80	1.00	0.91	0.86	0.96	1.00	1.18	0.06	0.51	1.00	1.22	0.07	0.43	1.00	1.03	0.87	0.97	1.00
p-cresol glucuronide*	Amino Acid	Tyrosine Metabolism	0.83	0.53	0.86	1.00	0.91	0.97	0.99	1.00	1.09	0.22	0.76	1.00	1.10	0.10	0.61	1.00	1.31	0.09	0.43	1.00	1.19	0.42	0.82	1.00
phenylacetate	Amino Acid	Phenylalanine Metabolism	0.87	0.35	0.74	1.00	1.03	0.49	0.87	1.00	0.91	0.84	0.96	1.00	1.19	0.70	0.92	1.00	1.05	0.42	0.74	1.00	0.88	0.75	0.94	1.00
phenylacetylcarnitine	Peptide	Acetylated Peptides	0.75	0.14	0.57	1.00	0.96	0.61	0.91	1.00	0.81	0.44	0.87	1.00	1.29	0.33	0.74	1.00	1.08	0.69	0.88	1.00	0.84	0.78	0.95	1.00
phenylacetylglutamate	Peptide	Acetylated Peptides	0.90	0.37	0.76	1.00	1.03	0.89	0.97	1.00	1.08	0.43	0.87	1.00	1.14	0.43	0.79	1.00	1.20	0.12	0.48	1.00	1.06	0.81	0.96	1.00
phenylalanylglycine	Peptide	Dipeptide	0.96	0.66	0.93	1.00	1.06	0.63	0.91	1.00	1.02	0.82	0.96	1.00	1.10	0.36	0.77	1.00	1.06	0.48	0.78	1.00	0.97	0.97	1.00	1.00
phosphate	Energy	Oxidative Phosphorylation	1.10	0.24	0.67	1.00	1.20	0.00	0.24	0.60	1.14	0.05	0.57	1.00	1.09	0.21	0.68	1.00	1.03	0.58	0.85	1.00	0.95	0.43	0.83	1.00
picolinate	Amino Acid	Tryptophan Metabolism	0.89	0.24	0.67	1.00	0.97	0.33	0.79	1.00	0.78	0.06	0.57	1.00	1.10	0.60	0.87	1.00	0.88	0.40	0.74	1.00	0.80	0.32	0.78	1.00
piperine	Xenobiotics	Food Component/Plant	1.10	0.87	0.97	1.00	1.03	0.52	0.88	1.00	0.98	0.48	0.89	1.00	0.94	0.46	0.82	1.00	0.88	0.94	0.98	1.00	0.94	0.25	0.75	1.00
pregnanediol-3-glucuronide	Lipid	Progestin Steroids	1.04	0.19	0.64	1.00	0.96	0.60	0.91	1.00	1.13	0.06	0.57	1.00	0.92	0.24	0.68	1.00	1.08	0.06	0.39	1.00	1.18	0.01	0.50	1.00
pregnenolone sulfate	Lipid	Pregnenolone Steroids	0.85	0.06	0.47	1.00	0.92	0.21	0.76	1.00	1.02	0.63	0.92	1.00	1.08	0.42	0.79	1.00	1.20	0.02	0.31	1.00	1.11	0.04	0.57	1.00
pyrraline	Xenobiotics	Food Component/Plant	0.56	0.00	0.20	1.00	0.77	0.04	0.51	1.00	0.70	0.01	0.41	1.00	1.37	0.07	0.53	1.00	1.25	0.18	0.57	1.00	0.91	0.79	0.96	1.00
ribulonate/xylulonate*	Carbohydrate	Pentose Metabolism	0.69	0.02	0.30	1.00	0.79	0.13	0.69	1.00	0.70	0.02	0.47	1.00	1.15	0.22	0.68	1.00	1.02	0.46	0.77	1.00	0.89	0.15	0.73	1.00
S-1-pyrroline-5-carboxylate	Amino Acid	Glutamate Metabolism	0.98	0.79	0.95	1.00	0.90	0.26	0.77	1.00	0.95	0.70	0.94	1.00	0.91	0.58	0.87	1.00	0.97	0.76	0.92	1.00	1.06	0.19	0.73	1.00
S-adenosylhomocysteine (SAH)	Amino Acid	Methionine, Cysteine, SAM	1.02	0.49	0.83	1.00	1.06	0.32	0.79	1.00	1.07	0.06	0.57	1.00	1.04	0.64	0.89	1.00	1.06	0.30	0.67	1.00	1.02	0.81	0.96	1.00
sarcosine	Amino Acid	Glycine, Serine and Threoni	0.89	0.24	0.68	1.00	1.06	0.75	0.94	1.00	0.97	0.95	0.99	1.00	1.20	0.01	0.44	1.00	1.10	0.40	0.74	1.00	0.92	0.67	0.92	1.00
sphinganine	Lipid	Sphingolipid Synthesis	1.02	0.84	0.95	1.00	1.04	0.79	0.94	1.00	0.98	0.76	0.94	1.00	1.02	0.57	0.87	1.00	0.96	0.86	0.95	1.00	0.95	0.27	0.75	1.00
sphingomyelin (d18:1/25:0, d19:0/24:1, d20:1	Lipid	Sphingomyelins	1.01	0.95	0.99	1.00	0.87	0.04	0.51	1.00	1.04	0.40	0.86	1.00	0.85	0.03	0.46	1.00	1.03	0.39	0.73	1.00	1.21	0.00	0.15	0.55
stachydrine	Xenobiotics	Food Component/Plant	0.90	1.00	1.00	1.00	0.73	0.67	0.92	1.00	0.83	0.48	0.89	1.00	0.81	0.53	0.84	1.00	0.92	0.76	0.92	1.00	1.14	0.98	1.00	1.00
stearoyl-arachidonoyl-glycerol (18:0/20:4) [2]*	Lipid	Diacylglycerol	0.93	0.27	0.71	1.00	0.87	0.02	0.49	1.00	0.98	0.38	0.86	1.00	0.94	0.63	0.88	1.00	1.05	0.30	0.67	1.00	1.12	0.28	0.75	1.00
stearoylcholine*	Lipid	Fatty Acid Metabolism (Acy	0.99	0.94	0.99	1.00	0.99	0.95	0.99	1.00	1.13	0.29	0.82	1.00	0.99	0.97	0.99	1.00	1.13	0.06	0.39	1.00	1.14	0.21	0.73	1.00
suberate (C8-DC)	Lipid	Fatty Acid, Dicarboxylate	0.92	0.66	0.93	1.00	0.91	0.46	0.85	1.00	1.16	0.48	0.89	1.00	0.98	0.98	0.99	1.00	1.26	0.67	0.88	1.00	1.28	0.73	0.93	1.00
suberoylcarnitine (C8-DC)	Lipid	Fatty Acid Metabolism(Acyl	1.13	0.32	0.73	1.00	0.91	0.27	0.77	1.00	1.03	0.87	0.97	1.00	0.80	0.15	0.63	1.00	0.91	0.43	0.75	1.00	1.14	0.30	0.77	1.00
succinylcarnitine (C4)	Energy	TCA Cycle	1.07	0.18	0.61	1.00	1.04	0.42	0.83	1.00	1.01	0.36	0.85	1.00	0.97	0.54	0.85	1.00	0.95	0.22	0.62	1.00	0.98	0.58	0.88	1.00
sucrose	Carbohydrate	Disaccharides and Oligosac	0.10	0.18	0.61	1.00	0.11	0.28	0.77	1.00	0.20	0.92	0.98	1.00	1.11	0.78	0.94	1.00	2.02	0.16	0.54	1.00	1.82	0.57	0.88	1.00

sulfate of piperine metabolite C16H19NO3 (2)*	Xenobiotics	Food Component/Plant	1.04	1.00	1.00	1.00	1.07	0.50	0.88	1.00	1.02	0.97	1.00	1.00	1.03	0.50	0.84	1.00	0.98	0.66	0.88	1.00	0.95	0.60	0.89	1.00
sulfate of piperine metabolite C16H19NO3 (3)*	Xenobiotics	Food Component/Plant	1.04	0.87	0.97	1.00	1.06	0.52	0.88	1.00	1.03	0.75	0.94	1.00	1.01	0.53	0.84	1.00	0.98	0.64	0.87	1.00	0.97	0.57	0.88	1.00
tartarate	Xenobiotics	Food Component/Plant	0.93	0.84	0.95	1.00	1.04	0.78	0.94	1.00	0.84	0.50	0.89	1.00	1.13	0.57	0.87	1.00	0.90	0.90	0.97	1.00	0.80	0.92	0.98	1.00
taurodeoxycholate	Lipid	Secondary Bile Acid Metabo	0.67	0.06	0.47	1.00	0.88	0.16	0.71	1.00	0.68	0.60	0.92	1.00	1.31	0.34	0.75	1.00	1.02	0.37	0.72	1.00	0.78	0.37	0.81	1.00
taurolithocholate 3-sulfate	Lipid	Secondary Bile Acid Metabo	0.74	0.01	0.23	1.00	0.66	0.01	0.39	1.00	0.86	0.23	0.77	1.00	0.89	0.58	0.87	1.00	1.16	0.37	0.72	1.00	1.31	0.01	0.50	1.00
tetradecadienedioate (C14:2-DC)*	Lipid	Fatty Acid, Dicarboxylate	1.13	0.03	0.38	1.00	0.98	0.98	0.99	1.00	1.01	0.79	0.95	1.00	0.87	0.02	0.44	1.00	0.90	0.33	0.69	1.00	1.03	0.63	0.90	1.00
threonylphenylalanine	Peptide	Dipeptide	0.92	0.34	0.74	1.00	1.02	0.79	0.94	1.00	1.03	0.46	0.89	1.00	1.11	0.24	0.69	1.00	1.12	0.27	0.64	1.00	1.01	0.90	0.98	1.00
thymol sulfate	Xenobiotics	Food Component/Plant	1.59	0.30	0.73	1.00	1.10	0.37	0.82	1.00	1.35	0.66	0.93	1.00	0.69	0.69	0.91	1.00	0.84	0.34	0.70	1.00	1.22	0.76	0.95	1.00
tiglyl carnitine (C5)	Amino Acid	Leucine, Isoleucine and Val	0.98	0.76	0.95	1.00	1.01	0.76	0.94	1.00	0.98	0.63	0.92	1.00	1.03	0.45	0.81	1.00	0.99	0.43	0.75	1.00	0.97	0.75	0.94	1.00
trans-urocanate	Amino Acid	Histidine Metabolism	1.16	0.16	0.59	1.00	1.12	0.27	0.77	1.00	0.86	0.06	0.57	1.00	0.97	0.73	0.93	1.00	0.74	0.02	0.31	1.00	0.77	0.02	0.52	1.00
tryptophan betaine	Amino Acid	Tryptophan Metabolism	0.89	0.82	0.95	1.00	0.71	0.15	0.70	1.00	0.81	0.56	0.89	1.00	0.80	0.18	0.67	1.00	0.91	0.89	0.96	1.00	1.15	0.34	0.80	1.00
vanillactate	Amino Acid	Tyrosine Metabolism	0.85	0.09	0.50	1.00	0.99	0.98	0.99	1.00	0.95	0.92	0.98	1.00	1.16	0.02	0.44	1.00	1.12	0.08	0.43	1.00	0.97	0.89	0.98	1.00
xanthosine	Nucleotide	Purine Metabolism, (Hypo)	1.07	0.75	0.95	1.00	1.12	0.16	0.71	1.00	1.10	0.15	0.74	1.00	1.04	0.40	0.79	1.00	1.03	0.69	0.88	1.00	0.98	0.78	0.95	1.00
xylose	Carbohydrate	Pentose Metabolism	0.60	0.40	0.79	1.00	0.52	0.08	0.62	1.00	0.47	0.06	0.57	1.00	0.86	0.49	0.83	1.00	0.78	0.22	0.62	1.00	0.91	0.78	0.95	1.00
X - 11299	Unknown	Unknown	0.64	0.81	0.95	1.00	0.76	0.21	0.76	1.00	0.59	0.45	0.88	1.00	1.20	0.72	0.92	1.00	0.92	0.90	0.97	1.00	0.77	1.00	1.00	1.00
X - 11378	Unknown	Unknown	0.67	0.06	0.47	1.00	0.85	0.95	0.99	1.00	0.78	0.30	0.82	1.00	1.26	0.05	0.51	1.00	1.16	0.21	0.60	1.00	0.92	0.50	0.86	1.00
X - 11381	Unknown	Unknown	0.94	0.25	0.70	1.00	0.99	0.94	0.98	1.00	0.89	0.04	0.55	1.00	1.06	0.31	0.73	1.00	0.95	0.24	0.63	1.00	0.90	0.06	0.64	1.00
X - 11407	Unknown	Unknown	1.28	0.08	0.50	1.00	1.29	0.18	0.73	1.00	#####	0.02	0.44	1.00	1.01	0.90	0.98	1.00	8.45	0.18	0.57	1.00	8.35	0.18	0.73	1.00
X - 11441	Unknown	Unknown	1.71	0.38	0.77	1.00	1.17	0.32	0.79	1.00	1.39	0.27	0.82	1.00	0.68	0.94	0.99	1.00	0.81	0.98	1.00	1.00	1.19	0.40	0.82	1.00
X - 11442	Unknown	Unknown	1.54	0.45	0.81	1.00	1.11	0.69	0.92	1.00	1.32	0.50	0.89	1.00	0.72	0.44	0.80	1.00	0.86	0.84	0.94	1.00	1.19	0.20	0.73	1.00
X - 11478	Unknown	Unknown	0.96	0.79	0.95	1.00	0.90	0.18	0.73	1.00	1.02	0.73	0.94	1.00	0.93	0.42	0.79	1.00	1.06	0.69	0.88	1.00	1.14	0.21	0.73	1.00
X - 11483	Unknown	Unknown	0.56	0.30	0.73	1.00	0.70	0.66	0.92	1.00	0.58	0.66	0.93	1.00	1.26	0.97	0.99	1.00	1.05	0.72	0.89	1.00	0.83	0.75	0.94	1.00
X - 11491	Unknown	Unknown	1.20	0.03	0.40	1.00	1.12	0.22	0.77	1.00	1.17	0.10	0.62	1.00	0.93	0.43	0.79	1.00	0.98	0.82	0.94	1.00	1.05	0.42	0.82	1.00
X - 11522	Unknown	Unknown	1.07	1.00	1.00	1.00	0.89	0.69	0.92	1.00	1.09	0.75	0.94	1.00	0.83	0.08	0.58	1.00	1.02	0.70	0.89	1.00	1.22	0.08	0.65	1.00
X - 11843	Unknown	Unknown	0.59	0.04	0.42	1.00	0.83	0.17	0.72	1.00	0.97	0.50	0.89	1.00	1.40	0.27	0.70	1.00	1.65	0.04	0.37	1.00	1.18	0.33	0.79	1.00
X - 11850	Unknown	Unknown	0.72	0.01	0.23	1.00	0.87	0.13	0.69	1.00	0.90	0.40	0.86	1.00	1.22	0.25	0.70	1.00	1.25	0.07	0.41	1.00	1.03	0.35	0.80	1.00
X - 11852	Unknown	Unknown	1.10	0.37	0.76	1.00	0.73	0.87	0.96	1.00	1.29	0.86	0.96	1.00	0.67	0.92	0.99	1.00	1.16	0.11	0.47	1.00	1.75	0.21	0.73	1.00
X - 11880	Unknown	Unknown	0.99	0.78	0.95	1.00	1.05	0.57	0.89	1.00	1.00	0.61	0.92	1.00	1.06	0.52	0.84	1.00	1.02	0.97	0.99	1.00	0.96	0.28	0.75	1.00
X - 12007	Unknown	Unknown	0.33	0.00	0.06	0.07	0.61	0.10	0.65	1.00	0.49	0.02	0.44	1.00	1.83	0.01	0.44	1.00	1.45	0.03	0.35	1.00	0.79	0.24	0.75	1.00
X - 12013	Unknown	Unknown	0.66	0.13	0.55	1.00	0.92	0.46	0.85	1.00	1.00	0.72	0.94	1.00	1.40	0.40	0.79	1.00	1.52	0.21	0.60	1.00	1.09	0.45	0.83	1.00
X - 12101	Unknown	Unknown	1.14	0.08	0.50	1.00	1.07	0.18	0.73	1.00	1.13	0.17	0.75	1.00	0.94	0.95	0.99	1.00	0.99	0.54	0.83	1.00	1.05	0.36	0.81	1.00
X - 12111	Unknown	Unknown	0.86	0.67	0.93	1.00	0.55	0.43	0.83	1.00	0.94	0.53	0.89	1.00	0.63	0.60	0.87	1.00	1.09	0.67	0.88	1.00	1.72	0.78	0.95	1.00
X - 12117	Unknown	Unknown	1.08	0.27	0.71	1.00	1.16	0.18	0.73	1.00	1.10	0.30	0.82	1.00	1.07	0.79	0.94	1.00	1.02	0.81	0.93	1.00	0.95	0.87	0.97	1.00
X - 12126	Unknown	Unknown	1.03	0.95	0.99	1.00	1.21	0.60	0.91	1.00	1.28	0.06	0.57	1.00	1.17	0.75	0.93	1.00	1.24	0.09	0.43	1.00	1.06	0.11	0.71	1.00
X - 12170	Unknown	Unknown	0.93	0.67	0.93	1.00	1.05	0.82	0.95	1.00	0.91	0.57	0.90	1.00	1.13	0.24	0.69	1.00	0.98	0.72	0.89	1.00	0.86	0.57	0.88	1.00
X - 12212	Unknown	Unknown	0.44	0.45	0.81	1.00	0.60	0.73	0.94	1.00	0.54	0.19	0.76	1.00	1.37	0.29	0.72	1.00	1.24	0.43	0.75	1.00	0.90	0.07	0.65	1.00
X - 12283	Unknown	Unknown	1.15	0.28	0.72	1.00	0.96	0.31	0.79	1.00	1.10	0.72	0.94	1.00	0.83	0.16	0.64	1.00	0.95	0.70	0.89	1.00	1.14	0.54	0.87	1.00
X - 12680	Unknown	Unknown	0.64	0.06	0.47	1.00	0.93	0.79	0.94	1.00	0.82	0.58	0.91	1.00	1.45	0.01	0.44	1.00	1.27	0.04	0.37	1.00	0.88	0.17	0.73	1.00
X - 12707	Unknown	Unknown	0.90	0.45	0.81	1.00	1.08	0.21	0.75	1.00	0.97	0.50	0.89	1.00	1.20	0.02	0.44	1.00	1.07	0.43	0.75	1.00	0.90	0.18	0.73	1.00
X - 12718	Unknown	Unknown	1.04	0.67	0.93	1.00	1.11	0.27	0.77	1.00	1.20	0.05	0.57	1.00	1.07	0.70	0.92	1.00	1.15	0.11	0.48	1.00	1.08	0.28	0.75	1.00
X - 12739	Unknown	Unknown	1.22	0.05	0.43	1.00	0.97	0.69	0.92	1.00	1.26	0.04	0.54	1.00	0.80	0.22	0.68	1.00	1.04	0.60	0.86	1.00	1.30	0.01	0.50	1.00
X - 12798	Unknown	Unknown	0.94	0.21	0.65	1.00	1.01	0.82	0.95	1.00	0.93	0.39	0.86	1.00	1.08	0.44	0.80	1.00	0.99	0.81	0.93	1.00	0.92	0.40	0.82	1.00
X - 12812	Unknown	Unknown	1.00	0.76	0.95	1.00	0.79	0.31	0.79	1.00	0.92	0.92	0.98	1.00	0.79	0.29	0.72	1.00	0.92	0.89	0.96	1.00	1.17	0.09	0.67	1.00
X - 12816	Unknown	Unknown	0.86	0.09	0.50	1.00	1.00	0.76	0.94	1.00	1.06	0.94	0.99	1.00	1.17	0.14	0.62	1.00	1.24	0.08	0.43	1.00	1.06	0.36	0.81	1.00
X - 12822	Unknown	Unknown	1.05	0.48	0.82	1.00	1.02	0.92	0.98	1.00	1.07	0.63	0.92	1.00	0.97	0.21	0.68	1.00	1.02	0.75	0.92	1.00	1.06	0.60	0.89	1.00
X - 12847	Unknown	Unknown	0.68	0.97	0.99	1.00	1.44	0.54	0.89	1.00	1.70	0.56	0.89	1.00	2.13	0.45	0.81	1.00	2.51	0.42	0.74	1.00	1.18	0.78	0.95	1.00
X - 12849	Unknown	Unknown	1.38	0.11	0.52	1.00	0.97	1.00	1.00	1.00	1.20	0.21	0.76	1.00	0.70	0.14	0.62	1.00	0.87	0.29	0.67	1.00	1.24	0.26	0.75	1.00
X - 12851	Unknown	Unknown	0.54	0.09	0.50	1.00	0.61	0.04	0.51	1.00	0.55	0.21	0.76	1.00	1.14	0.19	0.67	1.00	1.02	0.90	0.97	1.00	0.89	0.16	0.73	1.00
X - 13684	Unknown	Unknown	1.06	0.34	0.74	1.00	1.00	0.56	0.89	1.00	0.93	0.21	0.76	1.00	0.94	0.43	0.79	1.00	0.88	0.10	0.45	1.00	0.94	0.40	0.82	1.00
X - 13728	Unknown	Unknown	0.71	0.07	0.48	1.00	0.92	0.76	0.94	1.00	0.93	0.50	0.89	1.00	1.30	0.23	0.68	1.00	1.32	0.24	0.63	1.00	1.02	0.90	0.98	1.00
X - 13737	Unknown	Unknown	0.99	0.34	0.74	1.00	1.01	0.28	0.77	1.00	1.07	0.07	0.58	1.00	1.01	0.64	0.89	1.00	1.07	0.13	0.50	1.00	1.06	0.16	0.73	1.00
X - 13835	Unknown	Unknown	0.93	0.78	0.95	1.00	0.82	0.48	0.86	1.00	0.84	0.56	0.89	1.00	0.88	0.78	0.94	1.00	0.91	0.87	0.95	1.00	1.03	0.87	0.97	1.00
X - 13844	Unknown	Unknown	0.84	0.43	0.80	1.00	0.96	0.81	0.95	1.00	1.03	0.97	1.00	1.00	1.14	0.26	0.70	1.00	1.22	0.23	0.62	1.00	1.07	0.39	0.82	1.00
X - 15461	Unknown	Unknown	1.02	0.97	0.99	1.00	1.03	0.57	0.89	1.00	1.12	0.13	0.69	1.00	1.01	0.70	0.92	1.00	1.10	0.43	0.75	1.00	1.09	0.39	0.82	1.00

X - 15666	Unknown	Unknown	0.93	0.58	0.90	1.00	0.98	0.32	0.79	1.00	0.92	0.50	0.89	1.00	1.06	0.86	0.97	1.00	0.99	0.97	0.99	1.00	0.94	0.49	0.85	1.00
X - 15674	Unknown	Unknown	1.08	0.67	0.93	1.00	0.93	0.70	0.93	1.00	0.96	0.90	0.98	1.00	0.86	0.16	0.64	1.00	0.90	0.48	0.78	1.00	1.04	0.75	0.94	1.00
X - 15728	Unknown	Unknown	0.52	0.63	0.93	1.00	0.95	0.76	0.94	1.00	0.59	0.39	0.86	1.00	1.85	0.84	0.96	1.00	1.14	0.97	0.99	1.00	0.61	0.95	0.99	1.00
X - 16397	Unknown	Unknown	1.07	0.48	0.82	1.00	0.99	0.94	0.98	1.00	1.06	0.60	0.92	1.00	0.93	0.03	0.46	1.00	0.99	0.56	0.83	1.00	1.07	0.37	0.81	1.00
X - 16570	Unknown	Unknown	1.04	0.38	0.77	1.00	1.02	0.63	0.91	1.00	1.20	0.08	0.59	1.00	0.98	0.58	0.87	1.00	1.16	0.31	0.68	1.00	1.18	0.10	0.68	1.00
X - 16654	Unknown	Unknown	0.72	0.25	0.70	1.00	0.69	0.28	0.77	1.00	0.79	0.87	0.97	1.00	0.96	0.78	0.94	1.00	1.10	0.36	0.71	1.00	1.15	0.34	0.80	1.00
X - 16946	Unknown	Unknown	1.16	0.66	0.93	1.00	1.04	0.38	0.82	1.00	1.16	0.53	0.89	1.00	0.89	0.63	0.88	1.00	1.00	0.79	0.93	1.00	1.12	0.90	0.98	1.00
X - 16964	Unknown	Unknown	1.09	0.37	0.76	1.00	1.06	0.43	0.83	1.00	1.15	0.16	0.75	1.00	0.97	0.78	0.94	1.00	1.06	0.18	0.57	1.00	1.09	0.08	0.65	1.00
X - 17010	Unknown	Unknown	0.75	0.01	0.20	1.00	0.95	0.36	0.82	1.00	0.91	0.12	0.65	1.00	1.26	0.09	0.60	1.00	1.21	0.03	0.35	1.00	0.96	0.25	0.75	1.00
X - 17145	Unknown	Unknown	0.95	0.97	0.99	1.00	0.83	0.32	0.79	1.00	0.95	0.57	0.90	1.00	0.88	0.28	0.72	1.00	1.00	0.67	0.88	1.00	1.14	0.33	0.79	1.00
X - 17146	Unknown	Unknown	0.74	0.14	0.57	1.00	1.01	0.78	0.94	1.00	0.81	0.76	0.94	1.00	1.37	0.24	0.69	1.00	1.10	0.24	0.63	1.00	0.80	0.58	0.88	1.00
X - 17185	Unknown	Unknown	0.42	0.02	0.30	1.00	0.84	0.39	0.82	1.00	0.59	0.40	0.86	1.00	1.98	0.07	0.53	1.00	1.40	0.03	0.35	1.00	0.71	0.33	0.79	1.00
X - 17325	Unknown	Unknown	0.71	0.06	0.47	1.00	0.62	0.04	0.51	1.00	0.62	0.07	0.58	1.00	0.88	0.87	0.97	1.00	0.88	0.64	0.87	1.00	1.00	0.31	0.78	1.00
X - 17327	Unknown	Unknown	1.22	0.03	0.39	1.00	0.96	0.90	0.98	1.00	1.14	0.56	0.89	1.00	0.79	0.03	0.45	1.00	0.93	0.73	0.90	1.00	1.19	0.32	0.78	1.00
X - 17351	Unknown	Unknown	1.07	0.64	0.93	1.00	0.86	0.05	0.54	1.00	0.97	0.70	0.94	1.00	0.81	0.10	0.60	1.00	0.91	0.26	0.64	1.00	1.13	0.52	0.86	1.00
X - 17354	Unknown	Unknown	1.24	0.92	0.99	1.00	0.87	0.42	0.83	1.00	1.03	0.50	0.89	1.00	0.71	0.13	0.62	1.00	0.83	1.00	1.00	1.00	1.18	0.31	0.78	1.00
X - 17359	Unknown	Unknown	1.03	0.95	0.99	1.00	1.09	0.09	0.64	1.00	1.00	0.61	0.92	1.00	1.06	0.32	0.73	1.00	0.97	0.64	0.87	1.00	0.92	0.32	0.78	1.00
X - 17367	Unknown	Unknown	0.76	0.23	0.66	1.00	0.60	0.09	0.65	1.00	0.68	0.16	0.75	1.00	0.80	0.56	0.86	1.00	0.90	0.72	0.89	1.00	1.13	0.25	0.75	1.00
X - 17438	Unknown	Unknown	0.90	0.72	0.95	1.00	0.94	0.60	0.91	1.00	1.02	0.84	0.96	1.00	1.05	0.82	0.95	1.00	1.14	0.52	0.82	1.00	1.08	0.37	0.81	1.00
X - 17612	Unknown	Unknown	0.91	0.61	0.92	1.00	0.96	0.87	0.96	1.00	1.27	0.86	0.96	1.00	1.05	0.50	0.84	1.00	1.40	0.46	0.77	1.00	1.33	0.95	0.99	1.00
X - 18345	Unknown	Unknown	0.80	0.84	0.95	1.00	0.93	0.53	0.89	1.00	1.25	0.78	0.94	1.00	1.17	0.15	0.63	1.00	1.56	0.24	0.63	1.00	1.34	0.87	0.97	1.00
X - 18886	Unknown	Unknown	1.02	0.97	0.99	1.00	0.91	0.26	0.77	1.00	1.00	0.81	0.95	1.00	0.90	0.04	0.50	1.00	0.98	0.58	0.85	1.00	1.10	0.04	0.57	1.00
X - 18899	Unknown	Unknown	0.89	0.09	0.50	1.00	1.07	0.30	0.78	1.00	1.13	0.87	0.97	1.00	1.20	0.02	0.44	1.00	1.27	0.08	0.43	1.00	1.06	0.72	0.93	1.00
X - 18901	Unknown	Unknown	3.12	0.18	0.61	1.00	1.84	0.07	0.60	1.00	2.15	0.97	1.00	1.00	0.59	0.70	0.92	1.00	0.69	0.90	0.97	1.00	1.17	0.89	0.98	1.00
X - 21364	Unknown	Unknown	1.04	0.39	0.78	1.00	1.02	0.42	0.83	1.00	1.08	0.12	0.65	1.00	0.98	0.75	0.93	1.00	1.04	0.33	0.69	1.00	1.06	0.04	0.57	1.00
X - 21441	Unknown	Unknown	1.08	0.11	0.53	1.00	1.02	0.44	0.84	1.00	1.05	0.73	0.94	1.00	0.95	0.49	0.83	1.00	0.97	0.61	0.87	1.00	1.03	0.63	0.90	1.00
X - 21442	Unknown	Unknown	0.79	0.18	0.62	1.00	0.87	0.73	0.94	1.00	0.90	0.67	0.93	1.00	1.11	0.49	0.83	1.00	1.14	0.15	0.52	1.00	1.03	0.31	0.78	1.00
X - 21448	Unknown	Unknown	1.21	0.28	0.72	1.00	1.05	0.40	0.82	1.00	1.18	0.24	0.78	1.00	0.86	0.16	0.64	1.00	0.98	0.73	0.90	1.00	1.13	0.20	0.73	1.00
X - 21467	Unknown	Unknown	1.06	0.28	0.72	1.00	1.03	0.50	0.88	1.00	1.11	0.36	0.85	1.00	0.97	0.58	0.87	1.00	1.05	0.40	0.74	1.00	1.08	0.37	0.81	1.00
X - 21470	Unknown	Unknown	0.97	0.50	0.84	1.00	0.93	0.90	0.98	1.00	1.08	0.39	0.86	1.00	0.95	0.89	0.98	1.00	1.11	0.57	0.85	1.00	1.17	0.19	0.73	1.00
X - 21471	Unknown	Unknown	0.95	0.26	0.71	1.00	0.95	0.56	0.89	1.00	0.98	0.76	0.94	1.00	1.00	0.82	0.95	1.00	1.03	0.82	0.94	1.00	1.03	0.92	0.98	1.00
X - 21607	Unknown	Unknown	1.05	0.34	0.74	1.00	1.03	0.67	0.92	1.00	1.07	0.24	0.79	1.00	0.98	0.50	0.84	1.00	1.03	0.64	0.87	1.00	1.05	0.26	0.75	1.00
X - 21729	Unknown	Unknown	0.95	1.00	1.00	1.00	1.14	0.30	0.78	1.00	0.91	0.46	0.89	1.00	1.20	0.78	0.94	1.00	0.96	0.58	0.85	1.00	0.80	0.97	1.00	1.00
X - 21752	Unknown	Unknown	0.93	0.97	0.99	1.00	0.95	0.98	0.99	1.00	1.03	0.52	0.89	1.00	1.02	0.42	0.79	1.00	1.11	0.37	0.72	1.00	1.09	0.61	0.90	1.00
X - 21792	Unknown	Unknown	1.16	0.01	0.23	1.00	1.02	0.38	0.82	1.00	1.07	0.42	0.87	1.00	0.88	0.00	0.31	0.95	0.92	0.03	0.31	1.00	1.05	0.29	0.75	1.00
X - 21821	Unknown	Unknown	1.07	0.57	0.89	1.00	0.95	0.13	0.69	1.00	1.04	0.87	0.97	1.00	0.89	0.24	0.68	1.00	0.97	0.64	0.87	1.00	1.10	0.52	0.86	1.00
X - 22508	Unknown	Unknown	0.97	0.86	0.96	1.00	1.14	0.45	0.85	1.00	1.21	0.11	0.65	1.00	1.18	0.48	0.82	1.00	1.25	0.21	0.61	1.00	1.06	0.70	0.93	1.00
X - 22509	Unknown	Unknown	0.93	0.86	0.96	1.00	1.25	0.38	0.82	1.00	0.89	0.70	0.94	1.00	1.34	0.09	0.60	1.00	0.95	0.72	0.89	1.00	0.71	0.31	0.78	1.00
X - 23196	Unknown	Unknown	0.53	0.00	0.11	1.00	0.58	0.04	0.51	1.00	0.69	0.10	0.64	1.00	1.11	0.46	0.82	1.00	1.31	0.03	0.31	1.00	1.19	0.05	0.60	1.00
X - 23276	Unknown	Unknown	0.92	0.87	0.97	1.00	1.05	0.39	0.82	1.00	1.05	0.42	0.87	1.00	1.14	0.10	0.60	1.00	1.13	0.12	0.49	1.00	0.99	0.98	1.00	1.00
X - 23296	Unknown	Unknown	1.31	0.01	0.20	1.00	1.16	0.26	0.77	1.00	1.08	0.79	0.95	1.00	0.88	0.18	0.66	1.00	0.82	0.04	0.36	1.00	0.93	0.46	0.84	1.00
X - 23297	Unknown	Unknown	1.18	0.04	0.40	1.00	1.10	0.34	0.80	1.00	1.09	0.32	0.83	1.00	0.93	0.70	0.92	1.00	0.92	0.30	0.67	1.00	0.99	0.42	0.82	1.00
X - 23587	Unknown	Unknown	0.98	0.95	0.99	1.00	1.22	0.69	0.92	1.00	1.21	0.21	0.76	1.00	1.24	0.72	0.92	1.00	1.22	0.23	0.62	1.00	0.99	0.52	0.86	1.00
X - 23641	Unknown	Unknown	1.23	0.64	0.93	1.00	1.19	0.34	0.80	1.00	1.19	0.46	0.89	1.00	0.97	0.42	0.79	1.00	0.97	0.40	0.74	1.00	1.00	0.84	0.97	1.00
X - 23644	Unknown	Unknown	3.61	0.87	0.97	1.00	0.64	0.76	0.94	1.00	1.68	0.95	0.99	1.00	0.18	0.36	0.77	1.00	0.47	0.92	0.98	1.00	2.64	0.15	0.73	1.00
X - 23659	Unknown	Unknown	1.08	0.21	0.66	1.00	1.04	0.38	0.82	1.00	1.07	0.37	0.85	1.00	0.96	0.97	0.99	1.00	0.99	0.70	0.89	1.00	1.03	0.94	0.99	1.00
X - 23665	Unknown	Unknown	1.18	0.38	0.77	1.00	1.28	0.18	0.73	1.00	1.04	0.79	0.95	1.00	1.09	0.44	0.80	1.00	0.88	0.43	0.75	1.00	0.81	0.16	0.73	1.00
X - 23666	Unknown	Unknown	1.06	0.35	0.74	1.00	1.04	0.38	0.82	1.00	1.05	0.31	0.82	1.00	0.98	0.69	0.91	1.00	0.99	0.89	0.96	1.00	1.01	0.76	0.95	1.00
X - 23739	Unknown	Unknown	0.97	0.32	0.73	1.00	0.97	0.70	0.93	1.00	1.00	0.76	0.94	1.00	0.99	0.67	0.91	1.00	1.03	0.58	0.85	1.00	1.04	0.35	0.80	1.00
X - 23782	Unknown	Unknown	1.28	0.01	0.20	1.00	1.10	0.30	0.78	1.00	1.07	0.43	0.87	1.00	0.86	0.02	0.44	1.00	0.84	0.01	0.31	1.00	0.98	0.49	0.85	1.00
X - 23787	Unknown	Unknown	1.03	0.58	0.90	1.00	1.07	0.72	0.94	1.00	1.06	0.44	0.87	1.00	1.04	0.76	0.93	1.00	1.03	0.81	0.93	1.00	0.99	0.81	0.96	1.00
X - 24243	Unknown	Unknown	1.11	1.00	1.00	1.00	1.24	0.13	0.69	1.00	1.20	0.03	0.54	1.00	1.12	0.53	0.84	1.00	1.07	0.30	0.67	1.00	0.96	0.50	0.86	1.00
X - 24293	Unknown	Unknown	1.09	0.72	0.95	1.00	1.21	0.82	0.95	1.00	1.19	0.78	0.94	1.00	1.11	0.53	0.84	1.00	1.08	0.94	0.98	1.00	0.98	0.48	0.85	1.00
X - 24295	Unknown	Unknown	1.13	0.23	0.66	1.00	0.95	0.66	0.92	1.00	0.83	0.40	0.86	1.00	0.84	0.06	0.53	1.00	0.73	0.01	0.31	1.00	0.87	0.07	0.64	1.00

X - 24328	Unknown	Unknown	0.97	0.52	0.85	1.00	1.00	0.76	0.94	1.00	1.00	0.37	0.85	1.00	1.04	0.97	0.99	1.00	1.03	0.61	0.87	1.00	0.99	0.49	0.85	1.00
X - 24337	Unknown	Unknown	0.97	0.63	0.93	1.00	1.00	0.60	0.91	1.00	0.97	0.72	0.94	1.00	1.03	0.76	0.93	1.00	1.00	0.92	0.98	1.00	0.97	1.00	1.00	1.00
X - 24455	Unknown	Unknown	0.95	0.58	0.90	1.00	0.86	0.20	0.75	1.00	0.76	0.09	0.60	1.00	0.90	0.25	0.70	1.00	0.80	0.05	0.39	1.00	0.89	0.90	0.98	1.00
X - 24473	Unknown	Unknown	1.08	0.35	0.74	1.00	1.05	0.48	0.86	1.00	0.78	0.21	0.76	1.00	0.97	0.24	0.68	1.00	0.73	0.16	0.54	1.00	0.75	0.38	0.82	1.00
X - 24475	Unknown	Unknown	0.84	0.24	0.67	1.00	0.99	0.73	0.94	1.00	1.06	0.75	0.94	1.00	1.19	0.52	0.84	1.00	1.27	0.09	0.43	1.00	1.07	0.98	1.00	1.00
X - 24494	Unknown	Unknown	1.05	0.75	0.95	1.00	1.02	0.61	0.91	1.00	1.08	0.23	0.77	1.00	0.97	0.35	0.76	1.00	1.02	0.69	0.88	1.00	1.06	0.10	0.68	1.00
X - 24527	Unknown	Unknown	1.22	0.23	0.66	1.00	0.96	0.95	0.99	1.00	1.26	0.13	0.68	1.00	0.79	0.30	0.73	1.00	1.03	0.61	0.87	1.00	1.31	0.02	0.50	1.00
X - 24542	Unknown	Unknown	0.47	0.00	0.20	1.00	0.44	0.00	0.24	0.95	0.49	0.01	0.39	1.00	0.93	0.39	0.79	1.00	1.04	0.18	0.57	1.00	1.12	0.72	0.93	1.00
X - 24544	Unknown	Unknown	1.04	0.43	0.80	1.00	0.99	0.66	0.92	1.00	1.05	0.54	0.89	1.00	0.95	0.53	0.84	1.00	1.01	0.43	0.75	1.00	1.06	0.37	0.81	1.00
X - 24556	Unknown	Unknown	0.96	0.82	0.95	1.00	0.97	0.86	0.95	1.00	1.02	0.84	0.96	1.00	1.01	0.92	0.99	1.00	1.06	0.45	0.77	1.00	1.05	0.60	0.89	1.00
X - 24571	Unknown	Unknown	1.20	0.66	0.93	1.00	1.06	0.92	0.98	1.00	0.75	0.35	0.85	1.00	0.88	0.61	0.88	1.00	0.62	0.86	0.95	1.00	0.71	0.44	0.83	1.00
X - 24686	Unknown	Unknown	0.81	0.17	0.60	1.00	0.85	0.24	0.77	1.00	0.74	0.10	0.63	1.00	1.05	0.87	0.97	1.00	0.91	0.21	0.60	1.00	0.87	0.37	0.81	1.00
X - 24748	Unknown	Unknown	0.84	0.15	0.58	1.00	1.01	0.70	0.93	1.00	0.97	0.81	0.95	1.00	1.20	0.05	0.51	1.00	1.15	0.13	0.50	1.00	0.96	0.63	0.90	1.00
X - 24757	Unknown	Unknown	0.76	0.08	0.50	1.00	0.60	0.02	0.49	1.00	0.68	0.04	0.55	1.00	0.79	0.54	0.85	1.00	0.90	0.45	0.77	1.00	1.15	0.32	0.78	1.00
X - 24809	Unknown	Unknown	1.00	0.26	0.71	1.00	1.05	0.46	0.85	1.00	0.96	0.53	0.89	1.00	1.05	0.66	0.90	1.00	0.95	0.07	0.43	1.00	0.91	0.34	0.80	1.00
X - 24811	Unknown	Unknown	0.79	0.21	0.66	1.00	0.95	1.00	1.00	1.00	0.97	0.73	0.94	1.00	1.20	0.12	0.61	1.00	1.23	0.15	0.52	1.00	1.03	0.97	1.00	1.00
X - 24849	Unknown	Unknown	1.01	0.66	0.93	1.00	0.86	0.73	0.94	1.00	1.00	0.98	1.00	1.00	0.85	0.23	0.68	1.00	0.99	0.57	0.85	1.00	1.17	0.30	0.77	1.00
X - 24947	Unknown	Unknown	1.11	0.16	0.59	1.00	1.07	0.21	0.75	1.00	1.09	0.11	0.65	1.00	0.97	0.36	0.77	1.00	0.99	0.64	0.87	1.00	1.02	0.81	0.96	1.00
X - 24949	Unknown	Unknown	1.07	0.56	0.88	1.00	0.97	0.70	0.93	1.00	1.01	1.00	1.00	1.00	0.90	0.97	0.99	1.00	0.94	0.95	0.99	1.00	1.04	0.63	0.90	1.00
X - 24972	Unknown	Unknown	0.91	0.72	0.95	1.00	1.02	0.86	0.95	1.00	0.96	0.63	0.92	1.00	1.12	0.53	0.84	1.00	1.06	0.39	0.73	1.00	0.94	0.63	0.90	1.00
X - 25271	Unknown	Unknown	0.62	0.52	0.85	1.00	1.20	0.69	0.92	1.00	0.76	0.64	0.92	1.00	1.95	0.82	0.95	1.00	1.24	0.48	0.78	1.00	0.63	0.42	0.82	1.00
X - 25343	Unknown	Unknown	1.05	0.44	0.80	1.00	0.84	0.35	0.82	1.00	0.91	0.79	0.95	1.00	0.80	0.05	0.51	1.00	0.87	0.25	0.64	1.00	1.09	0.97	1.00	1.00
X - 25419	Unknown	Unknown	0.99	0.69	0.94	1.00	1.19	0.15	0.70	1.00	0.96	0.78	0.94	1.00	1.20	0.58	0.87	1.00	0.97	0.69	0.88	1.00	0.80	0.42	0.82	1.00
X - 25420	Unknown	Unknown	1.07	0.78	0.95	1.00	1.19	0.21	0.76	1.00	1.26	0.08	0.58	1.00	1.11	0.03	0.46	1.00	1.18	0.46	0.77	1.00	1.06	0.63	0.90	1.00
X - 25519	Unknown	Unknown	0.99	0.81	0.95	1.00	1.07	0.38	0.82	1.00	0.96	0.76	0.94	1.00	1.08	0.89	0.98	1.00	0.97	0.56	0.83	1.00	0.90	0.75	0.94	1.00
X - 25520	Unknown	Unknown	1.02	0.58	0.90	1.00	0.98	0.92	0.98	1.00	0.82	0.38	0.86	1.00	0.97	0.52	0.84	1.00	0.81	0.20	0.60	1.00	0.84	0.27	0.75	1.00
