# Supplementary material for: Metabolic Alterations in Older Women With Low Bone Mineral Density Supplemented With Lactobacillus reuteri
Source: JBMR Plus. 2021 Mar 15;5(4):e10478. doi: 10.1002/jbm4.10478 (PMC8046097; doi:10.1002/jbm4.10478)
Supplement: Supplementary file 4 — Table S2: Metabolites responding differentially at any one time point between L. reuteri and placebo groups, identified by using the cut‐off of both VIP score of >1 in the PLS‐DA model and p value >0.05 in the Wilcoxon rank‐sum test. [file JBM4-5-e10478-s001.pdf]

Supplemental Table 2

			L.reuteri vs Placebo																	
			3M/0M						6M/0M						12M/0M					
Biochemical Name	Super Pathw	Sub Pathway	Mean ratio of L.reuter	Mean ratio of Placebo	P value	VIP	Adj.P FDR	Adj.P Bonf.	Mean ratio of L.reuter	Mean ratio of Pl	P value	VIP	Adj.P FDR	Adj.P Bonf.	Mean ratio of L.reuter	Mean ratio of Placebo	P value	VIP	Adj.P FDR	Adj.P Bonf.
(16 or 17)-methylstearate (a19:0 or i19:0)	Lipid	Fatty Acid, Branched	1.07	1.17	0.73	1.25	1.00	1.00	1.11	1.14	0.95	0.77	1.00	1.00	1.06	1.11	0.78	0.77	0.99	1.00
(N(1) + N(8))-acetylspermidine	Amino Acid	Polyamine Metabolism	1.13	1.10	0.75	0.85	1.00	1.00	1.11	1.04	0.27	0.97	0.85	1.00	1.18	1.22	0.65	0.74	0.99	1.00
1-(1-enyl-palmitoyl)-2-arachidonoyl-GPC (P-16:	Lipid	Plasmalogen	0.98	1.02	0.58	1.16	1.00	1.00	1.00	1.05	0.73	0.85	1.00	1.00	1.06	1.05	0.86	0.42	0.99	1.00
1-(1-enyl-palmitoyl)-2-arachidonoyl-GPE (P-16:	Lipid	Plasmalogen	0.96	1.04	0.47	0.96	1.00	1.00	1.09	1.02	0.63	0.57	1.00	1.00	1.10	1.06	0.98	0.77	0.99	1.00
1-(1-enyl-palmitoyl)-2-linoleoyl-GPC (P-16:0/1	Lipid	Plasmalogen	0.99	1.01	0.97	1.02	1.00	1.00	1.01	1.03	0.91	0.72	1.00	1.00	1.06	1.02	0.81	0.94	0.99	1.00
1-(1-enyl-palmitoyl)-2-linoleoyl-GPE (P-16:0/1	Lipid	Plasmalogen	1.02	1.00	0.87	0.64	1.00	1.00	1.16	1.03	0.27	1.11	0.85	1.00	1.15	1.06	0.40	1.11	0.99	1.00
1-(1-enyl-palmitoyl)-2-oleoyl-GPC (P-16:0/18:1	Lipid	Plasmalogen	1.02	1.01	0.72	0.83	1.00	1.00	0.98	1.01	0.29	0.94	0.86	1.00	1.04	1.00	0.41	1.04	0.99	1.00
1-(1-enyl-palmitoyl)-2-oleoyl-GPE (P-16:0/18:1	Lipid	Plasmalogen	0.96	0.98	0.92	0.99	1.00	1.00	1.04	1.02	0.56	0.58	0.97	1.00	1.08	1.00	0.15	1.14	0.99	1.00
1-(1-enyl-palmitoyl)-2-palmitoleoyl-GPC (P-16:	Lipid	Plasmalogen	1.04	1.08	0.66	0.96	1.00	1.00	1.03	1.04	0.88	0.61	1.00	1.00	1.08	1.05	0.38	0.55	0.99	1.00
1-(1-enyl-palmitoyl)-2-palmitoyl-GPC (P-16:0/1	Lipid	Plasmalogen	1.02	1.03	0.90	0.95	1.00	1.00	0.97	1.01	0.18	1.01	0.79	1.00	1.05	1.02	0.89	0.84	0.99	1.00
1-(1-enyl-palmitoyl)-GPC (P-16:0)*	Lipid	Lysoplasmalogen	1.03	1.11	0.52	1.11	1.00	1.00	1.00	1.09	0.71	1.21	1.00	1.00	1.09	1.10	0.58	0.99	0.99	1.00
1-(1-enyl-palmitoyl)-GPE (P-16:0)*	Lipid	Lysoplasmalogen	1.06	1.14	0.90	0.93	1.00	1.00	1.11	1.04	0.70	0.64	1.00	1.00	1.16	1.11	0.75	0.94	0.99	1.00
1-(1-enyl-stearoyl)-2-arachidonoyl-GPE (P-18:0/	Lipid	Plasmalogen	0.96	1.04	0.53	1.01	1.00	1.00	1.02	1.08	0.64	0.70	1.00	1.00	1.06	1.04	0.82	0.67	0.99	1.00
1-(1-enyl-stearoyl)-2-linoleoyl-GPE (P-18:0/18:	Lipid	Plasmalogen	0.99	0.97	0.52	0.78	1.00	1.00	1.05	1.03	0.70	0.46	1.00	1.00	1.10	1.02	0.22	1.04	0.99	1.00
1-(1-enyl-stearoyl)-2-oleoyl-GPE (P-18:0/18:1)	Lipid	Plasmalogen	0.98	1.01	0.67	0.89	1.00	1.00	1.04	1.02	0.68	0.51	1.00	1.00	1.10	1.05	0.44	0.73	0.99	1.00
1-(1-enyl-stearoyl)-GPE (P-18:0)*	Lipid	Lysoplasmalogen	1.04	1.17	0.91	1.05	1.00	1.00	1.09	1.05	0.65	0.54	1.00	1.00	1.14	1.12	0.52	0.84	0.99	1.00
1,2-dilinoleoyl-GPC (18:2/18:2)	Lipid	Phosphatidylcholine (PC)	0.96	0.99	0.89	1.29	1.00	1.00	1.00	1.05	0.71	0.96	1.00	1.00	1.06	1.06	0.87	0.77	0.99	1.00
1,2-dipalmitoyl-GPC (16:0/16:0)	Lipid	Phosphatidylcholine (PC)	0.95	0.97	0.32	1.33	1.00	1.00	0.93	0.97	0.34	1.21	0.88	1.00	0.99	0.97	0.91	0.67	0.99	1.00
1,3-dimethylurate	Xenobiotics	Xanthine Metabolism	0.92	0.98	0.55	0.91	1.00	1.00	1.23	1.03	0.17	1.08	0.79	1.00	1.41	1.11	0.31	1.30	0.99	1.00
1,5-anhydroglucitol (1,5-AG)	Carbohydrat	Glycolysis, Gluconeogenesis, a	1.01	1.01	0.64	1.20	1.00	1.00	1.01	1.00	0.93	1.26	1.00	1.00	1.02	1.03	0.85	1.09	0.99	1.00
1,7-dimethylurate	Xenobiotics	Xanthine Metabolism	0.90	0.96	0.64	0.97	1.00	1.00	1.10	0.95	0.29	0.98	0.87	1.00	1.30	1.06	0.87	1.03	0.99	1.00
10-heptadecenoate (17:1n7)	Lipid	Long Chain Fatty Acid	1.42	1.74	0.81	1.15	1.00	1.00	1.29	1.50	0.92	1.00	1.00	1.00	1.28	1.26	0.92	1.12	0.99	1.00
10-nonadecenoate (19:1n9)	Lipid	Long Chain Fatty Acid	1.30	1.53	0.98	1.21	1.00	1.00	1.25	1.30	0.96	0.97	1.00	1.00	1.22	1.17	0.76	1.13	0.99	1.00
10-undecenoate (11:1n1)	Lipid	Medium Chain Fatty Acid	1.18	1.17	0.51	0.30	1.00	1.00	1.11	1.11	0.42	0.45	0.90	1.00	1.04	1.04	0.76	0.25	0.99	1.00
13-HODE + 9-HODE	Lipid	Fatty Acid, Monohydroxy	1.07	0.94	0.17	0.91	1.00	1.00	1.02	1.08	0.94	0.35	1.00	1.00	1.12	0.87	0.14	1.58	0.99	1.00
16a-hydroxy DHEA 3-sulfate	Lipid	Androgenic Steroids	1.09	1.06	0.61	0.56	1.00	1.00	1.08	0.98	0.31	0.83	0.88	1.00	1.11	1.07	0.42	0.41	0.99	1.00
16-hydroxypalmitate	Lipid	Fatty Acid, Monohydroxy	1.16	1.09	0.19	0.84	1.00	1.00	1.13	1.16	0.88	0.34	1.00	1.00	1.15	1.05	0.18	1.35	0.99	1.00
1-arachidonoyl-GPC* (20:4)*	Lipid	Lysophospholipid	1.02	1.04	0.53	1.06	1.00	1.00	1.01	1.05	0.51	0.80	0.95	1.00	1.07	1.06	0.68	0.93	0.99	1.00
1-arachidonoyl-GPE (20:4n6)*	Lipid	Lysophospholipid	1.00	0.91	0.15	1.36	1.00	1.00	0.99	0.95	0.14	0.72	0.78	1.00	1.04	1.02	0.67	0.96	0.99	1.00
1-arachidonoyl-GPI* (20:4)*	Lipid	Lysophospholipid	0.92	1.01	0.67	0.70	1.00	1.00	1.04	1.06	0.61	0.20	1.00	1.00	1.00	1.02	0.86	0.39	0.99	1.00
1-arachidonylglycerol (20:4)	Lipid	Monoacylglycerol	1.21	1.12	0.97	0.57	1.00	1.00	1.29	1.18	0.93	0.39	1.00	1.00	1.35	1.07	0.14	1.17	0.99	1.00
1-carboxyethylphenylalanine	Amino Acid	Phenylalanine Metabolism	0.86	0.87	0.82	1.00	1.00	1.00	1.07	1.02	0.59	1.08	0.98	1.00	0.93	0.88	0.60	0.81	0.99	1.00
1-dihomo-linolenylglycerol (20:3)	Lipid	Monoacylglycerol	1.19	1.13	0.59	0.34	1.00	1.00	1.43	1.28	0.65	0.44	1.00	1.00	1.31	1.08	0.67	1.06	0.99	1.00
1-docosahexaenoylglycerol (22:6)	Lipid	Monoacylglycerol	1.06	1.16	0.77	0.60	1.00	1.00	1.02	1.23	0.18	1.09	0.79	1.00	1.11	1.06	0.27	0.43	0.99	1.00
1-linolenoyl-GPC (18:3)*	Lipid	Lysophospholipid	1.06	1.03	0.65	1.03	1.00	1.00	1.07	1.19	0.55	1.27	0.97	1.00	1.21	1.14	0.89	0.90	0.99	1.00
1-linoleoyl-2-linolenoyl-GPC (18:2/18:3)*	Lipid	Phosphatidylcholine (PC)	0.89	1.02	0.40	1.51	1.00	1.00	0.95	1.24	0.03	1.93	0.73	1.00	1.09	1.17	0.60	0.92	0.99	1.00
1-linoleoylglycerol (18:2)	Lipid	Monoacylglycerol	1.23	1.08	0.76	0.60	1.00	1.00	1.28	1.03	0.75	0.81	1.00	1.00	1.38	1.01	0.16	1.27	0.99	1.00
1-linoleoyl-GPC (18:2)	Lipid	Lysophospholipid	1.00	0.99	0.49	1.25	1.00	1.00	1.01	1.00	0.57	0.74	0.97	1.00	1.06	1.02	0.54	1.18	0.99	1.00
1-linoleoyl-GPE (18:2)*	Lipid	Lysophospholipid	0.96	0.80	0.03	1.61	1.00	1.00	0.99	0.92	0.12	0.89	0.76	1.00	1.03	0.94	0.36	1.28	0.99	1.00
1-linoleoyl-GPI* (18:2)*	Lipid	Lysophospholipid	0.92	1.00	0.77	0.72	1.00	1.00	1.11	1.08	0.59	0.49	0.98	1.00	1.00	1.07	0.58	0.59	0.99	1.00
1-methyl-4-imidazoleacetate	Amino Acid	Histidine Metabolism	1.25	0.99	0.02	1.60	1.00	1.00	1.26	1.11	0.01	0.59	0.73	1.00	1.15	1.00	0.02	1.68	0.86	1.00
1-methylhistidine	Amino Acid	Histidine Metabolism	1.14	0.96	0.28	1.12	1.00	1.00	1.11	1.06	0.56	0.62	0.97	1.00	1.23	1.13	0.50	0.91	0.99	1.00
1-methylnicotinamide	Cofactors ar	Nicotinate and Nicotinamide I	1.10	1.06	0.71	0.21	1.00	1.00	1.24	1.07	0.75	0.71	1.00	1.00	1.15	1.24	0.61	0.41	0.99	1.00
1-myristoyl-2-arachidonoyl-GPC (14:0/20:4)*	Lipid	Phosphatidylcholine (PC)	0.99	0.93	0.28	0.77	1.00	1.00	1.04	1.15	0.77	0.91	1.00	1.00	1.09	1.09	0.95	0.55	0.99	1.00
1-myristoyl-2-palmitoyl-GPC (14:0/16:0)	Lipid	Phosphatidylcholine (PC)	0.98	0.92	0.48	0.84	1.00	1.00	0.97	1.09	0.74	1.17	1.00	1.00	1.09	1.11	0.85	0.65	0.99	1.00
1-myristoylglycerol (14:0)	Lipid	Monoacylglycerol	1.02	0.97	1.00	0.33	1.00	1.00	1.12	1.18	0.96	0.75	1.00	1.00	1.14	1.23	0.71	0.45	0.99	1.00
1-oleoyl-2-docosahexaenoyl-GPC (18:1/22:6)*	Lipid	Phosphatidylcholine (PC)	1.00	1.00	0.99	1.12	1.00	1.00	0.96	1.00	0.34	0.83	0.88	1.00	0.97	1.03	0.54	1.34	0.99	1.00
1-oleoyl-2-linoleoyl-GPE (18:1/18:2)*	Lipid	Phosphatidylethanolamine (PI	1.02	0.73	0.04	1.62	1.00	1.00	1.05	0.94	0.20	0.95	0.81	1.00	1.12	0.85	0.12	1.77	0.99	1.00
1-oleoylglycerol (18:1)	Lipid	Monoacylglycerol	1.09	1.03	0.59	0.53	1.00	1.00	1.06	0.99	0.69	0.45	1.00	1.00	1.08	1.02	0.89	0.48	0.99	1.00

1-oleoyl-GPC (18:1)	Lipid	Lysophospholipid	1.04	1.01	0.50	1.25	1.00	1.00	1.04	1.03	0.81	0.73	1.00	1.00	1.08	1.06	0.65	1.14	0.99	1.00
1-oleoyl-GPE (18:1)	Lipid	Lysophospholipid	0.94	0.82	0.15	1.27	1.00	1.00	0.98	0.96	0.31	0.95	0.88	1.00	1.08	0.97	0.44	1.27	0.99	1.00
1-oleoyl-GPI (18:1)*	Lipid	Lysophospholipid	0.99	1.04	0.96	0.29	1.00	1.00	1.16	1.29	0.55	0.46	0.97	1.00	1.13	1.13	0.77	0.04	0.99	1.00
1-palmitoleoylglycerol (16:1)*	Lipid	Monoacylglycerol	1.25	1.08	0.57	0.77	1.00	1.00	1.26	1.10	0.65	0.56	1.00	1.00	1.34	1.06	0.37	1.05	0.99	1.00
1-palmitoleoyl-GPC* (16:1)*	Lipid	Lysophospholipid	1.06	1.05	0.70	1.15	1.00	1.00	1.06	1.11	0.58	0.76	0.98	1.00	1.14	1.09	0.87	0.93	0.99	1.00
1-palmitoyl-2-arachidonoyl-GPC (16:0/20:4n6	Lipid	Phosphatidylcholine (PC)	0.97	0.96	0.88	1.26	1.00	1.00	0.97	0.98	0.69	0.79	1.00	1.00	1.01	0.99	0.66	0.76	0.99	1.00
1-palmitoyl-2-arachidonoyl-GPE (16:0/20:4)*	Lipid	Phosphatidylethanolamine (PI	1.07	0.90	0.02	2.01	1.00	1.00	1.04	0.98	0.34	0.80	0.88	1.00	1.04	1.02	0.87	0.73	0.99	1.00
1-palmitoyl-2-arachidonoyl-GPI (16:0/20:4)*	Lipid	Phosphatidylinositol (PI)	1.02	1.01	0.63	1.13	1.00	1.00	0.96	1.11	0.36	1.45	0.89	1.00	1.07	1.06	0.70	0.87	0.99	1.00
1-palmitoyl-2-dihomo-linolenoyl-GPC (16:0/2	Lipid	Phosphatidylcholine (PC)	1.00	1.00	0.94	1.32	1.00	1.00	1.02	1.02	0.98	0.89	1.00	1.00	1.02	1.04	0.79	1.10	0.99	1.00
1-palmitoyl-2-docosahexaenoyl-GPC (16:0/22:	Lipid	Phosphatidylcholine (PC)	0.97	1.02	0.19	1.70	1.00	1.00	0.93	0.99	0.08	1.49	0.73	1.00	0.97	1.01	0.45	1.17	0.99	1.00
1-palmitoyl-2-docosahexaenoyl-GPE (16:0/22:(	Lipid	Phosphatidylethanolamine (PI	1.03	0.98	0.18	0.90	1.00	1.00	0.96	1.00	0.73	0.87	1.00	1.00	0.99	1.04	0.80	0.89	0.99	1.00
1-palmitoyl-2-linoleoyl-GPC (16:0/18:2)	Lipid	Phosphatidylcholine (PC)	0.98	0.99	0.83	1.43	1.00	1.00	0.98	1.01	0.41	1.24	0.89	1.00	1.01	1.02	0.83	1.18	0.99	1.00
1-palmitoyl-2-linoleoyl-GPE (16:0/18:2)	Lipid	Phosphatidylethanolamine (PI	1.06	0.81	0.02	1.98	1.00	1.00	1.04	0.94	0.24	1.05	0.84	1.00	1.05	0.93	0.36	1.30	0.99	1.00
1-palmitoyl-2-linoleoyl-GPI (16:0/18:2)	Lipid	Phosphatidylinositol (PI)	1.03	0.95	0.64	0.83	1.00	1.00	1.02	1.14	0.27	0.80	0.85	1.00	1.10	1.06	0.74	0.73	0.99	1.00
1-palmitoyl-2-oleoyl-GPC (16:0/18:1)	Lipid	Phosphatidylcholine (PC)	1.02	0.97	0.47	1.41	1.00	1.00	1.00	1.00	0.99	0.89	1.00	1.00	1.03	1.00	0.30	1.18	0.99	1.00
1-palmitoyl-2-oleoyl-GPI (16:0/18:1)*	Lipid	Phosphatidylinositol (PI)	0.99	0.92	0.31	1.24	1.00	1.00	0.95	1.06	0.45	1.26	0.92	1.00	1.03	1.03	0.90	1.02	0.99	1.00
1-palmitoyl-2-palmitoleoyl-GPC (16:0/16:1)*	Lipid	Phosphatidylcholine (PC)	1.08	0.97	0.19	1.34	1.00	1.00	1.06	1.04	0.73	0.52	1.00	1.00	1.11	1.04	0.45	0.98	0.99	1.00
1-palmitoyl-2-stearoyl-GPC (16:0/18:0)	Lipid	Phosphatidylcholine (PC)	0.95	0.98	0.66	1.63	1.00	1.00	0.92	0.98	0.07	1.68	0.73	1.00	1.01	1.00	0.83	1.06	0.99	1.00
1-palmitoyl-GPC (16:0)	Lipid	Lysophospholipid	0.99	0.98	0.67	1.30	1.00	1.00	0.99	1.01	0.99	1.04	1.00	1.00	1.05	1.02	0.25	0.95	0.99	1.00
1-palmitoyl-GPE (16:0)	Lipid	Lysophospholipid	1.01	0.94	0.19	1.18	1.00	1.00	0.98	0.97	0.79	0.49	1.00	1.00	1.06	1.00	0.44	0.97	0.99	1.00
1-palmitoyl-GPI* (16:0)	Lipid	Lysophospholipid	1.01	1.09	0.84	0.36	1.00	1.00	1.20	1.31	0.68	0.38	1.00	1.00	1.08	1.11	0.67	0.19	0.99	1.00
1-ribosyl-imidazoleacetate*	Amino Acid	Histidine Metabolism	1.28	1.10	0.14	1.21	1.00	1.00	1.33	1.07	0.00	1.45	0.73	1.00	1.46	1.16	0.02	1.25	0.86	1.00
1-stearoyl-2-arachidonoyl-GPC (18:0/20:4)	Lipid	Phosphatidylcholine (PC)	0.97	0.97	0.96	1.26	1.00	1.00	0.97	0.99	0.48	0.93	0.94	1.00	1.00	1.00	0.66	0.98	0.99	1.00
1-stearoyl-2-arachidonoyl-GPE (18:0/20:4)	Lipid	Phosphatidylethanolamine (PI	1.00	0.89	0.09	1.56	1.00	1.00	0.98	0.97	0.81	0.67	1.00	1.00	0.99	1.00	0.92	0.87	0.99	1.00
1-stearoyl-2-arachidonoyl-GPI (18:0/20:4)	Lipid	Phosphatidylinositol (PI)	0.99	0.97	0.72	1.13	1.00	1.00	0.94	1.02	0.23	1.19	0.84	1.00	1.00	0.98	0.53	0.75	0.99	1.00
1-stearoyl-2-docosahexaenoyl-GPC (18:0/22:6)	Lipid	Phosphatidylcholine (PC)	0.98	1.05	0.23	1.60	1.00	1.00	0.92	1.01	0.04	1.59	0.73	1.00	0.98	1.02	0.27	0.90	0.99	1.00
1-stearoyl-2-docosahexaenoyl-GPE (18:0/22:6)'	Lipid	Phosphatidylethanolamine (PI	0.99	0.93	0.34	0.96	1.00	1.00	0.95	0.97	0.76	0.78	1.00	1.00	0.95	0.97	0.79	0.67	0.99	1.00
1-stearoyl-2-linoleoyl-GPC (18:0/18:2)*	Lipid	Phosphatidylcholine (PC)	0.96	0.96	0.92	1.38	1.00	1.00	0.98	0.99	0.65	0.96	1.00	1.00	0.99	1.01	0.90	1.18	0.99	1.00
1-stearoyl-2-linoleoyl-GPE (18:0/18:2)*	Lipid	Phosphatidylethanolamine (PI	0.99	0.78	0.04	1.79	1.00	1.00	0.99	0.93	0.39	1.03	0.89	1.00	1.02	0.91	0.44	1.36	0.99	1.00
1-stearoyl-2-linoleoyl-GPI (18:0/18:2)	Lipid	Phosphatidylinositol (PI)	0.95	0.95	0.95	1.05	1.00	1.00	0.89	1.07	0.03	1.99	0.73	1.00	0.98	1.03	0.72	0.96	0.99	1.00
1-stearoyl-2-oleoyl-GPC (18:0/18:1)	Lipid	Phosphatidylcholine (PC)	1.02	0.95	0.22	1.53	1.00	1.00	1.00	1.00	0.87	1.02	1.00	1.00	1.05	1.01	0.53	1.22	0.99	1.00
1-stearoyl-2-oleoyl-GPI (18:0/18:1)*	Lipid	Phosphatidylinositol (PI)	0.95	0.99	0.73	1.26	1.00	1.00	0.95	1.12	0.13	1.36	0.76	1.00	1.04	1.10	0.67	1.15	0.99	1.00
1-stearoyl-GPC (18:0)	Lipid	Lysophospholipid	1.00	0.99	0.48	1.34	1.00	1.00	0.99	1.02	0.71	1.04	1.00	1.00	1.03	1.03	0.81	1.14	0.99	1.00
1-stearoyl-GPE (18:0)	Lipid	Lysophospholipid	1.00	0.94	0.26	1.22	1.00	1.00	0.97	1.00	0.74	0.70	1.00	1.00	1.05	1.03	0.95	0.85	0.99	1.00
1-stearoyl-GPI (18:0)	Lipid	Lysophospholipid	1.18	1.21	0.75	1.01	1.00	1.00	1.11	1.23	0.22	0.85	0.83	1.00	1.20	1.19	0.89	0.93	0.99	1.00
2,3-dihydroxy-5-methylthio-4-pentenoate (DMT	Amino Acid	Methionine, Cysteine, SAM an	1.02	0.97	0.33	1.30	1.00	1.00	1.05	0.97	0.13	1.73	0.76	1.00	1.04	1.01	0.18	1.18	0.99	1.00
2-aminobutyrate	Amino Acid	Glutathione Metabolism	1.07	1.03	0.68	0.68	1.00	1.00	1.05	1.00	0.27	0.78	0.85	1.00	1.05	1.05	0.94	0.63	0.99	1.00
2-aminooctanoate	Lipid	Fatty Acid, Amino	1.10	1.02	0.26	0.45	1.00	1.00	1.08	1.11	0.84	0.24	1.00	1.00	1.13	1.18	0.79	0.38	0.99	1.00
2-aminophenol sulfate	Xenobiotics	Chemical	1.05	1.44	0.13	1.35	1.00	1.00	0.98	1.25	0.38	1.00	0.89	1.00	1.13	1.60	0.49	1.24	0.99	1.00
2-hydroxy-3-methylvalerate	Amino Acid	Leucine, Isoleucine and Valine	1.02	0.95	0.84	1.10	1.00	1.00	1.06	0.99	0.89	1.27	1.00	1.00	1.06	1.01	0.77	0.80	0.99	1.00
2-hydroxybutyrate/2-hydroxyisobutyrate	Amino Acid	Glutathione Metabolism	0.97	0.99	1.00	0.24	1.00	1.00	0.92	0.93	0.66	0.18	1.00	1.00	0.91	0.95	0.69	0.61	0.99	1.00
2-hydroxydecanoate	Lipid	Fatty Acid, Monohydroxy	0.92	0.83	0.26	0.92	1.00	1.00	0.95	0.80	0.08	1.31	0.73	1.00	1.05	0.78	0.03	2.06	0.89	1.00
2-hydroxyglutarate	Lipid	Fatty Acid, Dicarboxylate	1.07	1.05	0.75	1.14	1.00	1.00	1.08	1.01	0.25	1.06	0.85	1.00	1.12	1.08	0.77	0.83	0.99	1.00
2-hydroxynervonate*	Lipid	Fatty Acid, Monohydroxy	0.84	1.04	0.05	1.75	1.00	1.00	0.87	0.96	0.33	0.96	0.88	1.00	0.88	0.99	0.15	1.39	0.99	1.00
2-hydroxyoctanoate	Lipid	Fatty Acid, Monohydroxy	1.14	0.89	0.61	0.79	1.00	1.00	0.85	0.97	0.55	1.36	0.97	1.00	0.97	1.30	0.63	0.93	0.99	1.00
2-hydroxypalmitate	Lipid	Fatty Acid, Monohydroxy	0.92	1.07	0.23	1.32	1.00	1.00	0.96	1.00	0.91	0.51	1.00	1.00	0.96	1.03	0.33	1.20	0.99	1.00
2-hydroxystearate	Lipid	Fatty Acid, Monohydroxy	0.87	1.01	0.30	1.18	1.00	1.00	0.93	1.02	0.37	0.97	0.89	1.00	0.91	1.04	0.05	1.67	0.99	1.00
2-methylcitrate/homocitrate	Energy	TCA Cycle	1.01	0.99	0.53	0.71	1.00	1.00	1.03	0.98	0.41	1.03	0.89	1.00	1.04	1.01	0.55	0.66	0.99	1.00
2-oleoylglycerol (18:1)	Lipid	Monoacylglycerol	1.09	1.05	0.42	0.37	1.00	1.00	1.14	1.10	0.36	0.42	0.89	1.00	1.12	1.09	0.91	0.17	0.99	1.00
2-palmitoyl-GPC* (16:0)*	Lipid	Lysophospholipid	0.97	1.11	0.86	0.64	1.00	1.00	1.12	1.11	0.34	0.40	0.88	1.00	1.01	1.19	0.68	0.84	0.99	1.00
2-piperidinone	Xenobiotics	Food Component/Plant	1.11	0.91	0.02	1.94	1.00	1.00	1.06	1.01	0.33	0.64	0.88	1.00	1.13	1.04	0.30	0.94	0.99	1.00
2-pyrrolidinone	Amino Acid	Glutamate Metabolism	1.03	1.03	0.79	0.82	1.00	1.00	1.01	1.02	0.83	0.72	1.00	1.00	1.12	1.23	0.36	1.35	0.99	1.00
2-stearoyl-GPE (18:0)*	Lipid	Lysophospholipid	0.90	0.94	0.96	0.28	1.00	1.00	0.94	0.93	0.80	0.28	1.00	1.00	0.96	1.02	0.44	0.58	0.99	1.00
3-(4-hydroxyphenyl)lactate (HPLA)	Amino Acid	Tyrosine Metabolism	0.96	0.92	0.43	1.00	1.00	1.00	1.00	0.94	0.32	1.24	0.88	1.00	1.07	0.97	0.11	1.55	0.99	1.00

3,4-dihydroxybutyrate	Amino Acid	Glutamate Metabolism	0.95	0.93	0.60	0.38	1.00	1.00	1.02	0.88	0.10	1.27	0.75	1.00	0.94	0.92	0.85	0.22	0.99	1.00
3-aminoisobutyrate	Nucleotide	Pyrimidine Metabolism, Thym	1.14	1.09	0.64	0.57	1.00	1.00	1.08	1.04	0.51	0.44	0.95	1.00	1.12	1.06	0.86	0.66	0.99	1.00
3beta,7alpha-dihydroxy-5-cholestenoate	Lipid	Sterol	1.25	1.03	0.05	1.63	1.00	1.00	1.19	0.99	0.07	1.69	0.73	1.00	1.20	1.05	0.39	1.35	0.99	1.00
3beta-hydroxy-5-cholestenoate	Lipid	Sterol	0.94	1.04	0.61	1.13	1.00	1.00	1.00	0.97	0.41	0.52	0.89	1.00	0.94	1.02	0.25	1.12	0.99	1.00
3-carboxy-4-methyl-5-pentyl-2-furanpropionate	Lipid	Fatty Acid, Dicarboxylate	1.05	1.06	0.83	0.50	1.00	1.00	1.06	1.02	0.68	0.68	1.00	1.00	1.12	1.02	0.22	1.02	0.99	1.00
3-carboxy-4-methyl-5-propyl-2-furanpropanoate	Lipid	Fatty Acid, Dicarboxylate	1.05	0.96	0.53	0.75	1.00	1.00	0.98	0.95	0.86	0.49	1.00	1.00	0.90	0.96	0.73	0.64	0.99	1.00
3-formylindole	Xenobiotics	Food Component/Plant	1.08	0.97	0.15	1.14	1.00	1.00	1.06	1.00	0.50	1.09	0.95	1.00	1.09	0.99	0.13	1.23	0.99	1.00
3-hydroxy-2-ethylpropionate	Amino Acid	Leucine, Isoleucine and Valine	1.01	0.98	0.60	0.47	1.00	1.00	0.98	0.98	0.79	0.64	1.00	1.00	1.03	0.95	0.14	1.38	0.99	1.00
3-hydroxy-3-methylglutarate	Lipid	Mevalonate Metabolism	0.97	0.96	0.64	0.57	1.00	1.00	1.04	0.97	0.36	0.78	0.89	1.00	1.01	0.99	0.80	0.32	0.99	1.00
3-hydroxybutyrate (BHBA)	Lipid	Ketone Bodies	1.56	1.97	0.91	0.80	1.00	1.00	1.36	1.83	0.77	0.92	1.00	1.00	1.08	1.22	0.45	0.62	0.99	1.00
3-hydroxydecanoate	Lipid	Fatty Acid, Monohydroxy	1.11	1.18	0.61	0.62	1.00	1.00	1.11	1.16	0.71	0.48	1.00	1.00	1.05	1.10	0.85	0.37	0.99	1.00
3-hydroxyhexanoate	Lipid	Fatty Acid, Monohydroxy	1.00	1.02	0.81	0.19	1.00	1.00	0.95	0.98	0.92	0.43	1.00	1.00	0.93	0.93	0.90	0.24	0.99	1.00
3-hydroxyhippurate	Xenobiotics	Benzoate Metabolism	2.09	1.90	0.41	0.27	1.00	1.00	1.22	1.48	0.37	0.67	0.89	1.00	1.31	2.67	0.40	1.38	0.99	1.00
3-hydroxylaurate	Lipid	Fatty Acid, Monohydroxy	1.38	1.56	0.45	0.82	1.00	1.00	1.44	1.56	0.48	0.67	0.94	1.00	1.36	1.35	0.56	0.53	0.99	1.00
3-hydroxyoctanoate	Lipid	Fatty Acid, Monohydroxy	0.99	1.06	0.29	0.69	1.00	1.00	0.96	1.02	0.48	0.55	0.94	1.00	0.96	0.98	0.90	0.21	0.99	1.00
3-hydroxyoleate*	Lipid	Fatty Acid, Monohydroxy	1.12	1.27	0.92	0.98	1.00	1.00	1.10	1.22	0.96	0.82	1.00	1.00	1.06	1.19	0.36	1.11	0.99	1.00
3-hydroxypyridine sulfate	Xenobiotics	Chemical	0.80	0.66	0.20	0.78	1.00	1.00	1.63	0.71	0.05	1.49	0.73	1.00	1.16	1.41	0.08	0.54	0.99	1.00
3-indoxyl sulfate	Amino Acid	Tryptophan Metabolism	1.04	0.98	0.91	1.01	1.00	1.00	1.14	1.09	0.67	0.98	1.00	1.00	1.22	1.05	0.20	1.42	0.99	1.00
3-methyl-2-oxobutyrate	Amino Acid	Leucine, Isoleucine and Valine	1.02	0.97	0.32	0.88	1.00	1.00	1.02	1.01	0.76	0.22	1.00	1.00	1.03	1.02	0.60	0.20	0.99	1.00
3-methyl-2-oxovalerate	Amino Acid	Leucine, Isoleucine and Valine	0.93	0.93	0.93	1.17	1.00	1.00	0.94	0.92	0.75	0.88	1.00	1.00	0.97	0.99	0.80	0.98	0.99	1.00
3-methylglutaconate	Amino Acid	Leucine, Isoleucine and Valine	1.02	1.00	0.42	0.86	1.00	1.00	1.06	0.99	0.28	1.16	0.85	1.00	1.09	1.03	0.23	1.01	0.99	1.00
3-methylhistidine	Amino Acid	Histidine Metabolism	2.76	2.25	0.60	0.35	1.00	1.00	2.50	2.77	0.17	0.21	0.79	1.00	6.98	2.83	0.50	0.85	0.99	1.00
3-ureidopropionate	Nucleotide	Pyrimidine Metabolism, Uracil	1.03	1.09	0.93	1.01	1.00	1.00	1.02	1.00	0.85	0.96	1.00	1.00	0.97	1.12	0.17	1.93	0.99	1.00
4-acetamidobutanoate	Amino Acid	Polyamine Metabolism	1.16	0.99	0.04	1.60	1.00	1.00	1.16	1.00	0.05	1.60	0.73	1.00	1.19	0.99	0.02	2.15	0.86	1.00
4-allylphenol sulfate	Xenobiotics	Food Component/Plant	1.66	0.92	0.01	1.97	1.00	1.00	2.73	1.74	0.38	1.00	0.89	1.00	1.60	1.14	0.69	1.11	0.99	1.00
4-ethylphenyl sulfate	Xenobiotics	Benzoate Metabolism	1.16	0.88	0.17	1.23	1.00	1.00	1.42	0.91	0.01	1.98	0.73	1.00	1.24	1.17	0.02	0.33	0.86	1.00
4-hydroxychlorothalonil	Xenobiotics	Chemical	0.93	0.95	0.89	0.46	1.00	1.00	1.10	0.97	0.06	1.39	0.73	1.00	1.09	0.94	0.47	1.50	0.99	1.00
4-hydroxyphenylpyruvate	Amino Acid	Tyrosine Metabolism	0.93	0.88	0.36	0.94	1.00	1.00	1.00	0.94	0.79	1.20	1.00	1.00	0.98	0.92	0.47	1.19	0.99	1.00
4-methyl-2-oxopentanoate	Amino Acid	Leucine, Isoleucine and Valine	0.98	0.96	0.64	0.94	1.00	1.00	0.95	0.98	0.73	0.95	1.00	1.00	0.97	1.01	0.51	1.08	0.99	1.00
4-methylcatechol sulfate	Xenobiotics	Benzoate Metabolism	1.03	1.26	0.17	1.41	1.00	1.00	1.02	1.27	0.36	1.40	0.89	1.00	0.94	1.37	0.08	2.13	0.99	1.00
4-vinylphenol sulfate	Xenobiotics	Benzoate Metabolism	2.32	0.93	0.41	1.13	1.00	1.00	1.04	1.14	0.70	0.32	1.00	1.00	1.35	1.45	0.96	0.27	0.99	1.00
5,6-dihydrouridine	Nucleotide	Pyrimidine Metabolism, Uracil	1.05	1.03	0.38	0.79	1.00	1.00	1.05	1.04	0.66	0.98	1.00	1.00	1.05	1.07	0.66	0.91	0.99	1.00
5-acetylamino-6-amino-3-methyluracil	Xenobiotics	Xanthine Metabolism	0.89	1.12	0.11	1.80	1.00	1.00	1.17	1.06	0.65	0.72	1.00	1.00	1.29	1.18	0.79	0.59	0.99	1.00
5-dodecenoate (12:1n7)	Lipid	Medium Chain Fatty Acid	1.55	1.49	0.75	0.69	1.00	1.00	1.38	1.57	0.66	0.76	1.00	1.00	1.45	1.35	0.83	0.73	0.99	1.00
5-dodecenoylcarnitine (C12:1)	Lipid	Fatty Acid Metabolism(Acyl Ca	1.53	1.50	0.86	0.61	1.00	1.00	1.48	1.56	0.59	0.57	0.98	1.00	1.29	1.39	0.89	0.61	0.99	1.00
5-hydroxylysine	Amino Acid	Lysine Metabolism	1.07	1.06	0.88	1.02	1.00	1.00	1.01	1.01	0.77	1.13	1.00	1.00	1.19	1.06	0.88	0.99	0.99	1.00
5-methylthioadenosine (MTA)	Amino Acid	Polyamine Metabolism	1.11	1.02	0.54	1.17	1.00	1.00	1.12	1.01	0.09	1.32	0.75	1.00	1.13	1.04	0.27	1.14	0.99	1.00
5-methylthioribose	Amino Acid	Methionine, Cysteine, SAM an	1.10	1.03	0.53	1.09	1.00	1.00	1.04	1.01	0.72	0.85	1.00	1.00	1.07	1.05	0.98	0.69	0.99	1.00
5-methyluridine (ribothymidine)	Nucleotide	Pyrimidine Metabolism, Uracil	0.97	1.03	0.67	1.17	1.00	1.00	1.01	1.01	0.90	0.63	1.00	1.00	1.03	1.05	0.92	0.52	0.99	1.00
5-oxoproline	Amino Acid	Glutathione Metabolism	1.02	1.01	0.99	1.28	1.00	1.00	1.07	0.97	0.19	1.68	0.80	1.00	1.06	1.04	0.55	0.97	0.99	1.00
6-bromotryptophan	Amino Acid	Tryptophan Metabolism	0.97	0.97	0.75	1.01	1.00	1.00	0.98	0.97	0.76	0.86	1.00	1.00	1.06	1.02	0.82	0.71	0.99	1.00
6-hydroxyindole sulfate	Xenobiotics	Chemical	1.02	1.03	0.48	1.00	1.00	1.00	1.10	1.14	0.40	1.03	0.89	1.00	1.20	1.07	0.51	1.14	0.99	1.00
6-oxopiperidine-2-carboxylate	Amino Acid	Lysine Metabolism	1.07	0.86	0.03	1.67	1.00	1.00	1.20	0.88	0.03	1.87	0.73	1.00	1.06	0.89	0.05	1.82	0.99	1.00
7-HOCA	Lipid	Sterol	1.05	0.98	0.10	1.02	1.00	1.00	1.03	0.97	0.17	0.81	0.79	1.00	1.02	1.02	0.54	0.11	0.99	1.00
7-methylguanine	Nucleotide	Purine Metabolism, Guanine c	1.06	1.00	0.45	1.04	1.00	1.00	1.09	1.01	0.21	1.24	0.81	1.00	1.09	1.05	0.44	0.82	0.99	1.00
9-hydroxystearate	Lipid	Fatty Acid, Monohydroxy	1.09	1.07	0.60	0.75	1.00	1.00	1.16	1.17	0.44	0.57	0.91	1.00	1.21	1.01	0.10	1.65	0.99	1.00
acetylcarnitine (C2)	Lipid	Fatty Acid Metabolism(Acyl Ca	1.14	1.01	0.14	1.29	1.00	1.00	1.11	1.01	0.13	1.08	0.76	1.00	1.02	0.96	0.32	0.72	0.99	1.00
acisoga	Amino Acid	Polyamine Metabolism	1.20	1.24	0.90	0.61	1.00	1.00	1.11	1.11	0.70	0.26	1.00	1.00	1.13	1.22	0.63	0.90	0.99	1.00
aconitate [cis or trans]	Energy	TCA Cycle	0.95	0.96	0.94	0.85	1.00	1.00	0.96	0.98	0.99	0.92	1.00	1.00	0.99	0.98	0.53	0.62	0.99	1.00
adrenate (22:4n6)	Lipid	Polyunsaturated Fatty Acid (n3	1.25	1.54	0.76	1.30	1.00	1.00	1.37	1.43	0.82	1.05	1.00	1.00	1.21	1.27	0.42	1.03	0.99	1.00
alanine	Amino Acid	Alanine and Aspartate Metabol	1.04	0.99	0.57	1.13	1.00	1.00	1.12	0.99	0.08	1.55	0.73	1.00	1.09	1.05	0.66	0.82	0.99	1.00
allantoin	Nucleotide	Purine Metabolism, (Hypo)Xar	0.90	1.02	0.25	1.53	1.00	1.00	1.15	0.91	0.08	1.67	0.73	1.00	0.98	1.01	0.71	0.83	0.99	1.00
alpha-hydroxyisocaproate	Amino Acid	Leucine, Isoleucine and Valine	0.93	1.02	0.61	1.32	1.00	1.00	0.93	0.98	0.53	1.25	0.96	1.00	1.00	1.06	0.40	0.97	0.99	1.00
alpha-hydroxyisovalerate	Amino Acid	Leucine, Isoleucine and Valine	1.05	1.08	0.94	0.85	1.00	1.00	1.05	1.04	0.74	0.55	1.00	1.00	1.01	1.06	0.81	0.82	0.99	1.00

alpha-ketoglutaramate*	Amino Acid	Glutamate Metabolism	0.96	0.97	0.60	0.54	1.00	1.00	1.07	0.92	0.12	1.51	0.76	1.00	0.96	0.97	0.71	0.13	0.99	1.00
alpha-ketoglutarate	Energy	TCA Cycle	1.14	1.08	0.94	0.53	1.00	1.00	1.17	1.06	0.93	0.48	1.00	1.00	1.20	1.06	0.81	0.67	0.99	1.00
alpha-tocopherol	Cofactors ar	Tocopherol Metabolism	1.03	1.01	0.76	0.75	1.00	1.00	1.03	0.97	0.64	0.80	1.00	1.00	0.99	1.01	0.76	0.45	0.99	1.00
androstenediol (3beta,17beta) disulfate (1)	Lipid	Androgenic Steroids	1.08	1.04	0.79	0.71	1.00	1.00	1.09	1.00	0.45	0.99	0.92	1.00	1.10	1.09	0.69	0.74	0.99	1.00
androstenediol (3beta,17beta) disulfate (2)	Lipid	Androgenic Steroids	1.03	1.09	0.92	0.67	1.00	1.00	1.00	1.00	0.82	0.68	1.00	1.00	1.07	1.06	0.88	0.40	0.99	1.00
androstenediol (3beta,17beta) monosulfate (1)	Lipid	Androgenic Steroids	1.13	0.97	0.10	1.58	1.00	1.00	1.10	1.04	0.53	0.61	0.96	1.00	1.04	1.00	0.61	0.46	0.99	1.00
androsterone glucuronide	Lipid	Androgenic Steroids	1.04	1.11	0.92	0.96	1.00	1.00	1.04	1.09	0.90	0.82	1.00	1.00	1.06	1.12	0.88	0.90	0.99	1.00
androsterone sulfate	Lipid	Androgenic Steroids	1.10	1.14	0.95	0.33	1.00	1.00	1.07	1.25	0.86	0.68	1.00	1.00	1.03	1.10	0.50	0.69	0.99	1.00
arabitol/xylitol	Carbohydrat	Pentose Metabolism	1.08	0.96	0.16	1.11	1.00	1.00	1.08	0.95	0.24	1.13	0.84	1.00	1.10	1.01	0.57	0.88	0.99	1.00
arabonate/xylonate	Carbohydrat	Pentose Metabolism	0.91	0.98	0.43	0.70	1.00	1.00	1.00	0.99	0.93	0.46	1.00	1.00	0.90	1.02	0.14	1.14	0.99	1.00
arachidate (20:0)	Lipid	Long Chain Fatty Acid	0.90	1.11	0.12	1.52	1.00	1.00	0.98	0.95	0.96	0.24	1.00	1.00	0.88	0.95	0.53	0.87	0.99	1.00
arachidonate (20:4n6)	Lipid	Polyunsaturated Fatty Acid (n3	0.97	1.03	0.53	0.94	1.00	1.00	1.05	1.04	0.87	0.52	1.00	1.00	1.08	1.05	0.99	0.93	1.00	1.00
arachidonoylcarnitine (C20:4)	Lipid	Fatty Acid Metabolism(Acyl Ca	1.04	1.04	0.62	0.63	1.00	1.00	1.11	1.10	0.87	0.29	1.00	1.00	1.10	1.12	0.78	0.67	0.99	1.00
arachidonoylcholine	Lipid	Fatty Acid Metabolism (Acyl Cl	1.14	1.15	0.27	0.50	1.00	1.00	1.13	1.35	0.48	1.09	0.94	1.00	1.23	1.23	0.44	0.61	0.99	1.00
argininate*	Amino Acid	Urea cycle; Arginine and Prolin	1.03	0.94	0.21	0.93	1.00	1.00	1.21	1.03	0.18	1.21	0.79	1.00	1.24	1.13	0.32	0.94	0.99	1.00
arginine	Amino Acid	Urea cycle; Arginine and Prolin	1.03	1.03	0.86	1.23	1.00	1.00	1.03	1.00	0.54	1.17	0.97	1.00	1.05	1.04	0.93	1.05	0.99	1.00
asparagine	Amino Acid	Alanine and Aspartate Metabol	1.02	1.01	0.88	1.37	1.00	1.00	1.05	0.99	0.42	1.50	0.90	1.00	1.07	1.05	0.76	1.18	0.99	1.00
aspartate	Amino Acid	Alanine and Aspartate Metabol	0.96	1.04	0.19	1.64	1.00	1.00	1.08	1.00	0.36	0.98	0.89	1.00	1.01	1.02	0.91	0.65	0.99	1.00
azelate (nonanedioate; C9)	Lipid	Fatty Acid, Dicarboxylate	0.89	0.88	0.77	0.67	1.00	1.00	0.97	0.87	0.41	0.94	0.89	1.00	1.30	0.90	0.36	1.02	0.99	1.00
behenoyl dihydrosphingomyelin (d18:0/22:0)	Lipid	Dihydrosphingomyelins	1.14	1.00	0.36	1.05	1.00	1.00	0.99	1.00	0.32	0.35	0.88	1.00	1.15	1.01	0.46	1.29	0.99	1.00
behenoyl sphingomyelin (d18:1/22:0)*	Lipid	Sphingomyelins	1.03	1.00	0.55	1.08	1.00	1.00	0.96	0.99	0.48	0.75	0.94	1.00	1.04	0.99	0.46	1.17	0.99	1.00
beta-alanine	Nucleotide	Pyrimidine Metabolism, Uracil	0.99	1.08	0.49	1.37	1.00	1.00	1.03	0.91	0.08	1.34	0.73	1.00	1.09	1.11	0.90	0.84	0.99	1.00
beta-citrylglutamate	Amino Acid	Glutamate Metabolism	1.03	1.10	0.38	1.23	1.00	1.00	1.10	1.01	0.29	1.14	0.87	1.00	1.08	1.03	0.63	0.77	0.99	1.00
beta-cryptoxanthin	Cofactors ar	Vitamin A Metabolism	1.18	1.17	0.84	0.27	1.00	1.00	1.15	1.46	0.38	0.91	0.89	1.00	1.12	1.25	0.76	0.52	0.99	1.00
beta-hydroxyisovalerate	Amino Acid	Leucine, Isoleucine and Valine	0.99	1.03	0.85	0.87	1.00	1.00	1.02	1.06	0.83	1.15	1.00	1.00	1.03	1.08	0.94	0.88	0.99	1.00
betaine	Amino Acid	Glycine, Serine and Threonine	0.99	1.02	0.64	1.32	1.00	1.00	0.99	0.98	0.90	1.00	1.00	1.00	0.98	1.01	0.45	1.17	0.99	1.00
bilirubin (E,E)*	Cofactors ar	Hemoglobin and Porphyrin M	1.22	1.33	0.60	0.70	1.00	1.00	1.05	1.29	0.02	1.71	0.73	1.00	1.08	1.20	0.22	1.10	0.99	1.00
bilirubin (E,Z or Z,E)*	Cofactors ar	Hemoglobin and Porphyrin M	1.11	1.06	0.83	0.38	1.00	1.00	1.03	1.16	0.03	1.02	0.73	1.00	1.08	1.15	0.39	0.75	0.99	1.00
bilirubin	Cofactors ar	Hemoglobin and Porphyrin M	1.08	1.03	0.70	0.61	1.00	1.00	1.01	1.08	0.24	1.13	0.85	1.00	1.06	1.08	0.80	0.34	0.99	1.00
biliverdin	Cofactors ar	Hemoglobin and Porphyrin M	1.36	1.28	0.52	0.61	1.00	1.00	1.11	1.23	0.41	0.85	0.89	1.00	1.15	1.19	0.61	0.47	0.99	1.00
butyrylcarnitine (C4)	Lipid	Fatty Acid Metabolism (also B(	1.22	1.01	0.00	2.19	0.47	0.94	1.22	0.98	0.00	2.58	0.26	0.26	1.21	0.99	0.00	2.43	0.86	1.00
caffeine	Xenobiotics	Xanthine Metabolism	0.81	0.78	0.44	0.62	1.00	1.00	1.07	0.77	0.08	1.24	0.73	1.00	1.33	0.96	0.81	0.76	0.99	1.00
caprate (10:0)	Lipid	Medium Chain Fatty Acid	1.03	0.91	0.15	0.96	1.00	1.00	1.14	0.97	0.20	1.00	0.81	1.00	1.08	0.84	0.02	2.02	0.86	1.00
carnitine	Lipid	Carnitine Metabolism	1.03	0.97	0.44	1.30	1.00	1.00	1.05	0.98	0.25	1.46	0.85	1.00	1.03	0.97	0.19	1.44	0.99	1.00
carotene diol (1)	Cofactors ar	Vitamin A Metabolism	0.96	0.90	0.30	0.73	1.00	1.00	0.97	1.00	0.45	0.60	0.92	1.00	1.06	0.95	0.17	1.33	0.99	1.00
carotene diol (2)	Cofactors ar	Vitamin A Metabolism	1.04	0.92	0.13	1.20	1.00	1.00	1.06	0.98	1.00	0.84	1.00	1.00	1.11	1.02	0.36	0.87	0.99	1.00
catechol sulfate	Xenobiotics	Benzoate Metabolism	1.15	0.89	0.17	1.37	1.00	1.00	0.99	0.86	0.48	0.89	0.94	1.00	1.04	1.73	0.67	0.86	0.99	1.00
ceramide (d16:1/24:1, d18:1/22:1)*	Lipid	Ceramides	1.06	1.00	0.30	0.96	1.00	1.00	1.04	1.00	0.70	0.66	1.00	1.00	1.03	1.05	0.84	0.74	0.99	1.00
ceramide (d18:1/14:0, d16:1/16:0)*	Lipid	Ceramides	1.06	0.99	0.24	1.03	1.00	1.00	1.09	1.05	0.90	0.63	1.00	1.00	1.03	1.02	0.21	0.96	0.99	1.00
ceramide (d18:1/20:0, d16:1/22:0, d20:1/18	Lipid	Ceramides	1.14	1.02	0.25	1.26	1.00	1.00	1.06	0.95	0.36	1.18	0.89	1.00	1.06	1.02	0.71	1.03	0.99	1.00
ceramide (d18:2/24:1, d18:1/24:2)*	Lipid	Ceramides	1.09	1.02	0.26	1.10	1.00	1.00	1.05	1.01	0.71	0.67	1.00	1.00	1.02	1.05	0.73	0.81	0.99	1.00
cerotoylcarnitine (C26)*	Lipid	Fatty Acid Metabolism(Acyl Ca	0.99	0.96	0.38	0.94	1.00	1.00	0.90	0.98	0.26	1.44	0.85	1.00	1.00	0.97	0.96	0.80	0.99	1.00
C-glycosyltryptophan	Amino Acid	Tryptophan Metabolism	1.01	1.06	0.94	1.03	1.00	1.00	1.04	1.01	0.41	0.65	0.89	1.00	1.06	1.07	0.62	0.61	0.99	1.00
cholate	Lipid	Primary Bile Acid Metabolism	2.98	1.33	0.28	1.51	1.00	1.00	2.82	1.37	0.13	1.81	0.76	1.00	2.31	2.23	0.36	0.13	0.99	1.00
cholesterol	Lipid	Sterol	1.03	0.98	0.50	1.20	1.00	1.00	1.01	0.99	0.80	0.91	1.00	1.00	1.02	0.95	0.11	1.59	0.99	1.00
choline	Lipid	Phospholipid Metabolism	1.02	1.02	0.81	1.19	1.00	1.00	1.02	1.02	0.81	1.21	1.00	1.00	1.03	1.06	0.68	1.17	0.99	1.00
cinnamoylglycine	Xenobiotics	Food Component/Plant	1.08	1.50	0.08	1.35	1.00	1.00	0.93	1.30	0.17	1.38	0.79	1.00	1.23	1.47	0.47	1.01	0.99	1.00
cis-4-decenoate (10:1n6)*	Lipid	Medium Chain Fatty Acid	1.41	1.33	0.95	0.88	1.00	1.00	1.27	1.42	0.48	0.77	0.94	1.00	1.29	1.28	0.69	0.61	0.99	1.00
cis-4-decenoylcarnitine (C10:1)	Lipid	Fatty Acid Metabolism(Acyl Ca	1.25	1.22	0.84	0.41	1.00	1.00	1.16	1.29	0.76	0.56	1.00	1.00	1.17	1.17	0.72	0.24	0.99	1.00
citraconate/glutaconate	Energy	TCA Cycle	0.80	0.63	0.15	1.14	1.00	1.00	1.39	0.63	0.01	1.61	0.73	1.00	0.99	0.90	0.08	0.40	0.99	1.00
citrate	Energy	TCA Cycle	0.96	1.04	0.37	1.29	1.00	1.00	0.92	0.99	0.13	1.21	0.76	1.00	0.98	1.02	0.68	0.58	0.99	1.00
citrulline	Amino Acid	Urea cycle; Arginine and Prolin	1.09	1.02	0.29	1.11	1.00	1.00	1.11	1.01	0.11	1.27	0.76	1.00	1.21	1.06	0.27	1.36	0.99	1.00
cortisol	Lipid	Corticosteroids	1.14	1.27	0.50	0.85	1.00	1.00	1.13	1.24	0.68	0.66	1.00	1.00	1.35	1.55	0.48	1.11	0.99	1.00
creatine	Amino Acid	Creatine Metabolism	0.99	0.96	0.53	0.96	1.00	1.00	1.04	1.02	0.91	1.13	1.00	1.00	1.06	0.99	0.60	1.21	0.99	1.00

creatinine	Amino Acid	Creatine Metabolism	1.02	0.98	0.61	1.37	1.00	1.00	1.02	0.98	0.31	1.44	0.88	1.00	1.01	1.02	0.97	1.31	0.99	1.00
cys-gly, oxidized	Amino Acid	Glutathione Metabolism	1.08	1.08	0.88	0.90	1.00	1.00	1.10	1.04	0.27	0.85	0.85	1.00	1.17	1.16	0.64	0.45	0.99	1.00
cysteine	Amino Acid	Methionine, Cysteine, SAM an	1.32	1.03	0.36	1.41	1.00	1.00	1.28	1.06	0.35	1.34	0.89	1.00	1.33	1.08	0.09	1.76	0.99	1.00
cysteine-glutathione disulfide	Amino Acid	Glutathione Metabolism	1.13	1.13	0.76	0.72	1.00	1.00	1.07	1.17	0.65	0.81	1.00	1.00	1.22	1.32	0.56	0.87	0.99	1.00
cysteinylglycine disulfide*	Amino Acid	Glutathione Metabolism	1.03	1.01	0.71	1.09	1.00	1.00	1.03	0.99	0.21	1.03	0.81	1.00	1.07	1.08	0.75	0.85	0.99	1.00
cystine	Amino Acid	Methionine, Cysteine, SAM an	1.14	1.16	0.66	0.65	1.00	1.00	1.02	1.18	0.72	1.30	1.00	1.00	1.07	1.18	0.63	1.02	0.99	1.00
decanoylcarnitine (C10)	Lipid	Fatty Acid Metabolism(Acyl Ca	1.18	1.44	0.38	1.10	1.00	1.00	1.14	1.34	0.53	0.71	0.96	1.00	1.16	1.25	0.43	0.46	0.99	1.00
dehydroepiandrosterone sulfate (DHEA-S)	Lipid	Androgenic Steroids	1.02	1.03	0.82	0.38	1.00	1.00	1.02	1.04	0.97	0.57	1.00	1.00	1.02	1.01	0.86	0.29	0.99	1.00
deoxycarnitine	Lipid	Carnitine Metabolism	1.02	0.98	0.71	1.11	1.00	1.00	1.11	0.98	0.05	1.81	0.73	1.00	1.08	1.04	0.59	1.19	0.99	1.00
diacylglycerol (12:0/18:1, 14:0/16:1, 16:0/14	Lipid	Diacylglycerol	1.12	0.85	0.22	1.27	1.00	1.00	1.27	1.00	0.48	1.09	0.94	1.00	1.14	1.17	0.79	1.03	0.99	1.00
diacylglycerol (14:0/18:1, 16:0/16:1) [1]*	Lipid	Diacylglycerol	1.13	0.89	0.15	1.40	1.00	1.00	1.07	0.95	0.38	0.86	0.89	1.00	1.06	0.99	0.70	0.91	0.99	1.00
diacylglycerol (14:0/18:1, 16:0/16:1) [2]*	Lipid	Diacylglycerol	1.09	0.88	0.14	1.40	1.00	1.00	1.06	0.98	0.50	0.66	0.95	1.00	1.09	1.02	0.85	0.80	0.99	1.00
diacylglycerol (16:1/18:2 [2], 16:0/18:3 [1])*	Lipid	Diacylglycerol	1.02	0.99	0.67	0.91	1.00	1.00	0.95	1.00	0.71	0.54	1.00	1.00	0.97	1.03	0.43	0.73	0.99	1.00
dihomolinoleate (20:2n6)	Lipid	Polyunsaturated Fatty Acid (n3	1.17	1.44	0.84	1.31	1.00	1.00	1.16	1.25	0.92	1.06	1.00	1.00	1.10	1.15	0.67	1.17	0.99	1.00
dihomolinolenate (20:3n3 or 3n6)	Lipid	Polyunsaturated Fatty Acid (n3	1.03	1.02	0.96	0.93	1.00	1.00	1.11	1.08	0.82	0.58	1.00	1.00	1.08	1.05	0.97	1.00	0.99	1.00
dihomo-linolenoyl-choline	Lipid	Fatty Acid Metabolism (Acyl Cl	1.25	1.27	0.16	0.39	1.00	1.00	1.26	1.49	0.92	0.87	1.00	1.00	1.37	1.28	0.40	0.62	0.99	1.00
dihydroorotate	Nucleotide	Pyrimidine Metabolism, Orota	1.07	1.00	0.92	0.50	1.00	1.00	1.07	0.99	0.80	0.71	1.00	1.00	1.09	1.02	0.89	0.67	0.99	1.00
dimethylarginine (ADMA + SDMA)	Amino Acid	Urea cycle; Arginine and Prolin	1.07	1.04	0.72	1.20	1.00	1.00	1.08	1.02	0.23	1.21	0.84	1.00	1.09	1.08	0.84	0.92	0.99	1.00
dimethylglycine	Amino Acid	Glycine, Serine and Threonine	1.01	1.02	0.99	1.14	1.00	1.00	1.01	0.95	0.41	1.23	0.89	1.00	1.04	1.00	0.44	0.94	0.99	1.00
docosadienoate (22:2n6)	Lipid	Polyunsaturated Fatty Acid (n3	1.07	1.32	0.41	1.52	1.00	1.00	1.09	1.27	0.17	1.34	0.79	1.00	1.03	1.11	0.69	1.16	0.99	1.00
docosahexaenoate (DHA; 22:6n3)	Lipid	Polyunsaturated Fatty Acid (n3	0.98	1.16	0.24	1.44	1.00	1.00	1.00	1.11	0.26	1.06	0.85	1.00	1.01	1.08	0.46	1.15	0.99	1.00
docosahexaenoylcarnitine (C22:6)*	Lipid	Fatty Acid Metabolism(Acyl Ca	1.18	1.16	0.66	0.18	1.00	1.00	1.12	1.18	0.67	0.35	1.00	1.00	1.16	1.15	0.87	0.10	0.99	1.00
docosahexaenoylcholine	Lipid	Fatty Acid Metabolism (Acyl Cl	1.11	1.12	0.28	0.59	1.00	1.00	1.06	1.29	0.33	1.27	0.88	1.00	1.20	1.21	0.69	0.73	0.99	1.00
docosapentaenoate (DPA; 22:5n3)	Lipid	Polyunsaturated Fatty Acid (n3	1.16	1.36	0.62	1.37	1.00	1.00	1.10	1.23	0.32	1.14	0.88	1.00	1.08	1.15	0.61	1.18	0.99	1.00
dodecadienoate (12:2)*	Lipid	Fatty Acid, Dicarboxylate	1.19	1.19	0.88	0.63	1.00	1.00	1.12	1.20	0.89	0.78	1.00	1.00	1.13	1.16	0.90	0.57	0.99	1.00
dodecanedioate (C12)	Lipid	Fatty Acid, Dicarboxylate	0.92	0.97	0.93	0.44	1.00	1.00	0.89	1.02	0.96	0.75	1.00	1.00	0.93	0.96	0.91	0.56	0.99	1.00
dodecenedioate (C12:1-DC)*	Lipid	Fatty Acid, Dicarboxylate	1.00	1.04	0.98	0.29	1.00	1.00	0.96	1.01	0.50	0.61	0.95	1.00	0.96	0.95	0.68	0.11	0.99	1.00
dopamine 3-O-sulfate	Amino Acid	Tyrosine Metabolism	0.99	0.92	0.07	0.65	1.00	1.00	1.10	0.93	0.03	0.76	0.73	1.00	1.12	1.05	0.10	0.20	0.99	1.00
eicosanedioate (C20-DC)	Lipid	Fatty Acid, Dicarboxylate	0.97	0.94	0.51	0.44	1.00	1.00	1.22	0.94	0.23	1.49	0.84	1.00	1.19	1.11	0.90	0.54	0.99	1.00
eicosapentaenoate (EPA; 20:5n3)	Lipid	Polyunsaturated Fatty Acid (n3	1.00	1.08	0.88	0.86	1.00	1.00	1.02	1.04	0.98	0.49	1.00	1.00	1.10	1.02	0.39	1.13	0.99	1.00
eicosenoate (20:1n9 or 1n11)	Lipid	Long Chain Fatty Acid	1.22	1.58	0.52	1.42	1.00	1.00	1.15	1.45	0.24	1.44	0.84	1.00	1.11	1.21	0.34	1.16	0.99	1.00
ergothioneine	Xenobiotics	Food Component/Plant	1.05	1.00	0.55	1.16	1.00	1.00	1.00	0.95	0.32	0.97	0.88	1.00	1.00	0.98	0.82	0.99	0.99	1.00
erucate (22:1n9)	Lipid	Long Chain Fatty Acid	1.05	1.28	0.10	1.10	1.00	1.00	1.03	1.15	0.48	0.71	0.94	1.00	0.90	1.17	0.39	1.42	0.99	1.00
erythritol	Xenobiotics	Food Component/Plant	1.04	1.01	0.51	0.66	1.00	1.00	1.05	1.00	0.59	0.68	0.98	1.00	0.97	1.13	0.17	1.50	0.99	1.00
erythronate*	Carbohydrat	Aminosugar Metabolism	0.96	0.96	0.97	0.87	1.00	1.00	1.02	0.96	0.29	1.18	0.87	1.00	0.97	1.01	0.51	0.87	0.99	1.00
ethylmalonate	Amino Acid	Leucine, Isoleucine and Valine	1.10	1.03	0.11	0.80	1.00	1.00	1.07	0.98	0.08	1.36	0.73	1.00	1.03	1.01	0.63	0.45	0.99	1.00
etiocholanolone glucuronide	Lipid	Androgenic Steroids	1.08	1.23	0.55	1.24	1.00	1.00	1.10	1.19	0.88	0.69	1.00	1.00	1.16	1.24	0.84	0.71	0.99	1.00
Fibrinopeptide A (2-15)	Peptide	Fibrinogen Cleavage Peptide	0.94	1.03	0.17	1.48	1.00	1.00	1.00	0.95	0.41	0.93	0.89	1.00	1.00	1.02	0.78	0.64	0.99	1.00
Fibrinopeptide A (3-15)	Peptide	Fibrinogen Cleavage Peptide	0.90	1.05	0.02	2.32	1.00	1.00	1.01	1.00	0.69	0.43	1.00	1.00	0.99	1.04	0.90	0.77	0.99	1.00
Fibrinopeptide A (3-16)	Peptide	Fibrinogen Cleavage Peptide	1.13	1.16	0.36	0.18	1.00	1.00	1.11	1.11	0.35	0.02	0.89	1.00	1.21	1.28	0.46	0.42	0.99	1.00
Fibrinopeptide A (4-15)	Peptide	Fibrinogen Cleavage Peptide	0.92	1.06	0.29	1.20	1.00	1.00	1.07	1.07	0.88	0.26	1.00	1.00	1.01	1.09	0.60	0.87	0.99	1.00
Fibrinopeptide A (5-16)*	Peptide	Fibrinogen Cleavage Peptide	1.06	1.17	0.73	0.50	1.00	1.00	1.10	1.13	0.34	0.14	0.88	1.00	1.13	1.28	0.58	0.62	0.99	1.00
Fibrinopeptide A (7-16)*	Peptide	Fibrinogen Cleavage Peptide	0.97	1.25	0.04	1.37	1.00	1.00	1.02	1.20	0.76	0.64	1.00	1.00	1.03	1.27	0.48	1.06	0.99	1.00
Fibrinopeptide A (8-16)	Peptide	Fibrinogen Cleavage Peptide	1.16	1.60	0.03	0.96	1.00	1.00	1.24	1.51	0.55	0.60	0.97	1.00	1.22	1.56	0.41	0.84	0.99	1.00
DSGEGDFXAEGGGVR*	Peptide	Fibrinogen Cleavage Peptide	1.12	1.18	0.42	0.28	1.00	1.00	1.10	1.11	0.62	0.05	1.00	1.00	1.20	1.30	0.65	0.53	0.99	1.00
Fibrinopeptide B (1-12)	Peptide	Fibrinogen Cleavage Peptide	0.98	1.02	0.58	1.29	1.00	1.00	0.98	0.95	0.69	0.98	1.00	1.00	1.02	1.03	0.97	0.56	0.99	1.00
Fibrinopeptide B (1-13)	Peptide	Fibrinogen Cleavage Peptide	1.06	1.08	0.98	0.40	1.00	1.00	1.07	1.02	0.36	0.44	0.89	1.00	1.11	1.11	0.43	0.40	0.99	1.00
fructose	Carbohydrat	Fructose, Mannose and Galacto	1.00	0.98	0.88	0.45	1.00	1.00	1.13	1.05	0.40	0.91	0.89	1.00	0.99	1.04	0.86	0.65	0.99	1.00
gamma-CEHC	Cofactors ar	Tocopherol Metabolism	0.92	1.00	0.38	1.07	1.00	1.00	1.01	1.12	0.38	1.15	0.89	1.00	1.19	1.24	0.79	0.56	0.99	1.00
gamma-glutamylalanine	Peptide	Gamma-glutamyl Amino Acid	1.05	1.32	0.34	1.18	1.00	1.00	1.08	1.24	0.20	0.78	0.81	1.00	1.14	1.32	0.52	0.82	0.99	1.00
gamma-glutamyl-alpha-lysine	Peptide	Gamma-glutamyl Amino Acid	1.10	1.01	0.30	1.36	1.00	1.00	1.12	0.99	0.04	1.91	0.73	1.00	1.13	1.08	0.30	1.19	0.99	1.00
gamma-glutamylcitrulline*	Peptide	Gamma-glutamyl Amino Acid	1.09	1.01	0.47	0.99	1.00	1.00	1.14	1.01	0.20	1.23	0.81	1.00	1.27	1.11	0.42	1.02	0.99	1.00
gamma-glutamyl-epsilon-lysine	Peptide	Gamma-glutamyl Amino Acid	0.99	1.00	0.82	0.92	1.00	1.00	0.99	1.04	0.92	0.91	1.00	1.00	1.06	1.06	0.57	0.56	0.99	1.00
gamma-glutamylglutamine	Peptide	Gamma-glutamyl Amino Acid	1.03	1.02	0.85	1.22	1.00	1.00	1.07	0.99	0.17	1.38	0.79	1.00	1.06	1.08	0.90	0.84	0.99	1.00

gamma-glutamylglycine	Peptide	Gamma-glutamyl Amino Acid	1.11	1.04	0.38	1.09	1.00	1.00	1.15	1.02	0.16	1.36	0.79	1.00	1.17	1.06	0.12	1.54	0.99	1.00
gamma-glutamylhistidine	Peptide	Gamma-glutamyl Amino Acid	1.01	1.00	0.76	0.81	1.00	1.00	0.95	1.10	0.13	1.66	0.76	1.00	1.11	1.13	0.60	0.77	0.99	1.00
gamma-glutamylisoleucine*	Peptide	Gamma-glutamyl Amino Acid	1.08	0.95	0.26	1.26	1.00	1.00	1.12	0.96	0.07	1.71	0.73	1.00	1.13	1.04	0.39	1.29	0.99	1.00
gamma-glutamylleucine	Peptide	Gamma-glutamyl Amino Acid	1.00	0.88	0.04	1.21	1.00	1.00	1.08	0.90	0.02	1.68	0.73	1.00	1.04	0.97	0.22	1.09	0.99	1.00
gamma-glutamylmethionine	Peptide	Gamma-glutamyl Amino Acid	1.04	1.03	0.99	1.21	1.00	1.00	1.15	1.00	0.26	1.59	0.85	1.00	1.15	1.05	0.14	1.26	0.99	1.00
gamma-glutamylthreonine	Peptide	Gamma-glutamyl Amino Acid	1.14	1.05	0.40	1.11	1.00	1.00	1.18	1.00	0.05	1.63	0.73	1.00	1.24	1.09	0.06	1.56	0.99	1.00
gamma-glutamylvaline	Peptide	Gamma-glutamyl Amino Acid	1.11	1.00	0.13	1.17	1.00	1.00	1.15	1.02	0.06	1.44	0.73	1.00	1.10	1.09	0.55	0.95	0.99	1.00
gamma-tocopherol/beta-tocopherol	Cofactors ar	Tocopherol Metabolism	1.13	1.13	0.79	0.82	1.00	1.00	1.19	1.10	0.24	0.70	0.85	1.00	1.08	1.14	0.97	0.71	0.99	1.00
gluconate	Xenobiotics	Food Component/Plant	1.09	1.01	0.71	0.59	1.00	1.00	1.17	0.99	0.11	0.95	0.76	1.00	1.08	1.04	0.98	0.29	0.99	1.00
glucose	Carbohydrat	Glycolysis, Gluconeogenesis, a	1.01	1.03	0.85	0.90	1.00	1.00	1.04	1.01	0.18	0.61	0.79	1.00	1.02	1.06	0.43	1.14	0.99	1.00
glucuronate	Carbohydrat	Aminosugar Metabolism	0.98	0.97	0.56	0.34	1.00	1.00	1.01	1.05	0.50	0.91	0.95	1.00	0.98	0.93	0.31	1.16	0.99	1.00
glu-gly-asn-val	Peptide	Polypeptide	0.93	1.30	0.22	1.30	1.00	1.00	1.30	1.36	0.44	0.55	0.91	1.00	1.13	1.29	0.67	0.53	0.99	1.00
glutamate	Amino Acid	Glutamate Metabolism	1.12	1.13	0.68	0.98	1.00	1.00	1.29	1.16	0.54	1.11	0.97	1.00	1.12	1.10	0.99	0.72	1.00	1.00
glutamine	Amino Acid	Glutamate Metabolism	1.02	1.02	0.99	1.33	1.00	1.00	1.06	0.99	0.07	1.66	0.73	1.00	1.06	1.05	0.67	0.91	0.99	1.00
glycerate	Carbohydrat	Glycolysis, Gluconeogenesis, a	0.97	0.98	0.68	0.82	1.00	1.00	1.01	0.93	0.32	1.19	0.88	1.00	0.95	0.99	0.58	0.51	0.99	1.00
glycerol	Lipid	Glycerolipid Metabolism	1.32	1.11	0.19	1.73	1.00	1.00	1.16	1.12	0.58	0.78	0.98	1.00	1.20	0.99	0.03	2.15	0.86	1.00
glycerophosphorylcholine (GPC)	Lipid	Phospholipid Metabolism	1.24	1.12	0.93	0.56	1.00	1.00	1.09	1.09	0.76	1.04	1.00	1.00	1.20	1.27	0.83	0.89	0.99	1.00
glycine	Amino Acid	Glycine, Serine and Threonine	1.03	1.05	0.97	1.26	1.00	1.00	1.08	1.02	0.13	1.19	0.77	1.00	1.10	1.04	0.26	1.33	0.99	1.00
glycochenodeoxycholate	Lipid	Primary Bile Acid Metabolism	0.91	0.85	0.84	0.31	1.00	1.00	1.54	1.22	0.66	1.07	1.00	1.00	1.21	1.02	0.89	0.76	0.99	1.00
glycocholate	Lipid	Primary Bile Acid Metabolism	1.10	1.08	0.38	0.16	1.00	1.00	1.31	1.38	0.97	1.02	1.00	1.00	1.30	1.10	0.70	0.78	0.99	1.00
glycocholenate sulfate*	Lipid	Secondary Bile Acid Metabolis	1.10	1.18	0.81	0.79	1.00	1.00	1.09	1.08	0.71	0.83	1.00	1.00	1.15	1.16	0.99	0.82	1.00	1.00
glycolithocholate sulfate*	Lipid	Secondary Bile Acid Metabolis	1.02	1.15	0.32	0.90	1.00	1.00	0.86	1.13	0.67	1.11	1.00	1.00	1.09	1.01	0.66	0.63	0.99	1.00
glycosyl ceramide (d18:2/24:1, d18:1/24:2)*	Lipid	Hexosylceramides (HCER)	1.03	1.02	0.57	0.67	1.00	1.00	0.97	1.01	0.38	0.80	0.89	1.00	1.05	0.98	0.16	1.07	0.99	1.00
glycosyl-N-palmitoyl-sphingosine (d18:1/16:0	Lipid	Hexosylceramides (HCER)	1.01	1.02	0.86	0.60	1.00	1.00	0.98	0.97	0.86	0.71	1.00	1.00	1.00	0.97	0.83	0.79	0.99	1.00
glycosyl-N-stearoyl-sphingosine (d18:1/18:0)	Lipid	Hexosylceramides (HCER)	1.13	1.02	0.15	1.17	1.00	1.00	1.05	0.97	0.93	0.75	1.00	1.00	1.14	1.00	0.06	1.42	0.99	1.00
guaiacol sulfate	Xenobiotics	Benzoate Metabolism	1.22	0.96	0.30	1.28	1.00	1.00	1.03	0.93	0.72	0.74	1.00	1.00	1.12	1.55	0.58	0.79	0.99	1.00
guanidinoacetate	Amino Acid	Creatine Metabolism	1.13	1.12	0.85	0.39	1.00	1.00	1.15	1.00	0.11	1.23	0.76	1.00	1.19	1.10	0.59	0.77	0.99	1.00
guanosine	Nucleotide	Purine Metabolism, Guanine c	1.54	1.62	0.58	0.16	1.00	1.00	1.27	1.42	0.45	0.52	0.92	1.00	1.39	1.56	0.88	0.71	0.99	1.00
heptenedioate (C7:1-DC)*	Lipid	Fatty Acid, Dicarboxylate	1.19	1.00	0.12	1.29	1.00	1.00	1.11	1.00	0.27	0.87	0.85	1.00	1.12	0.88	0.02	2.11	0.86	1.00
hexadecadienoate (16:2n6)	Lipid	Polyunsaturated Fatty Acid (n3	1.32	1.38	0.69	0.88	1.00	1.00	1.19	1.22	0.97	0.88	1.00	1.00	1.19	1.16	0.61	0.91	0.99	1.00
hexadecanedioate (C16)	Lipid	Fatty Acid, Dicarboxylate	1.09	1.02	0.21	0.72	1.00	1.00	0.98	1.06	0.85	0.62	1.00	1.00	1.08	0.95	0.08	1.14	0.99	1.00
hexadecenedioate (C16:1-DC)*	Lipid	Fatty Acid, Dicarboxylate	0.97	0.94	0.49	0.33	1.00	1.00	0.98	0.92	0.36	0.56	0.89	1.00	1.08	0.92	0.16	1.49	0.99	1.00
hexanoylcarnitine (C6)	Lipid	Fatty Acid Metabolism(Acyl Ca	1.27	1.16	0.39	0.89	1.00	1.00	1.29	1.12	0.09	1.26	0.75	1.00	1.27	1.11	0.19	1.27	0.99	1.00
hexanoylglutamine	Lipid	Fatty Acid Metabolism (Acyl Gl	1.28	1.41	0.96	0.55	1.00	1.00	1.19	1.21	0.74	0.48	1.00	1.00	1.19	1.22	0.86	0.41	0.99	1.00
hippurate	Xenobiotics	Benzoate Metabolism	1.05	1.18	0.87	1.03	1.00	1.00	0.94	1.18	0.65	0.90	1.00	1.00	1.18	1.49	0.93	0.81	0.99	1.00
histidine	Amino Acid	Histidine Metabolism	0.98	0.98	0.87	1.24	1.00	1.00	1.01	0.99	0.86	1.38	1.00	1.00	1.04	1.02	0.59	0.97	0.99	1.00
homoarginine	Amino Acid	Urea cycle; Arginine and Prolin	1.05	1.06	0.82	0.91	1.00	1.00	1.06	1.07	0.67	0.83	1.00	1.00	1.09	1.12	0.76	0.72	0.99	1.00
homostachydrine*	Xenobiotics	Food Component/Plant	1.02	1.13	0.15	0.83	1.00	1.00	1.00	1.00	0.83	0.12	1.00	1.00	1.25	1.13	0.91	0.64	0.99	1.00
hydroxyasparagine	Amino Acid	Alanine and Aspartate Metabol	1.13	1.03	0.14	1.49	1.00	1.00	1.10	1.01	0.15	1.49	0.78	1.00	1.12	1.05	0.15	1.45	0.99	1.00
hydroxy-CMPF*	Lipid	Fatty Acid, Dicarboxylate	0.96	0.99	0.70	0.91	1.00	1.00	0.92	0.95	0.86	1.01	1.00	1.00	0.88	0.98	0.27	1.40	0.99	1.00
hydroxy-N6,N6,N6-trimethyllysine*	Amino Acid	Lysine Metabolism	1.12	1.08	0.62	0.71	1.00	1.00	1.24	1.08	0.10	1.06	0.75	1.00	1.23	1.13	0.35	0.94	0.99	1.00
hydroxypalmitoyl sphingomyelin (d18:1/16:0(	Lipid	Sphingomyelins	1.00	1.02	0.78	1.29	1.00	1.00	0.95	1.02	0.03	1.66	0.73	1.00	1.01	1.02	0.92	1.00	0.99	1.00
hypotaurine	Amino Acid	Methionine, Cysteine, SAM an	0.98	1.03	0.47	1.18	1.00	1.00	1.09	0.97	0.11	1.44	0.76	1.00	1.13	1.05	0.08	1.18	0.99	1.00
hypoxanthine	Nucleotide	Purine Metabolism, (Hypo)Xar	1.03	1.15	0.30	1.13	1.00	1.00	1.06	1.09	0.92	0.19	1.00	1.00	1.03	1.14	0.20	1.11	0.99	1.00
imidazole lactate	Amino Acid	Histidine Metabolism	1.07	1.03	0.60	1.13	1.00	1.00	1.08	1.01	0.33	1.24	0.88	1.00	1.10	1.10	0.71	1.05	0.99	1.00
indoleacetate	Amino Acid	Tryptophan Metabolism	0.99	0.90	0.77	0.77	1.00	1.00	1.11	1.02	0.87	0.66	1.00	1.00	1.09	1.02	0.63	0.86	0.99	1.00
indolelactate	Amino Acid	Tryptophan Metabolism	0.98	0.95	0.32	0.93	1.00	1.00	1.03	0.99	0.47	0.90	0.94	1.00	1.05	1.07	0.68	0.74	0.99	1.00
indolepropionate	Amino Acid	Tryptophan Metabolism	1.01	1.28	0.19	1.37	1.00	1.00	0.90	1.13	0.64	0.87	1.00	1.00	1.03	1.58	0.01	2.37	0.86	1.00
inosine	Nucleotide	Purine Metabolism, (Hypo)Xar	1.31	1.39	0.86	0.28	1.00	1.00	1.11	1.40	0.25	1.15	0.85	1.00	1.20	1.38	0.65	0.86	0.99	1.00
isobutyrylcarnitine (C4)	Amino Acid	Leucine, Isoleucine and Valine	1.03	0.84	0.11	1.70	1.00	1.00	1.14	0.93	0.21	1.45	0.83	1.00	1.17	0.97	0.29	1.48	0.99	1.00
isoleucine	Amino Acid	Leucine, Isoleucine and Valine	0.99	0.96	0.83	1.39	1.00	1.00	1.02	0.95	0.33	1.58	0.88	1.00	1.04	0.99	0.45	1.37	0.99	1.00
isovalerylcarnitine (C5)	Amino Acid	Leucine, Isoleucine and Valine	1.17	0.88	0.02	2.05	1.00	1.00	1.21	1.08	0.19	0.98	0.80	1.00	1.10	0.93	0.13	1.72	0.99	1.00
kynurenate	Amino Acid	Tryptophan Metabolism	1.01	0.96	0.32	0.64	1.00	1.00	1.03	1.00	0.96	0.81	1.00	1.00	1.04	1.03	0.98	0.43	0.99	1.00
kynurenine	Amino Acid	Tryptophan Metabolism	1.06	0.94	0.14	1.40	1.00	1.00	1.07	0.98	0.17	1.31	0.79	1.00	1.08	1.05	0.44	0.87	0.99	1.00

lactate	Carbohydrat	Glycolysis, Gluconeogenesis, a	0.95	1.06	0.14	1.91	1.00	1.00	1.04	1.02	0.93	0.91	1.00	1.00	1.03	1.06	0.60	0.82	0.99	1.00
lactosyl-N-nervonoyl-sphingosine (d18:1/24:1	Lipid	Lactosylceramides (LCER)	1.09	1.05	0.83	0.87	1.00	1.00	0.97	0.99	0.27	0.38	0.85	1.00	1.05	1.00	0.76	1.01	0.99	1.00
lactosyl-N-palmitoyl-sphingosine (d18:1/16:0)	Lipid	Lactosylceramides (LCER)	1.01	1.04	0.54	1.23	1.00	1.00	0.93	1.04	0.04	1.77	0.73	1.00	1.01	1.04	0.54	1.04	0.99	1.00
laurate (12:0)	Lipid	Medium Chain Fatty Acid	1.16	1.13	0.57	0.75	1.00	1.00	1.10	1.20	0.83	0.75	1.00	1.00	1.12	1.08	0.95	0.31	0.99	1.00
laurylcarnitine (C12)	Lipid	Fatty Acid Metabolism(Acyl Ca	1.32	1.37	0.97	0.41	1.00	1.00	1.31	1.41	0.99	0.48	1.00	1.00	1.27	1.19	0.56	0.64	0.99	1.00
leucine	Amino Acid	Leucine, Isoleucine and Valine	1.00	0.98	0.82	1.36	1.00	1.00	1.02	0.99	0.81	1.52	1.00	1.00	1.02	1.00	0.87	1.24	0.99	1.00
lignoceroyl sphingomyelin (d18:1/24:0)	Lipid	Sphingomyelins	1.11	1.01	0.19	0.99	1.00	1.00	0.97	0.97	0.54	0.21	0.97	1.00	1.11	0.97	0.13	1.69	0.99	1.00
lignoceroylcarnitine (C24)*	Lipid	Fatty Acid Metabolism(Acyl Ca	1.07	0.95	0.18	1.21	1.00	1.00	0.95	1.02	0.75	0.87	1.00	1.00	1.05	0.99	0.14	0.98	0.99	1.00
linoleate (18:2n6)	Lipid	Polyunsaturated Fatty Acid (n3	1.12	1.21	0.94	1.32	1.00	1.00	1.09	1.16	0.86	1.21	1.00	1.00	1.04	1.07	0.81	1.21	0.99	1.00
linolenate (18:3n3 or 3n6)	Lipid	Polyunsaturated Fatty Acid (n3	1.20	1.39	0.87	1.15	1.00	1.00	1.15	1.33	0.53	1.02	0.96	1.00	1.05	1.14	0.76	0.96	0.99	1.00
linolenoylcarnitine (C18:3)*	Lipid	Fatty Acid Metabolism(Acyl Ca	1.06	1.15	0.22	1.08	1.00	1.00	1.05	1.25	0.02	1.37	0.73	1.00	1.07	1.16	0.37	1.04	0.99	1.00
linoleoyl-arachidonoyl-glycerol (18:2/20:4) [2]	Lipid	Diacylglycerol	0.94	0.93	0.87	0.46	1.00	1.00	0.82	0.98	0.19	1.17	0.80	1.00	0.91	0.99	0.76	0.72	0.99	1.00
linoleoylcarnitine (C18:2)*	Lipid	Fatty Acid Metabolism(Acyl Ca	1.03	1.07	0.36	0.82	1.00	1.00	1.01	1.13	0.04	1.43	0.73	1.00	1.05	1.09	0.38	0.75	0.99	1.00
linoleoylcholine*	Lipid	Fatty Acid Metabolism (Acyl Cl	1.15	1.07	0.30	0.60	1.00	1.00	1.17	1.33	0.75	0.88	1.00	1.00	1.29	1.19	0.41	0.71	0.99	1.00
linoleoyl-linoleoyl-glycerol (18:2/18:2) [1]*	Lipid	Diacylglycerol	1.11	1.02	0.71	0.73	1.00	1.00	1.02	1.06	0.86	0.27	1.00	1.00	1.03	1.02	0.87	0.21	0.99	1.00
lysine	Amino Acid	Lysine Metabolism	1.05	1.02	0.97	1.32	1.00	1.00	1.07	1.02	0.25	1.39	0.85	1.00	1.08	1.07	0.55	1.11	0.99	1.00
malate	Energy	TCA Cycle	1.00	1.02	0.99	0.84	1.00	1.00	0.99	0.99	0.76	0.82	1.00	1.00	1.04	1.06	0.70	0.77	0.99	1.00
mannitol/sorbitol	Carbohydrat	Fructose, Mannose and Galact	1.75	0.97	0.10	1.09	1.00	1.00	3.52	1.36	0.01	1.47	0.73	1.00	1.87	1.69	0.06	0.38	0.99	1.00
mannonate*	Xenobiotics	Food Component/Plant	1.01	1.02	0.87	0.08	1.00	1.00	1.08	1.01	0.24	0.85	0.85	1.00	1.02	1.05	0.73	0.50	0.99	1.00
mannose	Carbohydrat	Fructose, Mannose and Galact	1.06	1.02	0.21	0.64	1.00	1.00	1.09	1.00	0.06	1.35	0.73	1.00	1.06	1.10	0.87	0.69	0.99	1.00
margarate (17:0)	Lipid	Long Chain Fatty Acid	1.10	1.33	0.81	1.34	1.00	1.00	1.11	1.16	0.77	1.02	1.00	1.00	1.06	1.08	0.75	1.14	0.99	1.00
margaroylcarnitine (C17)*	Lipid	Fatty Acid Metabolism(Acyl Ca	1.05	1.01	0.21	0.71	1.00	1.00	0.99	1.02	0.61	0.41	1.00	1.00	1.07	0.99	0.17	1.11	0.99	1.00
methionine	Amino Acid	Methionine, Cysteine, SAM an	1.03	0.99	0.97	1.29	1.00	1.00	1.06	1.00	0.41	1.57	0.89	1.00	1.09	1.04	0.41	1.18	0.99	1.00
methionine sulfone	Amino Acid	Methionine, Cysteine, SAM an	1.00	0.98	0.88	1.24	1.00	1.00	1.03	1.03	0.79	1.10	1.00	1.00	1.02	1.07	0.47	1.34	0.99	1.00
methionine sulfoxide	Amino Acid	Methionine, Cysteine, SAM an	0.88	0.91	0.82	1.50	1.00	1.00	1.01	0.93	0.68	1.45	1.00	1.00	0.95	0.94	0.74	1.05	0.99	1.00
methyl glucopyranoside (alpha + beta)	Xenobiotics	Food Component/Plant	1.70	1.02	0.10	1.73	1.00	1.00	0.91	1.12	0.41	0.92	0.89	1.00	1.18	1.20	0.58	0.12	0.99	1.00
methylsuccinate	Amino Acid	Leucine, Isoleucine and Valine	0.93	0.92	0.97	0.26	1.00	1.00	1.06	0.92	0.60	1.11	0.99	1.00	1.03	0.97	0.39	0.60	0.99	1.00
myo-inositol	Lipid	Inositol Metabolism	1.20	1.02	0.62	0.76	1.00	1.00	1.17	1.02	0.69	0.78	1.00	1.00	1.30	1.12	0.82	1.02	0.99	1.00
myristate (14:0)	Lipid	Long Chain Fatty Acid	1.30	1.41	0.60	1.09	1.00	1.00	1.21	1.29	0.71	0.99	1.00	1.00	1.17	1.11	0.70	1.13	0.99	1.00
myristoleate (14:1n5)	Lipid	Long Chain Fatty Acid	1.64	1.45	0.60	0.99	1.00	1.00	1.38	1.51	0.84	0.79	1.00	1.00	1.48	1.28	0.46	0.99	0.99	1.00
myristoleoylcarnitine (C14:1)*	Lipid	Fatty Acid Metabolism(Acyl Ca	1.36	1.46	0.65	0.57	1.00	1.00	1.21	1.54	0.28	1.19	0.85	1.00	1.24	1.28	0.99	0.42	1.00	1.00
myristoyl dihydrosphingomyelin (d18:0/14:0)	Lipid	Dihydrosphingomyelins	0.98	0.99	0.99	0.92	1.00	1.00	0.96	1.08	0.05	1.94	0.73	1.00	1.00	1.06	0.46	1.33	0.99	1.00
myristoylcarnitine (C14)	Lipid	Fatty Acid Metabolism(Acyl Ca	1.14	1.12	0.74	0.22	1.00	1.00	1.09	1.16	0.52	0.64	0.96	1.00	1.09	1.05	0.42	0.50	0.99	1.00
N,N,N-trimethyl-5-aminovalerate	Amino Acid	Lysine Metabolism	1.02	1.00	0.76	1.20	1.00	1.00	1.10	1.01	0.14	1.37	0.78	1.00	1.10	1.02	0.32	1.25	0.99	1.00
N,N,N-trimethyl-alanylproline betaine (TMAP)	Amino Acid	Urea cycle; Arginine and Prolin	1.14	1.00	0.05	1.88	1.00	1.00	1.09	0.99	0.17	1.48	0.79	1.00	1.11	1.03	0.13	1.21	0.99	1.00
N1-Methyl-2-pyridone-5-carboxamide	Cofactors ar	Nicotinate and Nicotinamide I	0.93	1.03	0.53	0.72	1.00	1.00	1.01	1.00	0.94	0.26	1.00	1.00	0.87	1.10	0.04	2.18	0.99	1.00
1-methyladenosine	Nucleotide	Purine Metabolism, Adenine c	1.09	1.01	0.36	1.23	1.00	1.00	1.09	0.99	0.07	1.42	0.73	1.00	1.07	1.09	0.94	1.00	0.99	1.00
N1-methylinosine	Nucleotide	Purine Metabolism, (Hypo)Xar	1.19	1.15	0.73	0.50	1.00	1.00	1.20	1.15	0.70	0.68	1.00	1.00	1.16	1.26	0.73	0.99	0.99	1.00
N2,N2-dimethylguanosine	Nucleotide	Purine Metabolism, Guanine c	1.10	1.12	0.87	0.79	1.00	1.00	1.13	1.08	0.18	0.62	0.79	1.00	1.11	1.23	0.68	1.08	0.99	1.00
N6,N6,N6-trimethyllysine	Amino Acid	Lysine Metabolism	1.12	1.07	0.17	0.72	1.00	1.00	1.42	1.03	0.06	1.80	0.73	1.00	1.48	1.10	0.45	1.63	0.99	1.00
N6,N6-dimethyllysine	Amino Acid	Lysine Metabolism	1.07	1.01	0.38	0.98	1.00	1.00	1.15	0.98	0.05	1.72	0.73	1.00	1.18	1.05	0.29	1.40	0.99	1.00
N6-acetyllysine	Amino Acid	Lysine Metabolism	1.02	0.96	0.30	1.34	1.00	1.00	1.04	0.96	0.26	1.60	0.85	1.00	1.06	1.00	0.37	1.30	0.99	1.00
N6-carbamoylthreonyladenosine	Nucleotide	Purine Metabolism, Adenine c	1.04	1.02	0.70	0.94	1.00	1.00	1.09	1.06	0.85	0.87	1.00	1.00	1.05	1.06	0.87	0.65	0.99	1.00
N6-methyllysine	Amino Acid	Lysine Metabolism	1.17	1.01	0.17	1.01	1.00	1.00	1.23	1.00	0.09	1.39	0.75	1.00	1.14	1.07	0.93	0.76	0.99	1.00
N-acetyl-2-aminooctanoate*	Lipid	Fatty Acid, Amino	1.09	1.12	0.71	0.33	1.00	1.00	1.06	1.13	0.84	0.69	1.00	1.00	1.19	1.31	0.42	0.76	0.99	1.00
N-acetylalanine	Amino Acid	Alanine and Aspartate Metabol	1.00	1.03	0.57	1.17	1.00	1.00	1.05	1.00	0.15	1.12	0.79	1.00	1.04	1.08	0.61	1.21	0.99	1.00
N-acetylarginine	Amino Acid	Urea cycle; Arginine and Prolin	1.04	1.04	0.70	1.13	1.00	1.00	1.09	1.03	0.66	1.06	1.00	1.00	1.13	1.06	0.44	1.12	0.99	1.00
N-acetylasparagine	Amino Acid	Alanine and Aspartate Metabol	1.09	1.06	0.91	0.99	1.00	1.00	1.05	1.05	0.97	0.98	1.00	1.00	1.07	1.13	0.96	0.98	0.99	1.00
N-acetyl-beta-alanine	Nucleotide	Pyrimidine Metabolism, Uracil	1.20	1.14	0.68	0.61	1.00	1.00	1.39	1.22	0.12	0.92	0.76	1.00	1.45	1.33	0.34	0.79	0.99	1.00
N-acetylglutamate	Amino Acid	Glutamate Metabolism	0.95	0.87	0.41	1.24	1.00	1.00	1.00	0.87	0.23	1.46	0.84	1.00	0.99	0.93	0.32	1.18	0.99	1.00
N-acetylglutamine	Amino Acid	Glutamate Metabolism	0.95	1.03	0.21	0.92	1.00	1.00	1.08	0.98	0.34	0.91	0.88	1.00	0.96	1.00	0.39	0.42	0.99	1.00
N-acetylglycine	Amino Acid	Glycine, Serine and Threonine	1.13	1.14	0.86	0.10	1.00	1.00	1.10	1.07	0.89	0.33	1.00	1.00	1.06	1.02	0.59	0.46	0.99	1.00
N-acetyl-isoputreanine*	Amino Acid	Polyamine Metabolism	1.14	1.13	0.89	0.84	1.00	1.00	1.10	1.05	0.75	0.71	1.00	1.00	1.11	1.18	0.34	1.06	0.99	1.00
N-acetylleucine	Amino Acid	Leucine, Isoleucine and Valine	1.02	0.90	0.15	1.53	1.00	1.00	1.09	0.93	0.48	1.71	0.94	1.00	1.08	0.92	0.24	1.81	0.99	1.00

N-acetylmethionine	Amino Acid	Methionine, Cysteine, SAM an	1.00	1.01	0.96	1.07	1.00	1.00	1.07	0.98	0.07	1.54	0.73	1.00	1.08	1.04	0.10	1.28	0.99	1.00
N-acetylputrescine	Amino Acid	Polyamine Metabolism	1.09	1.03	0.43	1.15	1.00	1.00	1.08	1.03	0.54	1.04	0.97	1.00	1.12	1.04	0.24	1.27	0.99	1.00
N-acetylserine	Amino Acid	Glycine, Serine and Threonine	1.03	1.04	0.77	0.91	1.00	1.00	1.07	1.04	0.62	0.94	1.00	1.00	1.05	1.06	0.76	0.67	0.99	1.00
N-acetyltaurine	Amino Acid	Methionine, Cysteine, SAM an	0.99	1.03	0.55	1.44	1.00	1.00	1.03	0.99	0.94	1.02	1.00	1.00	1.02	1.04	0.53	1.11	0.99	1.00
N-acetylthreonine	Amino Acid	Glycine, Serine and Threonine	1.07	1.06	0.69	1.15	1.00	1.00	1.06	1.04	0.36	0.87	0.89	1.00	1.12	1.10	0.82	0.75	0.99	1.00
N-acetyltryptophan	Amino Acid	Tryptophan Metabolism	1.00	0.93	0.27	1.12	1.00	1.00	1.07	0.97	0.51	1.31	0.95	1.00	1.17	1.07	0.17	1.10	0.99	1.00
N-acetylvaline	Amino Acid	Leucine, Isoleucine and Valine	1.02	1.00	0.51	1.18	1.00	1.00	1.05	1.02	0.73	1.23	1.00	1.00	1.08	1.04	0.43	0.98	0.99	1.00
N-behenoyl-sphingadienine (d18:2/22:0)*	Lipid	Ceramides	1.16	1.02	0.13	1.45	1.00	1.00	1.09	1.02	0.53	0.77	0.96	1.00	1.07	1.05	0.76	0.85	0.99	1.00
N-delta-acetylornithine	Amino Acid	Urea cycle; Arginine and Prolin	1.01	0.96	0.59	0.74	1.00	1.00	0.99	1.00	0.36	0.58	0.89	1.00	0.98	1.14	0.55	1.21	0.99	1.00
N-formylmethionine	Amino Acid	Methionine, Cysteine, SAM an	0.99	0.98	0.76	1.15	1.00	1.00	1.02	0.99	0.56	1.23	0.97	1.00	1.05	1.05	0.61	0.97	0.99	1.00
N-methylpipecolate	Xenobiotics	Bacterial/Fungal	1.12	0.94	0.10	1.54	1.00	1.00	1.27	0.92	0.01	2.35	0.73	1.00	1.22	1.02	0.07	1.74	0.99	1.00
N-methylproline	Amino Acid	Urea cycle; Arginine and Prolin	2.84	1.46	0.68	1.23	1.00	1.00	1.17	2.67	0.61	1.21	1.00	1.00	2.01	2.44	0.96	0.43	0.99	1.00
N-oleoylserine	Lipid	Endocannabinoid	0.94	1.08	0.26	1.17	1.00	1.00	1.06	1.03	0.45	0.30	0.92	1.00	0.99	1.02	0.49	0.42	0.99	1.00
N-oleoyltaurine	Lipid	Endocannabinoid	0.99	1.22	0.09	1.54	1.00	1.00	0.96	1.18	0.13	1.47	0.76	1.00	0.91	1.08	0.14	1.60	0.99	1.00
nonadecanoate (19:0)	Lipid	Long Chain Fatty Acid	0.97	1.18	0.16	1.60	1.00	1.00	1.01	1.12	0.29	1.06	0.86	1.00	0.98	1.06	0.41	1.10	0.99	1.00
nonanoylcarnitine (C9)	Lipid	Fatty Acid Metabolism(Acyl Ca	1.26	1.18	0.36	0.55	1.00	1.00	1.22	1.22	0.80	0.14	1.00	1.00	1.30	1.11	0.41	1.45	0.99	1.00
N-palmitoylglycine	Lipid	Fatty Acid Metabolism(Acyl Gly	0.96	1.15	0.14	1.71	1.00	1.00	0.99	1.08	0.44	0.96	0.91	1.00	0.96	1.07	0.13	1.60	0.99	1.00
N-palmitoyl-heptadecasphingosine (d17:1/16:	Lipid	Ceramides	1.04	1.00	0.58	0.93	1.00	1.00	1.00	1.00	0.41	0.42	0.89	1.00	1.01	1.00	0.96	1.02	0.99	1.00
N-palmitoyl-sphingadienine (d18:2/16:0)*	Lipid	Ceramides	1.14	1.05	0.09	0.86	1.00	1.00	1.13	1.07	0.71	0.53	1.00	1.00	1.05	1.03	0.32	0.82	0.99	1.00
N-palmitoyl-sphinganine (d18:0/16:0)	Lipid	Dihydroceramides	1.01	0.83	0.15	1.57	1.00	1.00	0.94	0.83	0.41	1.01	0.89	1.00	1.02	0.84	0.21	1.78	0.99	1.00
N-palmitoyl-sphingosine (d18:1/16:0)	Lipid	Ceramides	1.04	0.96	0.43	1.13	1.00	1.00	0.96	0.92	0.80	0.57	1.00	1.00	0.98	0.95	0.76	1.00	0.99	1.00
N-stearoyl-sphingadienine (d18:2/18:0)*	Lipid	Ceramides	1.07	1.02	0.64	0.67	1.00	1.00	0.98	0.94	0.74	0.50	1.00	1.00	0.96	0.99	0.71	0.78	0.99	1.00
N-stearoyl-sphingosine (d18:1/18:0)*	Lipid	Ceramides	1.10	1.03	0.78	1.00	1.00	1.00	1.03	0.94	0.72	1.01	1.00	1.00	1.00	0.98	0.92	1.01	0.99	1.00
octadecadienedioate (C18:2-DC)*	Lipid	Fatty Acid, Dicarboxylate	1.06	0.96	0.76	0.51	1.00	1.00	1.46	0.95	0.61	0.82	1.00	1.00	1.06	1.09	0.70	0.31	0.99	1.00
octadecanedioate (C18)	Lipid	Fatty Acid, Dicarboxylate	1.02	1.06	0.87	0.37	1.00	1.00	0.93	1.01	0.68	0.65	1.00	1.00	1.06	1.01	0.31	0.49	0.99	1.00
octadecanedioylcarnitine (C18-DC)*	Lipid	Fatty Acid Metabolism(Acyl Ca	1.06	1.08	0.60	0.22	1.00	1.00	1.01	1.07	0.38	0.62	0.89	1.00	1.14	1.04	0.34	1.11	0.99	1.00
octadecenedioate (C18:1-DC)*	Lipid	Fatty Acid, Dicarboxylate	0.88	0.88	0.43	0.45	1.00	1.00	0.90	0.81	0.25	0.82	0.85	1.00	0.94	0.76	0.01	1.80	0.86	1.00
octadecenedioylcarnitine (C18:1-DC)*	Lipid	Fatty Acid Metabolism(Acyl Ca	1.01	1.08	0.63	0.47	1.00	1.00	0.97	1.01	0.69	0.35	1.00	1.00	1.00	0.97	0.74	0.34	0.99	1.00
octanoylcarnitine (C8)	Lipid	Fatty Acid Metabolism(Acyl Ca	1.27	1.36	0.94	0.47	1.00	1.00	1.23	1.41	0.71	0.77	1.00	1.00	1.23	1.24	0.85	0.28	0.99	1.00
oleate/vaccenate (18:1)	Lipid	Long Chain Fatty Acid	1.14	1.35	0.98	1.32	1.00	1.00	1.10	1.24	0.97	1.25	1.00	1.00	1.05	1.17	0.53	1.34	0.99	1.00
oleoyl ethanolamide	Lipid	Endocannabinoid	1.00	1.31	0.07	1.83	1.00	1.00	1.09	1.11	0.90	0.69	1.00	1.00	1.00	1.14	0.25	1.37	0.99	1.00
oleoylcarnitine (C18)	Lipid	Fatty Acid Metabolism(Acyl Ca	1.07	1.18	0.15	1.29	1.00	1.00	1.02	1.16	0.07	1.46	0.73	1.00	1.03	1.14	0.20	1.51	0.99	1.00
oleoylcholine	Lipid	Fatty Acid Metabolism (Acyl Cl	1.20	1.15	0.17	0.60	1.00	1.00	1.18	1.32	0.82	0.92	1.00	1.00	1.32	1.20	0.52	0.76	0.99	1.00
oleoyl-linoleoyl-glycerol (18:1/18:2) [1]	Lipid	Diacylglycerol	1.10	0.99	0.36	0.90	1.00	1.00	1.00	0.98	0.96	0.25	1.00	1.00	1.01	1.01	0.84	0.46	0.99	1.00
oleoyl-linoleoyl-glycerol (18:1/18:2) [2]	Lipid	Diacylglycerol	1.10	1.01	0.78	0.82	1.00	1.00	0.95	0.98	0.88	0.28	1.00	1.00	1.01	1.04	0.93	0.50	0.99	1.00
oleoyl-oleoyl-glycerol (18:1/18:1) [1]*	Lipid	Diacylglycerol	1.13	1.01	0.24	1.13	1.00	1.00	0.99	0.96	0.80	0.39	1.00	1.00	1.06	1.05	0.83	0.69	0.99	1.00
oleoyl-oleoyl-glycerol (18:1/18:1) [2]*	Lipid	Diacylglycerol	1.19	0.98	0.13	1.51	1.00	1.00	1.03	0.92	0.33	0.95	0.88	1.00	1.08	1.06	0.65	0.61	0.99	1.00
ornithine	Amino Acid	Urea cycle; Arginine and Prolin	1.02	0.97	0.65	1.37	1.00	1.00	1.04	0.96	0.14	1.61	0.78	1.00	1.05	1.02	0.41	1.19	0.99	1.00
orotate	Nucleotide	Pyrimidine Metabolism, Orota	0.94	0.98	0.70	1.18	1.00	1.00	1.05	0.96	0.66	1.19	1.00	1.00	0.99	0.97	0.87	0.99	0.99	1.00
orotidine	Nucleotide	Pyrimidine Metabolism, Orota	1.06	0.96	0.23	0.90	1.00	1.00	1.19	0.90	0.00	2.32	0.73	1.00	1.11	1.01	0.42	0.93	0.99	1.00
O-sulfo-L-tyrosine	Xenobiotics	Chemical	1.10	1.10	0.76	0.79	1.00	1.00	1.12	1.11	0.84	0.69	1.00	1.00	1.11	1.14	0.92	0.49	0.99	1.00
oxalate (ethanedioate)	Cofactors ar	Ascorbate and Aldarate Metabo	1.04	1.01	0.34	0.54	1.00	1.00	0.96	0.95	0.90	0.43	1.00	1.00	1.01	1.00	0.56	0.21	0.99	1.00
palmitate (16:0)	Lipid	Long Chain Fatty Acid	1.09	1.18	0.83	1.34	1.00	1.00	1.07	1.13	0.79	1.19	1.00	1.00	1.04	1.07	0.80	1.24	0.99	1.00
palmitoleate (16:1n7)	Lipid	Long Chain Fatty Acid	1.67	1.97	0.65	0.96	1.00	1.00	1.41	1.71	1.00	0.91	1.00	1.00	1.39	1.41	0.97	0.98	0.99	1.00
palmitoleoylcarnitine (C16:1)*	Lipid	Fatty Acid Metabolism(Acyl Ca	1.23	1.27	0.95	0.48	1.00	1.00	1.12	1.26	0.40	1.09	0.89	1.00	1.15	1.18	0.97	0.38	0.99	1.00
palmitoleoyl-linoleoyl-glycerol (16:1/18:2) [1]'	Lipid	Diacylglycerol	1.14	1.00	0.26	0.99	1.00	1.00	1.04	0.97	0.66	0.51	1.00	1.00	1.06	1.06	0.82	0.33	0.99	1.00
palmitoloelycholine	Lipid	Fatty Acid Metabolism (Acyl Cl	1.22	1.23	0.07	0.52	1.00	1.00	1.20	1.51	0.92	1.03	1.00	1.00	1.36	1.31	0.41	0.69	0.99	1.00
palmitoyl dihydrosphingomyelin (d18:0/16:0)	Lipid	Dihydrosphingomyelins	0.99	1.01	0.34	1.26	1.00	1.00	0.97	1.02	0.14	1.30	0.78	1.00	1.02	1.02	0.73	1.07	0.99	1.00
palmitoyl ethanolamide	Lipid	Endocannabinoid	0.98	1.16	0.08	1.57	1.00	1.00	1.00	1.00	0.72	0.71	1.00	1.00	0.99	1.01	0.88	0.80	0.99	1.00
palmitoyl sphingomyelin (d18:1/16:0)	Lipid	Sphingomyelins	0.98	1.01	0.32	1.50	1.00	1.00	0.96	1.02	0.06	1.77	0.73	1.00	1.00	1.02	0.72	1.10	0.99	1.00
palmitoylcarnitine (C16)	Lipid	Fatty Acid Metabolism(Acyl Ca	1.07	1.05	0.45	0.46	1.00	1.00	0.99	1.07	0.07	1.24	0.73	1.00	1.07	1.04	0.53	0.62	0.99	1.00
palmitoylcholine	Lipid	Fatty Acid Metabolism (Acyl Cl	1.10	1.13	0.29	0.67	1.00	1.00	1.11	1.34	0.53	1.13	0.96	1.00	1.22	1.22	0.81	0.75	0.99	1.00
palmitoyl-docosahexaenoyl-glycerol (16:0/22:6	Lipid	Diacylglycerol	1.10	1.15	0.70	0.48	1.00	1.00	0.91	1.22	0.03	1.76	0.73	1.00	1.01	1.10	0.41	0.79	0.99	1.00
palmitoyl-myristoyl-glycerol (16:0/14:0) [1]*	Lipid	Diacylglycerol	1.07	0.77	0.06	1.69	1.00	1.00	1.15	0.94	0.19	1.11	0.80	1.00	1.12	0.92	0.56	1.31	0.99	1.00

palmitoyl-oleoyl-glycerol (16:0/18:1) [1]*	Lipid	Diacylglycerol	1.07	0.92	0.12	1.36	1.00	1.00	0.98	0.93	0.75	0.60	1.00	1.00	1.01	0.95	0.80	0.93	0.99	1.00
palmitoyl-oleoyl-glycerol (16:0/18:1) [2]*	Lipid	Diacylglycerol	1.08	0.92	0.12	1.38	1.00	1.00	1.01	0.93	0.53	0.73	0.96	1.00	1.02	0.96	0.78	0.89	0.99	1.00
palmitoyl-palmitoyl-glycerol (16:0/16:0) [2]*	Lipid	Diacylglycerol	1.00	0.92	0.39	0.88	1.00	1.00	1.02	1.01	0.73	0.79	1.00	1.00	1.04	0.94	0.91	1.22	0.99	1.00
pantothenate (Vitamin B5)	Cofactors ar	Pantothenate and CoA Metabo	0.97	1.03	0.33	1.00	1.00	1.00	1.05	1.00	0.43	0.58	0.91	1.00	1.02	1.11	0.83	0.93	0.99	1.00
p-cresol sulfate	Xenobiotics	Benzoate Metabolism	0.94	1.21	0.17	1.92	1.00	1.00	1.02	1.25	0.23	1.54	0.84	1.00	1.15	1.16	0.56	0.53	0.99	1.00
pelargonate (9:0)	Lipid	Medium Chain Fatty Acid	0.98	1.07	0.81	1.04	1.00	1.00	1.01	0.99	0.17	0.66	0.79	1.00	1.02	0.96	0.24	0.89	0.99	1.00
pentadecanoate (15:0)	Lipid	Long Chain Fatty Acid	1.14	1.24	0.83	1.22	1.00	1.00	1.10	1.19	0.98	0.96	1.00	1.00	1.08	1.08	0.73	1.16	0.99	1.00
perfluorooctanesulfonate (PFOS)	Xenobiotics	Chemical	0.87	0.97	0.19	1.19	1.00	1.00	0.93	0.92	0.88	0.13	1.00	1.00	0.90	0.93	0.28	0.87	0.99	1.00
perfluorooctanoate (PFOA)*	Xenobiotics	Chemical	0.96	0.98	0.88	0.88	1.00	1.00	0.94	0.94	0.95	0.79	1.00	1.00	0.93	0.93	0.54	0.36	0.99	1.00
phenol sulfate	Amino Acid	Tyrosine Metabolism	1.15	1.04	0.36	0.64	1.00	1.00	1.05	0.99	0.91	0.70	1.00	1.00	1.30	1.10	0.48	1.11	0.99	1.00
phenylacetylglutamine	Peptide	Acetylated Peptides	1.06	1.09	0.78	0.83	1.00	1.00	1.11	1.14	0.86	0.70	1.00	1.00	1.27	1.15	0.25	0.98	0.99	1.00
phenylalanine	Amino Acid	Phenylalanine Metabolism	1.00	0.96	0.64	1.37	1.00	1.00	1.02	0.97	0.75	1.58	1.00	1.00	1.02	1.01	0.55	1.24	0.99	1.00
phenyllactate (PLA)	Amino Acid	Phenylalanine Metabolism	0.92	0.86	0.17	1.09	1.00	1.00	1.00	1.05	0.69	1.10	1.00	1.00	1.03	0.95	0.31	1.14	0.99	1.00
phenylpyruvate	Amino Acid	Phenylalanine Metabolism	0.97	0.93	0.68	0.70	1.00	1.00	0.99	1.00	0.68	0.93	1.00	1.00	1.02	1.05	0.72	0.88	0.99	1.00
phosphoethanolamine (PE)	Lipid	Phospholipid Metabolism	0.98	0.96	0.75	0.78	1.00	1.00	1.04	0.99	0.41	0.56	0.89	1.00	1.10	1.06	0.25	0.68	0.99	1.00
phytanate	Xenobiotics	Food Component/Plant	1.02	1.11	0.69	0.78	1.00	1.00	1.18	1.23	0.94	0.28	1.00	1.00	1.20	1.09	0.44	0.87	0.99	1.00
pipecolate	Amino Acid	Lysine Metabolism	0.91	1.19	0.55	1.10	1.00	1.00	1.28	1.09	0.97	0.60	1.00	1.00	1.09	1.59	0.40	1.35	0.99	1.00
pregnen-diol disulfate*	Lipid	Pregnenolone Steroids	1.07	1.13	0.67	0.48	1.00	1.00	1.04	0.98	0.18	0.78	0.79	1.00	1.09	1.06	0.80	0.55	0.99	1.00
pregnenediol sulfate (C21H34O5S)*	Lipid	Pregnenolone Steroids	1.02	1.05	0.61	0.47	1.00	1.00	1.00	1.05	0.23	0.88	0.84	1.00	1.06	1.09	0.54	0.48	0.99	1.00
pregnenetriol disulfate*	Lipid	Pregnenolone Steroids	1.06	1.10	0.96	0.56	1.00	1.00	1.07	1.00	0.28	0.83	0.85	1.00	1.10	1.05	0.60	0.66	0.99	1.00
pregnenetriol sulfate*	Lipid	Pregnenolone Steroids	1.04	1.02	0.65	0.66	1.00	1.00	1.04	0.99	0.61	0.79	1.00	1.00	1.11	1.02	0.14	0.98	0.99	1.00
prolylhydroxyproline	Amino Acid	Urea cycle; Arginine and Prolin	1.10	1.20	0.22	1.47	1.00	1.00	1.12	1.13	0.67	0.90	1.00	1.00	1.24	1.15	0.71	0.66	0.99	1.00
proline	Amino Acid	Urea cycle; Arginine and Prolin	0.99	0.95	0.69	1.34	1.00	1.00	1.06	0.95	0.03	1.58	0.73	1.00	1.06	0.97	0.12	1.74	0.99	1.00
propionylcarnitine (C3)	Lipid	Fatty Acid Metabolism (also BC	1.03	0.90	0.18	1.52	1.00	1.00	1.14	0.95	0.07	1.71	0.73	1.00	1.10	0.96	0.12	1.77	0.99	1.00
propionylglycine (C3)	Lipid	Fatty Acid Metabolism (also BC	0.94	0.91	0.99	0.96	1.00	1.00	1.12	1.01	0.96	1.12	1.00	1.00	1.19	0.94	0.12	1.95	0.99	1.00
propyl 4-hydroxybenzoate sulfate	Xenobiotics	Benzoate Metabolism	0.86	1.01	0.18	1.58	1.00	1.00	1.06	0.95	0.30	0.94	0.88	1.00	0.91	0.95	0.48	0.52	0.99	1.00
pseudouridine	Nucleotide	Pyrimidine Metabolism, Uracil	1.05	1.05	0.86	0.99	1.00	1.00	1.05	1.03	0.90	0.61	1.00	1.00	1.05	1.06	0.96	0.78	0.99	1.00
pyridoxate	Cofactors ar	Vitamin B6 Metabolism	0.86	0.91	0.93	0.63	1.00	1.00	1.03	0.93	0.82	0.79	1.00	1.00	0.94	1.77	0.09	1.21	0.99	1.00
pyroglutamine*	Amino Acid	Glutamate Metabolism	1.14	1.15	0.86	0.69	1.00	1.00	1.15	1.06	0.11	0.93	0.76	1.00	1.16	1.22	0.54	0.64	0.99	1.00
pyruvate	Carbohydrat	Glycolysis, Gluconeogenesis, a	1.11	1.13	0.49	0.61	1.00	1.00	1.04	1.17	0.28	1.09	0.85	1.00	1.06	1.08	0.76	0.29	0.99	1.00
quinate	Xenobiotics	Food Component/Plant	0.84	0.93	0.70	0.89	1.00	1.00	1.14	0.81	0.10	1.52	0.75	1.00	1.77	0.98	0.77	1.03	0.99	1.00
quinolinate	Cofactors ar	Nicotinate and Nicotinamide I	1.13	1.02	0.39	0.89	1.00	1.00	1.31	0.99	0.03	1.36	0.73	1.00	1.13	1.06	0.21	1.02	0.99	1.00
retinol (Vitamin A)	Cofactors ar	Vitamin A Metabolism	1.01	0.94	0.18	1.33	1.00	1.00	1.01	0.98	0.34	0.82	0.88	1.00	1.03	1.01	0.41	0.77	0.99	1.00
ribitol	Carbohydrat	Pentose Metabolism	0.99	1.04	0.73	0.99	1.00	1.00	1.04	1.00	0.68	0.68	1.00	1.00	1.00	1.06	0.26	0.90	0.99	1.00
ribonate (ribonolactone)	Carbohydrat	Pentose Metabolism	1.08	0.95	0.36	0.92	1.00	1.00	1.12	0.96	0.20	1.20	0.81	1.00	1.03	0.93	0.21	1.05	0.99	1.00
salicylate	Xenobiotics	Drug - Topical Agents	8.66	2.18	0.64	0.83	1.00	1.00	47.22	2.73	0.28	0.85	0.85	1.00	11.62	1.52	0.47	1.06	0.99	1.00
sebacate (C10-DC)	Lipid	Fatty Acid, Dicarboxylate	1.13	1.06	0.67	0.84	1.00	1.00	1.04	1.03	0.91	0.46	1.00	1.00	1.14	1.13	0.63	0.27	0.99	1.00
serine	Amino Acid	Glycine, Serine and Threonine	1.01	1.00	0.85	1.27	1.00	1.00	1.05	0.98	0.17	1.52	0.79	1.00	1.06	1.00	0.06	1.68	0.99	1.00
serotonin	Amino Acid	Tryptophan Metabolism	1.08	1.07	0.88	0.71	1.00	1.00	1.10	0.99	0.15	1.23	0.78	1.00	1.19	1.07	0.61	1.03	0.99	1.00
S-methylcysteine	Amino Acid	Methionine, Cysteine, SAM an	1.15	1.28	0.83	0.94	1.00	1.00	1.38	1.24	0.29	0.65	0.87	1.00	1.21	1.52	0.51	1.18	0.99	1.00
S-methylcysteine sulfoxide	Amino Acid	Methionine, Cysteine, SAM an	1.33	1.59	0.98	0.99	1.00	1.00	1.66	1.49	0.33	0.46	0.88	1.00	1.50	1.88	0.91	0.85	0.99	1.00
sphinganine-1-phosphate	Lipid	Sphingolipid Synthesis	1.02	1.06	0.14	0.95	1.00	1.00	1.09	1.12	0.49	0.37	0.95	1.00	1.07	1.08	0.44	0.87	0.99	1.00
sphingomyelin (d17:1/14:0, d16:1/15:0)*	Lipid	Sphingomyelins	0.99	0.97	0.81	0.80	1.00	1.00	0.95	1.12	0.04	1.89	0.73	1.00	1.04	1.04	0.88	0.79	0.99	1.00
sphingomyelin (d17:1/16:0, d18:1/15:0, d16	Lipid	Sphingomyelins	0.99	0.99	0.87	1.21	1.00	1.00	0.99	1.03	0.41	1.14	0.89	1.00	1.01	1.01	0.89	1.05	0.99	1.00
sphingomyelin (d17:2/16:0, d18:2/15:0)*	Lipid	Sphingomyelins	1.01	0.98	0.63	0.92	1.00	1.00	0.95	1.10	0.03	1.77	0.73	1.00	1.01	1.01	0.82	0.77	0.99	1.00
sphingomyelin (d18:0/18:0, d19:0/17:0)*	Lipid	Dihydrosphingomyelins	1.00	1.01	0.44	0.81	1.00	1.00	0.92	1.00	0.09	1.17	0.75	1.00	0.99	0.96	0.89	1.03	0.99	1.00
sphingomyelin (d18:0/20:0, d16:0/22:0)*	Lipid	Dihydrosphingomyelins	1.04	1.01	0.84	0.75	1.00	1.00	0.90	1.04	0.06	1.39	0.73	1.00	1.04	0.99	0.90	0.93	0.99	1.00
sphingomyelin (d18:1/14:0, d16:1/16:0)*	Lipid	Sphingomyelins	1.00	0.98	0.67	1.19	1.00	1.00	0.98	1.04	0.16	1.64	0.79	1.00	1.02	1.01	0.59	0.98	0.99	1.00
sphingomyelin (d18:1/17:0, d17:1/18:0, d19	Lipid	Sphingomyelins	1.00	1.01	0.41	1.08	1.00	1.00	0.96	1.01	0.13	1.37	0.77	1.00	0.99	0.98	0.96	0.82	0.99	1.00
sphingomyelin (d18:1/18:1, d18:2/18:0)	Lipid	Sphingomyelins	0.99	1.02	0.43	1.29	1.00	1.00	0.97	1.02	0.12	1.30	0.76	1.00	0.98	1.00	0.72	0.75	0.99	1.00
sphingomyelin (d18:1/19:0, d19:1/18:0)*	Lipid	Sphingomyelins	1.01	0.98	0.89	0.89	1.00	1.00	0.96	1.03	0.09	1.29	0.74	1.00	1.02	0.97	0.53	1.09	0.99	1.00
sphingomyelin (d18:1/20:0, d16:1/22:0)*	Lipid	Sphingomyelins	1.02	1.00	0.90	1.12	1.00	1.00	0.98	1.00	0.44	0.80	0.91	1.00	1.03	0.99	0.25	1.19	0.99	1.00
sphingomyelin (d18:1/20:1, d18:2/20:0)*	Lipid	Sphingomyelins	0.99	1.02	0.56	1.15	1.00	1.00	0.96	1.03	0.08	1.37	0.74	1.00	0.99	1.01	0.44	0.77	0.99	1.00
sphingomyelin (d18:1/20:2, d18:2/20:1, d16	Lipid	Sphingomyelins	1.06	1.07	0.40	0.62	1.00	1.00	1.02	1.09	0.78	0.96	1.00	1.00	1.03	1.10	0.59	0.99	0.99	1.00

sphingomyelin (d18:1/21:0, d17:1/22:0, d16	Lipid	Sphingomyelins	1.07	0.97	0.24	1.22	1.00	1.00	1.00	1.07	0.10	1.00	0.75	1.00	1.10	1.00	0.15	1.56	0.99	1.00
sphingomyelin (d18:1/22:1, d18:2/22:0, d16	Lipid	Sphingomyelins	0.99	0.99	0.87	1.16	1.00	1.00	0.96	1.01	0.10	1.50	0.76	1.00	1.02	1.00	0.57	0.94	0.99	1.00
sphingomyelin (d18:1/22:2, d18:2/22:1, d16	Lipid	Sphingomyelins	0.97	1.01	0.22	1.17	1.00	1.00	0.97	1.04	0.04	1.57	0.73	1.00	0.99	1.02	0.26	1.20	0.99	1.00
sphingomyelin (d18:1/24:1, d18:2/24:0)*	Lipid	Sphingomyelins	1.00	0.98	0.90	1.26	1.00	1.00	0.94	0.98	0.13	1.21	0.77	1.00	1.01	0.98	0.64	1.07	0.99	1.00
sphingomyelin (d18:2/14:0, d18:1/14:1)*	Lipid	Sphingomyelins	1.00	0.98	0.86	0.89	1.00	1.00	0.97	1.13	0.04	1.90	0.73	1.00	1.03	1.04	0.73	0.80	0.99	1.00
sphingomyelin (d18:2/16:0, d18:1/16:1)*	Lipid	Sphingomyelins	1.00	1.01	0.94	1.28	1.00	1.00	0.99	1.03	0.30	1.23	0.88	1.00	1.00	1.01	0.91	0.91	0.99	1.00
sphingomyelin (d18:2/18:1)*	Lipid	Sphingomyelins	1.04	1.03	0.86	0.86	1.00	1.00	1.01	1.05	0.41	0.79	0.89	1.00	1.02	1.01	1.00	0.58	1.00	1.00
sphingomyelin (d18:2/21:0, d16:2/23:0)*	Lipid	Sphingomyelins	1.02	0.98	0.52	0.97	1.00	1.00	0.95	1.07	0.01	1.89	0.73	1.00	1.03	1.00	0.34	0.87	0.99	1.00
sphingomyelin (d18:2/23:0, d18:1/23:1, d17	Lipid	Sphingomyelins	1.09	1.01	0.35	0.78	1.00	1.00	1.04	1.11	0.04	0.55	0.73	1.00	1.13	1.02	0.13	1.07	0.99	1.00
sphingomyelin (d18:2/23:1)*	Lipid	Sphingomyelins	1.00	0.98	0.89	0.92	1.00	1.00	0.97	1.05	0.02	1.67	0.73	1.00	1.01	1.00	0.71	0.85	0.99	1.00
sphingomyelin (d18:2/24:1, d18:1/24:2)*	Lipid	Sphingomyelins	0.99	0.99	0.89	1.19	1.00	1.00	0.97	1.01	0.09	1.43	0.75	1.00	1.01	1.00	0.74	0.79	0.99	1.00
sphingomyelin (d18:2/24:2)*	Lipid	Sphingomyelins	0.98	1.00	0.77	1.00	1.00	1.00	0.98	1.03	0.20	1.03	0.81	1.00	1.00	1.02	0.62	0.79	0.99	1.00
sphingosine 1-phosphate	Lipid	Sphingosines	0.95	1.03	0.08	1.64	1.00	1.00	1.00	1.08	0.05	1.16	0.73	1.00	1.03	1.08	0.23	1.30	0.99	1.00
stearate (18:0)	Lipid	Long Chain Fatty Acid	1.01	1.13	0.35	1.41	1.00	1.00	1.04	1.10	0.57	0.93	0.97	1.00	1.00	1.08	0.29	1.16	0.99	1.00
stearidonate (18:4n3)	Lipid	Polyunsaturated Fatty Acid (n3	1.39	1.30	0.87	1.10	1.00	1.00	1.29	1.39	0.48	0.78	0.94	1.00	1.21	1.19	0.50	0.82	0.99	1.00
stearoyl sphingomyelin (d18:1/18:0)	Lipid	Sphingomyelins	0.99	1.01	0.55	0.92	1.00	1.00	0.97	0.98	0.70	0.55	1.00	1.00	0.99	0.97	0.68	0.75	0.99	1.00
stearoyl-arachidonoyl-glycerol (18:0/20:4) [1]*	Lipid	Diacylglycerol	1.09	1.12	0.88	0.59	1.00	1.00	1.05	1.18	0.33	1.07	0.88	1.00	1.10	1.12	0.55	0.90	0.99	1.00
stearoylcarnitine (C18)	Lipid	Fatty Acid Metabolism(Acyl Ca	1.04	1.01	0.52	0.58	1.00	1.00	1.01	1.05	0.80	0.39	1.00	1.00	1.06	1.02	0.46	0.70	0.99	1.00
succinate	Energy	TCA Cycle	1.05	1.04	0.73	0.79	1.00	1.00	1.03	1.01	0.99	0.84	1.00	1.00	1.07	1.10	0.61	0.68	0.99	1.00
succinimide	Xenobiotics	Chemical	1.16	1.13	0.78	0.54	1.00	1.00	1.14	1.06	0.10	0.89	0.75	1.00	1.09	1.07	0.60	0.57	0.99	1.00
sulfate*	Xenobiotics	Chemical	1.00	0.96	0.44	1.26	1.00	1.00	1.01	0.94	0.14	1.46	0.78	1.00	1.03	0.97	0.14	1.65	0.99	1.00
tartronate (hydroxymalonate)	Xenobiotics	Food Component/Plant	1.07	1.09	0.73	0.98	1.00	1.00	1.02	0.97	0.83	0.73	1.00	1.00	1.07	1.06	0.84	0.74	0.99	1.00
taurine	Amino Acid	Methionine, Cysteine, SAM an	0.94	0.97	0.63	1.39	1.00	1.00	1.03	0.96	0.17	1.43	0.79	1.00	1.03	1.00	0.67	1.00	0.99	1.00
taurochenodeoxycholate	Lipid	Primary Bile Acid Metabolism	1.43	1.02	0.23	1.16	1.00	1.00	1.60	1.81	0.77	1.04	1.00	1.00	1.38	1.35	0.43	0.31	0.99	1.00
taurocholate	Lipid	Primary Bile Acid Metabolism	1.13	1.19	0.86	0.35	1.00	1.00	1.15	1.44	0.53	1.13	0.96	1.00	1.20	1.10	0.94	0.51	0.99	1.00
taurocholenate sulfate*	Lipid	Secondary Bile Acid Metabolis	0.92	1.09	0.48	0.74	1.00	1.00	0.98	0.97	0.96	0.57	1.00	1.00	0.98	0.94	0.53	0.37	0.99	1.00
tetradecadienoate (14:2)*	Lipid	Polyunsaturated Fatty Acid (n3	1.46	1.46	0.48	0.68	1.00	1.00	1.38	1.49	0.31	0.73	0.88	1.00	1.41	1.34	0.77	0.67	0.99	1.00
tetradecanedioate (C14)	Lipid	Fatty Acid, Dicarboxylate	1.06	1.04	0.24	0.53	1.00	1.00	0.93	1.02	0.95	0.64	1.00	1.00	1.00	0.95	0.40	0.57	0.99	1.00
theobromine	Xenobiotics	Xanthine Metabolism	1.13	0.97	0.82	0.71	1.00	1.00	1.12	1.09	0.36	0.65	0.89	1.00	1.23	1.32	0.82	0.62	0.99	1.00
theophylline	Xenobiotics	Xanthine Metabolism	0.93	0.92	0.80	0.67	1.00	1.00	1.15	0.96	0.33	1.18	0.88	1.00	1.32	1.03	0.97	1.02	0.99	1.00
thioproline	Xenobiotics	Chemical	1.24	1.11	0.27	1.09	1.00	1.00	1.18	1.11	0.29	0.92	0.86	1.00	1.23	1.20	0.40	0.69	0.99	1.00
threonate	Cofactors ar	Ascorbate and Aldarate Metabc	1.03	1.02	0.53	0.21	1.00	1.00	0.97	0.95	0.52	0.22	0.96	1.00	0.97	1.00	0.34	0.38	0.99	1.00
threonine	Amino Acid	Glycine, Serine and Threonine	1.05	0.99	0.44	1.21	1.00	1.00	1.11	0.99	0.03	1.70	0.73	1.00	1.16	1.03	0.09	2.02	0.99	1.00
thyroxine	Amino Acid	Tyrosine Metabolism	0.99	0.98	0.56	0.22	1.00	1.00	1.00	0.97	0.65	0.49	1.00	1.00	1.01	1.00	0.46	0.18	0.99	1.00
hydroxyproline	Amino Acid	Urea cycle; Arginine and Prolin	1.07	1.09	0.71	1.07	1.00	1.00	1.12	1.06	0.39	0.57	0.89	1.00	1.24	1.16	0.49	0.73	0.99	1.00
tricosanoyl sphingomyelin (d18:1/23:0)*	Lipid	Sphingomyelins	1.09	0.98	0.10	1.19	1.00	1.00	0.99	0.98	0.62	0.21	1.00	1.00	1.11	0.97	0.14	1.73	0.99	1.00
tridecenedioate (C13:1-DC)*	Lipid	Fatty Acid, Dicarboxylate	1.16	1.14	0.52	0.48	1.00	1.00	1.05	1.13	0.83	0.49	1.00	1.00	1.03	0.96	0.21	0.61	0.99	1.00
triethanolamine	Xenobiotics	Chemical	0.94	0.95	1.00	1.28	1.00	1.00	0.94	0.89	0.40	1.07	0.89	1.00	1.01	1.02	0.56	0.82	0.99	1.00
trigonelline (N'-methylnicotinate)	Cofactors ar	Nicotinate and Nicotinamide I	1.08	0.82	0.17	1.20	1.00	1.00	1.23	0.79	0.01	1.72	0.73	1.00	1.47	0.91	0.09	1.42	0.99	1.00
trimethylamine N-oxide	Lipid	Phospholipid Metabolism	1.35	1.15	0.61	0.45	1.00	1.00	1.60	1.15	0.99	1.05	1.00	1.00	1.85	1.26	0.53	1.19	0.99	1.00
tryptophan	Amino Acid	Tryptophan Metabolism	1.00	0.94	0.44	1.35	1.00	1.00	1.02	0.94	0.25	1.58	0.85	1.00	1.05	1.01	0.36	1.11	0.99	1.00
tyrosine	Amino Acid	Tyrosine Metabolism	1.03	0.92	0.10	1.70	1.00	1.00	1.06	0.95	0.06	1.95	0.73	1.00	1.07	0.97	0.06	1.91	0.99	1.00
urate	Nucleotide	Purine Metabolism, (Hypo)Xar	1.02	1.02	0.88	1.05	1.00	1.00	1.04	1.03	0.98	0.96	1.00	1.00	1.04	1.06	0.93	0.95	0.99	1.00
urea	Amino Acid	Urea cycle; Arginine and Prolin	1.04	0.93	0.09	1.62	1.00	1.00	1.10	0.98	0.13	1.55	0.76	1.00	1.09	0.99	0.14	1.61	0.99	1.00
uridine	Nucleotide	Pyrimidine Metabolism, Uracil	1.06	1.11	1.00	0.85	1.00	1.00	1.05	1.10	0.77	0.48	1.00	1.00	1.06	1.14	0.27	1.19	0.99	1.00
valine	Amino Acid	Leucine, Isoleucine and Valine	1.03	0.94	0.26	1.59	1.00	1.00	1.08	0.97	0.05	1.86	0.73	1.00	1.04	0.98	0.13	1.44	0.99	1.00
vanillylmandelate (VMA)	Amino Acid	Tyrosine Metabolism	1.08	1.07	0.64	0.77	1.00	1.00	1.05	1.03	0.74	1.00	1.00	1.00	1.04	1.05	0.92	0.67	0.99	1.00
xanthine	Nucleotide	Purine Metabolism, (Hypo)Xar	0.99	1.09	0.30	1.21	1.00	1.00	1.02	1.05	0.66	0.52	1.00	1.00	1.02	1.11	0.22	1.49	0.99	1.00
ximenoylcarnitine (C26:1)*	Lipid	Fatty Acid Metabolism(Acyl Ca	0.96	0.97	0.41	0.92	1.00	1.00	0.92	0.98	0.22	0.99	0.84	1.00	0.99	1.00	0.97	0.71	0.99	1.00
X - 09789	Unknown	Unknown	1.09	1.12	0.91	0.25	1.00	1.00	0.88	1.04	0.71	0.81	1.00	1.00	0.83	1.19	0.13	1.92	0.99	1.00
X - 11261	Unknown	Unknown	1.03	1.09	0.67	0.67	1.00	1.00	1.00	1.07	0.32	0.75	0.88	1.00	1.09	1.16	0.82	0.59	0.99	1.00
X - 11308	Unknown	Unknown	1.03	1.00	0.62	0.99	1.00	1.00	1.05	0.98	0.21	1.12	0.83	1.00	1.04	1.02	0.66	0.59	0.99	1.00
X - 11315	Unknown	Unknown	1.03	1.05	0.61	0.79	1.00	1.00	1.08	1.02	0.48	0.80	0.94	1.00	1.02	1.04	0.74	0.55	0.99	1.00
X - 11372	Unknown	Unknown	1.11	0.98	0.16	1.50	1.00	1.00	1.14	0.98	0.04	1.80	0.73	1.00	1.11	1.06	0.95	0.67	0.99	1.00

X - 11444	Unknown	Unknown	1.10	1.11	0.65	0.63	1.00	1.00	1.09	1.10	0.64	0.39	1.00	1.00	1.02	1.09	0.33	1.54	0.99	1.00
X - 11470	Unknown	Unknown	1.15	1.13	0.92	0.39	1.00	1.00	1.11	1.14	0.51	0.48	0.95	1.00	1.19	1.24	0.51	0.45	0.99	1.00
X - 11530	Unknown	Unknown	1.32	1.26	0.50	0.38	1.00	1.00	1.10	1.10	0.85	0.36	1.00	1.00	1.30	1.20	0.96	0.49	0.99	1.00
X - 11787	Unknown	Unknown	1.03	1.00	0.56	1.29	1.00	1.00	1.06	0.99	0.19	1.45	0.80	1.00	1.09	1.02	0.14	1.26	0.99	1.00
X - 11795	Unknown	Unknown	1.14	1.12	0.91	0.95	1.00	1.00	1.19	1.08	0.62	0.93	1.00	1.00	1.14	1.06	0.39	0.75	0.99	1.00
X - 12026	Unknown	Unknown	1.01	1.10	0.78	1.21	1.00	1.00	1.04	1.06	0.92	0.90	1.00	1.00	1.11	1.14	0.88	0.95	0.99	1.00
X - 12063	Unknown	Unknown	1.03	1.03	0.59	0.64	1.00	1.00	1.06	1.04	0.72	0.51	1.00	1.00	1.07	1.07	0.86	0.70	0.99	1.00
X - 12100	Unknown	Unknown	1.07	1.02	0.41	1.12	1.00	1.00	1.12	0.98	0.07	1.63	0.73	1.00	1.10	1.05	0.49	1.11	0.99	1.00
X - 12104	Unknown	Unknown	1.08	1.14	0.44	1.16	1.00	1.00	1.08	1.08	0.97	0.37	1.00	1.00	1.08	1.18	0.18	1.70	0.99	1.00
X - 12206	Unknown	Unknown	1.02	1.05	0.96	1.05	1.00	1.00	1.00	1.03	0.87	0.80	1.00	1.00	1.04	1.07	0.68	0.78	0.99	1.00
X - 12216	Unknown	Unknown	1.15	1.18	0.90	0.50	1.00	1.00	1.18	1.26	0.78	0.56	1.00	1.00	1.42	1.21	0.36	1.01	0.99	1.00
X - 12230	Unknown	Unknown	0.88	0.67	0.20	1.00	1.00	1.00	1.25	0.89	0.17	1.14	0.79	1.00	1.17	1.00	0.05	0.56	0.99	1.00
X - 12411	Unknown	Unknown	1.02	0.95	0.29	0.61	1.00	1.00	1.14	0.93	0.18	1.37	0.79	1.00	1.27	1.22	0.39	0.52	0.99	1.00
X - 12462	Unknown	Unknown	1.06	1.04	0.82	0.94	1.00	1.00	1.10	0.98	0.14	1.06	0.78	1.00	1.16	1.00	0.08	1.40	0.99	1.00
X - 12472	Unknown	Unknown	1.36	1.47	0.41	0.66	1.00	1.00	1.12	1.19	0.53	0.61	0.96	1.00	1.34	1.14	0.34	1.04	0.99	1.00
X - 12524	Unknown	Unknown	0.92	1.04	0.58	0.95	1.00	1.00	0.99	1.01	0.85	0.37	1.00	1.00	0.97	1.03	0.42	0.79	0.99	1.00
X - 12544	Unknown	Unknown	1.45	1.68	0.13	0.48	1.00	1.00	1.94	1.41	0.99	0.84	1.00	1.00	1.49	1.19	0.43	0.96	0.99	1.00
X - 12844	Unknown	Unknown	1.10	1.11	0.60	0.60	1.00	1.00	1.10	1.06	0.39	0.68	0.89	1.00	1.04	1.06	0.94	0.72	0.99	1.00
X - 12846	Unknown	Unknown	1.06	1.09	0.82	0.89	1.00	1.00	1.07	1.13	0.64	1.14	1.00	1.00	1.15	1.19	0.87	1.06	0.99	1.00
X - 13431	Unknown	Unknown	1.24	1.00	0.04	1.55	1.00	1.00	1.36	1.12	0.09	1.46	0.75	1.00	1.31	1.03	0.03	2.17	0.86	1.00
X - 13729	Unknown	Unknown	1.09	1.29	0.17	1.17	1.00	1.00	1.08	1.39	0.20	1.46	0.81	1.00	1.27	1.27	0.97	0.27	0.99	1.00
X - 13866	Unknown	Unknown	1.19	1.21	0.93	0.25	1.00	1.00	1.08	1.17	0.71	0.50	1.00	1.00	1.08	1.18	0.64	0.46	0.99	1.00
X - 14056	Unknown	Unknown	1.34	1.12	0.79	1.04	1.00	1.00	1.32	1.17	0.61	1.06	1.00	1.00	1.39	1.17	0.35	1.09	0.99	1.00
X - 14939	Unknown	Unknown	1.07	1.23	0.24	0.78	1.00	1.00	1.04	1.06	0.95	0.26	1.00	1.00	1.12	1.23	0.30	0.72	0.99	1.00
X - 15245	Unknown	Unknown	1.14	1.10	0.78	0.40	1.00	1.00	1.07	1.16	0.77	0.73	1.00	1.00	1.13	1.05	0.50	0.66	0.99	1.00
X - 15469	Unknown	Unknown	1.15	1.18	0.93	0.40	1.00	1.00	1.09	1.23	0.38	0.84	0.89	1.00	1.08	1.12	0.64	0.38	0.99	1.00
X - 15486	Unknown	Unknown	1.08	1.32	0.19	1.22	1.00	1.00	1.11	1.15	0.82	0.47	1.00	1.00	1.28	1.37	0.91	0.37	0.99	1.00
X - 15492	Unknown	Unknown	1.27	1.27	0.85	0.50	1.00	1.00	1.22	1.25	0.94	0.50	1.00	1.00	1.28	1.37	0.54	0.93	0.99	1.00
X - 15503	Unknown	Unknown	1.11	0.97	0.01	1.71	1.00	1.00	1.13	1.00	0.10	1.58	0.75	1.00	1.17	1.03	0.02	1.87	0.86	1.00
X - 16087	Unknown	Unknown	1.17	1.22	0.88	0.24	1.00	1.00	1.15	1.11	0.75	0.26	1.00	1.00	1.18	1.11	0.34	0.40	0.99	1.00
X - 16580	Unknown	Unknown	1.17	1.11	0.53	0.42	1.00	1.00	1.13	1.02	0.38	0.84	0.89	1.00	1.30	1.07	0.19	1.24	0.99	1.00
X - 16938	Unknown	Unknown	1.25	1.18	0.58	0.62	1.00	1.00	1.17	0.99	0.04	0.87	0.73	1.00	1.31	1.10	0.36	1.16	0.99	1.00
X - 16944	Unknown	Unknown	1.01	1.13	0.75	0.81	1.00	1.00	0.98	0.95	0.90	0.34	1.00	1.00	1.10	1.14	0.89	0.37	0.99	1.00
X - 17335	Unknown	Unknown	1.19	1.21	0.87	0.41	1.00	1.00	1.05	1.18	0.78	0.83	1.00	1.00	1.02	1.11	0.86	0.74	0.99	1.00
X - 17337	Unknown	Unknown	1.23	1.11	0.25	0.74	1.00	1.00	1.19	1.10	0.30	0.74	0.88	1.00	1.14	1.02	0.30	1.17	0.99	1.00
X - 17340	Unknown	Unknown	1.19	1.24	0.62	0.57	1.00	1.00	1.18	1.24	0.68	0.56	1.00	1.00	1.26	1.31	0.71	0.60	0.99	1.00
X - 17357	Unknown	Unknown	1.10	1.07	0.38	0.45	1.00	1.00	1.10	1.03	0.36	0.81	0.89	1.00	0.97	0.96	0.66	0.48	0.99	1.00
X - 17653	Unknown	Unknown	1.10	1.05	0.68	0.96	1.00	1.00	1.17	1.08	0.51	0.76	0.95	1.00	1.20	1.15	0.85	0.50	0.99	1.00
X - 17654	Unknown	Unknown	1.06	0.98	0.12	1.20	1.00	1.00	1.08	0.98	0.16	1.29	0.79	1.00	1.08	1.03	0.53	0.88	0.99	1.00
X - 17676	Unknown	Unknown	0.94	0.80	0.05	1.39	1.00	1.00	1.09	0.80	0.02	2.11	0.73	1.00	0.99	0.89	0.08	1.01	0.99	1.00
X - 18249	Unknown	Unknown	0.94	0.93	0.38	0.33	1.00	1.00	0.91	0.89	0.80	0.47	1.00	1.00	0.83	0.85	0.38	0.49	0.99	1.00
X - 18779	Unknown	Unknown	1.01	0.99	0.63	0.83	1.00	1.00	1.14	1.02	0.18	1.22	0.79	1.00	1.05	0.99	0.33	0.70	0.99	1.00
X - 18913	Unknown	Unknown	0.89	0.80	0.08	0.56	1.00	1.00	0.84	0.75	0.16	0.92	0.79	1.00	0.85	0.74	0.18	1.02	0.99	1.00
X - 18914	Unknown	Unknown	0.96	0.97	0.96	0.27	1.00	1.00	1.00	0.90	0.06	1.69	0.73	1.00	0.95	0.89	0.19	1.35	0.99	1.00
X - 18921	Unknown	Unknown	1.02	1.26	0.14	1.37	1.00	1.00	0.99	1.11	0.19	0.80	0.80	1.00	1.01	1.11	0.86	0.63	0.99	1.00
X - 18922	Unknown	Unknown	1.07	1.05	0.91	0.57	1.00	1.00	1.07	0.99	0.34	0.68	0.88	1.00	1.11	1.07	0.59	0.53	0.99	1.00
X - 19141	Unknown	Unknown	0.98	1.03	0.56	0.85	1.00	1.00	1.08	0.98	0.57	0.80	0.97	1.00	1.24	1.10	0.39	0.91	0.99	1.00
X - 21258	Unknown	Unknown	1.69	1.13	0.51	0.87	1.00	1.00	2.38	1.31	0.15	1.37	0.78	1.00	1.44	1.09	0.43	1.05	0.99	1.00
X - 21286	Unknown	Unknown	1.08	1.12	0.68	0.89	1.00	1.00	1.10	1.14	0.76	1.06	1.00	1.00	1.20	1.08	0.26	1.26	0.99	1.00
X - 21310	Unknown	Unknown	1.09	0.96	0.13	1.43	1.00	1.00	1.13	1.05	0.51	1.10	0.95	1.00	1.19	1.02	0.12	1.72	0.99	1.00
X - 21319	Unknown	Unknown	1.16	1.17	0.61	0.15	1.00	1.00	1.07	0.99	0.60	0.52	0.99	1.00	1.22	1.21	0.98	0.05	0.99	1.00
X - 21339	Unknown	Unknown	1.09	1.01	0.15	0.79	1.00	1.00	1.13	1.04	0.28	0.81	0.85	1.00	1.10	1.14	0.64	0.75	0.99	1.00
X - 21353	Unknown	Unknown	1.21	1.30	0.61	0.77	1.00	1.00	1.22	1.30	0.93	0.81	1.00	1.00	1.16	1.18	0.93	0.58	0.99	1.00
X - 21383	Unknown	Unknown	1.12	1.13	0.80	0.19	1.00	1.00	1.08	1.22	0.91	0.65	1.00	1.00	1.19	1.16	0.89	0.23	0.99	1.00

X - 21411	Unknown	Unknown	0.99	0.89	0.38	0.80	1.00	1.00	0.86	0.96	0.38	1.25	0.89	1.00	0.93	1.09	0.48	1.26	0.99	1.00
X - 21628	Unknown	Unknown	1.00	1.03	0.94	0.99	1.00	1.00	0.98	0.92	0.35	1.04	0.89	1.00	1.02	1.00	0.62	0.44	0.99	1.00
X - 21736	Unknown	Unknown	1.12	1.12	0.78	0.26	1.00	1.00	1.11	1.00	0.19	0.93	0.80	1.00	1.18	1.05	0.39	0.96	0.99	1.00
X - 21785	Unknown	Unknown	1.05	1.00	0.29	0.96	1.00	1.00	1.03	1.00	0.94	0.63	1.00	1.00	1.06	1.04	0.91	0.59	0.99	1.00
X - 21796	Unknown	Unknown	1.05	1.03	0.84	0.49	1.00	1.00	1.01	0.98	0.80	0.56	1.00	1.00	1.04	1.09	0.44	0.64	0.99	1.00
X - 21829	Unknown	Unknown	1.06	1.08	0.94	0.12	1.00	1.00	0.98	1.04	0.56	0.59	0.97	1.00	1.03	1.02	0.47	0.15	0.99	1.00
X - 22162	Unknown	Unknown	1.10	1.04	0.57	1.12	1.00	1.00	1.11	1.03	0.29	1.13	0.86	1.00	1.08	1.16	0.68	1.19	0.99	1.00
X - 22519	Unknown	Unknown	1.20	1.12	0.53	0.62	1.00	1.00	1.18	1.12	0.43	0.46	0.91	1.00	1.09	1.11	0.83	0.14	0.99	1.00
X - 22771	Unknown	Unknown	1.19	1.01	0.09	1.11	1.00	1.00	1.14	0.96	0.07	1.18	0.73	1.00	1.23	1.06	0.19	1.19	0.99	1.00
X - 22775	Unknown	Unknown	0.99	1.00	0.93	1.35	1.00	1.00	0.99	0.97	0.84	1.25	1.00	1.00	1.01	1.01	0.92	1.02	0.99	1.00
X - 23314	Unknown	Unknown	2.58	1.02	0.25	1.63	1.00	1.00	1.69	1.36	0.73	0.52	1.00	1.00	1.43	1.23	0.61	0.64	0.99	1.00
X - 23369	Unknown	Unknown	1.00	0.95	0.49	1.19	1.00	1.00	1.02	0.84	0.10	1.67	0.75	1.00	1.09	0.92	0.05	1.98	0.99	1.00
X - 23585	Unknown	Unknown	1.08	1.09	0.92	0.59	1.00	1.00	1.08	1.09	0.98	0.60	1.00	1.00	1.10	1.15	0.80	0.78	0.99	1.00
X - 23593	Unknown	Unknown	1.06	1.00	0.92	1.09	1.00	1.00	1.07	0.95	0.15	1.60	0.79	1.00	1.06	1.06	0.61	0.84	0.99	1.00
X - 23639	Unknown	Unknown	1.09	1.10	0.85	0.99	1.00	1.00	1.06	1.05	0.68	0.57	1.00	1.00	1.08	1.16	0.58	1.03	0.99	1.00
X - 23680	Unknown	Unknown	1.01	1.09	0.44	0.72	1.00	1.00	1.11	1.10	0.69	0.20	1.00	1.00	1.08	1.07	0.82	0.06	0.99	1.00
X - 23974	Unknown	Unknown	1.07	1.19	0.21	1.29	1.00	1.00	1.00	1.09	0.15	1.33	0.78	1.00	1.03	1.12	0.50	0.90	0.99	1.00
X - 23997	Unknown	Unknown	0.98	1.24	0.36	1.62	1.00	1.00	1.02	1.23	0.41	1.38	0.89	1.00	1.15	1.19	0.72	0.51	0.99	1.00
X - 24106 - retired for palmitoyl-sphingosine-p	Unknown	Unknown	1.01	1.01	0.83	1.00	1.00	1.00	0.98	1.01	0.98	0.65	1.00	1.00	1.02	1.00	0.90	0.74	0.99	1.00
X - 24435	Unknown	Unknown	0.99	0.89	0.88	0.95	1.00	1.00	1.00	0.94	0.57	0.76	0.97	1.00	0.96	0.85	0.97	1.12	0.99	1.00
X - 24549	Unknown	Unknown	1.01	0.90	0.17	0.79	1.00	1.00	1.08	1.09	0.41	0.51	0.89	1.00	1.07	1.13	0.85	0.36	0.99	1.00
X - 24588	Unknown	Unknown	0.95	0.97	0.86	1.03	1.00	1.00	0.94	0.94	0.90	0.91	1.00	1.00	0.94	0.99	0.69	1.25	0.99	1.00
X - 24699	Unknown	Unknown	1.12	1.03	0.22	1.34	1.00	1.00	1.06	1.01	0.46	0.92	0.93	1.00	1.06	1.07	0.83	0.84	0.99	1.00
X - 24765	Unknown	Unknown	1.00	1.04	0.95	1.39	1.00	1.00	1.07	1.00	0.13	1.22	0.76	1.00	1.07	1.06	0.32	0.94	0.99	1.00
X - 24813	Unknown	Unknown	1.05	0.98	0.31	1.18	1.00	1.00	1.05	0.96	0.18	1.30	0.79	1.00	1.06	0.97	0.17	1.31	0.99	1.00
X - 24951	Unknown	Unknown	1.21	0.98	0.15	1.05	1.00	1.00	1.11	1.17	0.41	0.69	0.89	1.00	1.20	1.05	0.63	0.90	0.99	1.00
X - 24952	Unknown	Unknown	1.12	0.99	0.53	0.98	1.00	1.00	1.20	1.29	0.45	0.66	0.92	1.00	1.26	1.21	0.58	0.45	0.99	1.00
X - 25422	Unknown	Unknown	1.19	1.03	0.11	1.42	1.00	1.00	1.23	1.04	0.01	1.44	0.73	1.00	1.17	1.07	0.06	1.20	0.99	1.00
(14 or 15)-methylpalmitate (a17:0 or i17:0)	Lipid	Fatty Acid, Branched	1.12	1.34	0.92	1.31	1.00	1.00	1.14	1.19	0.76	1.05	1.00	1.00	1.07	1.10	0.64	1.18	0.99	1.00
1-(1-enyl-oleoyl)-GPE (P-18:1)*	Lipid	Lysoplasmalogen	1.11	1.25	0.86	0.76	1.00	1.00	1.12	1.03	0.56	0.64	0.97	1.00	1.21	1.10	0.63	0.81	0.99	1.00
1,2,3-benzenetriol sulfate (2)	Xenobiotics	Chemical	2.33	1.35	0.91	0.69	1.00	1.00	6.35	1.75	0.92	1.30	1.00	1.00	1.51	4.55	0.72	1.12	0.99	1.00
1,3,7-trimethylurate	Xenobiotics	Xanthine Metabolism	0.83	0.76	0.41	0.67	1.00	1.00	1.10	0.80	0.09	1.16	0.75	1.00	1.69	0.91	0.48	1.26	0.99	1.00
12,13-DiHOME	Lipid	Fatty Acid, Dihydroxy	0.97	1.05	0.38	0.60	1.00	1.00	0.97	0.84	0.70	0.79	1.00	1.00	0.87	0.81	0.89	0.72	0.99	1.00
12-HETE	Lipid	Eicosanoid	1.18	4.39	0.12	1.07	1.00	1.00	2.23	4.14	0.53	0.60	0.96	1.00	2.26	4.04	0.64	0.86	0.99	1.00
12-HHTrE	Lipid	Eicosanoid	3.75	2.31	0.10	0.58	1.00	1.00	3.20	1.32	0.02	1.16	0.73	1.00	6.50	4.35	0.84	0.48	0.99	1.00
1-arachidonoyl-GPA (20:4)	Lipid	Lysophospholipid	4.37	1.14	0.03	0.82	1.00	1.00	7.64	1.14	0.42	1.08	0.90	1.00	4.35	1.11	0.79	1.06	0.99	1.00
1-carboxyethylisoleucine	Amino Acid	Leucine, Isoleucine and Valine	1.00	0.98	0.27	0.73	1.00	1.00	1.85	1.41	0.06	1.08	0.73	1.00	1.17	1.37	0.10	0.70	0.99	1.00
1-carboxyethylleucine	Amino Acid	Leucine, Isoleucine and Valine	0.98	1.18	0.24	0.76	1.00	1.00	1.58	1.51	0.23	0.86	0.84	1.00	1.62	1.56	0.09	0.62	0.99	1.00
1-carboxyethylvaline	Amino Acid	Leucine, Isoleucine and Valine	1.38	1.57	0.45	0.73	1.00	1.00	0.96	1.89	0.96	1.42	1.00	1.00	1.86	2.02	0.71	0.32	0.99	1.00
1-heptadecenoylglycerol (17:1)*	Lipid	Monoacylglycerol	1.23	1.15	0.74	0.30	1.00	1.00	1.35	1.01	0.26	0.96	0.85	1.00	1.43	1.02	0.44	1.36	0.99	1.00
1-lignoceroyl-GPC (24:0)	Lipid	Lysophospholipid	4.23	0.95	0.74	0.90	1.00	1.00	3.88	1.11	0.04	0.78	0.73	1.00	5.65	1.05	0.56	1.03	0.99	1.00
1-linolenoylglycerol (18:3)	Lipid	Monoacylglycerol	1.15	1.04	0.78	0.48	1.00	1.00	1.09	1.28	0.63	1.01	1.00	1.00	1.26	1.20	0.79	0.28	0.99	1.00
1-linoleoyl-2-arachidonoyl-GPC (18:2/20:4n6)	Lipid	Phosphatidylcholine (PC)	521.35	176.35	0.46	0.53	1.00	1.00	528.03	124.90	0.81	0.64	1.00	1.00	543.52	192.80	0.21	0.61	0.99	1.00
1-linoleoyl-GPG (18:2)*	Lipid	Lysophospholipid	2.76	0.84	0.69	0.88	1.00	1.00	1.85	0.89	0.24	1.07	0.84	1.00	0.76	0.81	0.59	0.41	0.99	1.00
1-methyl-5-imidazoleacetate	Amino Acid	Histidine Metabolism	5.20	1.58	0.76	0.98	1.00	1.00	34.50	1.93	0.47	0.90	0.94	1.00	11.39	1.99	0.58	1.15	0.99	1.00
1-methylurate	Xenobiotics	Xanthine Metabolism	0.92	1.43	0.30	0.96	1.00	1.00	1.49	1.37	0.61	0.21	1.00	1.00	3.39	1.26	0.99	0.94	1.00	1.00
1-methylxanthine	Xenobiotics	Xanthine Metabolism	0.90	0.93	0.97	0.82	1.00	1.00	1.04	0.95	0.17	0.63	0.79	1.00	16.64	1.09	0.86	1.00	0.99	1.00
1-oleoyl-2-arachidonoyl-GPE (18:1/20:4)*	Lipid	Phosphatidylethanolamine (PI	89.18	41.04	0.30	0.65	1.00	1.00	88.82	56.49	0.75	0.40	1.00	1.00	75.04	40.28	0.36	0.61	0.99	1.00
1-palmitoleoyl-2-linolenoyl-GPC (16:1/18:3)*	Lipid	Phosphatidylcholine (PC)	1.03	43.70	0.52	1.34	1.00	1.00	1.00	52.33	0.03	1.45	0.73	1.00	1.14	44.03	0.39	1.74	0.99	1.00
1-palmitoyl-2-oleoyl-GPE (16:0/18:1)	Lipid	Phosphatidylethanolamine (PI	1.06	0.82	0.03	1.71	1.00	1.00	1.02	0.95	0.41	0.81	0.89	1.00	1.05	0.97	0.38	1.10	0.99	1.00
1-palmitoyl-GPG (16:0)*	Lipid	Lysophospholipid	0.86	0.92	0.82	0.32	1.00	1.00	1.03	0.90	0.22	0.62	0.83	1.00	0.88	0.95	0.75	0.50	0.99	1.00
1-stearoyl-2-oleoyl-GPE (18:0/18:1)	Lipid	Phosphatidylethanolamine (PI	0.92	0.77	0.19	1.31	1.00	1.00	0.91	0.96	0.98	0.97	1.00	1.00	1.01	0.92	0.49	1.14	0.99	1.00
1-stearoyl-2-oleoyl-GPS (18:0/18:1)	Lipid	Phosphatidylserine (PS)	4.15	5.87	0.46	0.47	1.00	1.00	3.90	6.99	0.07	0.63	0.73	1.00	10.50	6.49	0.96	0.52	0.99	1.00
1-stearoyl-GPG (18:0)	Lipid	Lysophospholipid	2.08	1.84	0.67	0.22	1.00	1.00	2.90	1.94	0.65	0.76	1.00	1.00	2.05	1.87	0.45	0.36	0.99	1.00

2,2'-Methylenebis(6-tert-butyl-p-cresol)	Xenobiotics	Chemical	1.40	1.49	0.67	0.60	1.00	1.00	1.85	1.44	0.78	1.13	1.00	1.00	1.37	1.18	0.53	1.15	0.99	1.00
2,3-dihydroxy-2-methylbutyrate	Amino Acid	Leucine, Isoleucine and Valine	0.91	2.29	1.00	0.77	1.00	1.00	1.10	1.73	0.53	0.65	0.96	1.00	1.06	1.86	0.61	0.88	0.99	1.00
2,3-dihydroxyisovalerate	Xenobiotics	Food Component/Plant	3.41	1.01	0.44	1.07	1.00	1.00	2.63	2.30	0.76	0.23	1.00	1.00	1.64	3.09	0.78	1.01	0.99	1.00
21-hydroxypregnenolone disulfate	Lipid	Pregnenolone Steroids	1.07	1.14	0.98	0.67	1.00	1.00	1.08	1.03	0.48	0.59	0.94	1.00	1.13	1.13	1.00	0.56	1.00	1.00
2-aminoadipate	Amino Acid	Lysine Metabolism	10.13	8.98	0.91	0.20	1.00	1.00	10.50	10.88	0.88	0.09	1.00	1.00	10.35	8.30	0.94	0.33	0.99	1.00
2-aminoheptanoate	Lipid	Fatty Acid, Amino	9.40	29.89	0.01	1.55	1.00	1.00	11.26	25.01	0.31	1.09	0.88	1.00	7.22	31.10	0.26	2.00	0.99	1.00
2-arachidonoylglycerol (20:4)	Lipid	Monoacylglycerol	1.32	10.89	0.70	0.81	1.00	1.00	1.34	16.78	0.35	0.85	0.89	1.00	1.43	17.02	0.06	0.89	0.99	1.00
2'-deoxyuridine	Nucleotide	Pyrimidine Metabolism, Uracil	4.87	2.71	0.86	1.27	1.00	1.00	4.69	2.17	0.55	1.36	0.97	1.00	6.10	2.51	0.30	2.08	0.99	1.00
2-docosahexaenoylglycerol (22:6)*	Lipid	Monoacylglycerol	31.47	4.67	0.30	1.64	1.00	1.00	29.41	9.17	0.62	1.30	1.00	1.00	27.21	12.19	0.12	1.06	0.99	1.00
2-hydroxybehenate	Lipid	Fatty Acid, Monohydroxy	0.82	0.88	0.22	0.78	1.00	1.00	0.89	0.97	0.40	0.83	0.89	1.00	0.86	0.96	0.16	1.23	0.99	1.00
2-hydroxyhippurate (salicylurate)	Xenobiotics	Benzoate Metabolism	10.78	2.31	0.09	0.89	1.00	1.00	39.04	12.13	0.06	0.62	0.73	1.00	11.82	7.92	0.51	0.45	0.99	1.00
2-hydroxyphenylacetate	Amino Acid	Phenylalanine Metabolism	45.20	21.69	0.49	0.58	1.00	1.00	46.07	17.41	0.20	0.74	0.81	1.00	38.47	25.65	0.27	0.36	0.99	1.00
2-keto-3-deoxy-gluconate	Xenobiotics	Food Component/Plant	1.02	0.87	0.35	1.15	1.00	1.00	1.11	0.93	0.03	1.70	0.73	1.00	1.01	0.97	0.82	0.42	0.99	1.00
2-linoleoylglycerol (18:2)	Lipid	Monoacylglycerol	1.10	52.51	0.85	0.82	1.00	1.00	1.10	62.92	0.99	0.85	1.00	1.00	1.25	68.07	0.18	0.90	0.99	1.00
2-methylbutyrylcarnitine (C5)	Amino Acid	Leucine, Isoleucine and Valine	1.02	0.80	0.01	2.22	1.00	1.00	1.03	0.91	0.06	1.48	0.73	1.00	1.06	0.91	0.02	1.68	0.86	1.00
2-myristoylglycerol (14:0)	Lipid	Monoacylglycerol	16.15	26.39	0.41	0.72	1.00	1.00	16.36	34.48	0.40	1.09	0.89	1.00	14.99	40.66	0.05	1.59	0.99	1.00
2-naphthol sulfate	Xenobiotics	Chemical	2.24	1.41	0.29	0.96	1.00	1.00	1.39	1.13	0.29	0.70	0.87	1.00	1.99	2.54	0.81	0.50	0.99	1.00
2'-O-methyluridine	Nucleotide	Pyrimidine Metabolism, Uracil	1.91	0.98	0.07	1.32	1.00	1.00	2.00	0.95	0.01	1.43	0.73	1.00	2.14	1.08	0.05	1.46	0.99	1.00
2-oxoarginine*	Amino Acid	Urea cycle; Arginine and Prolin	4.75	2.04	0.17	0.63	1.00	1.00	6.10	3.48	0.09	0.38	0.74	1.00	8.02	4.17	0.14	0.51	0.99	1.00
2-palmitoleoylglycerol (16:1)*	Lipid	Monoacylglycerol	26.13	1.18	0.94	1.43	1.00	1.00	34.72	1.36	0.86	1.38	1.00	1.00	24.17	1.28	0.44	1.68	0.99	1.00
2-palmitoleoyl-GPC* (16:1)*	Lipid	Lysophospholipid	28.34	7.80	0.10	1.32	1.00	1.00	35.36	8.61	0.17	1.60	0.79	1.00	41.88	7.13	0.08	1.50	0.99	1.00
3-(3-amino-3-carboxypropyl)uridine*	Nucleotide	Pyrimidine Metabolism, Uracil	1.04	1.01	0.26	0.76	1.00	1.00	1.06	1.02	0.43	0.64	0.91	1.00	1.10	1.06	0.49	0.64	0.99	1.00
3-(3-hydroxyphenyl)propionate sulfate	Xenobiotics	Benzoate Metabolism	5.12	5.94	0.21	0.26	1.00	1.00	2.45	2.75	0.41	0.20	0.89	1.00	1.39	11.39	0.29	1.48	0.99	1.00
3,4-methyleneheptanoate	Xenobiotics	Food Component/Plant	1.12	5.59	0.04	1.71	1.00	1.00	0.94	6.34	0.49	1.64	0.95	1.00	1.18	4.65	0.12	1.89	0.99	1.00
3,7-dimethylurate	Xenobiotics	Xanthine Metabolism	0.99	1.76	0.96	0.94	1.00	1.00	1.14	2.53	0.38	1.07	0.89	1.00	1.22	4.20	0.97	1.21	0.99	1.00
3b-hydroxy-5-cholenoic acid	Lipid	Secondary Bile Acid Metabolis	1.64	2.31	0.28	0.60	1.00	1.00	1.62	1.57	0.34	0.44	0.88	1.00	1.63	1.40	0.74	0.30	0.99	1.00
3-ethylcatechol sulfate (1)	Xenobiotics	Food Component/Plant	3.14	1.21	0.41	1.02	1.00	1.00	3.56	0.71	0.01	1.46	0.73	1.00	2.73	0.90	0.02	1.16	0.86	1.00
3-hydroxyadipate*	Lipid	Fatty Acid, Dicarboxylate	65.10	18.91	0.32	0.90	1.00	1.00	42.60	15.62	0.34	0.85	0.88	1.00	36.19	20.40	0.06	0.62	0.99	1.00
3-hydroxybutyroylglycine	Lipid	Fatty Acid Metabolism(Acyl Gly	1.07	1.40	0.58	1.03	1.00	1.00	0.99	1.16	0.97	0.82	1.00	1.00	1.19	1.04	0.17	0.87	0.99	1.00
3-hydroxybutyrylcarnitine (1)	Lipid	Fatty Acid Metabolism(Acyl Ca	1.31	2.52	0.58	0.73	1.00	1.00	1.12	3.32	0.86	0.75	1.00	1.00	1.06	3.62	0.34	0.83	0.99	1.00
3-hydroxybutyrylcarnitine (2)	Lipid	Fatty Acid Metabolism(Acyl Ca	1.16	1.00	0.24	1.21	1.00	1.00	1.12	0.98	0.22	1.00	0.84	1.00	1.13	0.90	0.05	1.83	0.99	1.00
3-hydroxyhippurate sulfate	Xenobiotics	Benzoate Metabolism	4.21	4.02	0.24	0.17	1.00	1.00	2.45	3.00	0.58	0.31	0.98	1.00	1.86	8.63	0.34	1.65	0.99	1.00
3-hydroxyisobutyrate	Amino Acid	Leucine, Isoleucine and Valine	0.96	105.31	0.98	0.72	1.00	1.00	1.01	47.66	0.66	0.71	1.00	1.00	0.98	45.06	0.66	0.87	0.99	1.00
3-hydroxymyristate	Lipid	Fatty Acid, Monohydroxy	15.56	1.40	0.53	0.86	1.00	1.00	20.71	1.47	0.27	0.83	0.85	1.00	17.80	1.29	0.41	0.96	0.99	1.00
3-hydroxysebacate	Lipid	Fatty Acid, Monohydroxy	4.96	4.30	0.87	0.15	1.00	1.00	3.30	3.75	0.76	0.27	1.00	1.00	3.72	2.32	0.72	0.61	0.99	1.00
3-hydroxystachydrine*	Xenobiotics	Food Component/Plant	396.49	85.92	0.34	1.03	1.00	1.00	69.56	91.09	0.83	0.34	1.00	1.00	54.46	100.57	0.34	0.48	0.99	1.00
3-methoxytyrosine	Amino Acid	Tyrosine Metabolism	0.97	4.20	0.56	0.76	1.00	1.00	1.04	3.91	0.56	0.70	0.97	1.00	1.10	5.76	0.81	0.88	0.99	1.00
3-methyl catechol sulfate (1)	Xenobiotics	Benzoate Metabolism	1.12	0.79	0.25	0.95	1.00	1.00	1.46	0.66	0.01	2.11	0.73	1.00	1.26	0.84	0.02	1.66	0.86	1.00
3-methyladipate	Lipid	Fatty Acid, Dicarboxylate	3.62	1.13	0.38	0.88	1.00	1.00	3.80	1.02	0.58	0.87	0.98	1.00	6.30	0.99	0.15	1.02	0.99	1.00
3-methylglutarylcarnitine (2)	Amino Acid	Leucine, Isoleucine and Valine	2.53	1.05	0.54	1.00	1.00	1.00	3.38	1.11	0.44	0.91	0.91	1.00	4.66	1.27	0.34	1.03	0.99	1.00
3-methylxanthine	Xenobiotics	Xanthine Metabolism	2.78	10.80	0.67	0.59	1.00	1.00	1.13	10.07	0.77	0.71	1.00	1.00	7.99	7.70	0.75	0.06	0.99	1.00
3-phenylpropionate (hydrocinnamate)	Xenobiotics	Benzoate Metabolism	0.97	1.32	0.23	1.44	1.00	1.00	0.89	1.09	0.34	1.01	0.88	1.00	1.02	1.30	0.19	1.24	0.99	1.00
3-sulfo-L-alanine	Amino Acid	Methionine, Cysteine, SAM an	15.21	27.67	0.58	0.81	1.00	1.00	19.39	25.44	0.24	0.69	0.84	1.00	16.96	27.25	0.27	0.99	0.99	1.00
4-cholesten-3-one	Lipid	Sterol	0.98	11.20	0.44	0.73	1.00	1.00	0.96	9.22	0.55	0.77	0.97	1.00	0.99	11.89	0.85	0.83	0.99	1.00
4-guanidinobutanoate	Amino Acid	Guanidino and Acetamido Me	7.06	5.78	0.95	0.40	1.00	1.00	7.50	4.31	0.49	0.50	0.95	1.00	8.46	8.80	0.92	0.15	0.99	1.00
4-hydroxycoumarin	Xenobiotics	Drug - Cardiovascular	1.66	1.80	0.69	0.36	1.00	1.00	1.88	3.10	0.97	0.65	1.00	1.00	1.33	1.52	0.70	0.57	0.99	1.00
4-hydroxyhippurate	Xenobiotics	Benzoate Metabolism	7.35	0.93	0.97	0.95	1.00	1.00	5.09	1.17	0.27	0.98	0.85	1.00	6.18	1.32	0.63	1.07	0.99	1.00
4-methoxyphenol sulfate	Amino Acid	Tyrosine Metabolism	1.49	2.77	0.03	0.83	1.00	1.00	1.16	1.71	0.10	0.97	0.75	1.00	1.47	2.16	0.35	1.04	0.99	1.00
4-methylguaiacol sulfate	Xenobiotics	Benzoate Metabolism	1.44	1.61	0.32	0.63	1.00	1.00	2.00	1.93	0.39	0.58	0.89	1.00	2.01	2.65	0.23	0.95	0.99	1.00
5-(galactosylhydroxy)-L-lysine	Amino Acid	Lysine Metabolism	1.02	4.04	0.85	1.24	1.00	1.00	3.34	5.32	0.51	0.51	0.95	1.00	2.89	5.13	1.00	0.69	1.00	1.00
5,6-dihydrothymine	Nucleotide	Pyrimidine Metabolism, Thym	1.11	1.05	0.40	0.77	1.00	1.00	1.07	1.06	0.96	0.38	1.00	1.00	1.16	1.05	0.08	1.56	0.99	1.00
5,6-dihydrouracil	Nucleotide	Pyrimidine Metabolism, Uracil	1.38	2.25	0.86	0.87	1.00	1.00	1.32	2.23	0.64	0.88	1.00	1.00	1.29	2.36	0.28	1.14	0.99	1.00
5-acetylamino-6-formylamino-3-methyluracil	Xenobiotics	Xanthine Metabolism	0.91	1.07	0.21	1.07	1.00	1.00	1.10	1.01	0.97	0.49	1.00	1.00	1.22	1.16	0.90	0.42	0.99	1.00
5alpha-androstan-3alpha,17beta-diol monosul	Lipid	Androgenic Steroids	1.29	1.46	0.31	0.49	1.00	1.00	1.45	1.28	0.46	0.38	0.93	1.00	0.93	1.41	0.30	1.27	0.99	1.00

5alpha-androstan-3beta,17alpha-diol disulfate	Lipid	Androgenic Steroids	0.99	1.22	0.99	0.78	1.00	1.00	1.09	1.06	0.84	0.32	1.00	1.00	1.42	1.22	0.53	0.65	0.99	1.00
5alpha-androstan-3beta,17beta-diol disulfate	Lipid	Androgenic Steroids	1.18	1.12	0.87	0.36	1.00	1.00	1.21	1.10	0.97	0.58	1.00	1.00	1.20	1.14	0.44	0.53	0.99	1.00
5alpha-pregnan-3beta,20alpha-diol disulfate	Lipid	Progestin Steroids	3.47	6.11	0.76	0.55	1.00	1.00	3.00	4.81	0.92	0.62	1.00	1.00	4.36	4.03	0.64	0.39	0.99	1.00
5alpha-pregnan-3beta,20alpha-diol monosulfa	Lipid	Progestin Steroids	1.02	0.96	0.44	0.56	1.00	1.00	0.89	1.03	0.25	1.16	0.85	1.00	1.08	0.95	0.10	1.28	0.99	1.00
5alpha-pregnan-3beta,20beta-diol monosulfate	Lipid	Progestin Steroids	1.05	1.61	0.86	1.01	1.00	1.00	0.97	2.15	0.13	1.54	0.76	1.00	1.05	1.26	0.55	1.06	0.99	1.00
5-hydroxyhexanoate	Lipid	Fatty Acid, Monohydroxy	0.92	1.85	0.24	0.82	1.00	1.00	1.02	1.60	0.79	0.62	1.00	1.00	1.02	1.73	0.53	0.81	0.99	1.00
7-methylxanthine	Xenobiotics	Xanthine Metabolism	1.14	1.03	0.77	0.54	1.00	1.00	1.14	1.16	0.74	0.71	1.00	1.00	5.25	1.42	0.95	0.95	0.99	1.00
9,10-DiHOME	Lipid	Fatty Acid, Dihydroxy	0.97	1.49	0.38	0.93	1.00	1.00	0.93	0.92	0.34	0.51	0.88	1.00	0.83	1.11	0.77	0.84	0.99	1.00
adenine	Nucleotide	Purine Metabolism, Adenine c	1.12	5.65	0.46	0.93	1.00	1.00	1.10	5.75	0.74	0.92	1.00	1.00	1.11	6.44	0.51	1.18	0.99	1.00
adipoylcarnitine (C6-DC)	Lipid	Fatty Acid Metabolism(Acyl Ca	1.09	4.37	0.96	0.72	1.00	1.00	1.06	5.24	0.97	0.71	1.00	1.00	1.17	4.65	0.87	0.81	0.99	1.00
adrenoylcarnitine (C22:4)*	Lipid	Fatty Acid Metabolism(Acyl Ca	10.00	1.90	0.28	1.86	1.00	1.00	7.15	3.40	0.91	0.95	1.00	1.00	7.85	4.47	0.83	0.87	0.99	1.00
alpha-ketobutyrate	Amino Acid	Methionine, Cysteine, SAM an	10.31	6.34	0.82	0.43	1.00	1.00	12.57	11.55	0.18	0.38	0.79	1.00	4.85	5.20	0.41	0.11	0.99	1.00
andro steroid monosulfate C19H28O6S (1)*	Lipid	Androgenic Steroids	1.23	1.80	0.22	1.11	1.00	1.00	1.26	1.14	0.03	0.39	0.73	1.00	1.30	1.27	0.22	0.10	0.99	1.00
androstenediol (3alpha, 17alpha) monosulfate	Lipid	Androgenic Steroids	1.01	1.05	0.66	0.44	1.00	1.00	1.01	1.06	0.87	0.66	1.00	1.00	1.01	1.01	0.73	0.33	0.99	1.00
androstenediol (3beta,17beta) monosulfate (2)	Lipid	Androgenic Steroids	1.15	1.02	0.48	1.01	1.00	1.00	1.19	0.99	0.24	1.38	0.84	1.00	1.18	1.13	0.69	0.76	0.99	1.00
arabinose	Carbohydrat	Pentose Metabolism	42.60	14.55	0.00	1.37	0.47	0.62	76.77	7.20	0.02	2.04	0.73	1.00	73.76	13.77	0.00	2.50	0.86	1.00
arachidoylcarnitine (C20)*	Lipid	Fatty Acid Metabolism(Acyl Ca	0.98	6.58	0.71	0.73	1.00	1.00	1.01	4.79	0.74	0.75	1.00	1.00	1.07	5.42	0.25	0.81	0.99	1.00
behenoylcarnitine (C22)*	Lipid	Fatty Acid Metabolism(Acyl Ca	1.08	0.97	0.29	0.85	1.00	1.00	0.94	1.08	0.39	1.13	0.89	1.00	1.08	0.97	0.08	0.91	0.99	1.00
benzoate	Xenobiotics	Benzoate Metabolism	1.02	0.90	0.48	0.76	1.00	1.00	1.11	0.96	0.22	0.85	0.83	1.00	0.91	0.97	0.89	0.46	0.99	1.00
benzoylcarnitine*	Xenobiotics	Chemical	1.28	3.91	0.24	0.88	1.00	1.00	1.07	3.57	0.83	1.01	1.00	1.00	1.19	7.33	0.43	1.08	0.99	1.00
caproate (6:0)	Lipid	Medium Chain Fatty Acid	12.10	16.14	0.83	0.32	1.00	1.00	12.74	26.45	0.79	0.71	1.00	1.00	10.83	23.57	0.67	0.87	0.99	1.00
caprylate (8:0)	Lipid	Medium Chain Fatty Acid	0.98	22.36	0.14	0.73	1.00	1.00	1.11	12.30	0.25	0.74	0.85	1.00	1.07	19.34	0.03	0.89	0.86	1.00
carboxyethyl-GABA	Amino Acid	Glutamate Metabolism	1.03	3.17	0.82	0.74	1.00	1.00	0.94	2.60	0.80	0.75	1.00	1.00	0.91	3.66	0.41	0.92	0.99	1.00
carotene diol (3)	Cofactors ar	Vitamin A Metabolism	3.53	0.91	0.12	0.93	1.00	1.00	3.84	0.97	0.24	0.90	0.85	1.00	4.41	1.04	0.45	1.02	0.99	1.00
ceramide (d18:1/17:0, d17:1/18:0)*	Lipid	Ceramides	1.18	1.01	0.23	1.20	1.00	1.00	1.08	0.91	0.30	1.23	0.88	1.00	1.06	0.97	0.52	1.10	0.99	1.00
phosphocholine	Lipid	Phospholipid Metabolism	1.04	0.96	0.36	1.23	1.00	1.00	1.03	0.96	0.45	0.91	0.92	1.00	1.05	1.00	0.23	0.67	0.99	1.00
corticosterone	Lipid	Corticosteroids	4.34	3.71	0.83	0.45	1.00	1.00	5.40	4.13	0.66	0.56	1.00	1.00	6.89	5.75	0.61	0.54	0.99	1.00
cortisone	Lipid	Corticosteroids	1.17	1.16	0.91	0.16	1.00	1.00	1.15	1.17	0.85	0.34	1.00	1.00	1.17	1.24	0.98	0.85	0.99	1.00
cysteine s-sulfate	Amino Acid	Methionine, Cysteine, SAM an	0.78	1.04	0.03	1.84	1.00	1.00	0.76	1.00	0.23	1.29	0.84	1.00	0.60	0.88	0.00	2.39	0.86	1.00
cysteine sulfinic acid	Amino Acid	Methionine, Cysteine, SAM an	2.91	1.91	0.57	0.77	1.00	1.00	2.90	2.05	0.41	0.94	0.89	1.00	3.77	2.40	0.63	0.59	0.99	1.00
cysteinylglycine	Amino Acid	Glutathione Metabolism	5.66	9.72	0.36	0.70	1.00	1.00	5.64	8.46	0.91	0.55	1.00	1.00	6.73	11.81	0.71	0.72	0.99	1.00
cytidine	Nucleotide	Pyrimidine Metabolism, Cytid	1.76	1.10	0.36	1.51	1.00	1.00	2.82	1.26	0.96	1.09	1.00	1.00	2.07	1.48	0.52	0.88	0.99	1.00
deoxycholate	Lipid	Secondary Bile Acid Metabolis	20.70	37.77	0.58	0.34	1.00	1.00	16.68	25.93	0.93	0.24	1.00	1.00	19.88	22.64	0.10	0.23	0.99	1.00
diacylglycerol (12:0/18:1, 14:0/16:1, 16:0/14	Lipid	Diacylglycerol	3.19	0.94	0.13	1.03	1.00	1.00	6.45	1.10	0.53	0.89	0.96	1.00	4.97	1.18	0.46	1.13	0.99	1.00
dihomo-linolenoylcarnitine (C20:3n3 or 6)*	Lipid	Fatty Acid Metabolism(Acyl Ca	1.01	11.37	0.22	0.85	1.00	1.00	1.15	13.08	0.16	0.86	0.79	1.00	1.31	6.16	0.68	0.88	0.99	1.00
dihomo-linoleoylcarnitine (C20:2)*	Lipid	Fatty Acid Metabolism(Acyl Ca	1.12	1.12	0.85	0.26	1.00	1.00	1.10	1.14	0.56	0.49	0.97	1.00	1.08	1.13	0.61	0.53	0.99	1.00
dihydrocaffeate sulfate (2)	Xenobiotics	Food Component/Plant	3.78	9.29	0.89	0.53	1.00	1.00	0.67	3.10	0.25	0.75	0.85	1.00	1.01	5.19	0.61	0.98	0.99	1.00
docosapentaenoate (n6 DPA; 22:5n6)	Lipid	Polyunsaturated Fatty Acid (n3	294.93	108.15	0.39	1.09	1.00	1.00	416.76	180.97	0.10	1.05	0.75	1.00	444.36	190.19	0.62	1.03	0.99	1.00
docosapentaenoylcarnitine (C22:5n3)*	Lipid	Fatty Acid Metabolism(Acyl Ca	13.18	2.12	0.71	2.03	1.00	1.00	13.64	2.23	0.92	2.10	1.00	1.00	14.57	2.05	0.79	2.43	0.99	1.00
ectoine	Xenobiotics	Chemical	14.64	10.61	0.98	0.42	1.00	1.00	39.55	12.86	0.35	0.76	0.89	1.00	5.26	11.50	0.05	1.34	0.99	1.00
eicosapentaenoylcholine	Lipid	Fatty Acid Metabolism (Acyl Cl	3.91	1.26	0.23	0.88	1.00	1.00	3.40	1.45	0.85	0.88	1.00	1.00	3.37	1.16	0.10	1.12	0.99	1.00
eicosenedioate (C20:1-DC)*	Lipid	Fatty Acid, Dicarboxylate	1.03	0.95	0.33	0.81	1.00	1.00	1.05	0.96	0.15	0.61	0.78	1.00	1.13	1.08	0.51	0.46	0.99	1.00
eicosenoylcarnitine (C20:1)*	Lipid	Fatty Acid Metabolism(Acyl Ca	1.07	4.19	0.93	0.88	1.00	1.00	1.00	10.37	0.34	1.09	0.88	1.00	1.07	7.88	0.75	1.15	0.99	1.00
epiandrosterone sulfate	Lipid	Androgenic Steroids	1.07	1.09	0.66	0.22	1.00	1.00	1.09	1.15	0.63	0.30	1.00	1.00	1.06	0.99	0.70	0.66	0.99	1.00
ADSGEGDFXAEGGGVR*	Peptide	Fibrinogen Cleavage Peptide	5.64	2.54	0.61	1.02	1.00	1.00	6.04	1.97	0.08	1.49	0.73	1.00	6.55	2.89	0.02	1.32	0.86	1.00
ADpSGEGDFXAEGGGVR*	Peptide	Fibrinogen Cleavage Peptide	1.50	1.24	0.24	0.51	1.00	1.00	1.48	1.07	0.85	0.71	1.00	1.00	1.29	1.11	0.91	0.51	0.99	1.00
Fibrinopeptide B (1-11)	Peptide	Fibrinogen Cleavage Peptide	0.97	1.07	0.35	1.47	1.00	1.00	1.07	0.96	0.16	0.99	0.79	1.00	1.05	1.45	0.79	0.82	0.99	1.00
Fibrinopeptide B (1-9)	Peptide	Fibrinogen Cleavage Peptide	15.84	3.86	0.80	1.55	1.00	1.00	22.45	4.92	0.57	1.49	0.97	1.00	21.66	4.25	0.88	1.92	0.99	1.00
fumarate	Energy	TCA Cycle	0.97	1.04	0.76	1.02	1.00	1.00	1.00	0.95	0.41	0.60	0.89	1.00	1.04	1.08	0.71	0.90	0.99	1.00
galactonate	Carbohydrat	Fructose, Mannose and Galact	36.31	16.42	0.32	0.47	1.00	1.00	29.35	0.70	0.26	0.90	0.85	1.00	21.45	18.71	0.56	0.10	0.99	1.00
gamma-glutamyl-2-aminobutyrate	Peptide	Gamma-glutamyl Amino Acid	4.48	21.55	0.18	1.69	1.00	1.00	6.65	18.49	0.26	1.17	0.85	1.00	6.13	12.87	0.54	1.05	0.99	1.00
gamma-glutamylserine	Peptide	Gamma-glutamyl Amino Acid	1.12	11.39	0.13	1.03	1.00	1.00	1.09	11.62	0.34	0.89	0.88	1.00	1.12	12.05	0.21	1.17	0.99	1.00
gamma-glutamyltryptophan	Peptide	Gamma-glutamyl Amino Acid	1.08	1.25	0.01	0.54	1.00	1.00	1.13	1.25	0.06	0.44	0.73	1.00	1.17	1.27	0.01	0.32	0.86	1.00
gamma-glutamyltyrosine	Peptide	Gamma-glutamyl Amino Acid	1.07	0.93	0.17	1.15	1.00	1.00	1.14	0.95	0.21	1.23	0.81	1.00	1.16	0.92	0.02	2.05	0.86	1.00

gentisate	Amino Acid	Tyrosine Metabolism	1.41	0.86	0.08	1.47	1.00	1.00	1.54	0.84	0.06	1.96	0.73	1.00	1.13	1.01	0.96	0.62	0.99	1.00
glucuronide of piperine metabolite C17H21NO	Xenobiotics	Food Component/Plant	5.00	2.54	0.96	0.94	1.00	1.00	4.51	2.34	0.97	1.02	1.00	1.00	3.39	3.28	0.57	0.08	0.99	1.00
glucuronide of piperine metabolite C17H21NO	Xenobiotics	Food Component/Plant	8.44	1.56	0.84	1.09	1.00	1.00	8.08	1.58	0.76	1.18	1.00	1.00	3.70	1.77	0.60	1.09	0.99	1.00
glucuronide of piperine metabolite C17H21NO	Xenobiotics	Food Component/Plant	5.00	5.43	0.92	0.26	1.00	1.00	3.85	3.86	0.64	0.08	1.00	1.00	4.74	5.96	0.56	0.29	0.99	1.00
glutarate (C5-DC)	Lipid	Fatty Acid, Dicarboxylate	1.18	6.96	0.38	0.86	1.00	1.00	1.19	6.58	0.72	0.86	1.00	1.00	1.22	4.87	0.73	0.83	0.99	1.00
glutaroylcarnitine (C5)	Amino Acid	Lysine Metabolism	1.08	7.16	0.54	0.72	1.00	1.00	1.07	6.75	0.32	0.75	0.88	1.00	1.11	6.58	0.73	0.82	0.99	1.00
glycerol 3-phosphate	Lipid	Glycerolipid Metabolism	1.08	2.15	0.81	0.74	1.00	1.00	1.10	2.53	0.56	0.76	0.97	1.00	1.00	2.78	0.29	0.93	0.99	1.00
glycerophosphoethanolamine	Lipid	Phospholipid Metabolism	1.06	1.00	0.73	0.86	1.00	1.00	1.01	0.99	0.79	1.33	1.00	1.00	1.06	1.08	0.79	1.18	0.99	1.00
glycerophosphoinositol*	Lipid	Phospholipid Metabolism	46.96	50.86	0.52	0.21	1.00	1.00	39.00	71.93	0.61	0.71	1.00	1.00	101.38	82.78	0.99	0.51	1.00	1.00
glycine conjugate of C10H14O2 (1)*	Partially Cha	Partially Characterized Molecul	0.97	1.22	0.07	1.34	1.00	1.00	0.97	1.06	0.92	0.98	1.00	1.00	1.17	1.21	0.85	0.23	0.99	1.00
glyco-beta-muricholate	Lipid	Primary Bile Acid Metabolism	1.71	3.18	0.71	0.93	1.00	1.00	2.03	4.48	0.30	0.75	0.88	1.00	2.07	2.41	0.10	0.40	0.99	1.00
glycochenodeoxycholate 3-sulfate	Lipid	Primary Bile Acid Metabolism	3.43	1.43	0.51	0.80	1.00	1.00	4.63	1.76	0.82	0.70	1.00	1.00	3.02	2.63	1.00	0.20	1.00	1.00
glycochenodeoxycholate glucuronide (1)	Lipid	Primary Bile Acid Metabolism	1.52	0.93	0.81	0.80	1.00	1.00	1.84	0.91	0.44	0.94	0.91	1.00	2.11	1.12	0.25	0.93	0.99	1.00
glycodeoxycholate	Lipid	Secondary Bile Acid Metabolis	143.11	41.69	0.55	0.96	1.00	1.00	138.96	50.71	0.69	0.61	1.00	1.00	160.92	34.42	0.47	1.39	0.99	1.00
glycodeoxycholate 3-sulfate	Lipid	Secondary Bile Acid Metabolis	0.83	1.90	0.22	1.33	1.00	1.00	1.07	3.41	0.65	1.56	1.00	1.00	1.22	3.79	0.72	1.12	0.99	1.00
glycosyl ceramide (d18:1/23:1, d17:1/24:1)*	Lipid	Hexosylceramides (HCER)	3.25	23.92	0.83	1.52	1.00	1.00	1.63	16.94	0.12	1.31	0.76	1.00	5.67	22.18	0.37	1.37	0.99	1.00
glycosyl-N-(2-hydroxynervonoyl)-sphingosine (c	Lipid	Hexosylceramides (HCER)	1.19	1.09	0.76	0.80	1.00	1.00	0.96	0.85	0.48	0.86	0.94	1.00	1.12	0.97	0.26	1.36	0.99	1.00
glycosyl-N-behenoyl-sphingadienine (d18:2/22	Lipid	Hexosylceramides (HCER)	1.05	0.97	0.34	0.78	1.00	1.00	1.00	1.01	0.42	0.28	0.90	1.00	1.08	0.99	0.38	1.04	0.99	1.00
glycosyl-N-tricosanoyl-sphingadienine (d18:2/:	Lipid	Hexosylceramides (HCER)	13.83	1.12	0.55	0.85	1.00	1.00	14.97	1.10	0.54	0.86	0.97	1.00	15.92	1.08	0.10	0.98	0.99	1.00
glycoursodeoxycholate	Lipid	Secondary Bile Acid Metabolis	0.99	0.93	0.68	0.36	1.00	1.00	2.32	1.22	0.25	1.13	0.85	1.00	1.10	1.01	0.94	0.77	0.99	1.00
guanidinosuccinate	Amino Acid	Guanidino and Acetamido Me	1.05	1.01	0.48	0.64	1.00	1.00	1.01	0.98	0.39	0.46	0.89	1.00	1.01	1.01	0.71	0.38	0.99	1.00
gulonate*	Cofactors ar	Ascorbate and Aldarate Metabo	0.97	45.99	0.83	0.82	1.00	1.00	1.06	52.54	0.18	0.85	0.79	1.00	1.01	41.60	0.31	0.91	0.99	1.00
heneicosapentaenoate (21:5n3)	Lipid	Polyunsaturated Fatty Acid (n3	1.43	1.60	0.99	0.83	1.00	1.00	1.18	1.33	0.42	0.71	0.90	1.00	1.15	1.19	0.61	0.82	0.99	1.00
hexanoylglycine (C6)	Lipid	Fatty Acid Metabolism(Acyl Gly	1.46	2.52	0.94	0.95	1.00	1.00	1.16	2.06	0.89	1.41	1.00	1.00	1.40	2.34	0.83	1.19	0.99	1.00
homocitrulline	Amino Acid	Urea cycle; Arginine and Prolin	1.25	1.01	0.19	1.32	1.00	1.00	1.24	1.09	0.07	0.59	0.73	1.00	1.29	1.38	0.14	0.33	0.99	1.00
homovanillate (HVA)	Amino Acid	Tyrosine Metabolism	1.79	2.13	0.29	0.62	1.00	1.00	1.74	1.58	0.16	0.32	0.79	1.00	1.37	2.08	0.83	0.93	0.99	1.00
hydantoin-5-propionate	Amino Acid	Histidine Metabolism	1.06	1.13	0.85	0.49	1.00	1.00	1.02	1.08	0.83	0.97	1.00	1.00	1.12	1.02	0.69	0.66	0.99	1.00
hydroquinone sulfate	Xenobiotics	Drug - Topical Agents	1.47	1.02	0.59	0.93	1.00	1.00	3.84	1.10	0.83	0.90	1.00	1.00	1.78	1.47	0.32	0.57	0.99	1.00
hyocholate	Lipid	Secondary Bile Acid Metabolis	4.03	2.13	0.26	1.00	1.00	1.00	5.15	2.97	0.12	0.59	0.76	1.00	4.33	3.23	0.69	0.50	0.99	1.00
imidazole propionate	Amino Acid	Histidine Metabolism	2.62	4.37	0.61	0.74	1.00	1.00	37.85	5.41	0.83	0.87	1.00	1.00	3.86	5.85	0.76	0.58	0.99	1.00
indole-3-carboxylate	Amino Acid	Tryptophan Metabolism	0.93	1.67	0.12	1.27	1.00	1.00	0.95	1.76	0.02	1.32	0.73	1.00	1.06	2.19	0.22	1.31	0.99	1.00
indoleacetylcarnitine*	Xenobiotics	Chemical	2.70	1.36	0.50	1.11	1.00	1.00	3.44	2.46	0.74	0.40	1.00	1.00	2.88	1.06	0.36	1.32	0.99	1.00
indoleacetylglutamine	Amino Acid	Tryptophan Metabolism	1.75	1.11	0.89	0.94	1.00	1.00	3.04	1.59	0.64	1.10	1.00	1.00	2.25	1.80	0.19	0.75	0.99	1.00
isobutyrylglycine (C4)	Amino Acid	Leucine, Isoleucine and Valine	1.02	0.82	0.15	1.66	1.00	1.00	1.21	0.98	0.16	1.22	0.79	1.00	1.22	0.98	0.14	1.70	0.99	1.00
isocitrate	Energy	TCA Cycle	1.07	0.99	0.52	0.83	1.00	1.00	1.02	0.98	0.85	0.47	1.00	1.00	1.05	1.02	0.80	0.38	0.99	1.00
isoleucylglycine	Peptide	Dipeptide	3.89	2.24	0.99	0.92	1.00	1.00	5.14	2.85	0.38	0.95	0.89	1.00	4.85	3.08	0.26	0.86	0.99	1.00
isoursodeoxycholate	Lipid	Secondary Bile Acid Metabolis	1.22	1.75	0.90	0.68	1.00	1.00	1.17	1.58	0.26	0.54	0.85	1.00	1.25	1.59	0.41	0.49	0.99	1.00
isovalerate (C5)	Amino Acid	Leucine, Isoleucine and Valine	7.37	30.52	0.77	1.34	1.00	1.00	34.72	26.22	0.61	0.47	1.00	1.00	8.02	36.59	0.26	1.64	0.99	1.00
isovalerylglycine	Amino Acid	Leucine, Isoleucine and Valine	1.41	0.77	0.09	1.06	1.00	1.00	3.09	0.90	0.11	1.18	0.76	1.00	1.82	0.96	0.04	1.51	0.99	1.00
lactosyl-N-behenoyl-sphingosine (d18:1/22:0)	Lipid	Lactosylceramides (LCER)	19.61	28.41	0.44	0.65	1.00	1.00	12.95	24.38	0.97	0.76	1.00	1.00	14.96	23.49	0.71	0.78	0.99	1.00
leucylalanine	Peptide	Dipeptide	0.85	1.14	0.14	1.46	1.00	1.00	1.48	1.08	0.34	0.88	0.88	1.00	0.97	1.00	0.59	0.16	0.99	1.00
linoleoyl ethanolamide	Lipid	Endocannabinoid	2.04	1.54	0.34	0.54	1.00	1.00	2.37	2.75	0.67	0.32	1.00	1.00	2.10	2.29	0.98	0.51	0.99	1.00
linoleoyl-arachidonoyl-glycerol (18:2/20:4) [1]	Lipid	Diacylglycerol	34.01	61.94	0.80	0.57	1.00	1.00	59.89	38.68	0.57	0.43	0.97	1.00	33.67	98.54	0.44	1.06	0.99	1.00
linoleoyl-docosahexaenoyl-glycerol (18:2/22:6	Lipid	Diacylglycerol	75.59	27.10	0.90	0.94	1.00	1.00	69.65	23.58	0.25	0.85	0.85	1.00	55.66	24.75	0.52	0.97	0.99	1.00
lithocholate sulfate (1)	Lipid	Secondary Bile Acid Metabolis	1.42	3.76	0.08	1.47	1.00	1.00	1.10	2.18	0.34	1.06	0.88	1.00	1.23	3.67	0.89	1.35	0.99	1.00
maleate	Lipid	Fatty Acid, Dicarboxylate	9.76	0.71	0.47	0.86	1.00	1.00	11.18	0.73	0.11	0.92	0.76	1.00	15.47	0.86	0.36	1.02	0.99	1.00
malonate	Lipid	Fatty Acid Synthesis	1.22	3.65	0.21	1.51	1.00	1.00	1.30	3.53	0.27	1.40	0.85	1.00	1.37	3.98	0.29	1.60	0.99	1.00
methyl-4-hydroxybenzoate sulfate	Xenobiotics	Benzoate Metabolism	1.80	3.97	0.77	0.61	1.00	1.00	2.43	3.24	0.99	0.35	1.00	1.00	4.06	3.83	0.96	0.35	0.99	1.00
methylmalonate (MMA)	Lipid	Fatty Acid Metabolism (also B(	71.84	82.18	0.30	0.27	1.00	1.00	104.18	80.99	0.13	0.22	0.76	1.00	77.65	79.09	0.30	0.09	0.99	1.00
N-(2-furoyl)glycine	Xenobiotics	Food Component/Plant	2.69	0.71	0.16	1.41	1.00	1.00	8.08	0.64	0.02	1.53	0.73	1.00	3.48	2.95	0.01	0.24	0.86	1.00
N2,N5-diacetylornithine	Amino Acid	Urea cycle; Arginine and Prolin	1.31	1.41	1.00	0.31	1.00	1.00	1.44	1.55	0.76	0.54	1.00	1.00	1.20	1.89	0.21	1.00	0.99	1.00
N2-acetyl,N6-methyllysine	Amino Acid	Lysine Metabolism	1.96	2.77	0.77	0.33	1.00	1.00	2.30	2.73	0.32	0.21	0.88	1.00	2.22	3.17	0.86	0.38	0.99	1.00
N4-acetylcytidine	Nucleotide	Pyrimidine Metabolism, Cytid	2.09	11.40	0.87	1.53	1.00	1.00	5.04	10.55	0.58	0.89	0.98	1.00	2.78	10.79	0.72	1.56	0.99	1.00
N6-succinyladenosine	Nucleotide	Purine Metabolism, Adenine c	1.10	1.73	0.32	0.88	1.00	1.00	1.04	1.63	0.71	0.93	1.00	1.00	0.94	1.92	0.44	1.29	0.99	1.00

N-acetyl-1-methylhistidine*	Amino Acid	Histidine Metabolism	2.81	9.17	0.43	1.22	1.00	1.00	6.74	14.94	0.53	0.88	0.96	1.00	7.86	19.40	0.92	1.13	0.99	1.00
N-acetylaspartate (NAA)	Amino Acid	Alanine and Aspartate Metabol	308.43	212.72	0.38	0.38	1.00	1.00	502.56	173.64	0.81	1.07	1.00	1.00	449.73	172.21	0.97	1.17	0.99	1.00
N-acetylcarnosine	Amino Acid	Histidine Metabolism	9.44	5.59	0.48	0.62	1.00	1.00	13.00	4.46	0.92	1.00	1.00	1.00	7.01	6.07	0.56	0.30	0.99	1.00
N-acetylcitrulline	Amino Acid	Urea cycle; Arginine and Prolin	1.86	1.10	0.44	0.89	1.00	1.00	2.28	1.22	0.12	1.09	0.76	1.00	2.20	1.18	0.31	1.29	0.99	1.00
N-acetylglucosamine/N-acetylgalactosamine	Carbohydrat	Aminosugar Metabolism	11.91	27.00	0.21	0.60	1.00	1.00	11.47	30.38	0.87	0.70	1.00	1.00	12.19	32.85	0.50	0.84	0.99	1.00
N-acetylhistidine	Amino Acid	Histidine Metabolism	1.86	25.07	0.07	1.97	1.00	1.00	2.06	20.26	0.61	1.68	1.00	1.00	3.21	16.38	0.53	1.53	0.99	1.00
N-acetylisoleucine	Amino Acid	Leucine, Isoleucine and Valine	1.68	0.96	0.66	0.87	1.00	1.00	1.66	0.93	0.36	1.01	0.89	1.00	1.11	0.96	0.27	1.45	0.99	1.00
N-acetylneuraminate	Carbohydrat	Aminosugar Metabolism	6.32	1.00	0.08	0.89	1.00	1.00	4.36	0.98	0.00	0.93	0.73	1.00	2.42	1.02	0.96	0.98	0.99	1.00
N-acetylphenylalanine	Amino Acid	Phenylalanine Metabolism	0.95	1.55	0.47	0.86	1.00	1.00	1.02	1.43	0.88	0.76	1.00	1.00	1.05	1.64	0.75	0.91	0.99	1.00
N-acetyltyrosine	Amino Acid	Tyrosine Metabolism	0.98	1.00	0.81	1.01	1.00	1.00	1.09	1.03	0.38	0.85	0.89	1.00	1.19	1.08	0.06	1.00	0.99	1.00
N-carbamoylvaline	Amino Acid	Leucine, Isoleucine and Valine	1.81	1.45	0.31	0.33	1.00	1.00	1.61	1.28	0.80	0.39	1.00	1.00	1.49	1.12	0.62	0.66	0.99	1.00
nervonoylcarnitine (C24:1)*	Lipid	Fatty Acid Metabolism(Acyl Ca	0.99	3.69	0.93	0.75	1.00	1.00	0.90	5.14	0.23	0.76	0.84	1.00	1.02	6.15	0.48	0.85	0.99	1.00
N-formylanthranilic acid	Amino Acid	Tryptophan Metabolism	4.36	1.12	0.67	0.86	1.00	1.00	5.57	0.88	0.22	0.98	0.83	1.00	4.75	0.98	0.30	0.98	0.99	1.00
nicotinamide	Cofactors ar	Nicotinate and Nicotinamide I	1.13	1.41	0.05	1.42	1.00	1.00	1.14	1.24	0.25	0.44	0.85	1.00	1.30	1.53	0.20	0.78	0.99	1.00
nisinate (24:6n3)	Lipid	Polyunsaturated Fatty Acid (n3	12.35	19.84	0.82	0.43	1.00	1.00	18.39	27.15	0.44	0.38	0.91	1.00	15.81	29.22	0.79	0.55	0.99	1.00
N-palmitoylserine	Lipid	Endocannabinoid	1.88	7.87	0.18	1.18	1.00	1.00	5.73	5.34	0.48	0.11	0.94	1.00	11.97	5.30	0.63	0.86	0.99	1.00
N-stearoylserine*	Lipid	Endocannabinoid	0.85	1.08	0.14	1.14	1.00	1.00	1.07	1.06	0.88	0.10	1.00	1.00	1.00	1.11	0.21	0.79	0.99	1.00
N-stearoyltaurine	Lipid	Endocannabinoid	1.26	1.04	0.92	0.87	1.00	1.00	1.65	0.94	0.49	1.14	0.95	1.00	1.65	1.02	0.52	1.03	0.99	1.00
o-cresol sulfate	Xenobiotics	Benzoate Metabolism	3.25	1.25	0.71	1.01	1.00	1.00	3.02	0.73	0.16	1.47	0.79	1.00	2.58	2.32	0.27	0.27	0.99	1.00
oleoyl-arachidonoyl-glycerol (18:1/20:4) [1]*	Lipid	Diacylglycerol	1.11	1.03	0.85	0.75	1.00	1.00	1.04	1.05	0.92	0.14	1.00	1.00	0.98	1.08	0.62	0.89	0.99	1.00
oleoyl-arachidonoyl-glycerol (18:1/20:4) [2]*	Lipid	Diacylglycerol	1.07	1.00	0.95	0.56	1.00	1.00	0.95	1.01	0.38	0.56	0.89	1.00	0.93	1.04	0.40	1.06	0.99	1.00
oleoyl-linolenoyl-glycerol (18:1/18:3) [2]*	Lipid	Diacylglycerol	96.20	135.08	0.34	0.34	1.00	1.00	58.21	83.78	0.03	0.41	0.73	1.00	58.40	93.82	0.09	0.52	0.99	1.00
palmitoleoyl-oleoyl-glycerol (16:1/18:1) [2]*	Lipid	Diacylglycerol	272.30	294.60	0.27	0.15	1.00	1.00	246.03	319.33	0.84	0.31	1.00	1.00	163.89	329.32	0.18	0.83	0.99	1.00
palmitoyl-arachidonoyl-glycerol (16:0/20:4) [1	Lipid	Diacylglycerol	1.09	1.07	0.88	0.26	1.00	1.00	0.96	1.37	0.05	1.75	0.73	1.00	1.00	1.29	0.21	1.71	0.99	1.00
palmitoyl-arachidonoyl-glycerol (16:0/20:4) [2	Lipid	Diacylglycerol	0.93	0.98	0.92	0.57	1.00	1.00	0.91	1.13	0.12	1.41	0.76	1.00	0.97	0.98	0.48	0.57	0.99	1.00
palmitoyl-linolenoyl-glycerol (16:0/18:3) [2]*	Lipid	Diacylglycerol	0.94	2.62	0.62	0.71	1.00	1.00	0.88	4.92	0.56	0.77	0.97	1.00	0.93	4.55	0.67	0.82	0.99	1.00
palmitoyl-linoleoyl-glycerol (16:0/18:2) [1]*	Lipid	Diacylglycerol	60.29	72.86	0.38	0.12	1.00	1.00	45.57	108.31	0.53	0.42	0.96	1.00	1.10	225.61	0.66	0.86	0.99	1.00
palmitoyl-linoleoyl-glycerol (16:0/18:2) [2]*	Lipid	Diacylglycerol	277.91	498.01	0.66	0.60	1.00	1.00	286.83	433.35	0.69	0.53	1.00	1.00	320.57	413.39	0.48	0.30	0.99	1.00
palmitoyl-myristoyl-glycerol (16:0/14:0) [2]	Lipid	Diacylglycerol	1.07	0.75	0.05	1.68	1.00	1.00	1.18	0.98	0.18	1.03	0.79	1.00	1.21	0.93	0.48	1.33	0.99	1.00
paraxanthine	Xenobiotics	Xanthine Metabolism	7.86	0.84	0.79	0.90	1.00	1.00	28.61	0.87	0.18	0.85	0.79	1.00	24.31	0.95	0.36	1.00	0.99	1.00
p-cresol glucuronide*	Amino Acid	Tyrosine Metabolism	1.30	1.95	0.15	1.57	1.00	1.00	1.57	2.01	0.37	1.00	0.89	1.00	1.62	2.36	0.65	0.95	0.99	1.00
phenylacetate	Amino Acid	Phenylalanine Metabolism	1.47	1.08	0.60	0.85	1.00	1.00	1.26	1.10	1.00	0.53	1.00	1.00	1.52	1.44	0.23	0.56	0.99	1.00
phenylacetylcarnitine	Peptide	Acetylated Peptides	3.62	3.08	0.86	0.50	1.00	1.00	2.83	4.97	0.61	0.86	1.00	1.00	5.92	4.90	0.72	0.86	0.99	1.00
phenylacetylglutamate	Peptide	Acetylated Peptides	1.08	1.23	0.69	0.95	1.00	1.00	1.26	1.36	0.71	0.70	1.00	1.00	1.41	1.48	0.36	0.72	0.99	1.00
phenylalanylglycine	Peptide	Dipeptide	1.55	1.70	0.51	0.23	1.00	1.00	2.15	1.51	0.46	0.69	0.93	1.00	1.98	1.66	0.79	0.43	0.99	1.00
phosphate	Energy	Oxidative Phosphorylation	1.14	5.98	1.00	1.23	1.00	1.00	1.23	5.97	0.12	1.26	0.76	1.00	1.19	7.83	0.37	1.38	0.99	1.00
picolinate	Amino Acid	Tryptophan Metabolism	0.98	4.86	0.23	1.12	1.00	1.00	1.08	4.16	0.05	1.06	0.73	1.00	0.93	4.32	0.80	1.28	0.99	1.00
piperine	Xenobiotics	Food Component/Plant	4.79	2.07	0.31	0.82	1.00	1.00	2.61	1.97	0.96	0.51	1.00	1.00	1.56	2.77	0.15	1.26	0.99	1.00
pregnanediol-3-glucuronide	Lipid	Progestin Steroids	1.09	1.14	0.91	0.56	1.00	1.00	1.00	1.06	0.48	0.75	0.94	1.00	1.16	1.09	0.23	0.73	0.99	1.00
pregnenolone sulfate	Lipid	Pregnenolone Steroids	0.93	1.17	0.18	1.30	1.00	1.00	0.98	1.11	0.33	0.90	0.88	1.00	1.12	1.22	0.91	0.60	0.99	1.00
pyrraline	Xenobiotics	Food Component/Plant	0.92	1.06	0.38	0.81	1.00	1.00	1.08	0.86	0.71	1.11	1.00	1.00	0.94	1.00	0.53	0.42	0.99	1.00
ribulonate/xylulonate*	Carbohydrat	Pentose Metabolism	142.34	193.66	0.09	0.21	1.00	1.00	191.51	149.16	0.62	0.18	1.00	1.00	95.02	223.16	0.03	0.50	0.86	1.00
S-1-pyrroline-5-carboxylate	Amino Acid	Glutamate Metabolism	5.95	7.91	0.75	0.22	1.00	1.00	5.40	9.70	0.78	0.40	1.00	1.00	7.45	12.90	0.83	0.55	0.99	1.00
S-adenosylhomocysteine (SAH)	Amino Acid	Methionine, Cysteine, SAM an	1.06	1.00	0.95	0.96	1.00	1.00	1.09	0.98	0.17	1.00	0.79	1.00	1.13	1.10	0.61	0.67	0.99	1.00
sarcosine	Amino Acid	Glycine, Serine and Threonine	0.98	0.99	0.73	0.90	1.00	1.00	1.15	0.93	0.09	1.48	0.74	1.00	1.06	1.06	0.75	0.68	0.99	1.00
sphinganine	Lipid	Sphingolipid Synthesis	4.22	0.97	0.42	0.85	1.00	1.00	4.48	1.03	0.95	0.86	1.00	1.00	5.05	0.98	0.72	0.97	0.99	1.00
sphingomyelin (d18:1/25:0, d19:0/24:1, d20	Lipid	Sphingomyelins	1.10	1.00	0.52	0.97	1.00	1.00	0.89	1.02	0.06	1.44	0.73	1.00	1.11	0.96	0.18	1.67	0.99	1.00
stachydrine	Xenobiotics	Food Component/Plant	1.76	1.19	0.65	1.00	1.00	1.00	1.37	2.04	0.90	0.89	1.00	1.00	1.47	1.56	0.63	0.31	0.99	1.00
stearoyl-arachidonoyl-glycerol (18:0/20:4) [2]*	Lipid	Diacylglycerol	1.00	1.13	0.47	1.09	1.00	1.00	0.90	1.14	0.07	1.54	0.73	1.00	1.03	1.05	0.55	1.01	0.99	1.00
stearoylcholine*	Lipid	Fatty Acid Metabolism (Acyl Cl	1.17	1.10	0.20	0.57	1.00	1.00	1.15	1.34	0.59	1.07	0.98	1.00	1.28	1.18	0.39	0.72	0.99	1.00
suberate (C8-DC)	Lipid	Fatty Acid, Dicarboxylate	9.64	11.16	0.83	0.19	1.00	1.00	8.74	10.11	0.45	0.24	0.92	1.00	12.51	11.09	0.91	0.52	0.99	1.00
suberoylcarnitine (C8-DC)	Lipid	Fatty Acid Metabolism(Acyl Ca	5.27	6.54	0.74	0.42	1.00	1.00	4.14	9.68	0.61	0.95	1.00	1.00	6.46	7.86	0.82	0.53	0.99	1.00
succinylcarnitine (C4)	Energy	TCA Cycle	1.12	0.91	0.01	2.17	1.00	1.00	1.08	0.92	0.04	1.70	0.73	1.00	1.06	1.02	0.51	0.98	0.99	1.00
sucrose	Carbohydrat	Disaccharides and Oligosaccha	1.03	1.01	0.83	0.54	1.00	1.00	1.09	1.36	0.27	0.85	0.85	1.00	1.20	1.36	0.72	0.45	0.99	1.00

sulfate of piperine metabolite C16H19NO3 (2)*	Xenobiotics	Food Component/Plant	6.95	1.76	0.46	1.04	1.00	1.00	4.85	1.79	0.99	1.14	1.00	1.00	2.27	2.01	0.45	0.28	0.99	1.00
sulfate of piperine metabolite C16H19NO3 (3)*	Xenobiotics	Food Component/Plant	3.05	2.14	0.42	0.57	1.00	1.00	2.38	2.36	0.98	0.36	1.00	1.00	1.38	3.62	0.46	1.38	0.99	1.00
tartarate	Xenobiotics	Food Component/Plant	30.03	8.51	0.36	1.20	1.00	1.00	32.76	7.76	0.50	1.21	0.95	1.00	33.44	21.99	0.94	0.61	0.99	1.00
taurodeoxycholate	Lipid	Secondary Bile Acid Metabolis	11.11	2.63	0.32	0.87	1.00	1.00	3.26	1.85	0.89	0.82	1.00	1.00	7.75	1.30	0.38	1.27	0.99	1.00
taurolithocholate 3-sulfate	Lipid	Secondary Bile Acid Metabolis	0.85	1.07	0.52	0.92	1.00	1.00	0.83	0.95	0.82	0.57	1.00	1.00	1.04	0.75	0.22	1.51	0.99	1.00
tetradecadienedioate (C14:2-DC)*	Lipid	Fatty Acid, Dicarboxylate	1.17	1.22	0.95	0.69	1.00	1.00	1.03	1.13	0.93	0.79	1.00	1.00	1.09	1.12	0.98	0.37	0.99	1.00
threonylphenylalanine	Peptide	Dipeptide	1.02	1.12	0.44	0.51	1.00	1.00	1.10	0.96	0.15	0.93	0.79	1.00	1.15	1.38	0.79	0.60	0.99	1.00
thymol sulfate	Xenobiotics	Food Component/Plant	43.14	3.24	0.08	0.94	1.00	1.00	6.43	2.66	0.80	0.86	1.00	1.00	2.86	3.45	0.48	0.49	0.99	1.00
tiglyl carnitine (C5)	Amino Acid	Leucine, Isoleucine and Valine	4.61	0.86	0.06	0.98	1.00	1.00	3.62	0.93	0.12	1.00	0.76	1.00	3.88	0.92	0.19	1.09	0.99	1.00
trans-urocanate	Amino Acid	Histidine Metabolism	1.52	28.32	0.83	1.02	1.00	1.00	1.34	50.22	0.66	0.88	1.00	1.00	0.90	25.43	0.54	1.12	0.99	1.00
tryptophan betaine	Amino Acid	Tryptophan Metabolism	69.46	35.64	0.93	0.61	1.00	1.00	8.69	13.50	0.95	0.26	1.00	1.00	19.11	2.34	0.94	0.94	0.99	1.00
vanillactate	Amino Acid	Tyrosine Metabolism	0.93	1.14	0.77	1.05	1.00	1.00	1.08	1.03	0.07	0.52	0.73	1.00	1.03	1.19	0.79	0.94	0.99	1.00
xanthosine	Nucleotide	Purine Metabolism, (Hypo)Xar	1.21	0.99	0.29	1.18	1.00	1.00	1.22	1.14	0.15	0.64	0.79	1.00	1.21	1.06	0.20	0.93	0.99	1.00
xylose	Carbohydrat	Pentose Metabolism	13.92	12.16	0.35	0.20	1.00	1.00	20.01	11.68	0.91	0.88	1.00	1.00	20.09	23.01	0.38	0.23	0.99	1.00
X - 11299	Unknown	Unknown	3.01	3.24	0.68	0.17	1.00	1.00	2.46	2.11	0.43	0.37	0.91	1.00	2.32	4.19	0.50	0.74	0.99	1.00
X - 11378	Unknown	Unknown	18.60	19.99	0.64	0.08	1.00	1.00	12.85	11.57	0.55	0.08	0.97	1.00	18.65	28.55	0.52	0.43	0.99	1.00
X - 11381	Unknown	Unknown	0.97	0.95	0.74	1.05	1.00	1.00	1.02	0.90	0.05	1.85	0.73	1.00	0.92	0.90	0.37	0.96	0.99	1.00
X - 11407	Unknown	Unknown	4.72	4.20	0.36	0.36	1.00	1.00	7.90	8.28	0.55	0.13	0.97	1.00	16.13	14.49	0.63	0.19	0.99	1.00
X - 11441	Unknown	Unknown	4.93	6.09	0.57	0.41	1.00	1.00	4.39	5.39	0.15	0.36	0.78	1.00	6.69	4.19	0.29	0.60	0.99	1.00
X - 11442	Unknown	Unknown	2.18	4.97	0.99	0.77	1.00	1.00	1.59	6.88	0.37	0.80	0.89	1.00	1.85	3.71	0.69	0.83	0.99	1.00
X - 11478	Unknown	Unknown	16.06	49.88	0.25	0.70	1.00	1.00	8.03	41.42	0.97	0.80	1.00	1.00	13.75	38.80	0.71	0.66	0.99	1.00
X - 11483	Unknown	Unknown	4.54	2.30	0.92	0.98	1.00	1.00	4.70	1.90	0.78	1.08	1.00	1.00	4.56	2.52	0.53	0.99	0.99	1.00
X - 11491	Unknown	Unknown	1.46	1.39	0.35	0.32	1.00	1.00	1.41	1.45	0.31	0.30	0.88	1.00	1.46	1.37	0.53	0.52	0.99	1.00
X - 11522	Unknown	Unknown	1.31	1.26	0.57	0.40	1.00	1.00	1.09	1.11	0.71	0.40	1.00	1.00	1.31	1.14	0.60	0.69	0.99	1.00
X - 11843	Unknown	Unknown	1.49	1.83	0.32	0.58	1.00	1.00	1.38	3.01	0.04	1.03	0.73	1.00	3.45	1.97	0.45	1.13	0.99	1.00
X - 11850	Unknown	Unknown	1.18	1.66	0.05	0.91	1.00	1.00	1.43	1.76	0.12	0.63	0.76	1.00	2.06	1.83	0.41	0.59	0.99	1.00
X - 11852	Unknown	Unknown	33.14	27.27	0.43	0.55	1.00	1.00	19.08	9.10	0.87	0.92	1.00	1.00	146.37	35.80	0.87	1.33	0.99	1.00
X - 11880	Unknown	Unknown	1.10	0.93	0.20	1.18	1.00	1.00	1.13	0.95	0.28	1.12	0.85	1.00	1.14	1.03	0.91	0.80	0.99	1.00
X - 12007	Unknown	Unknown	0.73	1.06	0.98	1.00	1.00	1.00	1.16	1.33	0.27	0.85	0.85	1.00	0.82	1.05	0.98	1.08	0.99	1.00
X - 12013	Unknown	Unknown	2.85	2.02	0.77	0.63	1.00	1.00	1.89	2.14	0.38	0.48	0.89	1.00	7.37	1.93	0.94	1.44	0.99	1.00
X - 12101	Unknown	Unknown	1.15	1.00	0.07	1.12	1.00	1.00	1.16	0.96	0.03	1.45	0.73	1.00	1.28	0.96	0.01	1.72	0.86	1.00
X - 12111	Unknown	Unknown	2.59	1.33	0.56	1.40	1.00	1.00	1.69	2.24	0.60	0.73	0.99	1.00	2.99	2.58	0.92	0.38	0.99	1.00
X - 12117	Unknown	Unknown	3.23	4.20	0.55	0.32	1.00	1.00	6.86	5.07	0.75	0.23	1.00	1.00	6.19	5.80	0.23	0.32	0.99	1.00
X - 12126	Unknown	Unknown	2.05	2.05	0.53	0.54	1.00	1.00	1.71	2.79	0.51	0.87	0.95	1.00	3.39	3.41	0.39	0.82	0.99	1.00
X - 12170	Unknown	Unknown	0.97	0.76	0.01	1.40	1.00	1.00	1.32	0.90	0.05	1.86	0.73	1.00	1.29	1.00	0.32	1.26	0.99	1.00
X - 12212	Unknown	Unknown	1.52	1.20	0.41	0.66	1.00	1.00	1.66	2.23	0.62	0.46	1.00	1.00	1.86	1.82	0.04	0.46	0.99	1.00
X - 12283	Unknown	Unknown	7.60	13.61	0.32	0.66	1.00	1.00	3.46	13.17	0.31	1.42	0.88	1.00	2.30	18.18	0.05	1.81	0.99	1.00
X - 12680	Unknown	Unknown	1.04	1.00	0.92	0.68	1.00	1.00	1.29	1.04	0.14	1.03	0.78	1.00	1.27	0.98	0.39	1.17	0.99	1.00
X - 12707	Unknown	Unknown	1.64	1.22	0.52	0.68	1.00	1.00	2.01	1.05	0.07	1.82	0.73	1.00	2.05	1.22	0.44	1.29	0.99	1.00
X - 12718	Unknown	Unknown	1.10	1.09	0.94	0.54	1.00	1.00	1.18	1.21	0.90	0.52	1.00	1.00	1.31	1.16	0.32	0.97	0.99	1.00
X - 12739	Unknown	Unknown	2.05	1.18	0.14	1.33	1.00	1.00	2.08	1.06	0.27	1.27	0.85	1.00	2.98	1.11	0.02	1.47	0.86	1.00
X - 12798	Unknown	Unknown	0.96	3.96	0.82	0.73	1.00	1.00	1.06	3.54	0.53	0.99	0.96	1.00	0.97	4.74	0.68	1.12	0.99	1.00
X - 12812	Unknown	Unknown	1.86	1.34	0.01	0.65	1.00	1.00	6.69	2.05	0.17	0.69	0.79	1.00	6.72	0.96	0.02	1.18	0.86	1.00
X - 12816	Unknown	Unknown	1.61	16.66	0.26	0.80	1.00	1.00	4.95	5.55	0.98	0.17	1.00	1.00	18.97	5.90	0.82	0.70	0.99	1.00
X - 12822	Unknown	Unknown	2.45	1.19	0.13	0.96	1.00	1.00	2.55	1.21	0.13	0.95	0.76	1.00	3.20	1.20	0.02	1.24	0.86	1.00
X - 12847	Unknown	Unknown	4.12	1.89	0.25	1.18	1.00	1.00	4.47	1.71	0.20	1.47	0.81	1.00	5.69	4.21	0.54	0.50	0.99	1.00
X - 12849	Unknown	Unknown	1.55	1.19	0.09	0.88	1.00	1.00	1.36	4.80	0.13	0.82	0.76	1.00	1.67	1.50	0.10	0.40	0.99	1.00
X - 12851	Unknown	Unknown	3.63	6.04	0.05	0.47	1.00	1.00	2.33	1.52	0.98	0.43	1.00	1.00	1.87	1.08	0.94	1.24	0.99	1.00
X - 13684	Unknown	Unknown	1.08	1.10	0.80	0.60	1.00	1.00	1.05	0.92	0.15	0.95	0.78	1.00	0.99	1.00	0.83	0.29	0.99	1.00
X - 13728	Unknown	Unknown	0.95	1.12	0.62	0.76	1.00	1.00	1.15	1.38	0.73	0.90	1.00	1.00	1.31	1.45	0.52	0.70	0.99	1.00
X - 13737	Unknown	Unknown	1.06	6.18	0.38	1.02	1.00	1.00	1.04	5.81	0.35	1.02	0.89	1.00	1.14	7.37	0.29	1.12	0.99	1.00
X - 13835	Unknown	Unknown	2.80	1.70	0.39	1.09	1.00	1.00	4.53	2.10	0.93	0.88	1.00	1.00	13.36	2.09	0.50	0.96	0.99	1.00
X - 13844	Unknown	Unknown	1.10	0.93	0.23	0.70	1.00	1.00	1.28	0.82	0.01	1.61	0.73	1.00	1.92	0.98	0.11	1.62	0.99	1.00
X - 15461	Unknown	Unknown	6.43	0.97	0.32	0.94	1.00	1.00	6.52	0.98	0.22	0.89	0.83	1.00	7.93	0.98	0.01	1.00	0.86	1.00

X - 15666	Unknown	Unknown	4.73	7.17	0.92	0.54	1.00	1.00	3.71	5.91	0.82	0.72	1.00	1.00	6.84	7.94	0.86	0.61	0.99	1.00
X - 15674	Unknown	Unknown	1.92	2.02	0.41	0.27	1.00	1.00	2.23	2.06	0.95	0.12	1.00	1.00	2.07	1.67	0.30	0.45	0.99	1.00
X - 15728	Unknown	Unknown	5.24	0.96	0.15	1.44	1.00	1.00	4.57	2.39	0.36	0.76	0.89	1.00	1.88	0.93	0.41	1.46	0.99	1.00
X - 16397	Unknown	Unknown	1.17	2.33	0.98	0.74	1.00	1.00	1.09	3.32	0.36	0.72	0.89	1.00	1.16	2.71	0.38	0.81	0.99	1.00
X - 16570	Unknown	Unknown	1.04	1.25	0.23	1.04	1.00	1.00	1.03	1.03	0.63	0.10	1.00	1.00	1.24	1.24	0.61	0.07	0.99	1.00
X - 16654	Unknown	Unknown	1.81	2.80	0.78	0.69	1.00	1.00	2.55	2.39	0.30	0.36	0.88	1.00	3.40	4.01	0.10	0.39	0.99	1.00
X - 16946	Unknown	Unknown	2.86	3.77	0.30	0.66	1.00	1.00	2.07	3.15	0.91	0.73	1.00	1.00	2.15	2.41	0.96	0.56	0.99	1.00
X - 16964	Unknown	Unknown	2.08	1.06	0.12	0.96	1.00	1.00	1.82	0.97	0.08	1.01	0.73	1.00	1.96	1.09	0.26	1.21	0.99	1.00
X - 17010	Unknown	Unknown	0.90	1.02	0.23	0.83	1.00	1.00	1.14	1.13	0.96	0.38	1.00	1.00	1.03	1.01	0.78	0.16	0.99	1.00
X - 17145	Unknown	Unknown	1.70	1.09	0.41	1.05	1.00	1.00	1.54	1.32	0.68	0.56	1.00	1.00	3.38	1.43	0.47	0.92	0.99	1.00
X - 17146	Unknown	Unknown	2.72	10.86	0.05	1.21	1.00	1.00	5.45	29.80	0.32	1.10	0.88	1.00	2.57	45.36	0.89	1.31	0.99	1.00
X - 17185	Unknown	Unknown	1.61	0.80	0.29	0.96	1.00	1.00	2.50	0.86	0.12	1.37	0.76	1.00	9.16	1.07	0.23	1.05	0.99	1.00
X - 17325	Unknown	Unknown	1.19	1.05	0.71	0.79	1.00	1.00	1.33	1.24	0.91	1.08	1.00	1.00	1.33	1.82	0.94	0.85	0.99	1.00
X - 17327	Unknown	Unknown	1.55	20.88	0.46	0.98	1.00	1.00	1.31	15.57	0.44	1.00	0.91	1.00	1.61	7.75	0.29	0.94	0.99	1.00
X - 17351	Unknown	Unknown	1.19	1.49	0.09	0.98	1.00	1.00	0.91	1.20	0.20	1.09	0.81	1.00	1.08	1.83	0.00	2.32	0.86	1.00
X - 17354	Unknown	Unknown	2.58	4.02	0.36	0.55	1.00	1.00	6.32	2.23	0.43	0.77	0.91	1.00	3.05	2.98	0.97	0.31	0.99	1.00
X - 17359	Unknown	Unknown	1.27	5.73	0.34	1.47	1.00	1.00	1.29	6.01	0.23	1.53	0.84	1.00	2.92	4.88	0.26	0.69	0.99	1.00
X - 17367	Unknown	Unknown	1.53	1.80	0.73	0.63	1.00	1.00	1.11	2.72	0.62	1.04	1.00	1.00	1.90	4.03	0.82	0.99	0.99	1.00
X - 17438	Unknown	Unknown	2.51	1.57	0.58	1.08	1.00	1.00	2.18	1.69	0.72	0.63	1.00	1.00	3.57	2.00	0.17	0.81	0.99	1.00
X - 17612	Unknown	Unknown	2.47	4.44	0.37	0.78	1.00	1.00	1.99	2.15	0.78	0.14	1.00	1.00	1.48	1.37	0.37	0.56	0.99	1.00
X - 18345	Unknown	Unknown	1.81	1.19	0.01	0.93	1.00	1.00	2.46	3.10	0.26	0.75	0.85	1.00	3.57	1.84	0.62	1.23	0.99	1.00
X - 18886	Unknown	Unknown	1.25	1.18	0.89	0.20	1.00	1.00	1.12	1.32	0.88	0.47	1.00	1.00	1.20	1.31	0.40	0.36	0.99	1.00
X - 18899	Unknown	Unknown	0.94	0.90	0.40	0.36	1.00	1.00	1.11	0.96	0.13	1.12	0.76	1.00	1.16	1.02	0.71	0.80	0.99	1.00
X - 18901	Unknown	Unknown	4.68	2.26	0.09	1.18	1.00	1.00	3.26	1.74	0.21	1.34	0.81	1.00	3.69	1.05	0.14	1.82	0.99	1.00
X - 21364	Unknown	Unknown	1.09	1.01	0.30	0.87	1.00	1.00	1.06	0.96	0.10	1.17	0.75	1.00	1.10	1.04	0.25	0.85	0.99	1.00
X - 21441	Unknown	Unknown	1.20	2.98	0.48	1.55	1.00	1.00	1.11	2.67	0.08	1.41	0.73	1.00	1.12	2.67	0.98	1.63	0.99	1.00
X - 21442	Unknown	Unknown	1.66	1.25	0.52	0.45	1.00	1.00	6.07	1.07	0.25	0.85	0.85	1.00	25.58	0.98	0.20	1.01	0.99	1.00
X - 21448	Unknown	Unknown	3.18	2.14	0.98	0.59	1.00	1.00	2.70	1.36	0.56	1.17	0.97	1.00	3.15	1.49	0.65	1.43	0.99	1.00
X - 21467	Unknown	Unknown	1.27	1.36	0.53	0.88	1.00	1.00	1.11	1.14	0.52	0.76	0.96	1.00	1.57	1.30	0.89	0.62	0.99	1.00
X - 21470	Unknown	Unknown	1.24	1.86	0.53	0.87	1.00	1.00	1.20	1.62	0.96	0.89	1.00	1.00	1.48	1.73	0.97	0.57	0.99	1.00
X - 21471	Unknown	Unknown	1.04	1.41	0.62	0.78	1.00	1.00	1.05	1.28	0.44	0.81	0.91	1.00	1.04	1.22	0.84	0.82	0.99	1.00
X - 21607	Unknown	Unknown	1.14	1.22	0.45	0.59	1.00	1.00	1.17	1.26	0.87	0.46	1.00	1.00	1.19	1.15	0.93	0.43	0.99	1.00
X - 21729	Unknown	Unknown	6.75	1.23	0.89	1.10	1.00	1.00	3.44	1.14	0.81	0.92	1.00	1.00	6.58	1.18	0.95	1.30	0.99	1.00
X - 21752	Unknown	Unknown	2.78	12.63	0.22	0.71	1.00	1.00	2.56	8.92	0.34	0.80	0.88	1.00	2.26	3.16	0.34	0.58	0.99	1.00
X - 21792	Unknown	Unknown	1.24	1.19	0.39	0.75	1.00	1.00	1.06	1.11	0.96	0.58	1.00	1.00	1.14	1.17	0.69	0.29	0.99	1.00
X - 21821	Unknown	Unknown	1.22	1.50	0.24	0.82	1.00	1.00	1.01	1.40	0.23	1.20	0.84	1.00	1.16	1.97	0.01	2.39	0.86	1.00
X - 22508	Unknown	Unknown	1.97	1.44	0.60	0.67	1.00	1.00	1.73	1.96	0.73	0.44	1.00	1.00	2.32	1.58	0.24	0.91	0.99	1.00
X - 22509	Unknown	Unknown	1.75	3.56	0.09	1.33	1.00	1.00	3.10	2.53	0.04	0.27	0.73	1.00	1.52	2.83	0.19	1.23	0.99	1.00
X - 23196	Unknown	Unknown	0.73	0.83	0.61	0.64	1.00	1.00	0.89	1.05	0.10	0.67	0.75	1.00	1.01	0.73	0.13	1.25	0.99	1.00
X - 23276	Unknown	Unknown	1.06	1.63	0.40	0.78	1.00	1.00	1.16	1.49	0.50	0.57	0.95	1.00	1.12	1.83	0.19	0.74	0.99	1.00
X - 23296	Unknown	Unknown	3.42	3.17	0.76	0.49	1.00	1.00	3.85	3.27	0.84	0.48	1.00	1.00	2.74	3.03	0.38	0.14	0.99	1.00
X - 23297	Unknown	Unknown	1.33	4.27	0.73	0.77	1.00	1.00	1.27	5.19	0.58	0.75	0.98	1.00	1.24	3.74	0.95	0.86	0.99	1.00
X - 23587	Unknown	Unknown	12.00	13.02	0.15	0.08	1.00	1.00	5.92	24.67	0.42	1.01	0.90	1.00	13.13	9.36	0.19	0.31	0.99	1.00
X - 23641	Unknown	Unknown	8.67	6.13	0.31	0.36	1.00	1.00	9.42	6.40	0.84	0.55	1.00	1.00	10.26	6.09	0.90	0.80	0.99	1.00
X - 23644	Unknown	Unknown	470.01	2.49	0.09	0.89	1.00	1.00	5.09	5.35	0.98	0.41	1.00	1.00	#######	3.39	0.91	1.05	0.99	1.00
X - 23659	Unknown	Unknown	1.14	2.73	0.73	0.72	1.00	1.00	1.14	3.16	0.61	0.73	1.00	1.00	1.10	1.10	0.81	0.26	0.99	1.00
X - 23665	Unknown	Unknown	18.63	6.06	0.04	0.76	1.00	1.00	11.71	5.76	0.06	0.57	0.73	1.00	9.44	13.00	0.07	0.30	0.99	1.00
X - 23666	Unknown	Unknown	3.69	0.98	0.13	0.94	1.00	1.00	3.31	0.98	0.18	0.99	0.79	1.00	3.65	1.02	0.29	1.01	0.99	1.00
X - 23739	Unknown	Unknown	1.04	2.82	0.19	0.67	1.00	1.00	1.02	2.70	0.15	0.72	0.79	1.00	1.06	3.44	0.55	0.81	0.99	1.00
X - 23782	Unknown	Unknown	29.01	3.20	0.27	1.38	1.00	1.00	29.57	2.82	0.54	1.55	0.97	1.00	17.49	1.96	0.49	1.80	0.99	1.00
X - 23787	Unknown	Unknown	2.29	1.63	0.87	0.60	1.00	1.00	2.44	1.95	0.44	0.38	0.91	1.00	2.67	1.56	0.92	1.00	0.99	1.00
X - 24243	Unknown	Unknown	27.07	6.54	0.40	0.90	1.00	1.00	13.03	5.42	0.57	0.78	0.97	1.00	10.23	11.14	0.10	0.24	0.99	1.00
X - 24293	Unknown	Unknown	5.43	1.46	0.57	1.13	1.00	1.00	4.13	2.48	0.83	0.76	1.00	1.00	5.10	2.20	0.29	1.10	0.99	1.00
X - 24295	Unknown	Unknown	1.51	2.41	0.73	0.57	1.00	1.00	1.28	3.32	0.77	0.70	1.00	1.00	1.15	3.95	0.49	0.84	0.99	1.00

X - 24328	Unknown	Unknown	1.18	0.96	0.22	0.96	1.00	1.00	1.15	0.97	0.28	0.93	0.85	1.00	1.13	1.00	0.27	0.90	0.99	1.00
X - 24337	Unknown	Unknown	2.97	1.79	0.04	0.54	1.00	1.00	2.30	3.68	0.05	0.55	0.73	1.00	2.78	3.65	0.83	0.35	0.99	1.00
X - 24455	Unknown	Unknown	6.54	3.48	0.10	0.73	1.00	1.00	1.92	3.15	0.44	0.62	0.91	1.00	5.59	4.14	0.41	0.44	0.99	1.00
X - 24473	Unknown	Unknown	21.90	5.48	0.03	1.25	1.00	1.00	18.53	7.50	0.51	0.75	0.95	1.00	9.87	15.56	0.90	0.38	0.99	1.00
X - 24475	Unknown	Unknown	1.06	1.35	0.35	1.20	1.00	1.00	1.19	1.37	0.88	0.64	1.00	1.00	1.31	1.22	0.80	0.51	0.99	1.00
X - 24494	Unknown	Unknown	1.12	1.19	0.37	0.58	1.00	1.00	1.08	1.15	0.92	0.67	1.00	1.00	1.16	1.19	0.47	0.81	0.99	1.00
X - 24527	Unknown	Unknown	1.26	6.50	0.68	0.87	1.00	1.00	1.16	3.53	0.52	0.89	0.96	1.00	1.51	4.01	0.15	0.87	0.99	1.00
X - 24542	Unknown	Unknown	1.50	1.65	0.78	0.80	1.00	1.00	2.12	1.42	0.31	1.05	0.88	1.00	1.63	1.51	0.45	0.60	0.99	1.00
X - 24544	Unknown	Unknown	1.11	1.04	0.40	0.56	1.00	1.00	1.11	1.04	0.44	0.68	0.91	1.00	1.13	1.07	0.53	0.68	0.99	1.00
X - 24556	Unknown	Unknown	2.02	1.06	0.53	0.97	1.00	1.00	2.15	1.00	0.70	1.02	1.00	1.00	1.61	1.06	0.31	1.04	0.99	1.00
X - 24571	Unknown	Unknown	3.86	4.41	0.74	0.18	1.00	1.00	5.95	4.37	0.52	0.38	0.96	1.00	4.00	2.14	0.94	1.07	0.99	1.00
X - 24686	Unknown	Unknown	3.11	2.05	0.60	0.46	1.00	1.00	1.82	2.08	0.84	0.22	1.00	1.00	1.66	3.98	0.46	0.68	0.99	1.00
X - 24748	Unknown	Unknown	5.65	9.39	0.54	0.88	1.00	1.00	4.46	10.51	0.77	1.27	1.00	1.00	5.08	8.60	0.67	0.84	0.99	1.00
X - 24757	Unknown	Unknown	1.02	1.93	0.52	0.83	1.00	1.00	0.78	3.04	0.28	1.10	0.85	1.00	0.98	3.11	0.56	1.05	0.99	1.00
X - 24809	Unknown	Unknown	2.99	9.67	0.96	1.03	1.00	1.00	1.18	4.53	0.83	1.22	1.00	1.00	4.10	7.02	0.32	0.64	0.99	1.00
X - 24811	Unknown	Unknown	4.95	4.62	0.60	0.10	1.00	1.00	5.28	3.31	0.08	0.47	0.73	1.00	8.09	6.23	0.42	0.42	0.99	1.00
X - 24849	Unknown	Unknown	1.31	1.32	0.48	0.48	1.00	1.00	1.08	1.17	0.62	0.63	1.00	1.00	1.25	1.27	0.53	0.35	0.99	1.00
X - 24947	Unknown	Unknown	1.24	1.14	0.30	0.59	1.00	1.00	1.21	1.04	0.14	1.08	0.78	1.00	1.18	1.08	0.34	0.73	0.99	1.00
X - 24949	Unknown	Unknown	1.37	1.84	0.62	0.70	1.00	1.00	1.51	1.44	0.55	0.13	0.97	1.00	1.72	1.68	0.84	0.26	0.99	1.00
X - 24972	Unknown	Unknown	4.57	7.40	0.44	0.54	1.00	1.00	4.41	5.26	0.97	0.16	1.00	1.00	1.03	5.22	0.58	1.21	0.99	1.00
X - 25271	Unknown	Unknown	4.89	6.94	0.27	0.32	1.00	1.00	3.09	6.78	0.84	0.60	1.00	1.00	2.05	5.13	0.69	1.10	0.99	1.00
X - 25343	Unknown	Unknown	6.58	14.04	0.26	0.67	1.00	1.00	6.01	21.09	0.09	0.84	0.75	1.00	5.53	14.65	0.33	0.96	0.99	1.00
X - 25419	Unknown	Unknown	3.60	0.94	0.07	1.02	1.00	1.00	3.68	1.00	0.02	1.05	0.73	1.00	5.88	1.04	0.20	1.03	0.99	1.00
X - 25420	Unknown	Unknown	1.58	4.21	0.28	0.86	1.00	1.00	1.73	1.78	0.32	0.11	0.88	1.00	1.59	3.13	0.60	0.84	0.99	1.00
X - 25519	Unknown	Unknown	6.37	9.24	0.65	0.41	1.00	1.00	4.32	12.77	0.76	0.97	1.00	1.00	5.84	10.30	0.48	0.67	0.99	1.00
X - 25520	Unknown	Unknown	8.89	7.42	0.98	0.18	1.00	1.00	21.60	15.36	0.54	0.28	0.97	1.00	10.36	43.41	0.19	0.77	0.99	1.00
