# Supplementary material for: Metabolic Alterations in Older Women With Low Bone Mineral Density Supplemented With Lactobacillus reuteri
Source: JBMR Plus. 2021 Mar 15;5(4):e10478. doi: 10.1002/jbm4.10478 (PMC8046097; doi:10.1002/jbm4.10478)
Supplement: Supplementary file 5 — Table S3: The four response patterns identified by clustering analysis of the metabolites that responded differently between the L. reuteri and placebo groups. [file JBM4-5-e10478-s002.pdf]

**Supplemental Table 3**

| Biochemical Name | Super Pathway | Sub Pathway | Cluster |
|---|---|---|---|
| 1-linoleoyl-2-linolenoyl-GPC (18:2/18:3)* | Lipid | Phosphatidylcholine (PC) | 1 |
| 1-methyl-4-imidazoleacetate | Amino Acid | Histidine Metabolism | 2 |
| 1-oleoyl-2-linoleoyl-GPE (18:1/18:2)* | Lipid | Phosphatidylethanolamine (PE) | 3 |
| 1-palmitoyl-2-linoleoyl-GPE (16:0/18:2) | Lipid | Phosphatidylethanolamine (PE) | 3 |
| 1-ribosyl-imidazoleacetate* | Amino Acid | Histidine Metabolism | 2 |
| 1-stearoyl-2-linoleoyl-GPE (18:0/18:2)* | Lipid | Phosphatidylethanolamine (PE) | 3 |
| 1-stearoyl-2-linoleoyl-GPI (18:0/18:2) | Lipid | Phosphatidylinositol (PI) | 1 |
| 3-hydroxypyridine sulfate | Xenobiotics | Chemical | 3 |
| 4-acetamidobutanoate | Amino Acid | Polyamine Metabolism | 4 |
| 4-ethylphenyl sulfate | Xenobiotics | Benzoate Metabolism | 3 |
| bilirubin (E,Z or Z,E)* | Cofactors and Vitamins | Hemoglobin and Porphyrin Metabolism | 1 |
| butyrylcarnitine (C4) | Lipid | Fatty Acid Metabolism (also BCAA Metabolism) | 4 |
| caprate (10:0) | Lipid | Medium Chain Fatty Acid | 3 |
| citraconate/glutaconate | Energy | TCA Cycle | 3 |
| deoxycarnitine | Lipid | Carnitine Metabolism | 2 |
| dopamine 3-O-sulfate | Amino Acid | Tyrosine Metabolism | 3 |
| gamma-glutamyl-alpha-lysine | Peptide | Gamma-glutamyl Amino Acid | 2 |
| gamma-glutamylleucine | Peptide | Gamma-glutamyl Amino Acid | 3 |
| gamma-glutamylthreonine | Peptide | Gamma-glutamyl Amino Acid | 4 |
| glycerol | Lipid | Glycerolipid Metabolism | 4 |
| heptenedioate (C7:1-DC)* | Lipid | Fatty Acid, Dicarboxylate | 4 |
| hydroxypalmitoyl sphingomyelin (d18:1/16:0(OH)) | Lipid | Sphingomyelins | 1 |
| lactosyl-N-palmitoyl-sphingosine (d18:1/16:0) | Lipid | Lactosylceramides (LCER) | 1 |
| mannitol/sorbitol | Carbohydrate | Fructose, Mannose and Galactose Metabolism | 3 |
| myristoyl dihydrosphingomyelin (d18:0/14:0)* | Lipid | Dihydrosphingomyelins | 1 |
| N,N,N-trimethyl-alanylproline betaine (TMAP) | Amino Acid | Urea cycle; Arginine and Proline Metabolism | 2 |
| N6,N6-dimethyllysine | Amino Acid | Lysine Metabolism | 2 |
| N-methylpipecolate | Xenobiotics | Bacterial/Fungal | 2 |
| orotidine | Nucleotide | Pyrimidine Metabolism, Orotate containing | 2 |
| proline | Amino Acid | Urea cycle; Arginine and Proline Metabolism | 3 |
| quinolinate | Cofactors and Vitamins | Nicotinate and Nicotinamide Metabolism | 2 |
| sphingomyelin (d17:1/14:0, d16:1/15:0)* | Lipid | Sphingomyelins | 1 |
| sphingomyelin (d17:2/16:0, d18:2/15:0)* | Lipid | Sphingomyelins | 1 |
| sphingomyelin (d18:1/22:2, d18:2/22:1, d16:1/24:2)* | Lipid | Sphingomyelins | 1 |
| sphingomyelin (d18:2/14:0, d18:1/14:1)* | Lipid | Sphingomyelins | 1 |
| sphingomyelin (d18:2/21:0, d16:2/23:0)* | Lipid | Sphingomyelins | 1 |
| sphingomyelin (d18:2/23:0, d18:1/23:1, d17:1/24:1)* | Lipid | Sphingomyelins | 1 |
| sphingomyelin (d18:2/23:1)* | Lipid | Sphingomyelins | 1 |
| trigonelline (N'-methylnicotinate) | Cofactors and Vitamins | Nicotinate and Nicotinamide Metabolism | 3 |
| X - 11372 | Unknown | Unknown | 2 |
| X - 13431 | Unknown | Unknown | 2 |

| | | | |
|---|---|---|---|
| X - 17676 | Unknown | Unknown | 3 |
| X - 23369 | Unknown | Unknown | 3 |
| X - 25422 | Unknown | Unknown | 2 |
| 1-lignoceroyl-GPC (24:0) | Lipid | Lysophospholipid | 1 |
| 2'-O-methyluridine | Nucleotide | Pyrimidine Metabolism, Uracil containing | 4 |
| 3-ethylcatechol sulfate (1) | Xenobiotics | Food Component/Plant | 3 |
| 3-methyl catechol sulfate (1) | Xenobiotics | Benzoate Metabolism | 3 |
| andro steroid monosulfate C19H28O6S (1)* | Lipid | Androgenic Steroids | 4 |
| caprylate (8:0) | Lipid | Medium Chain Fatty Acid | 3 |
| gamma-glutamyltryptophan | Peptide | Gamma-glutamyl Amino Acid | 2 |
| gamma-glutamyltyrosine | Peptide | Gamma-glutamyl Amino Acid | 3 |
| isovalerylglycine | Amino Acid | Leucine, Isoleucine and Valine Metabolism | 3 |
| N-(2-furoyl)glycine | Xenobiotics | Food Component/Plant | 3 |
| N-acetylneuraminate | Carbohydrate | Aminosugar Metabolism | 2 |
| palmitoyl-arachidonoyl-glycerol (16:0/20:4) [1]* | Lipid | Diacylglycerol | 1 |
| succinylcarnitine (C4) | Energy | TCA Cycle | 2 |
| X - 12101 | Unknown | Unknown | 4 |
| X - 12170 | Unknown | Unknown | 3 |
| X - 12739 | Unknown | Unknown | 4 |
| X - 12812 | Unknown | Unknown | 3 |
| X - 12822 | Unknown | Unknown | 4 |
| X - 13844 | Unknown | Unknown | 3 |
| X - 15461 | Unknown | Unknown | 3 |
| X - 18345 | Unknown | Unknown | 3 |
