# Supplementary material for: Metabolic Alterations in Older Women With Low Bone Mineral Density Supplemented With Lactobacillus reuteri
Source: JBMR Plus. 2021 Mar 15;5(4):e10478. doi: 10.1002/jbm4.10478 (PMC8046097; doi:10.1002/jbm4.10478)
Supplement: Supplementary file 6 — Table S4: The differential metabolites between the case and control groups, comparing by linear regression models adjusted by the confounders age and BMI. [file JBM4-5-e10478-s004.pdf]

Supplemental Table 4

Biochemical Name	Super Pathway	Sub Pathway	log2 (fold change	P	Adj. P FDR	Adj. P Bonf.	VIP
(14 or 15)-methylpalmitate (a17:0 or i17:0)	Lipid	Fatty Acid, Branched	0.10	0.45	0.84	1.00	1.62
(16 or 17)-methylstearate (a19:0 or i19:0)	Lipid	Fatty Acid, Branched	0.08	0.37	0.80	1.00	1.43
(N(1) + N(8))-acetylspermidine	Amino Acid	Polyamine Metabolism	0.08	0.30	0.75	1.00	1.45
1-(1-enyl-palmitoyl)-2-arachidonoyl-GPC (P-16:0/20:4)*	Lipid	Plasmalogen	-0.02	0.69	0.91	1.00	0.88
1-(1-enyl-palmitoyl)-2-arachidonoyl-GPE (P-16:0/20:4)*	Lipid	Plasmalogen	-0.05	0.22	0.70	1.00	1.07
1-(1-enyl-palmitoyl)-2-linoleoyl-GPC (P-16:0/18:2)*	Lipid	Plasmalogen	-0.08	0.12	0.63	1.00	0.87
1-(1-enyl-palmitoyl)-2-linoleoyl-GPE (P-16:0/18:2)*	Lipid	Plasmalogen	-0.07	0.32	0.78	1.00	1.21
1-(1-enyl-palmitoyl)-2-oleoyl-GPC (P-16:0/18:1)*	Lipid	Plasmalogen	0.04	0.62	0.90	1.00	0.97
1-(1-enyl-palmitoyl)-2-oleoyl-GPE (P-16:0/18:1)*	Lipid	Plasmalogen	-0.01	0.66	0.90	1.00	0.98
1-(1-enyl-palmitoyl)-2-palmitoleoyl-GPC (P-16:0/16:1)*	Lipid	Plasmalogen	0.08	0.26	0.72	1.00	1.00
1-(1-enyl-palmitoyl)-2-palmitoyl-GPC (P-16:0/16:0)*	Lipid	Plasmalogen	-0.04	0.23	0.70	1.00	0.93
1-(1-enyl-palmitoyl)-GPC (P-16:0)*	Lipid	Lysoplasmalogen	-0.05	0.32	0.78	1.00	0.86
1-(1-enyl-palmitoyl)-GPE (P-16:0)*	Lipid	Lysoplasmalogen	-0.08	0.10	0.63	1.00	1.20
1-(1-enyl-stearoyl)-2-arachidonoyl-GPE (P-18:0/20:4)*	Lipid	Plasmalogen	-0.03	0.36	0.79	1.00	0.89
1-(1-enyl-stearoyl)-2-linoleoyl-GPE (P-18:0/18:2)*	Lipid	Plasmalogen	-0.02	0.70	0.91	1.00	1.03
1-(1-enyl-stearoyl)-2-oleoyl-GPE (P-18:0/18:1)	Lipid	Plasmalogen	0.00	0.79	0.94	1.00	0.95
1-(1-enyl-stearoyl)-GPE (P-18:0)*	Lipid	Lysoplasmalogen	-0.07	0.13	0.64	1.00	1.03
1,2-dilinoleoyl-GPC (18:2/18:2)	Lipid	Phosphatidylcholine (PC)	-0.03	0.60	0.90	1.00	1.16
1,2-dipalmitoyl-GPC (16:0/16:0)	Lipid	Phosphatidylcholine (PC)	-0.01	0.43	0.83	1.00	1.12
1,5-anhydroglucitol (1,5-AG)	Carbohydrate	Glycolysis, Gluconeogenesis, and Pyruvate Metabolism	-0.06	0.34	0.78	1.00	0.66
1,7-dimethylurate	Xenobiotics	Xanthine Metabolism	0.16	0.27	0.72	1.00	0.94
10-heptadecenoate (17:1n7)	Lipid	Long Chain Fatty Acid	0.16	0.23	0.70	1.00	1.98
10-nonadecenoate (19:1n9)	Lipid	Long Chain Fatty Acid	0.07	0.64	0.90	1.00	1.92
10-undecenoate (11:1n1)	Lipid	Medium Chain Fatty Acid	-0.09	0.04	0.42	1.00	1.39
13-HODE + 9-HODE	Lipid	Fatty Acid, Monohydroxy	0.21	0.11	0.63	1.00	1.00
16a-hydroxy DHEA 3-sulfate	Lipid	Androgenic Steroids	-0.12	0.95	0.98	1.00	0.35
16-hydroxypalmitate	Lipid	Fatty Acid, Monohydroxy	0.08	0.16	0.69	1.00	1.10
1-arachidonoyl-GPC* (20:4)*	Lipid	Lysophospholipid	-0.05	0.25	0.72	1.00	0.83
1-arachidonoyl-GPE (20:4n6)*	Lipid	Lysophospholipid	0.03	0.44	0.84	1.00	0.77
1-arachidonoyl-GPI* (20:4)*	Lipid	Lysophospholipid	0.13	0.04	0.45	1.00	1.37
1-arachidonylglycerol (20:4)	Lipid	Monoacylglycerol	0.00	0.83	0.97	1.00	0.65
1-carboxyethylphenylalanine	Amino Acid	Phenylalanine Metabolism	0.16	0.11	0.63	1.00	1.36
1-dihomo-linolenylglycerol (20:3)	Lipid	Monoacylglycerol	0.10	0.29	0.75	1.00	0.53
1-docosahexaenoylglycerol (22:6)	Lipid	Monoacylglycerol	-0.04	0.57	0.89	1.00	0.97
1-linolenoyl-GPC (18:3)*	Lipid	Lysophospholipid	-0.04	0.64	0.90	1.00	0.84
1-linoleoyl-2-linolenoyl-GPC (18:2/18:3)*	Lipid	Phosphatidylcholine (PC)	-0.04	0.80	0.94	1.00	0.94
1-linoleoylglycerol (18:2)	Lipid	Monoacylglycerol	0.02	0.99	1.00	1.00	0.44
1-linoleoyl-GPC (18:2)	Lipid	Lysophospholipid	-0.03	0.25	0.72	1.00	1.30
1-linoleoyl-GPE (18:2)*	Lipid	Lysophospholipid	0.06	0.39	0.81	1.00	1.01
1-linoleoyl-GPI* (18:2)*	Lipid	Lysophospholipid	0.13	0.12	0.63	1.00	1.05
1-methyl-4-imidazoleacetate	Amino Acid	Histidine Metabolism	0.11	0.33	0.78	1.00	1.00
1-methylhistidine	Amino Acid	Histidine Metabolism	-0.02	0.57	0.89	1.00	0.84
1-methylnicotinamide	Cofactors and Vitamins	Nicotinate and Nicotinamide Metabolism	0.08	0.72	0.92	1.00	0.65
1-myristoyl-2-arachidonoyl-GPC (14:0/20:4)*	Lipid	Phosphatidylcholine (PC)	-0.16	0.07	0.57	1.00	1.09
1-myristoyl-2-palmitoyl-GPC (14:0/16:0)	Lipid	Phosphatidylcholine (PC)	-0.05	0.50	0.86	1.00	0.58

1-myristoylglycerol (14:0)	Lipid	Monoacylglycerol	-0.03	0.76	0.93	1.00	0.24
1-oleoyl-2-docosahexaenoyl-GPC (18:1/22:6)*	Lipid	Phosphatidylcholine (PC)	0.00	0.98	1.00	1.00	1.03
1-oleoyl-2-linoleoyl-GPE (18:1/18:2)*	Lipid	Phosphatidylethanolamine (PE)	-0.05	0.31	0.77	1.00	0.61
1-oleoylglycerol (18:1)	Lipid	Monoacylglycerol	0.07	0.35	0.79	1.00	0.53
1-oleoyl-GPC (18:1)	Lipid	Lysophospholipid	0.02	0.61	0.90	1.00	1.52
1-oleoyl-GPE (18:1)	Lipid	Lysophospholipid	0.02	0.99	1.00	1.00	0.44
1-oleoyl-GPI (18:1)*	Lipid	Lysophospholipid	0.14	0.19	0.69	1.00	1.03
1-palmitoleoylglycerol (16:1)*	Lipid	Monoacylglycerol	0.10	0.21	0.70	1.00	0.64
1-palmitoleoyl-GPC* (16:1)*	Lipid	Lysophospholipid	0.09	0.08	0.59	1.00	1.39
1-palmitoyl-2-arachidonoyl-GPC (16:0/20:4n6)	Lipid	Phosphatidylcholine (PC)	-0.04	0.19	0.69	1.00	1.11
1-palmitoyl-2-arachidonoyl-GPE (16:0/20:4)*	Lipid	Phosphatidylethanolamine (PE)	-0.01	0.68	0.90	1.00	0.28
1-palmitoyl-2-arachidonoyl-GPI (16:0/20:4)*	Lipid	Phosphatidylinositol (PI)	0.05	0.41	0.83	1.00	0.82
1-palmitoyl-2-dihomo-linolenoyl-GPC (16:0/20:3n3 or 6)*	Lipid	Phosphatidylcholine (PC)	0.00	0.98	0.99	1.00	1.27
1-palmitoyl-2-docosahexaenoyl-GPC (16:0/22:6)	Lipid	Phosphatidylcholine (PC)	-0.06	0.02	0.37	1.00	1.41
1-palmitoyl-2-docosahexaenoyl-GPE (16:0/22:6)*	Lipid	Phosphatidylethanolamine (PE)	-0.10	0.09	0.62	1.00	0.98
1-palmitoyl-2-linoleoyl-GPC (16:0/18:2)	Lipid	Phosphatidylcholine (PC)	-0.04	0.09	0.60	1.00	1.45
1-palmitoyl-2-linoleoyl-GPE (16:0/18:2)	Lipid	Phosphatidylethanolamine (PE)	-0.01	0.56	0.89	1.00	0.40
1-palmitoyl-2-linoleoyl-GPI (16:0/18:2)	Lipid	Phosphatidylinositol (PI)	-0.01	0.68	0.90	1.00	0.65
1-palmitoyl-2-oleoyl-GPC (16:0/18:1)	Lipid	Phosphatidylcholine (PC)	-0.01	0.59	0.90	1.00	1.02
1-palmitoyl-2-oleoyl-GPE (16:0/18:1)	Lipid	Phosphatidylethanolamine (PE)	-0.06	0.40	0.82	1.00	0.58
1-palmitoyl-2-oleoyl-GPI (16:0/18:1)*	Lipid	Phosphatidylinositol (PI)	-0.06	0.93	0.98	1.00	0.20
1-palmitoyl-2-palmitoleoyl-GPC (16:0/16:1)*	Lipid	Phosphatidylcholine (PC)	0.07	0.17	0.69	1.00	0.85
1-palmitoyl-2-stearoyl-GPC (16:0/18:0)	Lipid	Phosphatidylcholine (PC)	0.00	0.78	0.94	1.00	1.33
1-palmitoyl-GPC (16:0)	Lipid	Lysophospholipid	-0.03	0.27	0.72	1.00	1.40
1-palmitoyl-GPE (16:0)	Lipid	Lysophospholipid	0.01	0.99	1.00	1.00	0.89
1-palmitoyl-GPI* (16:0)	Lipid	Lysophospholipid	0.15	0.12	0.63	1.00	1.00
1-ribosyl-imidazoleacetate*	Amino Acid	Histidine Metabolism	-0.05	0.20	0.69	1.00	0.80
1-stearoyl-2-arachidonoyl-GPC (18:0/20:4)	Lipid	Phosphatidylcholine (PC)	-0.03	0.28	0.73	1.00	1.24
1-stearoyl-2-arachidonoyl-GPE (18:0/20:4)	Lipid	Phosphatidylethanolamine (PE)	-0.04	0.48	0.86	1.00	0.52
1-stearoyl-2-arachidonoyl-GPI (18:0/20:4)	Lipid	Phosphatidylinositol (PI)	0.03	0.52	0.88	1.00	1.16
1-stearoyl-2-docosahexaenoyl-GPC (18:0/22:6)	Lipid	Phosphatidylcholine (PC)	-0.06	0.08	0.60	1.00	1.17
1-stearoyl-2-docosahexaenoyl-GPE (18:0/22:6)*	Lipid	Phosphatidylethanolamine (PE)	-0.15	0.03	0.41	1.00	1.34
1-stearoyl-2-linoleoyl-GPC (18:0/18:2)*	Lipid	Phosphatidylcholine (PC)	-0.04	0.13	0.64	1.00	1.62
1-stearoyl-2-linoleoyl-GPE (18:0/18:2)*	Lipid	Phosphatidylethanolamine (PE)	-0.04	0.40	0.82	1.00	0.65
1-stearoyl-2-linoleoyl-GPI (18:0/18:2)	Lipid	Phosphatidylinositol (PI)	0.00	0.85	0.97	1.00	0.80
1-stearoyl-2-oleoyl-GPC (18:0/18:1)	Lipid	Phosphatidylcholine (PC)	0.01	0.70	0.91	1.00	1.11
1-stearoyl-2-oleoyl-GPI (18:0/18:1)*	Lipid	Phosphatidylinositol (PI)	-0.05	0.51	0.87	1.00	0.54
1-stearoyl-GPC (18:0)	Lipid	Lysophospholipid	-0.02	0.36	0.79	1.00	1.44
1-stearoyl-GPE (18:0)	Lipid	Lysophospholipid	0.02	0.89	0.98	1.00	1.02
1-stearoyl-GPI (18:0)	Lipid	Lysophospholipid	-0.04	0.83	0.97	1.00	0.73
2,3-dihydroxy-5-methylthio-4-pentenoate (DMTPA)*	Amino Acid	Methionine, Cysteine, SAM and Taurine Metabolism	0.02	0.54	0.89	1.00	1.40
21-hydroxypregnenolone disulfate	Lipid	Pregnenolone Steroids	-0.28	0.03	0.41	1.00	1.28
2-aminobutyrate	Amino Acid	Glutathione Metabolism	-0.07	0.09	0.60	1.00	1.25
2-aminophenol sulfate	Xenobiotics	Chemical	-0.30	0.19	0.69	1.00	0.85
2-hydroxy-3-methylvalerate	Amino Acid	Leucine, Isoleucine and Valine Metabolism	-0.04	0.62	0.90	1.00	0.37
2-hydroxybutyrate/2-hydroxyisobutyrate	Amino Acid	Glutathione Metabolism	0.01	0.91	0.98	1.00	0.35
2-hydroxydecanoate	Lipid	Fatty Acid, Monohydroxy	0.08	0.78	0.94	1.00	0.29
2-hydroxyglutarate	Lipid	Fatty Acid, Dicarboxylate	0.10	0.12	0.63	1.00	1.79

2-hydroxynervonate*	Lipid	Fatty Acid, Monohydroxy	0.10	0.10	0.63	1.00	1.08
2-hydroxyoctanoate	Lipid	Fatty Acid, Monohydroxy	-0.09	0.49	0.86	1.00	0.57
2-hydroxypalmitate	Lipid	Fatty Acid, Monohydroxy	0.12	0.05	0.48	1.00	1.16
2-hydroxystearate	Lipid	Fatty Acid, Monohydroxy	0.08	0.36	0.79	1.00	0.86
2-keto-3-deoxy-gluconate	Xenobiotics	Food Component/Plant	0.04	0.73	0.93	1.00	1.12
2-linoleoylglycerol (18:2)	Lipid	Monoacylglycerol	-0.02	0.91	0.98	1.00	0.12
2-methylcitrate/homocitrate	Energy	TCA Cycle	0.00	0.93	0.98	1.00	1.13
2-oleoylglycerol (18:1)	Lipid	Monoacylglycerol	0.11	0.17	0.69	1.00	0.79
2-palmitoyl-GPC* (16:0)*	Lipid	Lysophospholipid	0.21	0.10	0.63	1.00	1.03
2-piperidinone	Xenobiotics	Food Component/Plant	-0.06	0.34	0.78	1.00	1.11
2-pyrrolidinone	Amino Acid	Glutamate Metabolism	-0.07	0.13	0.64	1.00	0.96
2-stearoyl-GPE (18:0)*	Lipid	Lysophospholipid	0.11	0.23	0.70	1.00	0.87
3-(4-hydroxyphenyl)lactate (HPLA)	Amino Acid	Tyrosine Metabolism	0.07	0.47	0.84	1.00	1.24
3,4-dihydroxybutyrate	Amino Acid	Glutamate Metabolism	0.23	0.03	0.41	1.00	1.92
3-aminoisobutyrate	Nucleotide	Pyrimidine Metabolism, Thymine containing	-0.06	0.64	0.90	1.00	0.29
3beta,7alpha-dihydroxy-5-cholestenoate	Lipid	Sterol	0.00	0.90	0.98	1.00	0.51
3beta-hydroxy-5-cholestenoate	Lipid	Sterol	0.00	0.62	0.90	1.00	0.41
3-carboxy-4-methyl-5-pentyl-2-furanpropionate (3-CMPFP)	Lipid	Fatty Acid, Dicarboxylate	-0.23	0.00	0.12	0.61	1.98
3-carboxy-4-methyl-5-propyl-2-furanpropanoate (CMPF)	Lipid	Fatty Acid, Dicarboxylate	-0.45	0.00	0.11	0.30	2.14
3-formylindole	Xenobiotics	Food Component/Plant	-0.02	0.69	0.91	1.00	1.31
3-hydroxy-2-ethylpropionate	Amino Acid	Leucine, Isoleucine and Valine Metabolism	-0.03	0.49	0.86	1.00	0.41
3-hydroxy-3-methylglutarate	Lipid	Mevalonate Metabolism	0.05	0.65	0.90	1.00	0.48
3-hydroxydecanoate	Lipid	Fatty Acid, Monohydroxy	0.13	0.53	0.89	1.00	0.83
3-hydroxyhexanoate	Lipid	Fatty Acid, Monohydroxy	0.09	0.27	0.72	1.00	0.80
3-hydroxylaurate	Lipid	Fatty Acid, Monohydroxy	0.08	0.94	0.98	1.00	1.43
3-hydroxymyristate	Lipid	Fatty Acid, Monohydroxy	0.07	0.67	0.90	1.00	1.18
3-hydroxyoctanoate	Lipid	Fatty Acid, Monohydroxy	0.18	0.22	0.70	1.00	0.83
3-hydroxyoleate*	Lipid	Fatty Acid, Monohydroxy	0.15	0.34	0.78	1.00	1.33
3-hydroxypyridine sulfate	Xenobiotics	Chemical	0.31	0.35	0.79	1.00	1.30
3-indoxyl sulfate	Amino Acid	Tryptophan Metabolism	0.05	0.58	0.89	1.00	1.01
3-methyl-2-oxobutyrate	Amino Acid	Leucine, Isoleucine and Valine Metabolism	-0.06	0.05	0.48	1.00	1.13
3-methyl-2-oxovalerate	Amino Acid	Leucine, Isoleucine and Valine Metabolism	-0.05	0.19	0.69	1.00	1.03
3-methylglutaconate	Amino Acid	Leucine, Isoleucine and Valine Metabolism	-0.09	0.64	0.90	1.00	0.32
3-methylhistidine	Amino Acid	Histidine Metabolism	-0.13	0.65	0.90	1.00	0.55
3-ureidopropionate	Nucleotide	Pyrimidine Metabolism, Uracil containing	-0.03	0.23	0.70	1.00	0.79
4-acetamidobutanoate	Amino Acid	Polyamine Metabolism	-0.05	0.36	0.79	1.00	0.68
4-allylphenol sulfate	Xenobiotics	Food Component/Plant	-0.21	0.13	0.64	1.00	0.87
4-cholesten-3-one	Lipid	Sterol	-0.02	0.96	0.99	1.00	0.59
4-ethylphenyl sulfate	Xenobiotics	Benzoate Metabolism	-0.09	0.05	0.48	1.00	1.10
4-hydroxyhippurate	Xenobiotics	Benzoate Metabolism	0.04	0.82	0.96	1.00	0.46
4-hydroxyphenylpyruvate	Amino Acid	Tyrosine Metabolism	0.11	0.05	0.48	1.00	2.24
4-methylcatechol sulfate	Xenobiotics	Benzoate Metabolism	-0.22	0.39	0.80	1.00	0.55
4-vinylphenol sulfate	Xenobiotics	Benzoate Metabolism	0.05	0.82	0.96	1.00	0.57
5,6-dihydrothymine	Nucleotide	Pyrimidine Metabolism, Thymine containing	0.01	0.76	0.93	1.00	0.79
5,6-dihydrouridine	Nucleotide	Pyrimidine Metabolism, Uracil containing	-0.02	0.68	0.90	1.00	0.85
5-acetylamino-6-amino-3-methyluracil	Xenobiotics	Xanthine Metabolism	0.12	0.14	0.66	1.00	1.46
5-dodecenoate (12:1n7)	Lipid	Medium Chain Fatty Acid	0.35	0.01	0.32	1.00	1.61
5-hydroxylysine	Amino Acid	Lysine Metabolism	0.05	0.48	0.86	1.00	1.03

5-methylthioadenosine (MTA)	Amino Acid	Polyamine Metabolism	-0.02	0.78	0.94	1.00	1.00
5-methylthioribose	Amino Acid	Methionine, Cysteine, SAM and Taurine Metabolism	-0.04	0.35	0.79	1.00	0.97
5-methyluridine (ribothymidine)	Nucleotide	Pyrimidine Metabolism, Uracil containing	-0.04	0.29	0.74	1.00	1.26
5-oxoproline	Amino Acid	Glutathione Metabolism	0.01	1.00	1.00	1.00	1.61
6-bromotryptophan	Amino Acid	Tryptophan Metabolism	-0.08	0.13	0.64	1.00	1.05
6-oxopiperidine-2-carboxylate	Amino Acid	Lysine Metabolism	-0.03	0.51	0.87	1.00	0.79
7-HOCA	Lipid	Sterol	0.04	0.12	0.64	1.00	1.41
7-methylguanine	Nucleotide	Purine Metabolism, Guanine containing	0.01	0.70	0.91	1.00	1.13
9-hydroxystearate	Lipid	Fatty Acid, Monohydroxy	0.37	0.01	0.31	1.00	1.49
acetylcarnitine (C2)	Lipid	Fatty Acid Metabolism(Acyl Carnitine)	-0.03	0.53	0.89	1.00	0.44
acisoga	Amino Acid	Polyamine Metabolism	0.13	0.23	0.70	1.00	0.93
aconitate [cis or trans]	Energy	TCA Cycle	0.10	0.07	0.57	1.00	1.13
adrenate (22:4n6)	Lipid	Polyunsaturated Fatty Acid (n3 and n6)	0.11	0.12	0.64	1.00	1.70
alanine	Amino Acid	Alanine and Aspartate Metabolism	0.03	0.89	0.98	1.00	1.62
allantoin	Nucleotide	Purine Metabolism, (Hypo)Xanthine/Inosine containing	-0.02	0.77	0.94	1.00	0.98
alpha-hydroxyisocaproate	Amino Acid	Leucine, Isoleucine and Valine Metabolism	-0.02	0.48	0.86	1.00	0.73
alpha-hydroxyisovalerate	Amino Acid	Leucine, Isoleucine and Valine Metabolism	-0.04	0.54	0.89	1.00	0.38
alpha-ketoglutaramate*	Amino Acid	Glutamate Metabolism	0.04	0.63	0.90	1.00	1.29
alpha-ketoglutarate	Energy	TCA Cycle	0.14	0.15	0.66	1.00	0.89
alpha-tocopherol	Cofactors and Vitamins	Tocopherol Metabolism	-0.05	0.18	0.69	1.00	0.95
androstenediol (3alpha, 17alpha) monosulfate (3)	Lipid	Androgenic Steroids	-0.30	0.01	0.24	1.00	1.56
androstenediol (3beta,17beta) disulfate (1)	Lipid	Androgenic Steroids	-0.28	0.06	0.51	1.00	1.13
androstenediol (3beta,17beta) disulfate (2)	Lipid	Androgenic Steroids	-0.26	0.04	0.47	1.00	1.14
androstenediol (3beta,17beta) monosulfate (1)	Lipid	Androgenic Steroids	-0.35	0.01	0.29	1.00	1.62
androsterone sulfate	Lipid	Androgenic Steroids	-0.34	0.00	0.21	1.00	1.54
arabitol/xylitol	Carbohydrate	Pentose Metabolism	0.03	0.81	0.95	1.00	0.59
arabonate/xylonate	Carbohydrate	Pentose Metabolism	0.11	0.46	0.84	1.00	1.02
arachidate (20:0)	Lipid	Long Chain Fatty Acid	0.05	0.48	0.86	1.00	0.96
arachidonate (20:4n6)	Lipid	Polyunsaturated Fatty Acid (n3 and n6)	0.09	0.16	0.69	1.00	0.84
arachidonoylcarnitine (C20:4)	Lipid	Fatty Acid Metabolism(Acyl Carnitine)	-0.06	0.43	0.84	1.00	0.70
arachidonoylcholine	Lipid	Fatty Acid Metabolism (Acyl Choline)	-0.02	0.97	0.99	1.00	0.82
argininate*	Amino Acid	Urea cycle; Arginine and Proline Metabolism	-0.14	0.23	0.70	1.00	1.22
arginine	Amino Acid	Urea cycle; Arginine and Proline Metabolism	-0.06	0.04	0.45	1.00	1.67
asparagine	Amino Acid	Alanine and Aspartate Metabolism	-0.04	0.24	0.71	1.00	1.70
aspartate	Amino Acid	Alanine and Aspartate Metabolism	-0.03	0.36	0.79	1.00	1.00
azelate (nonanedioate; C9)	Lipid	Fatty Acid, Dicarboxylate	0.01	0.88	0.98	1.00	0.15
behenoyl dihydrosphingomyelin (d18:0/22:0)*	Lipid	Dihydrosphingomyelins	-0.08	0.37	0.80	1.00	0.89
behenoyl sphingomyelin (d18:1/22:0)*	Lipid	Sphingomyelins	-0.06	0.06	0.55	1.00	1.42
beta-citrylglutamate	Amino Acid	Glutamate Metabolism	-0.07	0.22	0.70	1.00	0.80
beta-hydroxyisovalerate	Amino Acid	Leucine, Isoleucine and Valine Metabolism	-0.05	0.27	0.73	1.00	0.92
betaine	Amino Acid	Glycine, Serine and Threonine Metabolism	-0.02	0.65	0.90	1.00	1.43
bilirubin (E,E)*	Cofactors and Vitamins	Hemoglobin and Porphyrin Metabolism	-0.04	0.45	0.84	1.00	0.65
bilirubin (E,Z or Z,E)*	Cofactors and Vitamins	Hemoglobin and Porphyrin Metabolism	-0.08	0.21	0.70	1.00	0.79
bilirubin	Cofactors and Vitamins	Hemoglobin and Porphyrin Metabolism	-0.10	0.02	0.38	1.00	1.29
biliverdin	Cofactors and Vitamins	Hemoglobin and Porphyrin Metabolism	-0.08	0.46	0.84	1.00	0.80
caprate (10:0)	Lipid	Medium Chain Fatty Acid	0.20	0.07	0.57	1.00	1.16
carnitine	Lipid	Carnitine Metabolism	-0.08	0.03	0.41	1.00	1.63
carotene diol (1)	Cofactors and Vitamins	Vitamin A Metabolism	-0.10	0.16	0.69	1.00	0.87

carotene diol (2)	Cofactors and Vitamins	Vitamin A Metabolism	-0.06	0.36	0.79	1.00	0.65
catechol sulfate	Xenobiotics	Benzoate Metabolism	-0.05	0.26	0.72	1.00	0.81
ceramide (d16:1/24:1, d18:1/22:1)*	Lipid	Ceramides	-0.13	0.06	0.52	1.00	1.16
ceramide (d18:1/14:0, d16:1/16:0)*	Lipid	Ceramides	-0.07	0.19	0.69	1.00	0.84
ceramide (d18:1/20:0, d16:1/22:0, d20:1/18:0)*	Lipid	Ceramides	-0.09	0.12	0.63	1.00	1.03
ceramide (d18:2/24:1, d18:1/24:2)*	Lipid	Ceramides	-0.08	0.13	0.64	1.00	1.01
cerotoylcarnitine (C26)*	Lipid	Fatty Acid Metabolism(Acyl Carnitine)	-0.12	0.03	0.41	1.00	1.24
C-glycosyltryptophan	Amino Acid	Tryptophan Metabolism	0.01	1.00	1.00	1.00	1.06
cholesterol	Lipid	Sterol	-0.02	0.66	0.90	1.00	0.87
choline	Lipid	Phospholipid Metabolism	-0.04	0.25	0.72	1.00	1.35
cis-4-decenoylcarnitine (C10:1)	Lipid	Fatty Acid Metabolism(Acyl Carnitine)	-0.01	0.65	0.90	1.00	0.39
citraconate/glutaconate	Energy	TCA Cycle	0.15	0.39	0.81	1.00	0.97
citrate	Energy	TCA Cycle	0.05	0.50	0.86	1.00	0.42
citrulline	Amino Acid	Urea cycle; Arginine and Proline Metabolism	-0.08	0.06	0.51	1.00	1.33
cortisol	Lipid	Corticosteroids	-0.09	0.04	0.44	1.00	1.21
cortisone	Lipid	Corticosteroids	-0.05	0.07	0.57	1.00	1.06
creatine	Amino Acid	Creatine Metabolism	-0.03	0.30	0.75	1.00	0.97
creatinine	Amino Acid	Creatine Metabolism	-0.06	0.02	0.37	1.00	1.75
cys-gly, oxidized	Amino Acid	Glutathione Metabolism	-0.09	0.49	0.86	1.00	0.48
cysteine	Amino Acid	Methionine, Cysteine, SAM and Taurine Metabolism	-0.08	0.06	0.51	1.00	1.11
cysteine-glutathione disulfide	Amino Acid	Glutathione Metabolism	-0.03	0.94	0.98	1.00	0.49
cysteinylglycine disulfide*	Amino Acid	Glutathione Metabolism	-0.03	0.57	0.89	1.00	1.15
cystine	Amino Acid	Methionine, Cysteine, SAM and Taurine Metabolism	-0.01	0.78	0.94	1.00	1.24
decanoylcarnitine (C10)	Lipid	Fatty Acid Metabolism(Acyl Carnitine)	-0.04	0.68	0.90	1.00	0.21
dehydroepiandrosterone sulfate (DHEA-S)	Lipid	Androgenic Steroids	-0.30	0.02	0.37	1.00	1.30
deoxycarnitine	Lipid	Carnitine Metabolism	-0.09	0.03	0.41	1.00	1.48
diacylglycerol (12:0/18:1, 14:0/16:1, 16:0/14:1) [2]*	Lipid	Diacylglycerol	-0.02	0.77	0.93	1.00	0.30
diacylglycerol (14:0/18:1, 16:0/16:1) [1]*	Lipid	Diacylglycerol	-0.01	0.77	0.93	1.00	0.23
diacylglycerol (14:0/18:1, 16:0/16:1) [2]*	Lipid	Diacylglycerol	0.00	0.67	0.90	1.00	0.32
diacylglycerol (16:1/18:2 [2], 16:0/18:3 [1])*	Lipid	Diacylglycerol	0.08	0.29	0.74	1.00	0.80
dihomolinoleate (20:2n6)	Lipid	Polyunsaturated Fatty Acid (n3 and n6)	0.06	0.50	0.86	1.00	1.90
dihomolinolenate (20:3n3 or 3n6)	Lipid	Polyunsaturated Fatty Acid (n3 and n6)	0.08	0.19	0.69	1.00	0.82
dihomo-linolenoyl-choline	Lipid	Fatty Acid Metabolism (Acyl Choline)	-0.01	0.72	0.92	1.00	0.93
dihydroorotate	Nucleotide	Pyrimidine Metabolism, Orotate containing	0.08	0.58	0.89	1.00	0.94
dimethylarginine (ADMA + SDMA)	Amino Acid	Urea cycle; Arginine and Proline Metabolism	-0.03	0.59	0.90	1.00	1.34
dimethylglycine	Amino Acid	Glycine, Serine and Threonine Metabolism	0.02	0.89	0.98	1.00	1.15
docosadienoate (22:2n6)	Lipid	Polyunsaturated Fatty Acid (n3 and n6)	0.08	0.24	0.70	1.00	1.72
docosahexaenoate (DHA; 22:6n3)	Lipid	Polyunsaturated Fatty Acid (n3 and n6)	-0.02	0.67	0.90	1.00	1.02
docosahexaenoylcarnitine (C22:6)*	Lipid	Fatty Acid Metabolism(Acyl Carnitine)	-0.12	0.02	0.38	1.00	1.25
docosahexaenoylcholine	Lipid	Fatty Acid Metabolism (Acyl Choline)	-0.10	0.48	0.86	1.00	0.70
docosapentaenoate (DPA; 22:5n3)	Lipid	Polyunsaturated Fatty Acid (n3 and n6)	-0.02	0.86	0.98	1.00	1.78
dodecadienoate (12:2)*	Lipid	Fatty Acid, Dicarboxylate	-0.06	0.36	0.79	1.00	0.91
dodecanedioate (C12)	Lipid	Fatty Acid, Dicarboxylate	0.25	0.09	0.61	1.00	1.10
dopamine 3-O-sulfate	Amino Acid	Tyrosine Metabolism	-0.05	0.39	0.81	1.00	0.49
eicosanedioate (C20-DC)	Lipid	Fatty Acid, Dicarboxylate	0.19	0.17	0.69	1.00	1.50
eicosapentaenoate (EPA; 20:5n3)	Lipid	Polyunsaturated Fatty Acid (n3 and n6)	-0.08	0.38	0.80	1.00	0.74
eicosenoate (20:1n9 or 1n11)	Lipid	Long Chain Fatty Acid	0.00	1.00	1.00	1.00	2.00
ergothioneine	Xenobiotics	Food Component/Plant	-0.09	0.02	0.36	1.00	1.39

erucate (22:1n9)	Lipid	Long Chain Fatty Acid	0.02	0.87	0.98	1.00	0.93
erythritol	Xenobiotics	Food Component/Plant	-0.08	0.18	0.69	1.00	0.83
erythronate*	Carbohydrate	Aminosugar Metabolism	0.05	0.42	0.83	1.00	1.27
ethylmalonate	Amino Acid	Leucine, Isoleucine and Valine Metabolism	-0.11	0.35	0.79	1.00	0.75
Fibrinopeptide A (2-15)	Peptide	Fibrinogen Cleavage Peptide	-0.24	0.00	0.17	1.00	1.81
Fibrinopeptide A (3-15)	Peptide	Fibrinogen Cleavage Peptide	-0.25	0.01	0.24	1.00	1.63
Fibrinopeptide A (3-16)	Peptide	Fibrinogen Cleavage Peptide	0.02	0.64	0.90	1.00	0.33
Fibrinopeptide A (4-15)	Peptide	Fibrinogen Cleavage Peptide	-0.10	0.18	0.69	1.00	0.74
Fibrinopeptide A (5-16)*	Peptide	Fibrinogen Cleavage Peptide	-0.03	0.45	0.84	1.00	0.54
DSGEGDFXAEGGGVR*	Peptide	Fibrinogen Cleavage Peptide	-0.03	0.37	0.80	1.00	0.59
Fibrinopeptide B (1-12)	Peptide	Fibrinogen Cleavage Peptide	-0.16	0.07	0.57	1.00	1.07
Fibrinopeptide B (1-13)	Peptide	Fibrinogen Cleavage Peptide	0.05	0.39	0.81	1.00	0.89
fructose	Carbohydrate	Fructose, Mannose and Galactose Metabolism	0.00	0.59	0.90	1.00	0.67
gamma-CEHC	Cofactors and Vitamins	Tocopherol Metabolism	-0.22	0.03	0.41	1.00	1.25
gamma-glutamyl-alpha-lysine	Peptide	Gamma-glutamyl Amino Acid	-0.09	0.02	0.37	1.00	1.57
gamma-glutamylcitrulline*	Peptide	Gamma-glutamyl Amino Acid	-0.10	0.04	0.45	1.00	1.24
gamma-glutamylglutamine	Peptide	Gamma-glutamyl Amino Acid	-0.02	0.46	0.84	1.00	1.53
gamma-glutamylglycine	Peptide	Gamma-glutamyl Amino Acid	-0.05	0.70	0.91	1.00	0.59
gamma-glutamylhistidine	Peptide	Gamma-glutamyl Amino Acid	-0.02	0.83	0.97	1.00	0.86
gamma-glutamylleucine	Peptide	Gamma-glutamyl Amino Acid	-0.09	0.04	0.46	1.00	1.37
gamma-glutamylmethionine	Peptide	Gamma-glutamyl Amino Acid	-0.07	0.12	0.64	1.00	1.30
gamma-glutamylserine	Peptide	Gamma-glutamyl Amino Acid	0.02	0.89	0.98	1.00	1.10
gamma-glutamylthreonine	Peptide	Gamma-glutamyl Amino Acid	-0.03	0.57	0.89	1.00	1.04
gamma-glutamylvaline	Peptide	Gamma-glutamyl Amino Acid	-0.07	0.19	0.69	1.00	1.26
gamma-tocopherol/beta-tocopherol	Cofactors and Vitamins	Tocopherol Metabolism	-0.02	0.98	0.99	1.00	0.89
gluconate	Xenobiotics	Food Component/Plant	0.10	0.08	0.59	1.00	1.77
glucose	Carbohydrate	Glycolysis, Gluconeogenesis, and Pyruvate Metabolism	-0.04	0.22	0.70	1.00	1.05
glucuronate	Carbohydrate	Aminosugar Metabolism	-0.02	0.85	0.98	1.00	0.15
glutamate	Amino Acid	Glutamate Metabolism	-0.16	0.01	0.31	1.00	1.52
glutamine	Amino Acid	Glutamate Metabolism	-0.03	0.30	0.75	1.00	1.70
glutaroylcarnitine (C5)	Amino Acid	Lysine Metabolism	0.01	0.93	0.98	1.00	0.89
glycerate	Carbohydrate	Glycolysis, Gluconeogenesis, and Pyruvate Metabolism	0.00	0.77	0.94	1.00	1.34
glycerol	Lipid	Glycerolipid Metabolism	0.06	0.23	0.70	1.00	1.00
glycerophosphorylcholine (GPC)	Lipid	Phospholipid Metabolism	-0.18	0.29	0.75	1.00	0.79
glycine	Amino Acid	Glycine, Serine and Threonine Metabolism	-0.05	0.37	0.80	1.00	1.05
glycochenodeoxycholate	Lipid	Primary Bile Acid Metabolism	0.30	0.03	0.41	1.00	1.55
glycocholate	Lipid	Primary Bile Acid Metabolism	0.58	0.11	0.63	1.00	1.26
glycocholenate sulfate*	Lipid	Secondary Bile Acid Metabolism	-0.10	0.24	0.70	1.00	0.74
glycosyl ceramide (d18:2/24:1, d18:1/24:2)*	Lipid	Hexosylceramides (HCER)	0.07	0.29	0.75	1.00	1.15
glycosyl-N-palmitoyl-sphingosine (d18:1/16:0)	Lipid	Hexosylceramides (HCER)	0.09	0.18	0.69	1.00	1.39
glycosyl-N-stearoyl-sphingosine (d18:1/18:0)	Lipid	Hexosylceramides (HCER)	0.08	0.21	0.70	1.00	1.11
glycoursodeoxycholate	Lipid	Secondary Bile Acid Metabolism	0.00	0.23	0.70	1.00	0.77
guaiacol sulfate	Xenobiotics	Benzoate Metabolism	-0.04	0.70	0.91	1.00	0.60
guanidinoacetate	Amino Acid	Creatine Metabolism	-0.03	0.66	0.90	1.00	0.78
gulonate*	Cofactors and Vitamins	Ascorbate and Aldarate Metabolism	0.09	0.29	0.74	1.00	0.91
hexadecadienoate (16:2n6)	Lipid	Polyunsaturated Fatty Acid (n3 and n6)	0.00	0.73	0.93	1.00	1.75
hexadecanedioate (C16)	Lipid	Fatty Acid, Dicarboxylate	0.13	0.16	0.69	1.00	1.01
hexadecenedioate (C16:1-DC)*	Lipid	Fatty Acid, Dicarboxylate	0.20	0.06	0.52	1.00	1.14

hexanoylcarnitine (C6)	Lipid	Fatty Acid Metabolism(Acyl Carnitine)	-0.06	0.45	0.84	1.00	0.59
hippurate	Xenobiotics	Benzoate Metabolism	-0.02	0.68	0.90	1.00	0.76
histidine	Amino Acid	Histidine Metabolism	-0.03	0.32	0.78	1.00	1.79
homoarginine	Amino Acid	Urea cycle; Arginine and Proline Metabolism	-0.23	0.00	0.15	1.00	1.87
homostachydrine*	Xenobiotics	Food Component/Plant	0.28	0.98	1.00	1.00	0.51
hydroxyasparagine	Amino Acid	Alanine and Aspartate Metabolism	-0.03	0.55	0.89	1.00	0.76
hydroxy-CMPF*	Lipid	Fatty Acid, Dicarboxylate	-0.32	0.00	0.11	0.39	2.02
hydroxy-N6,N6,N6-trimethyllysine*	Amino Acid	Lysine Metabolism	-0.09	0.22	0.70	1.00	0.96
hydroxypalmitoyl sphingomyelin (d18:1/16:0(OH))	Lipid	Sphingomyelins	-0.04	0.49	0.86	1.00	1.13
hypotaurine	Amino Acid	Methionine, Cysteine, SAM and Taurine Metabolism	0.07	0.54	0.89	1.00	0.93
hypoxanthine	Nucleotide	Purine Metabolism, (Hypo)Xanthine/Inosine containing	-0.02	0.28	0.73	1.00	0.64
imidazole lactate	Amino Acid	Histidine Metabolism	0.00	0.85	0.97	1.00	0.73
indoleacetate	Amino Acid	Tryptophan Metabolism	0.00	0.55	0.89	1.00	0.81
indolelactate	Amino Acid	Tryptophan Metabolism	0.02	0.92	0.98	1.00	1.35
inosine	Nucleotide	Purine Metabolism, (Hypo)Xanthine/Inosine containing	0.01	0.62	0.90	1.00	0.30
isobutyrylcarnitine (C4)	Amino Acid	Leucine, Isoleucine and Valine Metabolism	-0.10	0.12	0.63	1.00	1.06
isocitrate	Energy	TCA Cycle	0.06	0.88	0.98	1.00	0.82
isoleucine	Amino Acid	Leucine, Isoleucine and Valine Metabolism	-0.05	0.14	0.64	1.00	1.48
kynurenate	Amino Acid	Tryptophan Metabolism	-0.11	0.07	0.57	1.00	1.09
kynurenine	Amino Acid	Tryptophan Metabolism	-0.06	0.31	0.77	1.00	1.07
lactate	Carbohydrate	Glycolysis, Gluconeogenesis, and Pyruvate Metabolism	-0.01	0.75	0.93	1.00	0.80
lactosyl-N-nervonoyl-sphingosine (d18:1/24:1)*	Lipid	Lactosylceramides (LCER)	0.06	0.60	0.90	1.00	1.03
lactosyl-N-palmitoyl-sphingosine (d18:1/16:0)	Lipid	Lactosylceramides (LCER)	0.05	0.40	0.82	1.00	1.44
laurate (12:0)	Lipid	Medium Chain Fatty Acid	0.13	0.30	0.75	1.00	1.34
laurylcarnitine (C12)	Lipid	Fatty Acid Metabolism(Acyl Carnitine)	0.01	0.92	0.98	1.00	0.12
leucine	Amino Acid	Leucine, Isoleucine and Valine Metabolism	-0.07	0.01	0.32	1.00	1.78
lignoceroyl sphingomyelin (d18:1/24:0)	Lipid	Sphingomyelins	-0.05	0.23	0.70	1.00	1.03
lignoceroylcarnitine (C24)*	Lipid	Fatty Acid Metabolism(Acyl Carnitine)	-0.07	0.22	0.70	1.00	0.84
linoleate (18:2n6)	Lipid	Polyunsaturated Fatty Acid (n3 and n6)	0.06	0.53	0.89	1.00	1.76
linolenate (18:3n3 or 3n6)	Lipid	Polyunsaturated Fatty Acid (n3 and n6)	0.09	0.47	0.84	1.00	1.69
linolenoylcarnitine (C18:3)*	Lipid	Fatty Acid Metabolism(Acyl Carnitine)	-0.01	0.95	0.98	1.00	0.44
linoleoyl-arachidonoyl-glycerol (18:2/20:4) [2]*	Lipid	Diacylglycerol	-0.04	0.41	0.83	1.00	0.74
linoleoylcarnitine (C18:2)*	Lipid	Fatty Acid Metabolism(Acyl Carnitine)	-0.01	0.98	0.99	1.00	0.71
linoleoylcholine*	Lipid	Fatty Acid Metabolism (Acyl Choline)	-0.02	0.98	1.00	1.00	0.96
linoleoyl-linoleoyl-glycerol (18:2/18:2) [1]*	Lipid	Diacylglycerol	0.02	0.98	1.00	1.00	0.48
lysine	Amino Acid	Lysine Metabolism	-0.08	0.01	0.31	1.00	1.79
malate	Energy	TCA Cycle	0.13	0.01	0.31	1.00	1.68
mannitol/sorbitol	Carbohydrate	Fructose, Mannose and Galactose Metabolism	0.00	0.38	0.80	1.00	0.62
mannonate*	Xenobiotics	Food Component/Plant	0.02	0.76	0.93	1.00	0.74
mannose	Carbohydrate	Fructose, Mannose and Galactose Metabolism	-0.02	0.62	0.90	1.00	0.86
margarate (17:0)	Lipid	Long Chain Fatty Acid	0.02	0.81	0.95	1.00	1.95
margaroylcarnitine (C17)*	Lipid	Fatty Acid Metabolism(Acyl Carnitine)	0.04	0.57	0.89	1.00	0.70
methionine	Amino Acid	Methionine, Cysteine, SAM and Taurine Metabolism	-0.04	0.17	0.69	1.00	1.63
methionine sulfone	Amino Acid	Methionine, Cysteine, SAM and Taurine Metabolism	-0.03	0.38	0.80	1.00	0.71
methionine sulfoxide	Amino Acid	Methionine, Cysteine, SAM and Taurine Metabolism	0.01	0.90	0.98	1.00	1.22
methyl glucopyranoside (alpha + beta)	Xenobiotics	Food Component/Plant	-0.15	0.57	0.89	1.00	0.66
methylsuccinate	Amino Acid	Leucine, Isoleucine and Valine Metabolism	0.05	0.53	0.89	1.00	0.73
myo-inositol	Lipid	Inositol Metabolism	-0.04	0.42	0.83	1.00	0.63

myristate (14:0)	Lipid	Long Chain Fatty Acid	0.10	0.43	0.83	1.00	1.93
myristoleate (14:1n5)	Lipid	Long Chain Fatty Acid	0.27	0.04	0.42	1.00	1.57
myristoleoylcarnitine (C14:1)*	Lipid	Fatty Acid Metabolism(Acyl Carnitine)	0.15	0.22	0.70	1.00	0.79
myristoyl dihydrosphingomyelin (d18:0/14:0)*	Lipid	Dihydrosphingomyelins	-0.02	0.54	0.89	1.00	1.06
myristoylcarnitine (C14)	Lipid	Fatty Acid Metabolism(Acyl Carnitine)	0.05	0.69	0.91	1.00	0.30
N,N,N-trimethyl-5-aminovalerate	Amino Acid	Lysine Metabolism	-0.01	0.72	0.92	1.00	0.81
N,N,N-trimethyl-alanylproline betaine (TMAP)	Amino Acid	Urea cycle; Arginine and Proline Metabolism	-0.10	0.03	0.41	1.00	1.31
N1-Methyl-2-pyridone-5-carboxamide	Cofactors and Vitamins	Nicotinate and Nicotinamide Metabolism	0.01	0.41	0.83	1.00	0.74
1-methyladenosine	Nucleotide	Purine Metabolism, Adenine containing	-0.03	0.64	0.90	1.00	1.44
N1-methylinosine	Nucleotide	Purine Metabolism, (Hypo)Xanthine/Inosine containing	0.01	0.74	0.93	1.00	0.59
N2,N2-dimethylguanosine	Nucleotide	Purine Metabolism, Guanine containing	-0.02	0.79	0.94	1.00	0.84
N6,N6,N6-trimethyllysine	Amino Acid	Lysine Metabolism	-0.16	0.33	0.78	1.00	0.84
N6,N6-dimethyllysine	Amino Acid	Lysine Metabolism	0.18	0.08	0.60	1.00	1.47
N6-acetyllysine	Amino Acid	Lysine Metabolism	0.03	0.79	0.94	1.00	1.10
N6-methyllysine	Amino Acid	Lysine Metabolism	0.21	0.27	0.73	1.00	0.82
N-acetyl-2-aminooctanoate*	Lipid	Fatty Acid, Amino	-0.06	0.34	0.79	1.00	0.93
N-acetylalanine	Amino Acid	Alanine and Aspartate Metabolism	0.03	0.46	0.84	1.00	1.61
N-acetylarginine	Amino Acid	Urea cycle; Arginine and Proline Metabolism	-0.18	0.01	0.31	1.00	1.44
N-acetylasparagine	Amino Acid	Alanine and Aspartate Metabolism	-0.04	0.71	0.91	1.00	0.59
N-acetyl-beta-alanine	Nucleotide	Pyrimidine Metabolism, Uracil containing	0.04	0.23	0.70	1.00	1.74
N-acetylglutamate	Amino Acid	Glutamate Metabolism	0.04	0.54	0.89	1.00	1.00
N-acetylglutamine	Amino Acid	Glutamate Metabolism	0.08	0.34	0.78	1.00	1.07
N-acetylglycine	Amino Acid	Glycine, Serine and Threonine Metabolism	0.00	0.76	0.93	1.00	0.11
N-acetyl-isoputreanine*	Amino Acid	Polyamine Metabolism	0.13	0.17	0.69	1.00	1.13
N-acetylmethionine	Amino Acid	Methionine, Cysteine, SAM and Taurine Metabolism	0.06	0.20	0.69	1.00	1.55
N-acetylputrescine	Amino Acid	Polyamine Metabolism	-0.04	0.49	0.86	1.00	0.93
N-acetylserine	Amino Acid	Glycine, Serine and Threonine Metabolism	-0.02	0.88	0.98	1.00	0.76
N-acetyltaurine	Amino Acid	Methionine, Cysteine, SAM and Taurine Metabolism	0.02	0.76	0.93	1.00	0.60
N-acetyltryptophan	Amino Acid	Tryptophan Metabolism	-0.01	0.61	0.90	1.00	1.02
N-acetylvaline	Amino Acid	Leucine, Isoleucine and Valine Metabolism	0.02	0.66	0.90	1.00	1.63
N-behenoyl-sphingadienine (d18:2/22:0)*	Lipid	Ceramides	-0.12	0.02	0.37	1.00	1.40
N-delta-acetylornithine	Amino Acid	Urea cycle; Arginine and Proline Metabolism	0.04	0.99	1.00	1.00	0.81
N-formylmethionine	Amino Acid	Methionine, Cysteine, SAM and Taurine Metabolism	0.01	0.71	0.91	1.00	1.21
nicotinamide	Cofactors and Vitamins	Nicotinate and Nicotinamide Metabolism	-0.06	0.19	0.69	1.00	0.81
N-methylpipecolate	Xenobiotics	Bacterial/Fungal	0.32	0.11	0.63	1.00	1.32
N-methylproline	Amino Acid	Urea cycle; Arginine and Proline Metabolism	0.05	0.68	0.90	1.00	0.97
N-oleoylserine	Lipid	Endocannabinoid	0.18	0.00	0.15	1.00	2.13
nonadecanoate (19:0)	Lipid	Long Chain Fatty Acid	0.01	0.70	0.91	1.00	1.60
nonanoylcarnitine (C9)	Lipid	Fatty Acid Metabolism(Acyl Carnitine)	0.01	0.64	0.90	1.00	0.30
N-palmitoylglycine	Lipid	Fatty Acid Metabolism(Acyl Glycine)	0.08	0.25	0.72	1.00	0.92
N-palmitoyl-heptadecasphingosine (d17:1/16:0)*	Lipid	Ceramides	-0.04	0.35	0.79	1.00	0.75
N-palmitoyl-sphinganine (d18:0/16:0)	Lipid	Dihydroceramides	-0.01	0.83	0.97	1.00	0.52
N-palmitoyl-sphingosine (d18:1/16:0)	Lipid	Ceramides	-0.02	0.58	0.89	1.00	0.76
N-stearoyl-sphingadienine (d18:2/18:0)*	Lipid	Ceramides	-0.11	0.07	0.57	1.00	1.15
N-stearoyl-sphingosine (d18:1/18:0)*	Lipid	Ceramides	-0.06	0.27	0.73	1.00	0.76
octadecadienedioate (C18:2-DC)*	Lipid	Fatty Acid, Dicarboxylate	0.15	0.78	0.94	1.00	0.61
octadecanedioate (C18)	Lipid	Fatty Acid, Dicarboxylate	0.01	0.91	0.98	1.00	0.51
octadecanedioylcarnitine (C18-DC)*	Lipid	Fatty Acid Metabolism(Acyl Carnitine)	-0.15	0.15	0.66	1.00	0.80

octadecenedioate (C18:1-DC)*	Lipid	Fatty Acid, Dicarboxylate	0.06	0.95	0.98	1.00	0.43
octadecenedioylcarnitine (C18:1-DC)*	Lipid	Fatty Acid Metabolism(Acyl Carnitine)	0.04	0.67	0.90	1.00	0.52
octanoylcarnitine (C8)	Lipid	Fatty Acid Metabolism(Acyl Carnitine)	-0.01	0.70	0.91	1.00	0.19
oleate/vaccenate (18:1)	Lipid	Long Chain Fatty Acid	0.06	0.53	0.89	1.00	2.10
oleoyl ethanolamide	Lipid	Endocannabinoid	0.14	0.05	0.48	1.00	1.39
oleoyl-arachidonoyl-glycerol (18:1/20:4) [2]*	Lipid	Diacylglycerol	-0.01	1.00	1.00	1.00	0.39
oleoylcarnitine (C18)	Lipid	Fatty Acid Metabolism(Acyl Carnitine)	0.05	0.38	0.80	1.00	0.50
oleoylcholine	Lipid	Fatty Acid Metabolism (Acyl Choline)	0.01	0.64	0.90	1.00	0.96
oleoyl-linoleoyl-glycerol (18:1/18:2) [1]	Lipid	Diacylglycerol	0.03	0.56	0.89	1.00	0.71
oleoyl-linoleoyl-glycerol (18:1/18:2) [2]	Lipid	Diacylglycerol	0.04	0.50	0.86	1.00	0.92
oleoyl-oleoyl-glycerol (18:1/18:1) [1]*	Lipid	Diacylglycerol	0.04	0.36	0.79	1.00	0.72
oleoyl-oleoyl-glycerol (18:1/18:1) [2]*	Lipid	Diacylglycerol	0.06	0.27	0.72	1.00	0.83
ornithine	Amino Acid	Urea cycle; Arginine and Proline Metabolism	-0.04	0.22	0.70	1.00	1.45
orotate	Nucleotide	Pyrimidine Metabolism, Orotate containing	0.17	0.17	0.69	1.00	0.94
O-sulfo-L-tyrosine	Xenobiotics	Chemical	-0.04	0.30	0.75	1.00	0.94
oxalate (ethanedioate)	Cofactors and Vitamins	Ascorbate and Aldarate Metabolism	-0.01	0.64	0.90	1.00	1.00
palmitate (16:0)	Lipid	Long Chain Fatty Acid	0.04	0.63	0.90	1.00	2.14
palmitoleate (16:1n7)	Lipid	Long Chain Fatty Acid	0.31	0.02	0.37	1.00	1.89
palmitoleoylcarnitine (C16:1)*	Lipid	Fatty Acid Metabolism(Acyl Carnitine)	0.11	0.13	0.64	1.00	0.90
palmitoleoyl-linoleoyl-glycerol (16:1/18:2) [1]*	Lipid	Diacylglycerol	0.11	0.15	0.66	1.00	0.92
palmitoyl dihydrosphingomyelin (d18:0/16:0)*	Lipid	Dihydrosphingomyelins	0.01	0.95	0.98	1.00	1.43
palmitoyl ethanolamide	Lipid	Endocannabinoid	0.04	0.57	0.89	1.00	1.07
palmitoyl sphingomyelin (d18:1/16:0)	Lipid	Sphingomyelins	-0.03	0.36	0.79	1.00	1.55
palmitoylcarnitine (C16)	Lipid	Fatty Acid Metabolism(Acyl Carnitine)	-0.01	0.66	0.90	1.00	0.28
palmitoylcholine	Lipid	Fatty Acid Metabolism (Acyl Choline)	-0.05	0.90	0.98	1.00	0.79
palmitoyl-myristoyl-glycerol (16:0/14:0) [1]*	Lipid	Diacylglycerol	-0.12	0.36	0.79	1.00	0.62
palmitoyl-oleoyl-glycerol (16:0/18:1) [1]*	Lipid	Diacylglycerol	-0.01	0.94	0.98	1.00	0.27
palmitoyl-oleoyl-glycerol (16:0/18:1) [2]*	Lipid	Diacylglycerol	0.00	0.75	0.93	1.00	0.34
palmitoyl-palmitoyl-glycerol (16:0/16:0) [2]*	Lipid	Diacylglycerol	-0.04	0.84	0.97	1.00	0.34
pantothenate (Vitamin B5)	Cofactors and Vitamins	Pantothenate and CoA Metabolism	-0.10	0.12	0.63	1.00	0.94
paraxanthine	Xenobiotics	Xanthine Metabolism	0.23	0.22	0.70	1.00	1.03
p-cresol sulfate	Xenobiotics	Benzoate Metabolism	0.10	0.70	0.91	1.00	0.61
pelargonate (9:0)	Lipid	Medium Chain Fatty Acid	-0.03	0.64	0.90	1.00	0.98
pentadecanoate (15:0)	Lipid	Long Chain Fatty Acid	0.05	0.72	0.92	1.00	1.56
perfluorooctanesulfonate (PFOS)	Xenobiotics	Chemical	-0.11	0.06	0.53	1.00	1.19
perfluorooctanoate (PFOA)*	Xenobiotics	Chemical	-0.20	0.01	0.29	1.00	1.59
phenol sulfate	Amino Acid	Tyrosine Metabolism	0.06	0.74	0.93	1.00	0.35
phenylacetylglutamine	Peptide	Acetylated Peptides	0.07	0.48	0.86	1.00	0.90
phenylalanine	Amino Acid	Phenylalanine Metabolism	-0.04	0.15	0.66	1.00	1.73
phenylpyruvate	Amino Acid	Phenylalanine Metabolism	-0.01	0.92	0.98	1.00	1.45
phytanate	Xenobiotics	Food Component/Plant	-0.01	0.79	0.94	1.00	0.48
pipecolate	Amino Acid	Lysine Metabolism	-0.16	0.20	0.69	1.00	0.78
piperine	Xenobiotics	Food Component/Plant	-0.06	0.96	0.99	1.00	0.51
pregnanediol-3-glucuronide	Lipid	Progestin Steroids	-0.13	0.26	0.72	1.00	0.61
pregnen-diol disulfate*	Lipid	Pregnenolone Steroids	-0.14	0.50	0.86	1.00	0.44
pregnenediol sulfate (C21H34O5S)*	Lipid	Pregnenolone Steroids	-0.18	0.15	0.67	1.00	0.81
pregnenetriol disulfate*	Lipid	Pregnenolone Steroids	-0.16	0.33	0.78	1.00	0.63
pregnenetriol sulfate*	Lipid	Pregnenolone Steroids	-0.19	0.10	0.63	1.00	0.97

proline	Amino Acid	Urea cycle; Arginine and Proline Metabolism	0.00	0.94	0.98	1.00	1.08
propionylcarnitine (C3)	Lipid	Fatty Acid Metabolism (also BCAA Metabolism)	-0.09	0.22	0.70	1.00	1.40
propionylglycine (C3)	Lipid	Fatty Acid Metabolism (also BCAA Metabolism)	-0.11	0.34	0.78	1.00	0.86
propyl 4-hydroxybenzoate sulfate	Xenobiotics	Benzoate Metabolism	0.08	0.57	0.89	1.00	0.98
pseudouridine	Nucleotide	Pyrimidine Metabolism, Uracil containing	-0.03	0.42	0.83	1.00	0.81
pyridoxate	Cofactors and Vitamins	Vitamin B6 Metabolism	-0.32	0.01	0.31	1.00	1.47
pyroglutamine*	Amino Acid	Glutamate Metabolism	-0.01	0.76	0.93	1.00	0.26
pyruvate	Carbohydrate	Glycolysis, Gluconeogenesis, and Pyruvate Metabolism	-0.03	0.63	0.90	1.00	0.46
quinate	Xenobiotics	Food Component/Plant	0.23	0.15	0.66	1.00	1.47
quinolinate	Cofactors and Vitamins	Nicotinate and Nicotinamide Metabolism	-0.10	0.28	0.73	1.00	0.71
retinol (Vitamin A)	Cofactors and Vitamins	Vitamin A Metabolism	-0.10	0.03	0.41	1.00	1.37
ribitol	Carbohydrate	Pentose Metabolism	-0.02	0.67	0.90	1.00	0.50
ribonate (ribonolactone)	Carbohydrate	Pentose Metabolism	-0.01	0.49	0.86	1.00	0.45
salicylate	Xenobiotics	Drug - Topical Agents	0.64	0.76	0.93	1.00	0.20
sarcosine	Amino Acid	Glycine, Serine and Threonine Metabolism	0.02	0.94	0.98	1.00	1.20
sebacate (C10-DC)	Lipid	Fatty Acid, Dicarboxylate	-0.03	0.66	0.90	1.00	0.38
serine	Amino Acid	Glycine, Serine and Threonine Metabolism	-0.03	0.38	0.80	1.00	1.30
S-methylcysteine	Amino Acid	Methionine, Cysteine, SAM and Taurine Metabolism	0.05	0.46	0.84	1.00	1.04
S-methylcysteine sulfoxide	Amino Acid	Methionine, Cysteine, SAM and Taurine Metabolism	0.01	0.68	0.90	1.00	0.92
sphinganine	Lipid	Sphingolipid Synthesis	0.05	0.44	0.84	1.00	1.51
sphinganine-1-phosphate	Lipid	Sphingolipid Synthesis	0.00	0.88	0.98	1.00	1.34
sphingomyelin (d17:1/14:0, d16:1/15:0)*	Lipid	Sphingomyelins	-0.09	0.18	0.69	1.00	0.92
sphingomyelin (d17:1/16:0, d18:1/15:0, d16:1/17:0)*	Lipid	Sphingomyelins	-0.06	0.17	0.69	1.00	1.22
sphingomyelin (d17:2/16:0, d18:2/15:0)*	Lipid	Sphingomyelins	-0.11	0.04	0.44	1.00	1.30
sphingomyelin (d18:0/18:0, d19:0/17:0)*	Lipid	Dihydrosphingomyelins	-0.08	0.38	0.80	1.00	0.72
sphingomyelin (d18:0/20:0, d16:0/22:0)*	Lipid	Dihydrosphingomyelins	-0.16	0.11	0.63	1.00	0.98
sphingomyelin (d18:1/14:0, d16:1/16:0)*	Lipid	Sphingomyelins	-0.06	0.13	0.64	1.00	1.23
sphingomyelin (d18:1/17:0, d17:1/18:0, d19:1/16:0)	Lipid	Sphingomyelins	-0.11	0.01	0.31	1.00	1.54
sphingomyelin (d18:1/18:1, d18:2/18:0)	Lipid	Sphingomyelins	-0.09	0.02	0.36	1.00	1.51
sphingomyelin (d18:1/19:0, d19:1/18:0)*	Lipid	Sphingomyelins	-0.12	0.01	0.31	1.00	1.52
sphingomyelin (d18:1/20:0, d16:1/22:0)*	Lipid	Sphingomyelins	-0.09	0.00	0.24	1.00	1.75
sphingomyelin (d18:1/20:1, d18:2/20:0)*	Lipid	Sphingomyelins	-0.12	0.01	0.24	1.00	1.69
sphingomyelin (d18:1/21:0, d17:1/22:0, d16:1/23:0)*	Lipid	Sphingomyelins	-0.16	0.00	0.17	1.00	1.72
sphingomyelin (d18:1/22:1, d18:2/22:0, d16:1/24:1)*	Lipid	Sphingomyelins	-0.10	0.00	0.15	1.00	1.99
sphingomyelin (d18:1/22:2, d18:2/22:1, d16:1/24:2)*	Lipid	Sphingomyelins	-0.08	0.05	0.48	1.00	1.34
sphingomyelin (d18:1/24:1, d18:2/24:0)*	Lipid	Sphingomyelins	-0.02	0.42	0.83	1.00	1.48
sphingomyelin (d18:1/25:0, d19:0/24:1, d20:1/23:0, d19:1/2	Lipid	Sphingomyelins	-0.16	0.02	0.38	1.00	1.32
sphingomyelin (d18:2/14:0, d18:1/14:1)*	Lipid	Sphingomyelins	-0.09	0.10	0.63	1.00	1.08
sphingomyelin (d18:2/16:0, d18:1/16:1)*	Lipid	Sphingomyelins	-0.02	0.57	0.89	1.00	1.22
sphingomyelin (d18:2/18:1)*	Lipid	Sphingomyelins	-0.03	0.63	0.90	1.00	1.10
sphingomyelin (d18:2/21:0, d16:2/23:0)*	Lipid	Sphingomyelins	-0.13	0.01	0.26	1.00	1.63
sphingomyelin (d18:2/23:0, d18:1/23:1, d17:1/24:1)*	Lipid	Sphingomyelins	-0.11	0.03	0.41	1.00	1.37
sphingomyelin (d18:2/23:1)*	Lipid	Sphingomyelins	-0.09	0.04	0.44	1.00	1.38
sphingomyelin (d18:2/24:1, d18:1/24:2)*	Lipid	Sphingomyelins	-0.04	0.15	0.66	1.00	1.47
sphingomyelin (d18:2/24:2)*	Lipid	Sphingomyelins	-0.05	0.24	0.71	1.00	1.09
sphingosine 1-phosphate	Lipid	Sphingosines	0.01	0.90	0.98	1.00	1.63
stearate (18:0)	Lipid	Long Chain Fatty Acid	0.01	0.96	0.99	1.00	1.64
stearidonate (18:4n3)	Lipid	Polyunsaturated Fatty Acid (n3 and n6)	-0.03	0.84	0.97	1.00	1.65

stearoyl sphingomyelin (d18:1/18:0)	Lipid	Sphingomyelins	-0.12	0.00	0.17	1.00	1.82
stearoyl-arachidonoyl-glycerol (18:0/20:4) [1]*	Lipid	Diacylglycerol	0.04	0.32	0.78	1.00	1.09
stearoylcarnitine (C18)	Lipid	Fatty Acid Metabolism(Acyl Carnitine)	-0.05	0.17	0.69	1.00	0.78
stearoylcholine*	Lipid	Fatty Acid Metabolism (Acyl Choline)	-0.02	0.99	1.00	1.00	1.01
succinate	Energy	TCA Cycle	0.05	0.19	0.69	1.00	1.89
succinimide	Xenobiotics	Chemical	-0.09	0.18	0.69	1.00	0.93
sulfate*	Xenobiotics	Chemical	-0.08	0.03	0.41	1.00	1.51
tartronate (hydroxymalonate)	Xenobiotics	Food Component/Plant	-0.01	0.65	0.90	1.00	1.10
taurine	Amino Acid	Methionine, Cysteine, SAM and Taurine Metabolism	0.00	0.75	0.93	1.00	1.43
taurocholate	Lipid	Primary Bile Acid Metabolism	0.88	0.18	0.69	1.00	1.25
taurocholenate sulfate*	Lipid	Secondary Bile Acid Metabolism	-0.10	0.54	0.89	1.00	0.35
tetradecadienedioate (C14:2-DC)*	Lipid	Fatty Acid, Dicarboxylate	0.20	0.12	0.64	1.00	1.11
tetradecadienoate (14:2)*	Lipid	Polyunsaturated Fatty Acid (n3 and n6)	0.17	0.13	0.64	1.00	1.24
tetradecanedioate (C14)	Lipid	Fatty Acid, Dicarboxylate	0.16	0.34	0.78	1.00	1.07
theobromine	Xenobiotics	Xanthine Metabolism	0.00	0.95	0.98	1.00	0.23
theophylline	Xenobiotics	Xanthine Metabolism	0.23	0.12	0.63	1.00	1.18
thioproline	Xenobiotics	Chemical	-0.02	0.49	0.86	1.00	1.10
threonate	Cofactors and Vitamins	Ascorbate and Aldarate Metabolism	0.01	0.92	0.98	1.00	0.96
threonine	Amino Acid	Glycine, Serine and Threonine Metabolism	-0.04	0.26	0.72	1.00	1.26
thyroxine	Amino Acid	Tyrosine Metabolism	-0.01	0.74	0.93	1.00	0.46
hydroxyproline	Amino Acid	Urea cycle; Arginine and Proline Metabolism	-0.07	0.21	0.70	1.00	0.81
tricosanoyl sphingomyelin (d18:1/23:0)*	Lipid	Sphingomyelins	-0.07	0.08	0.58	1.00	1.29
tridecenedioate (C13:1-DC)*	Lipid	Fatty Acid, Dicarboxylate	-0.04	0.35	0.79	1.00	0.87
trigonelline (N'-methylnicotinate)	Cofactors and Vitamins	Nicotinate and Nicotinamide Metabolism	0.21	0.26	0.72	1.00	1.11
trimethylamine N-oxide	Lipid	Phospholipid Metabolism	-0.10	0.34	0.78	1.00	0.62
tryptophan	Amino Acid	Tryptophan Metabolism	-0.06	0.10	0.63	1.00	1.72
tyrosine	Amino Acid	Tyrosine Metabolism	-0.04	0.20	0.69	1.00	1.42
urate	Nucleotide	Purine Metabolism, (Hypo)Xanthine/Inosine containing	-0.07	0.14	0.65	1.00	1.28
urea	Amino Acid	Urea cycle; Arginine and Proline Metabolism	-0.08	0.18	0.69	1.00	1.08
uridine	Nucleotide	Pyrimidine Metabolism, Uracil containing	-0.07	0.03	0.41	1.00	1.58
valine	Amino Acid	Leucine, Isoleucine and Valine Metabolism	-0.09	0.00	0.24	1.00	1.81
vanillylmandelate (VMA)	Amino Acid	Tyrosine Metabolism	0.05	0.44	0.84	1.00	1.03
xanthine	Nucleotide	Purine Metabolism, (Hypo)Xanthine/Inosine containing	0.06	0.46	0.84	1.00	0.91
ximenoylcarnitine (C26:1)*	Lipid	Fatty Acid Metabolism(Acyl Carnitine)	-0.03	0.73	0.93	1.00	0.59
X - 09789			-0.15	0.23	0.70	1.00	0.74
X - 11261			-0.18	0.18	0.69	1.00	0.85
X - 11308			-0.06	0.42	0.83	1.00	0.56
X - 11372			0.12	0.86	0.98	1.00	0.81
X - 11381			-0.11	0.15	0.66	1.00	1.16
X - 11444			0.03	0.72	0.92	1.00	0.50
X - 11470			0.07	0.53	0.89	1.00	0.83
X - 11530			0.08	0.99	1.00	1.00	0.59
X - 11787			-0.03	0.33	0.78	1.00	1.69
X - 11795			-0.23	0.08	0.59	1.00	1.02
X - 12026			-0.07	0.19	0.69	1.00	0.85
X - 12063			-0.44	0.00	0.14	0.83	2.03
X - 12100			-0.02	0.63	0.90	1.00	0.63
X - 12101			-0.05	0.83	0.97	1.00	0.12

X - 12104	-0.09	0.21	0.70	1.00	1.05
X - 12206	0.01	0.94	0.98	1.00	1.05
X - 12411	0.11	0.68	0.90	1.00	1.06
X - 12462	-0.11	0.08	0.59	1.00	1.10
X - 12472	0.29	0.09	0.60	1.00	1.06
X - 12524	0.00	0.86	0.98	1.00	0.57
X - 12739	0.17	0.23	0.70	1.00	0.70
X - 12844	-0.04	0.68	0.90	1.00	0.88
X - 12846	-0.16	0.17	0.69	1.00	0.86
X - 13431	-0.15	0.03	0.42	1.00	1.28
X - 13866	-0.17	0.22	0.70	1.00	0.64
X - 14056	-0.02	0.54	0.89	1.00	0.51
X - 14939	0.08	0.85	0.97	1.00	0.55
X - 15245	0.10	0.25	0.72	1.00	0.67
X - 15469	0.05	0.86	0.98	1.00	0.17
X - 15486	0.02	0.68	0.90	1.00	0.41
X - 15492	-0.11	0.42	0.83	1.00	0.75
X - 15503	-0.10	0.16	0.69	1.00	0.93
X - 16087	-0.13	0.03	0.41	1.00	1.38
X - 16580	0.03	0.66	0.90	1.00	0.27
X - 16938	-0.01	0.71	0.91	1.00	0.94
X - 16944	-0.06	0.73	0.93	1.00	0.49
X - 17335	0.03	0.93	0.98	1.00	0.32
X - 17337	-0.18	0.03	0.41	1.00	1.28
X - 17340	-0.19	0.09	0.61	1.00	1.10
X - 17357	-0.08	0.32	0.78	1.00	0.74
X - 17654	-0.02	0.57	0.89	1.00	0.57
X - 18779	0.06	0.95	0.98	1.00	1.34
X - 18913	-0.06	0.54	0.89	1.00	0.37
X - 18921	-0.13	0.42	0.83	1.00	0.52
X - 19141	-0.11	0.21	0.70	1.00	0.79
X - 21258	-0.08	0.62	0.90	1.00	0.36
X - 21286	-0.12	0.60	0.90	1.00	0.58
X - 21310	-0.03	0.78	0.94	1.00	1.20
X - 21319	-0.03	0.53	0.89	1.00	0.37
X - 21353	0.07	0.69	0.91	1.00	0.76
X - 21383	-0.04	0.26	0.72	1.00	0.70
X - 21411	0.02	0.78	0.94	1.00	0.74
X - 21628	-0.08	0.18	0.69	1.00	0.89
X - 21736	-0.01	0.78	0.94	1.00	0.41
X - 21785	-0.01	0.79	0.94	1.00	0.81
X - 21796	-0.05	0.53	0.89	1.00	0.76
X - 22162	-0.08	0.33	0.78	1.00	0.66
X - 22519	0.03	0.94	0.98	1.00	0.11
X - 22775	-0.03	0.29	0.74	1.00	1.81
X - 23314	-0.06	0.76	0.93	1.00	0.30
X - 23593	0.10	0.19	0.69	1.00	1.49
X - 23639	0.09	0.27	0.72	1.00	1.80

X - 23974			-0.02	0.55	0.89	1.00	0.70
X - 24106 - retired for palmitoyl-sphingosine-phosphoethanolamine (d18:1/16:0)			-0.03	0.48	0.86	1.00	0.80
X - 24435			0.14	0.29	0.75	1.00	0.77
X - 24549			-0.10	0.36	0.79	1.00	1.03
X - 24699			-0.05	0.45	0.84	1.00	0.96
X - 24765			-0.08	0.01	0.31	1.00	1.71
X - 24952			-0.04	0.56	0.89	1.00	0.37
X - 25422			0.37	0.11	0.63	1.00	1.07
1-(1-enyl-oleoyl)-GPE (P-18:1)*	Lipid	Lysoplasmalogen	-0.09	0.12	0.64	1.00	0.83
1,2,3-benzenetriol sulfate (2)	Xenobiotics	Chemical	-0.12	0.19	0.69	1.00	0.76
1,3,7-trimethylurate	Xenobiotics	Xanthine Metabolism	0.16	0.94	0.98	1.00	0.08
1,3-dimethylurate	Xenobiotics	Xanthine Metabolism	0.24	0.13	0.64	1.00	1.44
12,13-DiHOME	Lipid	Fatty Acid, Dihydroxy	0.09	0.95	0.98	1.00	0.30
12-HETE	Lipid	Eicosanoid	0.07	0.28	0.73	1.00	0.69
1-arachidonoyl-GPA (20:4)	Lipid	Lysophospholipid	0.07	0.65	0.90	1.00	0.42
1-carboxyethylisoleucine	Amino Acid	Leucine, Isoleucine and Valine Metabolism	0.15	0.92	0.98	1.00	0.44
1-carboxyethylleucine	Amino Acid	Leucine, Isoleucine and Valine Metabolism	0.10	0.44	0.84	1.00	0.84
1-carboxyethylvaline	Amino Acid	Leucine, Isoleucine and Valine Metabolism	0.07	0.73	0.93	1.00	0.31
1-heptadecenoylglycerol (17:1)*	Lipid	Monoacylglycerol	0.02	0.34	0.78	1.00	0.47
1H-indole-7-acetic acid	Xenobiotics	Bacterial/Fungal	-0.06	0.95	0.98	1.00	0.28
1-lignoceroyl-GPC (24:0)	Lipid	Lysophospholipid	-0.01	0.89	0.98	1.00	0.57
1-linolenoylglycerol (18:3)	Lipid	Monoacylglycerol	-0.01	0.93	0.98	1.00	0.32
1-linoleoyl-2-arachidonoyl-GPC (18:2/20:4n6)*	Lipid	Phosphatidylcholine (PC)	0.02	0.96	0.99	1.00	0.20
1-linoleoyl-GPG (18:2)*	Lipid	Lysophospholipid	0.00	0.08	0.58	1.00	1.23
1-methyl-5-imidazoleacetate	Amino Acid	Histidine Metabolism	-0.14	0.61	0.90	1.00	0.47
1-methylurate	Xenobiotics	Xanthine Metabolism	0.27	0.27	0.73	1.00	1.00
1-methylxanthine	Xenobiotics	Xanthine Metabolism	0.27	0.34	0.78	1.00	0.67
1-oleoyl-2-arachidonoyl-GPE (18:1/20:4)*	Lipid	Phosphatidylethanolamine (PE)	0.03	0.49	0.86	1.00	0.49
1-oleoyl-GPG (18:1)*	Lipid	Lysophospholipid	0.19	0.26	0.72	1.00	1.34
1-palmitoleoyl-2-linolenoyl-GPC (16:1/18:3)*	Lipid	Phosphatidylcholine (PC)	0.08	0.32	0.78	1.00	0.72
1-palmitoyl-GPG (16:0)*	Lipid	Lysophospholipid	0.04	0.58	0.89	1.00	0.36
1-stearoyl-2-oleoyl-GPE (18:0/18:1)	Lipid	Phosphatidylethanolamine (PE)	-0.15	0.75	0.93	1.00	0.37
1-stearoyl-2-oleoyl-GPS (18:0/18:1)	Lipid	Phosphatidylserine (PS)	-0.19	0.62	0.90	1.00	0.32
1-stearoyl-GPG (18:0)	Lipid	Lysophospholipid	-0.05	0.33	0.78	1.00	0.88
2,3-dihydroxy-2-methylbutyrate	Amino Acid	Leucine, Isoleucine and Valine Metabolism	0.11	0.58	0.90	1.00	0.59
2,3-dihydroxyisovalerate	Xenobiotics	Food Component/Plant	-0.24	0.07	0.58	1.00	1.17
2-aminoadipate	Amino Acid	Lysine Metabolism	-0.14	0.32	0.78	1.00	0.66
2-aminoheptanoate	Lipid	Fatty Acid, Amino	0.02	0.61	0.90	1.00	0.79
2-aminooctanoate	Lipid	Fatty Acid, Amino	-0.04	0.88	0.98	1.00	0.58
2-arachidonoylglycerol (20:4)	Lipid	Monoacylglycerol	-0.04	0.48	0.86	1.00	0.58
2'-deoxyuridine	Nucleotide	Pyrimidine Metabolism, Uracil containing	0.09	0.14	0.66	1.00	1.36
2-docosahexaenoylglycerol (22:6)*	Lipid	Monoacylglycerol	-0.02	0.24	0.71	1.00	0.69
2-hydroxybehenate	Lipid	Fatty Acid, Monohydroxy	0.03	0.96	0.99	1.00	0.03
2-hydroxyhippurate (salicylurate)	Xenobiotics	Benzoate Metabolism	0.47	0.80	0.95	1.00	0.20
2-hydroxyphenylacetate	Amino Acid	Phenylalanine Metabolism	-0.14	0.11	0.63	1.00	0.93
2-methylbutyrylcarnitine (C5)	Amino Acid	Leucine, Isoleucine and Valine Metabolism	-0.08	0.10	0.63	1.00	1.01
2-myristoylglycerol (14:0)	Lipid	Monoacylglycerol	-0.10	0.78	0.94	1.00	0.40
2-naphthol sulfate	Xenobiotics	Chemical	0.12	0.61	0.90	1.00	0.88

2'-O-methyluridine	Nucleotide	Pyrimidine Metabolism, Uracil containing	0.05	0.12	0.64	1.00	1.74
2-oxoarginine*	Amino Acid	Urea cycle; Arginine and Proline Metabolism	-0.09	0.37	0.80	1.00	0.87
2-palmitoleoylglycerol (16:1)*	Lipid	Monoacylglycerol	0.11	0.20	0.69	1.00	0.74
2-palmitoleoyl-GPC* (16:1)*	Lipid	Lysophospholipid	0.33	0.24	0.70	1.00	1.00
3-(3-amino-3-carboxypropyl)uridine*	Nucleotide	Pyrimidine Metabolism, Uracil containing	-0.04	0.22	0.70	1.00	0.87
3-(3-hydroxyphenyl)propionate	Xenobiotics	Benzoate Metabolism	0.32	0.67	0.90	1.00	0.34
3-(3-hydroxyphenyl)propionate sulfate	Xenobiotics	Benzoate Metabolism	0.34	0.93	0.98	1.00	0.20
3,4-methyleneheptanoate	Xenobiotics	Food Component/Plant	0.13	0.57	0.89	1.00	0.54
3,7-dimethylurate	Xenobiotics	Xanthine Metabolism	-0.12	0.28	0.73	1.00	0.62
3-ethylcatechol sulfate (1)	Xenobiotics	Food Component/Plant	0.04	0.80	0.94	1.00	0.36
3-hydroxyadipate*	Lipid	Fatty Acid, Dicarboxylate	0.23	0.17	0.69	1.00	0.92
3-hydroxybutyrate (BHBA)	Lipid	Ketone Bodies	0.27	0.18	0.69	1.00	1.09
3-hydroxybutyroylglycine	Lipid	Fatty Acid Metabolism(Acyl Glycine)	0.14	0.15	0.67	1.00	0.85
3-hydroxybutyrylcarnitine (1)	Lipid	Fatty Acid Metabolism(Acyl Carnitine)	0.03	0.75	0.93	1.00	0.49
3-hydroxybutyrylcarnitine (2)	Lipid	Fatty Acid Metabolism(Acyl Carnitine)	-0.04	0.11	0.63	1.00	0.87
3-hydroxyhippurate	Xenobiotics	Benzoate Metabolism	-0.23	0.47	0.85	1.00	0.47
3-hydroxyhippurate sulfate	Xenobiotics	Benzoate Metabolism	0.08	0.84	0.97	1.00	0.47
3-hydroxyisobutyrate	Amino Acid	Leucine, Isoleucine and Valine Metabolism	-0.02	0.69	0.91	1.00	0.50
3-hydroxysebacate	Lipid	Fatty Acid, Monohydroxy	0.57	0.22	0.70	1.00	1.00
3-hydroxystachydrine*	Xenobiotics	Food Component/Plant	0.24	0.34	0.78	1.00	1.29
3-methoxytyramine sulfate	Amino Acid	Tyrosine Metabolism	0.17	0.49	0.86	1.00	0.89
3-methoxytyrosine	Amino Acid	Tyrosine Metabolism	1.02	0.27	0.73	1.00	0.68
3-methyl catechol sulfate (1)	Xenobiotics	Benzoate Metabolism	-0.01	0.57	0.89	1.00	0.45
3-methyladipate	Lipid	Fatty Acid, Dicarboxylate	-0.08	0.80	0.94	1.00	0.16
3-methylglutarylcarnitine (2)	Amino Acid	Leucine, Isoleucine and Valine Metabolism	-0.16	0.13	0.64	1.00	0.92
3-methylxanthine	Xenobiotics	Xanthine Metabolism	0.31	0.02	0.37	1.00	1.67
3-phenylpropionate (hydrocinnamate)	Xenobiotics	Benzoate Metabolism	-0.05	0.49	0.86	1.00	0.62
3-sulfo-L-alanine	Amino Acid	Methionine, Cysteine, SAM and Taurine Metabolism	0.29	0.15	0.66	1.00	1.26
4-guanidinobutanoate	Amino Acid	Guanidino and Acetamido Metabolism	-0.13	0.62	0.90	1.00	0.71
4-hydroxychlorothalonil	Xenobiotics	Chemical	-0.03	0.74	0.93	1.00	0.30
4-hydroxycoumarin	Xenobiotics	Drug - Cardiovascular	0.08	0.66	0.90	1.00	0.47
4-methoxyphenol sulfate	Amino Acid	Tyrosine Metabolism	-0.40	0.03	0.41	1.00	1.26
4-methyl-2-oxopentanoate	Amino Acid	Leucine, Isoleucine and Valine Metabolism	-0.08	0.12	0.63	1.00	1.00
4-methylguaiacol sulfate	Xenobiotics	Benzoate Metabolism	-0.32	0.21	0.70	1.00	0.89
5-(galactosylhydroxy)-L-lysine	Amino Acid	Lysine Metabolism	-0.07	0.30	0.75	1.00	0.66
5,6-dihydrouracil	Nucleotide	Pyrimidine Metabolism, Uracil containing	-0.03	0.56	0.89	1.00	0.71
5-acetylamino-6-formylamino-3-methyluracil	Xenobiotics	Xanthine Metabolism	0.11	0.40	0.82	1.00	0.73
5alpha-androstan-3alpha,17beta-diol monosulfate (1)	Lipid	Androgenic Steroids	-0.46	0.00	0.16	1.00	1.77
5alpha-androstan-3beta,17beta-diol disulfate	Lipid	Androgenic Steroids	-0.48	0.00	0.11	0.43	2.08
5alpha-pregnan-3beta,20alpha-diol disulfate	Lipid	Progestin Steroids	-0.27	0.43	0.83	1.00	0.54
5alpha-pregnan-3beta,20alpha-diol monosulfate (2)	Lipid	Progestin Steroids	-0.27	0.21	0.70	1.00	0.69
5alpha-pregnan-3beta,20beta-diol monosulfate (1)	Lipid	Progestin Steroids	-0.29	0.42	0.83	1.00	0.60
5-dodecenoylcarnitine (C12:1)	Lipid	Fatty Acid Metabolism(Acyl Carnitine)	0.21	0.05	0.48	1.00	1.20
5-hydroxyhexanoate	Lipid	Fatty Acid, Monohydroxy	0.05	0.15	0.67	1.00	1.21
5-hydroxyindole sulfate	Amino Acid	Tryptophan Metabolism	-0.16	0.84	0.97	1.00	0.74
6-hydroxyindole sulfate	Xenobiotics	Chemical	0.05	0.46	0.84	1.00	1.05
7-methylxanthine	Xenobiotics	Xanthine Metabolism	0.15	0.28	0.73	1.00	0.77
9,10-DiHOME	Lipid	Fatty Acid, Dihydroxy	0.02	0.93	0.98	1.00	0.08

adenine	Nucleotide	Purine Metabolism, Adenine containing	-0.08	0.95	0.98	1.00	0.70
adipoylcarnitine (C6-DC)	Lipid	Fatty Acid Metabolism(Acyl Carnitine)	0.06	0.38	0.80	1.00	0.78
adrenoylcarnitine (C22:4)*	Lipid	Fatty Acid Metabolism(Acyl Carnitine)	-0.06	0.52	0.88	1.00	0.44
alpha-ketobutyrate	Amino Acid	Methionine, Cysteine, SAM and Taurine Metabolism	-0.04	0.75	0.93	1.00	0.23
andro steroid monosulfate C19H28O6S (1)*	Lipid	Androgenic Steroids	-0.12	0.95	0.98	1.00	0.15
androstenediol (3beta,17beta) monosulfate (2)	Lipid	Androgenic Steroids	-0.32	0.02	0.36	1.00	1.42
androsterone glucuronide	Lipid	Androgenic Steroids	-0.25	0.01	0.29	1.00	1.53
arabinose	Carbohydrate	Pentose Metabolism	0.18	0.19	0.69	1.00	1.09
arachidoylcarnitine (C20)*	Lipid	Fatty Acid Metabolism(Acyl Carnitine)	-0.09	0.34	0.78	1.00	0.52
behenoylcarnitine (C22)*	Lipid	Fatty Acid Metabolism(Acyl Carnitine)	-0.10	0.14	0.64	1.00	0.82
benzoate	Xenobiotics	Benzoate Metabolism	-0.04	0.86	0.98	1.00	0.50
benzoylcarnitine*	Xenobiotics	Chemical	-0.13	0.62	0.90	1.00	0.90
beta-alanine	Nucleotide	Pyrimidine Metabolism, Uracil containing	-0.01	0.64	0.90	1.00	0.78
beta-cryptoxanthin	Cofactors and Vitamins	Vitamin A Metabolism	-0.03	0.36	0.79	1.00	0.74
butyrylcarnitine (C4)	Lipid	Fatty Acid Metabolism (also BCAA Metabolism)	-0.14	0.05	0.48	1.00	1.32
caffeine	Xenobiotics	Xanthine Metabolism	0.24	0.56	0.89	1.00	0.34
caprylate (8:0)	Lipid	Medium Chain Fatty Acid	0.11	0.62	0.90	1.00	0.33
carboxyethyl-GABA	Amino Acid	Glutamate Metabolism	-0.10	0.11	0.63	1.00	0.98
carotene diol (3)	Cofactors and Vitamins	Vitamin A Metabolism	-0.05	0.49	0.86	1.00	0.65
ceramide (d18:1/17:0, d17:1/18:0)*	Lipid	Ceramides	-0.10	0.04	0.45	1.00	1.35
cholate	Lipid	Primary Bile Acid Metabolism	-0.62	0.97	0.99	1.00	0.62
phosphocholine	Lipid	Phospholipid Metabolism	0.03	0.27	0.72	1.00	0.64
cinnamoylglycine	Xenobiotics	Food Component/Plant	0.03	0.91	0.98	1.00	0.23
cis-4-decenoate (10:1n6)*	Lipid	Medium Chain Fatty Acid	-0.05	0.95	0.98	1.00	0.53
corticosterone	Lipid	Corticosteroids	-0.01	0.68	0.90	1.00	0.62
cystathionine	Amino Acid	Methionine, Cysteine, SAM and Taurine Metabolism	-0.43	0.97	0.99	1.00	0.42
cysteine s-sulfate	Amino Acid	Methionine, Cysteine, SAM and Taurine Metabolism	-0.24	0.05	0.48	1.00	1.17
cysteine sulfinic acid	Amino Acid	Methionine, Cysteine, SAM and Taurine Metabolism	-0.10	0.62	0.90	1.00	0.35
cysteinylglycine	Amino Acid	Glutathione Metabolism	-0.15	0.38	0.80	1.00	0.51
cytidine	Nucleotide	Pyrimidine Metabolism, Cytidine containing	0.05	0.09	0.60	1.00	1.10
deoxycholate	Lipid	Secondary Bile Acid Metabolism	0.04	0.03	0.41	1.00	1.40
diacylglycerol (12:0/18:1, 14:0/16:1, 16:0/14:1) [1]*	Lipid	Diacylglycerol	-0.13	0.85	0.97	1.00	0.23
dihomo-linolenoylcarnitine (C20:3n3 or 6)*	Lipid	Fatty Acid Metabolism(Acyl Carnitine)	-0.05	0.27	0.72	1.00	0.79
dihomo-linoleoylcarnitine (C20:2)*	Lipid	Fatty Acid Metabolism(Acyl Carnitine)	0.03	0.26	0.72	1.00	0.84
dihydrocaffeate sulfate (2)	Xenobiotics	Food Component/Plant	0.37	0.46	0.84	1.00	0.51
docosapentaenoate (n6 DPA; 22:5n6)	Lipid	Polyunsaturated Fatty Acid (n3 and n6)	0.30	0.03	0.41	1.00	1.77
docosapentaenoylcarnitine (C22:5n3)*	Lipid	Fatty Acid Metabolism(Acyl Carnitine)	-0.08	0.94	0.98	1.00	0.33
dodecenedioate (C12:1-DC)*	Lipid	Fatty Acid, Dicarboxylate	0.29	0.40	0.82	1.00	1.08
ectoine	Xenobiotics	Chemical	-0.43	0.84	0.97	1.00	0.40
eicosapentaenoylcholine	Lipid	Fatty Acid Metabolism (Acyl Choline)	-0.14	0.91	0.98	1.00	1.12
eicosenedioate (C20:1-DC)*	Lipid	Fatty Acid, Dicarboxylate	0.02	0.46	0.84	1.00	0.39
eicosenoylcarnitine (C20:1)*	Lipid	Fatty Acid Metabolism(Acyl Carnitine)	0.01	0.94	0.98	1.00	0.12
epiandrosterone sulfate	Lipid	Androgenic Steroids	-0.33	0.02	0.37	1.00	1.23
etiocholanolone glucuronide	Lipid	Androgenic Steroids	-0.30	0.00	0.21	1.00	1.61
Fibrinopeptide A (7-16)*	Peptide	Fibrinogen Cleavage Peptide	-0.07	0.21	0.70	1.00	0.79
Fibrinopeptide A (8-16)	Peptide	Fibrinogen Cleavage Peptide	0.05	0.46	0.84	1.00	0.57
ADSGEGDFXAEGGGVR*	Peptide	Fibrinogen Cleavage Peptide	0.10	0.92	0.98	1.00	0.12
ADpSGEGDFXAEGGGVR*	Peptide	Fibrinogen Cleavage Peptide	0.05	0.06	0.55	1.00	1.25

Fibrinopeptide B (1-11)	Peptide	Fibrinogen Cleavage Peptide	-0.24	0.05	0.48	1.00	1.21
Fibrinopeptide B (1-9)	Peptide	Fibrinogen Cleavage Peptide	0.00	0.73	0.93	1.00	0.56
fumarate	Energy	TCA Cycle	0.15	0.02	0.37	1.00	1.61
galactonate	Carbohydrate	Fructose, Mannose and Galactose Metabolism	0.06	0.56	0.89	1.00	0.36
gamma-glutamyl-2-aminobutyrate	Peptide	Gamma-glutamyl Amino Acid	0.07	0.95	0.98	1.00	0.83
gamma-glutamylalanine	Peptide	Gamma-glutamyl Amino Acid	0.06	0.14	0.64	1.00	1.29
gamma-glutamyl-epsilon-lysine	Peptide	Gamma-glutamyl Amino Acid	-0.07	0.67	0.90	1.00	0.84
gamma-glutamylisoleucine*	Peptide	Gamma-glutamyl Amino Acid	-0.04	0.33	0.78	1.00	0.86
gamma-glutamyltryptophan	Peptide	Gamma-glutamyl Amino Acid	-0.02	1.00	1.00	1.00	0.62
gamma-glutamyltyrosine	Peptide	Gamma-glutamyl Amino Acid	0.01	0.67	0.90	1.00	0.48
gentisate	Amino Acid	Tyrosine Metabolism	1.01	0.20	0.69	1.00	1.10
glucuronide of C10H18O2 (7)*	Partially Characterized Molecu	Partially Characterized Molecules	0.80	0.19	0.69	1.00	1.44
glucuronide of piperine metabolite C17H21NO3 (3)*	Xenobiotics	Food Component/Plant	-0.08	0.56	0.89	1.00	0.87
glucuronide of piperine metabolite C17H21NO3 (4)*	Xenobiotics	Food Component/Plant	-0.15	0.76	0.93	1.00	0.50
glucuronide of piperine metabolite C17H21NO3 (5)*	Xenobiotics	Food Component/Plant	-0.08	0.66	0.90	1.00	0.63
glu-gly-asn-val	Peptide	Polypeptide	0.21	0.45	0.84	1.00	0.50
glutarate (C5-DC)	Lipid	Fatty Acid, Dicarboxylate	0.32	0.13	0.64	1.00	1.03
glycerol 3-phosphate	Lipid	Glycerolipid Metabolism	-0.08	0.88	0.98	1.00	0.35
glycerophosphoethanolamine	Lipid	Phospholipid Metabolism	-0.11	0.84	0.97	1.00	0.96
glycerophosphoinositol*	Lipid	Phospholipid Metabolism	-0.29	0.17	0.69	1.00	1.04
glycine conjugate of C10H14O2 (1)*	Partially Characterized Molecu	Partially Characterized Molecules	-0.13	0.41	0.83	1.00	0.53
glyco-beta-muricholate	Lipid	Primary Bile Acid Metabolism	0.11	0.09	0.61	1.00	1.09
glycochenodeoxycholate 3-sulfate	Lipid	Primary Bile Acid Metabolism	0.08	0.72	0.92	1.00	0.32
glycochenodeoxycholate glucuronide (1)	Lipid	Primary Bile Acid Metabolism	-0.21	0.68	0.90	1.00	0.51
glycodeoxycholate	Lipid	Secondary Bile Acid Metabolism	0.76	0.01	0.35	1.00	1.53
glycodeoxycholate 3-sulfate	Lipid	Secondary Bile Acid Metabolism	0.09	0.40	0.81	1.00	0.50
glycohyocholate	Lipid	Secondary Bile Acid Metabolism	-0.05	0.20	0.69	1.00	1.00
glycolithocholate sulfate*	Lipid	Secondary Bile Acid Metabolism	0.19	0.09	0.61	1.00	1.10
glycosyl ceramide (d18:1/20:0, d16:1/22:0)*	Lipid	Hexosylceramides (HCER)	-0.15	0.18	0.69	1.00	0.96
glycosyl ceramide (d18:1/23:1, d17:1/24:1)*	Lipid	Hexosylceramides (HCER)	0.05	0.92	0.98	1.00	0.25
glycosyl-N-(2-hydroxynervonoyl)-sphingosine (d18:1/24:1(2C	Lipid	Hexosylceramides (HCER)	-0.17	0.02	0.36	1.00	1.54
glycosyl-N-behenoyl-sphingadienine (d18:2/22:0)*	Lipid	Hexosylceramides (HCER)	-0.01	0.36	0.79	1.00	0.61
glycosyl-N-tricosanoyl-sphingadienine (d18:2/23:0)*	Lipid	Hexosylceramides (HCER)	-0.03	0.24	0.72	1.00	0.71
glycylvaline	Peptide	Dipeptide	0.52	0.72	0.92	1.00	0.25
guanidinosuccinate	Amino Acid	Guanidino and Acetamido Metabolism	-0.14	0.65	0.90	1.00	0.73
guanosine	Nucleotide	Purine Metabolism, Guanine containing	0.01	0.64	0.90	1.00	0.31
heneicosapentaenoate (21:5n3)	Lipid	Polyunsaturated Fatty Acid (n3 and n6)	-0.26	0.03	0.41	1.00	2.02
heptenedioate (C7:1-DC)*	Lipid	Fatty Acid, Dicarboxylate	-0.09	0.26	0.72	1.00	0.73
hexanoylglutamine	Lipid	Fatty Acid Metabolism (Acyl Glutamine)	0.28	0.15	0.66	1.00	0.94
hexanoylglycine (C6)	Lipid	Fatty Acid Metabolism(Acyl Glycine)	0.08	0.59	0.90	1.00	0.95
homocitrulline	Amino Acid	Urea cycle; Arginine and Proline Metabolism	0.04	0.50	0.86	1.00	0.59
homovanillate (HVA)	Amino Acid	Tyrosine Metabolism	-0.01	0.84	0.97	1.00	0.38
hydantoin-5-propionate	Amino Acid	Histidine Metabolism	-0.14	0.12	0.63	1.00	1.05
hydroquinone sulfate	Xenobiotics	Drug - Topical Agents	-0.14	0.34	0.78	1.00	0.59
hyocholate	Lipid	Secondary Bile Acid Metabolism	-0.51	0.95	0.98	1.00	0.57
imidazole propionate	Amino Acid	Histidine Metabolism	0.36	0.24	0.71	1.00	1.13
indole-3-carboxylate	Amino Acid	Tryptophan Metabolism	0.05	0.60	0.90	1.00	0.64
indoleacetylcarnitine*	Xenobiotics	Chemical	-0.16	0.20	0.69	1.00	0.76

indoleacetylglutamine	Amino Acid	Tryptophan Metabolism	0.15	0.84	0.97	1.00	0.75
indolepropionate	Amino Acid	Tryptophan Metabolism	0.69	0.97	0.99	1.00	0.49
isobutyrylglycine (C4)	Amino Acid	Leucine, Isoleucine and Valine Metabolism	-0.03	0.52	0.88	1.00	0.57
isoleucylglycine	Peptide	Dipeptide	-0.09	0.17	0.69	1.00	0.81
isoursodeoxycholate	Lipid	Secondary Bile Acid Metabolism	0.05	0.28	0.73	1.00	0.55
isovalerate (C5)	Amino Acid	Leucine, Isoleucine and Valine Metabolism	-0.06	0.56	0.89	1.00	0.61
isovalerylcarnitine (C5)	Amino Acid	Leucine, Isoleucine and Valine Metabolism	-0.17	0.01	0.28	1.00	1.57
isovalerylglycine	Amino Acid	Leucine, Isoleucine and Valine Metabolism	-0.13	0.17	0.69	1.00	1.08
lactosyl-N-behenoyl-sphingosine (d18:1/22:0)*	Lipid	Lactosylceramides (LCER)	-0.09	0.47	0.85	1.00	0.42
lanthionine	Amino Acid	Methionine, Cysteine, SAM and Taurine Metabolism	0.07	0.65	0.90	1.00	0.51
leucylalanine	Peptide	Dipeptide	0.00	0.72	0.92	1.00	0.30
linoleoyl ethanolamide	Lipid	Endocannabinoid	0.05	0.95	0.98	1.00	1.00
linoleoyl-arachidonoyl-glycerol (18:2/20:4) [1]*	Lipid	Diacylglycerol	0.06	0.90	0.98	1.00	0.25
linoleoyl-docosahexaenoyl-glycerol (18:2/22:6) [2]*	Lipid	Diacylglycerol	-0.13	1.00	1.00	1.00	0.11
lithocholate sulfate (1)	Lipid	Secondary Bile Acid Metabolism	0.09	0.18	0.69	1.00	0.78
maleate	Lipid	Fatty Acid, Dicarboxylate	0.63	0.03	0.41	1.00	1.42
malonate	Lipid	Fatty Acid Synthesis	-0.01	0.88	0.98	1.00	0.19
methyl indole-3-acetate	Xenobiotics	Food Component/Plant	0.21	0.44	0.84	1.00	0.81
methyl-4-hydroxybenzoate sulfate	Xenobiotics	Benzoate Metabolism	-0.35	0.30	0.75	1.00	0.50
methylmalonate (MMA)	Lipid	Fatty Acid Metabolism (also BCAA Metabolism)	0.24	0.25	0.72	1.00	0.93
N-(2-furoyl)glycine	Xenobiotics	Food Component/Plant	0.47	0.25	0.72	1.00	1.24
N2,N5-diacetylornithine	Amino Acid	Urea cycle; Arginine and Proline Metabolism	-0.03	0.30	0.75	1.00	0.67
N2-acetyl,N6-methyllysine	Amino Acid	Lysine Metabolism	-0.04	0.43	0.83	1.00	0.57
N4-acetylcytidine	Nucleotide	Pyrimidine Metabolism, Cytidine containing	0.01	0.79	0.94	1.00	0.39
N6-carbamoylthreonyladenosine	Nucleotide	Purine Metabolism, Adenine containing	0.00	0.66	0.90	1.00	1.18
N6-succinyladenosine	Nucleotide	Purine Metabolism, Adenine containing	0.08	0.52	0.88	1.00	0.71
N-acetyl-1-methylhistidine*	Amino Acid	Histidine Metabolism	0.04	0.59	0.90	1.00	0.31
N-acetylaspartate (NAA)	Amino Acid	Alanine and Aspartate Metabolism	0.12	0.95	0.98	1.00	0.17
N-acetylcarnosine	Amino Acid	Histidine Metabolism	-0.35	0.00	0.17	1.00	1.92
N-acetylcitrulline	Amino Acid	Urea cycle; Arginine and Proline Metabolism	-0.06	0.16	0.69	1.00	0.87
N-acetylglucosamine/N-acetylgalactosamine	Carbohydrate	Aminosugar Metabolism	0.00	0.75	0.93	1.00	0.69
N-acetylhistidine	Amino Acid	Histidine Metabolism	-0.04	0.46	0.84	1.00	0.46
N-acetylisoleucine	Amino Acid	Leucine, Isoleucine and Valine Metabolism	0.09	0.48	0.86	1.00	0.94
N-acetylleucine	Amino Acid	Leucine, Isoleucine and Valine Metabolism	-0.03	0.95	0.98	1.00	0.83
N-acetylneuraminate	Carbohydrate	Aminosugar Metabolism	0.10	0.68	0.90	1.00	0.59
N-acetylphenylalanine	Amino Acid	Phenylalanine Metabolism	0.01	0.61	0.90	1.00	0.24
N-acetylthreonine	Amino Acid	Glycine, Serine and Threonine Metabolism	0.02	0.43	0.83	1.00	1.43
N-acetyltyrosine	Amino Acid	Tyrosine Metabolism	-0.02	0.42	0.83	1.00	0.71
N-carbamoylvaline	Amino Acid	Leucine, Isoleucine and Valine Metabolism	0.07	0.60	0.90	1.00	0.63
nervonoylcarnitine (C24:1)*	Lipid	Fatty Acid Metabolism(Acyl Carnitine)	-0.05	0.35	0.79	1.00	0.70
N-formylanthranilic acid	Amino Acid	Tryptophan Metabolism	-0.07	0.09	0.61	1.00	1.05
nisinate (24:6n3)	Lipid	Polyunsaturated Fatty Acid (n3 and n6)	-0.33	0.34	0.78	1.00	0.52
N-methylhydroxyproline	Amino Acid	Urea cycle; Arginine and Proline Metabolism	-0.94	0.21	0.70	1.00	1.46
N-oleoyltaurine	Lipid	Endocannabinoid	0.18	0.01	0.31	1.00	1.43
N-palmitoylserine	Lipid	Endocannabinoid	-0.01	0.13	0.64	1.00	0.99
N-palmitoyl-sphingadienine (d18:2/16:0)*	Lipid	Ceramides	0.00	0.45	0.84	1.00	0.39
N-stearoylserine*	Lipid	Endocannabinoid	0.20	0.03	0.41	1.00	1.66
N-stearoyl-sphinganine (d18:0/18:0)*	Lipid	Dihydroceramides	-0.26	0.09	0.61	1.00	1.14

N-stearoyltaurine	Lipid	Endocannabinoid	0.11	0.92	0.98	1.00	0.23
o-cresol sulfate	Xenobiotics	Benzoate Metabolism	-0.40	0.13	0.64	1.00	0.82
oleoyl-arachidonoyl-glycerol (18:1/20:4) [1]*	Lipid	Diacylglycerol	0.00	0.49	0.86	1.00	0.56
oleoyl-linolenoyl-glycerol (18:1/18:3) [2]*	Lipid	Diacylglycerol	-0.04	0.37	0.80	1.00	0.68
orotidine	Nucleotide	Pyrimidine Metabolism, Orotate containing	1.58	0.13	0.64	1.00	1.09
palmitoleoyl-oleoyl-glycerol (16:1/18:1) [2]*	Lipid	Diacylglycerol	0.49	0.00	0.17	1.00	1.87
palmitoloelycholine	Lipid	Fatty Acid Metabolism (Acyl Choline)	0.05	0.19	0.69	1.00	1.18
palmitoyl-arachidonoyl-glycerol (16:0/20:4) [1]*	Lipid	Diacylglycerol	0.06	0.09	0.61	1.00	1.19
palmitoyl-arachidonoyl-glycerol (16:0/20:4) [2]*	Lipid	Diacylglycerol	0.03	0.57	0.89	1.00	0.32
palmitoyl-docosahexaenoyl-glycerol (16:0/22:6) [1]*	Lipid	Diacylglycerol	-0.11	0.91	0.98	1.00	0.11
palmitoyl-linolenoyl-glycerol (16:0/18:3) [2]*	Lipid	Diacylglycerol	0.02	0.17	0.69	1.00	1.24
palmitoyl-linoleoyl-glycerol (16:0/18:2) [1]*	Lipid	Diacylglycerol	-0.02	0.25	0.72	1.00	0.60
palmitoyl-linoleoyl-glycerol (16:0/18:2) [2]*	Lipid	Diacylglycerol	-0.01	0.77	0.93	1.00	0.52
palmitoyl-myristoyl-glycerol (16:0/14:0) [2]	Lipid	Diacylglycerol	-0.11	0.64	0.90	1.00	0.36
p-cresol glucuronide*	Amino Acid	Tyrosine Metabolism	0.31	0.74	0.93	1.00	0.17
phenylacetate	Amino Acid	Phenylalanine Metabolism	0.28	0.74	0.93	1.00	0.51
phenylacetylcarnitine	Peptide	Acetylated Peptides	0.09	0.87	0.98	1.00	0.59
phenylacetylglutamate	Peptide	Acetylated Peptides	0.09	0.76	0.93	1.00	0.51
phenyllactate (PLA)	Amino Acid	Phenylalanine Metabolism	0.02	0.63	0.90	1.00	0.96
phosphate	Energy	Oxidative Phosphorylation	0.08	0.44	0.84	1.00	0.91
phosphoethanolamine (PE)	Lipid	Phospholipid Metabolism	0.08	0.76	0.93	1.00	0.45
picolinate	Amino Acid	Tryptophan Metabolism	0.03	0.19	0.69	1.00	0.72
pregnenolone sulfate	Lipid	Pregnenolone Steroids	-0.27	0.03	0.41	1.00	1.23
prolylhydroxyproline	Amino Acid	Urea cycle; Arginine and Proline Metabolism	-0.09	0.55	0.89	1.00	0.35
pyrraline	Xenobiotics	Food Component/Plant	-0.03	0.88	0.98	1.00	0.85
ribulonate/xylulonate*	Carbohydrate	Pentose Metabolism	0.11	0.75	0.93	1.00	0.22
S-1-pyrroline-5-carboxylate	Amino Acid	Glutamate Metabolism	0.01	0.78	0.94	1.00	0.95
S-adenosylhomocysteine (SAH)	Amino Acid	Methionine, Cysteine, SAM and Taurine Metabolism	0.07	0.13	0.64	1.00	1.41
serotonin	Amino Acid	Tryptophan Metabolism	0.04	0.71	0.92	1.00	0.57
sphingomyelin (d18:1/20:2, d18:2/20:1, d16:1/22:2)*	Lipid	Sphingomyelins	-0.04	0.17	0.69	1.00	0.81
stachydrine	Xenobiotics	Food Component/Plant	0.14	0.62	0.90	1.00	0.54
stearoyl-arachidonoyl-glycerol (18:0/20:4) [2]*	Lipid	Diacylglycerol	0.09	0.06	0.52	1.00	1.64
suberate (C8-DC)	Lipid	Fatty Acid, Dicarboxylate	0.25	0.08	0.60	1.00	1.01
suberoylcarnitine (C8-DC)	Lipid	Fatty Acid Metabolism(Acyl Carnitine)	0.24	0.63	0.90	1.00	0.33
succinylcarnitine (C4)	Energy	TCA Cycle	-0.08	0.86	0.98	1.00	1.16
sucrose	Carbohydrate	Disaccharides and Oligosaccharides	0.61	0.18	0.69	1.00	1.07
sulfate of piperine metabolite C16H19NO3 (2)*	Xenobiotics	Food Component/Plant	-0.02	0.98	1.00	1.00	0.62
sulfate of piperine metabolite C16H19NO3 (3)*	Xenobiotics	Food Component/Plant	0.00	0.41	0.83	1.00	1.10
tartarate	Xenobiotics	Food Component/Plant	-0.12	0.49	0.86	1.00	0.43
taurochenodeoxycholate	Lipid	Primary Bile Acid Metabolism	0.44	0.05	0.48	1.00	1.58
taurodeoxycholate	Lipid	Secondary Bile Acid Metabolism	1.00	0.01	0.25	1.00	1.62
taurodeoxycholic acid 3-sulfate	Lipid	Secondary Bile Acid Metabolism	0.15	0.35	0.79	1.00	0.70
taurolithocholate 3-sulfate	Lipid	Secondary Bile Acid Metabolism	0.16	0.21	0.70	1.00	1.01
threonylphenylalanine	Peptide	Dipeptide	-0.02	0.93	0.98	1.00	0.24
thymol sulfate	Xenobiotics	Food Component/Plant	0.42	0.44	0.84	1.00	0.50
tiglyl carnitine (C5)	Amino Acid	Leucine, Isoleucine and Valine Metabolism	-0.08	0.04	0.44	1.00	1.10
trans-urocanate	Amino Acid	Histidine Metabolism	-0.06	0.26	0.72	1.00	0.65
triethanolamine	Xenobiotics	Chemical	0.02	0.53	0.89	1.00	0.58

tryptophan betaine	Amino Acid	Tryptophan Metabolism	-0.40	0.20	0.69	1.00	0.72
tyramine O-sulfate	Amino Acid	Tyrosine Metabolism	-0.98	0.87	0.98	1.00	0.67
vanillactate	Amino Acid	Tyrosine Metabolism	0.96	0.84	0.97	1.00	0.44
xanthosine	Nucleotide	Purine Metabolism, (Hypo)Xanthine/Inosine containing	0.14	0.89	0.98	1.00	0.53
xylose	Carbohydrate	Pentose Metabolism	0.32	0.14	0.65	1.00	1.18
X - 07765			-0.54	0.39	0.81	1.00	0.42
X - 11299			-0.43	0.42	0.83	1.00	0.48
X - 11315			-0.10	0.22	0.70	1.00	1.05
X - 11378			-0.06	0.45	0.84	1.00	0.48
X - 11407			0.08	0.77	0.93	1.00	0.78
X - 11441			-0.06	0.39	0.80	1.00	0.52
X - 11442			-0.05	0.52	0.88	1.00	0.47
X - 11478			-0.01	0.51	0.87	1.00	0.45
X - 11483			-0.36	0.38	0.80	1.00	0.58
X - 11491			-0.39	0.11	0.63	1.00	1.02
X - 11522			0.07	0.65	0.90	1.00	0.74
X - 11843			0.41	0.41	0.83	1.00	0.61
X - 11850			0.47	0.17	0.69	1.00	0.99
X - 11852			-0.35	0.03	0.41	1.00	1.29
X - 11880			0.15	0.20	0.69	1.00	1.06
X - 12007			0.06	0.61	0.90	1.00	0.97
X - 12013			0.44	0.48	0.86	1.00	0.52
X - 12111			0.18	0.79	0.94	1.00	0.63
X - 12117			0.21	0.07	0.57	1.00	1.50
X - 12126			-0.22	0.11	0.63	1.00	0.95
X - 12170			-0.08	0.09	0.61	1.00	1.11
X - 12212			-0.20	0.09	0.60	1.00	1.05
X - 12216			-0.09	0.78	0.94	1.00	0.66
X - 12230			0.44	0.89	0.98	1.00	0.76
X - 12283			0.04	0.61	0.90	1.00	0.31
X - 12544			-0.46	0.43	0.84	1.00	0.55
X - 12680			-0.19	0.27	0.72	1.00	0.77
X - 12707			0.18	0.35	0.79	1.00	0.91
X - 12718			0.01	0.69	0.91	1.00	0.54
X - 12798			-0.09	0.64	0.90	1.00	0.40
X - 12812			-0.41	0.59	0.90	1.00	1.00
X - 12816			0.26	0.46	0.84	1.00	0.84
X - 12822			-0.06	0.81	0.95	1.00	0.72
X - 12847			-0.04	0.63	0.90	1.00	0.84
X - 12849			-0.55	0.62	0.90	1.00	0.27
X - 12851			-0.23	0.46	0.84	1.00	0.51
X - 13507			-0.09	0.91	0.98	1.00	0.92
X - 13684			0.03	0.79	0.94	1.00	0.46
X - 13728			0.17	0.87	0.98	1.00	0.12
X - 13729			0.03	0.54	0.89	1.00	1.08
X - 13737			-0.12	0.07	0.57	1.00	1.10
X - 13835			-0.12	0.60	0.90	1.00	0.53
X - 13844			0.10	0.44	0.84	1.00	0.90

X - 15461	0.06	0.91	0.98	1.00	0.78
X - 15666	0.07	0.63	0.90	1.00	0.73
X - 15674	-0.05	0.11	0.63	1.00	1.08
X - 15728	-0.22	0.60	0.90	1.00	0.42
X - 16397	-0.01	0.57	0.89	1.00	0.38
X - 16570	-0.13	0.32	0.78	1.00	0.64
X - 16654	0.14	0.36	0.79	1.00	0.48
X - 16946	0.06	0.96	0.99	1.00	0.04
X - 16964	-0.01	0.92	0.98	1.00	1.04
X - 17010	0.10	0.61	0.90	1.00	0.33
X - 17145	-0.14	0.25	0.72	1.00	0.67
X - 17146	0.35	0.35	0.79	1.00	0.68
X - 17185	0.30	0.34	0.78	1.00	1.24
X - 17325	0.32	0.89	0.98	1.00	0.57
X - 17327	0.22	0.40	0.82	1.00	0.72
X - 17328	-0.37	0.55	0.89	1.00	0.51
X - 17351	0.00	0.76	0.93	1.00	0.20
X - 17354	-0.60	0.00	0.11	0.11	2.35
X - 17359	-0.04	0.35	0.79	1.00	0.95
X - 17367	0.35	0.81	0.95	1.00	0.53
X - 17438	0.41	0.92	0.98	1.00	0.42
X - 17612	0.25	0.05	0.48	1.00	1.24
X - 17653	0.04	0.88	0.98	1.00	0.62
X - 17676	-0.11	0.64	0.90	1.00	0.45
X - 18249	-0.03	0.53	0.89	1.00	0.42
X - 18345	0.43	0.93	0.98	1.00	0.65
X - 18886	0.00	0.90	0.98	1.00	0.10
X - 18899	0.11	0.38	0.80	1.00	1.17
X - 18901	0.41	0.55	0.89	1.00	0.54
X - 18914	0.01	0.33	0.78	1.00	0.56
X - 18922	0.01	0.85	0.98	1.00	0.86
X - 21339	0.05	0.95	0.98	1.00	0.63
X - 21364	-0.20	0.18	0.69	1.00	0.84
X - 21441	0.14	0.78	0.94	1.00	0.21
X - 21442	-0.04	0.93	0.98	1.00	0.68
X - 21448	0.04	0.84	0.97	1.00	0.40
X - 21467	-0.28	0.10	0.63	1.00	0.95
X - 21470	-0.26	0.90	0.98	1.00	0.37
X - 21471	-0.12	0.67	0.90	1.00	0.49
X - 21607	-0.04	0.51	0.87	1.00	0.60
X - 21729	-0.08	0.36	0.79	1.00	0.41
X - 21752	-0.14	0.04	0.47	1.00	1.15
X - 21792	0.25	0.22	0.70	1.00	0.73
X - 21815	0.26	0.26	0.72	1.00	0.62
X - 21821	-0.01	0.49	0.86	1.00	0.46
X - 21829	0.02	0.56	0.89	1.00	0.33
X - 21834	-0.25	0.92	0.98	1.00	0.87
X - 22508	-0.18	0.25	0.72	1.00	0.70

X - 22509	-0.43	0.00	0.17	1.00	1.85
X - 22771	-0.02	0.44	0.84	1.00	1.05
X - 23196	0.30	0.19	0.69	1.00	0.77
X - 23276	0.05	0.29	0.74	1.00	1.22
X - 23296	0.00	0.91	0.98	1.00	0.64
X - 23297	0.07	0.67	0.90	1.00	0.60
X - 23369	0.01	0.95	0.98	1.00	1.52
X - 23585	-0.05	0.63	0.90	1.00	0.87
X - 23587	-0.24	0.19	0.69	1.00	0.77
X - 23641	-0.19	0.02	0.36	1.00	1.47
X - 23644	-1.01	0.05	0.48	1.00	1.17
X - 23659	-0.03	0.23	0.70	1.00	0.67
X - 23665	-0.07	0.70	0.91	1.00	0.43
X - 23666	-0.14	0.21	0.70	1.00	0.72
X - 23680	-0.03	0.91	0.98	1.00	0.49
X - 23739	-0.06	0.29	0.75	1.00	0.65
X - 23782	-0.18	0.11	0.63	1.00	1.41
X - 23997	0.10	0.58	0.89	1.00	0.64
X - 24243	-0.30	0.67	0.90	1.00	0.56
X - 24293	0.30	0.97	0.99	1.00	0.09
X - 24295	-0.14	0.27	0.73	1.00	0.90
X - 24328	-0.15	0.32	0.78	1.00	0.59
X - 24337	0.13	0.55	0.89	1.00	0.58
X - 24455	0.12	0.56	0.89	1.00	0.99
X - 24473	-0.51	0.60	0.90	1.00	0.54
X - 24475	-0.28	0.10	0.63	1.00	0.95
X - 24494	-0.05	0.98	1.00	1.00	0.36
X - 24527	0.12	0.37	0.80	1.00	0.53
X - 24542	-0.33	0.11	0.63	1.00	0.99
X - 24544	-0.18	0.14	0.64	1.00	0.86
X - 24556	-0.10	0.28	0.73	1.00	0.68
X - 24571	-0.03	0.77	0.93	1.00	0.54
X - 24588	0.05	0.81	0.95	1.00	0.99
X - 24686	-0.01	0.57	0.89	1.00	0.96
X - 24748	0.08	0.66	0.90	1.00	0.51
X - 24757	0.18	0.61	0.90	1.00	0.49
X - 24809	0.19	0.62	0.90	1.00	0.35
X - 24811	0.18	0.62	0.90	1.00	0.63
X - 24813	-0.06	0.10	0.62	1.00	1.05
X - 24849	0.05	0.65	0.90	1.00	0.62
X - 24947	-0.17	0.17	0.69	1.00	0.77
X - 24949	0.14	0.44	0.84	1.00	0.57
X - 24951	0.01	0.27	0.72	1.00	0.68
X - 24972	-0.39	0.05	0.48	1.00	1.06
X - 25271	-0.25	0.07	0.57	1.00	1.03
X - 25343	0.01	0.46	0.84	1.00	0.91
X - 25419	-0.35	0.08	0.60	1.00	1.02
X - 25420	0.00	0.42	0.83	1.00	0.54

X - 25519	-0.07	0.69	0.91	1.00	0.48
X - 25520	0.06	0.44	0.84	1.00	0.50
